# Rapid and efficient genome-wide characterization of *Xanthomonas* TAL effector genes

**DOI:** 10.1038/srep13162

**Published:** 2015-08-14

**Authors:** Yan-Hua Yu, Ye Lu, Yong-Qiang He, Sheng Huang, Ji-Liang Tang

**Affiliations:** 1 State Key Laboratory for Conservation and Utilization of Subtropical Agro-bioresources, The Key Laboratory of Ministry of Education for Microbial and Plant Genetic Engineering, and College of Life Science and Technology, Guangxi University, 100 Daxue Road, Nanning, Guangxi 530004, China

## Abstract

*Xanthomonas* TALE transcriptional activators act as virulence or avirulence factors by activating host disease susceptibility or resistance genes. Their specificity is determined by a tandem repeat domain. Some *Xanthomonas* pathogens contain 10–30 TALEs per strain. Although TALEs play critical roles in pathogenesis, their studies have so far been limited to a few examples, due to their highly repetitive gene structure and extreme similarity among different members, which constrict sequencing and assembling. To facilitate TALE studies, we developed an efficient and rapid pipeline for genome-wide cloning of *tal* genes as many as possible from a strain. Here, we report the pipeline and its use to identify all 18 *tal* genes from a newly isolated strain of the rice pathogen *Xathomonas oryzae*. Target prediction revealed a number of potential rice targets including several notable genes such as genes encoding SWEET, WRKY, Hen1, and BAK1 proteins, which provide candidates for further experimental functional analysis of the TALEs.

*Xanthomonas* is a large genus of Gram-negative bacteria that cause disease in hundreds of plant hosts, including many economically important crops^1^. These pathogens use the type III secretion system to inject effector proteins into the plant cell, where the effectors promote disease symptoms or elicit disease resistance by diverse mechanisms^2^,^3^. The type III effectors can be grouped into different families based on sequence similarity and biochemical activity. The largest family is the TAL (transcription activator-like) (or AvrBs3/PthA) family which functions as transcriptional activators of plant genes^4^. A TAL effector (TALE) contains an N-terminal type III secretion signal, a central DNA-binding region, a nuclear localization signal, and a C-terminal transcriptional activation domain (Supplementary Fig. 1A). The DNA-binding region comprises variable numbers (from 1.5 up to over 30) of tandem 33–34 amino acid repeats that specify target nucleotide sequence. The repeats are almost identical, with variation occurring primarily at amino acids 12 and 13 in each repeat, which are referred as repeat variable di-residue (RVD). The number, sequence, and order of the RVDs across the whole repeat region of a TALE define its recognition and binding of distinctive DNA target^4^. Diverse TALEs with different RVDs may target various plant genes^4^,^5^,^6^,^7^. In addition to the importance to pathogen virulence, TALE target DNA-binding domain has been used to develop the so-called TALENs technology for high-throughput targeted genome editing^8^,^9^,^10^,^11^, which was named “Method of the Year” for 2011 by Nature Methods^12^.

The number of TALEs in *Xanthomonas* varies in different species, pathovars, and strains. The important rice pathogen *Xanthomonas oryzae* harbors more than 20 TALEs in some strains^13^. Up to date, about 100 TALE-encoding genes (*tal* genes) have been identified. Studies on the identified TALEs have revealed several target genes that confer disease resistance and/or host susceptibility^6^. Recently, a few algorithms have been developed for *in silico* TALE target predictions^14^,^15^,^16^. Even though such predictions are not flawless and the targets predicted for a given TALE could vary from script to script, the candidate targets obtained are of great value to the in-depth functional analysis of TALEs^17^. However, as a consequence of copious TALEs in a genome and their highly repetitive gene structures, TALE studies have been limited to a few examples over a long period of time. A large number of *tal* genes in *Xanthomonas* remain to be cloned and characterized. The cloning of *tal* genes from a strain could hardly be done by PCR^18^, it generally requires constructing a genomic library followed by dot blot/Southern blot-based or PCR screening^19^,^20^, which are laborious and inefficient. Furthermore, due to the tandem quasi-identical repeat units, *tal* gene sequencing has long been the bottle neck for large scale *tal* gene analysis. Unlike sequencing non-repetitive DNA molecules, sequencing the tandem repeats in *tal* genes could hardly be done neither by the conventional chromosome walking method nor using new high throughput sequencing technologies^21^. To facilitate TALE studies, we developed an efficient and rapid pipeline for genome-wide cloning of *tal* genes as many as possible from a strain, which include entire target DNA-binding repeat regions. Here, we report the pipeline and its use to identify most, if not all, of *tal* genes in a strain of *Xanthomonas oryzae* pv. *oryzae* (*Xoo*), the causal agent of rice bacterial blight, which is a destructive rice disease worldwide. Subsequent *in silico* target prediction revealed a number of potential TALE targets in rice genome, which provide candidates for further experimentally functional analysis of the TALEs.

## Results

### Estimation of the number of *tal* genes in *Xoo* strain K74 by RFLP analysis

**Figure 1:**
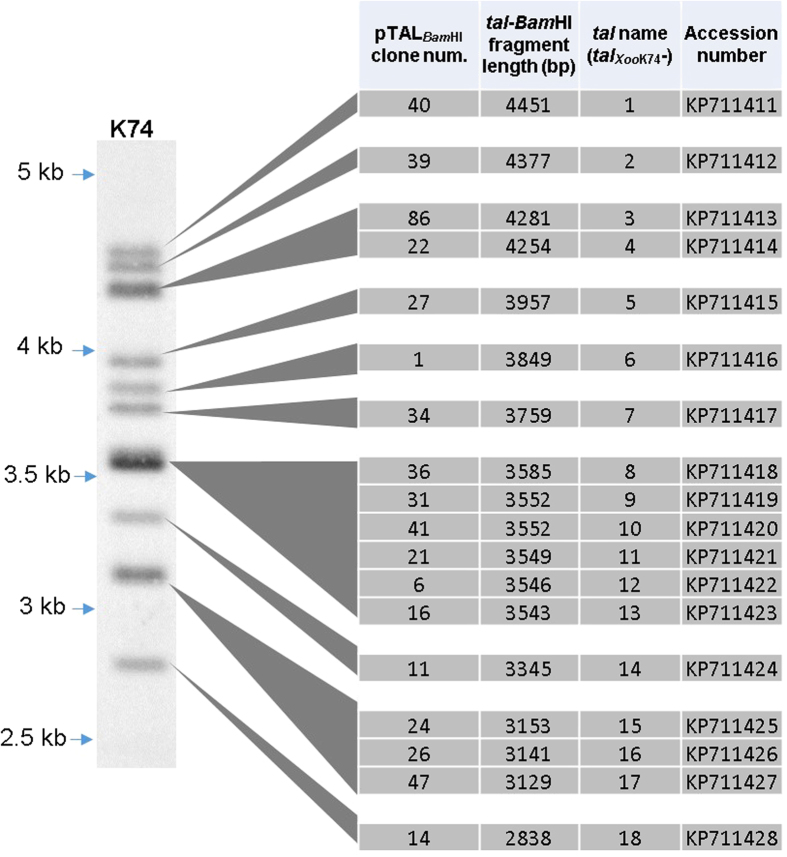
RFLP analysis of the *tal* genes in strain K74 and the correspondence between the banding pattern and the *tal*_*Xoo*K74_ genes. The genomic DNA was digested with *Bam*HI and a portion of 5′ sequence of *talC* was used as probe.

Strain K74 is a spontaneous streptomycin-resistant mutant of *Xoo* strain 1074 collected from southern coastline region of China with strong virulence to a number of rice cultivars^22^ (Supplementary Table 1). To approximate the number of *tal* genes in the strain, we performed a RFLP analysis. Since typical *tal* genes contain two conserved *Bam*HI restriction sites (Supplementary Fig. 1A), *Bam*HI was used to digest the genomic DNA of strain K74 followed by agarose gel electrophoresis. After transferred to a membrane from the gel, the DNA fragments were hybridized with labeled 500 bp N-terminal fragment of the previously identified *tal* gene *talC*^23^ (Supplementary Fig. 1A). The hybridization result showed 10 clear, visible bands with sizes ranging from 2.8–4.5 kb. Of which, seven bands displayed similar hybridization signal intensity, while three exhibited significantly stronger signal intensity (Fig. 1), indicating more than one *tal* gene copy within each of the three bands. The RFLP result suggests that the genome of strain K74 may contain more than 13 *tal* gene copies.

### Cloning of *tal*-*Bam*HI fragments containing entire target DNA-binding motif

As shown in Supplementary Figure 1A, the *Bam*HI fragment of a *tal* gene comprises a very large portion of the gene, including the entire tandem repeat region that encodes the essence for the functional specificity of TALEs. In addition, previous studies demonstrated that the *Bam*HI fragments of *tal* genes can be adapted with extraneous C-terminal transcriptional activation domain without alternating their original activities^24^. Therefore, we focused our study on the *Bam*HI fragments of *tal* genes in order to facilitate not only the subcloning and sequencing of the tandem repeats, but also to maintain the adaptation for further functional analysis. To this end, we envisioned a simple strategy with advantages in cloning massively *Bam*HI fragments of *tal* genes from strain K74 and screening unique types of *tal*-*Bam*HI fragments.

To increase cloning efficiency and facilitate the following downstream screening, the portion of *Bam*HI-digested total DNA of strain K74 with sizes ranging from 1.5 to 7.5 kb were recovered from agarose gel and cloned into the vector pUC19, followed by transformed into *E. coli* strain Trans5α. About 3,000 individual transformant colonies were collected for dot blotting with the same probe as used for RFLP analysis. In total, 115 positive clones were obtained, which were further confirmed by Southern blot (Supplementary Fig. 2). These positive clones were denoted as pTAL_*Bam*HI_ clones. Restriction analysis by *Bam*HI digestion revealed that 99 of the clones contained only one fragment, while 16 contained more than one, in addition to the vector fragment (data not shown). The Southern blot result showed that the sizes of hybridized *Bam*HI fragments were consistent with the RFLP pattern of *tal* genes in strain K74.

It is unlikely that the obtained 115 pTAL_*Bam*H1_ clones derive from the same *tal*-*Bam*HI fragment. To isolate all unique *tal*-*Bam*HI fragments from the 115 pTAL_*Bam*HI_ clones, we determined the DNA sequences of both 5′ and 3′ ends of the tandem repeat domain and their adjacent regions in all of the clones using the primers P1F and P1R (Supplementary Fig. 1B and Supplementary Table 2). Pairwise alignments were systematically performed with the experimental sequences to generate the identity matrix of Supplementary Tables 3 and 4. The clones containing *Bam*HI fragments with the same size and identical DNA sequences in both 5′ and 3′ portions of the repeat region were considered to derive from the same *tal* gene in strain K74. Based on these criterions, all pTAL_*Bam*HI_ clones were re-classified into 18 groups which represent 18 unique *tal-Bam*HI fragments. Each of the 18 unique *tal-Bam*H1 fragments was represented by at least four pTAL_*Bam*H1_ clones, suggesting that the 18 groups may cover all of the *tal* genes in strain K74. These data indicate that the 10 RFLP bands contain 18 different types of *tal* genes; in other word, strain K74 carries 18 different *tal* genes. Eighteen pTAL_*Bam*HI_ clones which represent the 18 unique *tal-Bam*HI fragments (Fig. 1) were chosen for further studies.

### Screen of a proper restriction enzyme for sequencing the tandem repeat regions

As described above, the tandem repeat regions of many *tal* genes consist of a large number of quasi identical repeat units. If using short-read technologies to determine the sequence of the repeat regions, the sequence reads would be inaccessible to be assembled. We therefore used Sanger method to sequence the 18 unique *tal*-*Bam*HI fragments. For this purpose, we screened a proper restriction enzyme that has, if at all possible, only one repeat-exclusive digestion site in each repeat unit for partial digestion and subcloning. DNA sequences of 87 *tal* genes reported previously were analyzed *in silico* and two such candidate enzymes (*Msc*I and *Bsm*BI) were found (Supplementary Table 5). Ideally, partial digestion of a tandem repeat region containing n repeat units with an enzyme should yield a ladder-like banding pattern with a range of sizes from one repeat unit to n-1 repeat units after agarose gel electrophoresis. Moreover, every band includes not only identical fragments but also fragments consisting of various repeat units. Therefore, cloning and sequencing the mixed populations of one or two bands could yield sufficient data to assemble the entire tandem repeat region. The number of recognition site of *Msc*I is in coherence with the number of repeat units of almost all of the *tal* genes tested (Supplementary Table 5). In addition, *Msc*I site is blunt-ended and absent in the vector pUC19. We thus chose *Msc*I to digest partially the *tal*-*Bam*HI fragemts for subsequent sequence determination.

**Figure 2:**
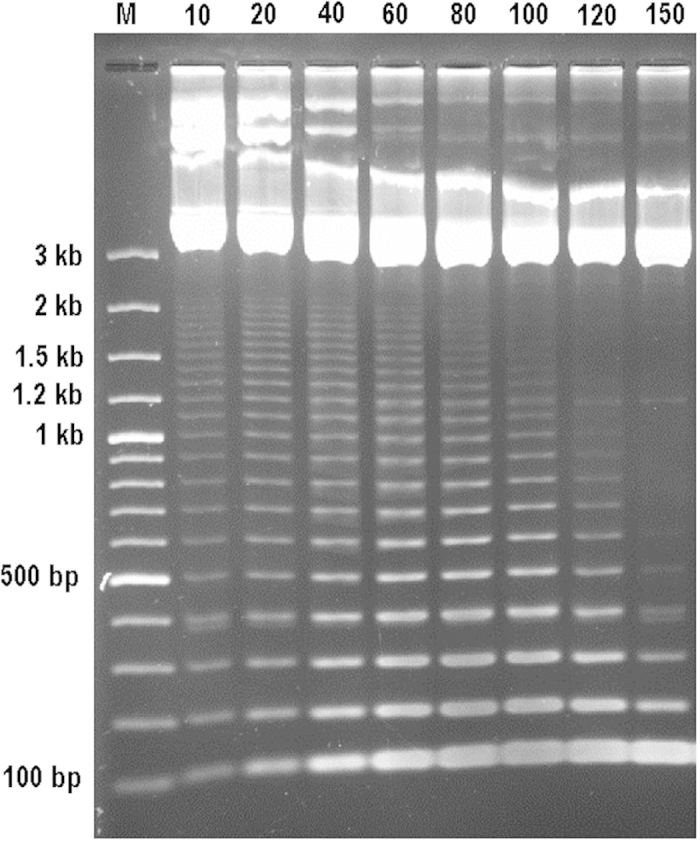
*Msc*I partial digestion of *talC-Bam*HI fragment cloned in pUC19. 2 μg plasmid DNA was digested with 5 u *Msc*I enzyme. The digestion times are given in minutes above the gel. M, 100 bp DNA ladder makers.

To test if the partial digestion is functional, the *Bam*HI fragment of *talC*^23^ was cloned into pUC19 and partially digested with *Msc*I. The digestion time varied from 10 minutes to 150 minutes while the initial quantity of plasmid DNA (2 μg) and *Msc*I (5 units) was maintained at the same level in all digestions. Agarose gel electrophoresis revealed ladder-like banding patterns as expected. As shown in Fig. 2, apart from the bands around 3 kb, which correspond to the vector and its derivatives, a total of 21 cascading bands were visible, which are consistent with the number of repeat units of *talC*. The intensity of the 100-bp band increased along the digestion course, which is consistent with the fact that the final digestion products of the repeat region are the fragments with only one repeat unit. Similarly, partial *Msc*I digestion of the 18 unique pTAL_*Bam*HI_ clones all yielded expected ladder-like banding patterns after agarose gel electrophoresis (data not shown).

### Sequencing of all 18 unique *tal*-*Bam*HI fragments of strain K74

Three additional steps were required to sequence all of the 18 unique *tal*-*Bam*HI fragments. First, both 5′ and 3′ non-repeat regions of the *tal*-*Bam*HI fragments cloned in pUC19 were sequenced using the primers M13F/M13R, P2F/P2R and P3F/P3R (Supplementary Fig. 1B and Supplementary Table 2). Second, the mixed populations of the 1-kb band generated by partial *Msc*I digestion and agarose gel electrophoresis were cloned into the vector pCC2FOS and sequenced using the primer pCC2-seqF (Supplementary Table 2) complementary to vector DNA. The number of the clones needed to be sequenced depends on the number of repeat units in the *tal*-*Bam*HI fragment. The more repeat units in the fragment, the more clones need to be sequenced. The sequencing data of the 18 unique *tal*-*Bam*HI fragments were listed in Supplementary Data 1. Finally, the *tal*-*Bam*HI fragments were *de novo* assembled by using the sequence assembly module of Geneious 7 to analyze the sequence data of the repeat region together with the DNA sequences of both 5′ and 3′ ends of the tandem repeat domain and their adjacent non-repeat regions obtained above. The scheme for DNA sequence assembling of the 18 unique *tal*-*Bam*HI fragments and the resulting DNA sequences are presented in Supplementary Figure 3 and Supplementary Data 2, respectively. The assembled DNA sequence lengths of all *tal*-*Bam*HI fragments are consistent with the sizes revealed by RFLP analysis (Fig. 1). Notably, the biggest *tal*-*Bam*HI fragment with 4,451 bp in length (No. 40) (Fig. 1) contains only 16.5 repeats, and the gene is interrupted by an *IS* element at its 3′ end. To complete the *IS* element sequence determination, chromosome walking technology was employed. *talC* was taken as a control and the assembled result showed that its *Bam*HI fragment consists of 3678 bp with 21.5 repeat units (Supplementary Fig. 4, Supplementary Data 2 and Supplementary Table 6), which is completely consistent with its published sequence^23^.

**Figure 3:**
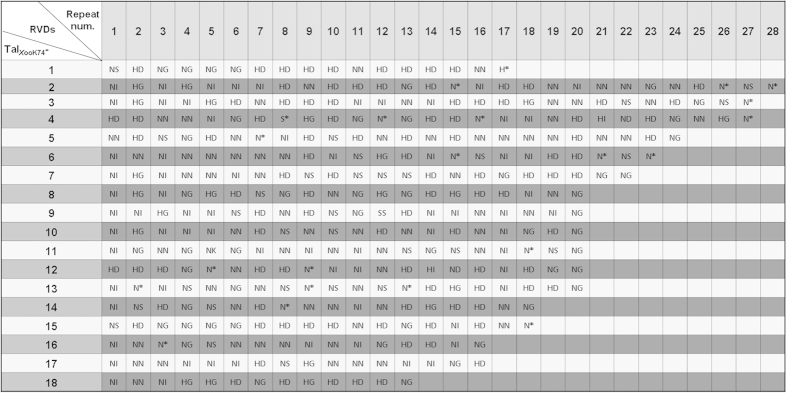
Repeat numbers and RVDs of the 18 Tal_*Xoo*K74_ effectors. The last half repeat is counted as one repeat.

According to the sizes of the *tal*-*Bam*HI fragments, the *tal* genes of *Xoo* strain K74 and their corresponding protein products were denoted as *tal*_*XooK74*_-1 to *tal*_*XooK74*_-18 (Fig. 1) and Tal_*Xoo*K74_-1 to Tal_*Xoo*K74_-18 (Fig. 3), respectively. The number of repeat units in the TALEs varies from 12.5 to 26.5 (Fig. 3) and the majority of the repeat units encode 34 amino acids. Similar to all known TALEs, the last unit in the tandem repeat motifs of all 18 Tal_*Xoo*K74_ effectors is only a half repeat (Supplementary Table 6).

### Comparison of the Tal_*Xoo*K74_ effectors with other *Xoo* TALEs and *in silico* target prediction

**Figure 4:**
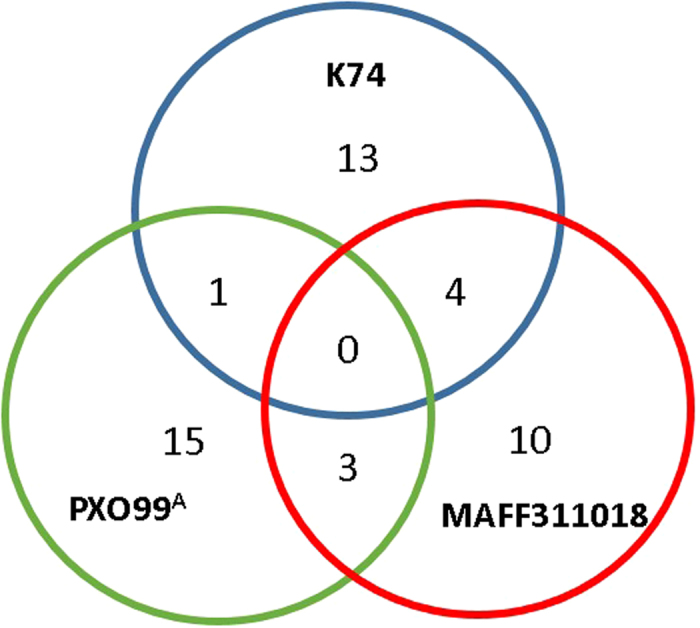
A Venn diagram illustrating the number of TALEs sharing identical RVD strings among *Xoo* strains K74, PXO99^A^ and MAFF311018.

As described above, DNA-binding specificity of TALEs to plant genes is determined by their RVD strings in the repeat motifs. To gain some general knowledge on the composition features and potential functions of the RVDs in the Tal_*Xoo*K74_ effectors, we compared them with those in the TALEs identified from the Philippine strain PXO99^A^ and the Japanese strain MAFF311018^25^,^26^. The result showed that the RVDs of Tal_*Xoo*K74_-1 and Tal_*Xoo*K74_-15 are similar to each other, and the RVDs of one (Tal_*Xoo*K74_-12) and four (Tal_*Xoo*K74_-3, Tal_*Xoo*K74_-11, Tal_*Xoo*K74_-13, and Tal_*Xoo*K74_-17) K74 TALEs are identical to those of PXO99^A^ and MAFF311018 TALEs, respectively, and no TALE is identical among the three strains (Fig. 4; Supplementary Table 7). In total, there are 13 TALEs which are similar among the three strains and may compose a set of core TALEs of the *Xoo* strains (Supplementary Table 7). Interestingly, the RVD strings of Tal_*Xoo*K74_-4 and Tal_*Xoo*K74_-16 are very similar to those of the TALEs AvrXa23 and AvrXa27 identified in PXO99^A^, respectively (Supplementary Table 7), which are avirulence determinants conferring incompatibility on rice cultivars harboring the *R* genes *Xa23* and *Xa27*, respectively^22^.

As mentioned above, several algorithms have been developed for *in silico* TALE target prediction. To gain an insight into potential roles of the 18 Tal_*Xoo*K74_ effectors in the interaction between strain K74 and its rice host, we employed one of such algorithms, TALgetter 1.0^15^ to search rice promoterome (1 kb sequences upstream all the translation start sites) for latent targets of the TALEs. Table 1 lists some of the top score candidate targets predicted for the Tal_*Xoo*K74_ effectors. The top ten high score predicted targets for each Tal_*Xoo*K74_ effector were collected and listed in Supplementary Table 8. The predicted targets include several remarkable genes such as *Os11N3* (*Os11g31190*), *Os01g61080*, *Os12g18110, Os08g07760*, and *Os07g06970*, which were predicted to be potential targets for Tal_*Xoo*K74_-2, Tal_*Xoo*K74_-3, Tal_*Xoo*K74_-4, Tal_*Xoo*K74_-10, and Tal_*Xoo*K74_-12, respectively. *Os11N3* encodes a member of the SWEET sucrose-efflux transporter family. It has been demonstrated that *Xoo* activates the gene by the TALEs AvrXa7 and TalC, and thus diverts sugars from the plant cell so as to gratify the pathogen’s nutritional needs and enhances its virulence^24^,^27^. The predicted recognition site of Tal_*Xoo*K74_-2 is 232 bp upstream of the start codon of *Os11N3*, which is different but overlapping with those recognized by AvrXa7 and its variants. The RVD string of Tal_*Xoo*K74_-2 differs mainly in additional “NI” and “HG” at the N-terminal from AvrXa7 and its variants (Supplementary Table 7). *Os01g61080* encodes the rice WRKY transcription factor OsWRKY24. Plant WRKY transcription factors are known to be involved in response to bacterial and environmental stresses via interactions with plant hormones. Interestingly, over expression of WRKY33 in *Arabidopsis*, which is an ortholog of rice OsWRKY24, can not only enhance resistance against necrotrophic fungal pathogenes, but also increase susceptibility to the bacterial pathogen *Pseudomonas syringae*^28^. *Os08g07760* encodes a brassinosteroid insensitive 1-associated receptor kinase 1 (BAK1). BAK1 was originally discovered as a coreceptor for the brassinosteroid receptor, and later studies revealed that BAK1 is involved in recognizing the pathogen-associated molecular patterns (PAMPs) and thus leads to the induction of PAMP-triggered immunity (PTI)^29^. It has been demonstrated that several non-TAL effectors of *Pseudomonas* target BAK1, resulting in an abolishment of the pattern recognition receptor (PRR)-PAMPs recognition and signaling transduction^30^. *Os12g18110* which encodes a PQ-loop repeat domain-containing protein is in the top list of Tal_*Xoo*K74_-4 predicted targets. PQ-loop proteins may function as transporters of amino acids and other solutes across membranes^31^. *Os07g06970* encodes Hen1 which is a methyltransferase that modifies the 3′ terminal nucleoside of small regulatory RNAs and increases their stabilities. Previous prediction using another algorithm showed that *hen1* is a potential target of the TALE Tal9a identified in PXO99^A^ strain^16^. As the RVD strings of Tal_*Xoo*K74_-12 and Tal9a are identical (Supplementary Table 7), it is not surprising that they share a common target. Although further experiments are needed to evaluate all the potential targets, the predictions provide us with useful clues for studies on the functions of the Tal_*Xoo*K74_ effectors.

## Discussion

**Figure 5:**
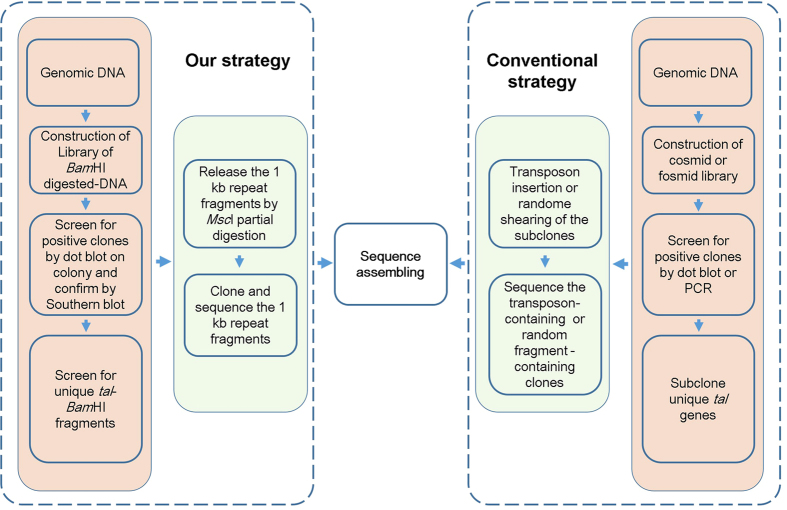
A flow chart diagram illustrating the steps in the pipeline and in conventional methods.

Here, we report a simple and convenient method for genome-wide identification of *Xanthomonas tal* genes. To meet the challenges of highly conserved *tal* gene structures and their large tandem repeat segments, divers strategies have been used to determine accurately the DNA sequences of the repeat region; however, most of the strategies rely on the use of transposon-based or random DNA shearing sequencing technologies^19^,^20^, which are cumbersome and expensive. The pipeline described in this paper aimed to overcome these drawbacks with simple experiments and approaches. Most experiments involved in the pipeline are standard routine experiments in many laboratories. The method is efficient, rapid, and economical. In our laboratory, a well-trained technician can complete the pipeline to identify *tal* genes genome-widely from one or two *Xoo* strains in less than one month. A flow chart diagram illustrating the steps in the pipeline is shown in Fig. 5.

In the pipeline, there are two key steps for *tal* gene sequencing. The first one is the screening of a proper restriction enzyme for partial digestion of the *tal*-*Bam*HI fragments. An ideal enzyme should have only one repeat-exclusive restriction site within every repeat unit, and if not such perfect enzyme available, restriction enzymes with a single recognition site in each of the most repeat units are sufficient for subsequent sequencing and assembling. Use of an enzyme with too many recognition sites in the repeats will increase the sequencing and assembling-associated workload. As shown in Supplementary Table 5, in addition to *Msc*I, *Bsm*BI is also a good candidate. Another key step is the sequencing of the mixed populations of the 1-kb fragments generated by partial digestion with the restriction enzyme selected. Each repeat unit in a *tal* gene consists of about 100 bp (encoding 33–34 aa) so that an 1-kb fragment should include about 10 repeat units. The mixed 1-kb fragments comprise all possible combinations of different 10-tandem-repeat-unit fragments. Therefore, sequencing and assembling of the mixed 1-kb fragment populations will reveal the entire tandem repeats in a *tal* gene. Since the normal credible read length obtained by Sanger sequencing method is around 1-kb, choosing the 1-kb fragments can minimize the sequencing cost while maintaining a high efficiency in assembling.

By the method, we were able to dissect genome-widely the *tal* genes of the newly isolated *Xoo* strain K74 without any prior genome sequence information. Since each of the 18 unique *tal-Bam*H1 fragments was represented by at least four pTAL_*Bam*H1_ clones and no solitary pTAL_*Bam*H1_ clone was found, it is highly possible that the 18 *tal* genes identified in this study compose all the *tal* genes of strain K74. To the best of our knowledge, this is the first report on genome-wide identification of *tal* genes from a strain whose genome sequences are not available. As described above, TAL effectors play very important roles in *Xanthomonas*-host interactions. They function as virulence or avirulence factors which promote the expression of host genes associated with disease symptoms or trigger genes that confer disease resistance, and their target specificity is determined by the RVDs. Identification of more *tal* genes from diverse *Xanthomonas* species, pathovars, or strains and analysis of their RVDs will facilitate greatly the investigation of disease resistance genes and susceptibility genes, and then enhance crop resistance by molecular plant breeding. In addition to *Xanthomonas*, TAL effectors have been also found in *Ralstonia solanacearum*^4^. The method may also apply to genome-wide identification of *Ralstonia tal* genes from diverse strains.

In addition to the variation in TALE numbers, some RVDs of the similar TALEs among strains K74, PXO99^A^, and MAFF311018 are diverse (Supplementary Table 7). Out of curiosity, we performed a target prediction for the counterparts of the Tal_*Xoo*K74_ in PXO99^A^ using TALgetter 1.0 with the same parameters. When comparing the results issued from the two sets of TALEs, we observed that the top targets of Tal_*Xoo*K74_ strikingly depart from those of the TALEs in PXO99^A^ (Supplementary Table 9), indicating that the polymorphism in the RVD sequences underlies diverging functions, which could be beneficial for maintaining virulence in different rice cultivars in different geographic regions.

*In silico* prediction revealed that the rice SWEET gene *Os11N3* is the top potential target of Tal_*Xoo*K74_-2 (Supplementary Table 8). As described above, some plant SWEET genes are susceptibility genes whose induction is essential for a pathogen’s successful infection. This suggests that Tal_*Xoo*K74_-2 may be a major virulence determinant of strain K74, although further experiments are needed to validate the prediction. The prediction also revealed that the BAK1-encoding gene *Os08g07760* and the Hen1 gene *Os07g06970* are the most likely targets for Tal_*Xoo*K74_-10 and Tal_*Xoo*K74_-12, respectively, and the WRKY gene *Os01g61080* is the second most likely target for Tal_*Xoo*K74_-3 (Supplementary Table 8). Are these rice genes and other predicted targets real targets of the TALEs? If so, how does plant response to their induction and what will happen to the pathogen? These will be the topics that merit our further studies.

## Methods

### Bacterial strains, plasmids, and growth conditions

*Xoo* and *E. coli* strains, as well as the plasmids used in this study are listed in Supplementary Table 1. *Xoo* was grown in PSA medium (10 g of peptone, 10 g of sucrose, 1 g of glutamic acid, 16 g of agar per liter of H_2_O) at 28 °C; *E. coli* was cultured in LB medium (10 g of tryptone, 5 g of yeast extract and 10 g of NaCl per liter of H_2_O) at 37 °C.

### RFLP analysis

The RFLP analysis of *tal* genes of strain K74 was performed as follow: 5 μg of *Bam*HI completely digested genomic DNA of strain K74 were separated by electrophoresis on an 1% agarose gel in 1 × TAE (Tris-acetate-EDTA) buffer at 60 V for 38 h in a cold chamber; TAE buffer was refreshed every 13 h. The neutral transfer gel treatment procedure and the capillary blotting procedure were carried out according to the manufacture manual, and DNA fragments in the gel were transferred to an Amersham Hybond-N+ membrane (GE Healthcare). A 500-bp fragment of *talC* gene (from 144^th^ bp to 644^th^ bp) labeled with Roche DIG High Prime DNA Labeling and Detection Starter Kit II was used as probe for the hybridization. The 500-bp fragment of *talC* was amplified by PCR using *talC* DNA as template and the primer set *tal*-probe-F/*tal*-probe-R (Supplementary Table 2).

### Construction of the pTAL_*Bam*HI_ library

20 μg of genomic DNA of strain K74 were completely digested with 50 u of the restriction enzyme *Bam*HI (Promega) and electrophoresised. The resulting *Bam*HI fragments with sizes from 1.5 kb to 7.5 kb were recovered from agarose gel using the QIAquick Gel Extraction Kit (Qiagen). The obtained *Bam*HI fragments were ligated into *Bam*HI-cleaved pUC19 vector as follow: 60 ng of purified DNA fragments, 100 ng of pUC19, 2 μl of ligation buffer, and 5 u of T4 ligase (Promega) were mixed; the final reaction volume was adjusted to 20 μl with water. The ligations were performed at 4 °C for 12 h, and the ligation products were desalted using MF^TM^ membrane filters (0.025 μm, VSWP, Millipore) before being electroporated into *E. coli* strain Trans5α. The transformants were selected on ampicillin-containing LB medium (100 μg/ml) supplemented with IPTG (120 μg/ml) and X-gal (80  μg/ml), the plating density was around 300 colonies per plate (Ø = 14.4 cm). The dot blot on colony was performed following the manual provided with the Amersham Hybond-N+ Membranes (GE Healthcare). The probe used for the hybridization was the same as used in the RFLP analysis. The potential positive clones were confirmed by Southern blot using the same protocol as used in the RFLP analysis, except that the duration of the electrophoresis was 16 h.

### Construction of pCC2-*tal* clones for sequencing

pTAL_*Bam*HI_ DNA was partially digested with the restriction enzyme *Msc*I as follow: 2 μg pTAL_*Bam*HI_ plasmid DNA, 5 u of *Msc*I (New England BioLabs), and 2 μl of digestion buffer were mixed; the final reaction volume was adjusted to 20 μl with water, and digestions were performed at 37 °C for a period of time and stopped by heating at 65 °C for 10 minutes and supplementing with 3 μl of 10% SDS-containing loading dye (Takara). The DNA fragments were separated by eletrophoresis in agarose gel and the about 1-kb DNA band was sliced out from the gel and purified using QIAquick Gel Extraction Kit. The purified DNA fragments were vacuum-concentrated, and 20 ng of them was used for the ligation with 15 ng of pCC2FOS vector linearized with the restriction enzyme *Eco*72 I (Epicentre). The ligation products were electroporated into the *E. coli* strain EPI300-T1^R^ (Epicenter) and the transformants were selected on chloramphenicol-containing LB medium supplemented with IPTG (120 μg/ml) and X-gal (80 μg/ml). The copy number induction of the pCC2FOS-*Msc*I clones were performed prior to plasmid extraction using CopyControl™ Induction Solution (Epicentre) according to the provided manual.

### DNA sequencing

DNA sequencing was performed at Sangon Biotech, and all sequencing primers used in this study were listed in Supplementary Table 2. The sequences were extracted from .ab1 files using Geneious 7.0.6, and reading errors were corrected manually regarding the chromatograms.

### Bioinformatics analysis

Multiple DNA sequence alignments were performed using the MUSCLE alignment module in Geneious 7. All parameters were set to default except that the output was grouped by similarity. The automatic *de novo* assembling of *tal-Bam*HI fragments was carried out using the *De Novo* Assemble Module of Geneious 7. For the assembling of all *tal-Bam*HI fragments, the minimum overlap value was set to 50 bp and 200 bp for the non-repeat and repeat regions, respectively; the minimum overlap identity was set to 100% in all cases. The online version of TALgetter (version 1.0)^15^ was used to predict the potential direct targets of the Tal_*Xoo*K74_ effectors in the promoterome of *Oryza sativa* cultivar Nipponbare (1 kb DNA sequences upstream of all start codons). The upstream and downstream offsets were set to 0, the fine-grained p-value mode was selected to compute the p-values, and 1e-06 was set as the maximum p-value to filter the hits generated by the program.

## Additional Information

**How to cite this article**: Yu, Y.-H. *et al*. Rapid and efficient genome-wide characterization of *Xanthomonas* TAL effector genes. *Sci. Rep*. **5**, 13162; doi: 10.1038/srep13162 (2015).

## Supplementary Material

Supplementary Information

Supplementary Data 1

Supplementary Data 2

## Figures and Tables

**Table 1:** List of the top predicted targets for each of the 18 Tal_*Xoo*K74_ effectors.

Tal_*Xoo*K74_	Target ID	Annotation	Target sequences	Score	p-value	E-value
1	LOC_Os06g18040.1	hypothetical protein	TCCTTTTCCCCTCCCCGC	−9.1854	2.1537E-08	1.4
2^a^	LOC_Os11g31190.1	nodulin MtN3 family protein	TATATAAACCCCCTCCAACCAGGTGCTAA	−16.1988	0	0
3^a^	LOC_Os08g30910.1	YDG/SRA domain containing protein	TCTCCTCGCCCAAAACCCCCCCAACTCC	−22.1250	3.1084E-09	0.2
	LOC_Os01g61080.1	WRKY24	TCTATCCGCCAAAAACCCTACCCGCTCC	−22.7700	1.2434E-08	0.8
4	LOC_Os12g38380.1	tetratricopeptide repeat containing protein	TCCGAATCCGCTCTCCCAATCCTCTTCT	−21.8771	3.1084E-09	0.2
	LOC_Os12g18110.1	PQ loop repeat domain containing protein	CCCGGTTCGTCCCTCCCAAGCCCCAACC	−23.1760	1.7096E-08	1.1
5	LOC_Os07g09384.1	expressed protein	AGCCTCGCACGCGCGCGAGGCCG	−16.0279	4.6387E-09	0.3
6^b^	LOC_Os07g05400.1	retrotransposon protein	TAGAAGGGACAATCGCTAACCTCC	−18.3096	4.9530E-08	3.2
7^a^	LOC_Os12g28330.1	retrotransposon protein	TATAAAACCCCAACCCCTCCCCT	−16.6017	2.1647E-08	1.4
8	LOC_Os01g40290.1	expressed protein	TATATACCTCGTTTCTCCAGG	−11.4833	0	0
	LOC_Os12g30590.1	disease resistance protein RGA2	TATATTCATCATTTTACCAGT	−12.1436	1.5431E-09	0.1
9	LOC_Os06g29530.1	retrotransposon protein	TAATAGACGCATCCACAAGAT	−13.4391	2.0060E-08	1.3
10	LOC_Os08g07760.1	brassinosteroid insensitive 1-associated receptor kinase 1 precursor	TATAAAGCGAGGCGACGAACT	−12.4998	3.0861E-09	0.2
11	LOC_Os01g47650.1	vacuolar protein sorting-associated protein 16	TATGTCTAGAGAAATAGAAGT	−13.3070	1.8517E-08	1.2
12	LOC_Os07g06970.1	HEN1, putative	TCCCTTCCCTAAACCCCACTT	−10.4036	0	0
13	LOC_Os06g29790.1	phosphate transporter 1	TATAAGTGACAGCCCTCCCCT	−11.2732	1.5431E-09	0.1
14	LOC_Os08g03550.1	expressed protein	TAGCTCGCCGGAGCACCGC	−12.6372	5.2400E-08	3.4
15	LOC_Os10g28990.1	MBTB42 - Bric-a-Brac, Tramtrack, Broad Complex BTB domain with Meprin and TRAF Homology MATH domain	TCCTTTTCCACGCGCACGC	−10.9153	2.4638E-08	1.6
16	LOC_Os06g32590.1	expressed protein	TACCTAGGCAGATCCAT	−8.5410	1.5368E-08	1
17	LOC_Os09g25200.1	protein binding protein	TAGCAAACATGCGAATC	−8.2442	1.2294E-08	0.8
18	LOC_Os08g05910.1	peptide transporter PTR2	TAGATTCTCTCCCT	−3.3726	7.6604E-09	0.5

^a^Counterpart presents in MAFF311018. but absent in PXO99^A^.

^b^Counterpart presents in PXO99^A^ but absent in MAFF311018.
