# Supplementary material for: Rapid and efficient genome-wide characterization of Xanthomonas TAL effector genes
Source: Sci Rep. 2015 Aug 14;5:13162. doi: 10.1038/srep13162 (PMC4536657; doi:10.1038/srep13162)
Supplement: Supplementary Information [file srep13162-s1.pdf]

**Rapid and efficient genome-wide characterization of *Xanthomonas* TAL effector genes**

Yan-Hua Yu*, Ye Lu*, Yong-Qiang He, Sheng Huang & Ji-Liang Tang**

State Key Laboratory for Conservation and Utilization of Subtropical Agro-bioresources, The Key Laboratory of Ministry of Education for Microbial and Plant Genetic Engineering, and College of Life Science and Technology, Guangxi University, 100 Daxue Road, Nanning, Guangxi 530004, China.

*These authors contributed equally to this work.

**Correspondence should be addressed to J.-L. Tang.

E-mail: jltang@gxu.edu.cn

**Supplementary Figure 1.** Schematic illustration of a typical *tal* gene **(A)** and a pTAL$_{Bam\mathrm{HI}}$ clone **(B)**. The translation start site is indicated in red and the *Bam*HI site at 5' end is underlined. The conserved motives are presented: TTS, type III translocation signal; RR, repeat region; NLS, nuclear localization signal; AD, transcription activation domain. The fragment used as probe for RFLP analysis and dot blot on colony is marked in gray; the primers used for sequencing are presented by arrows.

**Supplementary Figure 2.** Confirmation of the pTAL$_{Bam\mathrm{HI}}$ clones by Southern blot. The *Bam*HI-digested pTAL$_{Bam\mathrm{HI}}$ clones were probed with a 5' non-repeat portion of *talC*. The molecular weight standards and clone numbering are marked on the left and the top, respectively. The solid black lines separate the lanes that were run on different agarose gels.

**Supplementary Figure 3.** Scheme for DNA sequence assembling of the 18 unique *tal*-*Bam*HI fragments. Sequence reads are presented in broad solid lines and their proprietary clone numbers are listed on the left; the coverage of each nucleotide of the *tal*-*Bam*HI fragments is shown in blue.

**Supplementary Figure 4**. Scheme for DNA sequence assembling of the *talC*-*Bam*HI fragment. Sequence reads are presented in gray lines and the proprietary clone numbers are listed on the left. *talC*-*Bam*HI fragment is

presented in solid black line and the coverage of each nucleotide of *talC*-*Bam*HI fragment is shown in blue.

**Supplementary Table 1**. Bacterial strains and plasmids used in this study.

**Supplementary Table 2**. Primers used in this study.

**Supplementary Table 3**. Identity matrix obtained by multiple alignments of the DNA sequences of pTAL$_{BamHI}$ clones obtained from sequencing with primer P1F. The identity between clones is presented in percentage.

**Supplementary Table 4**. Identity matrix obtained by multiple alignments of the DNA sequences of pTAL$_{BamHI}$ clones obtained from sequencing with primer P1R. The identity between clones is presented in percentage.

**Supplementary Table 5**. *in silico* analysis of *Msc*I and *Bsm*BI restriction sites in the tandem repeat domains of *tal* genes.

**Supplementary Table 6**. The tandem amino acid repeats of the 18 Tal$_{XooK74}$ effectors and TalC effector.

**Supplementary Table 7**. Comparison of the RVD strings of the TALEs issued from *Xoo* strains K74, PXO99$^{A}$ and MAFF311018.

**Supplementary Table 8**. List of the top 10 predicted targets for each of the 18 Tal$_{\mathit{Xoo}\mathrm{K74}}$ effectors obtained by TALgetter 1.0.

**Supplementary Table 9.** Comparison of the top 10 predicted targets of the Tal$_{\mathit{Xoo}\mathrm{K74}}$ and their counterparts in PXO99$^{A}$.

**Supplementary Data 1**. The initial DNA sequence data of the 18 unique *tal-Bam*HI fragments of *Xoo* strain K74 and the *tal-Bam*HI fragment of *talC*, obtained from sequencing.

**Supplementary Data 2**. Assembled DNA sequences of the 18 unique *tal-Bam*HI fragments of *Xoo* strain K74 and the *tal-Bam*HI fragment of *talC*.

# Supplementary Figure 1

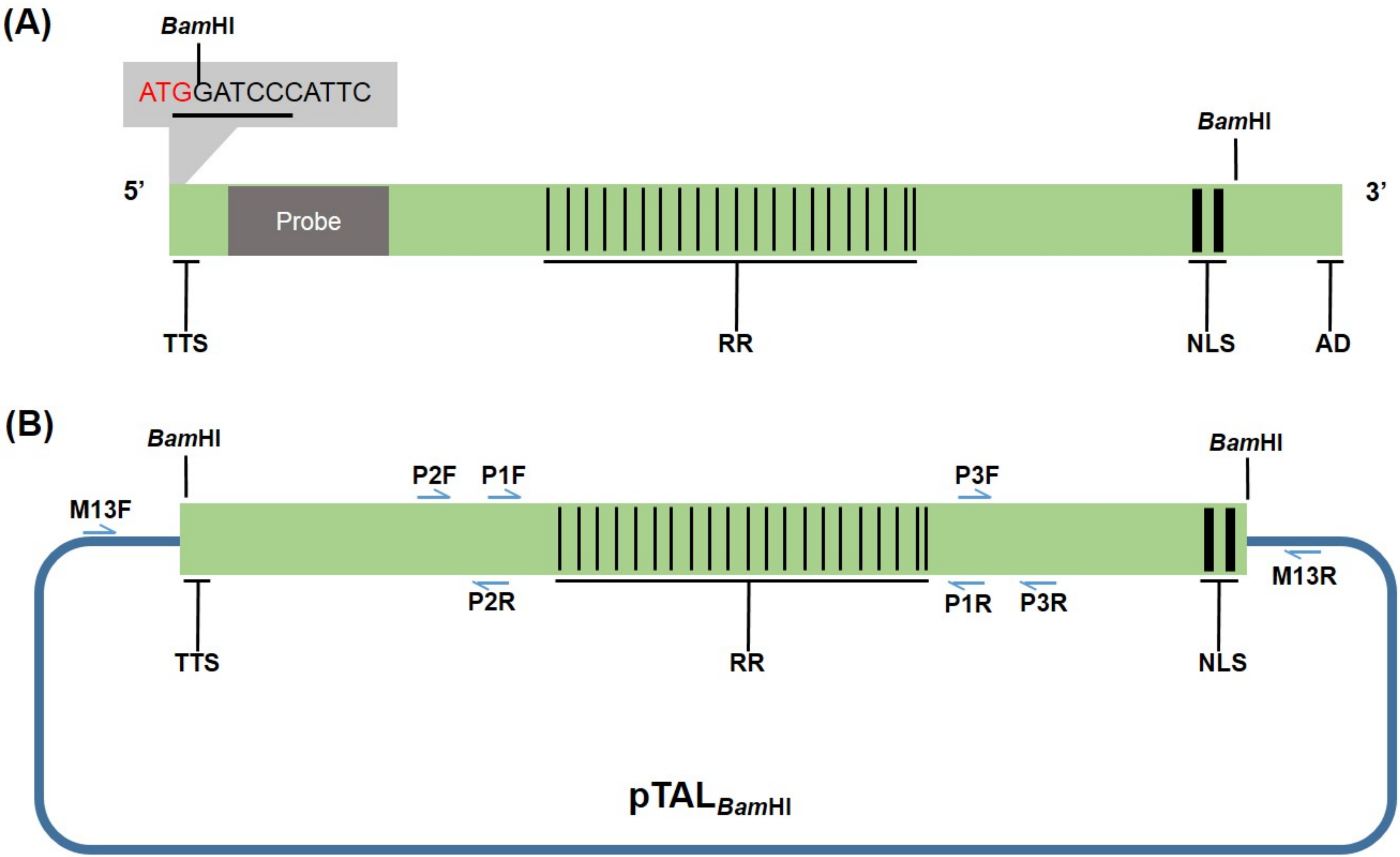

## Supplementary Figure 2

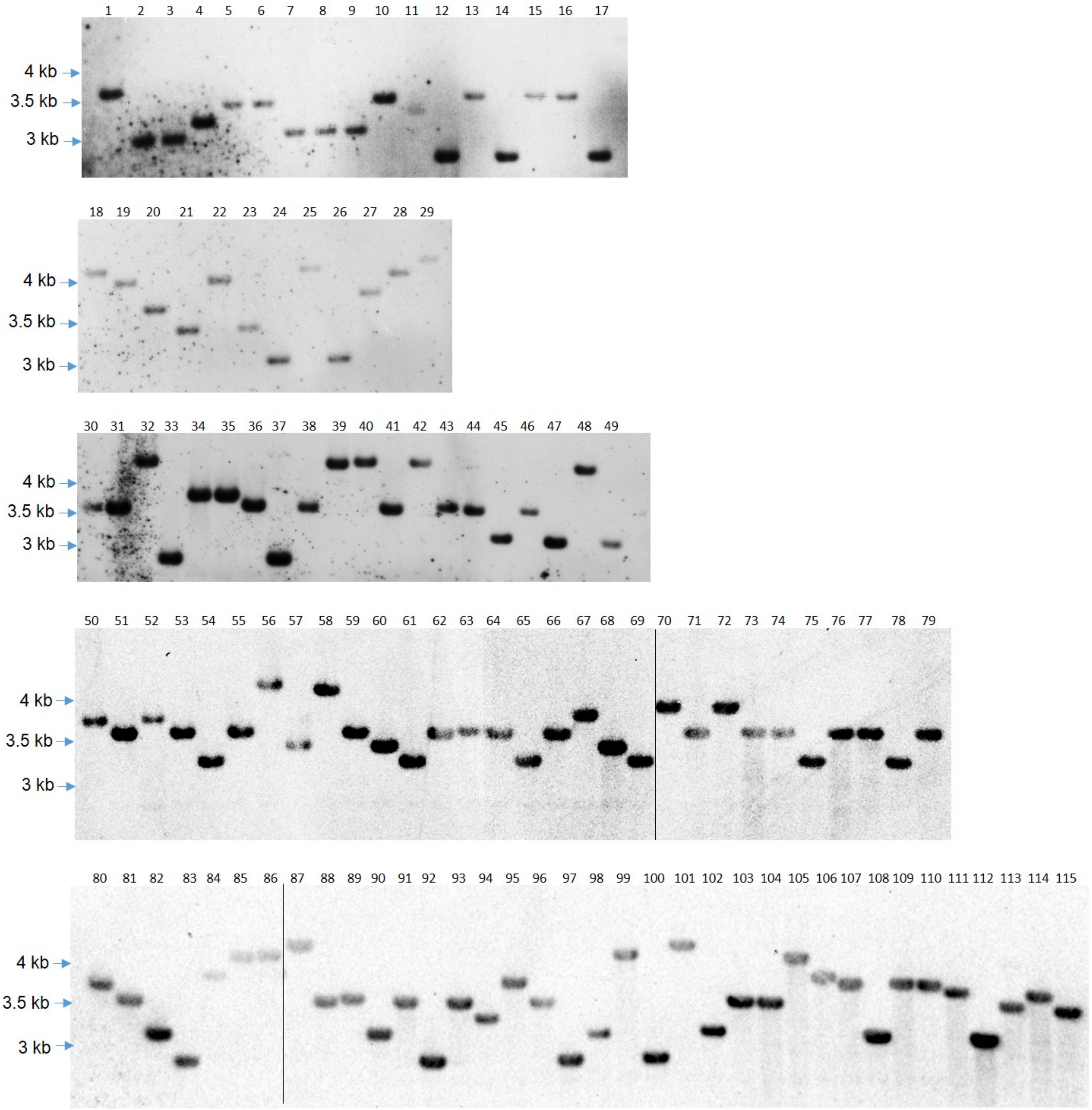

# Supplementary Figure 3

*tal-Bam*HI-1

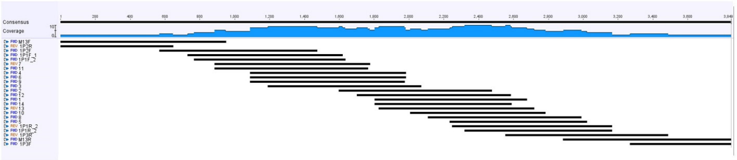

*tal-Bam*HI-11

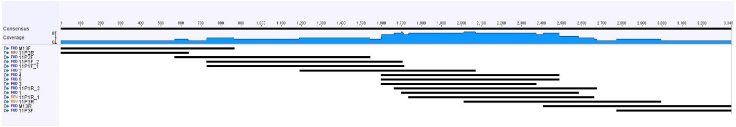

*tal-Bam*HI-14

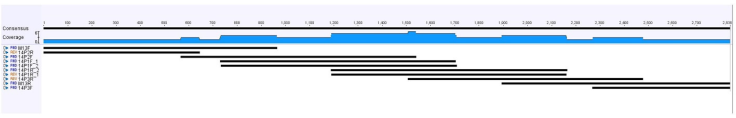

*tal-Bam*HI-16

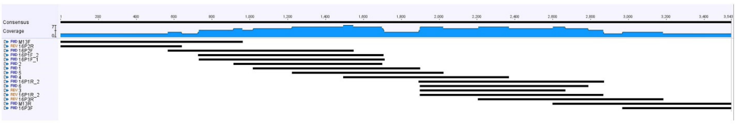

*tal-Bam*HI-21

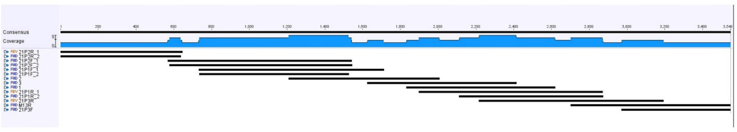

*tal-Bam*HI-22

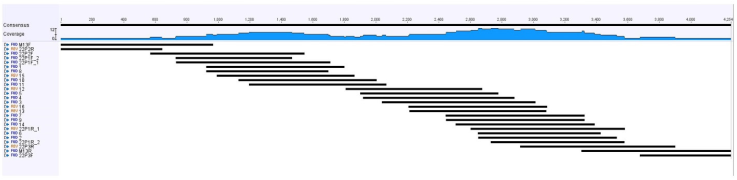

*tal-Bam*HI-24

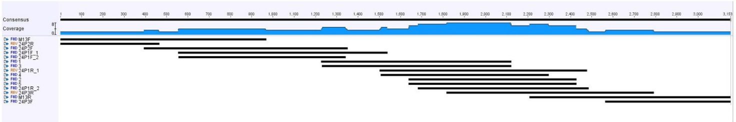

*tal-Bam*HI-26

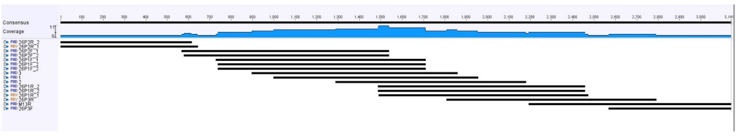

*tal-Bam*HI-27

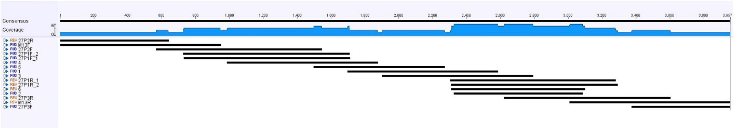

*tal-Bam*HI-31

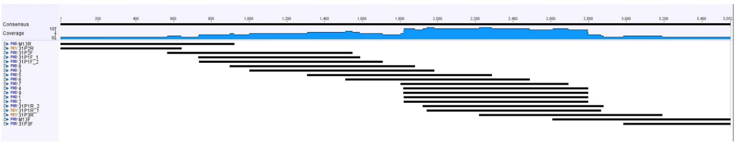

*tal-Bam*HI-34

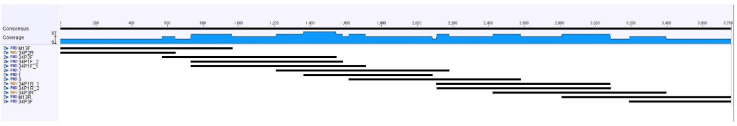

*tal-Bam*HI-36

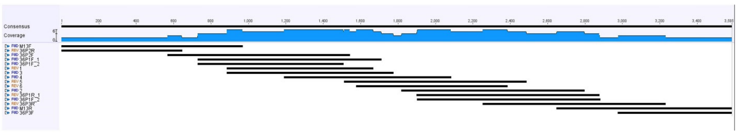

*tal-Bam*HI-39

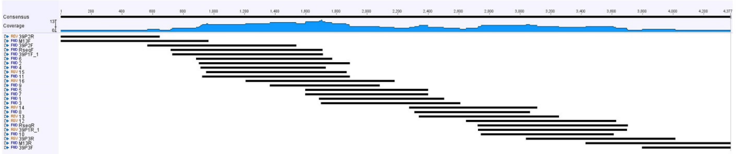

*tal-Bam*HI-40

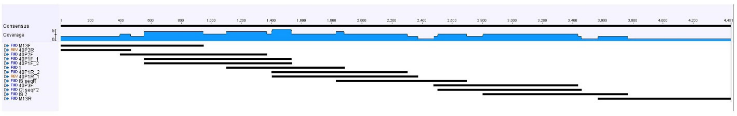

*tal-Bam*HI-41

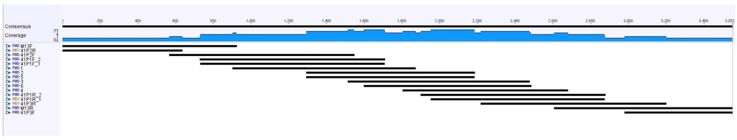

*tal-Bam*HI-47

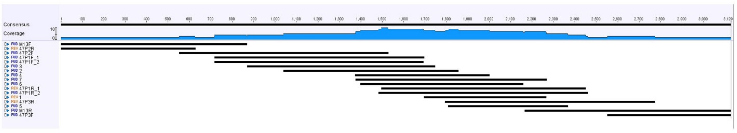

*tal-Bam*HI-6

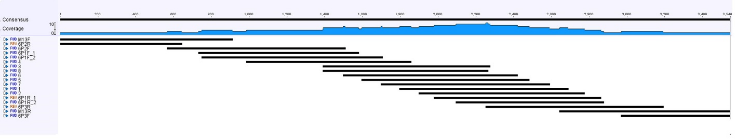

*tal-Bam*HI-86

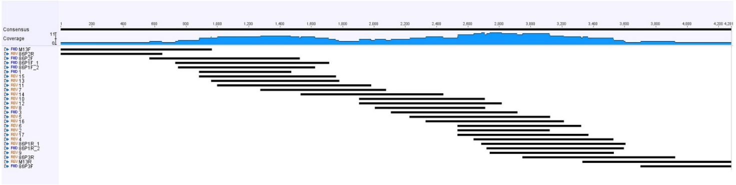

# Supplementary Figure 4

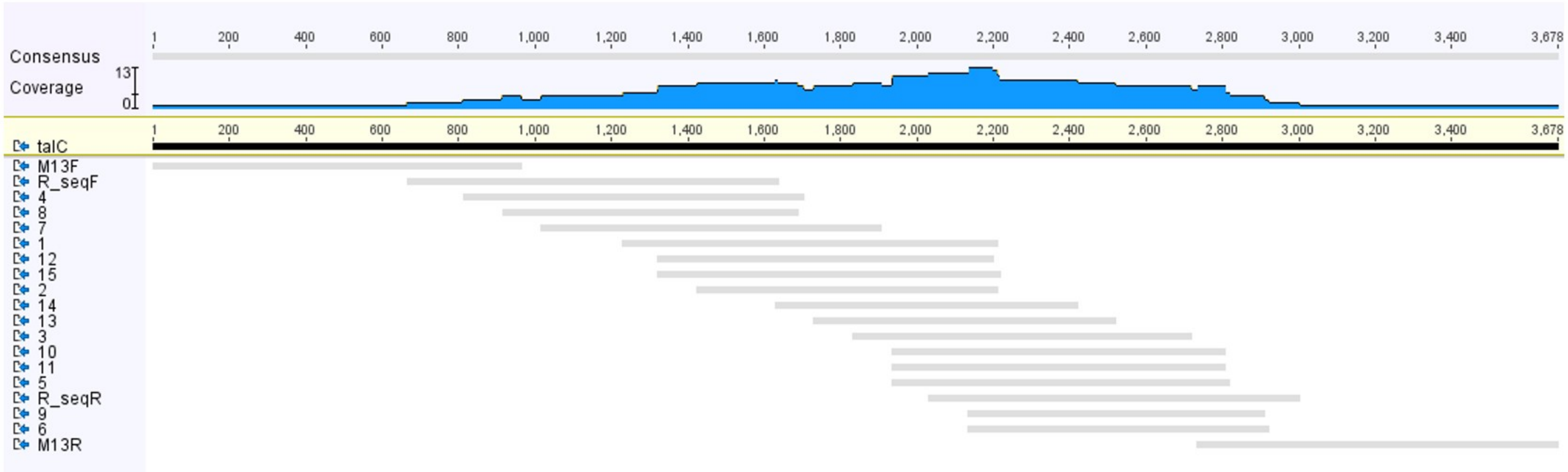

**Supplementary Table 1**. Bacterial strains and plasmids used in this study.

| Strains or plasmids | Relevant characteristics | Reference or source |
|---|---|---|
| Strains | | |
| *Xanthomonas oryzae* pv. *oryzae* | | |
| K74 | A spontaneous streptomycin-resistant mutant of the wild-type strain 1074, isolated from Fangchenggang city, China | Yang et al., 2013 |
| *Escherichia coli* | | |
| Trans5α | F-φ80 *lac* ZΔM15 Δ(*lac*ZYA-*arg*F) U169 *end*A1 *rec*A1 *hsd*R17(rk-,mk+) supE44λ- *thi -1 gyr*A96 *rel*A1 *pho*A | TransGene |
| EPI300 -T1[R] | F- mcrA D(*mrr*-*hsd*RMS-*mcr*BC) f80d*lac*ZDM15 D*lac*X74 *rec*A1 *end*A1 *ara*D139 D(*ara*, *leu*)7697 *gal*U *gal*K l- *rps*L *nup*G *trf*A *ton*A *dhfr* | Epicentre |
| Plasmids | | |
| pUC19 | Cloning vector, ampicillin resistant | TransGene |
| pCC2FOS | Fosmid vector, remaining single copy in EPI300 without induction, ampicillin and chloramphenicol resistant | Epicentre |
| pTAL$_{\textit{Bam}\mathrm{HI}}$ clones | pUC19 clones containing *tal*-*Bam*HI fragments | This study |
| pCC2FOS-*Msc*I clones | pCC2FOS clones containing *Msc*I fragments of *tal* genes | This study |

**Supplementary Table 2. Primers used in this study.**

| Primer name | Sequence (5'-3') | Application |
|---|---|---|
| P1F | CGGCAAACAGTGGTCCGGC | 5' end of repeat region sequencing |
| P1R | AGGCGACGAGGTGGTCGTTGG | 3' end of repeat region sequencing |
| P2F | CGACAGTGGCGCAGCACCAC | 5' non-repeat region sequencing and 5' end of repeat region sequencing |
| P2R | GCCGGACCACTGTTTGCCG | 5' non-repeat region sequencing |
| P3F | CCAACGACCACCTCGTCGCCT | 3' non-repeat region sequencing |
| P3R | ATACGGTCCCAACGCTGCGA | 3' non-repeat region sequencing and 3' end of repeat region sequencing |
| pCC2-seqF | GTACAACGACACCTAGAC | pCC2FOS-*Msc*I clones sequencing |
| *tal*-probe-F | ATCGGGATCCTGTCCCGGACCCGGCTGCCA | Amplification of the probe for RFLP analysis and dot blot analysis |
| *tal*-probe-R | ATCGAAGCTTCTCCAGGGCGCGTGCGCCGGA | Amplification of the probe for RFLP analysis and dot blot analysis |
| M13F | AGGGTTTTCCCAGTCACG | non repeat region sequencing |
| M13R | GAGCGGATAACAATTTCACAC | non repeat region sequencing |

**Supplementary Table 3**. Identity matrix obtained by multiple alignments of the DNA sequences of pTAL$_{Rem III}$ clones obtained from sequencing with primer P1F. The identity between clones is presented in percentage.

| pTAL$_{Rem III}$ clones | 85 | 28 | 19 | 22 | 87 | 101 | 40 | 18 | 32 | 20 | 42 | 69 | 75 | 9 | 8 | 7 | 3 | 2 | 24 |
|---|---|---|---|---|---|---|---|---|---|---|---|---|---|---|---|---|---|---|---|
| 85 | | 100 | 100 | 100 | 92,49 | 92,95 | 93,27 | 93,22 | 93,27 | 93,24 | 93,05 | 93,37 | 93,37 | 93,08 | 92,73 | 93,1 | 93,1 | 93,14 | 92,5 |
| 28 | 100 | | 100 | 100 | 92,44 | 92,44 | 92,99 | 92,97 | 92,99 | 93,01 | 92,99 | 93,1 | 93,1 | 93,12 | 92,74 | 93,11 | 93,13 | 93,13 | 92,51 |
| 19 | 100 | 100 | | 100 | 92,42 | 92,95 | 92,91 | 93,22 | 93,29 | 93,27 | 93,07 | 93,01 | 93,01 | 93,11 | 92,74 | 93,11 | 93,13 | 93,17 | 92,51 |
| 22 | 100 | 100 | 100 | | 92,72 | 92,72 | 93,23 | 93,21 | 93,23 | 93,23 | 93,07 | 93,33 | 93,33 | 93,1 | 92,74 | 93,11 | 93,12 | 93,16 | 92,51 |
| 87 | 92,49 | 92,44 | 92,42 | 92,72 | | 100 | 100 | 100 | 100 | 100 | 100 | 99,88 | 99,88 | 99,86 | 99,85 | 99,86 | 99,86 | 99,86 | 99,84 |
| 101 | 92,95 | 92,44 | 92,95 | 92,72 | 100 | | 100 | 100 | 100 | 100 | 100 | 99,88 | 99,88 | 99,86 | 99,85 | 99,86 | 99,86 | 99,86 | 99,84 |
| 40 | 93,27 | 92,99 | 92,91 | 93,23 | 100 | 100 | | 100 | 100 | 100 | 100 | 99,9 | 99,9 | 99,87 | 99,86 | 99,87 | 99,87 | 99,87 | 99,85 |
| 18 | 93,22 | 92,97 | 93,22 | 93,21 | 100 | 100 | 100 | | 100 | 100 | 100 | 99,88 | 99,88 | 99,87 | 99,86 | 99,87 | 99,87 | 99,87 | 99,85 |
| 32 | 93,27 | 92,99 | 93,29 | 93,23 | 100 | 100 | 100 | 100 | | 100 | 100 | 99,88 | 99,88 | 99,87 | 99,86 | 99,87 | 99,87 | 99,87 | 99,85 |
| 20 | 93,24 | 93,01 | 93,27 | 93,23 | 100 | 100 | 100 | 100 | 100 | | 100 | 99,88 | 99,88 | 99,87 | 99,86 | 99,87 | 99,87 | 99,87 | 99,85 |
| 42 | 93,05 | 92,99 | 93,07 | 93,07 | 100 | 100 | 100 | 100 | 100 | 100 | | 99,87 | 99,87 | 99,87 | 99,86 | 99,87 | 99,87 | 99,87 | 99,85 |
| 69 | 93,37 | 93,1 | 93,01 | 93,33 | 99,88 | 99,88 | 99,9 | 99,88 | 99,88 | 99,88 | 99,87 | | 100 | 100 | 100 | 100 | 100 | 100 | 100 |

| pTAL$_{Rem III}$ clones | 78 | 65 | 102 | 45 | 113 | 63 | 89 | 36 | 43 | 86 | 58 | 105 | 99 | 57 | 60 | 68 | 94 | 4 | 11 | 37 | 17 | 12 | 33 | 100 | 83 | 92 | 97 | 14 | 67 | 20 | 1 | 95 | 107 | 109 | 110 | 90 | 54 | 26 | 49 | 61 | 108 | 82 | 98 | 112 | 47 | 31 | 30 | 15 | 96 | 73 | 77 | 44 | 48 | 88 | 25 | 39 | 35 | 34 | 114 | 50 | 52 | 80 | 111 | 59 | 66 | 62 | 46 | 21 | 23 | 5 | 38 | 79 | 16 | 10 | 41 | 13 | 104 | 103 | 51 | 53 | 64 | 74 | 71 | 76 | 81 | 88 | 91 | 6 | 55 | 93 | 115 | 70 | 72 | 84 | 106 | 27 |
|---|---|---|---|---|---|---|---|---|---|---|---|---|---|---|---|---|---|---|---|---|---|---|---|---|---|---|---|---|---|---|---|---|---|---|---|---|---|---|---|---|---|---|---|---|---|---|---|---|---|---|---|---|---|---|---|---|---|---|---|---|---|---|---|---|---|---|---|---|---|---|---|---|---|---|---|---|---|---|---|---|---|---|---|---|---|---|---|---|---|---|---|---|---|---|---|---|
| [illegible] | [illegible] | [illegible] | [illegible] | [illegible] | [illegible] | [illegible] | [illegible] | [illegible] | [illegible] | [illegible] | [illegible] | [illegible] | [illegible] | [illegible] | [illegible] | [illegible] | [illegible] | [illegible] | [illegible] | [illegible] | [illegible] | [illegible] | [illegible] | [illegible] | [illegible] | [illegible] | [illegible] | [illegible] | [illegible] | [illegible] | [illegible] | [illegible] | [illegible] | [illegible] | [illegible] | [illegible] | [illegible] | [illegible] | [illegible] | [illegible] | [illegible] | [illegible] | [illegible] | [illegible] | [illegible] | [illegible] | [illegible] | [illegible] | [illegible] | [illegible] | [illegible] | [illegible] | [illegible] | [illegible] | [illegible] | [illegible] | [illegible] | [illegible] | [illegible] | [illegible] | [illegible] | [illegible] | [illegible] | [illegible] | [illegible] | [illegible] | [illegible] | [illegible] | [illegible] | [illegible] | [illegible] | [illegible] | [illegible] | [illegible] | [illegible] | [illegible] | [illegible] | [illegible] | [illegible] | [illegible] | [illegible] | [illegible] | [illegible] | [illegible] | [illegible] | [illegible] | [illegible] | [illegible] | [illegible] | [illegible] | [illegible] | [illegible] | [illegible] | [illegible] | [illegible] | [illegible] |

**Supplementary Table 4** . Identity matrix obtained by multiple alignments of the DNA sequences of pTAL$_{Bam III}$ clones obtained from sequencing with primer P1R.The identity between clones is presented in percentage.

| pTAL$_{Bam III}$ clones | 106 | 84 | 70 | 72 | 27 | 26 | 90 | 54 | 61 | 49 | 18 | 32 | 42 | 101 | 87 | 29 | 40 | 37 | 33 | 17 | 83 | 97 | 92 |
|---|---|---|---|---|---|---|---|---|---|---|---|---|---|---|---|---|---|---|---|---|---|---|---|
| 106 | | 100 | 100 | 100 | 100 | 93,59 | 93,56 | 93,65 | 93,68 | 93,81 | 93,4 | 93,31 | 93,48 | 93,24 | 93,26 | 93,28 | 94,1 | 93,97 | 93,4 | 93,5 | 93,49 | 93,49 | 93,52 |
| 84 | 100 | | 100 | 100 | 100 | 94,07 | 94,07 | 94,07 | 93,68 | 94,07 | 93,39 | 93,3 | 93,47 | 93,24 | 93,24 | 93,14 | 94,1 | 93,96 | 93,49 | 93,49 | 93,47 | 93,47 | 93,49 |
| 70 | 100 | 100 | | 100 | 100 | 93,65 | 93,69 | 93,7 | 93,68 | 93,83 | 93,4 | 93,4 | 93,56 | 93,24 | 93,26 | 93,3 | 94,1 | 94,07 | 93,48 | 93,52 | 93,49 | 93,49 | 93,52 |
| 72 | 100 | 100 | 100 | | 100 | 94,07 | 94,07 | 94,07 | 93,67 | 94,07 | 93,36 | 93,27 | 93,44 | 93,23 | 93,26 | 93,02 | 94,1 | 93,92 | 93,49 | 93,46 | 93,48 | 93,48 | 93,48 |
| 27 | 100 | 100 | 100 | 100 | | 93,57 | 93,61 | 93,71 | 93,68 | 93,83 | 93,4 | 93,4 | 93,56 | 93,24 | 93,26 | 93,32 | 94,1 | 94,07 | 93,17 | 93,52 | 93,49 | 93,49 | 93,52 |
| 26 | 93,59 | 94,07 | 93,65 | 94,07 | 93,57 | | 100 | 100 | 100 | 100 | 94,72 | 94,44 | 94,58 | 94,31 | 94,24 | 93,6 | 95,78 | 95,19 | 94,31 | 94,84 | 94,42 | 94,42 | 94,44 |
| 90 | 93,56 | 94,07 | 93,69 | 94,07 | 93,61 | 100 | | 100 | 100 | 100 | 94,72 | 94,48 | 94,62 | 94,31 | 94,24 | 93,61 | 95,78 | 95,23 | 94,35 | 94,84 | 94,42 | 94,42 | 94,44 |

Supplementary Table 5. *In silico* analysis of *Msc*I and *Bsm*BI restriction sites in the tandem repeat domains of *tal* genes.

| Accesion number | *tal* alias | repeat number | Strains | *Msc* I sites | | *Bsm* BI sites | | Species |
|---|---|---|---|---|---|---|---|---|
| | | | | num. of sites in repeats | Max. product length (bp) | num. of sites in repeats | Max. product length (bp) | |
| ACD11364.1 | *avr/pthC8b* | 17,5 | C8 | 18 | 111 | 17 | 115 | *X.oryzae* pv. *oryzae* |
| ABY60855.1 | *avr/pthC8a* | 20,5 | C8 | 21 | 102 | 19 | 208 | |
| AAT46122.1 | *avrXa7-1M* | 25,5 | PXO0314 | 26 | 117 | 25 | 124 | |
| AAF98343.1 | *avrXa7* | 25,5 | PXO2684 | 26 | 117 | 25 | 124 | |
| AAT46123.1 | *avrXa7-2M* | 22,5 | PXO348 | 22 | 204 | 21 | 208 | |
| AAT46124.1 | *avrXa7-3M* | 26,5 | PXO356 | 27 | 120 | 24 | 208 | |
| AAT46128.1 | *avrXa7-3M* | 26,5 | PXO357 | 27 | 120 | 24 | 208 | |
| AAT46125.1 | *avrXa7-4M* | 25,5 | PXO557 | 26 | 117 | 25 | 124 | |
| AAS46027.1 | *pthXo3* | 28,5 | PXO61 | 29 | 117 | 26 | 208 | |
| AAS46026.1 | *pthXo2* | 21,5 | JX01 | 22 | 102 | 20 | 106 | |
| ACM44927.1 | *avrxa5* | 19,5 | JXOIII | 20 | 102 | 19 | 102 | |
| AAS75145.1 | *avrXa7-2* | 25,5 | KXO85 | 26 | 117 | 25 | 124 | |
| AAA92974.1 | *avrXa10* | 15,5 | PXO86 | 16 | 102 | 12 | 208 | |
| AEK86668.1 | *talC* | 21,5 | BAI3 | 22 | 102 | 21 | 106 | |
| AAW75382.1 | - | 19,5 | KACC10331 | 20 | 102 | 19 | 106 | |
| AAW75385.1 | *pthA1* | 23,5 | KACC10331 | 23 | 204 | 21 | 208 | |
| AAW75518.1 | - | 16,5 | KACC10331 | 17 | 102 | 16 | 106 | |
| AAW75529.1 | *pthA4* | 26,5 | KACC10331 | 25 | 306 | 26 | 106 | |
| AAW75530.1 | - | 19,5 | KACC10331 | 19 | 204 | 18 | 106 | |
| AAW75533.1 | - | 22,5 | KACC10331 | 22 | 204 | 22 | 106 | |
| AAW76267.1 | *pthA2* | 19,5 | KACC10331 | 18 | 204 | 18 | 208 | |
| AAW76269.1 | *pthA3* | 18,5 | KACC10331 | 18 | 216 | 18 | 124 | |
| YP_200767.1 | - | 19,5 | KACC10331 | 20 | 102 | 19 | 106 | |
| YP_201653.1 | - | 33,5 | KACC10331 | 34 | 120 | 32 | 208 | |
| YP_200766.1 | - | 17,5 | KACC10331 | 18 | 102 | 16 | 205 | |
| YP_450161.1 | - | 12,5 | MAFF 311018 | 13 | 102 | 10 | 208 | |
| YP_450163.1 | *avrXa27* | 16,5 | MAFF 311018 | 16 | 204 | 16 | 106 | |
| YP_450165.1 | - | 26,5 | MAFF 311018 | 28 | 102 | 25 | 208 | |
| YP_450167.1 | - | 19,5 | MAFF 311018 | 20 | 102 | 19 | 106 | |
| YP_451025.1 | - | 17,5 | MAFF 311018 | 18 | 102 | 17 | 106 | |
| YP_451027.1 | - | 19,5 | MAFF 311018 | 20 | 102 | 19 | 106 | |
| YP_451030.1 | - | 23,5 | MAFF 311018 | 23 | 204 | 21 | 208 | |
| YP_451156.1 | - | 20,5 | MAFF 311018 | 21 | 102 | 20 | 106 | |
| YP_451158.1 | - | 15,5 | MAFF 311018 | 15 | 204 | 15 | 106 | |
| YP_451187.1 | - | 19,5 | MAFF 311018 | 18 | 306 | 19 | 106 | |
| YP_451189.1 | - | 30,5 | MAFF 311018 | 30 | 204 | 29 | 208 | |
| YP_451696.1 | - | 17,5 | MAFF 311018 | 18 | 102 | 17 | 106 | |
| YP_451894.1 | - | 19,5 | MAFF 311018 | 18 | 204 | 19 | 106 | |
| YP_451895.1 | - | 26,5 | MAFF 311018 | 27 | 120 | 24 | 208 | |
| YP_451897.1 | *avrXa7* | 25,5 | MAFF 311018 | 26 | 117 | 25 | 124 | |
| YP_453043.1 | - | 21,5 | MAFF 311018 | 22 | 102 | 20 | 106 | |
| BAE69422.1 | - | 17,5 | MAFF 311018 | 18 | 102 | 17 | 106 | |
| YP_451893.1 | - | 31,5 | MAFF 311018 | 32 | 102 | 31 | 106 | |
| ACD58246.1 | *talC2a* | 13,5 | PXO99$^{A}$ | 14 | 204 | 13 | 208 | |
| ACD58243.2 | *pthXo1* | 23,5 | PXO99$^{A}$ | 24 | 102 | 22 | 208 | |
| ACD58650.1 | *talC4* | 15,5 | PXO99$^{A}$ | 15 | 204 | 14 | 106 | |
| ACD58455.2 | *talC3b* | 17,5 | PXO99$^{A}$ | 18 | 102 | 17 | 106 | |
| ACD58449.2 | *talC3a* | 17,5 | PXO99$^{A}$ | 18 | 102 | 17 | 106 | |
| ACD58948.2 | *talC6a* | 19,5 | PXO99$^{A}$ | 18 | 306 | 19 | 106 | |
| ACD58925.2 | *talC5a* | 15,5 | PXO99$^{A}$ | 16 | 102 | 13 | 310 | |
| ACD58920.2 | *pthXo6* | 22,5 | PXO99$^{A}$ | 22 | 204 | 22 | 106 | |
| ACD59227.2 | *talC7b* | 19,5 | PXO99$^{A}$ | 19 | 204 | 18 | 208 | |
| ACD60557.2 | *talC9a* | 19,5 | PXO99$^{A}$ | 20 | 102 | 19 | 106 | |
| ACD60569.2 | *talC9d* | 12,5 | PXO99$^{A}$ | 13 | 102 | 9 | 208 | |
| ACD60573.2 | *talC9e* | 23,5 | PXO99$^{A}$ | 23 | 204 | 21 | 208 | |
| ACD57198.2 | *pthXo7* | 21,5 | PXO99$^{A}$ | 22 | 204 | 18 | 208 | |
| ACD58952.1 | *talC6b* | 20,5 | PXO99$^{A}$ | 21 | 102 | 20 | 106 | |
| ACD59223.2 | *talC7a* | 17,5 | PXO99$^{A}$ | 18 | 102 | 17 | 106 | |
| ACD60561.1 | *talC9b* | 27,5 | PXO99$^{A}$ | 28 | 102 | 25 | 208 | |
| ACD60565.2 | *avrXa27* | 16,5 | PXO99$^{A}$ | 16 | 204 | 16 | 106 | |
| ACD59416.2 | *talC8a* | 17,5 | PXO99$^{A}$ | 18 | 102 | 17 | 106 | |
| ACD59420.2 | *talC8b* | 19,5 | PXO99$^{A}$ | 19 | 204 | 18 | 208 | |
| AEQ94695.1 | *tal1c* | 15,5 | BLS256 | 16 | 102 | 14 | 208 | *X.oryzae* pv. *oryzicola* |
| AEQ94698.1 | *tal1a* | 12,5 | BLS256 | 11 | 306 | 12 | 106 | |
| AEQ95741.1 | *tal2g* | 16,5 | BLS256 | 16 | 201 | 16 | 106 | |
| AEQ95744.1 | *tal2d* | 15,5 | BLS256 | 14 | 204 | 15 | 106 | |
| AEQ95875.1 | *tal3b* | 17,5 | BLS256 | 17 | 204 | 17 | 106 | |
| AEQ95881.1 | *tal3c* | 15,5 | BLS256 | 16 | 102 | 15 | 106 | |
| AEQ96157.1 | *tal4c* | 22,5 | BLS256 | 21 | 204 | 20 | 208 | |
| AEQ96159.1 | *tal4a* | 25,5 | BLS256 | 26 | 102 | 24 | 208 | |
| AEQ96964.1 | *tal6* | 19,5 | BLS256 | 19 | 204 | 19 | 106 | |
| AEQ96996.1 | *tal7* | 16,5 | BLS256 | 15 | 204 | 17 | 106 | |
| AEQ98113.1 | *tal8* | 19,5 | BLS256 | 18 | 204 | 18 | 208 | |
| AEQ98324.1 | *tal9a* | 20,5 | BLS256 | 20 | 204 | 20 | 106 | |
| AEQ98589.1 | *tal12* | 19,5 | BLS256 | 20 | 102 | 18 | 106 | |
| AEQ98462.1 | *tal11b* | 17,5 | BLS256 | 18 | 102 | 17 | 106 | |
| AEQ95751.1 | *tal2a* | 20,5 | BLS256 | 20 | 204 | 20 | 106 | |
| AEQ96610.1 | *tal5b* | 17,5 | BLS256 | 17 | 204 | 17 | 106 | |
| ACZ62653.1 | *pthA* | 17,5 | XW19 | 18 | 102 | 17 | 106 | *X.axonopodis* pv. *citri* |
| AAY43358.1 | *hax2* | 21,5 | 5 | 22 | 102 | 21 | 102 | *X.campestris* pv. *armoraciae* |
| AAY43359.1 | *hax3* | 11,5 | 5 | 12 | 102 | 11 | 102 | |
| AAY43360.1 | *hax4* | 14,5 | 5 | 15 | 102 | 14 | 102 | |
| AAB00675.1 | *avrb6* | 13,5 | XcmH | 13 | 102 | 11 | 208 | *X. citri* pv. *malvacearum* |
| YP_001965982.1 | - | 20,5 | AG1 | 21 | 102 | 20 | 106 | *X. axonopodis* pv. *glycines* |
| NG_034463.1 | *avrBs3* | 17,5 | 71-21 | 18 | 102 | 17 | 102 | *X. campestris* pv. *vesicatoria* |

Supplementary Table 6. The tandem amino acid repeats of the 18 Tal $_{XooK74}$ effectors and TalC effector[a].

| Tal$_{XooK74}$- | Repeat num. | Repeat sequence |
|---|---|---|
| 1 | 1 | LTPDQVVAIASNSGGKQALETVQRLLPVLCQDHG |
| | 2 | LTPDQVVAIASHDGGKQALETVQRLLPVLCQDHG |
| | 3 | LTPDQVVAIASNGGGKQALETVQRLLPVLCQDHG |
| | 4 | LTPDQVVAIASNGGGKQALETVLCQAHG |
| | 5 | LTPAQVVAIASNGGGKQALETVQRLLPVLCQAHG |
| | 6 | LTLDQVVAIASNGGGKQALETVQRLLPVLCQAHG |
| | 7 | LTPDQVVAIASHDGGKQALETVQRLLPVLCQAHG |
| | 8 | LTPAQVVAIASHDGGKQALETVQRLLPVLCQAHG |
| | 9 | LTLDQVVAIASHDGGKQALETVQRLLPVLCQAHG |
| | 10 | LTLDQVVAIASHDGGKQALETVQRLLPVLCQDHG |
| | 11 | LTPAQVVAIASNNGGKQALETVQRLLPVLCQDHG |
| | 12 | LTPDQVVAIASHDGGKQALETVQRLLPVLCQAHG |
| | 13 | LTPDQVVAIASHDGGKQALETVQRLLPVLCQDHG |
| | 14 | LTPAQVVAIASHDGGKQALETVQRLLQVLCQDHG |
| | 15 | LTPDQVVAIASHDGGKQALETVQRLLPVLCQDHG |
| | 16 | LTPDQVVAIANNNGGKQALETVQRLLPVLCQAHG |
| | 17 | LTPDQVVAIASH*GGKQALESIV |
| 2 | 1 | LTPDQVVAIASNIGGKQALETVQRLLPVLCQANG |
| | 2 | LTPDQVVAIASHGGGKQALETVQRLLPVLCQDHG |
| | 3 | LTPDQVVAIASNIGGKQALETVQRLLPVLCQDHG |
| | 4 | LTPDQVVAIASHGGGKQALETVQRLLPVLCQDHG |
| | 5 | LTPDQVVAIASNIGGKQALETVQRLLPVLCQAHG |
| | 6 | LTPDQVVAIASNIGGKQALETVQRLLPVLCQDHG |
| | 7 | LTPAQVVAIASNIGGKQALETVQRLLPVLCQDHG |
| | 8 | LTPDQVVAIASHDGGKQALETVQRLLPVLCQDHG |
| | 9 | LTPDQVVAIANNNGGKQALETVQRLLPVLCQDHG |
| | 10 | LTPDQVVAIASHDGGKQALETVQRLLPVLCQDHG |
| | 11 | LTPDQVVAIASHDGGKQALETVQRLLPVLCQEHG |
| | 12 | LTPAQVVAIASHDGGKQALETVQRLLPVLCQDHG |
| | 13 | LTPDQVVAIASNGGGKQALATVQRLLPVLCQAHG |
| | 14 | LTPDQVVAIASHDGGKQALETVQRLLPVLCQANG |
| | 15 | LTPDQVVAIASNGGKQALETVQRLLPVQRLLPVLCQDHG |
| | 16 | LTQDQVVAIASNIGGKQALETVQRLLPVLCQANG |
| | 17 | LTQDQVVAIASHDGGKQALETVQRLLPVLCQDHG |
| | 18 | LTPDQVVAIASHDGGKQALETVQRLLPVLCQDHG |
| | 19 | LTPAQVVAIANNNGGKQALETVQRLLPVLCQDHG |
| | 20 | LTPDQVVAIASNIGGKQALATVQRLLPVLCQDHG |

| | | |
|---|---|---|
| | 21 | LTPDQVVAIANNNGGKQALETVQRLLPVLCQDHG |
| | 22 | LTPAQVVAIASNNGGKQALETVQRLLPVLCQDHG |
| | 23 | LTLDQVVAIASNGGSKQALETVQRLLPVLCQDHG |
| | 24 | LTPDQVVAIANNNGGKQALETVQRLLPVLCQDHG |
| | 25 | LTLDQVVAIASHDGGKQALETVQRLLPVLCQDHG |
| | 26 | LTLDKVVAIASN*GGKQALETVQRLLPVLCQDHG |
| | 27 | LTPNQVVAIASNSGGKQALETVQRLLPVLCQDHG |
| | 28 | LTPNQVVAIASN*GGKQALE |
| 3 | 1 | LTPDQVVAIASNIGGKQALETVQRLLPVLCQDHG |
| | 2 | LTPDQVVAIASHGGGKQALETVQRLLPVLCQDHG |
| | 3 | LTPDQVVAIASNIGGKQALETVQRLLPVLCQDHG |
| | 4 | LTPDQVVAIASNIGGKQALETVQRLLPVLCQDHG |
| | 5 | LTPDQVVAIASHGGGKQALETVQRLLPVLCQAHG |
| | 6 | LTPDQVVAIARHDGGKQALETVQRLLPVLCQAHG |
| | 7 | LTPDQVVAIANNNGGKQALETVQRLLPVLCQAHG |
| | 8 | LTPDQVVAIASHDGGKQALETVQRLLPVLCQAHG |
| | 9 | LTPDQVVAIASHDGGKQALETVQRLLPVLCQDHG |
| | 10 | LTPDQVVAIASHDGGKQALETVQRLLPVLCQDHG |
| | 11 | LTPDQVVAIASNIGGKQALETVQRLLPVLCQDHG |
| | 12 | LTPDQVVAIASNIGGKQALETVQRLLPVLCQDHG |
| | 13 | LTPDQVVAIANNNGGKQALETVQRLLPVQRLVPVLCQDHG |
| | 14 | LTQDQVVAIASNIGGKQALETVQRLLPVLYQDHG |
| | 15 | LTQDQVVAIASHDGGKQALETVQRLLPVLCQDHG |
| | 16 | LTPDQVVAIASHDGGKQALETVQRLLPVLCQEHG |
| | 17 | LTPDQVVAIASHDGGKQALETVQRLLPVLCQDHG |
| | 18 | LTPDQVVAIASHGGGKQALGTVQRLLPVLCQDHG |
| | 19 | LTPDQVVAIANNNGGKQALETVQRLLPVLCQDHG |
| | 20 | LTPDQVVAIANNNGGKQALETVQRLLPVLCQAHG |
| | 21 | LTPDQVVAIASHDGGKQALETVQRLLPVLCQDHG |
| | 22 | LTPAQVVAIASNSGGKQALETVQRLLPVLCQDHG |
| | 23 | LTPAQVVAIANNNGGKQALETVQRLLPVLCQDHG |
| | 24 | LTPDQVVAIASHDGGKQALETVQRLLPVLCQDHG |
| | 25 | LTPDQVVAIASNGGGKQALATVQRLLPVLCQAHG |
| | 26 | LTPDQVVAIASNSGGKQALETVQRLLPVLCQDHG |
| | 27 | LTPNQVVAIASN*GGKQALE |
| 4 | 1 | LTPDQVVAIASHDGGKQALETVQRLLPVLCQAHG |
| | 2 | LTPAQVVAIASHDGGKQALETVQRLLPVLCQAHG |
| | 3 | LTPAQVVAIASNNGGKQALETVQRLLPVLCQAHG |
| | 4 | LTPDQVVAIASNNGGKQALETVQRLLPVLCQAHG |
| | 5 | LTPDQVVAIASNIGGKQALETVQRLLPVLCQDHG |
| | 6 | LTPDQVVAIASNGGGKQALETVQRLLPVLCQAHG |
| | 7 | LPPAQVVAIASHDGGKQALETVQRLLPVLCQDHG |
| | 8 | LTPAQVVAIASS*GGKQALETVQRLLPVLCQAHG |

| | | |
|---|---|---|
| | 9 | LTLDQVVAIASHGGSKQALETVQRLLPVLCQDHG |
| | 10 | LTPDQVVAIASHDGGKQALETVQRLLPVLCQAHG |
| | 11 | LTPDQVVAIASNGGGKQALETVQRLLPVLCQDHG |
| | 12 | LTPDQVVAIASN*GGKQALETVQRLLPVLCQDHG |
| | 13 | LTPDQVVAIASNGGGKQALATVQRLLPVLCQAHG |
| | 14 | LTPDQVVAIASHDGGKQALETVQRLLPVLCQDHG |
| | 15 | LTPDQVVAIASHDGGKQALETVQRLLPVLCQAHG |
| | 16 | LTPDQVVAIASN*GGKQALETVQRLLPVLCQAHG |
| | 17 | LTLAQVVAIASNIGGKQALETVQRLLPVLCQDHG |
| | 18 | LTPDQVVAIASNIGGKQALETVQRLLPVLCQDHG |
| | 19 | LTPDQVVAIASNNGGKQALETVQRLLPVLCQDHG |
| | 20 | LTLDQVVAIASHDGGKQALETVQRLLPVLCQAHG |
| | 21 | LTPAQVVAIASHIGGKQALETVQRLLPVLCQDHG |
| | 22 | LTLDQVVAIASNDGSKQALETVQRLLPVLCQDHG |
| | 23 | LTPDQVVAIASHDGGKQALETVQRLLPVLCQDHG |
| | 24 | LTPDQVVAIASNGGGKQALETVQRLLPVLCQDHG |
| | 25 | LTPAQVVAIANNNGGKQALETVQRLLPVLCQDHG |
| | 26 | LTPDQVVAIASHGGGKQALETVQRLLPVLCQDHG |
| | 27 | LTPDQVVAIASN*GGKQALE |
| **5** | 1 | LTPAQVVAIASNNGGKQALETVQRLLPLLCQAHG |
| | 2 | LTPAQVVAIASHDGGKQALETVQRLLPVLCQDHG |
| | 3 | LTPDQVVAIASNSGGKQALETVQRLLPVLCQDHG |
| | 4 | LTPDQVVAIASNGGGKQALETVQRLLPVLCQDHG |
| | 5 | LTPDQVVAIASHDGGKQALETVQRLLPVLCQDHG |
| | 6 | LTPDQLVAIANNNGGKQALETVQRLLPVLCQDHG |
| | 7 | LTPDQVVAIASN*GGKQALETVQRLLPVLCQDHG |
| | 8 | LTPDQVVAIASNIGGKQALETVQRLLPVLCQAHG |
| | 9 | LTPAQVVAIASHDGGKQALETMQRLLPVLCQAHG |
| | 10 | LTPAQVVAIASNSGGKQALETVQRLLPVLCQAHG |
| | 11 | LTPAQVVAIVSHDGGKQALETVQRLLPVLCQAHG |
| | 12 | LTPDQVVAIASNNGGKQALETVQRLLPVLCQDHG |
| | 13 | LTPDQVVAIASHDGGKQALGTVQRLLPVLCQDHG |
| | 14 | LTPDQVVAIASNNGGKQALETVQRLLPVLCQDHG |
| | 15 | LTPDQVVAIASHDGGKQALETVQRLLPVLCQAHG |
| | 16 | LTPAQVVAIASNNGGKQALETVQRLLPVLCQDHG |
| | 17 | LTPDQVVAIANNNGGKQALETVQRLLPVLCQAHG |
| | 18 | LTPAQVVAIASNNGSKQALETVQRLLPVLCQAHG |
| | 19 | LTPAQVVAIASNNGGKQALETVQRLLPVLCQAHG |
| | 20 | LTPDQVVAIASHDGGKQALETVQRLLPVLCQAHG |
| | 21 | LTPDQVVAIASNNGGKQALETVQRLLPVLCQAHG |
| | 22 | LTPDQVVAIASNNGGKQALETVQRLLPVLCQDHG |
| | 23 | LTPAQVVAIASHDGGKQALETVQRLLPVLCQDHG |
| | 24 | LTPAQVVAIASNGGGKQALE |

| | | |
|---|---|---|
| 6 | 1 | LTPDQVVAIASNIGGKQALETVQRLLPVLCQDHG |
| | 2 | LTPDQVVAIANNNGGKQALETVQRLLPVLCQDHG |
| | 3 | LTPDQVVAIASNIGGKQALETVQRLLPVLCQAHG |
| | 4 | LTPAQVVAIASNNGGKQALETVQRLLPVLCQAHG |
| | 5 | LTPDQVVAIASNNGGKQALETVQRLLPVLCQAHG |
| | 6 | LTPDQVVAIANNNGGKQALETVQRLLPVLCQDHG |
| | 7 | LSPDQVVAIANNNGGKQALETLQRLLPVLCQTHA |
| | 8 | LTPDQVVAIANNNGGKQALETVQRLLPVLCQDHG |
| | 9 | LTPDQVVAIASHDGGKQALETVQRLLPVLCQDHG |
| | 10 | LTPDQVVAIASNIGGKQALETVQRLLPVLCQDHG |
| | 11 | LTPDQVVAIASNSGGKQALETVQRLLPVLCQDHG |
| | 12 | LTPDQVVAIASHGGGKQALETVQRLLPVLCQDHG |
| | 13 | LTPDQVVAIASHDGGKQALETVQRLLPVLCQAHG |
| | 14 | LTPNQVVAIASNIGGKQALETVQRLLPVLCQDHG |
| | 15 | LTPDQVVAIASN*GGKQALETVQRLLPVLCQAHG |
| | 16 | LTPDQVVAIASNSGGKQALETVQRLLPVLCQDHG |
| | 17 | LTPNQVVAIASNIGGKQALETVQRLLPVLCQDHG |
| | 18 | LTPDQVVAIASNIGGKQALETVQRLLPVLCQDHG |
| | 19 | LTLDQVVAIASHDGGKQALETVQRLLPVLCQAHG |
| | 20 | LTPDQVVAIASHDGGKQALETVQRLLPVLCQAHG |
| | 21 | LTLDKVVAIASN*GGKQALETVQRLLPVLCQDHG |
| | 22 | LTPNQVVAIASNSGGKQALETVQRLLPVLCQDHG |
| | 23 | LTPNQVVAIASN*GGKQALE |
| 7 | 1 | LTPDQVVAIASNIGGKQALETVQRLLPVLCQAHG |
| | 2 | LTPDQVVAIASHGGGKQALETVQRLLPVLCQDHG |
| | 3 | LTPDQVVAIASNIGGKQALETVQRLLPVLCQDHG |
| | 4 | LTPDQVMAIANNNGGKQALETVQRLLPVLCQDHG |
| | 5 | LTPDQVMAIANNNGGKQALETVQRLLPVLCQDHG |
| | 6 | LTPDQVVAIASNIGGKQALETVQRLLPVLCQDHG |
| | 7 | LTPTQVMAIANNNGGKQALETVQRLLPVLCQDHG |
| | 8 | LTPDQVVAIASHDGGKQALETVQRLLPVLCQDHG |
| | 9 | LTPAQVVAIASNSGGKQALETVQRLLPVLCQDHG |
| | 10 | LTPDQVVAIASHDGGKQALETVQRLLPVLCQDHG |
| | 11 | LTPDQVVAIASNSGGKQALETVQRLLPVLCQDHG |
| | 12 | LTPDQVVAIASNSGGKQALETVQRLLPVLCQDHG |
| | 13 | LTPAQVVAIASNSGGKQALETVQRLLPVLCQDHG |
| | 14 | LTPDQVVAIASHDGGKQALETVQRLLPVLCQDHG |
| | 15 | LTPDQVVAIANNNGGKQALETLQRLLPVLCQDHG |
| | 16 | LTPDQVVAIASHDGGKQALETVQRLLPVLCQDHG |
| | 17 | LTPDQVVAIASNGGGKQALETVQRLLPVLCQDHG |
| | 18 | LTPDQVVAIASHDGGKQALETVQRLLPVLCQDHG |
| | 19 | LTPAQVVAIASHDGGKQALETVQRLLPVLCQDHG |
| | 20 | LTPDQVVAIASHDGGKQALETVQRLLPVLCQDHG |

| | | |
|---|---|---|
| | 21 | LTPDQVVAIASNGGGKQALKTVQRLLPVLCQDHG |
| | 22 | LTPDQVVAIASNGGGKQALE |
| 8 | 1 | LTPDQVVAIASNIGGNQALETVQRLLPVLCQAHG |
| | 2 | LTPDQVVAIASHGGGKQALETLQRLLPVLCQDHG |
| | 3 | LTPDQVVAIASNIGGKQALETVQRLLPVLCQDHG |
| | 4 | LTPDQVVAIASNGGGKQALETLQRLLPVLCQAHG |
| | 5 | LTPDQVLAIASHGGGKQALETLQRLLPVLCQDHG |
| | 6 | LTPAQVVALASHDGGKQALETVQRLLPVLCQDHG |
| | 7 | LTPAQVVAIASNSGGKQALETVQRLLPVLCQDHG |
| | 8 | LTPDQVVAIASNGGGKQALETVQRLLPVLCQDHG |
| | 9 | LTPDQVVAIASHDGGKQALETVQRLLPVLCQDHG |
| | 10 | LTPDQVVAIANNNGGKQALETVQRLLLVLCQAHG |
| | 11 | LTPDQVVAIASNGGGKQALETVQRLLPVLCQDHG |
| | 12 | LTPDQVVAIASHGGGKQALETVQRLLPVLCQDHG |
| | 13 | LTPDQVVAIASNGGGKQALETVQRLLPVLCQDHG |
| | 14 | LTPDQVVAIASHDGGKQALETVQRLLPVLCQDHG |
| | 15 | LTPDQVVAIASHGGGKQALETVQRLLPVLCQDHG |
| | 16 | LTPDQVVAIASHDGGKQALETVQRLLPVLCQDHG |
| | 17 | LTPDQVVAIASHDGGKQALETVQRLLPVLCQDHG |
| | 18 | LTLDQVVAIASNIGGKQALETVQRLLPVLCQDHG |
| | 19 | LTPDQVVAIANNNGGKQALETVQRLLPVLCQDHG |
| | 20 | LTPDQVVAIASNGGGKQALE |
| 9 | 1 | LTPDQVVAIASNIGGKQALETVQRLLPVLCQAHG |
| | 2 | LTPDQVVAIASNIGGKQALETVQRLLPVLCQDHG |
| | 3 | LTPDQVVAIASHGGGKQALETVQRLLPVLCQDHG |
| | 4 | LTPDQVVAIASNIGGKQALETVQRLLPVLCQDHG |
| | 5 | LIPDQVVAIASNIGGKQALETVQRLLPVLCQDHG |
| | 6 | LTPDQVVAIASNSGGKQALETVQRLLPVLCQAHG |
| | 7 | LTPDQVVAIASHDGGKQALETVQRLLPVLCQDHG |
| | 8 | LTPAQVVAIASNNGGKQALETVQRLLPVLCQAHG |
| | 9 | LTPDQVVAIASHDGGKQALETVQRLLPVLCQDHG |
| | 10 | LTPAQVVAIASNSGGKQALETVQRLLPVLCQDHG |
| | 11 | LTPDQVVAIASNGGGKQALETVQRLLPVLCQDHG |
| | 12 | LTPDQVVAIASSSGGKQALETVQRLLPVLCQAHG |
| | 13 | LTPDQVVAIASHDGGKQALETVQRLLPVLCQDHG |
| | 14 | LTPDQVVAIASNIGGKQALETVQRLLPVLCQDHG |
| | 15 | LTPDQVVAIASNIGGKQALETVQRLLPVLCQDHG |
| | 16 | LTPDQVVAIANNNGGKQALETVQRLLPVLCQDHG |
| | 17 | LTPDQVVAIASNIGGKQALETVQRLLPVLCQDHG |
| | 18 | LTPAQVVAIANNNGGKQALETVQRLLPVLCQDHG |
| | 19 | LTPDQVVAIASNIGGKQALETVQRLLPVLCQDHG |
| | 20 | LTPDQVVAIASNGGGKQALE |
| **10** | 1 | LTPDQVVAIASNIGGNQALETVQRLLPVLCQAHG |

| | | |
|---|---|---|
| | 2 | LTPDQVVAIASHGGGKQALETVQRLLPVLCQDHG |
| | 3 | LTPDQVVAIASNIGGKQALETVQRLLPVLCQDHG |
| | 4 | LTPDQVVAIASNIGGKQALETVQRLLPVLCQDHG |
| | 5 | LTPDQVVAIASNIGGKQALETVQRLLPVLCQDHG |
| | 6 | LTPDQVVAIANNNGGKQALETVQRLLPVLCQDHG |
| | 7 | LTPDQVVAIASHDGGKQALETVQRLLPVLCQGHG |
| | 8 | LTPDKVVAIASNSGGKQALETVQRLLPVLCQAHG |
| | 9 | LTPDKVVAIANNNGGKQALETVQRLLPVLCQDHG |
| | 10 | LTPDQVVAIASNSGGKQALETVQRLLPVLCQDHG |
| | 11 | LTPAQVVAIANNNGGKQALETVRRLLPVLCQDHG |
| | 12 | LTPDQVVAIASHDGGKQALETVQRLLPVLCQDHG |
| | 13 | LTPDQVVAIANNNGGKQALETVQRLLPVLCQDHG |
| | 14 | LTQDQVVAIASNIGGKQALETVQRLLPVLCQDHG |
| | 15 | LTLDQVVAIASHDGGKQALETVQRLLPVLCQDHG |
| | 16 | LTPAQVVAIANNNGGKQALETVQRLLPVLCQDHG |
| | 17 | LSPDQVVAIASNIGGKQALETVQRLLPVLCQDHG |
| | 18 | LTPDQVVAIASNGGGKQALETVQRLLPVLCQDHG |
| | 19 | LSPDQVVAIASHDGGKQALETVQRLLPVLCQDHG |
| | 20 | LTPDQVVAIASNGGGKQALE |
| **11** | 1 | LTPDQVVAIASNIGGKQALETVQRLLPVLCQDHG |
| | 2 | LTPDQVVAIASNGGGKQALETVQRLLPVLCQDHG |
| | 3 | LTPDQVVAIASNNGGKQALETVQRLLPVLCQDHG |
| | 4 | LTPDQVVAIASNGGGKQALETVQRLLPVLCQDHG |
| | 5 | LTPDQVVAIANNKGGKQALETLQRLLPVLCQAHG |
| | 6 | LTPDQVVAIASNGGGKQALETVQRLLPVLCQDHG |
| | 7 | LTPAQVVAIASNIGGKQALETVQRLLPVLCQDHG |
| | 8 | LTPDQVVAIANNNGGKQALETVQRLLPVLCQDHG |
| | 9 | LTPDQVVTIASNIGGKQALETVQRLLPVLCQDHG |
| | 10 | LTPDQVVAIANNNGGKQALETVQRLLPVLCQAHG |
| | 11 | LTPDQVVAIASNIGGKQTLETVQRLLPVLCQDHG |
| | 12 | LTPDQVVAIANNNGGKQALETVQRLLPVLCQDHG |
| | 13 | LTPDQVVAIASNSGGKQALETVQRLLPVLCQDHG |
| | 14 | LTPNQVVAIASNGGGKQALETVQRLLPVLCQDHG |
| | 15 | LTPDQVVGIASNSGGKQALETVQRLLPVLCQDHG |
| | 16 | LTPDQVVAIANNNGGKQALETVQRLLPVLCQDHG |
| | 17 | LTPDQVVAIASNIGGKQALETVQRLLPVLCQDHG |
| | 18 | LTLDKVVAIASN*GGKQALETVQRLLPVLCQDHG |
| | 19 | LTPDQVVAIASNSGGKQALETVQRLLPVLCQDHG |
| | 20 | LTPDQVVAIASNGGGKQALE |
| **12** | 1 | LTPAQVVAIASHDGGNQALETVQRLLPVLCQDHG |
| | 2 | LTPAQVVAIASHDGGKQALETVQRLLPVLCQDHG |
| | 3 | LTPDQVVAIASHDGGKQALETVQRLLPVLCQDHG |
| | 4 | LTPAQAVAIASNGGGKQALETVQRLLPVLCQDHG |

| | | |
|---|---|---|
| | 5 | LTPDQVVAIASN*GGKQALETVQRLLPVLCQDHG |
| | 6 | LTPDQVVAIASNNGGKQALETVQRLLPVLCQDHG |
| | 7 | LTPDQVVAIASHDGGKQALETVQRLLPVLCQDHG |
| | 8 | LTPDQVVAIASHDGGKQALETVQRLLPVLCQDHG |
| | 9 | LTPDQVVAIASN*GGKQALETVQRLLPVLCQDHG |
| | 10 | LTLAQVVAIASNIGGKQALETVQRLLPVLCQDHG |
| | 11 | LTPDQVVAIASNIGGKQALETVQRLLPVLCQDHG |
| | 12 | LTPDQVVAIASNNGGKQALETVQRLLPVLCQDHG |
| | 13 | LTPDQVVAIASHDGGKQALETVQRLLPVLCQDHG |
| | 14 | LTPAQVVAIASHIGGKQALETVQRLLPVLCQDHG |
| | 15 | LTLDQVVAIASNDGSKQALETVQRLLPVLCQDHG |
| | 16 | LTPDQVVAIASHDGGKQALETVQRLLPVLCQDHG |
| | 17 | LTPNQVVAIASNIGGKQALETVQRLLPVLCQAHG |
| | 18 | LTPDQVVAIASHDGGKQALETVQRLLPVLCQNHG |
| | 19 | LTPDQVVAIASNGGGKQALETVQRLLPVLCQDHG |
| | 20 | LTPDQVVAIASNGGGKQALE |
| **13** | 1 | LTPDQVVAIASNIGGNQALETVQRLLPVLCQAHG |
| | 2 | LTPDQVVAIASN*GGKQALETVQRLLPVLCQDHS |
| | 3 | LTPDQVVAIASNIGGKQALETVQRLLPVLCQDHG |
| | 4 | LTPDQVVAIASNSGGKQALETVQRLLPVLCQDHG |
| | 5 | LTPDQVMTIASNNGGKQALETVQRLLPVLCQDHG |
| | 6 | LTPDQVVTIASNGGGKQALETVQRLLPVLCQAHG |
| | 7 | LTPDQVVAIANNNGGKQALETVQRLLPVLCQAHG |
| | 8 | LTPAQVVAIASNSGGKQALETVQRLLPVLCQAHG |
| | 9 | LTPDQVVAIASN*GGKQALETVQRLLPVLCQAHG |
| | 10 | LTPDQVVAIASNSGGKQALETVQRLLPVLCQAHG |
| | 11 | LTPDQVVAIANNNGGKQALETVQRLLPVLCQAHG |
| | 12 | LTPDQVVAIASNSGGKQALETVQRLLPVLCQDHG |
| | 13 | LTPDQVVAIASN*GGKQALETVQRLLPVLCQDHG |
| | 14 | LTPDQVVAIASHDGGKQALETVQRLLPVLCQDHG |
| | 15 | LTLDQVVAIASHGGGKQALETVQRLLPVLCQDHG |
| | 16 | LTSDQVVAIASHDGGKQALETVQRLLPVLCQDHG |
| | 17 | LTPDQVVAIASNIGGKQALETVQRLLPVLCQDHG |
| | 18 | LTLDQVVAIASHDGGKQALETVQRLLPVLCQDHG |
| | 19 | LTPDQVVAIASHDGGKQALETVQRLLPVLCQDHG |
| | 20 | LTPAQVVAIASNGGGKQALE |
| **14** | 1 | LTPDQVVAIASNIGGKQALETVQRLLPVLCQAHG |
| | 2 | LNPDQVVAIASNSGGKQALETVQRLLPVLCQDHG |
| | 3 | LTPDQVVAIASHDGGKQALETVQRLLPVLCQDHG |
| | 4 | LTPDQVVAIASNGGGKQALETVQRLLPVLCQAHG |
| | 5 | LNPDQVVAIASNSGGKQALETVQRLLPVLCQAHG |
| | 6 | LNPDQVVAIASNNGGKQALETVQRLLPVLCQDHG |
| | 7 | LSPDQVVAIASHDGGKQALETVQRLLPVLCQDHG |

| | | |
|---|---|---|
| | 8 | LTPDQVVAIASN*GGKQALETVQRLLPVLCQDHG |
| | 9 | LTPDQVVAIANNNGGKQALETVQRLLPVLCQDHG |
| | 10 | LTPDQVVAIANNNGGKQALETVQRLLPVLCQAHG |
| | 11 | LPPDQVVAIASNIGGKQALETVQRLLPVLCQDHG |
| | 12 | LTPDQVVAIANNNGGKQALETVQRLLPVLCQDHG |
| | 13 | LTPDQVVAIASHDGGKQALETVQRLLPVLCQDHG |
| | 14 | LTPDQVVAIASHGGGKQALETVQRLLPVLCQDHG |
| | 15 | LSPDQVVAIASHDGGKQALETVQRLLPVLCQDHG |
| | 16 | LTLDQVVAIASHDGGKQALETVQRLLPVLCQDHG |
| | 17 | LTPAQVVAIANNNGGKQALETVQRLLPVLCQDHG |
| | 18 | LTPDQVVAIASNGGGKQALE |
| **15** | 1 | LTPDQVVAIASNSGGKQALETVQRLLPVLCQDHG |
| | 2 | LTPDQVVAIASHDGGKQALETVQRLLPVLCQDHG |
| | 3 | LTPDQVVAIASNGGGKQALETVQRLLPVLCQDHG |
| | 4 | LTPDQVVAIASNGGGKQALETVLCQAHG |
| | 5 | LTPAQVVAIASNGGGKQALETVQRLLPVLCQAHG |
| | 6 | LTLDQVVAIASNGGGKQALETVQRLLPVLCQAHG |
| | 7 | LTPDQVVAIASHDGGKQALETVQRLLPVLCQAHG |
| | 8 | LTPAQVVAIASHDGGKQALETVQRLLPVLCQAHG |
| | 9 | LTLDQVVAIASHDGGKQALETVQRLLPVLCQAHG |
| | 10 | LTLDQVVAIASHDGGKQALETVQRLLPVLCQDHG |
| | 11 | LTPAQVVAIASNNGGKQALETVQRLLPVLCQDHG |
| | 12 | LTPDQVVAIASHDGGKQALETMQRLLPVLCQAHG |
| | 13 | LTPDQVVAIASNGGGKQALETVQRLLPVLCQAHG |
| | 14 | LTPDQVVAIASHDGGKQALETVQRLLPVLCQDHG |
| | 15 | LTPDQVVAIASNIGGKQALETVQRLLPVLCQDHG |
| | 16 | LTPDQVVAIASHDGGKQALETVQRLLPVLCQDHG |
| | 17 | LTPDQVVAIANNNGGKQALETVQRLLPVLCQDHG |
| | 18 | LTPDQVVAIASN*GGKQALE |
| **16** | 1 | LTPDQVVAIASNIGGNQALETVQRLLPVLCQDHG |
| | 2 | LTPDQVVAIANNNGGKQALETVQRLLPVLCQAHG |
| | 3 | LTPDQVVAIASN*GGKQALETVQRLLPVLCQAHG |
| | 4 | LTPDQVVAIASNGGGKQALETVQRLLPVLCQAHG |
| | 5 | LTPAQVVAIASNSGGKQALETVQRLLPVLCQDHG |
| | 6 | LTPAQVVAIANNNGGKQALETVQRLFPVLCQDHG |
| | 7 | LTPDQVVTIANNNGGKQALETVQRLLPVLCQAHG |
| | 8 | LIPDQVVAIANNNGGKQALETVQRLLPVLCQAHG |
| | 9 | LTPAQVVAIASNIGGKQALETVQRLLPVLCRAHG |
| | 10 | LTPAQVVAIANNNGGKQALETVQRLLPVLCQDHG |
| | 11 | LTPDQVVAIASNIGGKQALETVQRLLPVLCQAHG |
| | 12 | LTPDQVVAIASNGGGKQALETVQRLLPVLCQDHG |
| | 13 | LTPDQVVAIAGHDGGKQALETVQRLLPVLCQDHG |
| | 14 | LTPDQVVAIASHDGGKQALETVQRLLPVLCQDHG |

| | | |
|---|---|---|
| | 15 | LTLDQVVAIASNIGGKQALETVQRLLPVLCQDHG |
| | 16 | LTPDQVVAIASNGGGKQALE |
| **17** | 1 | LTPDQVVAIASNIGGKQALETVQRLLPVLCQAHG |
| | 2 | LTPDQVVAIANNNGGKQALETVQRLLPVLCQAHG |
| | 3 | LTPAQVVAIASNNGGKQALETVQRLLPVLCQDHG |
| | 4 | LTPDQVVAIASNIGGKQALETVQRLLPVLCQDHG |
| | 5 | LTPDQVVAIASNIGGKQALETVQRLLPVLCQTHA |
| | 6 | LTPDQVVAIASNIGGKQALETVQRLLPVLCQDHG |
| | 7 | LTPAQVVAIASHDGGKQALETVQRLLPVLCQDHG |
| | 8 | LTPDQVVAIASNSGGKQALETVQRLLPVLCQAHG |
| | 9 | LTLDQVVAIASHGGGKQALETVQRLLPVLCQDHG |
| | 10 | LTPDQVVAIASNNGGKQALETVQRLLPVLCQDHG |
| | 11 | LIPDQVVAIANNNGGKQALETVQRLLPVLCQAHG |
| | 12 | LTTDQVVTIASNNGGKQALETVQRLLPVLCQDHG |
| | 13 | LTPDQVVAIASNIGGKQALETVQRLLPVLCQDHG |
| | 14 | LTPDQVVAIASNIGGKQALETVQRLLPVLCQDHG |
| | 15 | LTPDQVVAIASNGGGKQALETVQRLLPVLCQDHG |
| | 16 | LTPDQVVAIASHDGGKQALE |
| **18** | 1 | LTPDQVVAIASNIGGKQALETVQRLLPVLCQDHG |
| | 2 | LTPDQVVAIANNNGGKQALETVQRLLPVLCQDHG |
| | 3 | LTPDQVVAIASNIGGKQALETVQRLLPVLCQDHG |
| | 4 | LTPDQVVAIASHGGGKQALETVQRLLPVLCQDHG |
| | 5 | LTPDQVVAIASHGGGKQALETLQRLLPVLCQDHG |
| | 6 | LTPDQVVAIASHDGGKQALETVQRLLPVLCQDHG |
| | 7 | LTPDQVVAIASNGGGKQALETLQRLLPVLCQDHG |
| | 8 | LTPDQVVAIASHDGGKQALETVQRLLPMLCQDHG |
| | 9 | LTPDQVVAIASHGGGKQALETVQRLLPVLCQDHG |
| | 10 | LTPDQVVAIASHDGGKQALETVQRLLPVLCQDHG |
| | 11 | LTLDQVVAIASHDGGKKALETVQRLLPVLCQDHG |
| | 12 | LTPDQVVAIASHDGGKQALETVQRLLPVLCQDHG |
| | 13 | LTPDQVVAIASNGGGKQALE |
| **TalC** | 1 | LTPAQVVAIASNSGGKQALETVQRLLPVLCQAHG |
| | 2 | LTPEQVVAIASNGGGKQALETVQRLLPVLCQAHG |
| | 3 | LTPAQVVAIASNSGGKQALETVQRLLPVLCQAHG |
| | 4 | LTPDQVVAIASHDGGKQALETVQRLLPVLCQAHG |
| | 5 | LTPEQVVAIASNIGGKQALETVQRLLPVLCQAHG |
| | 6 | LTPEQVVAIASNGGGKQALETVQRLLPMLCQAHG |
| | 7 | LTPEQVVAIASNNGGKQALETVQRLLPVLCQAHG |
| | 8 | LTPEQVVAIASNGGGKQALETVQRLLPVLCQAHG |
| | 9 | LTPDQVVAIASHDGGKQALETVQRLLPVLCQAHG |
| | 10 | LTPAQVVAIASNIGGKQALETVRRLLPVLCQAHG |
| | 11 | LTPAQVVAIANNNGGKQALETVQRLLPVLCQAHG |
| | 12 | LTPEQVVAIASN*GGKQALETVQRLLPVLCQAHG |

| | | |
|---|---|---|
| | 13 | LTPEQVVAIASNIGGKQALETVQRLLPVLCQAHG |
| | 14 | LTPEQVVAIASNNGGKQALETVQRLLPVLCQAHG |
| | 15 | LTPDQVVAIASHDGGKQALETVQRLLPVLCQAHG |
| | 16 | LTLEQVVAIASNGGGKQALETVQRLLPVLCQAHG |
| | 17 | LTPAQVVAIACNIGGKQALETVRRLLPVLCQAHG |
| | 18 | LTPAQVVAIANNNGGKQALETVQRLLPVLCQAHG |
| | 19 | LTPAQVVAIASN*GGKQALETVQRLLPVLCQAHG |
| | 20 | LTPAQVVAIASHDGGKQALETVQRLLPVLCQAHG |
| | 21 | LTPDQVVAIASNNGGKQALETVQRLLPVLCQAHG |
| | 22 | LTPEQVVAIASNGGGKQALE |

[a] The RVDs are marked in red; *represents a missing amino acid in the RVDs.

Supplementary Table 7. Comparison of the RVD strings of the TALEs issued from *Xoo* strains K74, PXO99$^{A}$ and MAFF311018.

| ***tal* name ( accession number)** | *Xoo* strain | Repeat num. | 1 | 2 | 3 | 4 | 5 | 6 | 7 | 8 | 9 | 10 | 11 | 12 | 13 | 14 | 15 | 16 | 17 | 18 | 19 | 20 | 21 | 22 | 23 | 24 | 25 | 26 | 27 | 28 | 29 | 30 | 31 |
|---|---|---|---|---|---|---|---|---|---|---|---|---|---|---|---|---|---|---|---|---|---|---|---|---|---|---|---|---|---|---|---|---|---|
| *tal*$_{Xoo\ K74}$-4 (KP711414) | K74 | 26.5 | HD | HD | NN | NN | NI | NG | HD | S* | HG | HD | NG | N* | NG | HD | HD | N* | NI | NI | NN | HD | HI | ND | HD | NG | NN | HG | N* | | | | |
| *tal9b* (ACD60561.1) | PXO99$^{A}$ | 26.5 | HD | HD | NN | NN | NG | NG | HD | NS | HG | HD | NG | N* | HD | HD | HD | N* | NN | NQ | NN | HD | HI | ND | HD | HG | NN | HG | N* | | | | |
| *XOO_1136* (YP_450165.1) | MAFF311018 | 26.5 | HD | HD | NN | NN | NS | NG | HD | NS | HG | HD | NG | N* | HD | HD | HD | N* | NN | NQ | NN | HD | HI | ND | HD | HG | NN | HG | N* | | | | |
| *tal*$_{Xoo\ K74}$-16 (KP711426) | K74 | 15.5 | NI | NN | N* | NG | NS | NN | NN | NN | NI | NN | NI | NG | HD | HD | NI | NG | | | | | | | | | | | | | | | |
| *avrXa27* (ACD60565.1) | PXO99$^{A}$ | 16.5 | NI | NN | N* | NG | NS | NN | NN | NN | NI | NN | NI | N* | HD | HD | NI | NG | NG | | | | | | | | | | | | | | |
| *avrXa27* (YP_450163.1) | MAFF311018 | 16.5 | NI | NN | N* | NG | NS | NN | NN | NN | NI | NN | NI | N* | HD | HD | NI | NG | NG | | | | | | | | | | | | | | |
| *tal*$_{Xoo\ K74}$-12 (KP711422) | K74 | 19.5 | HD | HD | HD | NG | N* | NN | HD | HD | N* | NI | NI | NN | HD | HI | ND | HD | NI | HD | NG | NG | | | | | | | | | | | |
| *tal9a* (ACD60557.1) | PXO99$^{A}$ | 19.5 | HD | HD | HD | NG | N* | NN | HD | HD | N* | NI | NI | NN | HD | HI | ND | HD | NI | HD | NG | NG | | | | | | | | | | | |
| *XOO_1138* (YP_450167.1) | MAFF311018 | 19.5 | HD | HD | HD | NG | N* | NG | HD | S* | HG | NI | NI | NN | HD | NN | ND | HD | NI | HD | HG | NG | | | | | | | | | | | |
| *tal*$_{Xoo\ K74}$-10 (KP711420) | K74 | 19.5 | NI | HG | NI | NI | NI | NN | HD | NS | NN | NS | NN | HD | NN | NI | HD | NN | NI | HG | HD | NG | | | | | | | | | | | |
| *talC7a* (ACD59223.1) | PXO99$^{A}$ | 17.5 | NI | HG | NI | NI | NI | NN | HD | NS | NN | NS | NN | HD | NN | NI | HD | NN | NS | NG | | | | | | | | | | | | | |
| *XOO_1998* (YP_451027.1) | MAFF311018 | 19.5 | NI | HG | NI | NI | NI | NN | HD | NS | NN | NS | NN | HD | NN | NI | HD | NN | NI | NG | HD | NG | | | | | | | | | | | |
| *tal*$_{Xoo\ K74}$-18 (KP711428) | K74 | 12.5 | NI | NN | NI | HG | HG | HD | NG | HD | HG | HD | HD | HD | NG | | | | | | | | | | | | | | | | | | |
| *talC9d* (ACD60569.1) | PXO99$^{A}$ | 12.5 | NI | NN | NI | HG | HG | NV | HG | HD | HG | HD | HD | HD | NG | | | | | | | | | | | | | | | | | | |
| *XOO_1132* (YP_450161.1) | MAFF311018 | 12.5 | NI | NN | NI | HG | HG | NV | HG | HD | HG | HD | HD | HD | NG | | | | | | | | | | | | | | | | | | |
| *tal*$_{Xoo\ K74}$-13 (KP711423) | K74 | 19.5 | NI | N* | NI | NS | NN | NG | NN | NS | N* | NS | NN | NS | N* | HD | HG | HD | NI | HD | HD | NG | | | | | | | | | | | |
| *talC6a* (ACD58948.1) | PXO99$^{A}$ | 19.5 | NI | N* | NI | NS | NN | NG | NN | NS | N* | NS | NN | NS | N* | NI | HG | HD | NI | HD | HD | NG | | | | | | | | | | | |
| *XOO_2158* (YP_451187.1) | MAFF311018 | 19.5 | NI | N* | NI | NS | NN | NG | NN | NS | N* | NS | NN | NS | N* | HD | HG | HD | NI | HD | HD | NG | | | | | | | | | | | |
| *tal*$_{Xoo\ K74}$-5 (KP711415) | K74 | 23.5 | NN | HD | NS | NG | HD | NN | N* | NI | HD | NS | HD | NN | HD | NN | HD | NN | NN | NN | NN | HD | NN | NN | HD | NG | | | | | | | |
| *talC9e* (ACD60573.1) | PXO99 | 23.5 | NN | HD | NS | NG | HD | NN | N* | NI | HD | NS | HD | NN | HD | NN | HD | NN | NN | NN | NN | NN | NN | NN | HD | NG | | | | | | | |
| *Xoo_2001* (YP_451030.1) | MAFF311018 | 23.5 | NN | HD | NS | NG | HD | NN | N* | NI | HD | NS | HD | NN | HD | NN | HD | NN | NN | NN | NN | NN | NN | NN | HD | NG | | | | | | | |
| *tal*$_{Xoo\ K74}$-1 (KP711411) | K74 | 16.5 | NS | HD | NG | NG | NG | NG | HD | HD | HD | HD | NN | HD | HD | HD | HD | NN | H* | | | | | | | | | | | | | | |
| *tal*$_{Xoo\ K74}$-15 (KP711425) | K74 | 17.5 | NS | HD | NG | NG | NG | NG | HD | HD | HD | HD | NN | HD | NG | HD | NI | HD | NN | N* | | | | | | | | | | | | | |
| *talC3b* (ACD58455.1) | PXO99 | 17.5 | NS | HD | NG | NG | NG | NG | NG | HD | HD | HD | NN | HD | NG | HD | HD | HD | HD | N* | | | | | | | | | | | | | |
| *XOO_2667* (YP_451696.1) | MAFF311018 | 17.5 | NS | HD | NG | NG | NG | NG | HD | HD | HD | HD | NN | HD | NG | HD | HD | HD | NN | H* | | | | | | | | | | | | | |
| *tal*$_{Xoo\ K74}$-17 (KP711427) | K74 | 15.5 | NI | NN | NN | NI | NI | NI | HD | NS | HG | NN | NN | NN | NI | NI | NG | HD | | | | | | | | | | | | | | | |
| *talC4* (ACD58650.1) | PXO99$^{A}$ | 15.5 | NI | NN | NN | NI | NI | NI | HD | NS | HG | NN | NN | NN | NI | NI | HG | HD | | | | | | | | | | | | | | | |
| *XOO_2129* (YP_451158.1) | MAFF311018 | 15.5 | NI | NN | NN | NI | NI | NI | HD | NS | HG | NN | NN | NN | NI | NI | NG | HD | | | | | | | | | | | | | | | |
| *tal*$_{Xoo\ K74}$-14 (KP711424) | K74 | 17.5 | NI | NS | HD | NG | NS | NN | HD | N* | NN | NN | NI | NN | HD | HG | HD | HD | NN | NG | | | | | | | | | | | | | |
| *talC5a* (ACD58925.1) | PXO99$^{A}$ | 15.5 | NI | NS | HD | HG | NS | NN | HD | H* | NG | NN | NN | HD | HD | NG | HD | NG | | | | | | | | | | | | | | | |
| *XOO_1996* (YP_451025.1) | MAFF311018 | 17.5 | NI | NS | HD | NG | NS | NN | HD | N* | NN | NN | NI | NG | HD | NG | HD | HD | HD | NG | | | | | | | | | | | | | |
| *tal*$_{Xoo\ K74}$-11 (KP711421) | K74 | 19.5 | NI | NG | NN | NG | NK | NG | NI | NN | NI | NN | NI | NN | NS | NG | NS | NN | NI | N* | NS | NG | | | | | | | | | | | |
| *talC2a* (ACD58246.1) | PXO99$^{A}$ | 14.5 | NI | NG | NN | NG | NK | NG | NI | NN | NI | NN | NI | HD | N* | NS | N* | | | | | | | | | | | | | | | | |
| *XOO_2865* (YP_451894.1) | MAFF311018 | 19.5 | NI | NG | NN | NG | NK | NG | NI | NN | NI | NN | NI | NN | NS | NG | NS | NN | NI | N* | NS | NG | | | | | | | | | | | |
| *tal*$_{Xoo\ K74}$-9 (KP711419) | K74 | 19.5 | | NI | NI | HG | NI | NI | NS | HD | NN | HD | NS | NG | SS | HD | NI | NI | NN | NI | NN | NI | NG | | | | | | | | | | |
| *talC6b* (ACD58952.1) | PXO99$^{A}$ | 20.5 | NI | HG | NI | HG | NI | NI | NI | HD | NN | HD | NS | NG | SS | HD | NI | NI | NN | NI | NN | NI | NG | | | | | | | | | | |
| *XOO_2127* (YP_451156.1) | MAFF311018 | 20.5 | NI | N* | NI | HG | NI | NI | NS | HD | NN | HD | NS | NG | SS | HD | NI | NI | NN | NI | NN | NS | NG | | | | | | | | | | |
| *tal*$_{Xoo\ K74}$-8 (KP711418) | K74 | 19.5 | NI | HG | NI | NG | HG | HD | NS | NG | HD | NN | NG | HG | NG | HD | HG | HD | HD | NI | NN | NG | | | | | | | | | | | |
| *talC7b* (ACD59227.1) | PXO99$^{A}$ | 19.5 | NI | HG | NS | HG | HG | HD | NS | NG | HD | NN | NG | HG | NG | HD | HG | HD | HD | NI | NN | NG | | | | | | | | | | | |
| *XOO_2160* (YP_451189.1) | MAFF311018 | 30.5 | NI | HG | NI | NG | HG | HD | NS | NG | HD | NN | NG | HG | NG | HD | HG | HD | HD | NI | NS | HG | HD | NI | N* | NS | NI | NI | HD | HD | N* | NS | N* |
| *tal*$_{Xoo\ K74}$-7 (KP711417) | K74 | 21.5 | NI | HG | NI | NN | NN | NI | NN | HD | NS | HD | NS | NS | NS | HD | NN | HD | NG | HD | HD | HD | NG | NG | | | | | | | | | |
| *XOO_4014* (YP_453043.1) | MAFF311018 | 21.5 | NI | HG | NI | NN | NN | NI | NN | HD | NI | HD | NS | NS | NS | HD | NN | HD | NG | HD | HD | HD | NG | NG | | | | | | | | | |
| *tal*$_{Xoo\ K74}$-2 (KP711412) | K74 | 27.5 | NI | HG | NI | HG | NI | NI | NI | HD | NN | HD | HD | HD | NG | HD | N* | NI | HD | HD | NN | NI | NN | NN | N* | NN | HD | N* | NS | N* | | | |
| *avrXa7* (YP_451897.1) | MAFF311018 | 25.5 | | | NI | HG | NI | NI | NS | HD | NN | HD | HD | HD | NS | N* | N* | HD | HD | NS | NS | NN | NN | NI | NG | NN | NI | N* | NS | N* | | | |
| *tal*$_{Xoo\ K74}$-6 (KP711416) | K74 | 22.5 | NI | NN | NI | NN | NN | NN | NN | NN | HD | NI | NS | HG | HD | NI | N* | NS | NI | NI | HD | HD | N* | NS | N* | | | | | | | | |
| *pthXo6* (ACD58920.2 ) | PXO99$^{A}$ | 22.5 | NI | H* | NI | NN | NN | NN | NN | NN | HD | NI | HD | HG | HD | NI | N* | NS | NI | NI | HG | HD | NS | NS | NG | | | | | | | | |
| *tal*$_{Xoo\ K74}$-3 (KP711413) | K74 | 26.5 | NI | HG | NI | NI | HG | HD | NN | HD | HD | HD | NI | NI | NN | NI | HD | HD | HD | HG | NN | NN | HD | NS | NN | HD | NG | NS | N* | | | | |
| *XOO_2866* (YP_451895.1) | MAFF311018 | 26.5 | NI | HG | NI | NI | HG | HD | NN | HD | HD | HD | NI | NI | NN | NI | HD | HD | HD | HG | NN | NN | HD | NS | NN | HD | NG | NS | N* | | | | |

* represents one missing amino acid in the RVDs.

RVDs of Tal$_{Xoo\ K74}$, which are different from the ones of either or both of PXO99$^{A}$ and MAFF311018 are labled in red.

Supplementary Table 8. List of the top 10 predicted targets for each of the 18 Tal$_{XooK74}$ effectors obtained by Talgetter 1.0.

| Tal$_{XooK74}$ | Target ID | Annotation | Distance to translation start site (bp) | Target sequences | Score | p-value | E-value |
|---|---|---|---|---|---|---|---|
| 1 | LOC_Os06g18040.1 | hypothetical protein | 524 | TCCTTTTCCCCTCCCCGC | -9,18539 | 2,15E-08 | 1,4 |
| | LOC_Os12g25220.1 | retrotransposon protein, putative, Ty3-gypsy subclass, expressed | 335 | TACTTTTCCCCAACCCCT | -9,40081 | 2,31E-08 | 1,5 |
| | LOC_Os04g26750.1 | retrotransposon protein, putative, unclassified, expressed | 518 | TCCTTTTCCCCTCCCCCT | -9,54559 | 2,46E-08 | 1,6 |
| | LOC_Os10g09430.1 | retrotransposon protein, putative, Ty3-gypsy subclass, expressed | 793 | TTCTTTTCCCCCCCCCCT | -9,56477 | 2,46E-08 | 1,6 |
| | LOC_Os09g10610.1 | expressed protein | 800 | AACTTTTCCCCTCCCCGT | -9,75614 | 2,77E-08 | 1,8 |
| | LOC_Os04g50120.1 | zinc-binding protein, putative, expressed | 816 | TCCTTTTCCCCCCCCCCC | -9,76652 | 2,77E-08 | 1,8 |
| | LOC_Os11g19220.1 | mitosis protein dim1, putative, expressed | 90 | TTCCTTTCCCCGCCCCGG | -9,79402 | 2,92E-08 | 1,9 |
| | LOC_Os06g40520.1 | TNP1, putative, expressed | 966 | TACTTTTCCACGCCACAT | -10,0732 | 4,00E-08 | 2,6 |
| | LOC_Os09g06910.1 | retrotransposon protein, putative, unclassified, expressed | 966 | TACTTTTCCACGCCACAT | -10,0732 | 4,00E-08 | 2,6 |
| | LOC_Os11g41720.1 | TNP1, putative, expressed | 966 | TACTTTTCCACGCCACAT | -10,0732 | 4,00E-08 | 2,6 |
| 2 | LOC_Os11g31190.1 | nodulin MtN3 family protein, putative, expressed | 232 | TATATAAACCCCCTCCAACCAGGTGCTAA | -16,1988 | 0 | 0 |
| | LOC_Os09g13460.1 | expressed protein | 220 | TATATAATCCCCCTCCCCCGAGGTCATTC | -21,8868 | 0 | 0 |
| | LOC_Os02g57120.1 | HEAT repeat family protein, putative, expressed | 97 | TATATAAACCTCCTCCCCAAAAGCCCCAC | -24,1281 | 9,90E-09 | 0,8 |
| | LOC_Os01g64970.1 | CAMK_CAMK_like.11 - CAMK includes calcium/calmodulin depedent protein kinases, expressed | 275 | TATATAAACCCACCCCCCCGACCCGCCTC | -24,2429 | 1,24E-08 | 1 |
| | LOC_Os03g04040.1 | expressed protein | 114 | TATAAAAACGCCCGCGATCCACTTGCCAC | -24,3024 | 1,24E-08 | 1 |
| | LOC_Os04g36800.1 | 3-oxoacyl-[acyl-carrier-protein] synthase | 26 | TATATAACCCACCTCCACCTCGCTTCCCC | -24,4583 | 1,36E-08 | 1,1 |
| | LOC_Os04g36800.2 | 3-oxoacyl-[acyl-carrier-protein] synthas | 26 | TATATAACCCACCTCCACCTCGCTTCCCC | -24,4583 | 1,36E-08 | 1,1 |
| | LOC_Os02g26430.1 | WRKY51-superfamily of TFs having WRKY and zinc finger domain | 117 | TACAAACCCGCCCTCTCCCGCCGTTCTAC | -25,3523 | 2,85E-08 | 2,3 |
| | LOC_Os12g39360.1 | Eukaryotic aspartyl protease family protein | 609 | TATATCCATCCCCTAGACCGAGCTACTAC | -25,3944 | 2,97E-08 | 2,4 |
| | LOC_Os01g74370.1 | expressed protein | 1086 | TATAAAATCGCCCTCCCCCAGGGTGGCAA | -25,6476 | 3,47E-08 | 2,8 |
| 3 | LOC_Os08g30910.1 | YDG/SRA domain containing protein, expressed | 256 | TCTCCTCGCCCAAAACCCCCCCAACTCC | -22,125 | 3,11E-09 | 0,2 |
| | LOC_Os01g61080.1 | WRKY24, expressed | 323 | TCTATCCGCCAAAAACCCTACCCGCTCC | -22,77 | 1,24E-08 | 0,8 |
| | LOC_Os03g54063.1 | expressed protein | 752 | CCCAATCCCCCGAAACCCTAGCCACTAC | -23,2692 | 1,55E-08 | 1 |
| | LOC_Os03g54063.2 | expressed protein | 752 | CCCAATCCCCCGAAACCCTAGCCACTAC | -23,2692 | 1,55E-08 | 1 |
| | LOC_Os03g54063.3 | expressed protein | 752 | CCCAATCCCCCGAAACCCTAGCCACTAC | -23,2692 | 1,55E-08 | 1 |
| | LOC_Os03g06190.1 | 3-5 exonuclease family protein, putative, expressed | 292 | CCTCCTCGCCCAAAACCCTAGCCGCACC | -23,4943 | 1,71E-08 | 1,1 |
| | LOC_Os07g23900.1 | OsFBX234 - F-box domain containing protein, expressed | 36 | TATCCCCACCCAAAACCCTCGCGTCCAC | -24,0469 | 2,49E-08 | 1,6 |
| | LOC_Os05g51650.1 | LSM domain containing protein, expressed | 685 | AAGACTAGCCAAAAACCCTAGCAGCTAG | -24,0759 | 2,49E-08 | 1,6 |
| | LOC_Os10g40790.1 | expressed protein | 813 | TCTAACTGCCCCAAACCCTAGCCGCCGC | -24,2162 | 3,57E-08 | 2,3 |
| | LOC_Os04g55800.1 | sulfate transporter, putative, expressed | 83 | TATACACACCCCAAACCCTCCTAGCTCT | -24,4691 | 4,51E-08 | 2,9 |
| 4 | LOC_Os12g38380.1 | tetratricopeptide repeat containing protein, putative, expressed | 90 | TCCGAATCCGCTCTCCCAATCCTCTTCT | -21,8771 | 3,11E-09 | 0,2 |
| | LOC_Os10g40580.1 | expressed protein | 273 | TCCGCATCCTCTTTCCGATTCATCTCTC | -22,9888 | 1,40E-08 | 0,9 |
| | LOC_Os12g18110.1 | PQ loop repeat domain containing protein, expressed | 263 | CCCGGTTCGTCCCTCCCAAGCCCCAACC | -23,176 | 1,71E-08 | 1,1 |
| | LOC_Os12g18110.2 | PQ loop repeat domain containing protein, expressed | 263 | CCCGGTTCGTCCCTCCCAAGCCCCAACC | -23,176 | 1,71E-08 | 1,1 |
| | LOC_Os12g18110.3 | PQ loop repeat domain containing protein, expressed | 263 | CCCGGTTCGTCCCTCCCAAGCCCCAACC | -23,176 | 1,71E-08 | 1,1 |
| | LOC_Os02g38860.1 | expressed protein | 72 | CCCCTATCTTCTCTCCCCACCCACTTTC | -23,4256 | 2,02E-08 | 1,3 |
| | LOC_Os03g44760.1 | SWI1, putative, expressed | 297 | TCCGCATCCTCTTTCTCATCCATCTCTC | -23,4405 | 2,02E-08 | 1,3 |
| | LOC_Os03g44760.2 | SWI1, putative, expressed | 297 | TCCGCATCCTCTTTCTCATCCATCTCTC | -23,4405 | 2,02E-08 | 1,3 |
| | LOC_Os06g21550.1 | thioredoxin domain-containing protein 17, putative, expressed | 761 | TCCCGACCTCCTCACCCAAGCCACAGCT | -23,6259 | 2,64E-08 | 1,7 |
| | LOC_Os05g08030.1 | retrotransposon protein, putative, unclassified, expressed | 303 | CCCGGCCCATCTCTCCTCCCCCCCTCTC | -24,0318 | 4,35E-08 | 2,8 |
| 5 | LOC_Os07g09384.1 | expressed protein | 76 | AGCCTCGCACGCGCGCGAGGCCG | -16,0279 | 4,64E-09 | 0,3 |
| | LOC_Os06g37364.1 | cytochrome P450, putative, expressed | 83 | CGCATCGCAACCGCGCGACGCGA | -17,3603 | 1,70E-08 | 1,1 |
| | LOC_Os06g37364.2 | cytochrome P450, putative, expressed | 83 | CGCATCGCAACCGCGCGACGCGA | -17,3603 | 1,70E-08 | 1,1 |
| | LOC_Os08g37090.1 | expressed protein | 662 | TGCATCGCATACGCAAAGGCCGG | -17,3734 | 1,86E-08 | 1,2 |
| | LOC_Os09g27180.1 | retrotransposon protein, putative, Ty1-copia subclass, expressed | 341 | TGCCACACACGCGCGCGCGCCGG | -17,4093 | 2,01E-08 | 1,3 |
| | LOC_Os07g20310.1 | expressed protein | 566 | TCCCTCCTACCCGAGCGGCGCGG | -17,4696 | 2,01E-08 | 1,3 |
| | LOC_Os03g46740.1 | ABC transporter, ATP-binding protein, putative, expressed | 26 | TTCCTCGCCCACGCGCGTGGCGC | -17,5375 | 2,16E-08 | 1,4 |
| | LOC_Os03g46740.2 | ABC transporter, ATP-binding protein, putative, expressed | 66 | TTCCTCGCCCACGCGCGTGGCGC | -17,5375 | 2,16E-08 | 1,4 |
| | LOC_Os03g16550.1 | expressed protein | 119 | CGCATCGCACGAGCGCGGCGAGG | -17,6094 | 2,63E-08 | 1,7 |
| | LOC_Os11g03570.1 | protein of unknown function, DUF618 domain containing protein, expressed | 321 | TGCAGCGAACACACGCAGCACGG | -17,8234 | 3,56E-08 | 2,3 |
| 6 | LOC_Os07g05400.1 | ferredoxin--NADP reductase, chloroplast precursor, putative, expressed | 954 | TAGAAGGGACAATCGCTAACCTCC | -18,3096 | 4,95E-08 | 3,2 |
| | LOC_Os12g17840.1 | ubiquitin family protein, expressed | 876 | TAGAAGAAGCAAGCAAAAACCAAA | -18,4675 | 5,88E-08 | 3,8 |
| | LOC_Os03g59390.1 | CAMK_CAMK_like.24 - CAMK includes calcium/calmodulin depedent protein kinases, expressed | 356 | TAGAAGAAGCAAACAAACACCAAC | -18,8273 | 7,74E-08 | 5 |
| | LOC_Os12g28750.1 | TPD1, putative, expressed | 894 | CAGAGAAGGCCACCAAAAACCCAA | -19,254 | 1,08E-07 | 7 |
| | LOC_Os02g43594.1 | MSP domain containing protein, putative, expressed | 217 | AAGAAAAGGCAAACCCAAACCCAT | -19,3393 | 1,22E-07 | 7,9 |
| | LOC_Os02g43594.3 | MSP domain containing protein, putative, expressed | 217 | AAGAAAAGGCAAACCCAAACCCAT | -19,3393 | 1,22E-07 | 7,9 |
| | LOC_Os10g19331.1 | expressed protein | 694 | TAGAAAGGGCAAAAAAAAAACTAT | -19,4818 | 1,36E-07 | 8,8 |
| | LOC_Os10g19331.2 | expressed protein | 694 | TAGAAAGGGCAAAAAAAAAACTAT | -19,4818 | 1,36E-07 | 8,8 |
| | LOC_Os04g58320.1 | zinc finger, RING-type, putative, expressed | 358 | TCGAAGGGGTAACCACACACCTAC | -19,522 | 1,39E-07 | 9 |
| | LOC_Os04g58320.2 | zinc finger, RING-type, putative, expressed | 358 | TCGAAGGGGTAACCACACACCTAC | -19,522 | 1,39E-07 | 9 |
| 7 | LOC_Os12g28330.1 | retrotransposon protein, putative, unclassified, expressed | 714 | TATAAAACCCCAACCCCTCCCCT | -16,6017 | 2,16E-08 | 1,4 |
| | LOC_Os07g07370.1 | zinc finger A20 domain-containing stress-associated protein 18, putative, expressed | 441 | TATAAAAGCATAAACTCTCCCTC | -17,1114 | 4,64E-08 | 3 |
| | LOC_Os11g17350.1 | expressed protein | 502 | TAACGGAGCAACACCGCTCCATT | -17,1153 | 4,64E-08 | 3 |
| | LOC_Os02g22700.1 | retrotransposon, putative, centromere-specific, expressed | 235 | GATCTGACCACACACGCTCCCTT | -17,5411 | 8,04E-08 | 5,2 |
| | LOC_Os03g16920.1 | DnaK family protein, putative, expressed | 148 | TATATAAGCAAATCCCCTCCCTT | -17,6083 | 8,81E-08 | 5,7 |
| | LOC_Os09g10770.1 | OsTOP6B - Topoisomerase 6 subunit B, expressed | 664 | CATAAAAACACACACGCTCACTC | -17,8782 | 1,04E-07 | 6,7 |
| | LOC_Os04g41780.1 | expressed protein | 963 | TATACACGCACACACACTCACCT | -18,307 | 1,64E-07 | 10,6 |
| | LOC_Os05g25270.1 | expressed protein | 42 | TATAAGAACCCATCCCTTCCCTT | -18,355 | 1,73E-07 | 11,2 |
| | LOC_Os02g42390.1 | expressed protein | 945 | TATACCAACGCAACCCCTCCCTA | -18,3688 | 1,75E-07 | 11,3 |
| | LOC_Os03g47570.1 | retrotransposon protein, putative, unclassified, expressed | 975 | TCCAGGAGCACAAGCACCTCCTT | -18,4814 | 1,89E-07 | 12,2 |
| 8 | LOC_Os01g40290.1 | expressed protein | 81 | TATATACCTCGTTTCTCCAGG | -11,4833 | 0 | 0 |
| | LOC_Os09g30250.1 | OsSub58 - Putative Subtilisin homologue, expressed | 372 | TAAATCCATCATATCTCCAGT | -11,9288 | 0 | 0 |
| | LOC_Os12g30590.1 | disease resistance protein RGA2, putative, expressed | 711 | TATATTCATCATTTTACCAGT | -12,1436 | 1,54E-09 | 0,1 |
| | LOC_Os02g50350.1 | dihydroorotate dihydrogenase protein, putative, expressed | 851 | TCTATGCATTGTTTCTCCAGT | -13,0244 | 1,08E-08 | 0,7 |
| | LOC_Os02g50350.2 | dihydroorotate dihydrogenase protein, putative, expressed | 851 | TCTATGCATTGTTTCTCCAGT | -13,0244 | 1,08E-08 | 0,7 |
| | LOC_Os02g57620.1 | Citrate transporter protein, putative, expressed | 95 | TAGATCCATACTTTCTCCAGT | -13,1273 | 1,39E-08 | 0,9 |
| | LOC_Os03g13614.1 | transcription factor, putative, expressed | 307 | TATATTCATCGTTTCACCTGA | -13,1354 | 1,39E-08 | 0,9 |
| | LOC_Os03g13614.2 | transcription factor, putative, expressed | 307 | TATATTCATCGTTTCACCTGA | -13,1354 | 1,39E-08 | 0,9 |
| | LOC_Os03g13614.3 | transcription factor, putative, expressed | 307 | TATATTCATCGTTTCACCTGA | -13,1354 | 1,39E-08 | 0,9 |
| | LOC_Os07g08860.1 | S-domain receptor-like protein kinase, putative, expressed | 30 | TATACTCCTCCCTTCTCCAAT | -13,9077 | 3,55E-08 | 2,3 |
| 9 | LOC_Os06g29530.1 | retrotransposon protein, putative, unclassified | 877 | TAATAGACGCATCCACAAGAT | -13,4391 | 2,01E-08 | 1,3 |
| | LOC_Os08g06900.1 | retrotransposon protein, putative, unclassified | 479 | TAATAGACGCATCCACAAGAT | -13,4391 | 2,01E-08 | 1,3 |
| | LOC_Os11g09520.1 | retrotransposon protein, putative, unclassified, expressed | 477 | TAATAGACGCATCCACAAGAT | -13,4391 | 2,01E-08 | 1,3 |
| | LOC_Os12g41010.1 | retrotransposon protein, putative, unclassified, expressed | 288 | TAATTAACGCACGCACGAGAT | -13,9812 | 4,78E-08 | 3,1 |
| | LOC_Os04g24520.1 | ribosomal protein L51, putative, expressed | 965 | TAACAAACATATACAAAAGAT | -14,0597 | 5,71E-08 | 3,7 |
| | LOC_Os05g04170.1 | AMP-binding enzyme, putative, expressed | 373 | TACTGACCGCATTCAAGAAAT | -14,3532 | 1,00E-07 | 6,5 |
| | LOC_Os05g04170.2 | AMP-binding enzyme, putative, expressed | 373 | TACTGACCGCATTCAAGAAAT | -14,3532 | 1,00E-07 | 6,5 |
| | LOC_Os05g04170.3 | AMP-binding enzyme, putative, expressed | 373 | TACTGACCGCATTCAAGAAAT | -14,3532 | 1,00E-07 | 6,5 |
| | LOC_Os12g18920.1 | expressed protein | 104 | CAACAAACGCACACCAGAGAT | -14,4944 | 1,03E-07 | 6,7 |
| | LOC_Os09g16590.1 | cysteine-rich receptor-like protein kinase 21 precursor, putative, expressed | 621 | TAATAAACACCAACACAAGAT | -14,8868 | 1,56E-07 | 10,1 |
| 10 | LOC_Os08g07760.1 | BRASSINOSTEROID INSENSITIVE 1-associated receptor kinase 1 precursor, putative, expressed | 255 | TATAAAGCGAGGCGACGAACT | -12,4998 | 3,09E-09 | 0,2 |
| | LOC_Os01g68740.1 | keratin, type I cytoskeletal 9, putative, expressed | 72 | TATAAAACAACGCAACGATCG | -14,356 | 4,47E-08 | 2,9 |
| | LOC_Os02g03190.1 | inhibitor I family protein, putative, expressed | 63 | TATAAAGCACACCTACGACCT | -13,6675 | 1,70E-08 | 1,1 |
| | LOC_Os03g26791.1 | expressed protein | 876 | TTTAAAACAGCGACACGATCT | -15,2696 | 1,14E-07 | 7,4 |
| | LOC_Os01g61570.1 | GDSL-like lipase/acylhydrolase, putative, expressed | 53 | TATAAACACGAGCGACCAACT | -14,5331 | 4,78E-08 | 3,1 |
| | LOC_Os11g39060.1 | expressed protein | 126 | TATCAATCAAAGAGACAATCT | -15,7274 | 2,22E-07 | 14,4 |
| | LOC_Os04g44540.1 | mitochondrial carrier protein, putative, expressed | 574 | TCCAAAGCAACACAACAATCT | -15,8382 | 2,42E-07 | 15,7 |
| | LOC_Os04g44540.2 | mitochondrial carrier protein, putative, expressed | 574 | TCCAAAGCAACACAACAATCT | -15,8382 | 2,42E-07 | 15,7 |
| | Os10g38050.1 | HOTHEAD precursor, putative, expressed | 139 | TACAAAGCTGCTCGAAGATCT | -15,7571 | 1,80E-07 | 14,6 |
| | Os04g20960.1 | expressed protein | 17886 | TATATAACAGAACAACAATCC | -15,9955 | 2,52E-07 | 20,5 |
| 11 | LOC_Os01g47650.1 | vacuolar protein sorting-associated protein 16, putative, expressed | 519 | TATGTCTAGAGAAATAGAAGT | -13,307 | 1,85E-08 | 1,2 |
| | LOC_Os12g18680.1 | retrotransposon protein, putative, unclassified | 311 | TATGTTTAGAGAGAAATACAT | -13,7865 | 2,78E-08 | 1,8 |
| | LOC_Os10g28810.1 | MBTB37 - Bric-a-Brac, Tramtrack, Broad Complex BTB domain with Meprin and TRAF Homology MATH domain, expressed | 90 | TATATATAGAGAGATAGCCAC | -14,1371 | 4,32E-08 | 2,8 |
| | LOC_Os04g30800.1 | ER lumen protein retaining receptor, putative, expressed | 747 | TATGTGCAGAAAGATCGCTAT | -14,2545 | 5,09E-08 | 3,3 |
| | LOC_Os04g30800.2 | ER lumen protein retaining receptor, putative, expressed | 747 | TATGTGCAGAAAGATCGCTAT | -14,2545 | 5,09E-08 | 3,3 |
| | LOC_Os02g18990.1 | GDSL-like lipase/acylhydrolase, putative, expressed | 326 | TATATATAGAGAGAGAGAGAT | -14,3387 | 5,56E-08 | 3,6 |
| | LOC_Os11g01140.1 | AGC_PVPK_like_kin82y.18 - ACG kinases include homologs to PKA, PKG and PKC, expressed | 249 | TATATATAGAGAGATAGAGAG | -14,3593 | 5,71E-08 | 3,7 |
| | LOC_Os11g01140.2 | AGC_PVPK_like_kin82y.18 - ACG kinases include homologs to PKA, PKG and PKC, expressed | 249 | TATATATAGAGAGATAGAGAG | -14,3593 | 5,71E-08 | 3,7 |
| | LOC_Os11g01140.3 | AGC_PVPK_like_kin82y.18 - ACG kinases include homologs to PKA, PKG and PKC, expressed | 249 | TATATATAGAGAGATAGAGAG | -14,3593 | 5,71E-08 | 3,7 |
| | LOC_Os12g01140.1 | AGC_PVPK_like_kin82y.2 - ACG kinases include homologs to PKA, PKG and PKC, expressed | 239 | TATATATAGAGAGATAGAGAG | -14,3593 | 5,71E-08 | 3,7 |
| 12 | LOC_Os07g06970.1 | HEN1, putative, expressed | 185 | TCCCTTCCCTAAACCCCACTT | -10,4036 | 0 | 0 |
| | LOC_Os01g20810.1 | expressed protein | 967 | TCCCTCCCCCCACCCCCACTT | -11,4434 | 0 | 0 |
| | LOC_Os02g21700.1 | STE_MEKK_ste11_MAP3K.8 - STE kinases include homologs to sterile 7, sterile 11 and sterile 20 from yeast, expressed | 236 | TCCCTTGCCAAAGCATCACCT | -12,2682 | 1,54E-09 | 0,1 |
| | LOC_Os04g11540.1 | retrotransposon protein, putative, unclassified | 971 | TCCCATGCCCAAGCCACTCTT | -13,2933 | 1,39E-08 | 0,9 |
| | LOC_Os12g17910.1 | T-complex protein, putative, expressed | 2 | TCCCTCCCCCAAGCTTCCCTC | -13,6732 | 2,78E-08 | 1,8 |
| | LOC_Os02g32440.1 | expressed protein | 298 | TCCCTTCCCCAAACCCAACCT | -14,1613 | 3,39E-08 | 2,2 |
| | LOC_Os07g40800.1 | PPR repeat domain containing protein, putative, expressed | 11 | ACCCTAGCCCAAACCCCACCT | -14,1955 | 3,39E-08 | 2,2 |
| | LOC_Os01g10000.1 | expressed protein | 35 | ACCCGCGCCCAACCCACACTT | -14,5672 | 5,25E-08 | 3,4 |
| | LOC_Os08g29370.1 | peptidyl-prolyl cis-trans isomerase, chloroplast precursor, putative, expressed | 78 | CACCTCACCAAAACCCCACTT | -14,5968 | 5,40E-08 | 3,5 |
| | LOC_Os08g29370.2 | peptidyl-prolyl cis-trans isomerase, chloroplast precursor, putative, expressed | 78 | CACCTCACCAAAACCCCACTT | -14,5968 | 5,40E-08 | 3,5 |
| 13 | LOC_Os06g29790.1 | phosphate transporter 1, putative, expressed | 332 | TATAAGTGACAGCCCTCCCCT | -11,2732 | 1,54E-09 | 0,1 |
| | LOC_Os03g40610.1 | cytochrome P450, putative, expressed | 137 | TATAAATAACAGGTCTCCCCT | -15,0218 | 6,17E-08 | 4 |
| | LOC_Os04g48530.1 | C4-dicarboxylate transporter/malic acid transport protein domain containing protein, expressed | 214 | CACACGTGAAAGCTCCCACCT | -15,2481 | 7,72E-08 | 5 |
| | LOC_Os10g39740.1 | glutathione S-transferase, putative, expressed | 66 | CACAACTGTCAAATCTCACCT | -15,4034 | 9,41E-08 | 6,1 |
| | LOC_Os08g07070.1 | expressed protein | 42 | TTCAATTGACAGACCTCAACT | -15,4214 | 9,88E-08 | 6,4 |
| | LOC_Os04g55120.1 | jp18, putative, expressed | 214 | CAAAAGTCACAAGCCTCACCT | -15,5044 | 1,13E-07 | 7,3 |
| | LOC_Os01g45470.1 | expressed protein | 541 | TACAACTGGCACAGCCCACCT | -15,636 | 1,31E-07 | 8,5 |
| | LOC_Os10g28254.1 | expressed protein | 268 | CACAGGTGAGATACCTCACCT | -15,6444 | 1,36E-07 | 8,8 |
| | LOC_Os12g04390.1 | hypothetical protein | 957 | CACAGGTGAGATACCTCACCT | -15,6444 | 1,36E-07 | 8,8 |
| | LOC_Os03g03190.1 | expressed protein | 524 | TCCACATGGCAACCCTCACCT | -15,8513 | 1,73E-07 | 11,2 |
| 14 | LOC_Os08g03550.1 | expressed protein | 81 | TAGCTCGCCGGAGCACCGC | -12,6372 | 5,24E-08 | 3,4 |
| | LOC_Os03g02690.1 | dihydrodipicolinate reductase, putative, expressed | 31 | TAACTACCTGAACCTCCCT | -12,8325 | 6,78E-08 | 4,4 |
| | LOC_Os03g02690.2 | dihydrodipicolinate reductase, putative, expressed | 31 | TAACTACCTGAACCTCCCT | -12,8325 | 6,78E-08 | 4,4 |
| | LOC_Os03g02690.3 | dihydrodipicolinate reductase, putative, expressed | 31 | TAACTACCTGAACCTCCCT | -12,8325 | 6,78E-08 | 4,4 |
| | LOC_Os10g40450.1 | expressed protein | 585 | TAACTAGCTAGAGCTACGA | -13,0464 | 9,24E-08 | 6 |
| | LOC_Os06g03530.1 | pentatricopeptide, putative, expressed | 375 | TAGATCGCCGGAGCTCCGC | -13,0739 | 9,55E-08 | 6,2 |
| | LOC_Os01g28930.1 | expressed protein | 514 | AAACCCGCCAGAGCTCCGT | -13,2827 | 1,37E-07 | 8,9 |
| | LOC_Os11g05520.1 | bifunctional monodehydroascorbate reductase and carbonic anhydrasenectarin-3 precursor, putative, expressed | 746 | TAACTAGCTAGACCTACCT | -13,5236 | 1,99E-07 | 12,9 |
| | LOC_Os10g18170.1 | RALFL16 - Rapid ALkalinization Factor RALF family protein precursor, putative, expressed | 918 | TAACTAGATGAAACTCCGC | -13,5858 | 2,09E-07 | 13,6 |
| | LOC_Os09g23595.1 | expressed protein | 189 | TAAGTCGCCGGAGCTCCGC | -13,6157 | 2,16E-07 | 14 |
| 15 | LOC_Os10g28990.1 | MBTB42 - Bric-a-Brac, Tramtrack, Broad Complex BTB domain with Meprin and TRAF Homology MATH domain, expressed | 704 | TCCTTTTCCACGCGCACGC | -10,9153 | 2,46E-08 | 1,6 |
| | LOC_Os12g19890.1 | retrotransposon protein, putative, LINE subclass, expressed | 479 | TCCTTTTCCCCCCTCTCCC | -11,5628 | 4,47E-08 | 2,9 |
| | LOC_Os03g14260.1 | guanine nucleotide exchange family protein, putative, expressed | 46 | TCCTTCTCCCCCCCCACGC | -11,5856 | 4,47E-08 | 2,9 |
| | LOC_Os03g14260.2 | guanine nucleotide exchange family protein, putative, expressed | 46 | TCCTTCTCCCCCCCCACGC | -11,5856 | 4,47E-08 | 2,9 |
| | LOC_Os07g40130.1 | retrotransposon protein, putative, Ty3-gypsy subclass, expressed | 105 | TCCTTCTCCCCCCCCACGC | -11,5856 | 4,47E-08 | 2,9 |
| | LOC_Os02g52040.1 | phosphate-induced protein 1 conserved region domain containing protein, expressed | 417 | TGCTTTTACCCACTCCCGC | -11,7631 | 5,08E-08 | 3,3 |
| | LOC_Os06g33810.1 | zinc finger protein, putative, expressed | 94 | TCCTTTTCCACTCTCACAC | -11,8109 | 5,24E-08 | 3,4 |
| | LOC_Os01g55720.1 | expressed protein | 127 | TCCCTTTCCCCACTCACCA | -12,0771 | 7,08E-08 | 4,6 |
| | LOC_Os03g49210.1 | BRCA1 C Terminus domain containing protein, expressed | 36 | TCCTTTCCCCCGCTCCCCT | -12,1163 | 7,24E-08 | 4,7 |
| | LOC_Os06g49180.1 | plant-specific domain TIGR01589 family protein, putative, expressed | 329 | TCCTCTTCCCCACTCCCAC | -12,2925 | 8,62E-08 | 5,6 |
| 16 | LOC_Os06g32590.1 | expressed protein | 865 | TACCTAGGCAGATCCAT | -8,54097 | 1,54E-08 | 1 |
| | LOC_Os12g13660.1 | expressed protein | 607 | TAGATTGGGAGATCCAT | -8,7052 | 1,69E-08 | 1,1 |
| | LOC_Os06g29790.1 | phosphate transporter 1, putative, expressed | 291 | TAGCTAGGGGAATCCAT | -9,69029 | 5,69E-08 | 3,7 |
| | LOC_Os08g17030.1 | transposon protein, putative, CACTA, En/Spm sub-class, expressed | 752 | TAGCTAGAAAGATCCCT | -9,87467 | 6,76E-08 | 4,4 |
| | LOC_Os02g18290.1 | retrotransposon protein, putative, unclassified, expressed | 616 | AAGTTAGGGAAATCCAT | -10,0721 | 8,91E-08 | 5,8 |
| | LOC_Os01g10690.1 | POLD3 - Putative DNA polymerase delta complex subunit, expressed | 485 | TAGTTAGAGGGATCCAT | -10,3723 | 1,29E-07 | 8,4 |
| | LOC_Os06g23390.1 | IQ calmodulin-binding motif family protein, putative, expressed | 471 | TACTTTGGGAAATCCAT | -10,7596 | 2,40E-07 | 15,6 |
| | LOC_Os08g34320.1 | expressed protein | 655 | TAGATACAAAGATCCAT | -10,7812 | 2,46E-07 | 16 |
| | LOC_Os03g28270.1 | Leucine Rich Repeat family protein, expressed | 302 | TATCTGGAGAGATCCAT | -10,8337 | 2,72E-07 | 17,7 |
| | LOC_Os01g58038.1 | photosystem II reaction center protein Z, putative, expressed | 783 | TAGCTACCGAGATCAAT | -10,845 | 2,74E-07 | 17,8 |
| 17 | LOC_Os09g25200.1 | protein binding protein, putative, expressed | 205 | TAGCAAACATGCGAATC | -8,24418 | 1,23E-08 | 0,8 |
| | LOC_Os09g29130.1 | ZF-HD protein dimerisation region containing protein, expressed | 698 | TAAGAAACATGGGAAAC | -8,66334 | 2,31E-08 | 1,5 |
| | LOC_Os09g27090.1 | OsFBLD9 - F-box, LRR and FBD domain containing protein, expressed | 253 | TAGGAAACACGAAAATC | -9,01383 | 3,38E-08 | 2,2 |
| | LOC_Os05g04700.1 | OsRCI2-6 - Hydrophobic protein LTI6B, expressed | 444 | TAGGAAACAAAGAAATC | -9,26278 | 4,15E-08 | 2,7 |
| | LOC_Os09g39510.1 | Cupin domain containing protein, expressed | 381 | TAGGAAACGAAGGAATC | -9,88316 | 1,05E-07 | 6,8 |
| | LOC_Os10g24360.1 | retrotransposon, putative, centromere-specific | 728 | TAAGAAAAATGGCAATC | -10,117 | 1,38E-07 | 9 |
| | LOC_Os03g15410.1 | expressed protein | 800 | TAACAAACATGAAAATC | -10,4302 | 1,77E-07 | 11,5 |
| | LOC_Os10g29730.1 | retrotransposon protein, putative, unclassified, expressed | 158 | TAGCAAACATTGAAATC | -10,4578 | 1,89E-07 | 12,3 |
| | LOC_Os11g35310.1 | phytosulfokines precursor, putative, expressed | 831 | TAGGAAACATGAAAAGC | -10,7146 | 2,58E-07 | 16,8 |
| | LOC_Os01g51290.2 | protein kinase family protein, putative, expressed | 112 | TAGAAAACATGGCATTC | -10,7544 | 2,70E-07 | 17,6 |
| 18 | LOC_Os08g05910.1 | peptide transporter PTR2, putative, expressed | 142 | TAGATTCTCTCCCT | -3,37264 | 7,66E-09 | 0,5 |
| | LOC_Os02g55320.1 | two-component response regulator, putative, expressed | 708 | TACATTCTCTCCCT | -4,98555 | 4,60E-08 | 3 |
| | LOC_Os01g12360.1 | retrotransposon protein, putative, unclassified, expressed | 155 | TAGAATCTCTCCCT | -5,1749 | 5,36E-08 | 3,5 |
| | LOC_Os01g54660.1 | retrotransposon protein, putative, unclassified, expressed | 155 | TAGAATCTCTCCCT | -5,1749 | 5,36E-08 | 3,5 |
| | LOC_Os01g64600.1 | retrotransposon protein, putative, unclassified, expressed | 154 | TAGAATCTCTCCCT | -5,1749 | 5,36E-08 | 3,5 |
| | LOC_Os01g74680.1 | retrotransposon protein, putative, unclassified, expressed | 155 | TAGAATCTCTCCCT | -5,1749 | 5,36E-08 | 3,5 |
| | LOC_Os02g01390.1 | retrotransposon protein, putative, unclassified, expressed | 155 | TAGAATCTCTCCCT | -5,1749 | 5,36E-08 | 3,5 |
| | LOC_Os03g19150.1 | retrotransposon protein, putative, unclassified, expressed | 155 | TAGAATCTCTCCCT | -5,1749 | 5,36E-08 | 3,5 |
| | LOC_Os03g53810.1 | retrotransposon protein, putative, unclassified, expressed | 155 | TAGAATCTCTCCCT | -5,1749 | 5,36E-08 | 3,5 |
| | LOC_Os04g10260.1 | basic region leucine zipper domain containing protein, expressed | 634 | TAGAATCTCTCCCT | -5,1749 | 5,36E-08 | 3,5 |

**Supplementary Table 9. Comparison of the top 10 predicted targets of the Tal$_{XooK74}$ and their counterparts in PXO99[A]**

| Tal$_{Xoo\ K74}$ | Corresponding Tal$_{PXO99}$[A] | Tal$_{Xoo\ K74}$ Target ID | Tal$_{PXO99}$[A] Target ID | Distance to translation start site (bp) | Target sequences | Score | p-value | E-value |
|---|---|---|---|---|---|---|---|---|
| **Tal$_{Xoo\ K74}$-1** | **Tal3b** | LOC_Os06g18040.1 | LOC_11g34210.1 | 491 | TCCTTTTTCCCACTCCCCT | -7.24197 | 2.4559E-9 | 0.2 |
| | | LOC_Os12g25220.1 | LOC_11g34210.2 | 491 | TCCTTTTTCCCACTCCCCT | -7.24197 | 2.4559E-9 | 0.2 |
| | | LOC_Os04g26750.1 | LOC_02g53590.1 | 980 | TCCTTCTTCCCCCTCCCCC | -9.16473 | 8.5956E-9 | 0.7 |
| | | LOC_Os10g09430.1 | LOC_11g34220.1 | 259 | TCCCTTTTCCCACTCCCCT | -9.39842 | 9.8236E-9 | 0.8 |
| | | LOC_Os09g10610.1 | LOC_02g43850.1 | 52 | TCCCTTTTCCCTCTCCCCC | -10.0975 | 2.4559E-8 | 2.0 |
| | | LOC_Os04g50120.1 | LOC_02g43850.2 | 1598 | TCCCTTTTCCCTCTCCCCC | -10.0975 | 2.4559E-8 | 2.0 |
| | | LOC_Os11g19220.1 | LOC_02g43850.3 | 52 | TCCCTTTTCCCTCTCCCCC | -10.0975 | 2.4559E-8 | 2.0 |
| | | LOC_Os06g40520.1 | LOC_02g43850.4 | 52 | TCCCTTTTCCCTCTCCCCC | -10.0975 | 2.4559E-8 | 2.0 |
| | | LOC_Os09g06910.1 | LOC_02g43850.5 | 52 | TCCCTTTTCCCTCTCCCCC | -10.0975 | 2.4559E-8 | 2.0 |
| | | LOC_Os11g41720.1 | LOC_05g15550.1 | 936 | TCCTTTTTCCCCCTCTCCC | -10.1579 | 2.5787E-8 | 2.0 |
| **Tal$_{Xoo\ K74}$-4** | **Tal9b** | LOC_Os12g38380.1 | LOC_04g05120.1 | 2086 | TCAGGTTCGTCTTCCCCGAGCCGCCGTC | -18.5629 | 0.0 | 0.0 |
| | | LOC_Os10g40580.1 | LOC_12g16040.1 | 742 | TCAGGTTCGTCATCCCCGAGCCGCCGTC | -21.3332 | 4.9481E-9 | 0.4 |
| | | LOC_Os12g18110.1 | LOC_08g09940.1 | 81 | TCCCCTTCTTCTTCCCTCACCCCCTTTT | -21.4644 | 6.1851E-9 | 0.5 |
| | | LOC_Os12g18110.2 | LOC_06g46366.1 | 68 | TCCAGTTCTCCTCCCCTGAGCTTCTCCC | -21.5012 | 6.1851E-9 | 0.5 |
| | | LOC_Os12g18110.3 | LOC_02g49080.1 | 116 | TCCAGTTCATCCCCCCCCCCCCCCCCCT | -22.3291 | 7.4221E-9 | 0.6 |
| | | LOC_Os02g38860.1 | LOC_06g22670.1 | 289 | TCCGATTCCGCTTCTCCGACCTCCTCCT | -23.1232 | 1.4844E-8 | 1.2 |
| | | LOC_Os03g44760.1 | LOC_07g17570.1 | 111 | ACCGATTCCCCTCCCCGAAACCCCTAAT | -23.1990 | 1.6081E-8 | 1.2 |
| | | LOC_Os03g44760.2 | LOC_02g07790.1 | 618 | TCCTCTTCCTCTTCCCCTCGCGGCTGCT | -23.3110 | 1.6081E-8 | 1.2 |
| | | LOC_Os06g21550.1 | LOC_03g56220.1 | 122 | TCCGATTCATATACACCCAACTCCTCCT | -23.3850 | 1.7318E-8 | 1.4 |
| | | LOC_Os05g08030.1 | LOC_12g04000.1 | 310 | TCCACTTCATCTCCACCAACAACCCCTT | -23.4713 | 1.9792E-8 | 1.6 |
| **Tal$_{Xoo\ K74}$-16** | **AvrXa27** | LOC_Os06g32590.1 | LOC_07g43130.1 | 505 | TAGCTAGGGCGACCAATT | -10.3201 | 2.6993E-8 | 2.2 |
| | | LOC_Os12g13660.1 | LOC_09g25314.1 | 1969 | TCGCTAGAGAAACCCATT | -10.5655 | 3.5581E-8 | 2.9 |
| | | LOC_Os06g29790.1 | LOC_07g40340.1 | 913 | TAGCTAGGCAGGCCCATT | -10.6975 | 4.2943E-8 | 3.5 |
| | | LOC_Os08g17030.1 | LOC_11g48040.1 | 433 | TAGTTAGGCATACCCATT | -10.9047 | 5.6440E-8 | 4.6 |
| | | LOC_Os02g18290.1 | LOC_02g18290.1 | 615 | AAGTTAGGGAAATCCATT | -11.3706 | 9.6929E-8 | 7.9 |
| | | LOC_Os01g10690.1 | LOC_06g04830.1 | 1697 | TAGCTAGCGAGCCCCCTT | -11.6260 | 1.3496E-7 | 11. |
| | | LOC_Os06g23390.1 | LOC_04g30090.1 | 757 | TAGCTAGGGTGTCCCATT | -11.9164 | 1.8772E-7 | 15.3 |
| | | LOC_Os08g34320.1 | LOC_11g03170.1 | 828 | TAGCTAGGGTGTCCCATT | -11.9164 | 1.8772E-7 | 15.3 |
| | | LOC_Os03g28270.1 | LOC_06g32590.1 | 864 | TACCTAGGCAGATCCATC | -12.0423 | 2.1839E-7 | 17.8 |
| | | LOC_Os01g58038.1 | LOC_03g28270.1 | 301 | TATCTGGAGAGATCCATT | -12.1233 | 2.4048E-7 | 19.6 |
| **Tal$_{Xoo\ K74}$-10** | **Tal7a** | LOC_Os08g07760.1 | LOC_01g68740.1 | 74 | TATAAAACAACGCAACGAT | -10.8759 | 1.1051E-8 | 0.9 |
| | | LOC_Os01g68740.1 | LOC_07g20390.1 | 467 | TATAAAACAGAGCAACTAT | -11.1396 | 1.7191E-8 | 1.4 |
| | | LOC_Os02g03190.1 | LOC_08g29050.1 | 439 | TATAAAACAGAGCAACTAT | -11.1396 | 1.7191E-8 | 1.4 |
| | | LOC_Os03g26791.1 | LOC_11g18480.1 | 420 | TATAAAACAGAGCAACTAT | -11.1396 | 1.7191E-8 | 1.4 |
| | | LOC_Os01g61570.1 | LOC_04g24300.1 | 102 | TATAAAGCAGCAAGACCAT | -12.2024 | 6.2625E-8 | 5.1 |
| | | LOC_Os11g39060.1 | LOC_03g05480.1 | 469 | TATAAAGCAACACAACGGT | -12.4402 | 7.9817E-8 | 6.5 |
| | | LOC_Os04g44540.1 | LOC_06g21910.1 | 104 | TATAAATCGAAACGACGAT | -12.8561 | 1.3016E-7 | 10.6 |
| | | LOC_Os04g44540.2 | LOC_02g42540.1 | 480 | TACAACGCCGCACGACGAT | -12.9691 | 1.5103E-7 | 12.3 |
| | | Os10g38050.1 | LOC_08g07760.1 | 257 | TATAAAGCGAGGCGACGAA | -13.0652 | 1.7436E-7 | 14.2 |
| | | Os04g20960.1 | LOC_08g43630.1 | 585 | TATAAAGCAAAGCTAAGCT | -13.2287 | 2.0752E-7 | 16.9 |
| **Tal$_{Xoo\ K74}$-12** | **Tal9a** | LOC_Os07g06970.1 | LOC_07g06970.1 | 185 | TCCCTTCCCTAAACCCCACTT | -10.4036 | 0.0 | 0.0 |
| | | LOC_Os01g20810.1 | LOC_01g20810.1 | 967 | TCCCTCCCCCCACCCCCACTT | -11.4434 | 0.0 | 0.0 |
| | | LOC_Os02g21700.1 | LOC_02g21700.1 | 236 | TCCCTTGCCAAAGCATCACCT | -12.2682 | 2.4599E-9 | 0.2 |
| | | LOC_Os04g11540.1 | LOC_04g11540.1 | 971 | TCCCATGCCCAAGCCACTCTT | -13.2933 | 8.6097E-9 | 0.7 |
| | | LOC_Os12g17910.1 | LOC_12g17910.1 | 2 | TCCCTCCCCCAAGCTTCCCTC | -13.6732 | 1.4759E-8 | 1.2 |
| | | LOC_Os02g32440.1 | LOC_08g44860.2 | 4513 | TCCCTTCCCCATCCTCCACTT | -13.9028 | 1.9679E-8 | 1.6 |
| | | LOC_Os07g40800.1 | LOC_08g44860.3 | 4513 | TCCCTTCCCCATCCTCCACTT | -13.9028 | 1.9679E-8 | 1.6 |
| | | LOC_Os01g10000.1 | LOC_08g44860.4 | 4513 | TCCCTTCCCCATCCTCCACTT | -13.9028 | 1.9679E-8 | 1.6 |
| | | LOC_Os08g29370.1 | LOC_08g44860.5 | 4513 | TCCCTTCCCCATCCTCCACTT | -13.9028 | 1.9679E-8 | 1.6 |
| | | LOC_Os08g29370.2 | LOC_08g44860.6 | 4513 | TCCCTTCCCCATCCTCCACTT | -13.9028 | 1.9679E-8 | 1.6 |
| **Tal$_{Xoo\ K74}$-18** | **Tal9d** | LOC_Os08g05910.1 | LOC_12g33040.1 | 153 | TAGATTGCCTCCCT | -5.39910 | 3.3020E-8 | 2.7 |
| | | LOC_Os02g55320.1 | LOC_02g51119.2 | 1602 | TAGATTCTCTCCCT | -5.47852 | 4.2804E-8 | 3.5 |
| | | LOC_Os01g12360.1 | LOC_08g05910.1 | 142 | TAGATTCTCTCCCT | -5.47852 | 4.2804E-8 | 3.5 |
| | | LOC_Os01g54660.1 | LOC_08g05910.2 | 1301 | TAGATTCTCTCCCT | -5.47852 | 4.2804E-8 | 3.5 |
| | | LOC_Os01g64600.1 | LOC_05g28990.1 | 818 | TAAATTGCCTCCCT | -6.65080 | 1.9200E-7 | 15.7 |
| | | LOC_Os01g74680.1 | LOC_04g34620.1 | 1033 | TAAATCGTCTCCCT | -6.67536 | 1.9812E-7 | 16.2 |
| | | LOC_Os02g01390.1 | LOC_07g44950.1 | 767 | TAAATCGTCTCCCT | -6.67536 | 1.9812E-7 | 16.2 |
| | | LOC_Os03g19150.1 | LOC_04g05030.1 | 4391 | TTGATTGTCTCCCT | -6.73949 | 2.5193E-7 | 20.6 |
| | | LOC_Os03g53810.1 | LOC_03g27054.1 | 769 | TAGATCGCCTCCCT | -6.81164 | 2.7027E-7 | 22.1 |
| | | LOC_Os04g10260.1 | LOC_09g20830.3 | 2030 | TAGTTTGTCTCCCT | -6.85030 | 2.7639E-7 | 22.6 |
| **Tal$_{Xoo\ K74}$-13** | **Tal6a** | LOC_Os06g29790.1 | LOC_06g29790.1 | 332 | TATAAGTGACAGCCCTCCCCT | -13.3421 | 2.4599E-9 | 0.2 |
| | | LOC_Os03g40610.1 | LOC_07g39810.1 | 1010 | TAAACGTGCCACAAATCACCT | -13.9572 | 1.5989E-8 | 1.2 |
| | | LOC_Os04g48530.1 | LOC_05g39630.1 | 527 | AACAAATGACAGATATCAACT | -14.6608 | 5.0428E-8 | 4.1 |
| | | LOC_Os10g39740.1 | LOC_10g10480.1 | 519 | TCCAAGTGACCAAAACCACCT | -14.7159 | 5.4118E-8 | 4.4 |
| | | LOC_Os08g07070.1 | LOC_10g14040.1 | 1274 | TATAAGTAACACAAATCACAT | -14.7883 | 5.7808E-8 | 4.7 |
| | | LOC_Os04g55120.1 | LOC_08g30650.1 | 490 | TACAACTGCCTGACAGCACCT | -14.8448 | 6.2727E-8 | 5.1 |
| | | LOC_Os01g45470.1 | LOC_03g43730.1 | 837 | TACACGTAGCAGCCATCACAT | -14.9905 | 6.7647E-8 | 5.5 |
| | | LOC_Os10g28254.1 | LOC_11g26790.1 | 101 | TATAAATGGCGGCCACCACCT | -15.1245 | 7.9947E-8 | 6.5 |
| | | LOC_Os12g04390.1 | LOC_02g23930.1 | 294 | AACAAGTGCAAGACATCACCC | -15.2189 | 8.9787E-8 | 7.3 |
| | | LOC_Os03g03190.1 | LOC_02g23930.2 | 294 | AACAAGTGCAAGACATCACCC | -15.2189 | 8.9787E-8 | 7.3 |
| **Tal$_{Xoo\ K74}$-5** | **Tal9e** | LOC_Os07g09384.1 | LOC_02g47580.1 | 592 | CGCATCGCACGCGCGCGCGCGCGCC | -18.9350 | 1.7275E-8 | 1.4 |
| | | LOC_Os06g37364.1 | LOC_02g34210.1 | 48 | CACGTCGCACACGCGCGCGGGAGCG | -19.2501 | 2.3445E-8 | 1.9 |
| | | LOC_Os06g37364.2 | LOC_08g37090.1 | 660 | TGCATCGCATACGCAAAGGCCGGCT | -19.6051 | 2.8381E-8 | 2.3 |
| | | LOC_Os08g37090.1 | LOC_01g42700.1 | 1347 | TACATCGCACTCGGGCGGGGGGACA | -19.9199 | 3.7019E-8 | 3.0 |
| | | LOC_Os09g27180.1 | LOC_01g42700.2 | 1347 | TACATCGCACTCGGGCGGGGGGACA | -19.9199 | 3.7019E-8 | 3.0 |
| | | LOC_Os07g20310.1 | LOC_01g42700.3 | 1524 | TACATCGCACTCGGGCGGGGGGACA | -19.9199 | 3.7019E-8 | 3.0 |
| | | LOC_Os03g46740.1 | LOC_01g42700.4 | 1524 | TACATCGCACTCGGGCGGGGGGACA | -19.9199 | 3.7019E-8 | 3.0 |
| | | LOC_Os03g46740.2 | LOC_01g42700.5 | 1347 | TACATCGCACTCGGGCGGGGGGACA | -19.9199 | 3.7019E-8 | 3.0 |
| | | LOC_Os03g16550.1 | LOC_03g50980.1 | 195 | TCCGCCGCACGCGCGCGCGCGCGCT | -20.0283 | 4.1955E-8 | 3.3 |
| | | LOC_Os11g03570.1 | LOC_04g44700.1 | 1361 | TGCACCGCTCGCGCGCGCAGGGGCC | -20.0646 | 4.4423E-8 | 3.6 |
| **Tal$_{Xoo\ K74}$-17** | **Tal4** | LOC_Os09g25200.1 | LOC_09g29130.1 | 698 | TAAGAAACATGGGAAAC | -8.06897 | 6.1297E-9 | 0.5 |
| | | LOC_Os09g29130.1 | LOC_09g25200.1 | 205 | TAGCAAACATGCGAATC | -8.45466 | 8.5817E-9 | 0.7 |
| | | LOC_Os09g27090.1 | LOC_09g27090.1 | 253 | TAGGAAACACGAAAATC | -9.22431 | 3.1874E-8 | 2.6 |
| | | LOC_Os05g04700.1 | LOC_05g04700.1 | 444 | TAGGAAACAAAGAAATC | -9.47325 | 4.9038E-8 | 4.0 |
| | | LOC_Os09g39510.1 | LOC_11g35310.1 | 831 | TAGGAAACATGAAAAGC | -9.98515 | 8.4591E-8 | 6.9 |
| | | LOC_Os10g24360.1 | LOC_09g39510.1 | 381 | TAGGAAACGAAGGAATC | -10.0936 | 1.0175E-7 | 8.3 |
| | | LOC_Os03g15410.1 | LOC_10g19338.1 | 113 | TAGGAAACAGAGGAACC | -10.2817 | 1.3730E-7 | 11.2 |
| | | LOC_Os10g29730.1 | LOC_10g24360.1 | 728 | TAAGAAAAATGGCAATC | -10.3275 | 1.4343E-7 | 11.7 |
| | | LOC_Os11g35310.1 | LOC_01g07780.1 | 1045 | TTGGAAACATGGGAATA | -10.3674 | 1.5324E-7 | 12.5 |
| | | LOC_Os01g51290.2 | LOC_03g15410.1 | 800 | TAACAAACATGAAAATC | -10.6406 | 2.2925E-7 | 18.7 |
| **Tal$_{Xoo\ K74}$-14** | **Tal5a** | LOC_Os08g03550.1 | LOC_02g06670.1 | 120 | TACCTAGCTTGGCCTCT | -6.19520 | 0.0 | 0.0 |
| | | LOC_Os03g02690.1 | LOC_07g30150.1 | 1212 | TACCTAGCATGGACTCT | -9.68174 | 5.6394E-8 | 4.6 |
| | | LOC_Os03g02690.2 | LOC_10g05130.1 | 33 | CAACTAGCTTTGCCTCT | -9.70234 | 5.6394E-8 | 4.6 |
| | | LOC_Os03g02690.3 | LOC_04g43560.1 | 225 | TAGCTCGCTTGGCCCCT | -9.87014 | 7.3557E-8 | 6.0 |
| | | LOC_Os10g40450.1 | LOC_02g42020.1 | 1652 | TAGCTAGCTTAGCCCCT | -10.2029 | 1.1278E-7 | 9.2 |
| | | LOC_Os06g03530.1 | LOC_02g42020.2 | 1652 | TAGCTAGCTTAGCCCCT | -10.2029 | 1.1278E-7 | 9.2 |
| | | LOC_Os01g28930.1 | LOC_02g42020.3 | 1652 | TAGCTAGCTTAGCCCCT | -10.2029 | 1.1278E-7 | 9.2 |
| | | LOC_Os11g05520.1 | LOC_03g55530.1 | 618 | TAGCTAGCTTAGCCCCT | -10.2029 | 1.1278E-7 | 9.2 |
| | | LOC_Os10g18170.1 | LOC_08g41470.1 | 101 | TAGCTAGCTTAGCCCCT | -10.2029 | 1.1278E-7 | 9.2 |
| | | LOC_Os09g23595.1 | LOC_08g41470.2 | 1831 | TAGCTAGCTTAGCCCCT | -10.2029 | 1.1278E-7 | 9.2 |
| **Tal$_{Xoo\ K74}$-11** | **Tal2a** | LOC_Os01g47650.1 | LOC_12g09550.1 | 1642 | TATGTTTAGAGACTAT | -7.01379 | 1.2249E-8 | 1.0 |
| | | LOC_Os12g18680.1 | LOC_12g09550.2 | 1642 | TATGTTTAGAGACTAT | -7.01379 | 1.2249E-8 | 1.0 |
| | | LOC_Os10g28810.1 | LOC_03g06420.1 | 1586 | TATGTATAGAGACTAT | -7.32386 | 1.5924E-8 | 1.3 |
| | | LOC_Os04g30800.1 | LOC_03g06420.2 | 1586 | TATGTATAGAGACTAT | -7.32386 | 1.5924E-8 | 1.3 |
| | | LOC_Os04g30800.2 | LOC_03g06420.3 | 1586 | TATGTATAGAGACTAT | -7.32386 | 1.5924E-8 | 1.3 |
| | | LOC_Os02g18990.1 | LOC_03g06420.4 | 1586 | TATGTATAGAGACTAT | -7.32386 | 1.5924E-8 | 1.3 |
| | | LOC_Os11g01140.1 | LOC_03g26510.1 | 675 | TATGTATAGAAACCAT | -7.94142 | 4.7773E-8 | 3.9 |
| | | LOC_Os11g01140.2 | LOC_08g31170.1 | 727 | TATGTATAGAAACCAT | -7.94142 | 4.7773E-8 | 3.9 |
| | | LOC_Os11g01140.3 | LOC_10g01830.1 | 137 | TATGTATACAGACCAT | -8.30510 | 6.8597E-8 | 5.6 |
| | | LOC_Os12g01140.1 | LOC_09g31434.1 | 485 | TCTCTGTAGAGACCAT | -8.56567 | 9.0647E-8 | 7.4 |
| **Tal$_{Xoo\ K74}$-9** | **Tal6b** | LOC_Os06g29530.1 | LOC_11g26970.1 | 505 | TATAGAAACTCCTCCAAGAGAT | -12.7016 | 1.2309E-9 | 0.1 |
| | | LOC_Os08g06900.1 | LOC_01g50370.1 | 142 | TATATAAACGCACACAAGCGCT | -12.9519 | 3.6928E-9 | 0.3 |
| | | LOC_Os11g09520.1 | LOC_02g53360.1 | 669 | TATAAAAACGCATGCACAAAAT | -13.3206 | 8.6167E-9 | 0.7 |
| | | LOC_Os12g41010.1 | LOC_03g62290.1 | 672 | AATATAAACACATCCAATAGAT | -13.7334 | 1.6002E-8 | 1.3 |
| | | LOC_Os04g24520.1 | LOC_11g29630.1 | 347 | TATATAAACAACTACATGAGAT | -14.4827 | 4.1852E-8 | 3.4 |
| | | LOC_Os05g04170.1 | LOC_04g21170.1 | 621 | TATATAAACACATGGAAAAAAT | -15.0766 | 6.1548E-8 | 5.0 |
| | | LOC_Os05g04170.2 | LOC_06g34890.1 | 566 | TATATAAACACATGAAAAAAAA | -15.3831 | 8.6167E-8 | 7.0 |
| | | LOC_Os05g04170.3 | LOC_06g41980.1 | 565 | TATATACATGCATGCAAGAGGT | -15.5630 | 9.6015E-8 | 7.8 |
| | | LOC_Os12g18920.1 | LOC_09g09860.1 | 378 | TATATGACCGCATAAAAAAGAT | -15.6196 | 1.0463E-7 | 8.5 |
| | | LOC_Os09g16590.1 | LOC_10g10840.1 | 382 | TATATAACCGCATAAAAAGGAT | -15.7366 | 1.1201E-7 | 9.1 |
| **Tal$_{Xoo\ K74}$-8** | **Tal7b** | LOC_Os01g40290.1 | LOC_01g40290.1 | 81 | TATATACCTCGTTTCTCCAGG | -11.9823 | 2.4599E-9 | 0.2 |
| | | LOC_Os09g30250.1 | LOC_09g30250.1 | 372 | TAAATCCATCATATCTCCAGT | -12.4323 | 4.9198E-9 | 0.4 |
| | | LOC_Os12g30590.1 | LOC_12g30590.1 | 711 | TATATTCATCATTTTACCAGT | -12.6425 | 8.6097E-9 | 0.7 |
| | | LOC_Os02g50350.1 | LOC_02g50350.1 | 851 | TCTATGCATTGTTTCTCCAGT | -13.5285 | 2.5829E-8 | 2.0 |
| | | LOC_Os02g50350.2 | LOC_02g50350.2 | 851 | TCTATGCATTGTTTCTCCAGT | -13.5285 | 2.5829E-8 | 2.0 |
| | | LOC_Os02g57620.1 | LOC_03g13614.1 | 307 | TATATTCATCGTTTCACCTGA | -13.6343 | 3.0749E-8 | 2.5 |
| | | LOC_Os03g13614.1 | LOC_03g13614.2 | 307 | TATATTCATCGTTTCACCTGA | -13.6343 | 3.0749E-8 | 2.5 |
| | | LOC_Os03g13614.2 | LOC_03g13614.3 | 307 | TATATTCATCGTTTCACCTGA | -13.6343 | 3.0749E-8 | 2.5 |
| | | LOC_Os03g13614.3 | LOC_02g57620.1 | 95 | TAGATCCATACTTTCTCCAGT | -13.6349 | 3.0749E-8 | 2.5 |
| | | LOC_Os07g08860.1 | LOC_07g08860.1 | 30 | TATACTCCTCCCTTCTCCAAT | -13.7011 | 3.0749E-8 | 2.5 |
| **Tal$_{Xoo\ K74}$-6** | **PthXo6** | LOC_Os07g05400.1 | LOC_01g70320.1 | 255 | TCCAAGAAACACTCACAAATCAAT | -16.4068 | 4.9319E-9 | 0.4 |
| | | LOC_Os12g17840.1 | LOC_09g29820.1 | 112 | TATAAAAGGCCCTCACCAACCCAT | -17.4024 | 2.5892E-8 | 2.0 |
| | | LOC_Os03g59390.1 | LOC_06g40600.1 | 641 | TAAAGGCGGCATACACCAATCCAT | -17.8619 | 4.5620E-8 | 3.7 |
| | | LOC_Os12g28750.1 | LOC_02g04880.1 | 788 | TATCGGGAGCATTCATAAAACCAT | -17.8724 | 4.6853E-8 | 3.8 |
| | | LOC_Os02g43594.1 | LOC_03g08330.1 | 997 | TAGAGGAAGCACACACAACTCAAC | -18.1288 | 5.4251E-8 | 4.4 |
| | | LOC_Os02g43594.3 | LOC_10g18910.1 | 970 | TCAAGAGAGCACTAATAAAACAAT | -18.2459 | 6.1648E-8 | 5.0 |
| | | LOC_Os10g19331.1 | LOC_09g04120.1 | 626 | TAGAAGAGGCACTCAGAAATACCT | -18.2866 | 6.1648E-8 | 5.0 |
| | | LOC_Os10g19331.2 | LOC_11g14910.1 | 678 | TGTAGGGCCCACACACAAATAAAT | -18.3504 | 6.7813E-8 | 5.4 |
| | | LOC_Os04g58320.1 | LOC_01g27449.1 | 844 | TATAGGGGAAAATCATGAATAAAT | -18.3658 | 6.9046E-8 | 5.6 |
| | | LOC_Os04g58320.2 | LOC_05g02360.1 | 184 | TATCGGGAGCATTCATAAAAAAAT | -19.0445 | 1.5165E-7 | 12.3 |
