# Supplementary material for: Rapid and efficient genome-wide characterization of Xanthomonas TAL effector genes
Source: Sci Rep. 2015 Aug 14;5:13162. doi: 10.1038/srep13162 (PMC4536657; doi:10.1038/srep13162)
Supplement: Supplementary Data 1 [file srep13162-s2.doc]

**Supplementary DNA sequence data 1**. The initial DNA sequence data of the 18 unique *tal*-*Bam*HI fragments of *Xoo* strain K74 and the *tal-Bam*HI fragment of *talC*, obtained from sequencing.

**pTAL*_Bam_*_HI_-1**

> 1P1F_1

GCGGGGGAGTTGAGAGGTCCACCGTTACAGTTGGACACAGGCCAACTTGTCAAGATTGCAAAACGTGGCGGCGTGACCGC

AGTGGAGGCAGTGCATGCATCGCGCAATGCACTGACGGGTGCCCCCCTGAACCTGACCCCGGACCAAGTGGTGGCCATCG

CCAGCAATATTGGCGGCAAGCAGGCGCTGGAGACGGTGCAGCGCCTGTTGCCGGTGCTGTGCCAGGACCATGGCCTGACC

CCGGACCAGGTGGTGGCCATCGCCAACAATAACGGCGGCAAGCAGGCGCTGGAGACGGTGCAGCGGCTGTTGCCGGTGCT

GTGCCAGGACCATGGCCTGACCCCGGACCAGGTGGTGGCCATCGCCAGCAATATTGGCGGCAAGCAGGCGCTGGAGACGG

TGCAGCGGCTGTTGCCGGTGCTGTGCCAGGCCCATGGCCTGACCCCGGCCCAGGTGGTGGCCATCGCCAGCAATAACGGC

GGCAAGCAGGCGCTGGAGACGGTGCAGCGGCTGTTGCCGGTGCTGTGCCAGGCCCATGGCCTGACCCCGGACCAGGTGGT

GGCTATCGCCAGCAATAACGGCGGCAAGCAGGCGCTGGAGACGGTGCAACGGCTGTTGCCGGTGCTGTGCCAGGCCCATG

GCCTGACCCCGGACCAAGTGGTGGCCATCGCCAACAATAACGGCGGCAAGCAGGCGCTGGAGACGGTGCAGCGGCTGTTG

CCGGTGCTGTGCCAGGACCATGGCCTGAGTCCGGACCAGGTGGTGGCCATCGCCAACAATAACGGCGGCAAGCAGGCGCT

GGAGACGCTGCAGCGGCTGTTGCCGGTGCTGTGCCAGACCCATGCCCTGACCCCGGACCAGGTGGTGGCCATCGCCAACA

ATAACGGC

> 1P1R_2

ACAATGCTCTCCAGCGCCTGCTTGCCGCCATTGCTGGCGATGGCCACCACCTGGTTCGGGGTCAGGCCATGGTCCTGGCA

CAGCACCGGCAACAGCCGCTGCACCGTCTCCAGCGCCTGCTTGCCGCCACTATTGCTGGCGATGGCCACCACCTGGTTCG

GGGTCAGGCCATGGTCCTGGCACAGCACCGGCAACAGCCGCTGCACCGTCTCCAGCGCCTGCTTGCCGCCATTGCTGGCG

ATGGCCACCACCTTGTCCAGGGTCAGGCCATGGGCCTGGCACAGCACCGGCAACAGCCGCTGCACCGTCTCCAGCGCCTG

CTTGCCGCCATCGTGGCTGGCGATGGCCACGACCTGGTCCGGGGTCAGGCCATGGGCCTGGCACAGCACCGGCAACAGCC

GCTGCACCGTCTCCAGCGCCTGTTTGCCGCCATCGTGGCTGGCGATGGCCACCACCTGGTCCAGGGTCAGGCCATGGTCC

TGGCACAGCACCGGCAACAGCCGCTGCACCGTCTCCAGCGCCTGCTTGCCGCCAATATTGCTGGCGATGGCCACCACCTG

GTCCGGGGTCAGGCCATGGTCCTGGCATAGCACCGGCAACAGCCGCTGCACCGTCTCCAGCGCCTGCTTGCCGCCAATAT

TGCTGGCGATGGCCACCACTTGGTTCGGGGTCAGGCCATGGTCCTGGCACAGCACCGGCAACAGCCGCTGCACCGTCTCC

AGCGCCTGCTTGCCGCCACTATTACTGGCGATGGCCACCACTTGGTCCGGGGTCAGGCCATGAGCCTGGCACAGCACCGG

CAACAGCCGCTGCACCGTCTCCAGCGCCTGCTTGCCGCCATTGCTGGCGATGGCCACGACCTGGTCCGGGGTCAGGCCAT

GGTCCTGGCACAGCACCGGCAACAGCCGTTGCACCGT

> 1P2F

CGCGCACATCGTTGCGCTCAGCCAACACCCGGCAGCGTTAGGGACCGTTGCTGTCACGTATCAGGACATAATCAGGGCGT

TGCCAGAGGCGACACACGAAGACATCGTTGGCGTCGGCAAACAGTGGTCCGGCGCACGCGCCCTGGAGGCCTTGCTCACG

AAGGCGGGGGAGTTGAGAGGTCCACCGTTACAGTTGGACACAGGCCAACTTGTCAAGATTGCAAAACGTGGCGGCGTGAC

CGCAGTGGAGGCAGTGCATGCATCGCGCAATGCACTGACGGGTGCCCCCCTGAACCTGACCCCGGACCAAGTGGTGGCCA

TCGCCAGCAATATTGGCGGCAAGCAGGCGCTGGAGACGGTGCAGCGCCTGTTGCCGGTGCTGTGCCAGGACCATGGCCTG

ACCCCGGACCAGGTGGTGGCCATCGCCAACAATAACGGCGGCAAGCAGGCGCTGGAGACGGTGCAGCGGCTGTTGCCGGT

GCTGTGCCAGGACCATGGCCTGACCCCGGACCAGGTGGTGGCCATCGCCAGCAATATTGGCGGCAAGCAGGCGCTGGAGA

CGGTGCAGCGGCTGTTGCCGGTGCTGTGCCAGGCCCATGGCCTGACCCCGGCCCAGGTGGTGGCCATCGCCAGCAATAAC

GGCGGCAAGCAGGCGCTGGAGACGGTGCAGCGGCTGTTGCCGGTGCTGTGCCAGGCCCATGGCCTGACCCCGGACCAGGT

GGTGGCTATCGCCAGCAATAACGGCGGCAAGCAGGCGCTGGAGACGGTGCAACGGCTGTTGCCGGTGCTGTGCCAGGCCC

ATGGCCTGACCCCGGACCAAGTGGTGGCCATCGCCAACAATAACGGCGGCAAGCAGGCGCTGGAGACGGTGCAGCGGCTG

TTGCCGGTGCTGTGCCAGGACCATGG

> 1P2R

CGCCCTGATTATGTCCTGATACGTGACAGCAACGGTCCCTAACGCTGCCGGGTGTTGGCTGAGCGCAACGATGTGCGCGT

GTGTAAACCCATGGCCCACCAGTGCCTCGTGGTGCTGCGCCACTGTCGAACGCACCTTCGGTTTGATCTTCTCTTGCTGC

TGCTGACTGTAGCCGAGCGTGCGTAGATCCACCTGCGCGGCCGGCGAAGCGTCGGAGGGTTGCGCCGCACGCCGTCGCGG

GGCCGGCTTGGCGCGCGGCGGCCGCGCGGCAGTGACAGCGACAGGCACGGTGGGTGGCGGGTCATCGGCTGCACGCAGAC

CCGATTGCGCCTCATCCCATTCTGCTGGGGCAGCCGCTGTATGCGGCGTGCCGACGGCAGGCATCGAATCAAGAAGCGAT

GTATCAAGAAGCGACGGATCGAACTGACGGAGCAGATCGCTGAAGCTGCCCGCCGAGAACGCAGGCGAGGGCGCAGGGGG

AGATGGCAGCCGGGTCCGGGACATCGTCCGCCGAGCGGGCAAGCCATCCAGGGGGCCGCCAGCAGGCGGAGCCCCCCCCC

GATCTGCAGTCGGCTGAACCCTATCCGGTTGGGGTCCGGGCAGAAGCTCGCGGGCAGGACTTGGCGTGCGCGAACGAATG

GGATCC

> 1P3F

AAGGGATTGCCGCACGCGCCGGAATTGATCAGAAGAATCAATCGCCGCATTCCCGAACGCACGTCCCATCGCGTTCCCGA

CCTCGCGCACGTGGTTCGCGTGCTTGGTTTTTTCCAGAGCCACTCCCACCCAGCGCAAGCATTCGATGACGCCATGACGC

AGTTCGAGATGAGCAGGCACGGCTTGGTACAGCTCTTTCGCAGAGTGGGCGTCACCGAATTCGAAGCCCGCTACGGAACG

CTCCCCCCAGCCTCGCAGCGTTGGGACCGTATCCTCCAGGCATCAGGGATGAAAAGGGCCAAACCGTCCCCTACTTCAGC

TCAAACACCGGATCAGGCGTCTTTGCATGCATTCGCCGATTCGCTGGAGCGTGACCTTGATGCGCCTAGCCCAATGCACG

AGGGAGATCAGACAGGGGCAAGCAGCCGTAAACGGTCCCGATCGGATCGTGCTGTCACCGGCCCCTCCGCACAGCAATCT

TTCGAGGTGCGCGTTCCCGAACAGCGCGATGCGCTGCATTTGCCCCTCAGCTGGAGGGTAAAACGCCCGCGTACCAGGAT

CGGGGGCGGCCTCCCGGATCC

> 1P3R

TCGGTGACGCCCACTCTGCGAAAGAGCTGTACCAAGCCGTGCCTGCTCATCTCGAACTGCGTCATGGCGTCATCGAATGC

TTGCGCTGGGTGGGAGTGGCTCTGGAAAAAACCAAGCACGCGAACCACGTGCGCGAGGTCGGGAACGCGATGGGACGTGC

GTTCGGGAATGCGGCGATTGATTCTTCTGATCAATTCCGGCGCGTGCGGCAATCCCTTTTTCACTGCATCCAGGGCAGGA

CGTCCGCCGAGGCAGGCCAAGGCGACGAGGTGGTCGTTGGTCAACGCGGCCAACGCCGGATCAGGGCGAGATAACTGGGC

AACAATGCTCTCCAGCGCCTGCTTGCCGCCATTGCTGGCGATGGCCACCACCTGGTTCGGGGTCAGGCCATGGTCCTGGC

ACAGCACCGGCAACAGCCGCTGCACCGTCTCCAGCGCCTGCTTGCCGCCACTATTGCTGGCGATGGCCACCACCTGGTTC

GGGGTCAGGCCATGGTCCTGGCACAGCACCGGCAACAGCCGCTGCACCGTCTCCAGCGCCTGCTTGCCGCCATTGCTGGC

GATGGCCACCACCTTGTCCAGGGTCAGGCCATGGGCCTGGCACAGCACCGGCAACAGCCGCTGCACCGTCTCCAGCGCCT

GCTTGCCGCCATCGTGGCTGGCGATGGCCACGACCTGGTCCGGGGTCAGGCCATGGGCCTGGCACAGCACCGGCAACAGC

CGCTGCACCGTCTCCAGCGCCTGTTTGCCGCCATCGTGGCTGGCGATGGCCACCACCTGGTCCAGGGTCAGGCCATGGTC

CTGGCACAGCACCGGCAACAGCCGCTGCACCGTCTCCAGCGCCTGCTTGCCGCCAATATTGCTGGCGATGGCCACCACCT

GGTCCGGGGTCAGGCCATGGTCCTGGCATAGCACCGGCAACAGCCGCTGCACCG

> pCC2FOS-MscI-1

CCATCGCCAGCAATATTGGCGGCAAGCAGGCGCTGGAGACGGTGCAGCGGCTGTTGCCGGTGCTGTGCCAGGACCATGGC

CTGACCCCGGACCAGGTGGTGGCCATCGCCAGCAATAGTGGCGGCAAGCAGGCGCTGGAGACGGTGCAGCGGCTGTTGCC

GGTGCTGTGCCAGGACCATGGCCTGACCCCGGACCAGGTGGTGGCCATCGCCAGCCATGGCGGCGGCAAGCAGGCGCTGG

AGACGGTGCAGCGGCTGTTGCCGGTGCTGTGCCAGGACCATGGCCTGACCCCAGACCAGGTCGTGGCCATCGCCAGCCAC

GATGGCGGCAAGCAGGCGCTGGAGACGGTGCAGCGGCTGTTGCCGGTGCTGTGCCAGGCCCATGGCCTGACCCCGAACCA

GGTGGTGGCCATCGCCAGCAATATTGGCGGCAAGCAGGCGCTGGAGACGGTGCAACGGCTGTTGCCGGTGCTGTGCCAGG

ACCATGGCCTGACCCCGGACCAGGTCGTGGCCATCGCCAGCAATGGCGGCAAGCAGGCGCTGGAGACGGTGCAGCGGCTG

TTGCCGGTGCTGTGCCAGGCTCATGGCCTGACCCCGGACCAAGTGGTGGCCATCGCCAGTAATAGTGGCGGCAAGCAGGC

GCTGGAGACGGTGCAGCGGCTGTTGCCGGTGCTGTGCCAGGACCATGGCCTGACCCCGAACCAAGTGGTGGCCATCGCCA

GCAATATTGGCGGCAAGCAGGCGCTGGAGACGGTGCAGCGGCTGTTGCCGGTGCTATGCCAGGACCATGGCCTGACCCCG

GACCAGGTGGTGGCCATCGCCAGCAATATTGGCGGCAAGCAGGCGCTGGAGACGGTGCAGCGGCTGTTGCCGGT

>1P1F_2

ACAGGCCAACTTGTCAAGATTGCAAAACGTGGCGGCGTGACCGCAGTGGAGGCAGTGCATGCATCGCGCAATGCACTGAC

GGGTGCCCCCCTGAACCTGACCCCGGACCAAGTGGTGGCCATCGCCAGCAATATTGGCGGCAAGCAGGCGCTGGAGACGG

TGCAGCGCCTGTTGCCGGTGCTGTGCCAGGACCATGGCCTGACCCCGGACCAGGTGGTGGCCATCGCCAACAATAACGGC

GGCAAGCAGGCGCTGGAGACGGTGCAGCGGCTGTTGCCGGTGCTGTGCCAGGACCATGGCCTGACCCCGGACCAGGTGGT

GGCCATCGCCAGCAATATTGGCGGCAAGCAGGCGCTGGAGACGGTGCAGCGGCTGTTGCCGGTGCTGTGCCAGGCCCATG

GCCTGACCCCGGCCCAGGTGGTGGCCATCGCCAGCAATAACGGCGGCAAGCAGGCGCTGGAGACGGTGCAGCGGCTGTTG

CCGGTGCTGTGCCAGGCCCATGGCCTGACCCCGGACCAGGTGGTGGCTATCGCCAGCAATAACGGCGGCAAGCAGGCGCT

GGAGACGGTGCAACGGCTGTTGCCGGTGCTGTGCCAGGCCCATGGCCTGACCCCGGACCAAGTGGTGGCCATCGCCAACA

ATAACGGCGGCAAGCAGGCGCTGGAGACGGTGCAGCGGCTGTTGCCGGTGCTGTGCCAGGACCATGGCCTGAGTCCGGAC

CAGGTGGTGGCCATCGCCAACAATAACGGCGGCAAGCAGGCGCTGGAGACGCTGCAGCGGCTGTTGCCGGTGCTGTGCCA

GACCCATGCCCTGACCCCGGACCAGGTGGTGGCCATCGCCAACAATAACGGCGGCAAGCAGGCGCTGG

>1P1R_2

CCAGCAATGGCGGCAAGCAGGCGCTGGAGACGGTGCAGCGGCTGTTGCCGGTGCTGTGCCAGGCTCATGGCCTGACCCCG

GACCAAGTGGTGGCCATCGCCAGTAATAGTGGCGGCAAGCAGGCGCTGGAGACGGTGCAGCGGCTGTTGCCGGTGCTGTG

CCAGGACCATGGCCTGACCCCGAACCAAGTGGTGGCCATCGCCAGCAATATTGGCGGCAAGCAGGCGCTGGAGACGGTGC

AGCGGCTGTTGCCGGTGCTATGCCAGGACCATGGCCTGACCCCGGACCAGGTGGTGGCCATCGCCAGCAATATTGGCGGC

AAGCAGGCGCTGGAGACGGTGCAGCGGCTGTTGCCGGTGCTGTGCCAGGACCATGGCCTGACCCTGGACCAGGTGGTGGC

CATCGCCAGCCACGATGGCGGCAAACAGGCGCTGGAGACGGTGCAGCGGCTGTTGCCGGTGCTGTGCCAGGCCCATGGCC

TGACCCCGGACCAGGTCGTGGCCATCGCCAGCCACGATGGCGGCAAGCAGGCGCTGGAGACGGTGCAGCGGCTGTTGCCG

GTGCTGTGCCAGGCCCATGGCCTGACCCTGGACAAGGTGGTGGCCATCGCCAGCAATGGCGGCAAGCAGGCGCTGGAGAC

GGTGCAGCGGCTGTTGCCGGTGCTGTGCCAGGACCATGGCCTGACCCCGAACCAGGTGGTGGCCATCGCCAGCAATAGTG

GCGGCAAGCAGGCGCTGGAGACGGTGCAGCGGCTGTTGCCGGTGCTGTGCCAGGACCATGGCCTGACCCCGAACCAGGTG

GTGGCCATCGCCAGCAATGGCGGCAAGCAGGCGCTGGAGAGCATTGT

> pCC2FOS-MscI-2

CCATCGCCAACAATAACGGCGGCAAGCAGGCGCTGGAGACGGTGCAGCGGCTGTTGCCGGTGCTGTGCCAGGACCATGGC

CTGACCCCGGACCAGGTGGTGGCCATCGCCAGCCACGATGGCGGCAAGCAGGCGCTGGAGACGGTGCAGCGGCTGTTGCC

GGTGCTGTGCCAGGACCATGGCCTGACCCCGGACCAAGTGGTGGCCATCGCCAGCAATATTGGCGGCAAGCAGGCGCTGG

AGACGGTGCAGCGGCTGTTGCCGGTGCTGTGCCAGGACCATGGCCTGACCCCGGACCAGGTGGTGGCCATCGCCAGCAAT

AGTGGCGGCAAGCAGGCGCTGGAGACGGTGCAGCGGCTGTTGCCGGTGCTGTGCCAGGACCATGGCCTGACCCCGGACCA

GGTGGTGGCCATCGCCAGCCATGGCGGCGGCAAGCAGGCGCTGGAGACGGTGCAGCGGCTGTTGCCGGTGCTGTGCCAGG

ACCATGGCCTGACCCCAGACCAGGTCGTGGCCATCGCCAGCCACGATGGCGGCAAGCAGGCGCTGGAGACGGTGCAGCGG

CTGTTGCCGGTGCTGTGCCAGGCCCATGGCCTGACCCCGAACCAGGTGGTGGCCATCGCCAGCAATATTGGCGGCAAGCA

GGCGCTGGAGACGGTGCAACGGCTGTTGCCGGTGCTGTGCCAGGACCATGGCCTGACCCCGGACCAGGTCGTGGCCATCG

CCAGCAATGGCGGCAAGCAGGCGCTGGAGACGGTGCAGCGGCTGTTGCCGGTGCTGTGCCAGGCTCATGGCCTGACCCCG

GACCAAGTGGTGGCCATCGCCAGTAATAGTGGCGGCAAGCAGGCGCTGGAGACGGTGCAGCGGCTGTTGCCGGTGC

> pCC2FOS-MscI-3

CCATCGCCAGCAATAACGGCGGCAAGCAGGCGCTGGAGACGGTGCAGCGGCTGTTGCCGGTGCTGTGCCAGGCCCATGGC

CTGACCCCGGACCAGGTGGTGGCTATCGCCAGCAATAACGGCGGCAAGCAGGCGCTGGAGACGGTGCAACGGCTGTTGCC

GGTGCTGTGCCAGGCCCATGGCCTGACCCCGGACCAAGTGGTGGCCATCGCCAACAATAACGGCGGCAAGCAGGCGCTGG

AGACGGTGCAGCGGCTGTTGCCGGTGCTGTGCCAGGACCATGGCCTGAGTCCGGACCAGGTGGTGGCCATCGCCAACAAT

AACGGCGGCAAGCAGGCGCTGGAGACGCTGCAGCGGCTGTTGCCGGTGCTGTGCCAGACCCATGCCCTGACCCCGGACCA

GGTGGTGGCCATCGCCAACAATAACGGCGGCAAGCAGGCGCTGGAGACGGTGCAGCGGCTGTTGCCGGTGCTGTGCCAGG

ACCATGGCCTGACCCCGGACCAGGTGGTGGCCATCGCCAGCCACGATGGCGGCAAGCAGGCGCTGGAGACGGTGCAGCGG

CTGTTGCCGGTGCTGTGCCAGGACCATGGCCTGACCCCGGACCAAGTGGTGGCCATCGCCAGCAATATTGGCGGCAAGCA

GGCGCTGGAGACGGTGCAGCGGCTGTTGCCGGTGCTGTGCCAGGACCATGGCCTGACCCCGGACCAGGTGGTGGCCATCG

CCAGCAATAGTGGCGGCAAGCAGGCGCTGGAGACGGTGCAGCGGCTGTTGCCGGTGCTGTGCCAGGACCATGGCCTGACC

CCGGACCAGGTGGTGGCCATCGCCAGCCATGGCGGCGGCAAGCAGGCGCTGGAGACGGTGCAGCGGCTGTTGCCGGTGC

> pCC2FOS-MscI-4

CCATCGCCAGCAATATTGGCGGCAAGCAGGCGCTGGAGACGGTGCAGCGGCTGTTGCCGGTGCTGTGCCAGGCCCATGGC

CTGACCCCGGCCCAGGTGGTGGCCATCGCCAGCAATAACGGCGGCAAGCAGGCGCTGGAGACGGTGCAGCGGCTGTTGCC

GGTGCTGTGCCAGGCCCATGGCCTGACCCCGGACCAGGTGGTGGCTATCGCCAGCAATAACGGCGGCAAGCAGGCGCTGG

AGACGGTGCAACGGCTGTTGCCGGTGCTGTGCCAGGCCCATGGCCTGACCCCGGACCAAGTGGTGGCCATCGCCAACAAT

AACGGCGGCAAGCAGGCGCTGGAGACGGTGCAGCGGCTGTTGCCGGTGCTGTGCCAGGACCATGGCCTGAGTCCGGACCA

GGTGGTGGCCATCGCCAACAATAACGGCGGCAAGCAGGCGCTGGAGACGCTGCAGCGGCTGTTGCCGGTGCTGTGCCAGA

CCCATGCCCTGACCCCGGACCAGGTGGTGGCCATCGCCAACAATAACGGCGGCAAGCAGGCGCTGGAGACGGTGCAGCGG

CTGTTGCCGGTGCTGTGCCAGGACCATGGCCTGACCCCGGACCAGGTGGTGGCCATCGCCAGCCACGATGGCGGCAAGCA

GGCGCTGGAGACGGTGCAGCGGCTGTTGCCGGTGCTGTGCCAGGACCATGGCCTGACCCCGGACCAAGTGGTGGCCATCG

CCAGCAATATTGGCGGCAAGCAGGCGCTGGAGACGGTGCAGCGGCTGTTGCCGGTGCTGTGCCAGGACCATGGCCTGACC

CCGGACCAGGTGGTGGCCATCGCCAGCAATAGTGGCGGCAAGCAGGCGCTGGAGACGGTGCAGCGGCTGTTGCCGGTGCT

GTGCCAGGACCAT

> pCC2FOS-MscI-5

AGCAGGCGCTGGAGACGGTGCAACGGCTGTTGCCGGTGCTGTGCCAGGACCATGGCCTGACCCCGGACCAGGTCGTGGCC

ATCGCCAGCAATGGCGGCAAGCAGGCGCTGGAGACGGTGCAGCGGCTGTTGCCGGTGCTGTGCCAGGCTCATGGCCTGAC

CCCGGACCAAGTGGTGGCCATCGCCAGTAATAGTGGCGGCAAGCAGGCGCTGGAGACGGTGCAGCGGCTGTTGCCGGTGC

TGTGCCAGGACCATGGCCTGACCCCGAACCAAGTGGTGGCCATCGCCAGCAATATTGGCGGCAAGCAGGCGCTGGAGACG

GTGCAGCGGCTGTTGCCGGTGCTATGCCAGGACCATGGCCTGACCCCGGACCAGGTGGTGGCCATCGCCAGCAATATTGG

CGGCAAGCAGGCGCTGGAGACGGTGCAGCGGCTGTTGCCGGTGCTGTGCCAGGACCATGGCCTGACCCTGGACCAGGTGG

TGGCCATCGCCAGCCACGATGGCGGCAAACAGGCGCTGGAGACGGTGCAGCGGCTGTTGCCGGTGCTGTGCCAGGCCCAT

GGCCTGACCCCGGACCAGGTCGTGGCCATCGCCAGCCACGATGGCGGCAAGCAGGCGCTGGAGACGGTGCAGCGGCTGTT

GCCGGTGCTGTGCCAGGCCCATGGCCTGACCCTGGACAAGGTGGTGGCCATCGCCAGCAATGGCGGCAAGCAGGCGCTGG

AGACGGTGCAGCGGCTGTTGCCGGTGCTGTGCCAGGACCATGGCCTGACCCCGAACCAGGTGGTGG

> pCC2FOS-MscI-6

CCATCGCCAGCAATATTGGCGGCAAGCAGGCGCTGGAGACGGTGCAGCGGCTGTTGCCGGTGCTGTGCCAGGCCCATGGC

CTGACCCCGGCCCAGGTGGTGGCCATCGCCAGCAATAACGGCGGCAAGCAGGCGCTGGAGACGGTGCAGCGGCTGTTGCC

GGTGCTGTGCCAGGCCCATGGCCTGACCCCGGACCAGGTGGTGGCTATCGCCAGCAATAACGGCGGCAAGCAGGCGCTGG

AGACGGTGCAACGGCTGTTGCCGGTGCTGTGCCAGGCCCATGGCCTGACCCCGGACCAAGTGGTGGCCATCGCCAACAAT

AACGGCGGCAAGCAGGCGCTGGAGACGGTGCAGCGGCTGTTGCCGGTGCTGTGCCAGGACCATGGCCTGAGTCCGGACCA

GGTGGTGGCCATCGCCAACAATAACGGCGGCAAGCAGGCGCTGGAGACGCTGCAGCGGCTGTTGCCGGTGCTGTGCCAGA

CCCATGCCCTGACCCCGGACCAGGTGGTGGCCATCGCCAACAATAACGGCGGCAAGCAGGCGCTGGAGACGGTGCAGCGG

CTGTTGCCGGTGCTGTGCCAGGACCATGGCCTGACCCCGGACCAGGTGGTGGCCATCGCCAGCCACGATGGCGGCAAGCA

GGCGCTGGAGACGGTGCAGCGGCTGTTGCCGGTGCTGTGCCAGGACCATGGCCTGACCCCGGACCAAGTGGTGGCCATCG

CCAGCAATATTGGCGGCAAGCAGGCGCTGGAGACGGTGCAGCGGCTGTTGCCGGTGCTGTGCCAGGACCATGGCCTGACC

CCGGACCAGGTGGTGGCCATCGCCAGCAATAGTGGCGGCAAGCAGGCGCTGGAGACGGTGCAGCGGCTGTTGCCGGTGCT

GTGCCAGGACCAT

> pCC2FOS-MscI-7

GGTCCTGGCACAGCACCGGCAACAGCCGCTGCACCGTCTCCAGCGCCTGCTTGCCGCCATCGTGGCTGGCGATGGCCACC

ACCTGGTCCGGGGTCAGGCCATGGTCCTGGCACAGCACCGGCAACAGCCGCTGCACCGTCTCCAGCGCCTGCTTGCCGCC

GTTATTGTTGGCGATGGCCACCACCTGGTCCGGGGTCAGGGCATGGGTCTGGCACAGCACCGGCAACAGCCGCTGCAGCG

TCTCCAGCGCCTGCTTGCCGCCGTTATTGTTGGCGATGGCCACCACCTGGTCCGGACTCAGGCCATGGTCCTGGCACAGC

ACCGGCAACAGCCGCTGCACCGTCTCCAGCGCCTGCTTGCCGCCGTTATTGTTGGCGATGGCCACCACTTGGTCCGGGGT

CAGGCCATGGGCCTGGCACAGCACCGGCAACAGCCGTTGCACCGTCTCCAGCGCCTGCTTGCCGCCGTTATTGCTGGCGA

TAGCCACCACCTGGTCCGGGGTCAGGCCATGGGCCTGGCACAGCACCGGCAACAGCCGCTGCACCGTCTCCAGCGCCTGC

TTGCCGCCGTTATTGCTGGCGATGGCCACCACCTGGGCCGGGGTCAGGCCATGGGCCTGGCACAGCACCGGCAACAGCCG

CTGCACCGTCTCCAGCGCCTGCTTGCCGCCAATATTGCTGGCGATGGCCACCACCTGGTCCGGGGTCAGGCCATGGTCCT

GGCACAGCACCGGCAACAGCCGCTGCACCGTCTCCAGCGCCTGCTTGCCGCCGTTATTGTTGGCGATGGCCACCACCTGG

TCCGGGGTCAGGCCATGGTCCTGGCACAGCACCGGCAACAGGCGCTGCACCGTCTCCAGCGCCTGCTTGCCGCCAATATT

GCTGGCGATGG

> pCC2FOS-MscI-8

CCATCGCCAGCCACGATGGCGGCAAGCAGGCGCTGGAGACGGTGCAGCGGCTGTTGCCGGTGCTGTGCCAGGCCCATGGC

CTGACCCCGAACCAGGTGGTGGCCATCGCCAGCAATATTGGCGGCAAGCAGGCGCTGGAGACGGTGCAACGGCTGTTGCC

GGTGCTGTGCCAGGACCATGGCCTGACCCCGGACCAGGTCGTGGCCATCGCCAGCAATGGCGGCAAGCAGGCGCTGGAGA

CGGTGCAGCGGCTGTTGCCGGTGCTGTGCCAGGCTCATGGCCTGACCCCGGACCAAGTGGTGGCCATCGCCAGTAATAGT

GGCGGCAAGCAGGCGCTGGAGACGGTGCAGCGGCTGTTGCCGGTGCTGTGCCAGGACCATGGCCTGACCCCGAACCAAGT

GGTGGCCATCGCCAGCAATATTGGCGGCAAGCAGGCGCTGGAGACGGTGCAGCGGCTGTTGCCGGTGCTATGCCAGGACC

ATGGCCTGACCCCGGACCAGGTGGTGGCCATCGCCAGCAATATTGGCGGCAAGCAGGCGCTGGAGACGGTGCAGCGGCTG

TTGCCGGTGCTGTGCCAGGACCATGGCCTGACCCTGGACCAGGTGGTGGCCATCGCCAGCCACGATGGCGGCAAACAGGC

GCTGGAGACGGTGCAGCGGCTGTTGCCGGTGCTGTGCCAGGCCCATGGCCTGACCCCGGACCAGGTCGTGGCCATCGCCA

GCCACGATGGCGGCAAGCAGGCGCTGGAGACGGTGCAGCGGCTGTTGCCGGTGCTGTGCCAGGCCCATGGCCTGACCCTG

GACAAGGTGGTGGCCATCGCCAGCAATGGCGGCAAGCAGGCGCTGGAGACGGTGCAGCGGCTGTTGCCGGTGCTGTGCCA

G

> pCC2FOS-MscI-9

CCATCGCCAGCAATATTGGCGGCAAGCAGGCGCTGGAGACGGTGCAGCGGCTGTTGCCGGTGCTGTGCCAGGCCCATGGC

CTGACCCCGGCCCAGGTGGTGGCCATCGCCAGCAATAACGGCGGCAAGCAGGCGCTGGAGACGGTGCAGCGGCTGTTGCC

GGTGCTGTGCCAGGCCCATGGCCTGACCCCGGACCAGGTGGTGGCTATCGCCAGCAATAACGGCGGCAAGCAGGCGCTGG

AGACGGTGCAACGGCTGTTGCCGGTGCTGTGCCAGGCCCATGGCCTGACCCCGGACCAAGTGGTGGCCATCGCCAACAAT

AACGGCGGCAAGCAGGCGCTGGAGACGGTGCAGCGGCTGTTGCCGGTGCTGTGCCAGGACCATGGCCTGAGTCCGGACCA

GGTGGTGGCCATCGCCAACAATAACGGCGGCAAGCAGGCGCTGGAGACGCTGCAGCGGCTGTTGCCGGTGCTGTGCCAGA

CCCATGCCCTGACCCCGGACCAGGTGGTGGCCATCGCCAACAATAACGGCGGCAAGCAGGCGCTGGAGACGGTGCAGCGG

CTGTTGCCGGTGCTGTGCCAGGACCATGGCCTGACCCCGGACCAGGTGGTGGCCATCGCCAGCCACGATGGCGGCAAGCA

GGCGCTGGAGACGGTGCAGCGGCTGTTGCCGGTGCTGTGCCAGGACCATGGCCTGACCCCGGACCAAGTGGTGGCCATCG

CCAGCAATATTGGCGGCAAGCAGGCGCTGGAGACGGTGCAGCGGCTGTTGCCGGTGCTGTGCCAGGACCATGGCCTGACC

CCGGACCAGGTGGTGGCCATCGCCAGCAATAGTGGCGGCAAGCAGGCGCTGGAGACGGTGCAGCGGCTGTTGCCGGTGCT

GTGCCA

> pCC2FOS-MscI-10

CCATCGCCAGCCATGGCGGCGGCAAGCAGGCGCTGGAGACGGTGCAGCGGCTGTTGCCGGTGCTGTGCCAGGACCATGGC

CTGACCCCAGACCAGGTCGTGGCCATCGCCAGCCACGATGGCGGCAAGCAGGCGCTGGAGACGGTGCAGCGGCTGTTGCC

GGTGCTGTGCCAGGCCCATGGCCTGACCCCGAACCAGGTGGTGGCCATCGCCAGCAATATTGGCGGCAAGCAGGCGCTGG

AGACGGTGCAACGGCTGTTGCCGGTGCTGTGCCAGGACCATGGCCTGACCCCGGACCAGGTCGTGGCCATCGCCAGCAAT

GGCGGCAAGCAGGCGCTGGAGACGGTGCAGCGGCTGTTGCCGGTGCTGTGCCAGGCTCATGGCCTGACCCCGGACCAAGT

GGTGGCCATCGCCAGTAATAGTGGCGGCAAGCAGGCGCTGGAGACGGTGCAGCGGCTGTTGCCGGTGCTGTGCCAGGACC

ATGGCCTGACCCCGAACCAAGTGGTGGCCATCGCCAGCAATATTGGCGGCAAGCAGGCGCTGGAGACGGTGCAGCGGCTG

TTGCCGGTGCTATGCCAGGACCATGGCCTGACCCCGGACCAGGTGGTGGCCATCGCCAGCAATATTGGCGGCAAGCAGGC

GCTGGAGACGGTGCAGCGGCTGTTGCCGGTGCTGTGCCAGGACCATGGCCTGACCCTGGACCAGGTGGTGGCCATCGCCA

GCCACGATGGCGGCAAACAGGCGCTGGAGACGGTGCAGCGGCTGTTGCCGGTGCTGT

> pCC2FOS-MscI-11

CCATCGCCAGCAATATTGGCGGCAAGCAGGCGCTGGAGACGGTGCAGCGCCTGTTGCCGGTGCTGTGCCAGGACCATGGC

CTGACCCCGGACCAGGTGGTGGCCATCGCCAACAATAACGGCGGCAAGCAGGCGCTGGAGACGGTGCAGCGGCTGTTGCC

GGTGCTGTGCCAGGACCATGGCCTGACCCCGGACCAGGTGGTGGCCATCGCCAGCAATATTGGCGGCAAGCAGGCGCTGG

AGACGGTGCAGCGGCTGTTGCCGGTGCTGTGCCAGGCCCATGGCCTGACCCCGGCCCAGGTGGTGGCCATCGCCAGCAAT

AACGGCGGCAAGCAGGCGCTGGAGACGGTGCAGCGGCTGTTGCCGGTGCTGTGCCAGGCCCATGGCCTGACCCCGGACCA

GGTGGTGGCTATCGCCAGCAATAACGGCGGCAAGCAGGCGCTGGAGACGGTGCAACGGCTGTTGCCGGTGCTGTGCCAGG

CCCATGGCCTGACCCCGGACCAAGTGGTGGCCATCGCCAACAATAACGGCGGCAAGCAGGCGCTGGAGACGGTGCAGCGG

CTGTTGCCGGTGCTGTGCCAGGACCATGGCCTGAGTCCGGACCAGGTGGTGGCCATCGCCAACAATAACGGCGGCAAGCA

GGCGCTGGAGACGCTGCAGCGGCTGTTGCCGGTGCTGTGCCAGACCCATGCCCTGACCCCGGACCAGGTGGTGGCCATCG

CCAACAATAACGGCGGCAAGCAGGCGCTGGAGACGGTGCAGCGGCTGTTGCCGGTGCTGTGCCAGGACCATGGCCTGACC

CCGGACCAGGTGGTGGCCATCGCCAGCCACGATGGCGGCAAGCAGGCGCTGGAGACGGTGCAGCGGCTGTTGCCGGTGC

> pCC2FOS-MscI-12

CCATCGCCAGCCACGATGGCGGCAAGCAGGCGCTGGAGACGGTGCAGCGGCTGTTGCCGGTGCTGTGCCAGGACCATGGC

CTGACCCCGGACCAAGTGGTGGCCATCGCCAGCAATATTGGCGGCAAGCAGGCGCTGGAGACGGTGCAGCGGCTGTTGCC

GGTGCTGTGCCAGGACCATGGCCTGACCCCGGACCAGGTGGTGGCCATCGCCAGCAATAGTGGCGGCAAGCAGGCGCTGG

AGACGGTGCAGCGGCTGTTGCCGGTGCTGTGCCAGGACCATGGCCTGACCCCGGACCAGGTGGTGGCCATCGCCAGCCAT

GGCGGCGGCAAGCAGGCGCTGGAGACGGTGCAGCGGCTGTTGCCGGTGCTGTGCCAGGACCATGGCCTGACCCCAGACCA

GGTCGTGGCCATCGCCAGCCACGATGGCGGCAAGCAGGCGCTGGAGACGGTGCAGCGGCTGTTGCCGGTGCTGTGCCAGG

CCCATGGCCTGACCCCGAACCAGGTGGTGGCCATCGCCAGCAATATTGGCGGCAAGCAGGCGCTGGAGACGGTGCAACGG

CTGTTGCCGGTGCTGTGCCAGGACCATGGCCTGACCCCGGACCAGGTCGTGGCCATCGCCAGCAATGGCGGCAAGCAGGC

GCTGGAGACGGTGCAGCGGCTGTTGCCGGTGCTGTGCCAGGCTCATGGCCTGACCCCGGACCAAGTGGTGGCCATCGCCA

GTAATAGTGGCGGCAAGCAGGCGCTGGAGACGGTGCAGCGGCTGTTGCCGGTGCTGTGCCAGGACCATGGCCTGACCCCG

AACCAAGTGGTGGCCATCGCCAGCAATATTGGCGGCAAGCAGGCGCTGGAGACGGTGCAGCGGCTGTTGCCGGTGCTATG

CC

> pCC2FOS-MscI-13

CCACCACCTGGTCCAGGGTCAGGCCATGGTCCTGGCACAGCACCGGCAACAGCCGCTGCACCGTCTCCAGCGCCTGCTTG

CCGCCAATATTGCTGGCGATGGCCACCACCTGGTCCGGGGTCAGGCCATGGTCCTGGCATAGCACCGGCAACAGCCGCTG

CACCGTCTCCAGCGCCTGCTTGCCGCCAATATTGCTGGCGATGGCCACCACTTGGTTCGGGGTCAGGCCATGGTCCTGGC

ACAGCACCGGCAACAGCCGCTGCACCGTCTCCAGCGCCTGCTTGCCGCCACTATTACTGGCGATGGCCACCACTTGGTCC

GGGGTCAGGCCATGAGCCTGGCACAGCACCGGCAACAGCCGCTGCACCGTCTCCAGCGCCTGCTTGCCGCCATTGCTGGC

GATGGCCACGACCTGGTCCGGGGTCAGGCCATGGTCCTGGCACAGCACCGGCAACAGCCGTTGCACCGTCTCCAGCGCCT

GCTTGCCGCCAATATTGCTGGCGATGGCCACCACCTGGTTCGGGGTCAGGCCATGGGCCTGGCACAGCACCGGCAACAGC

CGCTGCACCGTCTCCAGCGCCTGCTTGCCGCCATCGTGGCTGGCGATGGCCACGACCTGGTCTGGGGTCAGGCCATGGTC

CTGGCACAGCACCGGCAACAGCCGCTGCACCGTCTCCAGCGCCTGCTTGCCGCCGCCATGGCTGGCGATGGCCACCACCT

GGTCCGGGGTCAGGCCATGGTCCTGGCACAGCACCGGCAACAGCCGCTGCACCGTCTCCAGCGCCTGCTTGCCGCCACTA

TTGCTGGCGATGGCCACCACCTGGTCCGGGGTCAGGCCATGGTCCTGGCACAGCACCGGCAACAGCCGCTGCACCGTCTC

CAGCGCCTGC

> pCC2FOS-MscI-14

CCATCGCCAGCAATATTGGCGGCAAGCAGGCGCTGGAGACGGTGCAGCGGCTGTTGCCGGTGCTGTGCCAGGACCATGGC

CTGACCCCGGACCAGGTGGTGGCCATCGCCAGCAATAGTGGCGGCAAGCAGGCGCTGGAGACGGTGCAGCGGCTGTTGCC

GGTGCTGTGCCAGGACCATGGCCTGACCCCGGACCAGGTGGTGGCCATCGCCAGCCATGGCGGCGGCAAGCAGGCGCTGG

AGACGGTGCAGCGGCTGTTGCCGGTGCTGTGCCAGGACCATGGCCTGACCCCAGACCAGGTCGTGGCCATCGCCAGCCAC

GATGGCGGCAAGCAGGCGCTGGAGACGGTGCAGCGGCTGTTGCCGGTGCTGTGCCAGGCCCATGGCCTGACCCCGAACCA

GGTGGTGGCCATCGCCAGCAATATTGGCGGCAAGCAGGCGCTGGAGACGGTGCAACGGCTGTTGCCGGTGCTGTGCCAGG

ACCATGGCCTGACCCCGGACCAGGTCGTGGCCATCGCCAGCAATGGCGGCAAGCAGGCGCTGGAGACGGTGCAGCGGCTG

TTGCCGGTGCTGTGCCAGGCTCATGGCCTGACCCCGGACCAAGTGGTGGCCATCGCCAGTAATAGTGGCGGCAAGCAGGC

GCTGGAGACGGTGCAGCGGCTGTTGCCGGTGCTGTGCCAGGACCATGGCCTGACCCCGAACCAAGTGGTGGCCATCGCCA

GCAATATTGGCGGCAAGCAGGCGCTGGAGACGGTGCAGCGGCTGTTGCCGGTGCTATGCCAGGACC

>M13F

GGATCCCATTCGTTCGCGCACGCCAAGTCCTGCCCGCGAGCTTCTGCCCGGACCCCAACCGGATAGGGTTCAGCCGACTG

CAGATCGGGGGGGGGCTCCGCCTGCTGGCGGCCCCCTGGATGGCTTGCCCGCTCGGCGGACGATGTCCCGGACCCGGCTG

CCATCTCCCCCTGCGCCCTCGCCTGCGTTCTCGGCGGGCAGCTTCAGCGATCTGCTCCGTCAGTTCGATCCGTCGCTTCT

TGATACATCGCTTCTTGATTCGATGCCTGCCGTCGGCACGCCGCATACAGCGGCTGCCCCAGCAGAATGGGATGAGGCGC

AATCGGGTCTGCGTGCAGCCGATGACCCGCCACCCACCGTGCCTGTCGCTGTCACTGCCGCGCGGCCGCCGCGCGCCAAG

CCGGCCCCGCGACGGCGTGCGGCGCAACCCTCCGACGCTTCGCCGGCCGCGCAGGTGGATCTACGCACGCTCGGCTACAG

TCAGCAGCAGCAAGAGAAGATCAAACCGAAGGTGCGTTCGACAGTGGCGCAGCACCACGAGGCACTGGTGGGCCATGGGT

TTACACACGCGCACATCGTTGCGCTCAGCCAACACCCGGCAGCGTTAGGGACCGTTGCTGTCACGTATCAGGACATAATC

AGGGCGTTGCCAGAGGCGACACACGAAGACATCGTTGGCGTCGGCAAACAGTGGTCCGGCGCACGCGCCCTGGAGGCCTT

GCTCACGAAGGCGGGGGAGTTGAGAGGTCCACCGTTACAGTTGGACACAGGCCAACTTGTCAAGATTGCAAAACGTGGCG

GCGTGACCGCAGTGGAGGCAGTGCATGCATCGCGCAATGCACTGACGGGTGCCCCCCTGAACCTGACCCCGGACCAAGTG

GTGGCCATCGCCAGCAATATTGGCGGCAAGCAGGCGCTGGAGACGGTGCAGCGCCTGTTGCCGGTGCT

>M13R

GCCAGGCCCATGGCCTGACCCTGGACAAGGTGGTGGCCATCGCCAGCAATGGCGGCAAGCAGGCGCTGGAGACGGTGCAG

CGGCTGTTGCCGGTGCTGTGCCAGGACCATGGCCTGACCCCGAACCAGGTGGTGGCCATCGCCAGCAATAGTGGCGGCAA

GCAGGCGCTGGAGACGGTGCAGCGGCTGTTGCCGGTGCTGTGCCAGGACCATGGCCTGACCCCGAACCAGGTGGTGGCCA

TCGCCAGCAATGGCGGCAAGCAGGCGCTGGAGAGCATTGTTGCCCAGTTATCTCGCCCTGATCCGGCGTTGGCCGCGTTG

ACCAACGACCACCTCGTCGCCTTGGCCTGCCTCGGCGGACGTCCTGCCCTGGATGCAGTGAAAAAGGGATTGCCGCACGC

GCCGGAATTGATCAGAAGAATCAATCGCCGCATTCCCGAACGCACGTCCCATCGCGTTCCCGACCTCGCGCACGTGGTTC

GCGTGCTTGGTTTTTTCCAGAGCCACTCCCACCCAGCGCAAGCATTCGATGACGCCATGACGCAGTTCGAGATGAGCAGG

CACGGCTTGGTACAGCTCTTTCGCAGAGTGGGCGTCACCGAATTCGAAGCCCGCTACGGAACGCTCCCCCCAGCCTCGCA

GCGTTGGGACCGTATCCTCCAGGCATCAGGGATGAAAAGGGCCAAACCGTCCCCTACTTCAGCTCAAACACCGGATCAGG

CGTCTTTGCATGCATTCGCCGATTCGCTGGAGCGTGACCTTGATGCGCCTAGCCCAATGCACGAGGGAGATCAGACAGGG

GCAAGCAGCCGTAAACGGTCCCGATCGGATCGTGCTGTCACCGGCCCCTCCGCACAGCAATCTTTCGAGGTGCGCGTTCC

CGAACAGCGCGATGCGCTGCATTTGCCCCTCAGCTGGAGGGTAAAACGCCCGCGTACCAGGATCGGGGGCGGCCTCCCGG

ATCC

**pTAL*_Bam_*_HI_-6**

> pCC2FOS-MscI-1

CCATCGCCAGCAATATTGGCGGCAAGCAGGCGCTGGAGACGGTGCAGCGGCTGTTGCCGGTGCTGTGCCAGGACCATGGC

CTGACCCCGGACCAGGTCGTGGCCATCGCCAGTAATATTGGCGGCAAGCAGGCGCTGGAGACGGTGCAGCGGCTGTTGCC

GGTGCTGTGCCAGGACCATGGCCTGACCCCGGACCAGGTCGTGGCCATCGCCAGCAATAACGGCGGCAAGCAGGCGCTGG

AGACGGTGCAGCGGCTGTTGCCGGTGCTGTGCCAGGACCATGGCCTGACCCCGGACCAGGTCGTGGCCATCGCCAGCCAC

GATGGCGGCAAGCAGGCGCTGGAGACGGTGCAGCGGCTGTTGCCGGTGCTGTGCCAGGACCATGGCCTGACCCCGGCGCA

GGTGGTGGCCATCGCCAGCCATATTGGCGGCAAGCAGGCGCTGGAGACGGTGCAGCGGCTGTTGCCGGTGCTGTGCCAGG

ACCATGGCCTGACCCTGGACCAGGTGGTGGCCATTGCCAGCAATGACGGCAGCAAGCAGGCGCTGGAGACGGTGCAGCGG

CTGTTGCCGGTGCTGTGCCAGGACCATGGCCTGACCCCGGACCAGGTGGTGGCCATCGCCAGCCACGATGGCGGCAAGCA

GGCGCTGGAGACGGTGCAGCGGCTGTTGCCGGTGCTGTGCCAGGACCATGGCCTGACCCCGAACCAAGTGGTGGCCATCG

CCAGCAATATTGGCGGCAAGCAGGCGCTGGAGACGGTGCAGCGGCTGTTGCCGGTGCTGTGCCAGGCCCATGGCCTGACC

CCGGACCAGGTCGTGGCCATCGCCAGCCACGATGGCGGCAAGCAGGCGCTGGAGACGGTGCAGCGGCTGTTGCCGGTGCT

GTGCCAGAACCA

> pCC2FOS-MscI-2

CCATCGCCAGTAATATTGGCGGCAAGCAGGCGCTGGAGACGGTGCAGCGGCTGTTGCCGGTGCTGTGCCAGGACCATGGC

CTGACCCCGGACCAGGTCGTGGCCATCGCCAGCAATAACGGCGGCAAGCAGGCGCTGGAGACGGTGCAGCGGCTGTTGCC

GGTGCTGTGCCAGGACCATGGCCTGACCCCGGACCAGGTCGTGGCCATCGCCAGCCACGATGGCGGCAAGCAGGCGCTGG

AGACGGTGCAGCGGCTGTTGCCGGTGCTGTGCCAGGACCATGGCCTGACCCCGGCGCAGGTGGTGGCCATCGCCAGCCAT

ATTGGCGGCAAGCAGGCGCTGGAGACGGTGCAGCGGCTGTTGCCGGTGCTGTGCCAGGACCATGGCCTGACCCTGGACCA

GGTGGTGGCCATTGCCAGCAATGACGGCAGCAAGCAGGCGCTGGAGACGGTGCAGCGGCTGTTGCCGGTGCTGTGCCAGG

ACCATGGCCTGACCCCGGACCAGGTGGTGGCCATCGCCAGCCACGATGGCGGCAAGCAGGCGCTGGAGACGGTGCAGCGG

CTGTTGCCGGTGCTGTGCCAGGACCATGGCCTGACCCCGAACCAAGTGGTGGCCATCGCCAGCAATATTGGCGGCAAGCA

GGCGCTGGAGACGGTGCAGCGGCTGTTGCCGGTGCTGTGCCAGGCCCATGGCCTGACCCCGGACCAGGTCGTGGCCATCG

CCAGCCACGATGGCGGCAAGCAGGCGCTGGAGACGGTGCAGCGGCTGTTGCCGGTGCTGTGCCAGAACCATGGCCTGACC

CCGGACCAGGTCGTGGCCATCGCCAGCAATGGCGGCGGCAAGCAGGCGCTGGAGACGGTGCAACGGCTGTTGCCGGT

> pCC2FOS-MscI-3

CCATTGCCAGCAATAACGGCGGCAAGCAGGCGCTGGAGACGGTGCAGCGGCTGTTGCCGGTGCTGTGCCAGGACCATGGC

CTGACCCCGGACCAGGTCGTGGCCATCGCCAGCCACGATGGCGGCAAGCAGGCGCTGGAGACGGTGCAGCGGCTGTTGCC

GGTGCTGTGCCAGGACCATGGCCTGACCCCGGACCAGGTCGTGGCCATCGCCAGCCACGATGGCGGCAAGCAGGCGCTGG

AGACGGTGCAGCGGCTGTTGCCGGTGCTGTGCCAGGACCATGGCCTGACCCCGGACCAGGTGGTGGCCATCGCCAGCAAT

GGCGGCAAGCAGGCGCTGGAGACGGTGCAGCGGCTGTTGCCGGTGCTGTGCCAGGACCATGGCCTGACCCTGGCGCAGGT

GGTGGCCATCGCCAGCAATATTGGCGGCAAGCAGGCGCTGGAGACGGTGCAGCGGCTGTTGCCGGTGCTGTGCCAGGACC

ATGGCCTGACCCCGGACCAGGTCGTGGCCATCGCCAGTAATATTGGCGGCAAGCAGGCGCTGGAGACGGTGCAGCGGCTG

TTGCCGGTGCTGTGCCAGGACCATGGCCTGACCCCGGACCAGGTCGTGGCCATCGCCAGCAATAACGGCGGCAAGCAGGC

GCTGGAGACGGTGCAGCGGCTGTTGCCGGTGCTGTGCCAGGACCATGGCCTGACCCCGGACCAGGTCGTGGCCATCGCCA

GCCACGATGGCGGCAAGCAGGCGCTGGAGACGGTGCAGCGGCTGTTGCCGGTGCTGTGCCAGGACCATGGCCTGACCCCG

GCGCAGGTGGTGGCCATCGCCAGCCATATTGGCGGCAAGCAGGCGCTGGAGACGGTGCAGCGGCTGTTGCCGGTGCTGTG

CCAGG

> pCC2FOS-MscI-4

CCATCGCCAGCCACGATGGCGGCAAGCAGGCTCTGGAGACGGTGCAGCGGCTGTTGCCGGTGCTGTGCCAGGACCATGGC

CTGACCCCGGACCAGGTCGTGGCCATCGCCAGCCACGATGGCGGCAAGCAGGCGCTGGAGACGGTGCAGCGGCTGTTGCC

GGTGCTGTGCCAGGACCATGGCCTGACCCCGGCGCAGGCGGTGGCCATCGCCAGCAATGGCGGCGGCAAGCAGGCGCTGG

AGACGGTGCAACGGCTGTTGCCGGTGCTGTGCCAGGACCATGGCCTGACCCCGGACCAGGTGGTGGCCATCGCCAGCAAT

GGCGGCAAGCAGGCGCTGGAGACGGTGCAGCGGCTGTTGCCGGTGCTGTGCCAGGACCATGGCCTGACCCCGGACCAGGT

CGTGGCCATTGCCAGCAATAACGGCGGCAAGCAGGCGCTGGAGACGGTGCAGCGGCTGTTGCCGGTGCTGTGCCAGGACC

ATGGCCTGACCCCGGACCAGGTCGTGGCCATCGCCAGCCACGATGGCGGCAAGCAGGCGCTGGAGACGGTGCAGCGGCTG

TTGCCGGTGCTGTGCCAGGACCATGGCCTGACCCCGGACCAGGTCGTGGCCATCGCCAGCCACGATGGCGGCAAGCAGGC

GCTGGAGACGGTGCAGCGGCTGTTGCCGGTGCTGTGCCAGGACCATGGCCTGACCCCGGACCAGGTGGTGGCCATCGCCA

GCAATGGCGGCAAGCAGGCGCTGGAGACGGTGCAGCGGCTGTTGCCGGTGCTGTGCCAGGACCATGGCCTGACCCTGGCG

CAGGTGGTGGCCATCGCCAGCAATATTGGCGGCAAGCAGGCGCTGGAGACGGTGCAGCGGCTGTTGCCGGTG

> pCC2FOS-MscI-5

CCATCGCCAGCCACGATGGCGGCAAGCAGGCGCTGGAGACGGTGCAGCGGCTGTTGCCGGTGCTGTGCCAGGACCATGGC

CTGACCCCGGACCAGGTGGTGGCCATCGCCAGCAATGGCGGCAAGCAGGCGCTGGAGACGGTGCAGCGGCTGTTGCCGGT

GCTGTGCCAGGACCATGGCCTGACCCTGGCGCAGGTGGTGGCCATCGCCAGCAATATTGGCGGCAAGCAGGCGCTGGAGA

CGGTGCAGCGGCTGTTGCCGGTGCTGTGCCAGGACCATGGCCTGACCCCGGACCAGGTCGTGGCCATCGCCAGTAATATT

GGCGGCAAGCAGGCGCTGGAGACGGTGCAGCGGCTGTTGCCGGTGCTGTGCCAGGACCATGGCCTGACCCCGGACCAGGT

CGTGGCCATCGCCAGCAATAACGGCGGCAAGCAGGCGCTGGAGACGGTGCAGCGGCTGTTGCCGGTGCTGTGCCAGGACC

ATGGCCTGACCCCGGACCAGGTCGTGGCCATCGCCAGCCACGATGGCGGCAAGCAGGCGCTGGAGACGGTGCAGCGGCTG

TTGCCGGTGCTGTGCCAGGACCATGGCCTGACCCCGGCGCAGGTGGTGGCCATCGCCAGCCATATTGGCGGCAAGCAGGC

GCTGGAGACGGTGCAGCGGCTGTTGCCGGTGCTGTGCCAGGACCATGGCCTGACCCTGGACCAGGTGGTGGCCATTGCCA

GCAATGACGGCAGCAAGCAGGCGCTGGAGACGGTGCAGCGGCTGTTGCCGGTGCTGTGCCAGGACCATGGCCTGACCCCG

GACCAGGTGGTGGCCATCGCCAGCCACGATGGCGGCAAGCAGGCGCTGGAGACGGTGCAGCGGCTGTTGCCGGTGCTGTG

CCAGGACC

> pCC2FOS-MscI-6

CGCCAGCCACGATGGCGGCAAGCAGGCGCTGGAGACGGTGCAGCGGCTGTTGCCGGTGCTGTGCCAGGACCATGGCCTGA

CCCCGGACCAGGTCGTGGCCATCGCCAGCCACGATGGCGGCAAGCAGGCGCTGGAGACGGTGCAGCGGCTGTTGCCGGTG

CTGTGCCAGGACCATGGCCTGACCCCGGACCAGGTGGTGGCCATCGCCAGCAATGGCGGCAAGCAGGCGCTGGAGACGGT

GCAGCGGCTGTTGCCGGTGCTGTGCCAGGACCATGGCCTGACCCTGGCGCAGGTGGTGGCCATCGCCAGCAATATTGGCG

GCAAGCAGGCGCTGGAGACGGTGCAGCGGCTGTTGCCGGTGCTGTGCCAGGACCATGGCCTGACCCCGGACCAGGTCGTG

GCCATCGCCAGTAATATTGGCGGCAAGCAGGCGCTGGAGACGGTGCAGCGGCTGTTGCCGGTGCTGTGCCAGGACCATGG

CCTGACCCCGGACCAGGTCGTGGCCATCGCCAGCAATAACGGCGGCAAGCAGGCGCTGGAGACGGTGCAGCGGCTGTTGC

CGGTGCTGTGCCAGGACCATGGCCTGACCCCGGACCAGGTCGTGGCCATCGCCAGCCACGATGGCGGCAAGCAGGCGCTG

GAGACGGTGCAGCGGCTGTTGCCGGTGCTGTGCCAGGACCATGGCCTGACCCCGGCGCAGGTGGTGGCCATCGCCAGCCA

TATTGGCGGCAAGCAGGCGCTGGAGACGGTGCAGCGGCTGTTGCCGGTGCTGTGCCAGGACCATGGCCTGACCCTGGACC

AGGTGGTGGCCATTGCCAGCAATGACGGCAGCAAGCAGGCGCTGGAGACGGTGCAGCGGCTGTTGCCGGTGCTGTGCCAG

GACCATGGCCTGACCCCGGACCAGGTGGTGGCCATCGCCAGCC

>6P1F_1

CGGGGGAGTTGAGAGGTCCGCCGTTACAGTTGGACACAGGCCAACTTCTCAAGATTGCAAAACGTGGCGGCGTGACCGCA

GTGAAGGCAGTGCATGCATGGCGCAATGCACTGACGGGTGCCCCCCTGAACCTGACCCCGGCACAGGTGGTGGCCATCGC

CAGCCACGATGGCGGCAATCAGGCGCTGGAGACGGTGCAGCGGCTGTTGCCGGTGCTGTGCCAGGACCATGGCCTGACCC

CGGCGCAGGTGGTGGCCATCGCCAGCCACGATGGCGGCAAGCAGGCTCTGGAGACGGTGCAGCGGCTGTTGCCGGTGCTG

TGCCAGGACCATGGCCTGACCCCGGACCAGGTCGTGGCCATCGCCAGCCACGATGGCGGCAAGCAGGCGCTGGAGACGGT

GCAGCGGCTGTTGCCGGTGCTGTGCCAGGACCATGGCCTGACCCCGGCGCAGGCGGTGGCCATCGCCAGCAATGGCGGCG

GCAAGCAGGCGCTGGAGACGGTGCAACGGCTGTTGCCGGTGCTGTGCCAGGACCATGGCCTGACCCCGGACCAGGTGGTG

GCCATCGCCAGCAATGGCGGCAAGCAGGCGCTGGAGACGGTGCAGCGGCTGTTGCCGGTGCTGTGCCAGGACCATGGCCT

GACCCCGGACCAGGTCGTGGCCATTGCCAGCAATAACGGCGGCAAGCAGGCGCTGGAGACGGTGCAGCGGCTGTTGCCGG

TGCTGTGCCAGGACCATGGCCTGACCCCGGACCAGGTCGTGGCCATCGCCAGCCACGATGGCGGCAAGCAGGCGCTGGAG

ACGGTGCAGCGGCTGTTGCCGGTGCTGTGCCAGGACCATGGCCTGACCCC

>6P1F_2

CCGCCGTTACAGTTGGACACAGGCCAACTTCTCAAGATTGCAAAACGTGGCGGCGTGACCGCAGTGAAGGCAGTGCATGC

ATGGCGCAATGCACTGACGGGTGCCCCCCTGAACCTGACCCCGGCACAGGTGGTGGCCATCGCCAGCCACGATGGCGGCA

ATCAGGCGCTGGAGACGGTGCAGCGGCTGTTGCCGGTGCTGTGCCAGGACCATGGCCTGACCCCGGCGCAGGTGGTGGCC

ATCGCCAGCCACGATGGCGGCAAGCAGGCTCTGGAGACGGTGCAGCGGCTGTTGCCGGTGCTGTGCCAGGACCATGGCCT

GACCCCGGACCAGGTCGTGGCCATCGCCAGCCACGATGGCGGCAAGCAGGCGCTGGAGACGGTGCAGCGGCTGTTGCCGG

TGCTGTGCCAGGACCATGGCCTGACCCCGGCGCAGGCGGTGGCCATCGCCAGCAATGGCGGCGGCAAGCAGGCGCTGGAG

ACGGTGCAACGGCTGTTGCCGGTGCTGTGCCAGGACCATGGCCTGACCCCGGACCAGGTGGTGGCCATCGCCAGCAATGG

CGGCAAGCAGGCGCTGGAGACGGTGCAGCGGCTGTTGCCGGTGCTGTGCCAGGACCATGGCCTGACCCCGGACCAGGTCG

TGGCCATTGCCAGCAATAACGGCGGCAAGCAGGCGCTGGAGACGGTGCAGCGGCTGTTGCCGGTGCTGTGCCAGGACCAT

GGCCTGACCCCGGACCAGGTCGTGGCCATCGCCAGCCACGATGGCGGCAAGCAGGCGCTGGAGACGGTGCAGCGGCTGTT

GCCGGTGCTGTGCCAGGACCATGGCCTGACCCCGGACCAGGTCGTGGCCATCGCCAGCCACGATGGCGGCAAGCAGGCGC

TGGAGACGGTGCAGCGGCTGTTGCCGGTGCTGTGCCAGGACCATGGCCTGACCCCGGACCAGGTGGTGGCCATCGCC

>6P1R_1

ACAATGCTCTCCAGCGCCTGCTTGCCGCCGCCATTGCTGGCGATGGCCACGACCTGGTCCGGGGTCAGGCCATGGTCCTG

GCACAGCACCGGCAACAGCCGTTGCACCGTCTCCAGCGCCTGCTTGCCGCCGCCATTGCTGGCGATGGCCACGACCTGGT

CCGGGGTCAGGCCATGGTTCTGGCACAGCACCGGCAACAGCCGCTGCACCGTCTCCAGCGCCTGCTTGCCGCCATCGTGG

CTGGCGATGGCCACGACCTGGTCCGGGGTCAGGCCATGGGCCTGGCACAGCACCGGCAACAGCCGCTGCACCGTCTCCAG

CGCCTGCTTGCCGCCAATATTGCTGGCGATGGCCACCACTTGGTTCGGGGTCAGGCCATGGTCCTGGCACAGCACCGGCA

ACAGCCGCTGCACCGTCTCCAGCGCCTGCTTGCCGCCATCGTGGCTGGCGATGGCCACCACCTGGTCCGGGGTCAGGCCA

TGGTCCTGGCACAGCACCGGCAACAGCCGCTGCACCGTCTCCAGCGCCTGCTTGCTGCCGTCATTGCTGGCAATGGCCAC

CACCTGGTCCAGGGTCAGGCCATGGTCCTGGCACAGCACCGGCAACAGCCGCTGCACCGTCTCCAGCGCCTGCTTGCCGC

CAATATGGCTGGCGATGGCCACCACCTGCGCCGGGGTCAGGCCATGGTCCTGGCACAGCACCGGCAACAGCCGCTGCACC

GTCTCCAGCGCCTGCTTGCCGCCATCGTGGCTGGCGATGGCCACGACCTGGTCCGGGGTCAGGCCATGGTCCTGGCACAG

CACCGGCAACAGCCGCTGCACCGTCTCCAGCGCCTGCTTGCCGCCGTTATTGCTGGCGATGGCCACGACCTGGTCCGGGG

TCA

>6P1R_2

GGTCGTGGCCATCGCCAGCCACGATGGCGGCAAGCAGGCGCTGGAGACGGTGCAGCGGCTGTTGCCGGTGCTGTGCCAGG

ACCATGGCCTGACCCCGGCGCAGGTGGTGGCCATCGCCAGCCATATTGGCGGCAAGCAGGCGCTGGAGACGGTGCAGCGG

CTGTTGCCGGTGCTGTGCCAGGACCATGGCCTGACCCTGGACCAGGTGGTGGCCATTGCCAGCAATGACGGCAGCAAGCA

GGCGCTGGAGACGGTGCAGCGGCTGTTGCCGGTGCTGTGCCAGGACCATGGCCTGACCCCGGACCAGGTGGTGGCCATCG

CCAGCCACGATGGCGGCAAGCAGGCGCTGGAGACGGTGCAGCGGCTGTTGCCGGTGCTGTGCCAGGACCATGGCCTGACC

CCGAACCAAGTGGTGGCCATCGCCAGCAATATTGGCGGCAAGCAGGCGCTGGAGACGGTGCAGCGGCTGTTGCCGGTGCT

GTGCCAGGCCCATGGCCTGACCCCGGACCAGGTCGTGGCCATCGCCAGCCACGATGGCGGCAAGCAGGCGCTGGAGACGG

TGCAGCGGCTGTTGCCGGTGCTGTGCCAGAACCATGGCCTGACCCCGGACCAGGTCGTGGCCATCGCCAGCAATGGCGGC

GGCAAGCAGGCGCTGGAGACGGTGCAACGGCTGTTGCCGGTGCTGTGCCAGGACCATGGCCTGACCCCGGACCAGGTCGT

GGCCATCGCCAGCAATGGCGGCGGCAAGCAGGCGCTGGAGAGCATTGTTGCCCAGTTATCTC

>6P2F

ACGCGCACATCGTTGCGCTCAGCCAACACCCGGCAGCGTTAGGGACCGTTGCTGTCACGTATCAGGACATAATCAGGGCG

TTGCCAGAGGCGACACACGAAGACATCGTTGGCGTCGGCAAACAGTGGTCCGGCGCACGCGCCCTGGAGGCCTTGCTCAC

GGAGGCGGGGGAGTTGAGAGGTCCGCCGTTACAGTTGGACACAGGCCAACTTCTCAAGATTGCAAAACGTGGCGGCGTGA

CCGCAGTGAAGGCAGTGCATGCATGGCGCAATGCACTGACGGGTGCCCCCCTGAACCTGACCCCGGCACAGGTGGTGGCC

ATCGCCAGCCACGATGGCGGCAATCAGGCGCTGGAGACGGTGCAGCGGCTGTTGCCGGTGCTGTGCCAGGACCATGGCCT

GACCCCGGCGCAGGTGGTGGCCATCGCCAGCCACGATGGCGGCAAGCAGGCTCTGGAGACGGTGCAGCGGCTGTTGCCGG

TGCTGTGCCAGGACCATGGCCTGACCCCGGACCAGGTCGTGGCCATCGCCAGCCACGATGGCGGCAAGCAGGCGCTGGAG

ACGGTGCAGCGGCTGTTGCCGGTGCTGTGCCAGGACCATGGCCTGACCCCGGCGCAGGCGGTGGCCATCGCCAGCAATGG

CGGCGGCAAGCAGGCGCTGGAGACGGTGCAACGGCTGTTGCCGGTGCTGTGCCAGGACCATGGCCTGACCCCGGACCAGG

TGGTGGCCATCGCCAGCAATGGCGGCAAGCAGGCGCTGGAGACGGTGCAGCGGCTGTTGCCGGTGCTGTGCCAGGACCAT

GGCCTGACCCCGGACCAGGTCGTGGCCATTGCCAGCAATAACGGCGGCAAGCAGGCGCTGGAGACGGTGCAGCGGCTGTT

GCCGGTGCTGTGCCAGGACCATGGCCTGACCCCGGACCAGGTCGTGGCCATCGCCAGCCACGA

>6P3F

GGATTGCCGCACGCGCCGGAATTGATCAGAAGAATCAATCGCCGTATTCCCGAACGCACGTCCCATCGCGTTGCCGACTA

CGCGCAAGTGGTTCGCGTGCTGGAGTTTTTCCAGTGCCACTCCCACCCAGCGTACGCATTTGATGAGGCCATGACGCAGT

TCGGGATGAGCAGGAACGGGTTGGTACAGCTCTTTCGCAGAGTGGGCGTCACCGAACTCGAAGCCCGCTGCGGAACGCTC

CCCCCAGCCTCGCAGCGTTGGGACCGTATCCTCCAGGCATCAGGGATGAAAAGGGCCAAACCGTCCCCTACTTCAGCTCA

AACGCCGGATCAGGCGTCTTTGCATGCATTCGCCGATTCGCTGGAGCGTGACCTTGATGCGCCCAGCCCAATGCACGAGG

GAGATCAGACGCGGGCAAGCAGCCGTAAACGGTCCCGATCGGATCGTGCTGTCACCGGCCCCTCCGCACAGCAATCTTTC

GAGGTGCGCGTTCCCGAACAGCGCGATGCGCTGCATTTGCCCCTCAGCTGGAGGGTAAAACGCCCGCGTACCAGGATCGG

GGGCGGCCTCCCGGATCC

>6P2R

CCCTGATTATGTCCTGATACGTGACAGCAACGGTCCCTAACGCTGCCGGGTGTTGGCTGAGCGCAACGATGTGCGCGTGT

GTAAACCCATGACCCACCAGTGCCTCGTGGTGCCGCGCCACTGTCGAACGCACCTTCGGTTTGATCTTCTCTTGCTGCTG

CTGACTGTAGCCGAGCGTGCGTAGATCCACCTGCGCGGCCGGCGAAGCGTCGGAGGGTTGCGCCGCACGCCGTCGCGGGG

CCGGCTTGGCGCGCGGCGGCCGCGCGGCAGTGACAGCGACACGCACGGTGGGTGGCGGGTCATCGGCTGCACGCAGACCC

GATTGCGCCTCATCCCATTCTGCTGGGGCAGCCGCTGTATGCGGCGTGCCGACGGCAGGCATCGAATCAAGAAGCGATGT

ATCAAGAAGCGACGGATCGAACTGACGGAGCAGATCGCTGAAGCTGCCCGCCGAGAACGCAGGCGAGGGCGCAGGGGGAG

ATGGCAGCCGGGTCCGGGACATCGTCCGCCGAGCGGGCAAGCCATCCAGGGGGCCGCCAGCAGGCGGAGCCCCCCCCCGA

TCTGCAGTCGGCTGAACCCTATCCGGTTGGGGTCCGGGCAGAAGCTCGCGGGCAGGACTTGGCGTGCGCGAACGAATGGG

ATCC

>6P3R

CTTCGAGTTCGGTGACGCCCACTCTGCGAAAGAGCTGTACCAACCCGTTCCTGCTCATCCCGAACTGCGTCATGGCCTCA

TCAAATGCGTACGCTGGGTGGGAGTGGCACTGGAAAAACTCCAGCACGCGAACCACTTGCGCGTAGTCGGCAACGCGATG

GGACGTGCGTTCGGGAATACGGCGATTGATTCTTCTGATCAATTCCGGCGCGTGCGGCAATCCCTTTTTCACTGCATCCA

GGGCAGGACGTCCGCCGAGGCAGGCCAAGGCGACGAGGTGGTCGTTGGTCAACGCGGCCAACGCCGGATCAGGGCGAGAT

AACTGGGCAACAATGCTCTCCAGCGCCTGCTTGCCGCCGCCATTGCTGGCGATGGCCACGACCTGGTCCGGGGTCAGGCC

ATGGTCCTGGCACAGCACCGGCAACAGCCGTTGCACCGTCTCCAGCGCCTGCTTGCCGCCGCCATTGCTGGCGATGGCCA

CGACCTGGTCCGGGGTCAGGCCATGGTTCTGGCACAGCACCGGCAACAGCCGCTGCACCGTCTCCAGCGCCTGCTTGCCG

CCATCGTGGCTGGCGATGGCCACGACCTGGTCCGGGGTCAGGCCATGGGCCTGGCACAGCACCGGCAACAGCCGCTGCAC

CGTCTCCAGCGCCTGCTTGCCGCCAATATTGCTGGCGATGGCCACCACTTGGTTCGGGGTCAGGCCATGGTCCTGGCACA

GCACCGGCAACAGCCGCTGCACCGTCTCCAGCGCCTGCTTGCCGCCATCGTGGCTGGCGATGGCCACCACCTGGTCCGGG

GTCAGGCCATGGTCCTGGCACAGCACCGGCAACAGCCGCTGCACCGTCTCCAGCGCCTGCTTGCTGCCGTCATTGCTGGC

AATGGCCACCACCTGGTCCAGGGTCAGGCCATGGTCCTGGCACAGCACCGGCAACAGCC

> pCC2FOS-MscI-7

CCATCGCCAGCAATGGCGGCAAGCAGGCGCTGGAGACGGTGCAGCGGCTGTTGCCGGTGCTGTGCCAGGACCATGGCCTG

ACCCTGGCGCAGGTGGTGGCCATCGCCAGCAATATTGGCGGCAAGCAGGCGCTGGAGACGGTGCAGCGGCTGTTGCCGGT

GCTGTGCCAGGACCATGGCCTGACCCCGGACCAGGTCGTGGCCATCGCCAGTAATATTGGCGGCAAGCAGGCGCTGGAGA

CGGTGCAGCGGCTGTTGCCGGTGCTGTGCCAGGACCATGGCCTGACCCCGGACCAGGTCGTGGCCATCGCCAGCAATAAC

GGCGGCAAGCAGGCGCTGGAGACGGTGCAGCGGCTGTTGCCGGTGCTGTGCCAGGACCATGGCCTGACCCCGGACCAGGT

CGTGGCCATCGCCAGCCACGATGGCGGCAAGCAGGCGCTGGAGACGGTGCAGCGGCTGTTGCCGGTGCTGTGCCAGGACC

ATGGCCTGACCCCGGCGCAGGTGGTGGCCATCGCCAGCCATATTGGCGGCAAGCAGGCGCTGGAGACGGTGCAGCGGCTG

TTGCCGGTGCTGTGCCAGGACCATGGCCTGACCCTGGACCAGGTGGTGGCCATTGCCAGCAATGACGGCAGCAAGCAGGC

GCTGGAGACGGTGCAGCGGCTGTTGCCGGTGCTGTGCCAGGACCATGGCCTGACCCCGGACCAGGTGGTGGCCATCGCCA

GCCACGATGGCGGCAAGCAGGCGCTGGAGACGGTGCAGCGGCTGTTGCCGGTGCTGTGCCAGGACCATGGCCTGACCCCG

AACCAAGTGGTGGCCATCGCCAGCAATATTGGCGGCAAGCAGGCGCTGGAGACGGTGCAGCGGCTGTTGCCGGTGCTGTG

CCAGGCCCATGGC

> pCC2FOS-MscI-8

CCATTGCCAGCAATAACGGCGGCAAGCAGGCGCTGGAGACGGTGCAGCGGCTGTTGCCGGTGCTGTGCCAGGACCATGGC

CTGACCCCGGACCAGGTCGTGGCCATCGCCAGCCACGATGGCGGCAAGCAGGCGCTGGAGACGGTGCAGCGGCTGTTGCC

GGTGCTGTGCCAGGACCATGGCCTGACCCCGGACCAGGTCGTGGCCATCGCCAGCCACGATGGCGGCAAGCAGGCGCTGG

AGACGGTGCAGCGGCTGTTGCCGGTGCTGTGCCAGGACCATGGCCTGACCCCGGACCAGGTGGTGGCCATCGCCAGCAAT

GGCGGCAAGCAGGCGCTGGAGACGGTGCAGCGGCTGTTGCCGGTGCTGTGCCAGGACCATGGCCTGACCCTGGCGCAGGT

GGTGGCCATCGCCAGCAATATTGGCGGCAAGCAGGCGCTGGAGACGGTGCAGCGGCTGTTGCCGGTGCTGTGCCAGGACC

ATGGCCTGACCCCGGACCAGGTCGTGGCCATCGCCAGTAATATTGGCGGCAAGCAGGCGCTGGAGACGGTGCAGCGGCTG

TTGCCGGTGCTGTGCCAGGACCATGGCCTGACCCCGGACCAGGTCGTGGCCATCGCCAGCAATAACGGCGGCAAGCAGGC

GCTGGAGACGGTGCAGCGGCTGTTGCCGGTGCTGTGCCAGGACCATGGCCTGACCCCGGACCAGGTCGTGGCCATCGCCA

GCCACGATGGCGGCAAGCAGGCGCTGGAGACGGTGCAGCGGCTGTTGCCGGTGCTGTGCCAGGACCATGGCCTGACCCCG

GCGCAGGTGGTGGCCATCGCCAGCCATATTGGCGGCAAGCAGGCGCTGGAGACGGTGCAGCGGCTGTTGCCGGT

>M13F

GGATCCCATTCGTTCGCGCACGCCAAGTCCTGCCCGCGAGCTTCTGCCCGGACCCCAACCGGATAGGGTTCAGCCGACTG

CAGATCGGGGGGGGGCTCCGCCTGCTGGCGGCCCCCTGGATGGCTTGCCCGCTCGGCGGACGATGTCCCGGACCCGGCTG

CCATCTCCCCCTGCGCCCTCGCCTGCGTTCTCGGCGGGCAGCTTCAGCGATCTGCTCCGTCAGTTCGATCCGTCGCTTCT

TGATACATCGCTTCTTGATTCGATGCCTGCCGTCGGCACGCCGCATACAGCGGCTGCCCCAGCAGAATGGGATGAGGCGC

AATCGGGTCTGCGTGCAGCCGATGACCCGCCACCCACCGTGCGTGTCGCTGTCACTGCCGCGCGGCCGCCGCGCGCCAAG

CCGGCCCCGCGACGGCGTGCGGCGCAACCCTCCGACGCTTCGCCGGCCGCGCAGGTGGATCTACGCACGCTCGGCTACAG

TCAGCAGCAGCAAGAGAAGATCAAACCGAAGGTGCGTTCGACAGTGGCGCGGCACCACGAGGCACTGGTGGGTCATGGGT

TTACACACGCGCACATCGTTGCGCTCAGCCAACACCCGGCAGCGTTAGGGACCGTTGCTGTCACGTATCAGGACATAATC

AGGGCGTTGCCAGAGGCGACACACGAAGACATCGTTGGCGTCGGCAAACAGTGGTCCGGCGCACGCGCCCTGGAGGCCTT

GCTCACGGAGGCGGGGGAGTTGAGAGGTCCGCCGTTACAGTTGGACACAGGCCAACTTCTCAAGATTGCAAAACGTGGCG

GCGTGACCGCAGTGAAGGCAGTGCATGCATGGCGCAATGCACTGACGGGTGCCCCCCTGAACCTGACCCCGGCACAGGTG

GTGGCCATCGCCAGCCACGATGGCGGCAATCAG

>M13R

CGCTGGAGACGGTGCAGCGGCTGTTGCCGGTGCTGTGCCAGAACCATGGCCTGACCCCGGACCAGGTCGTGGCCATCGCC

AGCAATGGCGGCGGCAAGCAGGCGCTGGAGACGGTGCAACGGCTGTTGCCGGTGCTGTGCCAGGACCATGGCCTGACCCC

GGACCAGGTCGTGGCCATCGCCAGCAATGGCGGCGGCAAGCAGGCGCTGGAGAGCATTGTTGCCCAGTTATCTCGCCCTG

ATCCGGCGTTGGCCGCGTTGACCAACGACCACCTCGTCGCCTTGGCCTGCCTCGGCGGACGTCCTGCCCTGGATGCAGTG

AAAAAGGGATTGCCGCACGCGCCGGAATTGATCAGAAGAATCAATCGCCGTATTCCCGAACGCACGTCCCATCGCGTTGC

CGACTACGCGCAAGTGGTTCGCGTGCTGGAGTTTTTCCAGTGCCACTCCCACCCAGCGTACGCATTTGATGAGGCCATGA

CGCAGTTCGGGATGAGCAGGAACGGGTTGGTACAGCTCTTTCGCAGAGTGGGCGTCACCGAACTCGAAGCCCGCTGCGGA

ACGCTCCCCCCAGCCTCGCAGCGTTGGGACCGTATCCTCCAGGCATCAGGGATGAAAAGGGCCAAACCGTCCCCTACTTC

AGCTCAAACGCCGGATCAGGCGTCTTTGCATGCATTCGCCGATTCGCTGGAGCGTGACCTTGATGCGCCCAGCCCAATGC

ACGAGGGAGATCAGACGCGGGCAAGCAGCCGTAAACGGTCCCGATCGGATCGTGCTGTCACCGGCCCCTCCGCACAGCAA

TCTTTCGAGGTGCGCGTTCCCGAACAGCGCGATGCGCTGCATTTGCCCCTCAGCTGGAGGGTAAAACGCCCGCGTACCAG

GATCGGGGGCGGCCTCCCGGATCC

**pTAL*_Bam_*_HI_-11**

> pCC2FOS-MscI-1

CCATCGCCAACAATAACGGCGGCAAGCAGGCGCTGGAGACGGTGCAACGGCTGTTGCCGGTGCTGTGCCAGGACCATGGC

CTGACCCCGGACCAGGTGGTGGCCATCGCCAACAATAACGGCGGCAAGCAGGCGCTGGAGACGGTGCAGCGGCTGTTGCC

GGTGCTGTGCCAGGCCCATGGCCTGCCCCCGGACCAGGTGGTGGCCATCGCCAGCAATATTGGCGGCAAGCAGGCGCTGG

AGACGGTGCAGCGGCTGTTGCCGGTGCTGTGCCAGGACCATGGCCTGACCCCGGACCAAGTGGTGGCCATCGCCAACAAT

AACGGCGGCAAGCAGGCGCTGGAGACGGTGCAGCGGCTGTTGCCGGTGCTGTGCCAGGACCATGGCCTGACCCCGGACCA

GGTGGTGGCCATCGCCAGCCACGATGGCGGCAAGCAGGCGCTGGAGACGGTGCAGCGGCTGTTGCCGGTGCTGTGCCAGG

ACCATGGCCTGACCCCGGACCAGGTGGTGGCCATCGCCAGCCATGGCGGCGGCAAGCAGGCGCTGGAGACGGTGCAGCGG

CTGTTGCCGGTGCTGTGCCAGGACCATGGCCTGAGTCCGGACCAGGTGGTGGCCATCGCCAGCCACGATGGCGGCAAGCA

GGCGCTGGAGACGGTGCAGCGGCTGTTGCCGGTGCTGTGCCAGGACCATGGCCTGACCCTGGACCAGGTGGTGGCCATCG

CCAGCCACGATGGCGGCAAGCAGGCGCTGGAGACGGTGCAACGGCTGTTGCCGGTGCTGTGCCAGGACCATGGCCTGACC

CCGGCCCAGGTGGTGGCCATCGCCAACAATAACGGCGGCAAGCAGGCGCTGGAGACGGTGCAGCGGCTGTTGCCGGTGCT

GTGCCA

> pCC2FOS-MscI-2

CCATCGCCAGCAATGGCGGCGGCAAGCAGGCGCTGGAGACGGTGCAGCGGCTGTTGCCGGTGCTGTGCCAGGCCCATGGC

CTGAACCCGGACCAGGTGGTGGCCATCGCCAGCAATAGTGGCGGCAAGCAGGCGCTGGAGACGGTGCAGCGGCTGTTGCC

GGTGCTGTGCCAGGCCCATGGCCTGAACCCGGACCAAGTGGTGGCCATCGCCAGCAATAACGGCGGCAAGCAGGCGCTGG

AGACGGTGCAGCGGCTGTTGCCGGTGCTGTGCCAGGACCATGGCCTGAGTCCGGACCAGGTGGTGGCCATCGCCAGCCAC

GATGGCGGCAAGCAGGCGCTGGAGACGGTGCAGCGGCTGTTGCCGGTGCTGTGCCAGGACCATGGCCTGACCCCGGACCA

GGTCGTGGCCATCGCCAGCAATGGCGGCAAGCAGGCGCTGGAGACGGTGCAGCGCCTGTTGCCGGTGCTGTGCCAGGACC

ATGGCCTGACCCCGGACCAGGTGGTGGCCATCGCCAACAATAACGGCGGCAAGCAGGCGCTGGAGACGGTGCAACGGCTG

TTGCCGGTGCTGTGCCAGGACCATGGCCTGACCCCGGACCAGGTGGTGGCCATCGCCAACAATAACGGCGGCAAGCAGGC

GCTGGAGACGGTGCAGCGGCTGTTGCCGGTGCTGTGCCAGGCCCATGGCCTGCCCCCGGACCAGGTGGTGGCCATCGCCA

GCAATATTGGCGGCAAGCAGGCGCTGGAGACGGTGCAGCGGCTGTTGCCGGTGCTGTGCCAGGACCATGGCCTGACCCCG

GACCAAGTGGTGGCCATCGCCAACAATAACGGCGGCAAGCAGGCGCTGGAGACGGTGCAGCGGCTGTTGCCGGTGCTG

> pCC2FOS-MscI-3

CCATCGCCAGCAATGGCGGCAAGCAGGCGCTGGAGACGGTGCAGCGCCTGTTGCCGGTGCTGTGCCAGGACCATGGCCTG

ACCCCGGACCAGGTGGTGGCCATCGCCAACAATAACGGCGGCAAGCAGGCGCTGGAGACGGTGCAACGGCTGTTGCCGGT

GCTGTGCCAGGACCATGGCCTGACCCCGGACCAGGTGGTGGCCATCGCCAACAATAACGGCGGCAAGCAGGCGCTGGAGA

CGGTGCAGCGGCTGTTGCCGGTGCTGTGCCAGGCCCATGGCCTGCCCCCGGACCAGGTGGTGGCCATCGCCAGCAATATT

GGCGGCAAGCAGGCGCTGGAGACGGTGCAGCGGCTGTTGCCGGTGCTGTGCCAGGACCATGGCCTGACCCCGGACCAAGT

GGTGGCCATCGCCAACAATAACGGCGGCAAGCAGGCGCTGGAGACGGTGCAGCGGCTGTTGCCGGTGCTGTGCCAGGACC

ATGGCCTGACCCCGGACCAGGTGGTGGCCATCGCCAGCCACGATGGCGGCAAGCAGGCGCTGGAGACGGTGCAGCGGCTG

TTGCCGGTGCTGTGCCAGGACCATGGCCTGACCCCGGACCAGGTGGTGGCCATCGCCAGCCATGGCGGCGGCAAGCAGGC

GCTGGAGACGGTGCAGCGGCTGTTGCCGGTGCTGTGCCAGGACCATGGCCTGAGTCCGGACCAGGTGGTGGCCATCGCCA

GCCACGATGGCGGCAAGCAGGCGCTGGAGACGGTGCAGCGGCTGTTGCCGGTGC

> pCC2FOS-MscI-4

CCATCGCCAGCAATGGCGGCAAGCAGGCGCTGGAGACGGTGCAGCGCCTGTTGCCGGTGCTGTGCCAGGACCATGGCCTG

ACCCCGGACCAGGTGGTGGCCATCGCCAACAATAACGGCGGCAAGCAGGCGCTGGAGACGGTGCAACGGCTGTTGCCGGT

GCTGTGCCAGGACCATGGCCTGACCCCGGACCAGGTGGTGGCCATCGCCAACAATAACGGCGGCAAGCAGGCGCTGGAGA

CGGTGCAGCGGCTGTTGCCGGTGCTGTGCCAGGCCCATGGCCTGCCCCCGGACCAGGTGGTGGCCATCGCCAGCAATATT

GGCGGCAAGCAGGCGCTGGAGACGGTGCAGCGGCTGTTGCCGGTGCTGTGCCAGGACCATGGCCTGACCCCGGACCAAGT

GGTGGCCATCGCCAACAATAACGGCGGCAAGCAGGCGCTGGAGACGGTGCAGCGGCTGTTGCCGGTGCTGTGCCAGGACC

ATGGCCTGACCCCGGACCAGGTGGTGGCCATCGCCAGCCACGATGGCGGCAAGCAGGCGCTGGAGACGGTGCAGCGGCTG

TTGCCGGTGCTGTGCCAGGACCATGGCCTGACCCCGGACCAGGTGGTGGCCATCGCCAGCCATGGCGGCGGCAAGCAGGC

GCTGGAGACGGTGCAGCGGCTGTTGCCGGTGCTGTGCCAGGACCATGGCCTGAGTCCGGACCAGGTGGTGGCCATCGCCA

GCCACGATGGCGGCAAGCAGGCGCTGGAGACGGTGCAGCGGCTGTTGCCGGTGCTGTGCCAGGACCATGGCCTGACCCTG

GACCAGGTGGTGGCCATCGCCAGCCACGATGGCGGCAAGCAGGCGCTGGAGACGGTGCAACGGCTGTTGCCGGTGCTGTG

CCAGGACCA

> pCC2FOS-MscI-5

CCATCGCCAGCAATGGCGGCAAGCAGGCGCTGGAGACGGTGCAGCGCCTGTTGCCGGTGCTGTGCCAGGACCATGGCCTG

ACCCCGGACCAGGTGGTGGCCATCGCCAACAATAACGGCGGCAAGCAGGCGCTGGAGACGGTGCAACGGCTGTTGCCGGT

GCTGTGCCAGGACCATGGCCTGACCCCGGACCAGGTGGTGGCCATCGCCAACAATAACGGCGGCAAGCAGGCGCTGGAGA

CGGTGCAGCGGCTGTTGCCGGTGCTGTGCCAGGCCCATGGCCTGCCCCCGGACCAGGTGGTGGCCATCGCCAGCAATATT

GGCGGCAAGCAGGCGCTGGAGACGGTGCAGCGGCTGTTGCCGGTGCTGTGCCAGGACCATGGCCTGACCCCGGACCAAGT

GGTGGCCATCGCCAACAATAACGGCGGCAAGCAGGCGCTGGAGACGGTGCAGCGGCTGTTGCCGGTGCTGTGCCAGGACC

ATGGCCTGACCCCGGACCAGGTGGTGGCCATCGCCAGCCACGATGGCGGCAAGCAGGCGCTGGAGACGGTGCAGCGGCTG

TTGCCGGTGCTGTGCCAGGACCATGGCCTGACCCCGGACCAGGTGGTGGCCATCGCCAGCCATGGCGGCGGCAAGCAGGC

GCTGGAGACGGTGCAGCGGCTGTTGCCGGTGCTGTGCCAGGACCATGGCCTGAGTCCGGACCAGGTGGTGGCCATCGCCA

GCCACGATGGCGGCAAGCAGGCGCTGGAGACGGTGCAGCGGCTGTTGCCGGTGCTGTGCCAGGACCATGGCCTGACCCTG

GACCAGGTGGTGGCCATCGCCAGCCACGATGGCGGCAAGCAGGCGCTGGAGACGGTGCAACGGCTGTTGCCGGTGCTGTG

CCAGGACCA

>11P1F_1

AGGCGGGGGAGTTGAGAGGTCCGCCGTTACAGTTGGACACAGGCCAACTTCTCAAGATTGCAAAACGTGGCGGCGTGACC

GCAGTGGAGGCAGTGCATGCATGGCGCAATGCACTGACGGGTGCCCCCCTGAACCTGACCCCGGACCAAGTGGTGGCCAT

CGCCAGCAATATTGGCGGCAAGCAGGCGCTGGAGACGGTGCAGCGCCTGTTGCCGGTGCTGTGCCAGGCCCATGGCCTGA

ACCCGGACCAAGTGGTGGCCATCGCCAGCAATAGTGGCGGCAAGCAGGCGCTGGAGACGGTGCAGCGGCTGTTGCCGGTG

CTGTGCCAGGACCATGGCCTGACCCCGGACCAAGTGGTGGCCATCGCCAGCCACGATGGCGGCAAGCAGGCGCTGGAGAC

GGTGCAGCGGCTGTTGCCGGTGCTGTGCCAGGACCATGGCCTGACCCCAGACCAGGTCGTGGCCATCGCCAGCAATGGCG

GCGGCAAGCAGGCGCTGGAGACGGTGCAGCGGCTGTTGCCGGTGCTGTGCCAGGCCCATGGCCTGAACCCGGACCAGGTG

GTGGCCATCGCCAGCAATAGTGGCGGCAAGCAGGCGCTGGAGACGGTGCAGCGGCTGTTGCCGGTGCTGTGCCAGGCCCA

TGGCCTGAACCCGGACCAAGTGGTGGCCATCGCCAGCAATAACGGCGGCAAGCAGGCGCTGGAGACGGTGCAGCGGCTGT

TGCCGGTGCTGTGCCAGGACCATGGCCTGAGTCCGGACCAGGTGGTGGCCATCGCCAGCCACGATGGCGGCAAGCAGGCG

CTGGAGACGGTGCAGCGGCTGTTGCCGGTGCTGTGCCAGGACCATGGCCTGACCCCGGACCAGGTCGTGGCCATCGCCAG

CAATGGCGGCAAGCAGGCGCTGGAGACGGTGCAGCGCCTGTTGCCGGTGCTGTGCCAGGACCATGGCCTGACCCCGGACC

AGGTGGTGGCCATCGCCAACAAT

>11P1F_2

GAAGGCGGGGGAGTTGAGAGGTCCGCCGTTACAGTTGGACACAGGCCAACTTCTCAAGATTGCAAAACGTGGCGGCGTGA

CCGCAGTGGAGGCAGTGCATGCATGGCGCAATGCACTGACGGGTGCCCCCCTGAACCTGACCCCGGACCAAGTGGTGGCC

ATCGCCAGCAATATTGGCGGCAAGCAGGCGCTGGAGACGGTGCAGCGCCTGTTGCCGGTGCTGTGCCAGGCCCATGGCCT

GAACCCGGACCAAGTGGTGGCCATCGCCAGCAATAGTGGCGGCAAGCAGGCGCTGGAGACGGTGCAGCGGCTGTTGCCGG

TGCTGTGCCAGGACCATGGCCTGACCCCGGACCAAGTGGTGGCCATCGCCAGCCACGATGGCGGCAAGCAGGCGCTGGAG

ACGGTGCAGCGGCTGTTGCCGGTGCTGTGCCAGGACCATGGCCTGACCCCAGACCAGGTCGTGGCCATCGCCAGCAATGG

CGGCGGCAAGCAGGCGCTGGAGACGGTGCAGCGGCTGTTGCCGGTGCTGTGCCAGGCCCATGGCCTGAACCCGGACCAGG

TGGTGGCCATCGCCAGCAATAGTGGCGGCAAGCAGGCGCTGGAGACGGTGCAGCGGCTGTTGCCGGTGCTGTGCCAGGCC

CATGGCCTGAACCCGGACCAAGTGGTGGCCATCGCCAGCAATAACGGCGGCAAGCAGGCGCTGGAGACGGTGCAGCGGCT

GTTGCCGGTGCTGTGCCAGGACCATGGCCTGAGTCCGGACCAGGTGGTGGCCATCGCCAGCCACGATGGCGGCAAGCAGG

CGCTGGAGACGGTGCAGCGGCTGTTGCCGGTGCTGTGCCAGGACCATGGCCTGACCCCGGACCAGGTCGTGGCCATCGCC

AGCAATGGCGGCAAGCAGGCGCTGGAGACGGTGCAGCGCCTGTTGCCGGTGCTGTGCCAGGACCATGGCCTGACCCCGGA

CCAGGTGGTGGCCATCGC

>11P1R_1

CAATGCTCTCCAGCGCCTGCTTGCCGCCGCCATTGCTGGCGATGGCCACCACCTGGTCCGGGGTCAGGCCATGGTCCTGG

CACAGCACCGGCAACAGCCGCTGCACCGTCTCCAGCGCCTGCTTGCCGCCGTTATTGTTGGCGATGGCCACCACCTGGGC

CGGGGTCAGGCCATGGTCCTGGCACAGCACCGGCAACAGCCGTTGCACCGTCTCCAGCGCCTGCTTGCCGCCATCGTGGC

TGGCGATGGCCACCACCTGGTCCAGGGTCAGGCCATGGTCCTGGCACAGCACCGGCAACAGCCGCTGCACCGTCTCCAGC

GCCTGCTTGCCGCCATCGTGGCTGGCGATGGCCACCACCTGGTCCGGACTCAGGCCATGGTCCTGGCACAGCACCGGCAA

CAGCCGCTGCACCGTCTCCAGCGCCTGCTTGCCGCCGCCATGGCTGGCGATGGCCACCACCTGGTCCGGGGTCAGGCCAT

GGTCCTGGCACAGCACCGGCAACAGCCGCTGCACCGTCTCCAGCGCCTGCTTGCCGCCATCGTGGCTGGCGATGGCCACC

ACCTGGTCCGGGGTCAGGCCATGGTCCTGGCACAGCACCGGCAACAGCCGCTGCACCGTCTCCAGCGCCTGCTTGCCGCC

GTTATTGTTGGCGATGGCCACCACTTGGTCCGGGGTCAGGCCATGGTCCTGGCACAGCACCGGCAACAGCCGCTGCACCG

TCTCCAGCGCCTGCTTGCCGCCAATATTGCTGGCGATGGCCACCACCTGGTCCGGGGGCAGGCCATGGGCCTGGCACAGC

ACCGGCAACAGCCGCTGCACCGTCTCCAGCGCCTGCTTGCCGCCGTTATTGTTGGCGATGGCCACCACCTGGTCCGGGGT

CAGGCCATGGTCCTGGCACAGCACCGGCAACAGCCGTTGCACCGTC

>11P1R_2

CCAGGACCATGGCCTGACCCCGGACCAGGTGGTGGCCATCGCCAACAATAACGGCGGCAAGCAGGCGCTGGAGACGGTGC

AACGGCTGTTGCCGGTGCTGTGCCAGGACCATGGCCTGACCCCGGACCAGGTGGTGGCCATCGCCAACAATAACGGCGGC

AAGCAGGCGCTGGAGACGGTGCAGCGGCTGTTGCCGGTGCTGTGCCAGGCCCATGGCCTGCCCCCGGACCAGGTGGTGGC

CATCGCCAGCAATATTGGCGGCAAGCAGGCGCTGGAGACGGTGCAGCGGCTGTTGCCGGTGCTGTGCCAGGACCATGGCC

TGACCCCGGACCAAGTGGTGGCCATCGCCAACAATAACGGCGGCAAGCAGGCGCTGGAGACGGTGCAGCGGCTGTTGCCG

GTGCTGTGCCAGGACCATGGCCTGACCCCGGACCAGGTGGTGGCCATCGCCAGCCACGATGGCGGCAAGCAGGCGCTGGA

GACGGTGCAGCGGCTGTTGCCGGTGCTGTGCCAGGACCATGGCCTGACCCCGGACCAGGTGGTGGCCATCGCCAGCCATG

GCGGCGGCAAGCAGGCGCTGGAGACGGTGCAGCGGCTGTTGCCGGTGCTGTGCCAGGACCATGGCCTGAGTCCGGACCAG

GTGGTGGCCATCGCCAGCCACGATGGCGGCAAGCAGGCGCTGGAGACGGTGCAGCGGCTGTTGCCGGTGCTGTGCCAGGA

CCATGGCCTGACCCTGGACCAGGTGGTGGCCATCGCCAGCCACGATGGCGGCAAGCAGGCGCTGGAGACGGTGCAACGGC

TGTTGCCGGTGCTGTGCCAGGACCATGGCCTGACCCCGGCCCAGGTGGTGGCCATCGCCAACAATAACGGCGGCAAGCAG

GCGCTGGAGACGGTGCAGCGGCTGTTGCCGGTGCTGTGCCAGGACCATGGCCTGACCCCGGACCAGGTGGTGGCCATCGC

CAGCAATGGCGGCGGCAAGCAGGCGCTGGAGAGCATTGTTGCCCAGTTATAT

>11P2F

CGCGCACATCGTTGCGCTCAGCCAACACCCGGCAGCGTTAGGGACCGTCGCTGTCAAGTATCAGCACATAATCACGGCGT

TGCCAGAGGCGACACACGAAGACATCGTTGGCGTCGGCAAACAGTGGTCCGGCGCACGCGCCCTGGAGGCCTTGCTCACG

AAGGCGGGGGAGTTGAGAGGTCCGCCGTTACAGTTGGACACAGGCCAACTTCTCAAGATTGCAAAACGTGGCGGCGTGAC

CGCAGTGGAGGCAGTGCATGCATGGCGCAATGCACTGACGGGTGCCCCCCTGAACCTGACCCCGGACCAAGTGGTGGCCA

TCGCCAGCAATATTGGCGGCAAGCAGGCGCTGGAGACGGTGCAGCGCCTGTTGCCGGTGCTGTGCCAGGCCCATGGCCTG

AACCCGGACCAAGTGGTGGCCATCGCCAGCAATAGTGGCGGCAAGCAGGCGCTGGAGACGGTGCAGCGGCTGTTGCCGGT

GCTGTGCCAGGACCATGGCCTGACCCCGGACCAAGTGGTGGCCATCGCCAGCCACGATGGCGGCAAGCAGGCGCTGGAGA

CGGTGCAGCGGCTGTTGCCGGTGCTGTGCCAGGACCATGGCCTGACCCCAGACCAGGTCGTGGCCATCGCCAGCAATGGC

GGCGGCAAGCAGGCGCTGGAGACGGTGCAGCGGCTGTTGCCGGTGCTGTGCCAGGCCCATGGCCTGAACCCGGACCAGGT

GGTGGCCATCGCCAGCAATAGTGGCGGCAAGCAGGCGCTGGAGACGGTGCAGCGGCTGTTGCCGGTGCTGTGCCAGGCCC

ATGGCCTGAACCCGGACCAAGTGGTGGCCATCGCCAGCAATAACGGCGGCAAGCAGGCGCTGGAGACGGTGCAGCGGCTG

TTGCCGGTGCTGTGCCAGGACCATGGCCTGAGTCCGGACCAGGTGGTGGCCATCGCCAGCCACGATGGCGGCAAGCAGGC

GCTGGAGACGGTGCA

>11P2R

TATGTGCTGATACTTGACAGCGACGGTCCCTAACGCTGCCGGGTGTTGGCTGAGCGCAACGATGTGCGCGTGTGTAAACC

CATGGCCCACCAGTGCCTCGTGGTGCTGCGCCACTGTCGAACGCACCTTCGGTTTGATCTTCTCTTGCTGCTGCTGACTG

TAGCCGAGCGTGCGTAGATCCACCTGCGCGGCCGGCGAAGCGTCGGAGGGTTGCGCCGCACGCCGTCGCGGGGCCGGCTT

GGCGCGCGGCGGCCGCGCGGCAGTGACAGCGACACGCACGGTGGGTGGCGGGTCATCGGCTGCACGCAGACCCGATTGCA

CCTCATCCCACTCTGCTGGGGCAGCCGCTGTATGCGGCGTGCCGACGGCAGGCATCGAATCAAGAAGCGATGTATCAAGA

AGCGACGGATCGAACTGACGGAGCAGATCGCTGAAGCTGCCCGCCGAGAACGCAGGCGAGGGCGCAGGGGGAGATGGCAG

CCGGGTCCGGGACATCGTCCGCCGAGCGGGCAAGCCATCCAGGGGGCCGCCAGCAGGCGGAGCCCCCCCCCGATCTGCAG

TCGGCTGAACCCTATCCGGTTGGGGTCCGGGCAGAAGCTCGCGGGCAGGACTTGGCGTGCGCGAACGAATGGGATCC

>11P3F

TGCCGCACGCGCCGGAATTGATCAGAAGAATCAATCGCCGTATTCCCGAACGCACGTCCCATCGCGTTGCCGACCTCGCG

CACGTGGTGCGCGTGCTTGGTTTTTTCCAGAGCCACTCCCACCCAGCGCAAGCATTCGATGACGCCATGACGCAGTTCGG

GATGAGCAGGCACGGGTTGGTACAGCTCTTTCGCAGAGTGGGCGTCACCGAATTCGAAGCCCGCTGCGGAACGCTCCCCC

CAGCCTCGCAGCGTTGGGACCGTATCCTCCAGGCATCAGGGATGAAAAGGGCCAAACCGTCCCCTACTTCAGCTCAAACA

CCGGATCAGGCGTCTTTGCATGCATTCGCCGATTCGCTGGAGCGTGACCTTGATGCGCCCGGCCCAATGCACGAGGGAGA

TCAGACGCGGGCAAGCAGCCGTAAACGGTCCCGATCGGATCGTGCTGTCACCGGCCCCTCCGCACAGCAATCTTTCGAGG

TACGCGTTCCCGAACAGCGCGATGCTCTGCATTTGCCCCTCAGCTGGAGGGTAAAACGCCCGCGTACCAGGATCGGGGGC

GGCCTCCCGGATCC

>11P3R

CGGGCTTCGAATTCGGTGACGCCCACTCTGCGAAAGAGCTGTACCAACCCGTGCCTGCTCATCCCGAACTGCGTCATGGC

GTCATCGAATGCTTGCGCTGGGTGGGAGTGGCTCTGGAAAAAACCAAGCACGCGCACCACGTGCGCGAGGTCGGCAACGC

GATGGGACGTGCGTTCGGGAATACGGCGATTGATTCTTCTGATCAATTCCGGCGCGTGCGGCAATCCCTTTTTCACTGCA

TCCAGGGCAGGACGTCCGCCGAGGCAGGCCAAGGCGACGAGGTGGTCGTTGGTCAACGCGGCCAACGCCGGATCAGGGCG

ATATAACTGGGCAACAATGCTCTCCAGCGCCTGCTTGCCGCCGCCATTGCTGGCGATGGCCACCACCTGGTCCGGGGTCA

GGCCATGGTCCTGGCACAGCACCGGCAACAGCCGCTGCACCGTCTCCAGCGCCTGCTTGCCGCCGTTATTGTTGGCGATG

GCCACCACCTGGGCCGGGGTCAGGCCATGGTCCTGGCACAGCACCGGCAACAGCCGTTGCACCGTCTCCAGCGCCTGCTT

GCCGCCATCGTGGCTGGCGATGGCCACCACCTGGTCCAGGGTCAGGCCATGGTCCTGGCACAGCACCGGCAACAGCCGCT

GCACCGTCTCCAGCGCCTGCTTGCCGCCATCGTGGCTGGCGATGGCCACCACCTGGTCCGGACTCAGGCCATGGTCCTGG

CACAGCACCGGCAACAGCCGCTGCACCGTCTCCAGCGCCTGCTTGCCGCCGCCATGGCTGGCGATGGCCACCACCTGGTC

CGGGGTCAGGCCATGGTCCTGGCACAGCACCGGCAACAGCCGCTGCACCGTCTCCAGCGCCTGCTTGCCGCCATCGTGGC

TGGCGATGGCCACCACCTGGTCCGGGGTCAGGCCATGGTCCTGGCACAGCACCGGCAACAGCCGCTGCACCGTCTCCAGC

GCCTGCTTGCCGCCGTTATTGTTG

>M13F

GGATCCCATTCGTTCGCGCACGCCAAGTCCTGCCCGCGAGCTTCTGCCCGGACCCCAACCGGATAGGGTTCAGCCGACTG

CAGATCGGGGGGGGGCTCCGCCTGCTGGCGGCCCCCTGGATGGCTTGCCCGCTCGGCGGACGATGTCCCGGACCCGGCTG

CCATCTCCCCCTGCGCCCTCGCCTGCGTTCTCGGCGGGCAGCTTCAGCGATCTGCTCCGTCAGTTCGATCCGTCGCTTCT

TGATACATCGCTTCTTGATTCGATGCCTGCCGTCGGCACGCCGCATACAGCGGCTGCCCCAGCAGAGTGGGATGAGGTGC

AATCGGGTCTGCGTGCAGCCGATGACCCGCCACCCACCGTGCGTGTCGCTGTCACTGCCGCGCGGCCGCCGCGCGCCAAG

CCGGCCCCGCGACGGCGTGCGGCGCAACCCTCCGACGCTTCGCCGGCCGCGCAGGTGGATCTACGCACGCTCGGCTACAG

TCAGCAGCAGCAAGAGAAGATCAAACCGAAGGTGCGTTCGACAGTGGCGCAGCACCACGAGGCACTGGTGGGCCATGGGT

TTACACACGCGCACATCGTTGCGCTCAGCCAACACCCGGCAGCGTTAGGGACCGTCGCTGTCAAGTATCAGCACATAATC

ACGGCGTTGCCAGAGGCGACACACGAAGACATCGTTGGCGTCGGCAAACAGTGGTCCGGCGCACGCGCCCTGGAGGCCTT

GCTCACGAAGGCGGGGGAGTTGAGAGGTCCGCCGTTACAGTTGGACACAGGCCAACTTCTCAAGATTGCAAAACGTGGCG

GCGTGACCGCAGTGGAGGCAGTGCATGCATGGCGCAATGCACTGACGGGTGCCCCCCTGAACCTG

>M13R

GGTGGCCATCGCCAGCCACGATGGCGGCAAGCAGGCGCTGGAGACGGTGCAACGGCTGTTGCCGGTGCTGTGCCAGGACC

ATGGCCTGACCCCGGCCCAGGTGGTGGCCATCGCCAACAATAACGGCGGCAAGCAGGCGCTGGAGACGGTGCAGCGGCTG

TTGCCGGTGCTGTGCCAGGACCATGGCCTGACCCCGGACCAGGTGGTGGCCATCGCCAGCAATGGCGGCGGCAAGCAGGC

GCTGGAGAGCATTGTTGCCCAGTTATATCGCCCTGATCCGGCGTTGGCCGCGTTGACCAACGACCACCTCGTCGCCTTGG

CCTGCCTCGGCGGACGTCCTGCCCTGGATGCAGTGAAAAAGGGATTGCCGCACGCGCCGGAATTGATCAGAAGAATCAAT

CGCCGTATTCCCGAACGCACGTCCCATCGCGTTGCCGACCTCGCGCACGTGGTGCGCGTGCTTGGTTTTTTCCAGAGCCA

CTCCCACCCAGCGCAAGCATTCGATGACGCCATGACGCAGTTCGGGATGAGCAGGCACGGGTTGGTACAGCTCTTTCGCA

GAGTGGGCGTCACCGAATTCGAAGCCCGCTGCGGAACGCTCCCCCCAGCCTCGCAGCGTTGGGACCGTATCCTCCAGGCA

TCAGGGATGAAAAGGGCCAAACCGTCCCCTACTTCAGCTCAAACACCGGATCAGGCGTCTTTGCATGCATTCGCCGATTC

GCTGGAGCGTGACCTTGATGCGCCCGGCCCAATGCACGAGGGAGATCAGACGCGGGCAAGCAGCCGTAAACGGTCCCGAT

CGGATCGTGCTGTCACCGGCCCCTCCGCACAGCAATCTTTCGAGGTACGCGTTCCCGAACAGCGCGATGCTCTGCATTTG

CCCCTCAGCTGGAGGGTAAAACGCCCGCGTACCAGGATCGGGGGCGGCCTCCCGGATCC

**pTAL*_Bam_*_HI_-14**

>14P1F_1

GGCGGGGGAGTTGAGAGGTCCACCGTTACAGTTGGACACAGGCCAACTTGTCAAGATTGCAAAACGTGGCGGCGTGACCG

CAGTGGAGGCAGTGCATGCATCGCGCAATGCACTGACGGGTGCCCCCCTGAACCTGACCCCGGACCAAGTGGTGGCCATC

GCCAGCAATATTGGCGGCAAGCAGGCGCTGGAGACGGTGCAGCGCCTGTTGCCGGTGCTGTGCCAGGACCATGGCCTGAC

CCCGGACCAGGTGGTGGCCATCGCCAACAATAACGGCGGCAAGCAGGCGCTGGAGACGGTGCAGCGGCTGTTGCCGGTGC

TGTGCCAGGACCATGGCCTGACCCCGGACCAGGTGGTGGCCATCGCCAGCAATATTGGCGGCAAGCAGGCGCTGGAGACG

GTGCAGCGGCTGTTGCCGGTGCTGTGCCAGGACCATGGCCTGACCCCGGACCAGGTGGTGGCCATCGCCAGCCATGGCGG

CGGCAAGCAGGCGCTGGAGACGGTGCAGCGGCTGTTGCCGGTGCTGTGCCAGGACCATGGCCTGACCCCGGACCAGGTGG

TGGCCATCGCCAGCCATGGCGGCGGCAAGCAGGCGCTGGAGACGCTGCAGCGGCTGTTGCCGGTGCTGTGCCAGGACCAT

GGCCTGACCCCGGACCAGGTCGTGGCCATCGCCAGCCACGATGGCGGCAAGCAGGCGCTGGAGACGGTGCAGCGGCTGTT

GCCGGTGCTGTGCCAGGACCATGGCCTGACCCCGGACCAGGTCGTGGCCATCGCCAGCAATGGCGGCGGCAAGCAGGCGC

TGGAGACGCTGCAACGGCTGTTGCCGGTGCTGTGCCAGGACCATGGCCTGACCCCGGACCAGGTCGTGGCCATCGCCAGC

CACGATGGCGGCAAGCAGGCGCTGGAGACGGTGCAGCGGCTGTTGCCGATGCTGTGCCAGGACCATGGCCTGACCCCGGA

CCAGGTGGTGGCA

>14P1F_2

GGGGGAGTTGAGAGGTCCACCGTTACAGTTGGACACAGGCCAACTTGTCAAGATTGCAAAACGTGGCGGCGTGACCGCAG

TGGAGGCAGTGCATGCATCGCGCAATGCACTGACGGGTGCCCCCCTGAACCTGACCCCGGACCAAGTGGTGGCCATCGCC

AGCAATATTGGCGGCAAGCAGGCGCTGGAGACGGTGCAGCGCCTGTTGCCGGTGCTGTGCCAGGACCATGGCCTGACCCC

GGACCAGGTGGTGGCCATCGCCAACAATAACGGCGGCAAGCAGGCGCTGGAGACGGTGCAGCGGCTGTTGCCGGTGCTGT

GCCAGGACCATGGCCTGACCCCGGACCAGGTGGTGGCCATCGCCAGCAATATTGGCGGCAAGCAGGCGCTGGAGACGGTG

CAGCGGCTGTTGCCGGTGCTGTGCCAGGACCATGGCCTGACCCCGGACCAGGTGGTGGCCATCGCCAGCCATGGCGGCGG

CAAGCAGGCGCTGGAGACGGTGCAGCGGCTGTTGCCGGTGCTGTGCCAGGACCATGGCCTGACCCCGGACCAGGTGGTGG

CCATCGCCAGCCATGGCGGCGGCAAGCAGGCGCTGGAGACGCTGCAGCGGCTGTTGCCGGTGCTGTGCCAGGACCATGGC

CTGACCCCGGACCAGGTCGTGGCCATCGCCAGCCACGATGGCGGCAAGCAGGCGCTGGAGACGGTGCAGCGGCTGTTGCC

GGTGCTGTGCCAGGACCATGGCCTGACCCCGGACCAGGTCGTGGCCATCGCCAGCAATGGCGGCGGCAAGCAGGCGCTGG

AGACGCTGCAACGGCTGTTGCCGGTGCTGTGCCAGGACCATGGCCTGACCCCGGACCAGGTCGTGGCCATCGCCAGCCAC

GATGGCGGCAAGCAGGCGCTGGAGACGGTGCAGCGGCTGTTGCCGATGCTGTGCCAGGACCATGGCCTGACCCCGGACCA

GGTGGTGGCCATCGC

>14P1R_1

TGGGCAACAATGCTCTCCAGCGCCTGCTTGCCGCCGCCATTGCTGGCGATGGCCACGACCTGGTCCGGGGTCAGGCCATG

GTCCTGGCACAGCACCGGCAACAGCCGCTGCACCGTCTCCAGCGCCTGCTTGCCGCCATCGTGGCTGGCGATGGCCACGA

CCTGGTCCGGGGTCAGGCCATGGTCCTGGCACAGCACCGGCAACAGCCGTTGCACCGTCTCCAGCGCCTTCTTGCCGCCA

TCGTGGCTGGCGATGGCCACCACCTGGTCCAGGGTCAGGCCATGGTCCTGGCACAGCACCGGCAACAGCCGCTGCACCGT

CTCCAGCGCCTGCTTGCCGCCATCGTGGCTGGCGATGGCCACCACCTGGTCCGGGGTCAGGCCATGGTCCTGGCACAGCA

CCGGCAACAGCCGCTGCACTGTCTCCAGCGCCTGCTTGCCGCCGCCATGGCTGGCGATGGCCACCACCTGGTCCGGGGTC

AGGCCATGGTCCTGGCACAGCATCGGCAACAGCCGCTGCACCGTCTCCAGCGCCTGCTTGCCGCCATCGTGGCTGGCGAT

GGCCACGACCTGGTCCGGGGTCAGGCCATGGTCCTGGCACAGCACCGGCAACAGCCGTTGCAGCGTCTCCAGCGCCTGCT

TGCCGCCGCCATTGCTGGCGATGGCCACGACCTGGTCCGGGGTCAGGCCATGGTCCTGGCACAGCACCGGCAACAGCCGC

TGCACCGTCTCCAGCGCCTGCTTGCCGCCATCGTGGCTGGCGATGGCCACGACCTGGTCCGGGGTCAGGCCATGGTCCTG

GCACAGCACCGGCAACAGCCGCTGCAGCGTCTCCAGCGCCTGCTTGCCGCCGCCATGGCTGGCGATGGCCACCACCTGGT

CCGGGGTCAGGCCATGGTCCTGGCACAGCACCGGCAACAGCCGCTGCACCGTCTCCAGCGCCTGCTTGCCGCCGCCATGG

CTGGCGATGGC

>14P1R_2

TGGCCATCGCCAGCCATGGCGGCGGCAAGCAGGCGCTGGAGACGGTGCAGCGGCTGTTGCCGGTGCTGTGCCAGGACCAT

GGCCTGACCCCGGACCAGGTGGTGGCCATCGCCAGCCATGGCGGCGGCAAGCAGGCGCTGGAGACGCTGCAGCGGCTGTT

GCCGGTGCTGTGCCAGGACCATGGCCTGACCCCGGACCAGGTCGTGGCCATCGCCAGCCACGATGGCGGCAAGCAGGCGC

TGGAGACGGTGCAGCGGCTGTTGCCGGTGCTGTGCCAGGACCATGGCCTGACCCCGGACCAGGTCGTGGCCATCGCCAGC

AATGGCGGCGGCAAGCAGGCGCTGGAGACGCTGCAACGGCTGTTGCCGGTGCTGTGCCAGGACCATGGCCTGACCCCGGA

CCAGGTCGTGGCCATCGCCAGCCACGATGGCGGCAAGCAGGCGCTGGAGACGGTGCAGCGGCTGTTGCCGATGCTGTGCC

AGGACCATGGCCTGACCCCGGACCAGGTGGTGGCCATCGCCAGCCATGGCGGCGGCAAGCAGGCGCTGGAGACAGTGCAG

CGGCTGTTGCCGGTGCTGTGCCAGGACCATGGCCTGACCCCGGACCAGGTGGTGGCCATCGCCAGCCACGATGGCGGCAA

GCAGGCGCTGGAGACGGTGCAGCGGCTGTTGCCGGTGCTGTGCCAGGACCATGGCCTGACCCTGGACCAGGTGGTGGCCA

TCGCCAGCCACGATGGCGGCAAGAAGGCGCTGGAGACGGTGCAACGGCTGTTGCCGGTGCTGTGCCAGGACCATGGCCTG

ACCCCGGACCAGGTCGTGGCCATCGCCAGCCACGATGGCGGCAAGCAGGCGCTGGAGACGGTGCAGCGGCTGTTGCCGGT

GCTGTGCCAGGACCATGGCCTGACCCCGGACCAGGTCGTGGCCATCGCCAGCAATGGCGGCGGCAAGCAGGCGCTGGAGA

GCATTGTTGCCCAGTT

>14P2F

CGCGCACATCGTTGCGCTCAGCCAACACCCGGCAGCGTTAGGGACCGTTGCTGTCACGTATCAGGACATAATCAGGGCGT

TGCCAGAGGCGACACACGAAGACATCGTTGGCGTCGGCAAACAGTGGTCCGGCGCACGCGCCCTGGAGGCCTTGCTCACG

AAGGCGGGGGAGTTGAGAGGTCCACCGTTACAGTTGGACACAGGCCAACTTGTCAAGATTGCAAAACGTGGCGGCGTGAC

CGCAGTGGAGGCAGTGCATGCATCGCGCAATGCACTGACGGGTGCCCCCCTGAACCTGACCCCGGACCAAGTGGTGGCCA

TCGCCAGCAATATTGGCGGCAAGCAGGCGCTGGAGACGGTGCAGCGCCTGTTGCCGGTGCTGTGCCAGGACCATGGCCTG

ACCCCGGACCAGGTGGTGGCCATCGCCAACAATAACGGCGGCAAGCAGGCGCTGGAGACGGTGCAGCGGCTGTTGCCGGT

GCTGTGCCAGGACCATGGCCTGACCCCGGACCAGGTGGTGGCCATCGCCAGCAATATTGGCGGCAAGCAGGCGCTGGAGA

CGGTGCAGCGGCTGTTGCCGGTGCTGTGCCAGGACCATGGCCTGACCCCGGACCAGGTGGTGGCCATCGCCAGCCATGGC

GGCGGCAAGCAGGCGCTGGAGACGGTGCAGCGGCTGTTGCCGGTGCTGTGCCAGGACCATGGCCTGACCCCGGACCAGGT

GGTGGCCATCGCCAGCCATGGCGGCGGCAAGCAGGCGCTGGAGACGCTGCAGCGGCTGTTGCCGGTGCTGTGCCAGGACC

ATGGCCTGACCCCGGACCAGGTCGTGGCCATCGCCAGCCACGATGGCGGCAAGCAGGCGCTGGAGACGGTGCAGCGGCTG

TTGCCGGTGCTGTGCCAGGACCATGGCCTGACCCCGGACCAGGTCGTGGCCATCGCCAGCAATGGCGGCGGCAAGCAGGC

GCTGGAGACGCT

>14P2R

CCCTGATTATGTCCTGATACGTGACAGCAACGGTCCCTAACGCTGCCGGGTGTTGGCTGAGCGCAACGATGTGCGCGTGT

GTAAACCCATGGCCCACCAGTGCCTCGTGGTGCTGCGCCACTGTCGAACGCACCTTCGGTTTGATCTTCTCTTGCTGCTG

CTGACTGTAGCCGAGCGTGCGTAGATCCACCTGCGCGGCCGGCGAAGCGTCGGAGGGTTGCGCCGCACGCCGTCGCGGGG

CCGGCTTGGCGCGCGGCGGCCGCGCGGCAGTGACAGCGACAGGCACGGTGGGTGGCGGGTCATCGGCTGCACGCAGACCC

GATTGCGCCTCATCCCATTCTGCTGGGGCAGCCGCTGTATGCGGCGTGCCGACGGCAGGCATCGAATCAAGAAGCGATGT

ATCAAGAAGCGACGGATCGAACTGACGGAGCAGATCGCTGAAGCTGCCCGCCGAGAACGCAGGCGAGGGCGCAGGGGGAG

ATGGCAGCCGGGTCCGGGACATCGTCCGCCGAGCGGGCAAGCCATCCAGGGGGCCGCCAGCAGGCGGAGCCCCCCCCCGA

TCTGCAGTCGGCTGAACCCTATCCGGTTGGGGTCCGGGCAGAAGCTCGCGGGCAGGACTTGGCGTGCGCGAACGAATGGG

ATCC

>14P3F

CACGCGCCGGAATTGATCAGAAGAGTCAATCGCCGTATTGGCGAACGCACGTCCCATCGCGTTGCCGACTACGCGCAAGT

GGTTCGCGTGCTGGAGTTTTTCCAGTGCCACTCCCACCCAGCGCAAGCATTCGATGACGCCATGACGCAGTTCGGGATGA

GCAGGCACGGGTTGGTACAGCTCTTTCGCAGAGTGGGCGTCACCGAATTCGAAGCCCGCTGCGGAACGCTCCCCCCAGCC

TCGCAGCGTTGGGACCGTATCCTCCAGGCATCAGGGATGAAAAGGGCCAAACCGTCCCCTACTTCAGCTCAAACACCGGA

TCAGGCGTCTTTGCATGCATTCGCCGATTCGCTGGAGCGTGACCTTGATGCGCCCAGCCCAATGCACGAGGGAGATCAGA

CGCGGGCAAGCAGCCGTAAACGGTCCCGATCGGATCGTGCTGTCACCGGCCCCTCCACACAGCAATCTTTCGAGGTGCGC

GTTCCCGAACAGCGCGATGCGCTGCATTTGCCCCTCAGCTGGAGGGTAAAACGCCCGCGTACCAGGATCGGGGGCGGCCT

CCCGGATCC

>14P3R

ATTCGGTGACGCCCACTCTGCGAAAGAGCTGTACCAACCCGTGCCTGCTCATCCCGAACTGCGTCATGGCGTCATCGAAT

GCTTGCGCTGGGTGGGAGTGGCACTGGAAAAACTCCAGCACGCGAACCACTTGCGCGTAGTCGGCAACGCGATGGGACGT

GCGTTCGCCAATACGGCGATTGACTCTTCTGATCAATTCCGGCGCGTGCGGCAATCCCTTTTTCACTGCATCCAGGGCAG

GACGTCCGCCGAGGCAGGCCAAGGCGACGAGGTGGTCGTTGGTCAACGCGGCCAACGCCGGATCAGGGCGAGATAACTGG

GCAACAATGCTCTCCAGCGCCTGCTTGCCGCCGCCATTGCTGGCGATGGCCACGACCTGGTCCGGGGTCAGGCCATGGTC

CTGGCACAGCACCGGCAACAGCCGCTGCACCGTCTCCAGCGCCTGCTTGCCGCCATCGTGGCTGGCGATGGCCACGACCT

GGTCCGGGGTCAGGCCATGGTCCTGGCACAGCACCGGCAACAGCCGTTGCACCGTCTCCAGCGCCTTCTTGCCGCCATCG

TGGCTGGCGATGGCCACCACCTGGTCCAGGGTCAGGCCATGGTCCTGGCACAGCACCGGCAACAGCCGCTGCACCGTCTC

CAGCGCCTGCTTGCCGCCATCGTGGCTGGCGATGGCCACCACCTGGTCCGGGGTCAGGCCATGGTCCTGGCACAGCACCG

GCAACAGCCGCTGCACTGTCTCCAGCGCCTGCTTGCCGCCGCCATGGCTGGCGATGGCCACCACCTGGTCCGGGGTCAGG

CCATGGTCCTGGCACAGCATCGGCAACAGCCGCTGCACCGTCTCCAGCGCCTGCTTGCCGCCATCGTGGCTGGCGATGGC

CACGACCTGGTCCGGGGTCAGGCCATGGTCCTGGCACAGCACCGGCAACAGCCGTTGCAGCGTCTCCAGCGCCTGCTTGC

CGCCGCCATTG

>M13F

GGATCCCATTCGTTCGCGCACGCCAAGTCCTGCCCGCGAGCTTCTGCCCGGACCCCAACCGGATAGGGTTCAGCCGACTG

CAGATCGGGGGGGGGCTCCGCCTGCTGGCGGCCCCCTGGATGGCTTGCCCGCTCGGCGGACGATGTCCCGGACCCGGCTG

CCATCTCCCCCTGCGCCCTCGCCTGCGTTCTCGGCGGGCAGCTTCAGCGATCTGCTCCGTCAGTTCGATCCGTCGCTTCT

TGATACATCGCTTCTTGATTCGATGCCTGCCGTCGGCACGCCGCATACAGCGGCTGCCCCAGCAGAATGGGATGAGGCGC

AATCGGGTCTGCGTGCAGCCGATGACCCGCCACCCACCGTGCCTGTCGCTGTCACTGCCGCGCGGCCGCCGCGCGCCAAG

CCGGCCCCGCGACGGCGTGCGGCGCAACCCTCCGACGCTTCGCCGGCCGCGCAGGTGGATCTACGCACGCTCGGCTACAG

TCAGCAGCAGCAAGAGAAGATCAAACCGAAGGTGCGTTCGACAGTGGCGCAGCACCACGAGGCACTGGTGGGCCATGGGT

TTACACACGCGCACATCGTTGCGCTCAGCCAACACCCGGCAGCGTTAGGGACCGTTGCTGTCACGTATCAGGACATAATC

AGGGCGTTGCCAGAGGCGACACACGAAGACATCGTTGGCGTCGGCAAACAGTGGTCCGGCGCACGCGCCCTGGAGGCCTT

GCTCACGAAGGCGGGGGAGTTGAGAGGTCCACCGTTACAGTTGGACACAGGCCAACTTGTCAAGATTGCAAAACGTGGCG

GCGTGACCGCAGTGGAGGCAGTGCATGCATCGCGCAATGCACTGACGGGTGCCCCCCTGAACCTGACCCCGGACCAAGTG

GTGGCCATCGCCAGCAATATTGGCGGCAAGCAGGCGCTGGAGACGGTGCAGCGCCTGTTGCCGGTGCTGTGCCAGGACCA

TGGC

>M13R

CCAGGTGGTGGCCATCGCCAGCCACGATGGCGGCAAGAAGGCGCTGGAGACGGTGCAACGGCTGTTGCCGGTGCTGTGCC

AGGACCATGGCCTGACCCCGGACCAGGTCGTGGCCATCGCCAGCCACGATGGCGGCAAGCAGGCGCTGGAGACGGTGCAG

CGGCTGTTGCCGGTGCTGTGCCAGGACCATGGCCTGACCCCGGACCAGGTCGTGGCCATCGCCAGCAATGGCGGCGGCAA

GCAGGCGCTGGAGAGCATTGTTGCCCAGTTATCTCGCCCTGATCCGGCGTTGGCCGCGTTGACCAACGACCACCTCGTCG

CCTTGGCCTGCCTCGGCGGACGTCCTGCCCTGGATGCAGTGAAAAAGGGATTGCCGCACGCGCCGGAATTGATCAGAAGA

GTCAATCGCCGTATTGGCGAACGCACGTCCCATCGCGTTGCCGACTACGCGCAAGTGGTTCGCGTGCTGGAGTTTTTCCA

GTGCCACTCCCACCCAGCGCAAGCATTCGATGACGCCATGACGCAGTTCGGGATGAGCAGGCACGGGTTGGTACAGCTCT

TTCGCAGAGTGGGCGTCACCGAATTCGAAGCCCGCTGCGGAACGCTCCCCCCAGCCTCGCAGCGTTGGGACCGTATCCTC

CAGGCATCAGGGATGAAAAGGGCCAAACCGTCCCCTACTTCAGCTCAAACACCGGATCAGGCGTCTTTGCATGCATTCGC

CGATTCGCTGGAGCGTGACCTTGATGCGCCCAGCCCAATGCACGAGGGAGATCAGACGCGGGCAAGCAGCCGTAAACGGT

CCCGATCGGATCGTGCTGTCACCGGCCCCTCCACACAGCAATCTTTCGAGGTGCGCGTTCCCGAACAGCGCGATGCGCTG

CATTTGCCCCTCAGCTGGAGGGTAAAACGCCCGCGTACCAGGATCGGGGGCGGCCTCCCGGATCC

**pTAL*_Bam_*_HI_-16**

> pCC2FOS-MscI-1

GGAGACGGTGCAGCGGCTGTTGCCGGTGCTGTGCCAGGACCATAGCCTGACCCCGGACCAGGTCGTGGCCATCGCCAGCA

ATATAGGCGGCAAGCAGGCGCTGGAGACGGTGCAGCGGCTGTTGCCGGTGCTGTGCCAGGACCATGGCCTGACCCCGGAC

CAGGTGGTGGCCATCGCCAGCAATAGTGGCGGCAAGCAGGCGCTGGAGACGGTGCAGCGGCTGTTGCCGGTGCTGTGCCA

GGACCATGGCCTGACCCCGGACCAGGTGATGACCATCGCCAGCAATAACGGCGGCAAGCAGGCGCTGGAGACGGTGCAAC

GGCTGTTGCCGGTGCTGTGCCAGGACCATGGCCTGACCCCGGACCAGGTGGTGACCATCGCCAGCAATGGCGGCGGCAAG

CAGGCGCTGGAGACGGTGCAGCGGCTGTTGCCGGTGCTGTGCCAGGCCCATGGCCTGACCCCGGACCAAGTGGTGGCCAT

CGCCAACAATAACGGCGGCAAGCAGGCGCTGGAGACGGTGCAACGGCTGTTGCCGGTGCTATGCCAGGCCCATGGCCTGA

CCCCGGCCCAGGTCGTGGCCATCGCCAGCAATAGCGGCGGCAAGCAGGCGCTGGAGACGGTGCAGCGGCTGTTGCCGGTG

CTGTGCCAGGCCCATGGCCTGACCCCGGACCAGGTGGTGGCCATCGCCAGCAATGGCGGCAAGCAGGCGCTGGAGACGGT

GCAGCGGCTGTTGCCGGTGCTGTGCCAGGCCCATGGCCTGACCCCGGACCAGGTGGTGGCCATCGCCAGCAATAGTGGCG

GCAAGCAGGCGCTGGAGACGGTGCAGCGGCTGTTGCCGGTGCTGTGCCAGGCCCATGGCCTGACCCCGGACCAGGTGGTG

G

> pCC2FOS-MscI-2

GCGCTGGAGACGGTACAGCGGCTGTTGCCGGTGCTGTGCCAGGCCCATGGCCTGACCCCGGACCAGGTCGTGGCCATCGC

CAGCAATGGCGGCAAGCAGGCGCTGGAGACGGTGCAGCGGCTGTTGCCGGTGCTGTGCCAGGACCATAGCCTGACCCCGG

ACCAGGTCGTGGCCATCGCCAGCAATATAGGCGGCAAGCAGGCGCTGGAGACGGTGCAGCGGCTGTTGCCGGTGCTGTGC

CAGGACCATGGCCTGACCCCGGACCAGGTGGTGGCCATCGCCAGCAATAGTGGCGGCAAGCAGGCGCTGGAGACGGTGCA

GCGGCTGTTGCCGGTGCTGTGCCAGGACCATGGCCTGACCCCGGACCAGGTGATGACCATCGCCAGCAATAACGGCGGCA

AGCAGGCGCTGGAGACGGTGCAACGGCTGTTGCCGGTGCTGTGCCAGGACCATGGCCTGACCCCGGACCAGGTGGTGACC

ATCGCCAGCAATGGCGGCGGCAAGCAGGCGCTGGAGACGGTGCAGCGGCTGTTGCCGGTGCTGTGCCAGGCCCATGGCCT

GACCCCGGACCAAGTGGTGGCCATCGCCAACAATAACGGCGGCAAGCAGGCGCTGGAGACGGTGCAACGGCTGTTGCCGG

TGCTATGCCAGGCCCATGGCCTGACCCCGGCCCAGGTCGTGGCCATCGCCAGCAATAGCGGCGGCAAGCAGGCGCTGGAG

ACGGTGCAGCGGCTGTTGCCGGTGCTGTGCCAGGCCCATGGCCTGACCCCGGACCAGGTGGTGG

> pCC2FOS-MscI-3

CAACAGCCGTTGCACCGTCTCCAGCGCCTGCTTGCCGCCATCGTGGCTGGCGATGGCCACCACCTGGTCCAGGGTCAGGC

CATGGTCCTGGCACAGCACCGGCAACAGCCGCTGCACCGTCTCCAGCGCCTGCTTGCCGCCAATATTGCTGGCAATGGCC

ACCACCTGGTCCGGGGTCAGGCCATGGTCCTGGCACAGCACCGGCAACAGCCGCTGCACCGTCTCCAGCGCCTGCTTGCC

GCCATCGTGGCTGGCGATGGCCACCACCTGGTCCGAGGTCAGGCCATGGTCCTGGCACAGCACCGGCAACAGCCGCTGCA

CCGTCTCCAGCGCCTGCTTGCCGCCGCCATGGCTGGCGATGGCCACCACCTGGTCCAGGGTCAGGCCATGGTCCTGGCAC

AGCACCGGCAACAGCCGCTGCACCGTCTCCAGCGCCTGCTTGCCGCCATCGTGGCTGGCGATGGCCACCACCTGGTCCGG

GGTCAGGCCATGGTCCTGGCACAGCACCGGCAACAGCCGCTGCACCGTCTCCAGCGCCTGCTTGCCGCCATTGCTGGCGA

TGGCCACCACCTGGTCCGGGGTCAGGCCATGGTCCTGGCACAGCACCGGCAACAGCCGCTGCACCGTCTCCAGCGCCTGC

TTGCCGCCACTATTGCTGGCGATGGCCACCACCTGGTCCGGGGTCAGGCCATGGGCCTGGCACAGCACCGGCAACAGCCG

CTGCACCGTCTCCAGCGCCTGCTTGCCGCCGTTATTGTTGGCGATGG

> pCC2FOS-MscI-4

CCATCGCCAACAATAACGGCGGCAAGCAGGCGCTGGAGACGGTGCAACGGCTGTTGCCGGTGCTATGCCAGGCCCATGGC

CTGACCCCGGCCCAGGTCGTGGCCATCGCCAGCAATAGCGGCGGCAAGCAGGCGCTGGAGACGGTGCAGCGGCTGTTGCC

GGTGCTGTGCCAGGCCCATGGCCTGACCCCGGACCAGGTGGTGGCCATCGCCAGCAATGGCGGCAAGCAGGCGCTGGAGA

CGGTGCAGCGGCTGTTGCCGGTGCTGTGCCAGGCCCATGGCCTGACCCCGGACCAGGTGGTGGCCATCGCCAGCAATAGT

GGCGGCAAGCAGGCGCTGGAGACGGTGCAGCGGCTGTTGCCGGTGCTGTGCCAGGCCCATGGCCTGACCCCGGACCAGGT

GGTGGCCATCGCCAACAATAACGGCGGCAAGCAGGCGCTGGAGACGGTGCAGCGGCTGTTGCCGGTGCTGTGCCAGGCCC

ATGGCCTGACCCCGGACCAGGTGGTGGCCATCGCCAGCAATAGTGGCGGCAAGCAGGCGCTGGAGACGGTGCAGCGGCTG

TTGCCGGTGCTGTGCCAGGACCATGGCCTGACCCCGGACCAGGTGGTGGCCATCGCCAGCAATGGCGGCAAGCAGGCGCT

GGAGACGGTGCAGCGGCTGTTGCCGGTGCTGTGCCAGGACCATGGCCTGACCCCGGACCAGGTGGTGGCCATCGCCAGCC

ACGATGGCGGCAAGCAGGCGCTGGAGACGGTGCAGCGGCTGTTGCCGGTGCTGTGCCAGGACCATGGCCTGACCCTGGAC

CAGGTGGTGGCCATCGCCAGCCATGGCGGCGGCAAGCAGGCGCTGGAGACGGTGCAGCGGCTGTTGCCGGTGCTG

> pCC2FOS-MscI-5

GAGACGGTGCAGCGGCTGTTGCCGGTGCTGTGCCAGGACCATGGCCTGACCCCGGACCAGGTGATGACCATCGCCAGCAA

TAACGGCGGCAAGCAGGCGCTGGAGACGGTGCAACGGCTGTTGCCGGTGCTGTGCCAGGACCATGGCCTGACCCCGGACC

AGGTGGTGACCATCGCCAGCAATGGCGGCGGCAAGCAGGCGCTGGAGACGGTGCAGCGGCTGTTGCCGGTGCTGTGCCAG

GCCCATGGCCTGACCCCGGACCAAGTGGTGGCCATCGCCAACAATAACGGCGGCAAGCAGGCGCTGGAGACGGTGCAACG

GCTGTTGCCGGTGCTATGCCAGGCCCATGGCCTGACCCCGGCCCAGGTCGTGGCCATCGCCAGCAATAGCGGCGGCAAGC

AGGCGCTGGAGACGGTGCAGCGGCTGTTGCCGGTGCTGTGCCAGGCCCATGGCCTGACCCCGGACCAGGTGGTGGCCATC

GCCAGCAATGGCGGCAAGCAGGCGCTGGAGACGGTGCAGCGGCTGTTGCCGGTGCTGTGCCAGGCCCATGGCCTGACCCC

GGACCAGGTGGTGGCCATCGCCAGCAATAGTGGCGGCAAGCAGGCGCTGGAGACGGTGCAGCGGCTGTTGCCGGTGCTGT

GCCAGGCCCATGGCCTGACCCCGGACCAGGTGGTGGCCATCGCCAACAATAACGGCGGCAAGCAGGCGCTGGAGACGGTG

CAGCGGCTGTTGCCGGTGCTGTGCCAGGCCCATGGCCTGACCCCGGACCAGGTGGTGGCCATCGCCAGCAATAGTGGCG

> pCC2FOS-MscI-6

CCATCGCCAACAATAACGGCGGCAAGCAGGCGCTGGAGACGGTGCAGCGGCTGTTGCCGGTGCTGTGCCAGGCCCATGGC

CTGACCCCGGACCAGGTGGTGGCCATCGCCAGCAATAGTGGCGGCAAGCAGGCGCTGGAGACGGTGCAGCGGCTGTTGCC

GGTGCTGTGCCAGGACCATGGCCTGACCCCGGACCAGGTGGTGGCCATCGCCAGCAATGGCGGCAAGCAGGCGCTGGAGA

CGGTGCAGCGGCTGTTGCCGGTGCTGTGCCAGGACCATGGCCTGACCCCGGACCAGGTGGTGGCCATCGCCAGCCACGAT

GGCGGCAAGCAGGCGCTGGAGACGGTGCAGCGGCTGTTGCCGGTGCTGTGCCAGGACCATGGCCTGACCCTGGACCAGGT

GGTGGCCATCGCCAGCCATGGCGGCGGCAAGCAGGCGCTGGAGACGGTGCAGCGGCTGTTGCCGGTGCTGTGCCAGGACC

ATGGCCTGACCTCGGACCAGGTGGTGGCCATCGCCAGCCACGATGGCGGCAAGCAGGCGCTGGAGACGGTGCAGCGGCTG

TTGCCGGTGCTGTGCCAGGACCATGGCCTGACCCCGGACCAGGTGGTGGCCATTGCCAGCAATATTGGCGGCAAGCAGGC

GCTGGAGACGGTGCAGCGGCTGTTGCCGGTGCTGTGCCAGGACCATGGCCTGACCCTGGACCAGGTGGTGGCCATCGCCA

GCCACGATGGCGGCAAGCAGGCGCTGGAGACGGTGCAACGGCTGTTGCCGGTGCTGTGCCAGGACCATGGCCTGACCCCG

GACCAGGTCGTGGCCATCGCCAGCCACGATGGCGGCAAGCAGGCGCTGGAGACGGTGCAGCGGCTGTTGCCGGTGCTGTG

CCAGGACCA

>16P1F_1

CGAGGGAGTTGAGAGGTCCGCCGTTACAGTTGGACACAGGCCAACTTCTCAAGATTGCAAAACGTGGCGGCGTGACCGCA

GTGGAGGCAGTGCATGCATGGCGCAATGCACTGACGGGTGCCCCCCTGAACCTGACCCCGGACCAAGTGGTGGCCATCGC

CAGCAATATTGGCGGCAACCAGGCGCTGGAGACGGTACAGCGGCTGTTGCCGGTGCTGTGCCAGGCCCATGGCCTGACCC

CGGACCAGGTCGTGGCCATCGCCAGCAATGGCGGCAAGCAGGCGCTGGAGACGGTGCAGCGGCTGTTGCCGGTGCTGTGC

CAGGACCATAGCCTGACCCCGGACCAGGTCGTGGCCATCGCCAGCAATATAGGCGGCAAGCAGGCGCTGGAGACGGTGCA

GCGGCTGTTGCCGGTGCTGTGCCAGGACCATGGCCTGACCCCGGACCAGGTGGTGGCCATCGCCAGCAATAGTGGCGGCA

AGCAGGCGCTGGAGACGGTGCAGCGGCTGTTGCCGGTGCTGTGCCAGGACCATGGCCTGACCCCGGACCAGGTGATGACC

ATCGCCAGCAATAACGGCGGCAAGCAGGCGCTGGAGACGGTGCAACGGCTGTTGCCGGTGCTGTGCCAGGACCATGGCCT

GACCCCGGACCAGGTGGTGACCATCGCCAGCAATGGCGGCGGCAAGCAGGCGCTGGAGACGGTGCAGCGGCTGTTGCCGG

TGCTGTGCCAGGACCATGGCCTGACCCCGGACCAAGTGGTGGCCATCGCCAACAATAACGGCGGCAAGCAGGCGCTGGAG

ACGGTGCAACGGCTGTTGCCGGTGCTATGCCAGGACCATGGCCTGACCCCGGACCAGGTCGTGGCCATCGCCAGCAATAG

CGGCGGCAAGCAGGCGCTGGAGACGGTGCAGCGGCTGTTGCCGGTGCTGTGCCAGGACCATGGCCTGACCCCGGACCAGG

TGGTGGCCATCGCCAGCAA

>16P1F_2

GGAGGCGAGGGAGTTGAGAGGTCCGCCGTTACAGTTGGACACAGGCCAACTTCTCAAGATTGCAAAACGTGGCGGCGTGA

CCGCAGTGGAGGCAGTGCATGCATGGCGCAATGCACTGACGGGTGCCCCCCTGAACCTGACCCCGGACCAAGTGGTGGCC

ATCGCCAGCAATATTGGCGGCAACCAGGCGCTGGAGACGGTACAGCGGCTGTTGCCGGTGCTGTGCCAGGCCCATGGCCT

GACCCCGGACCAGGTCGTGGCCATCGCCAGCAATGGCGGCAAGCAGGCGCTGGAGACGGTGCAGCGGCTGTTGCCGGTGC

TGTGCCAGGACCATAGCCTGACCCCGGACCAGGTCGTGGCCATCGCCAGCAATATAGGCGGCAAGCAGGCGCTGGAGACG

GTGCAGCGGCTGTTGCCGGTGCTGTGCCAGGACCATGGCCTGACCCCGGACCAGGTGGTGGCCATCGCCAGCAATAGTGG

CGGCAAGCAGGCGCTGGAGACGGTGCAGCGGCTGTTGCCGGTGCTGTGCCAGGACCATGGCCTGACCCCGGACCAGGTGA

TGACCATCGCCAGCAATAACGGCGGCAAGCAGGCGCTGGAGACGGTGCAACGGCTGTTGCCGGTGCTGTGCCAGGACCAT

GGCCTGACCCCGGACCAGGTGGTGACCATCGCCAGCAATGGCGGCGGCAAGCAGGCGCTGGAGACGGTGCAGCGGCTGTT

GCCGGTGCTGTGCCAGGCCCATGGCCTGACCCCGGACCAAGTGGTGGCCATCGCCAACAATAACGGCGGCAAGCAGGCGC

TGGAGACGGTGCAACGGCTGTTGCCGGTGCTATGCCAGGCCCATGGCCTGACCCCGGCCCAGGTCGTGGCCATCGCCAGC

AATAGCGGCGGCAAGCAGGCGCTGGAGACGGTGCAGCGGCTGTTGCCGGTGCTGTGCCAGGCCCATGGCCTGACCCCGGA

CCAGGTGGTGGCCATCGCC

>16P1R_2

CTGGGCAACAATGCTCTCCAGCGCCTGCTTGCCGCCGCCATTGCTGGCGATGGCCACCACCTGGGCCGGGGTCAGGCCAT

GGTCCTGGCACAGCACCGGCAACAGCCGCTGCACCGTCTCCAGCGCCTGCTTGCCGCCATCGTGGCTGGCGATGGCCACG

ACCTGGTCCGGGGTCAGGCCATGGTCCTGGCACAGCACCGGCAACAGCCGTTGCACCGTCTCCAGCGCCTGCTTGCCGCC

ATCGTGGCTGGCGATGGCCACCACCTGGTCCAGGGTCAGGCCATGGTCCTGGCACAGCACCGGCAACAGCCGCTGCACCG

TCTCCAGCGCCTGCTTGCCGCCAATATTGCTGGCAATGGCCACCACCTGGTCCGGGGTCAGGCCATGGTCCTGGCACAGC

ACCGGCAACAGCCGCTGCACCGTCTCCAGCGCCTGCTTGCCGCCATCGTGGCTGGCGATGGCCACCACCTGGTCCGAGGT

CAGGCCATGGTCCTGGCACAGCACCGGCAACAGCCGCTGCACCGTCTCCAGCGCCTGCTTGCCGCCGCCATGGCTGGCGA

TGGCCACCACCTGGTCCAGGGTCAGGCCATGGTCCTGGCACAGCACCGGCAACAGCCGCTGCACCGTCTCCAGCGCCTGC

TTGCCGCCATCGTGGCTGGCGATGGCCACCACCTGGTCCGGGGTCAGGCCATGGTCCTGGCACAGCACCGGCAACAGCCG

CTGCACCGTCTCCAGCGCCTGCTTGCCGCCATTGCTGGCGATGGCCACCACCTGGTGCTGGGTCATGACATGGTGCTGGA

ACAGCAACAGCAACAGCCGCTGCATCGTCGACAGCGCCTGCTTGTCGCCACTATTGCTGGCGATGGACACCACCTGCTCC

GGCGTCAAGCCATGGTCCTGGTACAGCATCGGCAACAGCCGCTGCATCGTCTCCAGCGCCTGCTTGCCGCCATTATTGTT

GGCGATG

>16P1R_2

TGGTGGCCATCGCCAACAATAACGGCGGCAAGCAGGCGCTGGAGACGGTGCAGCGGCTGTTGCCGGTGCTGTGCCAGGCC

CATGGCCTGACCCCGGACCAGGTGGTGGCCATCGCCAGCAATAGTGGCGGCAAGCAGGCGCTGGAGACGGTGCAGCGGCT

GTTGCCGGTGCTGTGCCAGGACCATGGCCTGACCCCGGACCAGGTGGTGGCCATCGCCAGCAATGGCGGCAAGCAGGCGC

TGGAGACGGTGCAGCGGCTGTTGCCGGTGCTGTGCCAGGACCATGGCCTGACCCCGGACCAGGTGGTGGCCATCGCCAGC

CACGATGGCGGCAAGCAGGCGCTGGAGACGGTGCAGCGGCTGTTGCCGGTGCTGTGCCAGGACCATGGCCTGACCCTGGA

CCAGGTGGTGGCCATCGCCAGCCATGGCGGCGGCAAGCAGGCGCTGGAGACGGTGCAGCGGCTGTTGCCGGTGCTGTGCC

AGGACCATGGCCTGACCTCGGACCAGGTGGTGGCCATCGCCAGCCACGATGGCGGCAAGCAGGCGCTGGAGACGGTGCAG

CGGCTGTTGCCGGTGCTGTGCCAGGACCATGGCCTGACCCCGGACCAGGTGGTGGCCATTGCCAGCAATATTGGCGGCAA

GCAGGCGCTGGAGACGGTGCAGCGGCTGTTGCCGGTGCTGTGCCAGGACCATGGCCTGACCCTGGACCAGGTGGTGGCCA

TCGCCAGCCACGATGGCGGCAAGCAGGCGCTGGAGACGGTGCAACGGCTGTTGCCGGTGCTGTGCCAGGACCATGGCCTG

ACCCCGGACCAGGTCGTGGCCATCGCCAGCCACGATGGCGGCAAGCAGGCGCTGGAGACGGTGCAGCGGCTGTTGCCGGT

GCTGTGCCAGGACCATGGCCTGACCCCGGCCCAGGTGGTGGCCATCGCCAGCAATGGCGGCGGCAAGCAGGCGCTGGAGA

GCATTGTTGCCCAGTTAT

>16P2F

CGCGCACATCGTTGCGCTCAGCCAACACCCGGCAGCGTTAGGGACCGTTGCTGTCACGTATCAGGACATAATCAGGGCGT

TGCCAGAGGCGACACACGAAGACATCGTTGGCGTCGGCAAACAGTGGTCCGGCGCACGCGCCCTGGAGGCCTTGCTCACG

GAGGCGAGGGAGTTGAGAGGTCCGCCGTTACAGTTGGACACAGGCCAACTTCTCAAGATTGCAAAACGTGGCGGCGTGAC

CGCAGTGGAGGCAGTGCATGCATGGCGCAATGCACTGACGGGTGCCCCCCTGAACCTGACCCCGGACCAAGTGGTGGCCA

TCGCCAGCAATATTGGCGGCAACCAGGCGCTGGAGACGGTACAGCGGCTGTTGCCGGTGCTGTGCCAGGCCCATGGCCTG

ACCCCGGACCAGGTCGTGGCCATCGCCAGCAATGGCGGCAAGCAGGCGCTGGAGACGGTGCAGCGGCTGTTGCCGGTGCT

GTGCCAGGACCATAGCCTGACCCCGGACCAGGTCGTGGCCATCGCCAGCAATATAGGCGGCAAGCAGGCGCTGGAGACGG

TGCAGCGGCTGTTGCCGGTGCTGTGCCAGGACCATGGCCTGACCCCGGACCAGGTGGTGGCCATCGCCAGCAATAGTGGC

GGCAAGCAGGCGCTGGAGACGGTGCAGCGGCTGTTGCCGGTGCTGTGCCAGGACCATGGCCTGACCCCGGACCAGGTGAT

GACCATCGCCAGCAATAACGGCGGCAAGCAGGCGCTGGAGACGGTGCAACGGCTGTTGCCGGTGCTGTGCCAGGACCATG

GCCTGACCCCGGACCAGGTGGTGACCATCGCCAGCAATGGCGGCGGCAAGCAGGCGCTGGAGACGGTGCAGCGGCTGTTG

CCGGTGCTGTGCCAGGACCATGGCCTGACCCCGGACCAAGTGGTGGCCATCGCCAACAATAACGGCGGCAAGCAGGCGCT

GGAAACGGTGCAACGGCTG

>16P2R

GATTATGTCCTGATACGTGACAGCAACGGTCCCTAACGCTGCCGGGTGTTGGCTGAGCGCAACGATGTGCGCGTGTGTAA

ACCCATGGCCCACCAGTGCCTCGTGGTGCTGCGCCACTGTCGAACGCACCTTCGGTTTGATCTTCTCTTGCTGCTGCTGA

CTGTAGCCGAGCGTGCGTAGATCCACCTGCGCGGCCGGCGAAGCGTCGGAGGGTTGCGCCGCACGCCGTCGCGGGGCCGG

CTTGGCGCGCGGCGGCCGCGCGGCAGTGACAGCGACACGCACAGTGGGTGGCGGGTCATCGGCTGCACGCAGACCCGATT

GCACCTCATCCCACTCTGCTGGGGCAGCCGCTGTATGCGGCGTGCCGACGGCAGGCATCGAATCAAGAAGCGATGTATCA

AGAAGCGACGGATCGAACTGACGGAGCAGATCGCTGAAGCTGCCCGCCGAGAACGCAGGCGAGGGTGCAGGGGGAGATGG

CAGCCGGGTCCGGGACATCGTCCGCCGAACGGGCAAGCCATCCAGGGGGCCGCCAGCAGGCGGAGTCCCCCCCCGATCTG

CAGTCGGCTGAACCCTATCCGGTTGGGGTCCGGGCAGAAGCTCGCGGGCAGGACTTGGCGTGCGCGAACGAATGGGATCC

>16P3F

ATTGCCGCACGCGCCGGAATTGATCAGAAGAATCAATCGCCGTATTCCCGAACGCACGTCCCATCGCGTTGCCGACTACG

CGCAAGTGGTTCGCGTGCTGGAGTTTTTCCAGTGCCACTCCCACCCAGCGTACGCATTTGATGAGGCCATGACGCAGTTC

GGGATGAGCAGGCACGGGTTGGTACAGCTCTTTCGCAGAGTGGGCGTCACCGAATTCGAAGCCCGCTGCGGAACGCTCCC

CCCAGCCTCGCAGCGTTGGGACCGTATCCTCCAGGCATCAGGGATGAAAAGGGCCAAACCGTCCCCTACTTCAGCTCAAA

CACCGGATCAGGCGTCTTTGCATGCATTCGCCGATTCGCTGGAGCGTGACCTTGATGCGCCCAGCCCAATGCACGAGGGA

GATCAGACGCGGGCAAGCAGCCGTAAACGGTCCCGATCGGATCGTGCTGTCACCGGCCCCTCCACACAGCAATCTTTCGA

GGTGCGCGTTCCCGAACAGCGCGATGCGCTGCATTTGCCCCTCAGCTGGAGGGTAAAACGCCCGCGTACCAGGATCGGGG

GCGGCCTCCCGGATCC

>16P3R

ATTCGGTGACGCCCACTCTGCGAAAGAGCTGTACCAACCCGTGCCTGCTCATCCCGAACTGCGTCATGGCCTCATCAAAT

GCGTACGCTGGGTGGGAGTGGCACTGGAAAAACTCCAGCACGCGAACCACTTGCGCGTAGTCGGCAACGCGATGGGACGT

GCGTTCGGGAATACGGCGATTGATTCTTCTGATCAATTCCGGCGCGTGCGGCAATCCCTTTTTCACTGCATCCAGGGCAG

GACGTCCGCCGAGGCAGGCCAAGGCGACGAGGTGGTCGTTGGTCAACGCGGCCAACGCCGGATCAGGGCGAGATAACTGG

GCAACAATGCTCTCCAGCGCCTGCTTGCCGCCGCCATTGCTGGCGATGGCCACCACCTGGGCCGGGGTCAGGCCATGGTC

CTGGCACAGCACCGGCAACAGCCGCTGCACCGTCTCCAGCGCCTGCTTGCCGCCATCGTGGCTGGCGATGGCCACGACCT

GGTCCGGGGTCAGGCCATGGTCCTGGCACAGCACCGGCAACAGCCGTTGCACCGTCTCCAGCGCCTGCTTGCCGCCATCG

TGGCTGGCGATGGCCACCACCTGGTCCAGGGTCAGGCCATGGTCCTGGCACAGCACCGGCAACAGCCGCTGCACCGTCTC

CAGCGCCTGCTTGCCGCCAATATTGCTGGCAATGGCCACCACCTGGTCCGGGGTCAGGCCATGGTCCTGGCACAGCACCG

GCAACAGCCGCTGCACCGTCTCCAGCGCCTGCTTGCCGCCATCGTGGCTGGCGATGGCCACCACCTGGTCCGAGGTCAGG

CCATGGTCCTGGCACAGCACCGGCAACAGCCGCTGCACCGTCTCCAGCGCCTGCTTGCCGCCGCCATGGCTGGCGATGGC

CACCACCTGGTCCAGGGTCAGGCCATGGTCCTGGCACAGCACCGGCAACAGCCGCTGCACCGTCTCCAGCGCCTGCTTGC

CGCCATCGTGGCTGGC

>M13F

GGATCCCATTCGTTCGCGCACGCCAAGTCCTGCCCGCGAGCTTCTGCCCGGACCCCAACCGGATAGGGTTCAGCCGACTG

CAGATCGGGGGGGGACTCCGCCTGCTGGCGGCCCCCTGGATGGCTTGCCCGTTCGGCGGACGATGTCCCGGACCCGGCTG

CCATCTCCCCCTGCACCCTCGCCTGCGTTCTCGGCGGGCAGCTTCAGCGATCTGCTCCGTCAGTTCGATCCGTCGCTTCT

TGATACATCGCTTCTTGATTCGATGCCTGCCGTCGGCACGCCGCATACAGCGGCTGCCCCAGCAGAGTGGGATGAGGTGC

AATCGGGTCTGCGTGCAGCCGATGACCCGCCACCCACTGTGCGTGTCGCTGTCACTGCCGCGCGGCCGCCGCGCGCCAAG

CCGGCCCCGCGACGGCGTGCGGCGCAACCCTCCGACGCTTCGCCGGCCGCGCAGGTGGATCTACGCACGCTCGGCTACAG

TCAGCAGCAGCAAGAGAAGATCAAACCGAAGGTGCGTTCGACAGTGGCGCAGCACCACGAGGCACTGGTGGGCCATGGGT

TTACACACGCGCACATCGTTGCGCTCAGCCAACACCCGGCAGCGTTAGGGACCGTTGCTGTCACGTATCAGGACATAATC

AGGGCGTTGCCAGAGGCGACACACGAAGACATCGTTGGCGTCGGCAAACAGTGGTCCGGCGCACGCGCCCTGGAGGCCTT

GCTCACGGAGGCGAGGGAGTTGAGAGGTCCGCCGTTACAGTTGGACACAGGCCAACTTCTCAAGATTGCAAAACGTGGCG

GCGTGACCGCAGTGGAGGCAGTGCATGCATGGCGCAATGCACTGACGGGTGCCCCCCTGAACCTGACCCCGGACCAAGTG

GTGGCCATCGCCAGCAATATTGGCGGCAACCAGGCGCTGGAGACGGTACAGCGGCTGTTGCCGGTGCTGTGCCAGGCCCA

T

>M13R

AGGTGGTGGCCATCGCCAGCCACGATGGCGGCAAGCAGGCGCTGGAGACGGTGCAACGGCTGTTGCCGGTGCTGTGCCAG

GACCATGGCCTGACCCCGGACCAGGTCGTGGCCATCGCCAGCCACGATGGCGGCAAGCAGGCGCTGGAGACGGTGCAGCG

GCTGTTGCCGGTGCTGTGCCAGGACCATGGCCTGACCCCGGCCCAGGTGGTGGCCATCGCCAGCAATGGCGGCGGCAAGC

AGGCGCTGGAGAGCATTGTTGCCCAGTTATCTCGCCCTGATCCGGCGTTGGCCGCGTTGACCAACGACCACCTCGTCGCC

TTGGCCTGCCTCGGCGGACGTCCTGCCCTGGATGCAGTGAAAAAGGGATTGCCGCACGCGCCGGAATTGATCAGAAGAAT

CAATCGCCGTATTCCCGAACGCACGTCCCATCGCGTTGCCGACTACGCGCAAGTGGTTCGCGTGCTGGAGTTTTTCCAGT

GCCACTCCCACCCAGCGTACGCATTTGATGAGGCCATGACGCAGTTCGGGATGAGCAGGCACGGGTTGGTACAGCTCTTT

CGCAGAGTGGGCGTCACCGAATTCGAAGCCCGCTGCGGAACGCTCCCCCCAGCCTCGCAGCGTTGGGACCGTATCCTCCA

GGCATCAGGGATGAAAAGGGCCAAACCGTCCCCTACTTCAGCTCAAACACCGGATCAGGCGTCTTTGCATGCATTCGCCG

ATTCGCTGGAGCGTGACCTTGATGCGCCCAGCCCAATGCACGAGGGAGATCAGACGCGGGCAAGCAGCCGTAAACGGTCC

CGATCGGATCGTGCTGTCACCGGCCCCTCCACACAGCAATCTTTCGAGGTGCGCGTTCCCGAACAGCGCGATGCGCTGCA

TTTGCCCCTCAGCTGGAGGGTAAAACGCCCGCGTACCAGGATCGGGGGCGGCCTCCCGGATCC

**pTAL*_Bam_*_HI_-21**

> pCC2FOS-MscI-1

GCGCTGGAGACGGTGCAGCGGCTGTTGCCGGTGCTGTGCCAGGCCCATGGCCTGACCCCGGACCAGGTCGTGGCCATCGC

CAGCAATATTGGCGGCAAGCAGACGCTGGAGACGGTGCAGCGGCTGTTGCCGGTGCTGTGCCAGGACCATGGCCTAACCC

CGGACCAGGTCGTGGCCATCGCCAACAATAACGGCGGCAAGCAGGCGCTGGAGACGGTGCAGCGGCTGTTGCCGGTGCTG

TGCCAGGACCATGGCCTGACCCCGGACCAGGTCGTGGCCATCGCCAGCAATAGTGGCGGCAAGCAGGCGCTGGAGACGGT

GCAGCGGCTGTTGCCGGTGCTGTGCCAGGACCATGGCCTGACCCCGAACCAGGTGGTGGCCATCGCCAGCAATGGCGGCG

GCAAGCAGGCGCTGGAGACGGTGCAGCGGCTGTTGCCGGTGCTGTGCCAGGACCATGGCCTGACCCCGGACCAGGTCGTG

GGCATCGCCAGCAATAGTGGCGGCAAGCAGGCGCTGGAGACGGTGCAGCGGCTGTTGCCGGTGCTGTGCCAGGACCATGG

CCTGACCCCGGACCAGGTCGTGGCCATCGCCAACAATAACGGTGGCAAGCAGGCGCTGGAGACGGTGCAGCGGCTGTTGC

CGGTGCTGTGCCAGGACCATGGCCTGACCCCGGACCAGGTGGTGGCCATCGCCAGCAATATTGGCGGCAAGCAGGCGCTG

GAGACGGTGCAGCGGCTGTTGCCGGTGCTGTGCCAGGACCATGGCCTGACCCTGGACAAGGTGGTGG

> pCC2FOS-MscI-2

GCGGCAAGCAGGCGCTGGAGACGGTGCAGCGGCTGTTGCCGGTGCTGTGCCAGGACCATGGCCTGACCCCGGACCAAGTG

GTGGCCATCGCCAACAATAAAGGCGGCAAGCAGGCGCTGGAGACGCTGCAGCGGCTGTTGCCGGTGCTGTGCCAGGCCCA

TGGCCTGACCCCGGACCAGGTGGTGGCCATCGCCAGCAATGGCGGCGGCAAGCAGGCGCTGGAGACGGTGCAACGGCTGT

TGCCGGTGCTGTGCCAGGATCATGGCCTGACCCCGGCCCAGGTCGTGGCCATCGCCAGCAATATTGGCGGCAAGCAGGCG

CTGGAGACGGTGCAGCGGCTGTTGCCGGTGCTGTGCCAGGACCATGGCCTGACCCCGGACCAAGTGGTGGCCATCGCCAA

CAATAACGGCGGCAAGCAGGCGCTGGAGACGGTGCAGCGGCTGTTGCCGGTGCTGTGCCAGGACCATGGCCTGACCCCGG

ACCAGGTCGTGACCATCGCCAGCAATATTGGCGGCAAGCAGGCGCTGGAGACGGTGCAGCGGCTGTTGCCGGTGCTGTGC

CAGGACCATGGCCTGACCCCGGACCAAGTGGTGGCCATCGCCAACAATAACGGCGGCAAGCAGGCGCTGGAGACGGTGCA

GCGGCTGTTGCCGGTGCTGTGCCAGGCCCATGGCCTGACCCCGGACCAGGTCGTGGCCATCGCCAGCAATATTGGCGGCA

AGCAGACGCTGGAGACGGTGCAGCGGCTGTTGCCGGTGCTGTGCCAGGACCATGGCCTAACCCCGGACCAGGTCGTGG

> pCC2FOS-MscI-3

AGGCGCTGGAGACGGTGCAGCGGCTGTTGCCGGTGCTGTGCCAGGACCATGGCCTGACCCCGGACCAGGTCGTGACCATC

GCCAGCAATATTGGCGGCAAGCAGGCGCTGGAGACGGTGCAGCGGCTGTTGCCGGTGCTGTGCCAGGACCATGGCCTGAC

CCCGGACCAAGTGGTGGCCATCGCCAACAATAACGGCGGCAAGCAGGCGCTGGAGACGGTGCAGCGGCTGTTGCCGGTGC

TGTGCCAGGCCCATGGCCTGACCCCGGACCAGGTCGTGGCCATCGCCAGCAATATTGGCGGCAAGCAGACGCTGGAGACG

GTGCAGCGGCTGTTGCCGGTGCTGTGCCAGGACCATGGCCTAACCCCGGACCAGGTCGTGGCCATCGCCAACAATAACGG

CGGCAAGCAGGCGCTGGAGACGGTGCAGCGGCTGTTGCCGGTGCTGTGCCAGGACCATGGCCTGACCCCGGACCAGGTCG

TGGCCATCGCCAGCAATAGTGGCGGCAAGCAGGCGCTGGAGACGGTGCAGCGGCTGTTGCCGGTGCTGTGCCAGGACCAT

GGCCTGACCCCGAACCAGGTGGTGGCCATCGCCAGCAATGGCGGCGGCAAGCAGGCGCTGGAGACGGTGCAGCGGCTGTT

GCCGGTGCTGTGCCAGGACCATGGCCTGACCCCGGACCAGGTCGTGGGCATCGCCAGCAATAGTGGCGGCAAGCAGGCGC

TGGAGACGGTGCAGCGGCTGTTGCCGGTGCTGTGCCAGGACCATGGCCTGACCCCGGACCAGGTCGTGG

>21P1F_1

CGAGGGAGTTGAGAGGTCCGCCGTTACAGTTGGACACAGGCCAACTTCTCAAGATTGCAAAACGTGGCGGCGTGACCGCA

GTGGAGGCAGTGCATGCATGGCGCAATGCACTGACGGGTGCCCCCCTGAACCTGACCCCGGACCAAGTGGTGGCCATCGC

CAGCAATATTGGCGGCAAGCAGGCGCTGGAGACGGTGCAGCGGCTGTTGCCGGTGCTGTGCCAGGACCATGGCCTGACCC

CGGACCAGGTCGTGGCCATCGCCAGCAATGGCGGCGGCAAGCAGGCGCTGGAGACGGTGCAGCGGCTGTTGCCGGTGCTG

TGCCAGGACCATGGCCTGACCCCGGACCAGGTCGTGGCCATCGCCAGCAATAACGGCGGCAAGCAGGCGCTGGAGACGGT

GCAGCGGCTGTTGCCGGTGCTGTGCCAGGACCATGGCCTGACCCCGGACCAGGTCGTGGCCATCGCCAGCAATGGCGGCG

GCAAGCAGGCGCTGGAGACGGTGCAGCGGCTGTTGCCGGTGCTGTGCCAGGACCATGGCCTGACCCCGGACCAAGTGGTG

GCCATCGCCAACAATAAAGGCGGCAAGCAGGCGCTGGAGACGCTGCAGCGGCTGTTGCCGGTGCTGTGCCAGGCCCATGG

CCTGACCCCGGACCAGGTGGTGGCCATCGCCAGCAATGGCGGCGGCAAGCAGGCGCTGGAGACGGTGCAACGGCTGTTGC

CGGTGCTGTGCCAGGATCATGGCCTGACCCCGGCCCAGGTCGTGGCCATCGCCAGCAATATTGGCGGCAAGCAGGCGCTG

GAGACGGTGCAGCGGCTGTTGCCGGTGCTGTGCCAGGACCATGGCCTGACCCCGGACCAAGTGGTGGCCATCGCCAACAA

TAACGGCGGCAAGCAGGCGCTGGAGACGGTGCAGCGGCTGTTGCCGGTGCTGTGCCAGGACCATGGCCTGACCCCGGACC

AGGTCGTGACCATCGCCAGC

>21P1F_2

GGGAGTTGAGAGGTCCGCCGTTACAGTTGGACACAGGCCAACTTCTCAAGATTGCAAAACGTGGCGGCGTGACCGCAGTG

GAGGCAGTGCATGCATGGCGCAATGCACTGACGGGTGCCCCCCTGAACCTGACCCCGGACCAAGTGGTGGCCATCGCCAG

CAATATTGGCGGCAAGCAGGCGCTGGAGACGGTGCAGCGGCTGTTGCCGGTGCTGTGCCAGGACCATGGCCTGACCCCGG

ACCAGGTCGTGGCCATCGCCAGCAATGGCGGCGGCAAGCAGGCGCTGGAGACGGTGCAGCGGCTGTTGCCGGTGCTGTGC

CAGGACCATGGCCTGACCCCGGACCAGGTCGTGGCCATCGCCAGCAATAACGGCGGCAAGCAGGCGCTGGAGACGGTGCA

GCGGCTGTTGCCGGTGCTGTGCCAGGACCATGGCCTGACCCCGGACCAGGTCGTGGCCATCGCCAGCAATGGCGGCGGCA

AGCAGGCGCTGGAGACGGTGCAGCGGCTGTTGCCGGTGCTGTGCCAGGACCATGGCCTGACCCCGGACCAAGTGGTGGCC

ATCGCCAACAATAAAGGCGGCAAGCAGGCGCTGGAGACGCTGCAGCGGCTGTTGCCGGTGCTGTGCCAGGCCCATGGCCT

GACCCCGGACCAGGTGGTGGCCATCGCCAGCAATGGCGGCGGCAAGCAGGCGCTGGAGACGGTGCAACGGCTGTTGCCGG

TGCTGTGCCAGGATCATGGCCTGACCCCGGCCCAGGTCGTGGCCATCGCCAGCAATATTGGCGGCAAGCAG

>21P1R_1

GGGCAACAATGCTCTCCAGCGCCTGCTTGCCGCCGCCATTGCTGGCGATGGCCACGACCTGGTCCGGGGTCAGGCCATGG

TCCTGGCACAGCACCGGCAACAGCCGCTGCACCGTCTCCAGCGCCTGCTTGCCGCCACTATTGCTGGCGATGGCCACGAC

CTGGTCCGGGGTCAGGCCATGGTCCTGGCACAGCACCGGCAACAGCCGCTGCACCGTCTCCAGCGCCTGCTTGCCGCCAT

TGCTGGCGATGGCCACCACCTTGTCCAGGGTCAGGCCATGGTCCTGGCACAGCACCGGCAACAGCCGCTGCACCGTCTCC

AGCGCCTGCTTGCCGCCAATATTGCTGGCGATGGCCACCACCTGGTCCGGGGTCAGGCCATGGTCCTGGCACAGCACCGG

CAACAGCCGCTGCACCGTCTCCAGCGCCTGCTTGCCACCGTTATTGTTGGCGATGGCCACGACCTGGTCCGGGGTCAGGC

CATGGTCCTGGCACAGCACCGGCAACAGCCGCTGCACCGTCTCCAGCGCCTGCTTGCCGCCACTATTGCTGGCGATGCCC

ACGACCTGGTCCGGGGTCAGGCCATGGTCCTGGCACAGCACCGGCAACAGCCGCTGCACCGTCTCCAGCGCCTGCTTGCC

GCCGCCATTGCTGGCGATGGCCACCACCTGGTTCGGGGTCAGGCCATGGTCCTGGCACAGCACCGGCAACAGCCGCTGCA

CCGTCTCCAGCGCCTGCTTGCCGCCACTATTGCTGGCGATGGCCACGACCTGGTCCGGGGTCAGGCCATGGTCCTGGCAC

AGCACCGGCAACAGCCGCTGCACCGTCTCCAGCGCCTGCTTGCCGCCGTTATTGTTGGCGATGGCCACGACCTGGTCCGG

GGTTAGGCCATGGTCCTGGCACAGCACCGGCAACAGCCGCTGCACCGTCTCCAGCGTCTGCTTGCCGCCAATATTGCTGG

CGATGGCCACGAC

>21P1R_2

TCGCCAGCAATAGTGGCGGCAAGCAGGCGCTGGAGACGGTGCAGCGGCTGTTGCCGGTGCTGTGCCAGGACCATGGCCTG

ACCCCGAACCAGGTGGTGGCCATCGCCAGCAATGGCGGCGGCAAGCAGGCGCTGGAGACGGTGCAGCGGCTGTTGCCGGT

GCTGTGCCAGGACCATGGCCTGACCCCGGACCAGGTCGTGGGCATCGCCAGCAATAGTGGCGGCAAGCAGGCGCTGGAGA

CGGTGCAGCGGCTGTTGCCGGTGCTGTGCCAGGACCATGGCCTGACCCCGGACCAGGTCGTGGCCATCGCCAACAATAAC

GGTGGCAAGCAGGCGCTGGAGACGGTGCAGCGGCTGTTGCCGGTGCTGTGCCAGGACCATGGCCTGACCCCGGACCAGGT

GGTGGCCATCGCCAGCAATATTGGCGGCAAGCAGGCGCTGGAGACGGTGCAGCGGCTGTTGCCGGTGCTGTGCCAGGACC

ATGGCCTGACCCTGGACAAGGTGGTGGCCATCGCCAGCAATGGCGGCAAGCAGGCGCTGGAGACGGTGCAGCGGCTGTTG

CCGGTGCTGTGCCAGGACCATGGCCTGACCCCGGACCAGGTCGTGGCCATCGCCAGCAATAGTGGCGGCAAGCAGGCGCT

GGAGACGGTGCAGCGGCTGTTGCCGGTGCTGTGCCAGGACCATGGCCTGACCCCGGACCAGGTCGTGGCCATCGCCAGCA

ATGGCGGCGGCAAGCAGGCGCTGGAGAGCATTGTTGCCCAG

>21P2F_1

CGCGCACATCGTTGCGCTCAGCCAACACCCGGCAGCGTTAGGGACCGTCGCTGTCAAGTATCAGCACATAATCACGGCGT

TGCCAGAGGCGACACACGAAGACATCGTTGGCGTCGGCAAACAGTGGTCCGGCGCACGCGCCCTGGAGGCCTTGCTCACG

GAGGCGAGGGAGTTGAGAGGTCCGCCGTTACAGTTGGACACAGGCCAACTTCTCAAGATTGCAAAACGTGGCGGCGTGAC

CGCAGTGGAGGCAGTGCATGCATGGCGCAATGCACTGACGGGTGCCCCCCTGAACCTGACCCCGGACCAAGTGGTGGCCA

TCGCCAGCAATATTGGCGGCAAGCAGGCGCTGGAGACGGTGCAGCGGCTGTTGCCGGTGCTGTGCCAGGACCATGGCCTG

ACCCCGGACCAGGTCGTGGCCATCGCCAGCAATGGCGGCGGCAAGCAGGCGCTGGAGACGGTGCAGCGGCTGTTGCCGGT

GCTGTGCCAGGACCATGGCCTGACCCCGGACCAGGTCGTGGCCATCGCCAGCAATAACGGCGGCAAGCAGGCGCTGGAGA

CGGTGCAGCGGCTGTTGCCGGTGCTGTGCCAGGACCATGGCCTGACCCCGGACCAGGTCGTGGCCATCGCCAGCAATGGC

GGCGGCAAGCAGGCGCTGGAGACGGTGCAGCGGCTGTTGCCGGTGCTGTGCCAGGACCATGGCCTGACCCCGGACCAAGT

GGTGGCCATCGCCAACAATAAAGGCGGCAAGCAGGCGCTGGAGACGCTGCAGCGGCTGTTGCCGGTGCTGTGCCAGGCCC

ATGGCCTGACCCCGGACCAGGTGGTGGCCATCGCCAGCAATGGCGGCGGCAAGCAGGCGCTGGAGACGGTGCAACGGCTG

TTGCCGGTGCTGTGCCAGGATCATGGCCTGACCCCGGCCCAGGTCGTGGCCATCGCCAGCAATATTGGCGGCAAGCAGGC

GCTGGAGACGGTGC

>21P2F_2

CGTTGCGCTCAGCCAACACCCGGCAGCGTTAGGGACCGTCGCTGTCAAGTATCAGCACATAATCACGGCGTTGCCAGAGG

CGACACACGAAGACATCGTTGGCGTCGGCAAACAGTGGTCCGGCGCACGCGCCCTGGAGGCCTTGCTCACGGAGGCGAGG

GAGTTGAGAGGTCCGCCGTTACAGTTGGACACAGGCCAACTTCTCAAGATTGCAAAACGTGGCGGCGTGACCGCAGTGGA

GGCAGTGCATGCATGGCGCAATGCACTGACGGGTGCCCCCCTGAACCTGACCCCGGACCAAGTGGTGGCCATCGCCAGCA

ATATTGGCGGCAAGCAGGCGCTGGAGACGGTGCAGCGGCTGTTGCCGGTGCTGTGCCAGGACCATGGCCTGACCCCGGAC

CAGGTCGTGGCCATCGCCAGCAATGGCGGCGGCAAGCAGGCGCTGGAGACGGTGCAGCGGCTGTTGCCGGTGCTGTGCCA

GGACCATGGCCTGACCCCGGACCAGGTCGTGGCCATCGCCAGCAATAACGGCGGCAAGCAGGCGCTGGAGACGGTGCAGC

GGCTGTTGCCGGTGCTGTGCCAGGACCATGGCCTGACCCCGGACCAGGTCGTGGCCATCGCCAGCAATGGCGGCGGCAAG

CAGGCGCTGGAGACGGTGCAGCGGCTGTTGCCGGTGCTGTGCCAGGACCATGGCCTGACCCCGGACCAAGTGGTGGCCAT

CGCCAACAATAAAGGCGGCAAGCAGGCGCTGGAGACGCTGCAGCGGCTGTTGCCGGTGCTGTGCCAGGCCCATGGCCTGA

CCCCGGACCAGGTGGTGGCCATCGCCAGCAATGGCGGCGGCAAGCAGGCGCTGGAGACGGTGCAACGGCTGTTGCCGGTG

CTGTGCCAGGATCATGGCCTGACCCCGGCCCAGGTCGTGGCCATCGCCAGCAATATTGGCGGCAAGCAGGCGCTGGAGAC

GGTGCA

>21P2R_1

CCGTGATTATGTGCTGATACTTGACAGCGACGGTCCCTAACGCTGCCGGGTGTTGGCTGAGCGCAACGATGTGCGCGTGT

GTAAACCCATGGCCCACCAGTGCCTCGTGGTGCTGCGCCACTGTCGAACGCACCTTCGGTTTGATCTTCTCTTGCTGCTG

CTGACTGTAGCCGAGCGTGCGTAGATCCACCTGCGCGGCCGGCGAAGCGTCGGAGGGTTGCGCCGCACGCCGTCGCGGGG

CCGGCTTGGCGCGCGGCGGCCGCGCGGCAGTGACAGCGACACGCACGGTGGGTGGCGGGTCATCGGCTGCACGCAGACCC

GATTGCACCTCATCCCACTCTGCTGGGGCAGCCGCTGTATGCGGCGTGCCGACGGCAGGCATCGAATCAAGAAGCGATGT

ATCAAGAAGCGACGGATCGAACTGACGGAGCAGATCGTTGAAGCTGCCCGCCGAGAACGCAGGCGAGGGCGCAGGGGGAG

ATGGCAGCCGGGTCCGGGACATCGTCCGCCGAGCGGGCAAGCCATCCAGGGGGCCGCCAGCAGGCGGAGCCCCCCCCCGA

TCTGCAGTCGGCTGAACCCTATCCGGTTGGGGTCCGGGCAGAAGCTCGCGGGCAGGACTTGGCGTGCGCGAACGAATGGG

ATCC

>21P2R_2

GGATCCCATTCGTTCGCGCACGCCAAGTCCTGCCCGCGAGCTTCTGCCCGGACCCCAACCGGATAGGGTTCAGCCGACTG

CAGATCGGGGGGGGGCTCCGCCTGCTGGCGGCCCCCTGGATGGCTTGCCCGCTCGGCGGACGATGTCCCGGACCCGGCTG

CCATCTCCCCCTGCGCCCTCGCCTGCGTTCTCGGCGGGCAGCTTCAACGATCTGCTCCGTCAGTTCGATCCGTCGCTTCT

TGATACATCGCTTCTTGATTCGATGCCTGCCGTCGGCACGCCGCATACAGCGGCTGCCCCAGCAGAGTGGGATGAGGTGC

AATCGGGTCTGCGTGCAGCCGATGACCCGCCACCCACCGTGCGTGTCGCTGTCACTGCCGCGCGGCCGCCGCGCGCCAAG

CCGGCCCCGCGACGGCGTGCGGCGCAACCCTCCGACGCTTCGCCGGCCGCGCAGGTGGATCTACGCACGCTCGGCTACAG

TCAGCAGCAGCAAGAGAAGATCAAACCGAAGGTGCGTTCGACAGTGGCGCAGCACCACGAGGCACTGGTGGGCCATGGGT

TTACACACGCGCACATCGTTGCGCTCAGCCAACACCCGGCAGCGTTAGGGACCGTCGCTGTCAAGTATCAGCACAT

>21P3F

GGATTGCCGCACGCGCCGGAATTGATCAGAAGAGTCAATCGCCGTATTGGCGAACGCACGTCCCATCGCGTTGCCGACTA

CGCGCAAGTGGTTCGCGTGCTGGAGTTTTTCCAGTGCCACTCCCACCCAGCGTACGCATTTGATGAGGCCATGACGCAGT

TCGGGATGAGCAGGAACGGGTTGGTACAGCTCTTTCGCAGAGTGGGCGTCACCGAACTCGAAGCCCGCGGTGGAACGCTC

CCCCCAGCCTCGCAGCGTTGGGACCGTATCCTCCAGGCATCAGGGATGAAAAGGGCCAAACCGTCCCCTACTTCAGCTCA

AACACCGGATCAGGCGTCTTTGCATGCATTCGCCGATTCGCTGGAGCGTGACCTTGATGCGCCTAGCCCAATGCACGAGG

GAGATCAGACAGGGGCAAGCAGCCGTAAACGGTCCCGATCGGATCGTGCTGTCACCGGCCCCTCCGCACAGCAATCTTTC

GAGGTGCGCGTTCCCGAACAGCGCGATGCGCTGCATTTGCCCCTCAGCTGGAGGGTAAAACGCCCGCGTACCAGGATCGG

GGGCGGCCTCCCGGATCC

>21P3R

TTCGAGTTCGGTGACGCCCACTCTGCGAAAGAGCTGTACCAACCCGTTCCTGCTCATCCCGAACTGCGTCATGGCCTCAT

CAAATGCGTACGCTGGGTGGGAGTGGCACTGGAAAAACTCCAGCACGCGAACCACTTGCGCGTAGTCGGCAACGCGATGG

GACGTGCGTTCGCCAATACGGCGATTGACTCTTCTGATCAATTCCGGCGCGTGCGGCAATCCCTTTTTCACTGCATCCAT

GGCAGGACGTCCGCCGAGGCAGGCCAAGGCGACGAGGTGGTCGTTGGTCAACGCGGCCAACGCCGGATCAGGGCGAGATA

ACTGGGCAACAATGCTCTCCAGCGCCTGCTTGCCGCCGCCATTGCTGGCGATGGCCACGACCTGGTCCGGGGTCAGGCCA

TGGTCCTGGCACAGCACCGGCAACAGCCGCTGCACCGTCTCCAGCGCCTGCTTGCCGCCACTATTGCTGGCGATGGCCAC

GACCTGGTCCGGGGTCAGGCCATGGTCCTGGCACAGCACCGGCAACAGCCGCTGCACCGTCTCCAGCGCCTGCTTGCCGC

CATTGCTGGCGATGGCCACCACCTTGTCCAGGGTCAGGCCATGGTCCTGGCACAGCACCGGCAACAGCCGCTGCACCGTC

TCCAGCGCCTGCTTGCCGCCAATATTGCTGGCGATGGCCACCACCTGGTCCGGGGTCAGGCCATGGTCCTGGCACAGCAC

CGGCAACAGCCGCTGCACCGTCTCCAGCGCCTGCTTGCCACCGTTATTGTTGGCGATGGCCACGACCTGGTCCGGGGTCA

GGCCATGGTCCTGGCACAGCACCGGCAACAGCCGCTGCACCGTCTCCAGCGCCTGCTTGCCGCCACTATTGCTGGCGATG

CCCACGACCTGGTCCGGGGTCAGGCCATGGTCCTGGCACAGCACCGGCAACAGCCGCTGCACCGTCTCCAGCGCCTGCTT

GCCGCCGCCATTGCTGGC

>M13R

CCGGACCAGGTCGTGGCCATCGCCAGCAATAGTGGCGGCAAGCAGGCGCTGGAGACGGTGCAGCGGCTGTTGCCGGTGCT

GTGCCAGGACCATGGCCTGACCCCGGACCAGGTCGTGGCCATCGCCAGCAATGGCGGCGGCAAGCAGGCGCTGGAGAGCA

TTGTTGCCCAGTTATCTCGCCCTGATCCGGCGTTGGCCGCGTTGACCAACGACCACCTCGTCGCCTTGGCCTGCCTCGGC

GGACGTCCTGCCATGGATGCAGTGAAAAAGGGATTGCCGCACGCGCCGGAATTGATCAGAAGAGTCAATCGCCGTATTGG

CGAACGCACGTCCCATCGCGTTGCCGACTACGCGCAAGTGGTTCGCGTGCTGGAGTTTTTCCAGTGCCACTCCCACCCAG

CGTACGCATTTGATGAGGCCATGACGCAGTTCGGGATGAGCAGGAACGGGTTGGTACAGCTCTTTCGCAGAGTGGGCGTC

ACCGAACTCGAAGCCCGCGGTGGAACGCTCCCCCCAGCCTCGCAGCGTTGGGACCGTATCCTCCAGGCATCAGGGATGAA

AAGGGCCAAACCGTCCCCTACTTCAGCTCAAACACCGGATCAGGCGTCTTTGCATGCATTCGCCGATTCGCTGGAGCGTG

ACCTTGATGCGCCTAGCCCAATGCACGAGGGAGATCAGACAGGGGCAAGCAGCCGTAAACGGTCCCGATCGGATCGTGCT

GTCACCGGCCCCTCCGCACAGCAATCTTTCGAGGTGCGCGTTCCCGAACAGCGCGATGCGCTGCATTTGCCCCTCAGCTG

GAGGGTAAAACGCCCGCGTACCAGGATCGGGGGCGGCCTCCCGGATCC

**pTAL*_Bam_*_HI_-22**

> pCC2FOS-MscI-1

CGGTGCAGCGGCTGTTGCCGGTGCTGTGCCAGGCCCATGGCTTGACCCCGGCGCAGGTGGTGGCCATCGCCAGCCACGAT

GGCGGCAAGCAGGCGCTGGAGACGGTGCAGCGGCTGTTGCCGGTGCTGTGCCAGGCCCATGGTCTGACCCCAGCGCAGGT

GGTGGCCATCGCCAGCAATAACGGCGGCAAGCAGGCGCTGGAGACGGTGCAGCGGCTGTTGCCGGTGCTGTGCCAGGCCC

ATGGCCTGACCCCGGACCAGGTCGTGGCCATCGCCAGCAATAACGGCGGCAAGCAGGCGCTGGAGACGGTGCAGCGGCTG

TTGCCGGTGCTGTGCCAGGCCCATGGCCTGACCCCAGACCAGGTCGTGGCCATCGCCAGCAATATTGGCGGCAAGCAGGC

GCTGGAGACGGTGCAGCGGCTGTTGCCGGTGCTGTGCCAGGACCATGGCCTGACCCCGGACCAGGTCGTGGCCATCGCCA

GCAATGGCGGCGGCAAGCAGGCGCTGGAGACGGTGCAGCGGCTGTTGCCGGTGCTGTGCCAGGCCCATGGCCTGCCCCCG

GCGCAGGTGGTGGCCATCGCCAGCCACGATGGCGGCAAGCAGGCGCTGGAGACGGTGCAGCGGCTGTTGCCGGTGCTGTG

CCAGGACCATGGCCTGACCCCGGCGCAGGTGGTGGCCATCGCCAGCAGTGGCGGCAAGCAGGCGCTGGAGACGGTGCAGC

GGCTGTTGCCGGTGCTGTGCCAGGCCCATGGCCTGACCCTGGACCAGGTGGTGGCCATCGCCAGCCATGGCGGCAGCAAG

CAGGCGCTGGAGACGGTGCAGCGGCTGTTGCCGGTGCTGTGCCAGGACCATGGCCTGACCCCGGACCAGGTGGTGG

> pCC2FOS-MscI-2

GGTGCAGCGGCTGTTGCCGGTGCTGTGCCAGGACCATGGCCTGACCCCGGACCAGGTGGTGGCCATCGCCAGCAATAACG

GCGGCAAGCAGGCGCTGGAGACGGTGCAGCGGCTGTTGCCGGTGCTGTGCCAGGACCATGGCCTGACCCTGGATCAGGTC

GTGGCCATCGCCAGCCACGATGGCGGCAAGCAGGCGCTGGAGACGGTGCAGCGGCTGTTGCCGGTGCTGTGCCAGGCCCA

TGGCCTGACCCCGGCGCAGGTGGTGGCCATCGCCAGCCATATTGGCGGCAAGCAGGCGCTGGAGACGGTGCAGCGGCTGT

TGCCGGTGCTGTGCCAGGACCATGGCCTGACCCTGGACCAGGTGGTGGCCATTGCCAGCAATGACGGCAGCAAGCAGGCG

CTGGAGACGGTGCAGCGGCTGTTGCCGGTGCTGTGCCAGGACCATGGCCTGACCCCGGACCAGGTGGTGGCCATCGCCAG

CCACGATGGCGGCAAGCAGGCGCTGGAGACGGTGCAGCGGCTGTTGCCGGTGCTGTGCCAGGACCATGGCCTGACCCCGG

ACCAGGTGGTGGCCATCGCCAGCAATGGCGGCGGCAAGCAGGCGCTGGAGACGGTGCAACGGCTGTTGCCGGTGCTGTGC

CAGGACCATGGCCTGACCCCGGCCCAGGTCGTGGCCATCGCCAACAATAACGGCGGCAAGCAGGCGCTGGAGACGGTGCA

GCGGCTGTTGCCGGTGCTGTGCCAGGACCATGGCCTGACCCCGGACCAGGTCGTGGCCATCGCCAGCCATGGCGGCGGCA

AGCAGGCGCTGGAGACGGTGCAGCGGCTGTTGCCGGTGCTGTGCCAGGACCATGGCCTGACCCCGGACCAGGTGGTGG

> pCC2FOS-MscI-3

GGTGCAGCGGCTGTTGCCGGTGCTGTGCCAGGACCATGGCCTGACCCCGGACCAGGTCGTGGCCATCGCCAGCAATGGCG

GCGGCAAGCAGGCGCTGGCGACGGTGCAGCGGCTGTTGCCGGTGCTGTGCCAGGCCCATGGCCTGACCCCGGACCAGGTC

GTGGCCATCGCCAGCCACGATGGCGGCAAGCAGGCGCTGGAGACGGTGCAGCGGCTGTTGCCGGTGCTGTGCCAGGACCA

TGGCCTGACCCCGGACCAGGTGGTGGCCATCGCCAGCCACGATGGCGGCAAGCAGGCGCTGGAGACGGTGCAGCGGCTGT

TGCCGGTGCTGTGCCAGGCCCATGGCCTGACCCCGGACCAGGTGGTGGCCATCGCCAGCAATGGCGGCAAGCAGGCGCTG

GAGACGGTGCAGCGGCTGTTGCCGGTGCTGTGCCAGGCCCATGGCCTGACCCTGGCGCAGGTGGTGGCCATCGCCAGCAA

TATTGGCGGCAAGCAGGCGCTGGAGACGGTGCAGCGGCTGTTGCCGGTGCTGTGCCAGGACCATGGCCTGACCCCGGACC

AGGTGGTGGCCATCGCCAGTAATATTGGCGGCAAGCAGGCGCTGGAGACGGTGCAGCGGCTGTTGCCGGTGCTGTGCCAG

GACCATGGCCTGACCCCGGACCAGGTGGTGGCCATCGCCAGCAATAACGGCGGCAAGCAGGCGCTGGAGACGGTGCAGCG

GCTGTTGCCGGTGCTGTGCCAGGACCATGGCCTGACCCTGGATCAGGTCGTGGCCATCGCCAGCCACGATGGCGGCAAGC

AGGCGCTGGAGACGGTGCAGCGGCTGTTGCCGGTGCTGTGCCAGGCCCATGGCCTGACCCCGGCGCAGGTGGTGGCCATC

GCCAGCCATATTGGCGGCAAGCAGGCGCTGGAGACGGTGCAGCGGCTGTTGCCGGTGCTGTGCCAGGACCATGGCCTGAC

CCTGGACCAG

> pCC2FOS-MscI-4

GCGGCAAGCAGGCGCTGGAGACGGTGCAACGGCTGTTGCCGGTGCTGTGCCAGGACCATGGCCTGACCCCGGACCAGGTG

GTGGCCATCGCCAGCAATGGCGGCAAGCAGGCGCTGGAGACGGTGCAGCGGCTGTTGCCGGTGCTGTGCCAGGACCATGG

CCTGACCCCGGACCAGGTCGTGGCCATCGCCAGCAATGGCGGCGGCAAGCAGGCGCTGGCGACGGTGCAGCGGCTGTTGC

CGGTGCTGTGCCAGGCCCATGGCCTGACCCCGGACCAGGTCGTGGCCATCGCCAGCCACGATGGCGGCAAGCAGGCGCTG

GAGACGGTGCAGCGGCTGTTGCCGGTGCTGTGCCAGGACCATGGCCTGACCCCGGACCAGGTGGTGGCCATCGCCAGCCA

CGATGGCGGCAAGCAGGCGCTGGAGACGGTGCAGCGGCTGTTGCCGGTGCTGTGCCAGGCCCATGGCCTGACCCCGGACC

AGGTGGTGGCCATCGCCAGCAATGGCGGCAAGCAGGCGCTGGAGACGGTGCAGCGGCTGTTGCCGGTGCTGTGCCAGGCC

CATGGCCTGACCCTGGCGCAGGTGGTGGCCATCGCCAGCAATATTGGCGGCAAGCAGGCGCTGGAGACGGTGCAGCGGCT

GTTGCCGGTGCTGTGCCAGGACCATGGCCTGACCCCGGACCAGGTGGTGGCCATCGCCAGTAATATTGGCGGCAAGCAGG

CGCTGGAGACGGTGCAGCGGCTGTTGCCGGTGCTGTGCCAGGACCATGGCCTGACCCCGGACCAGGTGGTGGCCATCGCC

AGCAATAACGGCGGCAAGCAGGCGCTGGAGACGGTGCAGCGGCTGTTGCCGGTGCTGTGCCAGGACCATGGCCTGACCCT

GGATCAGGTCGTGGCCATCGCCAGCCACGATGGCGGCAAGCAGGCGCTGGAGACGGTGCAGCGGCTGTTGCCGGTGCT

pCC2FOS-MscI->5

CCATCGCCAGCAATGGCGGCGGCAAGCAGGCGCTGGAGACGGTGCAACGGCTGTTGCCGGTGCTGTGCCAGGACCATGGC

CTGACCCCGGACCAGGTGGTGGCCATCGCCAGCAATGGCGGCAAGCAGGCGCTGGAGACGGTGCAGCGGCTGTTGCCGGT

GCTGTGCCAGGACCATGGCCTGACCCCGGACCAGGTCGTGGCCATCGCCAGCAATGGCGGCGGCAAGCAGGCGCTGGCGA

CGGTGCAGCGGCTGTTGCCGGTGCTGTGCCAGGCCCATGGCCTGACCCCGGACCAGGTCGTGGCCATCGCCAGCCACGAT

GGCGGCAAGCAGGCGCTGGAGACGGTGCAGCGGCTGTTGCCGGTGCTGTGCCAGGACCATGGCCTGACCCCGGACCAGGT

GGTGGCCATCGCCAGCCACGATGGCGGCAAGCAGGCGCTGGAGACGGTGCAGCGGCTGTTGCCGGTGCTGTGCCAGGCCC

ATGGCCTGACCCCGGACCAGGTGGTGGCCATCGCCAGCAATGGCGGCAAGCAGGCGCTGGAGACGGTGCAGCGGCTGTTG

CCGGTGCTGTGCCAGGCCCATGGCCTGACCCTGGCGCAGGTGGTGGCCATCGCCAGCAATATTGGCGGCAAGCAGGCGCT

GGAGACGGTGCAGCGGCTGTTGCCGGTGCTGTGCCAGGACCATGGCCTGACCCCGGACCAGGTGGTGGCCATCGCCAGTA

ATATTGGCGGCAAGCAGGCGCTGGAGACGGTGCAGCGGCTGTTGCCGGTGCTGTGCCAGGACCATGGCCTGACCCCGGAC

CAGGTGGTGGCCATCGCCAGCAATAACGGCGGCAAGCAGGCGCTGGAGACGGTGCAGCGGCTGTTGCCGGTGCTG

> pCC2FOS-MscI-6

CGGTGCAGCGGCTGTTGCCGGTGCTGTGCCAGGACCATGGCCTGACCCCGGACCAGGTGGTGGCCATCGCCAGCAATAAC

GGCGGCAAGCAGGCGCTGGAGACGGTGCAGCGGCTGTTGCCGGTGCTGTGCCAGGACCATGGCCTGACCCTGGATCAGGT

CGTGGCCATCGCCAGCCACGATGGCGGCAAGCAGGCGCTGGAGACGGTGCAGCGGCTGTTGCCGGTGCTGTGCCAGGCCC

ATGGCCTGACCCCGGCGCAGGTGGTGGCCATCGCCAGCCATATTGGCGGCAAGCAGGCGCTGGAGACGGTGCAGCGGCTG

TTGCCGGTGCTGTGCCAGGACCATGGCCTGACCCTGGACCAGGTGGTGGCCATTGCCAGCAATGACGGCAGCAAGCAGGC

GCTGGAGACGGTGCAGCGGCTGTTGCCGGTGCTGTGCCAGGACCATGGCCTGACCCCGGACCAGGTGGTGGCCATCGCCA

GCCACGATGGCGGCAAGCAGGCGCTGGAGACGGTGCAGCGGCTGTTGCCGGTGCTGTGCCAGGACCATGGCCTGACCCCG

GACCAGGTGGTGGCCATCGCCAGCAATGGCGGCGGCAAGCAGGCGCTGGAGACGGTGCAACGGCTGTTGCCGGTGCTGTG

CCAGGACCATGGCCTGACCCCGGCCCAGGTCGTGGCCATCGCCAACAATAACGGCGGCAAGCAGGCGCTGGAGACGGTGC

AGCGGCTGTTGCCGGTGCTGTGCCAGGACCATGGCCTGACCCCGGACCAGGTCGTGG

> pCC2FOS-MscI-7

CGGTGCAGCGGCTGTTGCCGGTGCTGTGCCAGGCCCATGGCCTGACCCTGGCGCAGGTGGTGGCCATCGCCAGCAATATT

GGCGGCAAGCAGGCGCTGGAGACGGTGCAGCGGCTGTTGCCGGTGCTGTGCCAGGACCATGGCCTGACCCCGGACCAGGT

GGTGGCCATCGCCAGTAATATTGGCGGCAAGCAGGCGCTGGAGACGGTGCAGCGGCTGTTGCCGGTGCTGTGCCAGGACC

ATGGCCTGACCCCGGACCAGGTGGTGGCCATCGCCAGCAATAACGGCGGCAAGCAGGCGCTGGAGACGGTGCAGCGGCTG

TTGCCGGTGCTGTGCCAGGACCATGGCCTGACCCTGGATCAGGTCGTGGCCATCGCCAGCCACGATGGCGGCAAGCAGGC

GCTGGAGACGGTGCAGCGGCTGTTGCCGGTGCTGTGCCAGGCCCATGGCCTGACCCCGGCGCAGGTGGTGGCCATCGCCA

GCCATATTGGCGGCAAGCAGGCGCTGGAGACGGTGCAGCGGCTGTTGCCGGTGCTGTGCCAGGACCATGGCCTGACCCTG

GACCAGGTGGTGGCCATTGCCAGCAATGACGGCAGCAAGCAGGCGCTGGAGACGGTGCAGCGGCTGTTGCCGGTGCTGTG

CCAGGACCATGGCCTGACCCCGGACCAGGTGGTGGCCATCGCCAGCCACGATGGCGGCAAGCAGGCGCTGGAGACGGTGC

AGCGGCTGTTGCCGGTGCTGTGCCAGGACCATGGCCTGACCCCGGACCAGGTGGTGGCCATCGCCAGCAATGGCGGCGGC

AAGCAGGCGCTGGAGACGGTGCAACGGCTGTTGCCGGTGCTGTGCCAGGACCATGGCCTGACCCCGGCCCAGGTCGTGG

> pCC2FOS-MscI-8

CGGTGCAGCGGCTGTTGCCGGTGCTGTGCCAGGCCCATGGCTTGACCCCGGCGCAGGTGGTGGCCATCGCCAGCCACGAT

GGCGGCAAGCAGGCGCTGGAGACGGTGCAGCGGCTGTTGCCGGTGCTGTGCCAGGCCCATGGTCTGACCCCAGCGCAGGT

GGTGGCCATCGCCAGCAATAACGGCGGCAAGCAGGCGCTGGAGACGGTGCAGCGGCTGTTGCCGGTGCTGTGCCAGGCCC

ATGGCCTGACCCCGGACCAGGTCGTGGCCATCGCCAGCAATAACGGCGGCAAGCAGGCGCTGGAGACGGTGCAGCGGCTG

TTGCCGGTGCTGTGCCAGGCCCATGGCCTGACCCCAGACCAGGTCGTGGCCATCGCCAGCAATATTGGCGGCAAGCAGGC

GCTGGAGACGGTGCAGCGGCTGTTGCCGGTGCTGTGCCAGGACCATGGCCTGACCCCGGACCAGGTCGTGGCCATCGCCA

GCAATGGCGGCGGCAAGCAGGCGCTGGAGACGGTGCAGCGGCTGTTGCCGGTGCTGTGCCAGGCCCATGGCCTGCCCCCG

GCGCAGGTGGTGGCCATCGCCAGCCACGATGGCGGCAAGCAGGCGCTGGAGACGGTGCAGCGGCTGTTGCCGGTGCTGTG

CCAGGACCATGGCCTGACCCCGGCGCAGGTGGTGGCCATCGCCAGCAGTGGCGGCAAGCAGGCGCTGGAGACGGTGCAGC

GGCTGTTGCCGGTGCTGTGCCAGGCCCATGGCCTGACCCTGGACCAGGTGGTGG

> pCC2FOS-MscI-9

GGTGCAGCGGCTGTTGCCGGTGCTGTGCCAGGCCCATGGCCTGACCCTGGCGCAGGTGGTGGCCATCGCCAGCAATATTG

GCGGCAAGCAGGCGCTGGAGACGGTGCAGCGGCTGTTGCCGGTGCTGTGCCAGGACCATGGCCTGACCCCGGACCAGGTG

GTGGCCATCGCCAGTAATATTGGCGGCAAGCAGGCGCTGGAGACGGTGCAGCGGCTGTTGCCGGTGCTGTGCCAGGACCA

TGGCCTGACCCCGGACCAGGTGGTGGCCATCGCCAGCAATAACGGCGGCAAGCAGGCGCTGGAGACGGTGCAGCGGCTGT

TGCCGGTGCTGTGCCAGGACCATGGCCTGACCCTGGATCAGGTCGTGGCCATCGCCAGCCACGATGGCGGCAAGCAGGCG

CTGGAGACGGTGCAGCGGCTGTTGCCGGTGCTGTGCCAGGCCCATGGCCTGACCCCGGCGCAGGTGGTGGCCATCGCCAG

CCATATTGGCGGCAAGCAGGCGCTGGAGACGGTGCAGCGGCTGTTGCCGGTGCTGTGCCAGGACCATGGCCTGACCCTGG

ACCAGGTGGTGGCCATTGCCAGCAATGACGGCAGCAAGCAGGCGCTGGAGACGGTGCAGCGGCTGTTGCCGGTGCTGTGC

CAGGACCATGGCCTGACCCCGGACCAGGTGGTGGCCATCGCCAGCCACGATGGCGGCAAGCAGGCGCTGGAGACGGTGCA

GCGGCTGTTGCCGGTGCTGTGCCAGGACCATGGCCTGACCCCGGACCAGGTGGTGGCCATCGCCAGCAATGGCGGCGGCA

AGCAGGCGCTGGAGACGGTGCAACGGCTGTTGCCGGTGCTGTGCCAGGACCATGGCCTGACCCCGGCCCAGGTCGTGG

> pCC2FOS-MscI-10

GGTGCAGCGGCTGTTGCCGGTGCTGTGCCAGGCCCATGGCCTGACCCCGGACCAGGTCGTGGCCATCGCCAGCAATAACG

GCGGCAAGCAGGCGCTGGAGACGGTGCAGCGGCTGTTGCCGGTGCTGTGCCAGGCCCATGGCCTGACCCCAGACCAGGTC

GTGGCCATCGCCAGCAATATTGGCGGCAAGCAGGCGCTGGAGACGGTGCAGCGGCTGTTGCCGGTGCTGTGCCAGGACCA

TGGCCTGACCCCGGACCAGGTCGTGGCCATCGCCAGCAATGGCGGCGGCAAGCAGGCGCTGGAGACGGTGCAGCGGCTGT

TGCCGGTGCTGTGCCAGGCCCATGGCCTGCCCCCGGCGCAGGTGGTGGCCATCGCCAGCCACGATGGCGGCAAGCAGGCG

CTGGAGACGGTGCAGCGGCTGTTGCCGGTGCTGTGCCAGGACCATGGCCTGACCCCGGCGCAGGTGGTGGCCATCGCCAG

CAGTGGCGGCAAGCAGGCGCTGGAGACGGTGCAGCGGCTGTTGCCGGTGCTGTGCCAGGCCCATGGCCTGACCCTGGACC

AGGTGGTGGCCATCGCCAGCCATGGCGGCAGCAAGCAGGCGCTGGAGACGGTGCAGCGGCTGTTGCCGGTGCTGTGCCAG

GACCATGGCCTGACCCCGGACCAGGTGGTGGCCATCGCCAGCCACGATGGCGGCAAGCAGGCGCTGGAGACGGTGCAGCG

GCTGTTGCCGGTGCTGTGCCAGGCCCATGGCCTGACCCCGGACCAGGTCGTGGCCATCGCCAGCAATGGCGGCGGCAAGC

AGGCGCTGGAGACGGTGCAACGGCTGTTGCCGGTGCTGTGCCAGGACCATGGCCTGACCCCGGACCAGGTGGTGG

> pCC2FOS-MscI-11

CGCCAGCAATAACGGCGGCAAGCAGGCGCTGGAGACGGTGCAGCGGCTGTTGCCGGTGCTGTGCCAGGCCCATGGCCTGA

CCCCAGACCAGGTCGTGGCCATCGCCAGCAATATTGGCGGCAAGCAGGCGCTGGAGACGGTGCAGCGGCTGTTGCCGGTG

CTGTGCCAGGACCATGGCCTGACCCCGGACCAGGTCGTGGCCATCGCCAGCAATGGCGGCGGCAAGCAGGCGCTGGAGAC

GGTGCAGCGGCTGTTGCCGGTGCTGTGCCAGGCCCATGGCCTGCCCCCGGCGCAGGTGGTGGCCATCGCCAGCCACGATG

GCGGCAAGCAGGCGCTGGAGACGGTGCAGCGGCTGTTGCCGGTGCTGTGCCAGGACCATGGCCTGACCCCGGCGCAGGTG

GTGGCCATCGCCAGCAGTGGCGGCAAGCAGGCGCTGGAGACGGTGCAGCGGCTGTTGCCGGTGCTGTGCCAGGCCCATGG

CCTGACCCTGGACCAGGTGGTGGCCATCGCCAGCCATGGCGGCAGCAAGCAGGCGCTGGAGACGGTGCAGCGGCTGTTGC

CGGTGCTGTGCCAGGACCATGGCCTGACCCCGGACCAGGTGGTGGCCATCGCCAGCCACGATGGCGGCAAGCAGGCGCTG

GAGACGGTGCAGCGGCTGTTGCCGGTGCTGTGCCAGGCCCATGGCCTGACCCCGGACCAGGTCGTGGCCATCGCCAGCAA

TGGCGGCGGCAAGCAGGCGCTGGAGACGGTGCAACGGCTGTTGCCGGTGCTGTGCCAGGACCATGGCCTGACCCCGGACC

AGGTGGTGGCCATCGCCAGCAATGGCGGCAAGCAGGCGCTGGAGACGGTGCAGCGGCTGTTGCCGGTGCT

> pCC2FOS-MscI-12

AGCACCGGCAACAGCCGCTGCACCGTCTCCAGCGCCTGCTTGCCGCCAATATTACTGGCGATGGCCACCACCTGGTCCGG

GGTCAGGCCATGGTCCTGGCACAGCACCGGCAACAGCCGCTGCACCGTCTCCAGCGCCTGCTTGCCGCCAATATTGCTGG

CGATGGCCACCACCTGCGCCAGGGTCAGGCCATGGGCCTGGCACAGCACCGGCAACAGCCGCTGCACCGTCTCCAGCGCC

TGCTTGCCGCCATTGCTGGCGATGGCCACCACCTGGTCCGGGGTCAGGCCATGGGCCTGGCACAGCACCGGCAACAGCCG

CTGCACCGTCTCCAGCGCCTGCTTGCCGCCATCGTGGCTGGCGATGGCCACCACCTGGTCCGGGGTCAGGCCATGGTCCT

GGCACAGCACCGGCAACAGCCGCTGCACCGTCTCCAGCGCCTGCTTGCCGCCATCGTGGCTGGCGATGGCCACGACCTGG

TCCGGGGTCAGGCCATGGGCCTGGCACAGCACCGGCAACAGCCGCTGCACCGTCGCCAGCGCCTGCTTGCCGCCGCCATT

GCTGGCGATGGCCACGACCTGGTCCGGGGTCAGGCCATGGTCCTGGCACAGCACCGGCAACAGCCGCTGCACCGTCTCCA

GCGCCTGCTTGCCGCCATTGCTGGCGATGGCCACCACCTGGTCCGGGGTCAGGCCATGGTCCTGGCACAGCACCGGCAAC

AGCCGTTGCACCGTCTCCAGCGCCTGCTTGCCGCCGCCATTGCTGGCGATGGCCACGACCTGGTCCGGGGTCAGGCCATG

GGCCTGGCACAGCACCGGCAACAGCCGCTGCACCGTCTCCAGCGCCTGCTTGCCGCCATCGTGGC

> pCC2FOS-MscI-13

AGCACCGGCAACAGCCGCTGCACCGTCTCCAGCGCCTGCTTGCTGCCGTCATTGCTGGCAATGGCCACCACCTGGTCCAG

GGTCAGGCCATGGTCCTGGCACAGCACCGGCAACAGCCGCTGCACCGTCTCCAGCGCCTGCTTGCCGCCAATATGGCTGG

CGATGGCCACCACCTGCGCCGGGGTCAGGCCATGGGCCTGGCACAGCACCGGCAACAGCCGCTGCACCGTCTCCAGCGCC

TGCTTGCCGCCATCGTGGCTGGCGATGGCCACGACCTGATCCAGGGTCAGGCCATGGTCCTGGCACAGCACCGGCAACAG

CCGCTGCACCGTCTCCAGCGCCTGCTTGCCGCCGTTATTGCTGGCGATGGCCACCACCTGGTCCGGGGTCAGGCCATGGT

CCTGGCACAGCACCGGCAACAGCCGCTGCACCGTCTCCAGCGCCTGCTTGCCGCCAATATTACTGGCGATGGCCACCACC

TGGTCCGGGGTCAGGCCATGGTCCTGGCACAGCACCGGCAACAGCCGCTGCACCGTCTCCAGCGCCTGCTTGCCGCCAAT

ATTGCTGGCGATGGCCACCACCTGCGCCAGGGTCAGGCCATGGGCCTGGCACAGCACCGGCAACAGCCGCTGCACCGTCT

CCAGCGCCTGCTTGCCGCCATTGCTGGCGATGGCCACCACCTGGTCCGGGGTCAGGCCATGGGCCTGGCACAGCACCGGC

AACAGCCGCTGCACCGTCTCCAGCGCCTGCTTGCCGCCATCGTGGCTGGCGATGGCCACCACCTGGTCCGGGGTCAGGCC

ATGGTCCTGGCACAGCACCGGCAACAGCCGCTGCACCGTCTCCAGCGCCTGCTTGCCGCCATCGTGGCT

> pCC2FOS-MscI-14

CATCGCCAGCAATATTGGCGGCAAGCAGGCGCTGGAGACGGTGCAGCGGCTGTTGCCGGTGCTGTGCCAGGACCATGGCC

TGACCCCGGACCAGGTGGTGGCCATCGCCAGTAATATTGGCGGCAAGCAGGCGCTGGAGACGGTGCAGCGGCTGTTGCCG

GTGCTGTGCCAGGACCATGGCCTGACCCCGGACCAGGTGGTGGCCATCGCCAGCAATAACGGCGGCAAGCAGGCGCTGGA

GACGGTGCAGCGGCTGTTGCCGGTGCTGTGCCAGGACCATGGCCTGACCCTGGATCAGGTCGTGGCCATCGCCAGCCACG

ATGGCGGCAAGCAGGCGCTGGAGACGGTGCAGCGGCTGTTGCCGGTGCTGTGCCAGGCCCATGGCCTGACCCCGGCGCAG

GTGGTGGCCATCGCCAGCCATATTGGCGGCAAGCAGGCGCTGGAGACGGTGCAGCGGCTGTTGCCGGTGCTGTGCCAGGA

CCATGGCCTGACCCTGGACCAGGTGGTGGCCATTGCCAGCAATGACGGCAGCAAGCAGGCGCTGGAGACGGTGCAGCGGC

TGTTGCCGGTGCTGTGCCAGGACCATGGCCTGACCCCGGACCAGGTGGTGGCCATCGCCAGCCACGATGGCGGCAAGCAG

GCGCTGGAGACGGTGCAGCGGCTGTTGCCGGTGCTGTGCCAGGACCATGGCCTGACCCCGGACCAGGTGGTGGCCATCGC

CAGCAATGGCGGCGGCAAGCAGGCGCTGGAGACGGTGCAACGGCTGTTGCCGGTGCTGTGCCAGGACCATGGCCTGACCC

CGGCCCAGGTCGTGGCCATCGCCAACAATAACGGCGGCAAGCAGGCGCTGGAGACGGTGCAGCGGCTGTTGCCGGTGCT

> pCC2FOS-MscI-15

AGCACCGGCAACAGCCGCTGCACCGTCTCCAGCGCCTGCTTGCCGCCATCGTGGCTGGCGATGGCCACCACCTGGTCCGG

GGTCAGGCCATGGTCCTGGCACAGCACCGGCAACAGCCGCTGCACCGTCTCCAGCGCCTGCTTGCTGCCGCCATGGCTGG

CGATGGCCACCACCTGGTCCAGGGTCAGGCCATGGGCCTGGCACAGCACCGGCAACAGCCGCTGCACCGTCTCCAGCGCC

TGCTTGCCGCCACTGCTGGCGATGGCCACCACCTGCGCCGGGGTCAGGCCATGGTCCTGGCACAGCACCGGCAACAGCCG

CTGCACCGTCTCCAGCGCCTGCTTGCCGCCATCGTGGCTGGCGATGGCCACCACCTGCGCCGGGGGCAGGCCATGGGCCT

GGCACAGCACCGGCAACAGCCGCTGCACCGTCTCCAGCGCCTGCTTGCCGCCGCCATTGCTGGCGATGGCCACGACCTGG

TCCGGGGTCAGGCCATGGTCCTGGCACAGCACCGGCAACAGCCGCTGCACCGTCTCCAGCGCCTGCTTGCCGCCAATATT

GCTGGCGATGGCCACGACCTGGTCTGGGGTCAGGCCATGGGCCTGGCACAGCACCGGCAACAGCCGCTGCACCGTCTCCA

GCGCCTGCTTGCCGCCGTTATTGCTGGCGATGGCCACGACCTGGTCCGGGGTCAGGCCATGGGCCTGGCACAGCACCGGC

AACAGCCGCTGCACCGTCTCCAGCGCCTGCTTGCCGCCGTTATTGCTGGCGATGGCCACCACCTGCGCTGGGGTCAGACC

ATGGGCCTGGCACAGCACCGGCAACAGCCGCTGCACCGTCTCCAGCGCCTGCTTGCCGCCATCGTGGCTGGCG

> pCC2FOS-MscI-16

GGCACAGCACCGGCAACAGCCGCTGCACCGTCTCCAGCGCCTGCTTGCTGCCGTCATTGCTGGCAATGGCCACCACCTGG

TCCAGGGTCAGGCCATGGTCCTGGCACAGCACCGGCAACAGCCGCTGCACCGTCTCCAGCGCCTGCTTGCCGCCAATATG

GCTGGCGATGGCCACCACCTGCGCCGGGGTCAGGCCATGGGCCTGGCACAGCACCGGCAACAGCCGCTGCACCGTCTCCA

GCGCCTGCTTGCCGCCATCGTGGCTGGCGATGGCCACGACCTGATCCAGGGTCAGGCCATGGTCCTGGCACAGCACCGGC

AACAGCCGCTGCACCGTCTCCAGCGCCTGCTTGCCGCCGTTATTGCTGGCGATGGCCACCACCTGGTCCGGGGTCAGGCC

ATGGTCCTGGCACAGCACCGGCAACAGCCGCTGCACCGTCTCCAGCGCCTGCTTGCCGCCAATATTACTGGCGATGGCCA

CCACCTGGTCCGGGGTCAGGCCATGGTCCTGGCACAGCACCGGCAACAGCCGCTGCACCGTCTCCAGCGCCTGCTTGCCG

CCAATATTGCTGGCGATGGCCACCACCTGCGCCAGGGTCAGGCCATGGGCCTGGCACAGCACCGGCAACAGCCGCTGCAC

CGTCTCCAGCGCCTGCTTGCCGCCATTGCTGGCGATGGCCACCACCTGGTCCGGGGTCAGGCCATGGGCCTGGCACAGCA

CCGGCAACAGCCGCTGCACCGTCTCCAGCGCCTGCTTGCCGCCATCGTGGCTGGCGATGGCCACCACCTGGTCCGGGGTC

AGGCCATGGTCCTGGCACAGCACCGGCAACAGCCGCTGCACCGTCTCCAGCGCCTGCTTGCCGCCATCGTGGCTGGCG

>22P1F_1

CGGGGGAGTTGAGAGGTCCGCCGTTACAGTTGGACACAGGCCAACTTCTCAAGATTGCAAAACGTGGCGGCGTGACCGCA

GTGGAGGCAGTGCATGCATCGCGCAATGCACTGACGGGTGCCCCCCTGAACCTGACCCCGGACCAAGTGGTGGCCATCGC

CAGCCACGATGGCGGCAAGCAGGCACTGGAGACGGTGCAGCGGCTGTTGCCGGTGCTGTGCCAGGCCCATGGCTTGACCC

CGGCGCAGGTGGTGGCCATCGCCAGCCACGATGGCGGCAAGCAGGCGCTGGAGACGGTGCAGCGGCTGTTGCCGGTGCTG

TGCCAGGCCCATGGTCTGACCCCAGCGCAGGTGGTGGCCATCGCCAGCAATAACGGCGGCAAGCAGGCGCTGGAGACGGT

GCAGCGGCTGTTGCCGGTGCTGTGCCAGGCCCATGGCCTGACCCCGGACCAGGTCGTGGCCATCGCCAGCAATAACGGCG

GCAAGCAGGCGCTGGAGACGGTGCAGCGGCTGTTGCCGGTGCTGTGCCAGGCCCATGGCCTGACCCCAGACCAGGTCGTG

GCCATCGCCAGCAATATTGGCGGCAAGCAGGCGCTGGAGACGGTGCAGCGGCTGTTGCCGGTGCTGTGCCAGGACCATGG

CCTGACCCCGGACCAGGTCGTGGCCATCGCCAGCAATGGCGGCGGCAAGCAGGCGCTGGAGACGGTGCAGCGGCTGTTGC

CGGTGCTGTGCCAGGCCCATGGCCTGCCCCCGGCGCAGGTGGTGGCCATCGCCAGCCACGATGGCGGCAAGCAGGCGCTG

GAGACGGTGCAGCGGCTGTTGCCGGTGCTGTGCCAGGACCATGGCCTGACCCCGGCGCAGGTGGTGGCCATCGCCAGCAG

TGGCGGCAAGCAGGCGCTGGAGACGGTGCAGCGGCTGTTGCCGGTGCTGTGCCAGGCCCATGGCCTGACCCTGGACCAGG

TGGTGGCCATCGCCAGCCA

>22P1F_2

AGGCGGGGGAGTTGAGAGGTCCGCCGTTACAGTTGGACACAGGCCAACTTCTCAAGATTGCAAAACGTGGCGGCGTGACC

GCAGTGGAGGCAGTGCATGCATCGCGCAATGCACTGACGGGTGCCCCCCTGAACCTGACCCCGGACCAAGTGGTGGCCAT

CGCCAGCCACGATGGCGGCAAGCAGGCACTGGAGACGGTGCAGCGGCTGTTGCCGGTGCTGTGCCAGGCCCATGGCTTGA

CCCCGGCGCAGGTGGTGGCCATCGCCAGCCACGATGGCGGCAAGCAGGCGCTGGAGACGGTGCAGCGGCTGTTGCCGGTG

CTGTGCCAGGCCCATGGTCTGACCCCAGCGCAGGTGGTGGCCATCGCCAGCAATAACGGCGGCAAGCAGGCGCTGGAGAC

GGTGCAGCGGCTGTTGCCGGTGCTGTGCCAGGCCCATGGCCTGACCCCGGACCAGGTCGTGGCCATCGCCAGCAATAACG

GCGGCAAGCAGGCGCTGGAGACGGTGCAGCGGCTGTTGCCGGTGCTGTGCCAGGCCCATGGCCTGACCCCAGACCAGGTC

GTGGCCATCGCCAGCAATATTGGCGGCAAGCAGGCGCTGGAGACGGTGCAGCGGCTGTTGCCGGTGCTGTGCCAGGACCA

TGGCCTGACCCCGGACCAGGTCGTGGCCATCGCCAGCAATGGCGGCGGCAAGCAGGCGCTGGAGACGGTGCAGCGGCTGT

TGCCGGTGCTGTGCCAGG

>22P1R_1

ACTGGGCAACAATGCTCTCCAGCGCCTGCTTGCCGCCATTGCTGGCGATGGCCACCACCTGGTCCGGGGTCAGGCCATGG

TCCTGGCACAGCACCGGCAACAGCCGCTGCACCGTCTCCAGCGCCTGCTTGCCGCCGCCATGGCTGGCGATGGCCACGAC

CTGGTCCGGGGTCAGGCCATGGTCCTGGCACAGCACCGGCAACAGCCGCTGCACCGTCTCCAGCGCCTGCTTGCCGCCGT

TATTGTTGGCGATGGCCACGACCTGGGCCGGGGTCAGGCCATGGTCCTGGCACAGCACCGGCAACAGCCGTTGCACCGTC

TCCAGCGCCTGCTTGCCGCCGCCATTGCTGGCGATGGCCACCACCTGGTCCGGGGTCAGGCCATGGTCCTGGCACAGCAC

CGGCAACAGCCGCTGCACCGTCTCCAGCGCCTGCTTGCCGCCATCGTGGCTGGCGATGGCCACCACCTGGTCCGGGGTCA

GGCCATGGTCCTGGCACAGCACCGGCAACAGCCGCTGCACCGTCTCCAGCGCCTGCTTGCTGCCGTCATTGCTGGCAATG

GCCACCACCTGGTCCAGGGTCAGGCCATGGTCCTGGCACAGCACCGGCAACAGCCGCTGCACCGTCTCCAGCGCCTGCTT

GCCGCCAATATGGCTGGCGATGGCCACCACCTGCGCCGGGGTCAGGCCATGGGCCTGGCACAGCACCGGCAACAGCCGCT

GCACCGTCTCCAGCGCCTGCTTGCCGCCATCGTGGCTGGCGATGGCCACGACCTGATCCAGGGTCAGGCCATGGTCCTGG

CACAGCACCGGCAACAGCCGCTGCACCGTCTCCAGCGCCTGCTTGCCGCCGTTATTGCTGGCGATGGCCACCACCTGGTC

CGGGGTCAGGCCATGGTCCTGGCACAGCACCGGCAACAGCCGCTGCACCGTCTCCAGCGCCTGCTTGCCGCCAATATTAC

TGGCGATGGCCACCAC

>22P1R_2

CGGCAAGCAGGCGCTGGAGACGGTGCAGCGGCTGTTGCCGGTGCTGTGCCAGGACCATGGCCTGACCCTGGATCAGGTCG

TGGCCATCGCCAGCCACGATGGCGGCAAGCAGGCGCTGGAGACGGTGCAGCGGCTGTTGCCGGTGCTGTGCCAGGCCCAT

GGCCTGACCCCGGCGCAGGTGGTGGCCATCGCCAGCCATATTGGCGGCAAGCAGGCGCTGGAGACGGTGCAGCGGCTGTT

GCCGGTGCTGTGCCAGGACCATGGCCTGACCCTGGACCAGGTGGTGGCCATTGCCAGCAATGACGGCAGCAAGCAGGCGC

TGGAGACGGTGCAGCGGCTGTTGCCGGTGCTGTGCCAGGACCATGGCCTGACCCCGGACCAGGTGGTGGCCATCGCCAGC

CACGATGGCGGCAAGCAGGCGCTGGAGACGGTGCAGCGGCTGTTGCCGGTGCTGTGCCAGGACCATGGCCTGACCCCGGA

CCAGGTGGTGGCCATCGCCAGCAATGGCGGCGGCAAGCAGGCGCTGGAGACGGTGCAACGGCTGTTGCCGGTGCTGTGCC

AGGACCATGGCCTGACCCCGGCCCAGGTCGTGGCCATCGCCAACAATAACGGCGGCAAGCAGGCGCTGGAGACGGTGCAG

CGGCTGTTGCCGGTGCTGTGCCAGGACCATGGCCTGACCCCGGACCAGGTCGTGGCCATCGCCAGCCATGGCGGCGGCAA

GCAGGCGCTGGAGACGGTGCAGCGGCTGTTGCCGGTGCTGTGCCAGGACCATGGCCTGACCCCGGACCAGGTGGTGGCCA

TCGCCAGCAATGGCGGCAAGCAGGCGCTGGAGAGCATTGTTGCCC

>22P2F

CGCGCACATCGTTGCGCTCAGCCAACACCCGGCAGCGTTAGGGACCGTTGCTGTCACGTATCAGGACATAATCAGGGCGT

TGCCAGAGGCGACACACGAAGACATCGTTGGCGTCGGCAAACAGTGGTCCGGCGCACGCGCCCTGGAGGCCTTGCTCACG

AAGGCGGGGGAGTTGAGAGGTCCGCCGTTACAGTTGGACACAGGCCAACTTCTCAAGATTGCAAAACGTGGCGGCGTGAC

CGCAGTGGAGGCAGTGCATGCATCGCGCAATGCACTGACGGGTGCCCCCCTGAACCTGACCCCGGACCAAGTGGTGGCCA

TCGCCAGCCACGATGGCGGCAAGCAGGCACTGGAGACGGTGCAGCGGCTGTTGCCGGTGCTGTGCCAGGCCCATGGCTTG

ACCCCGGCGCAGGTGGTGGCCATCGCCAGCCACGATGGCGGCAAGCAGGCGCTGGAGACGGTGCAGCGGCTGTTGCCGGT

GCTGTGCCAGGCCCATGGTCTGACCCCAGCGCAGGTGGTGGCCATCGCCAGCAATAACGGCGGCAAGCAGGCGCTGGAGA

CGGTGCAGCGGCTGTTGCCGGTGCTGTGCCAGGCCCATGGCCTGACCCCGGACCAGGTCGTGGCCATCGCCAGCAATAAC

GGCGGCAAGCAGGCGCTGGAGACGGTGCAGCGGCTGTTGCCGGTGCTGTGCCAGGCCCATGGCCTGACCCCAGACCAGGT

CGTGGCCATCGCCAGCAATATTGGCGGCAAGCAGGCGCTGGAGACGGTGCAGCGGCTGTTGCCGGTGCTGTGCCAGGACC

ATGGCCTGACCCCGGACCAGGTCGTGGCCATCGCCAGCAATGGCGGCGGCAAGCAGGCGCTGGAGACGGTGCAGCGGCTG

TTGCCGGTGCTGTGCCAGGCCCATGGCCTGCCCCCGGCGCAGGTGGTGGCCATCGCCAGCCACGATGGCGGCAAGCAGGC

GCTGGAGACGGTGCAGCG

>22P2R

CTGATTATGTCCTGATACGTGACAGCAACGGTCCCTAACGCTGCCGGGTGTTGGCTGAGCGCAACGATGTGCGCGTGTGT

AAACCCATGGCCCACCAGTGCCTCGTGGTGCTGCGCCACTGTCGAACGCACCTTCGGTTTGATCTTCTCTTGCTGCTGCT

GACTGTAGCCGAGCGTGCGTAGATCCACCTGCGCGGCCGGCGAAGCGTCGGAGGGTTGCGCCGCACGCCGTCGCGGGGCC

GGCTTGGCGCGCGGCGGCCGCGCGGCAGTGACAGCGACAGGCACGGTGGGTGGCGGGTCATCGGCTGCACGCAGACCCGA

TTGCGCCTCATCCCATTCTGCTGGGGCAGCCGCTGTATGCGGCGTGCCGACGGCAGGCATCGAATCAAGAAGCGATGTAT

CAAGAAGCGACGGATCGAACTGACGGAGCAGATCGCTGAAGCTGCCCGCCGAGAACGCAGGCGAGGGCGCAGGGGGAGAT

GGCAGCCGGGTCCGGGACATCGTCCGCCGAGCGGGCAAGCCATCCAGGGGGCCGCCAGCAGGCGGAGCCCCCCCCCGATC

TGCAGTCGGCTGAACCCTATCCGGTTGGGGTCCGGGCAGAAGCTCGCGGGCAGGACTTGGCGTGCGCGAACGAATGGGAT

CC

>22P3F

GGATTGCCGCACGCGCCGGAATTGATCAGAAGAATCAATCGCCGCATTCCCGAACGCACGTCCCATCGCGTTCCCGACCT

CGCGCACGTGGTGCGCGTGCTTGGTTTTTTCCAGAGCCACTCCCACCCAGCGCAAGCATTCGATGACGCCATGACGCAGT

TCGGGATGAGCAGGAACGGGTTGGTACAGCTCTTTCGCAGAGTGGGCGTCACCGAACTCGAAGCCCGCGGTGGAACGCTC

CCCCCAGCCTCGCAGCGTTGGGACCGTATCCTCCAGGCATCAGGGATGAAAAGGGCCAAACCGTCCCCTACTTCAGCTCA

AACACCGGATCAGGCGTCTTTGCATGCATTCGCCGATTCGCTGGAGCGTGACCTTGATGCGCCTAGCCCAATGCACGAGG

GAGATCAGACAGGGGCAAGCAGCCGTAAACGGTCCCGATCGGATCGTGCTGTCACCAGCCCCTCCGCACAGCAATCTTTC

GAGGTGCGCGTTCCCGAACAGCACGATGCGCTGCATTTGCCCCTCAGCTGGAGGGTAAAACGCCCGCGTACCAGGATCGG

GGGCGGCCTCCCGGATCC

>22P3R

CTTCGAGTTCGGTGACGCCCACTCTGCGAAAGAGCTGTACCAACCCGTTCCTGCTCATCCCGAACTGCGTCATGGCGTCA

TCGAATGCTTGCGCTGGGTGGGAGTGGCTCTGGAAAAAACCAAGCACGCGCACCACGTGCGCGAGGTCGGGAACGCGATG

GGACGTGCGTTCGGGAATGCGGCGATTGATTCTTCTGATCAATTCCGGCGCGTGCGGCAATCCCTTTTTCACTGCATCCA

GGGCAGGACGTCCGCCGAGGCAGGCCAAGGCGACGAGGTGGTCGTTGGTCAACGCGGCCAACTCCGGATCAGGGCGAGAT

AACTGGGCAACAATGCTCTCCAGCGCCTGCTTGCCGCCATTGCTGGCGATGGCCACCACCTGGTCCGGGGTCAGGCCATG

GTCCTGGCACAGCACCGGCAACAGCCGCTGCACCGTCTCCAGCGCCTGCTTGCCGCCGCCATGGCTGGCGATGGCCACGA

CCTGGTCCGGGGTCAGGCCATGGTCCTGGCACAGCACCGGCAACAGCCGCTGCACCGTCTCCAGCGCCTGCTTGCCGCCG

TTATTGTTGGCGATGGCCACGACCTGGGCCGGGGTCAGGCCATGGTCCTGGCACAGCACCGGCAACAGCCGTTGCACCGT

CTCCAGCGCCTGCTTGCCGCCGCCATTGCTGGCGATGGCCACCACCTGGTCCGGGGTCAGGCCATGGTCCTGGCACAGCA

CCGGCAACAGCCGCTGCACCGTCTCCAGCGCCTGCTTGCCGCCATCGTGGCTGGCGATGGCCACCACCTGGTCCGGGGTC

AGGCCATGGTCCTGGCACAGCACCGGCAACAGCCGCTGCACCGTCTCCAGCGCCTGCTTGCTGCCGTCATTGCTGGCAAT

GGCCACCACCTGGTCCAGGGTCAGGCCATGGTCCTGGCACAGCACCGGCAACAGCCGCTGCACCGTCTCCAGCGCCTGCT

TGCCGCCAATATGGCTGGCGAT

>M13F

GGATCCCATTCGTTCGCGCACGCCAAGTCCTGCCCGCGAGCTTCTGCCCGGACCCCAACCGGATAGGGTTCAGCCGACTG

CAGATCGGGGGGGGGCTCCGCCTGCTGGCGGCCCCCTGGATGGCTTGCCCGCTCGGCGGACGATGTCCCGGACCCGGCTG

CCATCTCCCCCTGCGCCCTCGCCTGCGTTCTCGGCGGGCAGCTTCAGCGATCTGCTCCGTCAGTTCGATCCGTCGCTTCT

TGATACATCGCTTCTTGATTCGATGCCTGCCGTCGGCACGCCGCATACAGCGGCTGCCCCAGCAGAATGGGATGAGGCGC

AATCGGGTCTGCGTGCAGCCGATGACCCGCCACCCACCGTGCCTGTCGCTGTCACTGCCGCGCGGCCGCCGCGCGCCAAG

CCGGCCCCGCGACGGCGTGCGGCGCAACCCTCCGACGCTTCGCCGGCCGCGCAGGTGGATCTACGCACGCTCGGCTACAG

TCAGCAGCAGCAAGAGAAGATCAAACCGAAGGTGCGTTCGACAGTGGCGCAGCACCACGAGGCACTGGTGGGCCATGGGT

TTACACACGCGCACATCGTTGCGCTCAGCCAACACCCGGCAGCGTTAGGGACCGTTGCTGTCACGTATCAGGACATAATC

AGGGCGTTGCCAGAGGCGACACACGAAGACATCGTTGGCGTCGGCAAACAGTGGTCCGGCGCACGCGCCCTGGAGGCCTT

GCTCACGAAGGCGGGGGAGTTGAGAGGTCCGCCGTTACAGTTGGACACAGGCCAACTTCTCAAGATTGCAAAACGTGGCG

GCGTGACCGCAGTGGAGGCAGTGCATGCATCGCGCAATGCACTGACGGGTGCCCCCCTGAACCTGACCCCGGACCAAGTG

GTGGCCATCGCCAGCCACGATGGCGGCAAGCAGGCACTGGAGACGGTGCAGCGGCTGTTGCCGGTGCTGTGCCAGGCCCA

TGGCT

>M13R

CCCGGCCCAGGTCGTGGCCATCGCCAACAATAACGGCGGCAAGCAGGCGCTGGAGACGGTGCAGCGGCTGTTGCCGGTGC

TGTGCCAGGACCATGGCCTGACCCCGGACCAGGTCGTGGCCATCGCCAGCCATGGCGGCGGCAAGCAGGCGCTGGAGACG

GTGCAGCGGCTGTTGCCGGTGCTGTGCCAGGACCATGGCCTGACCCCGGACCAGGTGGTGGCCATCGCCAGCAATGGCGG

CAAGCAGGCGCTGGAGAGCATTGTTGCCCAGTTATCTCGCCCTGATCCGGAGTTGGCCGCGTTGACCAACGACCACCTCG

TCGCCTTGGCCTGCCTCGGCGGACGTCCTGCCCTGGATGCAGTGAAAAAGGGATTGCCGCACGCGCCGGAATTGATCAGA

AGAATCAATCGCCGCATTCCCGAACGCACGTCCCATCGCGTTCCCGACCTCGCGCACGTGGTGCGCGTGCTTGGTTTTTT

CCAGAGCCACTCCCACCCAGCGCAAGCATTCGATGACGCCATGACGCAGTTCGGGATGAGCAGGAACGGGTTGGTACAGC

TCTTTCGCAGAGTGGGCGTCACCGAACTCGAAGCCCGCGGTGGAACGCTCCCCCCAGCCTCGCAGCGTTGGGACCGTATC

CTCCAGGCATCAGGGATGAAAAGGGCCAAACCGTCCCCTACTTCAGCTCAAACACCGGATCAGGCGTCTTTGCATGCATT

CGCCGATTCGCTGGAGCGTGACCTTGATGCGCCTAGCCCAATGCACGAGGGAGATCAGACAGGGGCAAGCAGCCGTAAAC

GGTCCCGATCGGATCGTGCTGTCACCAGCCCCTCCGCACAGCAATCTTTCGAGGTGCGCGTTCCCGAACAGCACGATGCG

CTGCATTTGCCCCTCAGCTGGAGGGTAAAACGCCCGCGTACCAGGATCGGGGGCGGCCTCCCGGATCC

**pTAL*_Bam_*_HI_-24**

> pCC2FOS-MscI-1

AGCAGGCGCTGGAGACGGTGCAGCGGCTGTTGCCGGTGCTGTGCCAGGCCCATGGCCTGACCCCGGACCAGGTGGTGGCC

ATCGCCAGCCACGATGGCGGCAAGCAGGCGCTGGAGACGGTGCAGCGGCTGTTGCCGGTGCTGTGCCAGGCCCATGGCCT

GACCCCGGCCCAGGTGGTGGCCATCGCCAGCCACGATGGCGGCAAGCAGGCGCTGGAGACGGTGCAGCGGCTGTTGCCGG

TGCTGTGCCAGGCCCATGGTCTGACCCTGGACCAGGTAGTGGCCATTGCCAGCCACGATGGCGGCAAGCAGGCGCTGGAG

ACGGTGCAGCGGCTGTTGCCGGTGCTGTGCCAGGCCCATGGTCTGACCCTGGACCAGGTAGTGGCCATTGCCAGCCACGA

TGGCGGCAAGCAGGCGCTGGAGACGGTGCAGCGGCTGTTGCCGGTGCTGTGCCAGGACCATGGTCTGACCCCGGCGCAGG

TGGTGGCCATCGCCAGCAATAACGGCGGCAAGCAGGCGCTGGAGACGGTGCAGCGGCTGTTGCCGGTGCTGTGCCAGGAC

CATGGCCTGACCCCGGACCAGGTGGTGGCCATCGCCAGCCACGATGGCGGCAAGCAGGCGCTGGAGACGATGCAGCGGCT

GTTGCCGGTGCTGTGCCAGGCCCATGGCCTGACCCCGGACCAGGTCGTGGCCATCGCCAGCAATGGCGGCGGCAAGCAGG

CGCTGGAGACGGTGCAGCGGCTGTTGCCGGTGCTGTGCCAGGCCCATGGCCTGACCCCGGACCAGGTCGTGGCCATCGCC

AGCCACGATGGCGGCAAGCAGGCGCTGGAGACGGTGCAGCGGCTGTTGCCGGTGCTGTGCCAGGACCATGGCCTGACCCC

GGACCAGGTCGTGG

> pCC2FOS-MscI-2

GCGCTGGAGACGGTGCAGCGGCTGTTGCCGGTGCTGTGCCAGGACCATGGTCTGACCCCGGCGCAGGTGGTGGCCATCGC

CAGCAATAACGGCGGCAAGCAGGCGCTGGAGACGGTGCAGCGGCTGTTGCCGGTGCTGTGCCAGGACCATGGCCTGACCC

CGGACCAGGTGGTGGCCATCGCCAGCCACGATGGCGGCAAGCAGGCGCTGGAGACGATGCAGCGGCTGTTGCCGGTGCTG

TGCCAGGCCCATGGCCTGACCCCGGACCAGGTCGTGGCCATCGCCAGCAATGGCGGCGGCAAGCAGGCGCTGGAGACGGT

GCAGCGGCTGTTGCCGGTGCTGTGCCAGGCCCATGGCCTGACCCCGGACCAGGTCGTGGCCATCGCCAGCCACGATGGCG

GCAAGCAGGCGCTGGAGACGGTGCAGCGGCTGTTGCCGGTGCTGTGCCAGGACCATGGCCTGACCCCGGACCAGGTCGTG

GCCATCGCCAGCAATATTGGCGGCAAGCAGGCGCTGGAGACGGTGCAGCGGCTGTTGCCGGTGCTGTGCCAGGACCATGG

CCTGACCCCGGACCAGGTCGTGGCCATCGCCAGCCACGATGGCGGCAAGCAGGCGCTGGAGACGGTGCAGCGGCTGTTGC

CGGTGCTGTGCCAGGACCATGGCCTGACCCCGGACCAGGTCGTGGCCATCGCCAACAATAACGGCGGCAAGCAGGCGCTG

GAGACGGTGCAGCGGCTGTTGCCGGTGCTGTGCCAGGACCATGGCCTGACCCCGGACCAGGTCGTGG

> pCC2FOS-MscI-3

GCGCTGGAGACGGTGCAGCGGCTGTTGCCGGTGCTGTGCCAGGCCCATGGCCTGACCCCGGACCAGGTGGTGGCCATCGC

CAGCCACGATGGCGGCAAGCAGGCGCTGGAGACGGTGCAGCGGCTGTTGCCGGTGCTGTGCCAGGCCCATGGCCTGACCC

CGGCCCAGGTGGTGGCCATCGCCAGCCACGATGGCGGCAAGCAGGCGCTGGAGACGGTGCAGCGGCTGTTGCCGGTGCTG

TGCCAGGCCCATGGTCTGACCCTGGACCAGGTAGTGGCCATTGCCAGCCACGATGGCGGCAAGCAGGCGCTGGAGACGGT

GCAGCGGCTGTTGCCGGTGCTGTGCCAGGCCCATGGTCTGACCCTGGACCAGGTAGTGGCCATTGCCAGCCACGATGGCG

GCAAGCAGGCGCTGGAGACGGTGCAGCGGCTGTTGCCGGTGCTGTGCCAGGACCATGGTCTGACCCCGGCGCAGGTGGTG

GCCATCGCCAGCAATAACGGCGGCAAGCAGGCGCTGGAGACGGTGCAGCGGCTGTTGCCGGTGCTGTGCCAGGACCATGG

CCTGACCCCGGACCAGGTGGTGGCCATCGCCAGCCACGATGGCGGCAAGCAGGCGCTGGAGACGATGCAGCGGCTGTTGC

CGGTGCTGTGCCAGGCCCATGGCCTGACCCCGGACCAGGTCGTGGCCATCGCCAGCAATGGCGGCGGCAAGCAGGCGCTG

GAGACGGTGCAGCGGCTGTTGCCGGTGCTGTGCCAGGCCCATGGCCTGACCCCGGACCAGGTCGTGGCCATCGCCAGCCA

CGATGGCGGCAAGCAGGCGCTGGAGACGGTGCAGCGGCTGTTGCCGGTGCTGTGCCAGGACCATGGCCTGACCCCGGACC

AGGTCGTGG

> pCC2FOS-MscI-4

CCATTGCCAGCCACGATGGCGGCAAGCAGGCGCTGGAGACGGTGCAGCGGCTGTTGCCGGTGCTGTGCCAGGCCCATGGT

CTGACCCTGGACCAGGTAGTGGCCATTGCCAGCCACGATGGCGGCAAGCAGGCGCTGGAGACGGTGCAGCGGCTGTTGCC

GGTGCTGTGCCAGGACCATGGTCTGACCCCGGCGCAGGTGGTGGCCATCGCCAGCAATAACGGCGGCAAGCAGGCGCTGG

AGACGGTGCAGCGGCTGTTGCCGGTGCTGTGCCAGGACCATGGCCTGACCCCGGACCAGGTGGTGGCCATCGCCAGCCAC

GATGGCGGCAAGCAGGCGCTGGAGACGATGCAGCGGCTGTTGCCGGTGCTGTGCCAGGCCCATGGCCTGACCCCGGACCA

GGTCGTGGCCATCGCCAGCAATGGCGGCGGCAAGCAGGCGCTGGAGACGGTGCAGCGGCTGTTGCCGGTGCTGTGCCAGG

CCCATGGCCTGACCCCGGACCAGGTCGTGGCCATCGCCAGCCACGATGGCGGCAAGCAGGCGCTGGAGACGGTGCAGCGG

CTGTTGCCGGTGCTGTGCCAGGACCATGGCCTGACCCCGGACCAGGTCGTGGCCATCGCCAGCAATATTGGCGGCAAGCA

GGCGCTGGAGACGGTGCAGCGGCTGTTGCCGGTGCTGTGCCAGGACCATGGCCTGACCCCGGACCAGGTCGTGGCCATCG

CCAGCCACGATGGCGGCAAGCAGGCGCTGGAGACGGTGCAGCGGCTGTTGCCGGTGCTGTGCCAGGAC

> pCC2FOS-MscI-5

GCGCTGGAGACGGTGCAGCGGCTGTTGCCGGTGCTGTGCCAGGACCATGGTCTGACCCCGGCGCAGGTGGTGGCCATCGC

CAGCAATAACGGCGGCAAGCAGGCGCTGGAGACGGTGCAGCGGCTGTTGCCGGTGCTGTGCCAGGACCATGGCCTGACCC

CGGACCAGGTGGTGGCCATCGCCAGCCACGATGGCGGCAAGCAGGCGCTGGAGACGATGCAGCGGCTGTTGCCGGTGCTG

TGCCAGGCCCATGGCCTGACCCCGGACCAGGTCGTGGCCATCGCCAGCAATGGCGGCGGCAAGCAGGCGCTGGAGACGGT

GCAGCGGCTGTTGCCGGTGCTGTGCCAGGCCCATGGCCTGACCCCGGACCAGGTCGTGGCCATCGCCAGCCACGATGGCG

GCAAGCAGGCGCTGGAGACGGTGCAGCGGCTGTTGCCGGTGCTGTGCCAGGACCATGGCCTGACCCCGGACCAGGTCGTG

GCCATCGCCAGCAATATTGGCGGCAAGCAGGCGCTGGAGACGGTGCAGCGGCTGTTGCCGGTGCTGTGCCAGGACCATGG

CCTGACCCCGGACCAGGTCGTGGCCATCGCCAGCCACGATGGCGGCAAGCAGGCGCTGGAGACGGTGCAGCGGCTGTTGC

CGGTGCTGTGCCAGGACCATGGCCTGACCCCGGACCAGGTCGTGGCCATCGCCAACAATAACGGCGGCAAGCAGGCGCTG

GAGACGGTGCAGCGGCTGTTGCCGGTGCTGTGCCAGGACCATGGCCTGACCCCGGACCAGGTCGTGG

>24P1F_1

AGGCGGGGGAGTTGAGAGGTCCGCCGTTACAGTTGGACACAGGCCAACTTCTCAAGATTGCAAGACGTGGCGGCGTGACC

GCAGTGGAGGCAGTGCATGCATGGCGCAATGCACTGACGGGTGCCCCCCTGAACCTGACCCCGGACCAAGTGGTGGCCAT

CGCCAGCAATAGTGGCGGCAAGCAGGCGCTGGAGACGGTGCAGCGGCTGTTGCCGGTGCTGTGCCAGGACCATGGCCTGA

CCCCGGACCAGGTCGTGGCCATCGCCAGCCACGATGGCGGCAAGCAGGCGCTGGAGACGGTGCAGCGGCTGTTGCCGGTG

CTGTGCCAGGACCATGGCCTGACCCCGGACCAGGTGGTGGCCATCGCCAGCAATGGCGGCGGCAAGCAGGCGCTGGAGAC

GGTGCAGCGGCTGTTGCCGGTGCTGTGCCAGGACCATGGCCTGACCCCGGACCAGGTGGTGGCCATCGCCAGCAATGGCG

GCGGCAAGCAGGCGCTGGAGACGGTACTGTGCCAGGCCCATGGCCTGACCCCGGCGCAGGTGGTGGCCATCGCCAGCAAT

GGCGGCGGCAAGCAGGCGCTGGAGACGGTGCAGCGGCTGTTGCCGGTGCTGTGCCAGGCCCATGGCCTGACCCTGGACCA

GGTCGTGGCCATTGCCAGCAATGGCGGCGGCAAGCAGGCGCTGGAGACGGTGCAGCGGCTGTTGCCGGTGCTGTGCCAGG

CCCATGGCCTGACCCCGGACCAGGTGGTGGCCATCGCCAGCCACGATGGCGGCAAGCAGGCGCTGGAGACGGTGCAGCGG

CTGTTGCCGGTGCTGTGCCAGGCCCATGGCCTGACCCCGGCCCAGGTGGTGGCCATCGCCAGCCACGATGGCGGCAAGCA

GGCGCTGGAGACGGTGCAGCGGCTGTTGCCGGTGCTGTGCCAGGCCCATGGTCTGACCCTGGACCAGGTAGTGGCCATTG

CCAGCCACGATGGCGGCAAGCAG

>24P1F_2

GGCGGGGGAGTTGAGAGGTCCGCCGTTACAGTTGGACACAGGCCAACTTCTCAAGATTGCAAGACGTGGCGGCGTGACCG

CAGTGGAGGCAGTGCATGCATGGCGCAATGCACTGACGGGTGCCCCCCTGAACCTGACCCCGGACCAAGTGGTGGCCATC

GCCAGCAATAGTGGCGGCAAGCAGGCGCTGGAGACGGTGCAGCGGCTGTTGCCGGTGCTGTGCCAGGACCATGGCCTGAC

CCCGGACCAGGTCGTGGCCATCGCCAGCCACGATGGCGGCAAGCAGGCGCTGGAGACGGTGCAGCGGCTGTTGCCGGTGC

TGTGCCAGGACCATGGCCTGACCCCGGACCAGGTGGTGGCCATCGCCAGCAATGGCGGCGGCAAGCAGGCGCTGGAGACG

GTGCAGCGGCTGTTGCCGGTGCTGTGCCAGGACCATGGCCTGACCCCGGACCAGGTGGTGGCCATCGCCAGCAATGGCGG

CGGCAAGCAGGCGCTGGAGACGGTACTGTGCCAGGCCCATGGCCTGACCCCGGCGCAGGTGGTGGCCATCGCCAGCAATG

GCGGCGGCAAGCAGGCGCTGGAGACGGTGCAGCGGCTGTTGCCGGTGCTGTGCCAGGCCCATGGCCTGACCCTGGACCAG

GTCGTGGCCATTGCCAGCAATGGCGGCGGCAAGCAGGCGCTGGAGACGGTGCAGCGGCTGTTGCCGGTGCTGTGCCAGGC

CCATGGCCTGACCCCGGACCAGGTGGTGGCCATCGCCAGCCACGATGGCGGCAAGCAGGCGCTGGA

>24P1R_1

CTGGGCAACAATGCTCTCCAGCGCCTGCTTGCCGCCATTGCTGGCGATGGCCACGACCTGGTCCGGGGTCAGGCCATGGT

CCTGGCACAGCACCGGCAACAGCCGCTGCACCGTCTCCAGCGCCTGCTTGCCGCCGTTATTGTTGGCGATGGCCACGACC

TGGTCCGGGGTCAGGCCATGGTCCTGGCACAGCACCGGCAACAGCCGCTGCACCGTCTCCAGCGCCTGCTTGCCGCCATC

GTGGCTGGCGATGGCCACGACCTGGTCCGGGGTCAGGCCATGGTCCTGGCACAGCACCGGCAACAGCCGCTGCACCGTCT

CCAGCGCCTGCTTGCCGCCAATATTGCTGGCGATGGCCACGACCTGGTCCGGGGTCAGGCCATGGTCCTGGCACAGCACC

GGCAACAGCCGCTGCACCGTCTCCAGCGCCTGCTTGCCGCCATCGTGGCTGGCGATGGCCACGACCTGGTCCGGGGTCAG

GCCATGGGCCTGGCACAGCACCGGCAACAGCCGCTGCACCGTCTCCAGCGCCTGCTTGCCGCCGCCATTGCTGGCGATGG

CCACGACCTGGTCCGGGGTCAGGCCATGGGCCTGGCACAGCACCGGCAACAGCCGCTGCATCGTCTCCAGCGCCTGCTTG

CCGCCATCGTGGCTGGCGATGGCCACCACCTGGTCCGGGGTCAGGCCATGGTCCTGGCACAGCACCGGCAACAGCCGCTG

CACCGTCTCCAGCGCCTGCTTGCCGCCGTTATTGCTGGCGATGGCCACCACCTGCGCCGGGGTCAGACCATGGTCCTGGC

ACAGCACCGGCAACAGCCGCTGCACCGTCTCCAGCGCCTGCTTGCCGCCATCGTGGCTGGCAATGGCCACTACCTGGTCC

AGGGTCAGACCATGGGCCTGGCACAGCACCGGCAACAGCCGCTGCACCGTCTCCAGCGCCTGCTTGCCGCCATCGTGGCT

GGCAATGGCCACTA

>24P1R_2

GACCATGGTCTGACCCCGGCGCAGGTGGTGGCCATCGCCAGCAATAACGGCGGCAAGCAGGCGCTGGAGACGGTGCAGCG

GCTGTTGCCGGTGCTGTGCCAGGACCATGGCCTGACCCCGGACCAGGTGGTGGCCATCGCCAGCCACGATGGCGGCAAGC

AGGCGCTGGAGACGATGCAGCGGCTGTTGCCGGTGCTGTGCCAGGCCCATGGCCTGACCCCGGACCAGGTCGTGGCCATC

GCCAGCAATGGCGGCGGCAAGCAGGCGCTGGAGACGGTGCAGCGGCTGTTGCCGGTGCTGTGCCAGGCCCATGGCCTGAC

CCCGGACCAGGTCGTGGCCATCGCCAGCCACGATGGCGGCAAGCAGGCGCTGGAGACGGTGCAGCGGCTGTTGCCGGTGC

TGTGCCAGGACCATGGCCTGACCCCGGACCAGGTCGTGGCCATCGCCAGCAATATTGGCGGCAAGCAGGCGCTGGAGACG

GTGCAGCGGCTGTTGCCGGTGCTGTGCCAGGACCATGGCCTGACCCCGGACCAGGTCGTGGCCATCGCCAGCCACGATGG

CGGCAAGCAGGCGCTGGAGACGGTGCAGCGGCTGTTGCCGGTGCTGTGCCAGGACCATGGCCTGACCCCGGACCAGGTCG

TGGCCATCGCCAACAATAACGGCGGCAAGCAGGCGCTGGAGACGGTGCAGCGGCTGTTGCCGGTGCTGTGCCAGGACCAT

GGCCTGACCCCGGACCAGGTCGTGGCCATCGCCAGCAATGGCGGCAAGCAGGCGCTGGAGAGCATTGTTGCCCAGTTATC

TCG

>24P2F

CGCGCACATCGTTGCGCTCAGCCAACACCCGGCAGCGTTAGGGACCGTTGCTGTCACGTATCAAGACATAATCACGGCGT

TGCCAGAGGCGACACACGAAGACATCGTTGGCGTCGGCAAACAGTTGTCCGGCGCACGCGCCCTGGAGGCCTTGCTCACG

AAGGCGGGGGAGTTGAGAGGTCCGCCGTTACAGTTGGACACAGGCCAACTTCTCAAGATTGCAAGACGTGGCGGCGTGAC

CGCAGTGGAGGCAGTGCATGCATGGCGCAATGCACTGACGGGTGCCCCCCTGAACCTGACCCCGGACCAAGTGGTGGCCA

TCGCCAGCAATAGTGGCGGCAAGCAGGCGCTGGAGACGGTGCAGCGGCTGTTGCCGGTGCTGTGCCAGGACCATGGCCTG

ACCCCGGACCAGGTCGTGGCCATCGCCAGCCACGATGGCGGCAAGCAGGCGCTGGAGACGGTGCAGCGGCTGTTGCCGGT

GCTGTGCCAGGACCATGGCCTGACCCCGGACCAGGTGGTGGCCATCGCCAGCAATGGCGGCGGCAAGCAGGCGCTGGAGA

CGGTGCAGCGGCTGTTGCCGGTGCTGTGCCAGGACCATGGCCTGACCCCGGACCAGGTGGTGGCCATCGCCAGCAATGGC

GGCGGCAAGCAGGCGCTGGAGACGGTACTGTGCCAGGCCCATGGCCTGACCCCGGCGCAGGTGGTGGCCATCGCCAGCAA

TGGCGGCGGCAAGCAGGCGCTGGAGACGGTGCAGCGGCTGTTGCCGGTGCTGTGCCAGGCCCATGGCCTGACCCTGGACC

AGGTCGTGGCCATTGCCAGCAATGGCGGCGGCAAGCAGGCGCTGGAGACGGTGCAGCGGCTGTTGCCGGTGCTGTGCCAG

GCCCATGGCCTGACCCCGGACCAGGTGGTGGCCATCGCCAGCCACGATGGCGGCAAGCAGGCGCTGGAGACGGTGC

>24P2R

TTATGTCTTGATACGTGACAGCAACGGTCCCTAACGCTGCCGGGTGTTGGCTGAGCGCAACGATGTGCGCGTGTGTAAAC

CCATGGCCCACCAGTGCCTCGTGGTGCTGCGCCACTGTCGCGAGCGTGAGTAGATCCACCTGCGCGGCCGGCGAAGTGTC

TGCTGGGGCAGCCTCTGTATGAGGCGTGCCGACGGCAGGCATCGAATCAAAAAGCGATGTATCAGGAAGCGACGGATCGA

ACGGACGGAGCCGATCGGTGGAGCTGCCCGCCGAGAACGCAGGCAAGGGGGCAGGGGGAGATGGCAGCCGGGTCCGGGAC

ACCGTCCGCCGAGCGGGCAAGCCATCCAGAGGGCTGCCAGCAGGCGCAGACACCCCACGATCTGCAGTCGGCTGAACCCT

ATCCGGTTGGGGTCCGGGCAGAGGCTCGCGGGCAGGACTTGGCGTGCGCGAACGAATGGGATCC

>24P3F

GCAGTGAAAAAGGGATTGCCGCACGCGCCGGAATTGATCAGAAGAGTCAATAGCCGTATTGGCGAACGCACGTCCCATCG

CGTTGCCGACCTCGCGCACGTGGTGCGCGTGCTTGGTTTTTTCCAGAGCCACTCCCACCCAGCGCAAGCATTCGATGACG

CCATGACGCAGTTCGGGATGAGCAGGCACGGGTTGGTACAGCTCTTTCGCAGAGTGGGCGTCACCGAATTCGAAGCCCGC

TGCGGAACTATCCCCCCAGCCTCGCAGCGTTGGGACCGTATCCTCCAGGCATCAGGGACGAAAAGGGCCAAACCGTCCCC

TACTTCAGCTCAGACGCCGGATCAGGCGTCTTTGCATGCATTCCCCGACTCGCTGGAGCGTGACCTTGATGCGCCCAGCC

CAATGCACGAGGGAGATCAGACGCGGGCAAGCAGACGTAAACGGTCCTGATCGGATCGTGCTGTCACCGACCCCTCCGCA

CAGCAATCTTTCGAGGTGCGCGTTCCCGAACAGCACGATGCGCTGCATTTGCCCCTCAGCTGGAGGGTAAAACGCCCGCG

TACCAGGATCGGGGGCGGCCTCCCGGATCC

>24P3R

TTCGGTGACGCCCACTCTGCGAAAGAGCTGTACCAACCCGTGCCTGCTCATCCCGAACTGCGTCATGGCGTCATCGAATG

CTTGCGCTGGGTGGGAGTGGCTCTGGAAAAAACCAAGCACGCGCACCACGTGCGCGAGGTCGGCAACGCGATGGGACGTG

CGTTCGCCAATACGGCTATTGACTCTTCTGATCAATTCCGGCGCGTGCGGCAATCCCTTTTTCACTGCATCCAGGGCAGG

ACGTCCGCCGAGGCAGGCCAAGGCGACGAGGTGGTCGTTGGTCAACGCGGCCAACGCCGGATCAGGGCGAGATAACTGGG

CAACAATGCTCTCCAGCGCCTGCTTGCCGCCATTGCTGGCGATGGCCACGACCTGGTCCGGGGTCAGGCCATGGTCCTGG

CACAGCACCGGCAACAGCCGCTGCACCGTCTCCAGCGCCTGCTTGCCGCCGTTATTGTTGGCGATGGCCACGACCTGGTC

CGGGGTCAGGCCATGGTCCTGGCACAGCACCGGCAACAGCCGCTGCACCGTCTCCAGCGCCTGCTTGCCGCCATCGTGGC

TGGCGATGGCCACGACCTGGTCCGGGGTCAGGCCATGGTCCTGGCACAGCACCGGCAACAGCCGCTGCACCGTCTCCAGC

GCCTGCTTGCCGCCAATATTGCTGGCGATGGCCACGACCTGGTCCGGGGTCAGGCCATGGTCCTGGCACAGCACCGGCAA

CAGCCGCTGCACCGTCTCCAGCGCCTGCTTGCCGCCATCGTGGCTGGCGATGGCCACGACCTGGTCCGGGGTCAGGCCAT

GGGCCTGGCACAGCACCGGCAACAGCCGCTGCACCGTCTCCAGCGCCTGCTTGCCGCCGCCATTGCTGGCGATGGCCACG

ACCTGGTCCGGGGTCAGGCCATGGGCCTGGCACAGCACCGGCAACAGCCGCTGCATCGTCTCCAGCGCCTGCTTGCCGCC

ATCGTGGCTGGCGA

>M13F

GGATCCCATTCGTTCGCGCACGCCAAGTCCTGCCCGCGAGCCTCTGCCCGGACCCCAACCGGATAGGGTTCAGCCGACTG

CAGATCGTGGGGTGTCTGCGCCTGCTGGCAGCCCTCTGGATGGCTTGCCCGCTCGGCGGACGGTGTCCCGGACCCGGCTG

CCATCTCCCCCTGCCCCCTTGCCTGCGTTCTCGGCGGGCAGCTCCACCGATCGGCTCCGTCCGTTCGATCCGTCGCTTCC

TGATACATCGCTTTTTGATTCGATGCCTGCCGTCGGCACGCCTCATACAGAGGCTGCCCCAGCAGACACTTCGCCGGCCG

CGCAGGTGGATCTACTCACGCTCGCGACAGTGGCGCAGCACCACGAGGCACTGGTGGGCCATGGGTTTACACACGCGCAC

ATCGTTGCGCTCAGCCAACACCCGGCAGCGTTAGGGACCGTTGCTGTCACGTATCAAGACATAATCACGGCGTTGCCAGA

GGCGACACACGAAGACATCGTTGGCGTCGGCAAACAGTTGTCCGGCGCACGCGCCCTGGAGGCCTTGCTCACGAAGGCGG

GGGAGTTGAGAGGTCCGCCGTTACAGTTGGACACAGGCCAACTTCTCAAGATTGCAAGACGTGGCGGCGTGACCGCAGTG

GAGGCAGTGCATGCATGGCGCAATGCACTGACGGGTGCCCCCCTGAACCTGACCCCGGACCAAGTGGTGGCCATCGCCAG

CAATAGTGGCGGCAAGCAGGCGCTGGAGACGGTGCAGCGGCTGTTGCCGGTGCTGTGCCAGGACCATGGCCTGACCCCGG

ACCAGGTCGTGGCCATCGCCAGCCACGATGGCGGCAAGCAGGCGCTGGAGACGGTGCAGCGGCTGTTGCCGGTGCTGTGC

CAGGACCATGGCCTGACCCCGGACCAGGTGGTGGCCATCGCCAGCAATGGCGGCGGCAAGCAGGCGCTGGAGACGGTGCA

GCGGCT

>M13R

CCGGACCAGGTCGTGGCCATCGCCAGCCACGATGGCGGCAAGCAGGCGCTGGAGACGGTGCAGCGGCTGTTGCCGGTGCT

GTGCCAGGACCATGGCCTGACCCCGGACCAGGTCGTGGCCATCGCCAACAATAACGGCGGCAAGCAGGCGCTGGAGACGG

TGCAGCGGCTGTTGCCGGTGCTGTGCCAGGACCATGGCCTGACCCCGGACCAGGTCGTGGCCATCGCCAGCAATGGCGGC

AAGCAGGCGCTGGAGAGCATTGTTGCCCAGTTATCTCGCCCTGATCCGGCGTTGGCCGCGTTGACCAACGACCACCTCGT

CGCCTTGGCCTGCCTCGGCGGACGTCCTGCCCTGGATGCAGTGAAAAAGGGATTGCCGCACGCGCCGGAATTGATCAGAA

GAGTCAATAGCCGTATTGGCGAACGCACGTCCCATCGCGTTGCCGACCTCGCGCACGTGGTGCGCGTGCTTGGTTTTTTC

CAGAGCCACTCCCACCCAGCGCAAGCATTCGATGACGCCATGACGCAGTTCGGGATGAGCAGGCACGGGTTGGTACAGCT

CTTTCGCAGAGTGGGCGTCACCGAATTCGAAGCCCGCTGCGGAACTATCCCCCCAGCCTCGCAGCGTTGGGACCGTATCC

TCCAGGCATCAGGGACGAAAAGGGCCAAACCGTCCCCTACTTCAGCTCAGACGCCGGATCAGGCGTCTTTGCATGCATTC

CCCGACTCGCTGGAGCGTGACCTTGATGCGCCCAGCCCAATGCACGAGGGAGATCAGACGCGGGCAAGCAGACGTAAACG

GTCCTGATCGGATCGTGCTGTCACCGACCCCTCCGCACAGCAATCTTTCGAGGTGCGCGTTCCCGAACAGCACGATGCGC

TGCATTTGCCCCTCAGCTGGAGGGTAAAACGCCCGCGTACCAGGATCGGGGGCGGCCTCCCGGATCC

**pTAL*_Bam_*_HI_-26**

> pCC2FOS-MscI-1

AATAACGGCGGCAAGCAGGCGCTGGAGACGGTGCAGCGGCTGTTGCCGGTGCTGTGCCAGGCCCATGGCCTGACCCCGGA

CCAAGTGGTGGCCATCGCCAGCAATGGCGGCAAGCAGGCGCTGGAGACGGTGCAGCGGCTGTTGCCGGTGCTGTGCCAGG

CCCATGGCCTGACCCCGGACCAGGTCGTGGCCATCGCCAGCAATGGCGGCGGCAAGCAGGCGCTGGAGACGGTGCAGCGG

CTGTTGCCGGTGCTGTGCCAGGCCCATGGCCTGACCCCGGCCCAGGTGGTGGCCATCGCCAGCAATAGTGGCGGCAAGCA

GGCGCTGGAGACGGTGCAGCGGCTGTTGCCGGTGCTGTGCCAGGACCATGGCCTGACCCCGGCCCAAGTGGTGGCCATCG

CCAACAATAACGGCGGCAAGCAGGCGCTGGAGACGGTGCAGCGGCTGTTTCCGGTGCTGTGCCAGGACCATGGCCTGACC

CCGGACCAGGTGGTGACCATCGCCAACAATAACGGCGGCAAGCAGGCGCTGGAGACGGTGCAGCGGCTGTTGCCGGTGCT

GTGCCAGGCCCATGGCTTGATCCCGGACCAGGTGGTGGCCATCGCCAACAATAACGGCGGCAAGCAGGCGCTGGAGACGG

TGCAGCGGCTGTTGCCGGTGCTGTGCCAGGCCCATGGCCTGACCCCGGCCCAAGTGGTGGCCATCGCCAGCAATATTGGC

GGCAAGCAGGCGCTGGAGACGGTGCAGCGGCTGTTGCCGGTGCTGTGCCGGGCCCATGGCCTGACCCCGGCCCAAGTGGT

GGCCATCGCCAACAATAACGGCGGCAAGCAGGCGCTGGAGACGGTGCAGCGGCTGTTGCCGGTGCTGTGCCAGGACCATG

GCCTGACCCCGGATCAAGTGGTGGCCATCGCCAGCAATATTGGCGGCAAGCAGGCGCTGGAGACGGTGCAGCGCCTG

> pCC2FOS-MscI-2

CCATCGCCAGCAATAGTGGCGGCAAGCAGGCGCTGGAGACGGTGCAGCGGCTGTTGCCGGTGCTGTGCCAGGACCATGGC

CTGACCCCGGCCCAAGTGGTGGCCATCGCCAACAATAACGGCGGCAAGCAGGCGCTGGAGACGGTGCAGCGGCTGTTTCC

GGTGCTGTGCCAGGACCATGGCCTGACCCCGGACCAGGTGGTGACCATCGCCAACAATAACGGCGGCAAGCAGGCGCTGG

AGACGGTGCAGCGGCTGTTGCCGGTGCTGTGCCAGGCCCATGGCTTGATCCCGGACCAGGTGGTGGCCATCGCCAACAAT

AACGGCGGCAAGCAGGCGCTGGAGACGGTGCAGCGGCTGTTGCCGGTGCTGTGCCAGGCCCATGGCCTGACCCCGGCCCA

AGTGGTGGCCATCGCCAGCAATATTGGCGGCAAGCAGGCGCTGGAGACGGTGCAGCGGCTGTTGCCGGTGCTGTGCCGGG

CCCATGGCCTGACCCCGGCCCAAGTGGTGGCCATCGCCAACAATAACGGCGGCAAGCAGGCGCTGGAGACGGTGCAGCGG

CTGTTGCCGGTGCTGTGCCAGGACCATGGCCTGACCCCGGATCAAGTGGTGGCCATCGCCAGCAATATTGGCGGCAAGCA

GGCGCTGGAGACGGTGCAGCGCCTGTTGCCGGTGCTGTGCCAGGCCCATGGCCTGACCCCGGACCAGGTCGTGGCCATCG

CCAGCAATGGCGGCGGCAAGCAGGCGCTGGAGACGGTGCAGCGGCTGTTGCCGGTGCTGTGCCAGGACCATGGCCTGACC

CCGGACCAGGTCGTGGCCATCGCCGGCCACGATGGCGGCAAGCAGGCGCTGGAGACGGTGCAGCGGCTGTTGCCGGTGCT

GTGCCAGGAC

> pCC2FOS-MscI-3

ATATTGGCGGCAACCAGGCGCTGGAGACGGTGCAGCGGCTGTTGCCGGTGCTGTGCCAGGACCATGGCCTGACCCCGGAC

CAAGTGGTGGCCATCGCCAACAATAACGGCGGCAAGCAGGCGCTGGAGACGGTGCAGCGGCTGTTGCCGGTGCTGTGCCA

GGCCCATGGCCTGACCCCGGACCAAGTGGTGGCCATCGCCAGCAATGGCGGCAAGCAGGCGCTGGAGACGGTGCAGCGGC

TGTTGCCGGTGCTGTGCCAGGCCCATGGCCTGACCCCGGACCAGGTCGTGGCCATCGCCAGCAATGGCGGCGGCAAGCAG

GCGCTGGAGACGGTGCAGCGGCTGTTGCCGGTGCTGTGCCAGGCCCATGGCCTGACCCCGGCCCAGGTGGTGGCCATCGC

CAGCAATAGTGGCGGCAAGCAGGCGCTGGAGACGGTGCAGCGGCTGTTGCCGGTGCTGTGCCAGGACCATGGCCTGACCC

CGGCCCAAGTGGTGGCCATCGCCAACAATAACGGCGGCAAGCAGGCGCTGGAGACGGTGCAGCGGCTGTTTCCGGTGCTG

TGCCAGGACCATGGCCTGACCCCGGACCAGGTGGTGACCATCGCCAACAATAACGGCGGCAAGCAGGCGCTGGAGACGGT

GCAGCGGCTGTTGCCGGTGCTGTGCCAGGCCCATGGCTTGATCCCGGACCAGGTGGTGGCCATCGCCAACAATAACGGCG

GCAAGCAGGCGCTGGAGACGGTGCAGCGGCTGTTGCCGGTGCTGTGCCAGGCCCATGGCCTGACCCCGGCCCAAGTGGTG

GCCATCGCCAGCAATATTGGCGGCAAGCAGGCGCTGGAGACGGTGCAGCGGCTGTTGCCGGTGCTGTGCCGGGCCCATGG

CCTGACCCCGGCCCAAGTGGTGGCCATCGCCAACAATAACGGCGGCAAGCAGGCGCTGGAGACGGTGCAGCGGCTGTTGC

CG

>26P1F_1

AGGCGGGGGAGTTGAGAGGTCCGCCGTTACAGTTGGACACAGGCCAACTTCTCAAGATTGCAAAACGTGGCGGCGTGACC

GCAGTGGAGGCAGTGCATGCATCGCGCAATGCACTGACGGGTGCCCCCCTGAACCTGACCCCGGACCAAGTGGTGGCCAT

CGCCAGCAATATTGGCGGCAACCAGGCGCTGGAGACGGTGCAGCGGCTGTTGCCGGTGCTGTGCCAGGACCATGGCCTGA

CCCCGGACCAAGTGGTGGCCATCGCCAACAATAACGGCGGCAAGCAGGCGCTGGAGACGGTGCAGCGGCTGTTGCCGGTG

CTGTGCCAGGCCCATGGCCTGACCCCGGACCAAGTGGTGGCCATCGCCAGCAATGGCGGCAAGCAGGCGCTGGAGACGGT

GCAGCGGCTGTTGCCGGTGCTGTGCCAGGCCCATGGCCTGACCCCGGACCAGGTCGTGGCCATCGCCAGCAATGGCGGCG

GCAAGCAGGCGCTGGAGACGGTGCAGCGGCTGTTGCCGGTGCTGTGCCAGGCCCATGGCCTGACCCCGGCCCAGGTGGTG

GCCATCGCCAGCAATAGTGGCGGCAAGCAGGCGCTGGAGACGGTGCAGCGGCTGTTGCCGGTGCTGTGCCAGGACCATGG

CCTGACCCCGGCCCAAGTGGTGGCCATCGCCAACAATAACGGCGGCAAGCAGGCGCTGGAGACGGTGCAGCGGCTGTTTC

CGGTGCTGTGCCAGGACCATGGCCTGACCCCGGACCAGGTGGTGACCATCGCCAACAATAACGGCGGCAAGCAGGCGCTG

GAGACGGTGCAGCGGCTGTTGCCGGTGCTGTGCCAGGCCCATGGCTTGATCCCGGACCAGGTGGTGGCCATCGCCAACAA

TAACGGCGGCAAGCAGGCGCTGGAGACGGTGCAGCGGCTGTTGCCGGTGCTGTGCCAGGCCCATGGCCTGACCCCGGCCC

AAGTGGTGGCCATCGCCAGCAA

>26P1F_2

AGTTGAGAGGTCCGCCGTTACAGTTGGACACAGGCCAACTTCTCAAGATTGCAAAACGTGGCGGCGTGACCGCAGTGGAG

GCAGTGCATGCATCGCGCAATGCACTGACGGGTGCCCCCCTGAACCTGACCCCGGACCAAGTGGTGGCCATCGCCAGCAA

TATTGGCGGCAACCAGGCGCTGGAGACGGTGCAGCGGCTGTTGCCGGTGCTGTGCCAGGACCATGGCCTGACCCCGGACC

AAGTGGTGGCCATCGCCAACAATAACGGCGGCAAGCAGGCGCTGGAGACGGTGCAGCGGCTGTTGCCGGTGCTGTGCCAG

GCCCATGGCCTGACCCCGGACCAAGTGGTGGCCATCGCCAGCAATGGCGGCAAGCAGGCGCTGGAGACGGTGCAGCGGCT

GTTGCCGGTGCTGTGCCAGGCCCATGGCCTGACCCCGGACCAGGTCGTGGCCATCGCCAGCAATGGCGGCGGCAAGCAGG

CGCTGGAGACGGTGCAGCGGCTGTTGCCGGTGCTGTGCCAGGCCCATGGCCTGACCCCGGCCCAGGTGGTGGCCATCGCC

AGCAATAGTGGCGGCAAGCAGGCGCTGGAGACGGTGCAGCGGCTGTTGCCGGTGCTGTGCCAGGACCATGGCCTGACCCC

GGCCCAAGTGGTGGCCATCGCCAACAATAACGGCGGCAAGCAGGCGCTGGAGACGGTGCAGCGGCTGTTTCCGGTGCTGT

GCCAGGACCATGGCCTGACCCCGGACCAGGTGGTGACCATCGCCAACAATAACGGCGGCAAGCAGGCGCTGGAGACGGTG

CAGCGGCTGTTGCCGGTGCTGTGCCAGGCCCATGGCTTGATCCCGGACCAGGTGGTGGCCATCGCCAACAATAACGGCGG

CAAGCAGGCGCTGGAGACGGTGCAGCGGCTGTTGCCGGTGCTGTGCCAGGCCCATGGCCTGACCCCGGCCCAAGTGGTGG

CCATCGCCAGCAA

>26P1R_1

GAGATAACTGGGCAACAATGCTCTCCAGCGCCTGCTTGCCGCCGCCATTGCTGGCGATGGCCACGACCTGGTCCGGGGTC

AGGCCATGGTCCTGGCACAGCACCGGCAACAGCCGCTGCACCGTCTCCAGCGCCTGCTTGCCGCCAATATTGCTGGCGAT

GGCCACCACCTGGTCCAGGGTCAGGCCATGGTCCTGGCACAGCACCGGCAACAGCCGCTGCACCGTCTCCAGCGCCTGCT

TGCCGCCATCGTGGCTGGCGATGGCCACGACCTGGTCCGGGGTCAGGCCATGGTCCTGGCACAGCACCGGCAACAGCCGC

TGCACCGTCTCCAGCGCCTGCTTGCCGCCATCGTGGCCGGCGATGGCCACGACCTGGTCCGGGGTCAGGCCATGGTCCTG

GCACAGCACCGGCAACAGCCGCTGCACCGTCTCCAGCGCCTGCTTGCCGCCGCCATTGCTGGCGATGGCCACGACCTGGT

CCGGGGTCAGGCCATGGGCCTGGCACAGCACCGGCAACAGGCGCTGCACCGTCTCCAGCGCCTGCTTGCCGCCAATATTG

CTGGCGATGGCCACCACTTGATCCGGGGTCAGGCCATGGTCCTGGCACAGCACCGGCAACAGCCGCTGCACCGTCTCCAG

CGCCTGCTTGCCGCCGTTATTGTTGGCGATGGCCACCACTTGGGCCGGGGTCAGGCCATGGGCCCGGCACAGCACCGGCA

ACAGCCGCTGCACCGTCTCCAGCGCCTGCTTGCCGCCAATATTGCTGGCGATGGCCACCACTTGGGCCGGGGTCAGGCCA

TGGGCCTGGCACAGCACCGGCAACAGCCGCTGCACCGTCTCCAGCGCCTGCTTGCCGCCGTTATTGTTGGCGATGGCCAC

CACCTGGTCCGGGATCAAGCCATGGGCCTGGCACAGCACCGGCAACAGCCGCTGCACCGTCTCCAGCGCCTGCTTGCCGC

CGTTATTGTTGGCGATGGTCA

>26P1R_2

GTGGTGACCATCGCCAACAATAACGGCGGCAAGCAGGCGCTGGAGACGGTGCAGCGGCTGTTGCCGGTGCTGTGCCAGGC

CCATGGCTTGATCCCGGACCAGGTGGTGGCCATCGCCAACAATAACGGCGGCAAGCAGGCGCTGGAGACGGTGCAGCGGC

TGTTGCCGGTGCTGTGCCAGGCCCATGGCCTGACCCCGGCCCAAGTGGTGGCCATCGCCAGCAATATTGGCGGCAAGCAG

GCGCTGGAGACGGTGCAGCGGCTGTTGCCGGTGCTGTGCCGGGCCCATGGCCTGACCCCGGCCCAAGTGGTGGCCATCGC

CAACAATAACGGCGGCAAGCAGGCGCTGGAGACGGTGCAGCGGCTGTTGCCGGTGCTGTGCCAGGACCATGGCCTGACCC

CGGATCAAGTGGTGGCCATCGCCAGCAATATTGGCGGCAAGCAGGCGCTGGAGACGGTGCAGCGCCTGTTGCCGGTGCTG

TGCCAGGCCCATGGCCTGACCCCGGACCAGGTCGTGGCCATCGCCAGCAATGGCGGCGGCAAGCAGGCGCTGGAGACGGT

GCAGCGGCTGTTGCCGGTGCTGTGCCAGGACCATGGCCTGACCCCGGACCAGGTCGTGGCCATCGCCGGCCACGATGGCG

GCAAGCAGGCGCTGGAGACGGTGCAGCGGCTGTTGCCGGTGCTGTGCCAGGACCATGGCCTGACCCCGGACCAGGTCGTG

GCCATCGCCAGCCACGATGGCGGCAAGCAGGCGCTGGAGACGGTGCAGCGGCTGTTGCCGGTGCTGTGCCAGGACCATGG

CCTGACCCTGGACCAGGTGGTGGCCATCGCCAGCAATATTGGCGGCAAGCAGGCGCTGGAGACGGTGCAGCGGCTGTTGC

CGGTGCTGTGCCAGGACCATGGCCTGACCCCGGACCAGGTCGTGGCCATCGCCAGCAATGGCGGCGGCAAGCAGGCGCTG

GAGAGCATTG

>26P2F_1

CGCGCACATCGTTGCGCTCAGCCAACACCCGGCAGCGTTAGGGACCGTCGCTGTCAAGTATCAGCACATAATCACGGCGT

TGCCAGAGGCGACACACGAAGACATCGTTGGCGTCGGCAAACAGTGGTCCGGCGCACGCGCCCTGGAGGCCTTGCTCACG

AAGGCGGGGGAGTTGAGAGGTCCGCCGTTACAGTTGGACACAGGCCAACTTCTCAAGATTGCAAAACGTGGCGGCGTGAC

CGCAGTGGAGGCAGTGCATGCATCGCGCAATGCACTGACGGGTGCCCCCCTGAACCTGACCCCGGACCAAGTGGTGGCCA

TCGCCAGCAATATTGGCGGCAACCAGGCGCTGGAGACGGTGCAGCGGCTGTTGCCGGTGCTGTGCCAGGACCATGGCCTG

ACCCCGGACCAAGTGGTGGCCATCGCCAACAATAACGGCGGCAAGCAGGCGCTGGAGACGGTGCAGCGGCTGTTGCCGGT

GCTGTGCCAGGCCCATGGCCTGACCCCGGACCAAGTGGTGGCCATCGCCAGCAATGGCGGCAAGCAGGCGCTGGAGACGG

TGCAGCGGCTGTTGCCGGTGCTGTGCCAGGCCCATGGCCTGACCCCGGACCAGGTCGTGGCCATCGCCAGCAATGGCGGC

GGCAAGCAGGCGCTGGAGACGGTGCAGCGGCTGTTGCCGGTGCTGTGCCAGGCCCATGGCCTGACCCCGGCCCAGGTGGT

GGCCATCGCCAGCAATAGTGGCGGCAAGCAGGCGCTGGAGACGGTGCAGCGGCTGTTGCCGGTGCTGTGCCAGGACCATG

GCCTGACCCCGGCCCAAGTGGTGGCCATCGCCAACAATAACGGCGGCAAGCAGGCGCTGGAGACGGTGCAGCGGCTGTTT

CCGGTGCTGTGCCAGGACCATGGCCTGACCCCGGACCAGGTGGTGACCATCGCCAACAATAACGGCGGCAAGCAGGCGCT

GGAGACGGTGC

>26P2F_2

TTGCGCTCAGCCAACACCCGGCAGCGTTAGGGACCGTCGCTGTCAAGTATCAGCACATAATCACGGCGTTGCCAGAGGCG

ACACACGAAGACATCGTTGGCGTCGGCAAACAGTGGTCCGGCGCACGCGCCCTGGAGGCCTTGCTCACGAAGGCGGGGGA

GTTGAGAGGTCCGCCGTTACAGTTGGACACAGGCCAACTTCTCAAGATTGCAAAACGTGGCGGCGTGACCGCAGTGGAGG

CAGTGCATGCATCGCGCAATGCACTGACGGGTGCCCCCCTGAACCTGACCCCGGACCAAGTGGTGGCCATCGCCAGCAAT

ATTGGCGGCAACCAGGCGCTGGAGACGGTGCAGCGGCTGTTGCCGGTGCTGTGCCAGGACCATGGCCTGACCCCGGACCA

AGTGGTGGCCATCGCCAACAATAACGGCGGCAAGCAGGCGCTGGAGACGGTGCAGCGGCTGTTGCCGGTGCTGTGCCAGG

CCCATGGCCTGACCCCGGACCAAGTGGTGGCCATCGCCAGCAATGGCGGCAAGCAGGCGCTGGAGACGGTGCAGCGGCTG

TTGCCGGTGCTGTGCCAGGCCCATGGCCTGACCCCGGACCAGGTCGTGGCCATCGCCAGCAATGGCGGCGGCAAGCAGGC

GCTGGAGACGGTGCAGCGGCTGTTGCCGGTGCTGTGCCAGGCCCATGGCCTGACCCCGGCCCAGGTGGTGGCCATCGCCA

GCAATAGTGGCGGCAAGCAGGCGCTGGAGACGGTGCAGCGGCTGTTGCCGGTGCTGTGCCAGGACCATGGCCTGACCCCG

GCCCAAGTGGTGGCCATCGCCAACAATAACGGCGGCAAGCAGGCGCTGGAGACGGTGCAGCGGCTGTTTCCGGTGCTGTG

CCAGGACCATGGCCTGACCCCGGACCAGGTGGTGACCATCGCCAACAATAACGGCGGCAAGCAGGCGCTGGAGACGGTGC

>26P2R_1

GTGATTATGTGCTGATACTTGACAGCGACGGTCCCTAACGCTGCCGGGTGTTGGCTGAGCGCAACGATGTGCGCGTGTGT

AAAACCATGGCCCACCAGTGCCTCGTGGTGCTGCGCCACTGTCGAACGCACCTTCGGTTTGATCTTCTCTTGCTGCTGCT

GACTGTAGCCGAGCGTGCGTAGATCCACCTGCGCGGCCGGCGAAGCGTCGGAGGGTTGCGCCGCACGCCGTCGCGGGGCC

GGCTTTGCGCGCGGCGGCCGCGCGGCAGTGACAGCGACACGCACGGTGGGTGGCGGGTCATCGGCTGCACGCAGACCCGA

TTGCACCTCATCCCACTCTGCTGGGGCAGCCGCTGTATGCGGCGTGCCGACGGCAGGCATCGAATCAAGAAGCGATGTAT

CAAGAAGCGACGGATCGAACTGACGGAGCAGATCGTTGAAGCTGCCCGCCGAGAACGCAGGCGAGGGCGCAGGGGGAGAT

GGCAGCCGGGTCCGGGACATCGTCCGCCGAGCGGGCAAGCCATCCAGGGGGCCGCCAGCAGGCGGAGCCCCCCCCCGATC

TGCAGTCGGCTGAACCCTATCCGGTTGGGGTCCGGGCAGAAGCTCGCGGGCAGGACTTGGCGTGCGCGAACGAATGGGAT

CC

>26P2R_2

GGATCCCATTCGTTCGCGCACGCCAAGTCCTGCCCGCGAGCTTCTGCCCGGACCCCAACCGGATAGGGTTCAGCCGACTG

CAGATCGGGGGGGGGCTCCGCCTGCTGGCGGCCCCCTGGATGGCTTGCCCGCTCGGCGGACGATGTCCCGGACCCGGCTG

CCATCTCCCCCTGCGCCCTCGCCTGCGTTCTCGGCGGGCAGCTTCAACGATCTGCTCCGTCAGTTCGATCCGTCGCTTCT

TGATACATCGCTTCTTGATTCGATGCCTGCCGTCGGCACGCCGCATACAGCGGCTGCCCCAGCAGAGTGGGATGAGGTGC

AATCGGGTCTGCGTGCAGCCGATGACCCGCCACCCACCGTGCGTGTCGCTGTCACTGCCGCGCGGCCGCCGCGCGCAAAG

CCGGCCCCGCGACGGCGTGCGGCGCAACCCTCCGACGCTTCGCCGGCCGCGCAGGTGGATCTACGCACGCTCGGCTACAG

TCAGCAGCAGCAAGAGAAGATCAAACCGAAGGTGCGTTCGACAGTGGCGCAGCACCACGAGGCACTGGTGGGCCATGGTT

TTACACACGCGCACATCGTTGCGCTCAGCCAACACCCGGCAGCGTTAGGGACC

>26P3F

TGCCGCACGCGCCGGAATTGATCAGAAGAATCAATCGCCGTATTCCCGAACGCACGTCCCATCGCGTTGCCGACTACGCG

CAAGTGGTTCGCGTGCTGGAGTTTTTCCAGTGCCACTCCCACCCAGCGTACGCATTTGATGAGGCCATGACGCAGTTCGG

GATGAGCAGGAACGGGTTGGTACAGCTCTTTCGCAGAGTGGGCGTCACCGAACTCGAAGCCCGCGGTGGAACGCTCCCCC

CAGCCTCGCAGCGTTGGGACCGTATCCTCCAGGCATCAGGGATGAAAAGGGCCAAACCGTCCCCTACTTCAGCTCAAACA

CCGGATCAGGCGTCTTTGCATGCATTCGCCGATTCGCTGGAGCGTGACCTTGATGCGCCTAGCCCAATGCACGAGGGAGA

TCAGACAGGGGCAAGCAGCCGTAAACGGTCCCGATCGGATCGTGCTGTCACCGGCCCCTCCGCACAGCAATCTTTCGAGG

TGCGCGTTCCCGAACAGCGCGATGCGCTGCATTTGCCCCTCAGCTGGAGGGTAAAACGCCCGCGTACCAGGATCGGGGGC

GGCCTCCCGGATCC

>26P3R

GGGCTTCGAGTTCGGTGACGCCCACTCTGCGAAAGAGCTGTACCAACCCGTTCCTGCTCATCCCGAACTGCGTCATGGCC

TCATCAAATGCGTACGCTGGGTGGGAGTGGCACTGGAAAAACTCCAGCACGCGAACCACTTGCGCGTAGTCGGCAACGCG

ATGGGACGTGCGTTCGGGAATACGGCGATTGATTCTTCTGATCAATTCCGGCGCGTGCGGCAATCCCTTTTTCACTGCAT

CCAGGGCAGGACGTCCGCCGAGGCAGGCCAAGGCGACGAGGTGGTCGTTGGTCAACGCGGCCAACGCCGGATCAGGGCGA

GATAACTGGGCAACAATGCTCTCCAGCGCCTGCTTGCCGCCGCCATTGCTGGCGATGGCCACGACCTGGTCCGGGGTCAG

GCCATGGTCCTGGCACAGCACCGGCAACAGCCGCTGCACCGTCTCCAGCGCCTGCTTGCCGCCAATATTGCTGGCGATGG

CCACCACCTGGTCCAGGGTCAGGCCATGGTCCTGGCACAGCACCGGCAACAGCCGCTGCACCGTCTCCAGCGCCTGCTTG

CCGCCATCGTGGCTGGCGATGGCCACGACCTGGTCCGGGGTCAGGCCATGGTCCTGGCACAGCACCGGCAACAGCCGCTG

CACCGTCTCCAGCGCCTGCTTGCCGCCATCGTGGCCGGCGATGGCCACGACCTGGTCCGGGGTCAGGCCATGGTCCTGGC

ACAGCACCGGCAACAGCCGCTGCACCGTCTCCAGCGCCTGCTTGCCGCCGCCATTGCTGGCGATGGCCACGACCTGGTCC

GGGGTCAGGCCATGGGCCTGGCACAGCACCGGCAACAGGCGCTGCACCGTCTCCAGCGCCTGCTTGCCGCCAATATTGCT

GGCGATGGCCACCACTTGATCCGGGGTCAGGCCATGGTCCTGGCACAGCACCGGCAACAGCCGCTGCACCGTCTCCAGCG

CCTGCTTGCCGCCGTTATTGTT

>M13R

CGGACCAGGTCGTGGCCATCGCCAGCCACGATGGCGGCAAGCAGGCGCTGGAGACGGTGCAGCGGCTGTTGCCGGTGCTG

TGCCAGGACCATGGCCTGACCCTGGACCAGGTGGTGGCCATCGCCAGCAATATTGGCGGCAAGCAGGCGCTGGAGACGGT

GCAGCGGCTGTTGCCGGTGCTGTGCCAGGACCATGGCCTGACCCCGGACCAGGTCGTGGCCATCGCCAGCAATGGCGGCG

GCAAGCAGGCGCTGGAGAGCATTGTTGCCCAGTTATCTCGCCCTGATCCGGCGTTGGCCGCGTTGACCAACGACCACCTC

GTCGCCTTGGCCTGCCTCGGCGGACGTCCTGCCCTGGATGCAGTGAAAAAGGGATTGCCGCACGCGCCGGAATTGATCAG

AAGAATCAATCGCCGTATTCCCGAACGCACGTCCCATCGCGTTGCCGACTACGCGCAAGTGGTTCGCGTGCTGGAGTTTT

TCCAGTGCCACTCCCACCCAGCGTACGCATTTGATGAGGCCATGACGCAGTTCGGGATGAGCAGGAACGGGTTGGTACAG

CTCTTTCGCAGAGTGGGCGTCACCGAACTCGAAGCCCGCGGTGGAACGCTCCCCCCAGCCTCGCAGCGTTGGGACCGTAT

CCTCCAGGCATCAGGGATGAAAAGGGCCAAACCGTCCCCTACTTCAGCTCAAACACCGGATCAGGCGTCTTTGCATGCAT

TCGCCGATTCGCTGGAGCGTGACCTTGATGCGCCTAGCCCAATGCACGAGGGAGATCAGACAGGGGCAAGCAGCCGTAAA

CGGTCCCGATCGGATCGTGCTGTCACCGGCCCCTCCGCACAGCAATCTTTCGAGGTGCGCGTTCCCGAACAGCGCGATGC

GCTGCATTTGCCCCTCAGCTGGAGGGTAAAACGCCCGCGTACCAGGATCGGGGGCGGCCTCCCGGATCC

**pTAL*_Bam_*_HI_-27**

> pCC2FOS-MscI-1

CCATCGCCAGCCACGATGGCGGCAAGCAGGCGCTGGAGACGATGCAGCGGCTGTTGCCGGTGCTGTGCCAGGCCCATGGC

CTGACCCCGGCGCAGGTGGTGGCCATCGCCAGCAATAGCGGCGGCAAGCAGGCGCTGGAGACGGTGCAGCGGCTGTTGCC

GGTGCTGTGCCAGGCCCATGGCCTGACCCCGGCGCAGGTGGTGGCCATCGTCAGCCACGATGGCGGCAAGCAGGCGCTGG

AGACGGTGCAGCGGCTGTTGCCGGTGCTGTGCCAGGCCCATGGTCTGACCCCGGACCAGGTGGTGGCCATCGCCAGCAAT

AACGGCGGCAAGCAGGCGCTGGAGACGGTGCAGCGGCTGTTGCCGGTGCTGTGCCAGGACCATGGCCTGACCCCGGACCA

GGTGGTGGCCATCGCCAGCCACGATGGCGGCAAGCAGGCGCTGGGGACGGTGCAGCGGCTGTTGCCGGTGCTGTGCCAGG

ACCATGGCCTGACCCCGGACCAGGTGGTGGCCATCGCCAGCAATAACGGCGGCAAGCAGGCGCTGGAGACGGTGCAGCGG

CTGTTGCCGGTGCTGTGCCAGGACCATGGCCTGACCCCGGACCAGGTGGTGGCCATCGCCAGCCACGATGGCGGTAAGCA

GGCGCTGGAGACGGTGCAGCGGCTGTTGCCGGTGCTGTGCCAGGCCCATGGCCTGACCCCGGCGCAGGTGGTGGCCATCG

CCAGCAATAACGGCGGCAAGCAGGCGCTGGAGACGGTGCAGCGGCTGTTGCCGGTGCTGTGCCAGGACCATGGTCTGACC

CCGGACCAGGTGGTAGCCATCGCCAACAATAACGGCGGCAAGCAGGCGCTGGAGACGGTGCAGCGGCTGTTGCCAGTGCT

GTGCCAGG

> pCC2FOS-MscI-2

ATGGCGGTAAGCAGGCGCTGGAGACGGTGCAGCGGCTGTTGCCGGTGCTGTGCCAGGCCCATGGCCTGACCCCGGCGCAG

GTGGTGGCCATCGCCAGCAATAACGGCGGCAAGCAGGCGCTGGAGACGGTGCAGCGGCTGTTGCCGGTGCTGTGCCAGGA

CCATGGTCTGACCCCGGACCAGGTGGTAGCCATCGCCAACAATAACGGCGGCAAGCAGGCGCTGGAGACGGTGCAGCGGC

TGTTGCCAGTGCTGTGCCAGGCCCATGGCCTGACCCCGGCGCAGGTGGTGGCCATCGCCAGCAATAACGGCAGCAAGCAG

GCGCTGGAGACGGTGCAGCGGCTGTTGCCGGTGCTGTGCCAGGCCCATGGCCTGACCCCGGCGCAGGTGGTGGCCATCGC

CAGCAATAACGGCGGCAAGCAGGCGCTGGAGACGGTGCAGCGGCTGTTGCCGGTGCTGTGCCAGGCCCATGGTCTGACCC

CGGACCAGGTGGTGGCCATCGCCAGCCACGATGGCGGCAAGCAGGCGCTGGAGACGGTGCAGCGGCTGTTGCCGGTGCTG

TGCCAGGCCCATGGCCTGACCCCGGACCAGGTGGTGGCCATCGCCAGCAATAACGGCGGCAAGCAGGCGCTGGAGACGGT

GCAGCGGCTGTTGCCGGTGCTGTGCCAGGCCCATGGCCTGACCCCGGACCAGGTGGTGGCCATCGCCAGCAATAACGGCG

GCAAGCAGGCGCTGGAGACGGTGCAGCGGCTGTTGCCGGT

> pCC2FOS-MscI-3

CCATCGTCAGCCACGATGGCGGCAAGCAGGCGCTGGAGACGGTGCAGCGGCTGTTGCCGGTGCTGTGCCAGGCCCATGGT

CTGACCCCGGACCAGGTGGTGGCCATCGCCAGCAATAACGGCGGCAAGCAGGCGCTGGAGACGGTGCAGCGGCTGTTGCC

GGTGCTGTGCCAGGACCATGGCCTGACCCCGGACCAGGTGGTGGCCATCGCCAGCCACGATGGCGGCAAGCAGGCGCTGG

GGACGGTGCAGCGGCTGTTGCCGGTGCTGTGCCAGGACCATGGCCTGACCCCGGACCAGGTGGTGGCCATCGCCAGCAAT

AACGGCGGCAAGCAGGCGCTGGAGACGGTGCAGCGGCTGTTGCCGGTGCTGTGCCAGGACCATGGCCTGACCCCGGACCA

GGTGGTGGCCATCGCCAGCCACGATGGCGGTAAGCAGGCGCTGGAGACGGTGCAGCGGCTGTTGCCGGTGCTGTGCCAGG

CCCATGGCCTGACCCCGGCGCAGGTGGTGGCCATCGCCAGCAATAACGGCGGCAAGCAGGCGCTGGAGACGGTGCAGCGG

CTGTTGCCGGTGCTGTGCCAGGACCATGGTCTGACCCCGGACCAGGTGGTAGCCATCGCCAACAATAACGGCGGCAAGCA

GGCGCTGGAGACGGTGCAGCGGCTGTTGCCAGTGCTGTGCCAGGCCCATGGCCTGACCCCGGCGCAGGTGGTGGCCATCG

CCAGCAATAACGGCAGCAAGCAGGCGCTGGAGACGGTGCAGCGGCTGTTGCCGGTGCTGTGCCAGGCCCATGGCCTGACC

CCGGCGCAGGTGGTGGCCATCGCCAGCAATAACGGCGGCAAGCAGGCGCTGGAGACGGTGCAGCGGCTGTTGCCGGTGCT

GTGCCAGGCCC

> pCC2FOS-MscI-4

CCATCGCCAGCCACGATGGCGGCAAGCAGGCACTGGAGACGGTGCAGCGGCTGTTGCCGGTGCTGTGCCAGGACCATGGC

CTGACCCCGGACCAGGTGGTGGCCATCGCCAGCAATAGTGGCGGCAAGCAGGCGCTGGAGACGGTGCAGCGGCTGTTGCC

GGTGCTGTGCCAGGACCATGGCCTGACACCGGACCAGGTGGTGGCCATCGCCAGCAATGGCGGCGGCAAGCAGGCGCTGG

AGACGGTGCAGCGGCTGTTGCCGGTGCTGTGCCAGGACCATGGCCTGACCCCGGACCAGGTGGTGGCCATCGCCAGCCAC

GATGGCGGCAAGCAGGCGCTGGAGACGGTGCAGCGGCTGTTGCCGGTGCTGTGCCAGGACCATGGCCTGACCCCGGACCA

ATTGGTGGCCATCGCCAACAATAACGGCGGCAAGCAGGCGCTGGAGACGGTGCAGCGGCTGTTGCCGGTGCTGTGCCAGG

ACCATGGCCTGACCCCGGACCAGGTCGTGGCCATCGCCAGCAATGGCGGCAAGCAGGCGCTGGAGACAGTGCAGCGGCTG

TTGCCGGTGCTGTGCCAGGACCATGGTCTGACCCCGGACCAGGTCGTGGCCATCGCCAGCAATATTGGCGGCAAGCAGGC

GCTGGAGACGGTGCAGCGGCTGTTGCCGGTGCTGTGCCAGGCCCATGGTCTGACCCCGGCGCAGGTGGTGGCCATCGCCA

GCCACGATGGCGGCAAGCAGGCGCTGGAGACGATGCAGCGGCTGTTGCCGGTGCTGTGCCAGGCCCATGGCCTGACCCCG

GCGCAGGTGGTGGCCATCGCCAGCAATAGCGGCGGCAAGCAGGCGCTGGAGACGGTGCAGCGGCTGTTGCCGGTGCTGTG

CCAGGCCCA

> pCC2FOS-MscI-5

CCATCGCCAGCAATGGCGGCAAGCAGGCGCTGGAGACAGTGCAGCGGCTGTTGCCGGTGCTGTGCCAGGACCATGGTCTG

ACCCCGGACCAGGTCGTGGCCATCGCCAGCAATATTGGCGGCAAGCAGGCGCTGGAGACGGTGCAGCGGCTGTTGCCGGT

GCTGTGCCAGGCCCATGGTCTGACCCCGGCGCAGGTGGTGGCCATCGCCAGCCACGATGGCGGCAAGCAGGCGCTGGAGA

CGATGCAGCGGCTGTTGCCGGTGCTGTGCCAGGCCCATGGCCTGACCCCGGCGCAGGTGGTGGCCATCGCCAGCAATAGC

GGCGGCAAGCAGGCGCTGGAGACGGTGCAGCGGCTGTTGCCGGTGCTGTGCCAGGCCCATGGCCTGACCCCGGCGCAGGT

GGTGGCCATCGTCAGCCACGATGGCGGCAAGCAGGCGCTGGAGACGGTGCAGCGGCTGTTGCCGGTGCTGTGCCAGGCCC

ATGGTCTGACCCCGGACCAGGTGGTGGCCATCGCCAGCAATAACGGCGGCAAGCAGGCGCTGGAGACGGTGCAGCGGCTG

TTGCCGGTGCTGTGCCAGGACCATGGCCTGACCCCGGACCAGGTGGTGGCCATCGCCAGCCACGATGGCGGCAAGCAGGC

GCTGGGGACGGTGCAGCGGCTGTTGCCGGTGCTGTGCCAGGACCATGGCCTGACCCCGGACCAGGTGGTGGCCATCGCCA

GCAATAACGGCGGCAAGCAGGCGCTGGAGACGGTGCAGCGGCTGTTGCCGGTGC

> pCC2FOS-MscI-6

GGTCCTGGCACAGCACCGGCAACAGCCGCTGCACCGTCTCCAGCGCCTGCTTGCCGCCGTTATTGCTGGCGATGGCCACC

ACCTGGTCCGGGGTCAGGCCATGGGCCTGGCACAGCACCGGCAACAGCCGCTGCACCGTCTCCAGCGCCTGCTTGCCGCC

GTTATTGCTGGCGATGGCCACCACCTGGTCCGGGGTCAGGCCATGGGCCTGGCACAGCACCGGCAACAGCCGCTGCACCG

TCTCCAGCGCCTGCTTGCCGCCATCGTGGCTGGCGATGGCCACCACCTGGTCCGGGGTCAGACCATGGGCCTGGCACAGC

ACCGGCAACAGCCGCTGCACCGTCTCCAGCGCCTGCTTGCCGCCGTTATTGCTGGCGATGGCCACCACCTGCGCCGGGGT

CAGGCCATGGGCCTGGCACAGCACCGGCAACAGCCGCTGCACCGTCTCCAGCGCCTGCTTGCTGCCGTTATTGCTGGCGA

TGGCCACCACCTGCGCCGGGGTCAGGCCATGGGCCTGGCACAGCACTGGCAACAGCCGCTGCACCGTCTCCAGCGCCTGC

TTGCCGCCGTTATTGTTGGCGATGGCTACCACCTGGTCCGGGGTCAGACCATGGTCCTGGCACAGCACCGGCAACAGCCG

CTGCACCGTCTCCAGCGCCTGCTTGCCGCCGTTATTGCTGGCGATGGCCACCACCTGCGCCGGGGTCAGGCCATGGGCCT

GGCACAGCACCGGCAACAGCCGCTGCACCGTCTCCAGCGCCTGCTTACCGCCATCGTGGCTGGCGATGG

>27P1F_1

GCGGGGGAGTTGAGAGGTCCGCCGTTACAGTTGGACACAGGCCAACTTCTCAAGATTGCAAAACGTGGCGGCGTGACCGC

AGTGGAGGCAGTGCATGCATCGCGCAATGCACTGACGGGTGCCCCCCTGAACCTGACCCCGGCACAGGTGGTGGCCATCG

CCAGCAATAACGGCGGCAAGCAGGCGCTGGAGACGGTGCAGCGGCTGTTGCCGTTGCTGTGCCAGGCCCATGGCCTGACC

CCGGCGCAGGTGGTGGCCATCGCCAGCCACGATGGCGGCAAGCAGGCACTGGAGACGGTGCAGCGGCTGTTGCCGGTGCT

GTGCCAGGACCATGGCCTGACCCCGGACCAGGTGGTGGCCATCGCCAGCAATAGTGGCGGCAAGCAGGCGCTGGAGACGG

TGCAGCGGCTGTTGCCGGTGCTGTGCCAGGACCATGGCCTGACACCGGACCAGGTGGTGGCCATCGCCAGCAATGGCGGC

GGCAAGCAGGCGCTGGAGACGGTGCAGCGGCTGTTGCCGGTGCTGTGCCAGGACCATGGCCTGACCCCGGACCAGGTGGT

GGCCATCGCCAGCCACGATGGCGGCAAGCAGGCGCTGGAGACGGTGCAGCGGCTGTTGCCGGTGCTGTGCCAGGACCATG

GCCTGACCCCGGACCAATTGGTGGCCATCGCCAACAATAACGGCGGCAAGCAGGCGCTGGAGACGGTGCAGCGGCTGTTG

CCGGTGCTGTGCCAGGACCATGGCCTGACCCCGGACCAGGTCGTGGCCATCGCCAGCAATGGCGGCAAGCAGGCGCTGGA

GACAGTGCAGCGGCTGTTGCCGGTGCTGTGCCAGGACCATGGTCTGACCCCGGACCAGGTCGTGGCCATCGCCAGCAATA

TTGGCGGCAAGCAGGCGCTGGAGACGGTGCAGCGGCTGTTGCCGGTGCTGTGCCAGGCCCATGGTCTGACCCCGGCGCAG

GTGGTGGCCATCGCCAGCCA

>27P1F_2

GGATGCGGGGGAGTTGAGAGGTCCGCCGTTACAGTTGGACACAGGCCAACTTCTCAAGATTGCAAAACGTGGCGGCGTGA

CCGCAGTGGAGGCAGTGCATGCATCGCGCAATGCACTGACGGGTGCCCCCCTGAACCTGACCCCGGCACAGGTGGTGGCC

ATCGCCAGCAATAACGGCGGCAAGCAGGCGCTGGAGACGGTGCAGCGGCTGTTGCCGTTGCTGTGCCAGGCCCATGGCCT

GACCCCGGCGCAGGTGGTGGCCATCGCCAGCCACGATGGCGGCAAGCAGGCACTGGAGACGGTGCAGCGGCTGTTGCCGG

TGCTGTGCCAGGACCATGGCCTGACCCCGGACCAGGTGGTGGCCATCGCCAGCAATAGTGGCGGCAAGCAGGCGCTGGAG

ACGGTGCAGCGGCTGTTGCCGGTGCTGTGCCAGGACCATGGCCTGACACCGGACCAGGTGGTGGCCATCGCCAGCAATGG

CGGCGGCAAGCAGGCGCTGGAGACGGTGCAGCGGCTGTTGCCGGTGCTGTGCCAGGACCATGGCCTGACCCCGGACCAGG

TGGTGGCCATCGCCAGCCACGATGGCGGCAAGCAGGCGCTGGAGACGGTGCAGCGGCTGTTGCCGGTGCTGTGCCAGGAC

CATGGCCTGACCCCGGACCAATTGGTGGCCATCGCCAACAATAACGGCGGCAAGCAGGCGCTGGAGACGGTGCAGCGGCT

GTTGCCGGTGCTGTGCCAGGACCATGGCCTGACCCCGGACCAGGTCGTGGCCATCGCCAGCAATGGCGGCAAGCAGGCGC

TGGAGACAGTGCAGCGGCTGTTGCCGGTGCTGTGCCAGGACCATGGTCTGACCCCGGACCAGGTCGTGGCCATCGCCAGC

AATATTGGCGGCAAGCAGGCGCTGGAGACGGTGCAGCGGCTGTTGCCGGTGCTGTGCCAGGCCCATGGTCTGACCCCGGC

GCAGGTGGTGGCCATCGCCAGC

>27P1R_1

GGGCAACAATGCTCTCCAGCGCCTGCTTGCCGCCGCCATTGCTGGCGATGGCCACCACCTGCGCCGGGGTCAGGCCATGG

TCCTGGCACAGCACCGGCAACAGCCGCTGCACCGTCTCCAGCGCCTGCTTGCCGCCATCGTGGCTGGCGATGGCCACCAC

CTGCGCCGGGGTCAGGCCATGGTCCTGGCACAGCACCGGCAACAGCCGCTGCACCGTCTCCAGCGCCTGCTTGCCGCCGT

TATTGCTGGCGATGGCCACCACCTGGTCCGGGGTCAGGCCATGGGCCTGGCACAGCACCGGCAACAGCCGCTGCACCGTC

TCCAGCGCCTGCTTGCCGCCGTTATTGCTGGCGATGGCCACCACCTGGTCCGGGGTCAGGCCATGGGCCTGGCACAGCAC

CGGCAACAGCCGCTGCACCGTCTCCAGCGCCTGCTTGCCGCCATCGTGGCTGGCGATGGCCACCACCTGGTCCGGGGTCA

GACCATGGGCCTGGCACAGCACCGGCAACAGCCGCTGCACCGTCTCCAGCGCCTGCTTGCCGCCGTTATTGCTGGCGATG

GCCACCACCTGCGCCGGGGTCAGGCCATGGGCCTGGCACAGCACCGGCAACAGCCGCTGCACCGTCTCCAGCGCCTGCTT

GCTGCCGTTATTGCTGGCGATGGCCACCACCTGCGCCGGGGTCAGGCCATGGGCCTGGCACAGCACTGGCAACAGCCGCT

GCACCGTCTCCAGCGCCTGCTTGCCGCCGTTATTGTTGGCGATGGCTACCACCTGGTCCGGGGTCAGACCATGGTCCTGG

CACAGCACCGGCAACAGCCGCTGCACCGTCTCCAGCGCCTGCTTGCCGCCGTTATTGCTGGCGATGGCCACCACCTGCGC

CGGGGTCAGGCCATGGGCCTGGCACAGCACCGGCAACAGCCGCTGCACCGTCTCCAGCGCCTGCTTACCGCCATCGTGGC

TGGCGATGGCCACC

>27P1R_2

GGCGAGATAACTGGGCAACAATGCTCTCCAGCGCCTGCTTGCCGCCGCCATTGCTGGCGATGGCCACCACCTGCGCCGGG

GTCAGGCCATGGTCCTGGCACAGCACCGGCAACAGCCGCTGCACCGTCTCCAGCGCCTGCTTGCCGCCATCGTGGCTGGC

GATGGCCACCACCTGCGCCGGGGTCAGGCCATGGTCCTGGCACAGCACCGGCAACAGCCGCTGCACCGTCTCCAGCGCCT

GCTTGCCGCCGTTATTGCTGGCGATGGCCACCACCTGGTCCGGGGTCAGGCCATGGGCCTGGCACAGCACCGGCAACAGC

CGCTGCACCGTCTCCAGCGCCTGCTTGCCGCCGTTATTGCTGGCGATGGCCACCACCTGGTCCGGGGTCAGGCCATGGGC

CTGGCACAGCACCGGCAACAGCCGCTGCACCGTCTCCAGCGCCTGCTTGCCGCCATCGTGGCTGGCGATGGCCACCACCT

GGTCCGGGGTCAGACCATGGGCCTGGCACAGCACCGGCAACAGCCGCTGCACCGTCTCCAGCGCCTGCTTGCCGCCGTTA

TTGCTGGCGATGGCCACCACCTGCGCCGGGGTCAGGCCATGGGCCTGGCACAGCACCGGCAACAGCCGCTGCACCGTCTC

CAGCGCCTGCTTGCTGCCGTTATTGCTGGCGATGGCCACCACCTGCGCCGGGGTCAGGCCATGGGCCTGGCACAGCACTG

GCAACAGCCGCTGCACCGTCTCCAGCGCCTGCTTGCCGCCGTTATTGTTGGCGATGGCTACCACCTGGTCCGGGGTCAGA

CCATGGTCCTGGCACAGCACCGGCAACAGCCGCTGCACCGTCTCCAGCGCCTGCTTGCCGCCGTTATTGCTGGCGATGGC

CACCACCTGCGCCGGGGTCAGGCCATGGGCCTGGCACAGCACCGGCAACAGCCGCTGCACCGTCTCCAGCGCCTGCTTAC

CGCCATCGTGGCTGGCGATGGC

>27P2F

CGCGCACATCGTTGCGCTCAGCAAACACCCGGCAGCGTTAGGGACCGTTGCTGTCACGTATCAGCACATAATCACGGCGT

TGCCAGAGGCGACACACGAAGACATCGTTGGCGTCGGCAAACAGTGGTCCGGCGCACGCGCCCTGGAGGCCTTGCTCACG

GATGCGGGGGAGTTGAGAGGTCCGCCGTTACAGTTGGACACAGGCCAACTTCTCAAGATTGCAAAACGTGGCGGCGTGAC

CGCAGTGGAGGCAGTGCATGCATCGCGCAATGCACTGACGGGTGCCCCCCTGAACCTGACCCCGGCACAGGTGGTGGCCA

TCGCCAGCAATAACGGCGGCAAGCAGGCGCTGGAGACGGTGCAGCGGCTGTTGCCGTTGCTGTGCCAGGCCCATGGCCTG

ACCCCGGCGCAGGTGGTGGCCATCGCCAGCCACGATGGCGGCAAGCAGGCACTGGAGACGGTGCAGCGGCTGTTGCCGGT

GCTGTGCCAGGACCATGGCCTGACCCCGGACCAGGTGGTGGCCATCGCCAGCAATAGTGGCGGCAAGCAGGCGCTGGAGA

CGGTGCAGCGGCTGTTGCCGGTGCTGTGCCAGGACCATGGCCTGACACCGGACCAGGTGGTGGCCATCGCCAGCAATGGC

GGCGGCAAGCAGGCGCTGGAGACGGTGCAGCGGCTGTTGCCGGTGCTGTGCCAGGACCATGGCCTGACCCCGGACCAGGT

GGTGGCCATCGCCAGCCACGATGGCGGCAAGCAGGCGCTGGAGACGGTGCAGCGGCTGTTGCCGGTGCTGTGCCAGGACC

ATGGCCTGACCCCGGACCAATTGGTGGCCATCGCCAACAATAACGGCGGCAAGCAGGCGCTGGAGACGGTGCAGCGGCTG

TTGCCGGTGCTGTGCCAGGACCATGGCCTGACCCCGGACCAGGTCGTGGCCATCGCCAGCAATGGCGGCAAGCAGGCGCT

GGAGACAGTGCAGCGGCT

>27P2R

ATTATGTGCTGATACGTGACAGCAACGGTCCCTAACGCTGCCGGGTGTTTGCTGAGCGCAACGATGTGCGCGTGTGTAAA

CCCATGGCCCACCAGTGCCTCGTGGTGCTGCGCCACTGTCGAACGCACCTTCGGTTTGATCTTCTCTTGCTGCTGCTGAC

TGTAGCCGAGCGTGCGTAGATCCACCTGCGCGGCCGGCGAAGCGTCGGAGGGTTGCGCCGCACGCCGTCGCGGGGCCGGC

TTGGCGCGCGGCGGCCGCGCGGCAGTGACAGCGACACGCACGGTGGGTGGCGGGTCATCGGCTGCACGCAGAGCCGATTG

CGCCTCATCCCACTCTGCTGGGGCAGCCGCTGTATGCGGCGTGCCGACGGCAGGCATCGAATCAAAAAGCGATGTATCAA

GAAGCGACGGATCGAACTGACGCAGCGGATCGCTGAAGCTGCCCGCCGAGAACGCAGGCGAGGGCGCAGGGGGAGATGGC

AGCCGGGTCCGGGACATCGTCCGCCGAGCGGGCAAGCCATCCAGGGGGCCGCCAGCAGGCGGAGCCCCCCCCCGATCTGC

AGTCGGCTGAACCCTATCCGGTTGGGGTCCGGGCAGAAGCTCGCGGGCAGGACTTGGCGTGCGCGAACGAATGGGATCC

>27P3F

AAAAAGGATTGCCGCACGCGCCGGAATTGATCAGAAGAGTCAATAGCCGTATTGGCGAACGCACGTCCCATCGCGTTGCC

GACTACGCGCAAGTGGTTCGCGTGCTGGAGTTTTTCCAGTGCCACTCCCACCCAGCGTACGCATTTGATGAGGCCATGAC

GCAGTTCGGGATGAGCAGGAACGGGTTGTTACAGCTCTTTCGCAGAGTGGGCGTCACCGAACTCGAAGCCCGCGGTGGAA

CGCTCCCCCCAGCCTCGCAGCGTTGGGACCGTATCCTCCAGGCATCAGGGATGAAAAGGGCCAAACCGTCCCCTACTTCA

GCTCAAACGCCGGATCAGGCGTCTTTGCATGCATTCGCCGATTCGCTGGAGCGTGACCTTGATGCGCCCAGCCCAATGCA

CGAGGGAGATCAGACGCGGGCAAGCAGCCGTAAACGGTCCCGATCGGATCGTGCTGTCACCGGCCCCTCCGCACAGCAAT

CTTTCGAGGTGCGCGTTCCCGAACAGCGCGATGCGCTGCATTTGCCCCTCAGCTGGAGGGTAAAACGCCCGCGTACCAGG

ATCGGGGGCGGCCTCCCGGATCC

>27P3R

CTTCGAGTTCGGTGACGCCCACTCTGCGAAAGAGCTGTAACAACCCGTTCCTGCTCATCCCGAACTGCGTCATGGCCTCA

TCAAATGCGTACGCTGGGTGGGAGTGGCACTGGAAAAACTCCAGCACGCGAACCACTTGCGCGTAGTCGGCAACGCGATG

GGACGTGCGTTCGCCAATACGGCTATTGACTCTTCTGATCAATTCCGGCGCGTGCGGCAATCCTTTTTTCACTGCATCCA

GGGCAGGACGTCCGCCGAGGCAGGCCAAGGCGACGAGGTGGTCGTTGGTCAACGCGGCCAACGCCGGATCAGGGCGAGAT

AACTGGGCAACAATGCTCTCCAGCGCCTGCTTGCCGCCGCCATTGCTGGCGATGGCCACCACCTGCGCCGGGGTCAGGCC

ATGGTCCTGGCACAGCACCGGCAACAGCCGCTGCACCGTCTCCAGCGCCTGCTTGCCGCCATCGTGGCTGGCGATGGCCA

CCACCTGCGCCGGGGTCAGGCCATGGTCCTGGCACAGCACCGGCAACAGCCGCTGCACCGTCTCCAGCGCCTGCTTGCCG

CCGTTATTGCTGGCGATGGCCACCACCTGGTCCGGGGTCAGGCCATGGGCCTGGCACAGCACCGGCAACAGCCGCTGCAC

CGTCTCCAGCGCCTGCTTGCCGCCGTTATTGCTGGCGATGGCCACCACCTGGTCCGGGGTCAGGCCATGGGCCTGGCACA

GCACCGGCAACAGCCGCTGCACCGTCTCCAGCGCCTGCTTGCCGCCATCGTGGCTGGCGATGGCCACCACCTGGTCCGGG

GTCAGACCATGGGCCTGGCACAGCACCGGCAACAGCCGCTGCACCGTCTCCAGCGCCTGCTTGCCGCCGTTATTGCTGGC

GATGGCCACCACCTGCGCCGGGGTCAGGCCATGGGCCTGGCACAGCACCGGCAACAGCCGCTGCACCGTCTCCAGCGCCT

GCTTGCTGCCGTTATTGCTGGC

>M13F

GGATCCCATTCGTTCGCGCACGCCAAGTCCTGCCCGCGAGCTTCTGCCCGGACCCCAACCGGATAGGGTTCAGCCGACTG

CAGATCGGGGGGGGGCTCCGCCTGCTGGCGGCCCCCTGGATGGCTTGCCCGCTCGGCGGACGATGTCCCGGACCCGGCTG

CCATCTCCCCCTGCGCCCTCGCCTGCGTTCTCGGCGGGCAGCTTCAGCGATCCGCTGCGTCAGTTCGATCCGTCGCTTCT

TGATACATCGCTTTTTGATTCGATGCCTGCCGTCGGCACGCCGCATACAGCGGCTGCCCCAGCAGAGTGGGATGAGGCGC

AATCGGCTCTGCGTGCAGCCGATGACCCGCCACCCACCGTGCGTGTCGCTGTCACTGCCGCGCGGCCGCCGCGCGCCAAG

CCGGCCCCGCGACGGCGTGCGGCGCAACCCTCCGACGCTTCGCCGGCCGCGCAGGTGGATCTACGCACGCTCGGCTACAG

TCAGCAGCAGCAAGAGAAGATCAAACCGAAGGTGCGTTCGACAGTGGCGCAGCACCACGAGGCACTGGTGGGCCATGGGT

TTACACACGCGCACATCGTTGCGCTCAGCAAACACCCGGCAGCGTTAGGGACCGTTGCTGTCACGTATCAGCACATAATC

ACGGCGTTGCCAGAGGCGACACACGAAGACATCGTTGGCGTCGGCAAACAGTGGTCCGGCGCACGCGCCCTGGAGGCCTT

GCTCACGGATGCGGGGGAGTTGAGAGGTCCGCCGTTACAGTTGGACACAGGCCAACTTCTCAAGATTGCAAAACGTGGCG

GCGTGACCGCAGTGGAGGCAGTGCATGCATCGCGCAATGCACTGACGGGTGCCCCCCTGAACCTGACCCCGGCACAGGTG

GTGGCCATCGCCAGCAATAACGGCGGCAAGCAGGCGCTGGAGACGGTGCAGCGGCTGTTGCCGTTGC

>M13R

GGATCCGGGAGGCCGCCCCCGATCCTGGTACGCGGGCGTTTTACCCTCCAGCTGAGGGGCAAATGCAGCGCATCGCGCTG

TTCGGGAACGCGCACCTCGAAAGATTGCTGTGCGGAGGGGCCGGTGACAGCACGATCCGATCGGGACCGTTTACGGCTGC

TTGCCCGCGTCTGATCTCCCTCGTGCATTGGGCTGGGCGCATCAAGGTCACGCTCCAGCGAATCGGCGAATGCATGCAAA

GACGCCTGATCCGGCGTTTGAGCTGAAGTAGGGGACGGTTTGGCCCTTTTCATCCCTGATGCCTGGAGGATACGGTCCCA

ACGCTGCGAGGCTGGGGGGAGCGTTCCACCGCGGGCTTCGAGTTCGGTGACGCCCACTCTGCGAAAGAGCTGTAACAACC

CGTTCCTGCTCATCCCGAACTGCGTCATGGCCTCATCAAATGCGTACGCTGGGTGGGAGTGGCACTGGAAAAACTCCAGC

ACGCGAACCACTTGCGCGTAGTCGGCAACGCGATGGGACGTGCGTTCGCCAATACGGCTATTGACTCTTCTGATCAATTC

CGGCGCGTGCGGCAATCCTTTTTTCACTGCATCCAGGGCAGGACGTCCGCCGAGGCAGGCCAAGGCGACGAGGTGGTCGT

TGGTCAACGCGGCCAACGCCGGATCAGGGCGAGATAACTGGGCAACAATGCTCTCCAGCGCCTGCTTGCCGCCGCCATTG

CTGGCGATGGCCACCACCTGCGCCGGGGTCAGGCCATGGTCCTGGCACAGCACCGGCAACAGCCGCTGCACCGTCTCCAG

CGCCTGCTTGCCGCCATCGTGGCTGGCGATGGCCACCACCTGCGCCGGGGTCAGGCCATGGTCCTGGCACAGCACCGGCA

ACAGCCGCTGCACCGTCTCCAGCGCCTGCTTGCCGCCGTTATTGCTGGCGATGGCCACCACCTGGTCCG

**pTAL*_Bam_*_HI_-31**

> pCC2FOS-MscI-1

GGCGGCAAGCAGGCGCTGGAGACGGTGCAGCGGCTGTTGCCGGTGCTGTGCCAGGACCATGGCCTGACCCCGGACCAGGT

GGTGGCCATCGCCAGCAATGGCGGCGGCAAGCAGGCGCTGGAGACGGTGCAGCGGCTGTTGCCGGTGCTGTGCCAGGACC

ATGGCCTGACCCCGGACCAGGTCGTGGCCATCGCCAGCAGTAGCGGCGGCAAGCAGGCGCTGGAGACGGTGCAGCGGCTG

TTGCCGGTGCTGTGCCAGGCCCATGGCCTGACCCCGGACCAGGTGGTGGCCATCGCCAGCCACGATGGCGGCAAGCAGGC

GCTGGAGACGGTGCAGCGGCTGTTGCCGGTGCTGTGCCAGGACCATGGCCTGACCCCGGACCAGGTGGTGGCCATCGCCA

GCAATATTGGCGGCAAGCAGGCGCTGGAGACGGTGCAGCGGCTGTTGCCGGTGCTGTGCCAGGACCATGGCCTGACCCCG

GACCAGGTGGTGGCCATCGCCAGCAATATTGGCGGCAAGCAGGCGCTGGAGACGGTGCAGCGGCTGTTGCCGGTGCTGTG

CCAGGACCATGGCCTGACCCCGGACCAGGTGGTGGCCATCGCCAACAATAACGGCGGCAAGCAGGCGCTGGAGACGGTGC

AGCGGCTGTTGCCGGTGCTGTGCCAGGACCATGGCCTGACCCCGGACCAGGTGGTGGCCATCGCCAGCAATATTGGCGGC

AAGCAGGCGCTGGAGACGGTGCAGCGGCTGTTGCCGGTGCTGTGCCAGGACCATGGCCTGACCCCGGCCCAGGTGGTGGC

CATCGCCAACAATAACGGCGGCAAGCAGGCGCTGGAGACGGTGCAGCGGCTGTTGCCGGTGCTGTGCCAGGACCATGGCC

TGACCCCGGACCAGGTGGTGGCCATCGCCAGCAATATTGGCGGCAAGCAGGCGCTGGAGACGGTGCAGCGGCTGTTGCCG

GTGCTGTGCCAGGACCA

> pCC2FOS-MscI-2

GGCGGCAAGCAGGCGCTGGAGACGGTGCAGCGGCTGTTGCCGGTGCTGTGCCAGGACCATGGCCTGACCCCGGACCAGGT

GGTGGCCATCGCCAGCAATGGCGGCGGCAAGCAGGCGCTGGAGACGGTGCAGCGGCTGTTGCCGGTGCTGTGCCAGGACC

ATGGCCTGACCCCGGACCAGGTCGTGGCCATCGCCAGCAGTAGCGGCGGCAAGCAGGCGCTGGAGACGGTGCAGCGGCTG

TTGCCGGTGCTGTGCCAGGCCCATGGCCTGACCCCGGACCAGGTGGTGGCCATCGCCAGCCACGATGGCGGCAAGCAGGC

GCTGGAGACGGTGCAGCGGCTGTTGCCGGTGCTGTGCCAGGACCATGGCCTGACCCCGGACCAGGTGGTGGCCATCGCCA

GCAATATTGGCGGCAAGCAGGCGCTGGAGACGGTGCAGCGGCTGTTGCCGGTGCTGTGCCAGGACCATGGCCTGACCCCG

GACCAGGTGGTGGCCATCGCCAGCAATATTGGCGGCAAGCAGGCGCTGGAGACGGTGCAGCGGCTGTTGCCGGTGCTGTG

CCAGGACCATGGCCTGACCCCGGACCAGGTGGTGGCCATCGCCAACAATAACGGCGGCAAGCAGGCGCTGGAGACGGTGC

AGCGGCTGTTGCCGGTGCTGTGCCAGGACCATGGCCTGACCCCGGACCAGGTGGTGGCCATCGCCAGCAATATTGGCGGC

AAGCAGGCGCTGGAGACGGTGCAGCGGCTGTTGCCGGTGCTGTGCCAGGACCATGGCCTGACCCCGGCCCAGGTGGTGGC

CATCGCCAACAATAACGGCGGCAAGCAGGCGCTGGAGACGGTGCAGCGGCTGTTGCCGGTGCTGTGCCAGGACCATGGCC

TGACCCCGGACCAGGTGGTGGCCATCGCCAGCAATATTGGCGGCAAGCAGGCGCTGGAGACGGTGCAGCGGCTGTTGCCG

GTGCTGTGCCAGGACCA

> pCC2FOS-MscI-3

TTGGCGGCAAGCAGGCGCTGGAGACGGTGCAGCGGCTGTTGCCGGTGCTGTGCCAGGACCATGGCCTGACCCCGGACCAG

GTGGTGGCCATCGCCAGCCATGGCGGCGGCAAGCAGGCGCTGGAGACGGTGCAGCGGCTGTTGCCGGTGCTGTGCCAGGA

CCATGGCCTGACCCCGGACCAGGTGGTGGCCATCGCCAGCAATATTGGCGGCAAGCAGGCGCTGGAGACGGTGCAGCGGC

TGTTGCCGGTGCTGTGCCAGGACCATGGCCTGATCCCGGACCAGGTGGTGGCCATCGCCAGCAATATTGGCGGCAAGCAG

GCGCTGGAGACGGTGCAGCGGCTGTTGCCGGTGCTGTGCCAGGACCATGGCCTGACCCCGGACCAGGTGGTGGCCATCGC

CAGCAATAGTGGCGGCAAGCAGGCGCTGGAGACGGTGCAGCGGCTGTTGCCGGTGCTGTGCCAGGCCCATGGCCTGACCC

CAGACCAAGTGGTGGCCATCGCCAGCCACGATGGCGGCAAGCAGGCGCTGGAGACGGTGCAGCGGCTGTTGCCGGTGCTG

TGCCAGGACCATGGCCTGACCCCGGCCCAAGTGGTGGCCATCGCCAGCAATAACGGCGGCAAGCAGGCGCTGGAGACGGT

GCAGCGGCTGTTGCCGGTGCTGTGCCAGGCCCATGGCCTGACCCCAGACCAAGTGGTGGCCATCGCCAGCCACGATGGCG

GCAAGCAGGCGCTGGAGACGGTGCAGCGGCTGTTGCCGGTGCTGTGCCAGGACCATGGCCTGACCCCGGCCCAAGTGGTG

GCCATCGCCAGCAATAGTGGCGGCAAGCAGGCGCTGGAGACGGTGCAGCGGCTGTTGCCGGTGCTGTGCCAGGACCATGG

CCTGACCCCGGACCAGGTGGTGGCCATCGCCAGCAATGGCGGCGGCAAGCAGGCGCTGGAGACGGTGCAGCGGCTGTTGC

CGGTGCTGTGCCAGGACCA

> pCC2FOS-MscI-4

GTGGCGGCAAGCAGGCGCTGGAGACGGTGCAGCGGCTGTTGCCGGTGCTGTGCCAGGACCATGGCCTGACCCCGGACCAG

GTGGTGGCCATCGCCAGCAATGGCGGCGGCAAGCAGGCGCTGGAGACGGTGCAGCGGCTGTTGCCGGTGCTGTGCCAGGA

CCATGGCCTGACCCCGGACCAGGTCGTGGCCATCGCCAGCAGTAGCGGCGGCAAGCAGGCGCTGGAGACGGTGCAGCGGC

TGTTGCCGGTGCTGTGCCAGGCCCATGGCCTGACCCCGGACCAGGTGGTGGCCATCGCCAGCCACGATGGCGGCAAGCAG

GCGCTGGAGACGGTGCAGCGGCTGTTGCCGGTGCTGTGCCAGGACCATGGCCTGACCCCGGACCAGGTGGTGGCCATCGC

CAGCAATATTGGCGGCAAGCAGGCGCTGGAGACGGTGCAGCGGCTGTTGCCGGTGCTGTGCCAGGACCATGGCCTGACCC

CGGACCAGGTGGTGGCCATCGCCAGCAATATTGGCGGCAAGCAGGCGCTGGAGACGGTGCAGCGGCTGTTGCCGGTGCTG

TGCCAGGACCATGGCCTGACCCCGGACCAGGTGGTGGCCATCGCCAACAATAACGGCGGCAAGCAGGCGCTGGAGACGGT

GCAGCGGCTGTTGCCGGTGCTGTGCCAGGACCATGGCCTGACCCCGGACCAGGTGGTGGCCATCGCCAGCAATATTGGCG

GCAAGCAGGCGCTGGAGACGGTGCAGCGGCTGTTGCCGGTGCTGTGCCAGGACCATGGCCTGACCCCGGCCCAGGTGGTG

GCCATCGCCAACAATAACGGCGGCAAGCAGGCGCTGGAGACGGTGCAGCGGCTGTTGCCGGTGCTGTGCCAGGACCATGG

CCTGACCCCGGACCAGGTGGTGGCCATCGCCAGCAATATTGGCGGCAAGCAGGCGCTGGAGACGGTGCAGCGGCTGTTGC

CGGTGCTGTGCCAGGACCA

> pCC2FOS-MscI-5

TGGCGGCAAGCAGGCGCTGGAGACGGTGCAGCGGCTGTTGCCGGTGCTGTGCCAGGACCATGGCCTGACCCCGGACCAGG

TGGTGGCCATCGCCAGCAATAGTGGCGGCAAGCAGGCGCTGGAGACGGTGCAGCGGCTGTTGCCGGTGCTGTGCCAGGCC

CATGGCCTGACCCCAGACCAAGTGGTGGCCATCGCCAGCCACGATGGCGGCAAGCAGGCGCTGGAGACGGTGCAGCGGCT

GTTGCCGGTGCTGTGCCAGGACCATGGCCTGACCCCGGCCCAAGTGGTGGCCATCGCCAGCAATAACGGCGGCAAGCAGG

CGCTGGAGACGGTGCAGCGGCTGTTGCCGGTGCTGTGCCAGGCCCATGGCCTGACCCCAGACCAAGTGGTGGCCATCGCC

AGCCACGATGGCGGCAAGCAGGCGCTGGAGACGGTGCAGCGGCTGTTGCCGGTGCTGTGCCAGGACCATGGCCTGACCCC

GGCCCAAGTGGTGGCCATCGCCAGCAATAGTGGCGGCAAGCAGGCGCTGGAGACGGTGCAGCGGCTGTTGCCGGTGCTGT

GCCAGGACCATGGCCTGACCCCGGACCAGGTGGTGGCCATCGCCAGCAATGGCGGCGGCAAGCAGGCGCTGGAGACGGTG

CAGCGGCTGTTGCCGGTGCTGTGCCAGGACCATGGCCTGACCCCGGACCAGGTCGTGGCCATCGCCAGCAGTAGCGGCGG

CAAGCAGGCGCTGGAGACGGTGCAGCGGCTGTTGCCGGTGCTGTGCCAGGCCCATGGCCTGACCCCGGACCAGGTGGTGG

CCATCGCCAGCCACGATGGCGGCAAGCAGGCGCTGGAGACGGTGCAGCGGCTGTTGCCGGTGCTGTGCCAGGACCATGGC

CTGACCCCGGACCAGGTGGTGGCCATCGCCAGCAATATTGGCGGCAAGCAGGCGCTGGAGACGGTGCAGCGGCTGTTGCC

GGTGCTGTGCCAGGACCA

> pCC2FOS-MscI-6

GATGGCGGCAAGCAGGCGCTGGAGACGGTGCAGCGGCTGTTGCCGGTGCTGTGCCAGGACCATGGCCTGACCCCGGCCCA

AGTGGTGGCCATCGCCAGCAATAACGGCGGCAAGCAGGCGCTGGAGACGGTGCAGCGGCTGTTGCCGGTGCTGTGCCAGG

CCCATGGCCTGACCCCAGACCAAGTGGTGGCCATCGCCAGCCACGATGGCGGCAAGCAGGCGCTGGAGACGGTGCAGCGG

CTGTTGCCGGTGCTGTGCCAGGACCATGGCCTGACCCCGGCCCAAGTGGTGGCCATCGCCAGCAATAGTGGCGGCAAGCA

GGCGCTGGAGACGGTGCAGCGGCTGTTGCCGGTGCTGTGCCAGGACCATGGCCTGACCCCGGACCAGGTGGTGGCCATCG

CCAGCAATGGCGGCGGCAAGCAGGCGCTGGAGACGGTGCAGCGGCTGTTGCCGGTGCTGTGCCAGGACCATGGCCTGACC

CCGGACCAGGTCGTGGCCATCGCCAGCAGTAGCGGCGGCAAGCAGGCGCTGGAGACGGTGCAGCGGCTGTTGCCGGTGCT

GTGCCAGGCCCATGGCCTGACCCCGGACCAGGTGGTGGCCATCGCCAGCCACGATGGCGGCAAGCAGGCGCTGGAGACGG

TGCAGCGGCTGTTGCCGGTGCTGTGCCAGGACCATGGCCTGACCCCGGACCAGGTGGTGGCCATCGCCAGCAATATTGGC

GGCAAGCAGGCGCTGGAGACGGTGCAGCGGCTGTTGCCGGTGCTGTGCCAGGACCATGGCCTGACCCCGGACCAGGTGGT

GGCCATCGCCAGCAATATTGGCGGCAAGCAGGCGCTGGAGACGGTGCAGCGGCTGTTGCCGGTGCTGTGCCAGGACCATG

GCCTGACCCCGGACCAGGTGGTGGCCATCGCCAACAATAACGGCGGCAAGCAGGCGCTGGAGACGGTGCAGCGGCTGTTG

CCGGTGCTGTGCCAGG

> pCC2FOS-MscI-7

CCATCGCCAGCAATAGTGGCGGCAAGCAGGCGCTGGAGACGGTGCAGCGGCTGTTGCCGGTGCTGTGCCAGGACCATGGC

CTGACCCCGGACCAGGTGGTGGCCATCGCCAGCAATGGCGGCGGCAAGCAGGCGCTGGAGACGGTGCAGCGGCTGTTGCC

GGTGCTGTGCCAGGACCATGGCCTGACCCCGGACCAGGTCGTGGCCATCGCCAGCAGTAGCGGCGGCAAGCAGGCGCTGG

AGACGGTGCAGCGGCTGTTGCCGGTGCTGTGCCAGGCCCATGGCCTGACCCCGGACCAGGTGGTGGCCATCGCCAGCCAC

GATGGCGGCAAGCAGGCGCTGGAGACGGTGCAGCGGCTGTTGCCGGTGCTGTGCCAGGACCATGGCCTGACCCCGGACCA

GGTGGTGGCCATCGCCAGCAATATTGGCGGCAAGCAGGCGCTGGAGACGGTGCAGCGGCTGTTGCCGGTGCTGTGCCAGG

ACCATGGCCTGACCCCGGACCAGGTGGTGGCCATCGCCAGCAATATTGGCGGCAAGCAGGCGCTGGAGACGGTGCAGCGG

CTGTTGCCGGTGCTGTGCCAGGACCATGGCCTGACCCCGGACCAGGTGGTGGCCATCGCCAACAATAACGGCGGCAAGCA

GGCGCTGGAGACGGTGCAGCGGCTGTTGCCGGTGCTGTGCCAGGACCATGGCCTGACCCCGGACCAGGTGGTGGCCATCG

CCAGCAATATTGGCGGCAAGCAGGCGCTGGAGACGGTGCAGCGGCTGTTGCCGGTGCTGTGCCAGGACCATGGCCTGACC

CCGGCCCAGGTGGTGGCCATCGCCAACAATAACGGCGGCAAGCAGGCGCTGGAGACGGTGCAGCGGCTGTTGCCGGTGCT

GTGCCAGGA

> pCC2FOS-MscI-8

ATTGGCGGCAAGCAGGCGCTGGAGACGGTACAGCGGCTGTTGCCGGTGCTGTGCCAGGCCCATGGCCTGACCCCGGACCA

GGTGGTGGCCATCGCCAGCAATATTGGCGGCAAGCAGGCGCTGGAGACGGTGCAGCGGCTGTTGCCGGTGCTGTGCCAGG

ACCATGGCCTGACCCCGGACCAGGTGGTGGCCATCGCCAGCCATGGCGGCGGCAAGCAGGCGCTGGAGACGGTGCAGCGG

CTGTTGCCGGTGCTGTGCCAGGACCATGGCCTGACCCCGGACCAGGTGGTGGCCATCGCCAGCAATATTGGCGGCAAGCA

GGCGCTGGAGACGGTGCAGCGGCTGTTGCCGGTGCTGTGCCAGGACCATGGCCTGATCCCGGACCAGGTGGTGGCCATCG

CCAGCAATATTGGCGGCAAGCAGGCGCTGGAGACGGTGCAGCGGCTGTTGCCGGTGCTGTGCCAGGACCATGGCCTGACC

CCGGACCAGGTGGTGGCCATCGCCAGCAATAGTGGCGGCAAGCAGGCGCTGGAGACGGTGCAGCGGCTGTTGCCGGTGCT

GTGCCAGGCCCATGGCCTGACCCCAGACCAAGTGGTGGCCATCGCCAGCCACGATGGCGGCAAGCAGGCGCTGGAGACGG

TGCAGCGGCTGTTGCCGGTGCTGTGCCAGGACCATGGCCTGACCCCGGCCCAAGTGGTGGCCATCGCCAGCAATAACGGC

GGCAAGCAGGCGCTGGAGACGGTGCAGCGGCTGTTGCCGGTGCTGTGCCAGGCCCATGGCCTGACCCCAGACCAAGTGGT

GGCCATCGCCAGCCACGATGGCGGCAAGCAGGCGCTGGAGACGGTGCAGCGGCTGTTGCCGGTGCTGTGCCAGGACCATG

GCCTGACCCCGGCCCAAGTGGTGGCCATCGCCAGCAATAGTGGCGGCAAGCAGGCGCTGGAGACGGTGCAGCGGCTGTTG

CCGGTGCTGTGCCAGGACCA

> pCC2FOS-MscI-9

GTGGCGGCAAGCAGGCGCTGGAGACGGTGCAGCGGCTGTTGCCGGTGCTGTGCCAGGACCATGGCCTGACCCCGGACCAG

GTGGTGGCCATCGCCAGCAATGGCGGCGGCAAGCAGGCGCTGGAGACGGTGCAGCGGCTGTTGCCGGTGCTGTGCCAGGA

CCATGGCCTGACCCCGGACCAGGTCGTGGCCATCGCCAGCAGTAGCGGCGGCAAGCAGGCGCTGGAGACGGTGCAGCGGC

TGTTGCCGGTGCTGTGCCAGGCCCATGGCCTGACCCCGGACCAGGTGGTGGCCATCGCCAGCCACGATGGCGGCAAGCAG

GCGCTGGAGACGGTGCAGCGGCTGTTGCCGGTGCTGTGCCAGGACCATGGCCTGACCCCGGACCAGGTGGTGGCCATCGC

CAGCAATATTGGCGGCAAGCAGGCGCTGGAGACGGTGCAGCGGCTGTTGCCGGTGCTGTGCCAGGACCATGGCCTGACCC

CGGACCAGGTGGTGGCCATCGCCAGCAATATTGGCGGCAAGCAGGCGCTGGAGACGGTGCAGCGGCTGTTGCCGGTGCTG

TGCCAGGACCATGGCCTGACCCCGGACCAGGTGGTGGCCATCGCCAACAATAACGGCGGCAAGCAGGCGCTGGAGACGGT

GCAGCGGCTGTTGCCGGTGCTGTGCCAGGACCATGGCCTGACCCCGGACCAGGTGGTGGCCATCGCCAGCAATATTGGCG

GCAAGCAGGCGCTGGAGACGGTGCAGCGGCTGTTGCCGGTGCTGTGCCAGGACCATGGCCTGACCCCGGCCCAGGTGGTG

GCCATCGCCAACAATAACGGCGGCAAGCAGGCGCTGGAGACGGTGCAGCGGCTGTTGCCGGTGCTGTGCCAGGACCATGG

CCTGACCCCGGACCAGGTGGTGGCCATCGCCAGCAATATTGGCGGCAAGCAGGCGCTGGAGACGGTGCAGCGGCTGTTGC

CGGTGCTGTGCCAGGACCA

>31P1F_1

CGAGGGAGTTGAGAGGTCCGCCGTTACAGTTGGACACAGGCCAACTTCTCAAGATTGCAAAACGTGGAGGCGTGACCTCA

GTGCAGGCAGTGCATGCATGGCGCAATGCACTGACGGGTGCCCCCCTGAACCTGACCCCGGACCAAGTGGTGGCCATCGC

CAGCAATATTGGCGGCAAGCAGGCGCTGGAGACGGTACAGCGGCTGTTGCCGGTGCTGTGCCAGGCCCATGGCCTGACCC

CGGACCAGGTGGTGGCCATCGCCAGCAATATTGGCGGCAAGCAGGCGCTGGAGACGGTGCAGCGGCTGTTGCCGGTGCTG

TGCCAGGACCATGGCCTGACCCCGGACCAGGTGGTGGCCATCGCCAGCCATGGCGGCGGCAAGCAGGCGCTGGAGACGGT

GCAGCGGCTGTTGCCGGTGCTGTGCCAGGACCATGGCCTGACCCCGGACCAGGTGGTGGCCATCGCCAGCAATATTGGCG

GCAAGCAGGCGCTGGAGACGGTGCAGCGGCTGTTGCCGGTGCTGTGCCAGGACCATGGCCTGATCCCGGACCAGGTGGTG

GCCATCGCCAGCAATATTGGCGGCAAGCAGGCGCTGGAGACGGTGCAGCGGCTGTTGCCGGTGCTGTGCCAGGACCATGG

CCTGACCCCGGACCAGGTGGTGGCCATCGCCAGCAATAGTGGCGGCAAGCAGGCGCTGGAGACGGTGCAGCGGCTGTTGC

CGGTGCTGTGCCAGGCCCATGGCCTGACCCCAGACCAAGTGGTGGCCATCGCCAGCCACGATGGCGGCAAGCAGGCGCTG

GAGACGGTGCAGCGGCTGTTGCCGGTGCTGTGCCAGGACCATGGCCTGACCCCGG

>31P1F_2

GGGAGTTGAGAGGTCCGCCGTTACAGTTGGACACAGGCCAACTTCTCAAGATTGCAAAACGTGGAGGCGTGACCTCAGTG

CAGGCAGTGCATGCATGGCGCAATGCACTGACGGGTGCCCCCCTGAACCTGACCCCGGACCAAGTGGTGGCCATCGCCAG

CAATATTGGCGGCAAGCAGGCGCTGGAGACGGTACAGCGGCTGTTGCCGGTGCTGTGCCAGGCCCATGGCCTGACCCCGG

ACCAGGTGGTGGCCATCGCCAGCAATATTGGCGGCAAGCAGGCGCTGGAGACGGTGCAGCGGCTGTTGCCGGTGCTGTGC

CAGGACCATGGCCTGACCCCGGACCAGGTGGTGGCCATCGCCAGCCATGGCGGCGGCAAGCAGGCGCTGGAGACGGTGCA

GCGGCTGTTGCCGGTGCTGTGCCAGGACCATGGCCTGACCCCGGACCAGGTGGTGGCCATCGCCAGCAATATTGGCGGCA

AGCAGGCGCTGGAGACGGTGCAGCGGCTGTTGCCGGTGCTGTGCCAGGACCATGGCCTGATCCCGGACCAGGTGGTGGCC

ATCGCCAGCAATATTGGCGGCAAGCAGGCGCTGGAGACGGTGCAGCGGCTGTTGCCGGTGCTGTGCCAGGACCATGGCCT

GACCCCGGACCAGGTGGTGGCCATCGCCAGCAATAGTGGCGGCAAGCAGGCGCTGGAGACGGTGCAGCGGCTGTTGCCGG

TGCTGTGCCAGGCCCATGGCCTGACCCCAGACCAAGTGGTGGCCATCGCCAGCCACGATGGCGGCAAGCAGGCGCTGGAG

ACGGTGCAGCGGCTGTTGCCGGTGCTGTGCCAGGACCATGGCCTGACCCCGGCCCAAGTGGTGGCCATCGCCAGCAATAA

CGGCGGCAAGCAGGCGCTGGAGACGGTGCAGCGGCTGTTGCCGGTGCTGTGCCAGGCCCATGGCCTGACCCCAGACCAAG

TGGTGGCCATCGC

>31P1R_1

TGCTCTCCAGCGCCTGCTTGCCGCCGCCATTGCTGGCGATGGCCACCACCTGGTCCGGGGTCAGGCCATGGTCCTGGCAC

AGCACCGGCAACAGCCGCTGCACCGTCTCCAGCGCCTGCTTGCCGCCAATATTGCTGGCGATGGCCACCACCTGGTCCGG

GGTCAGGCCATGGTCCTGGCACAGCACCGGCAACAGCCGCTGCACCGTCTCCAGCGCCTGCTTGCCGCCGTTATTGTTGG

CGATGGCCACCACCTGGGCCGGGGTCAGGCCATGGTCCTGGCACAGCACCGGCAACAGCCGCTGCACCGTCTCCAGCGCC

TGCTTGCCGCCAATATTGCTGGCGATGGCCACCACCTGGTCCGGGGTCAGGCCATGGTCCTGGCACAGCACCGGCAACAG

CCGCTGCACCGTCTCCAGCGCCTGCTTGCCGCCGTTATTGTTGGCGATGGCCACCACCTGGTCCGGGGTCAGGCCATGGT

CCTGGCACAGCACCGGCAACAGCCGCTGCACCGTCTCCAGCGCCTGCTTGCCGCCAATATTGCTGGCGATGGCCACCACC

TGGTCCGGGGTCAGGCCATGGTCCTGGCACAGCACCGGCAACAGCCGCTGCACCGTCTCCAGCGCCTGCTTGCCGCCAAT

ATTGCTGGCGATGGCCACCACCTGGTCCGGGGTCAGGCCATGGTCCTGGCACAGCACCGGCAACAGCCGCTGCACCGTCT

CCAGCGCCTGCTTGCCGCCATCGTGGCTGGCGATGGCCACCACCTGGTCCGGGGTCAGGCCATGGGCCTGGCACAGCACC

GGCAACAGCCGCTGCACCGTCTCCAGCGCCTGCTTGCCGCCGCTACTGCTGGCGATGGCCACGACCTGGTCCGGGGTCAG

GCCATGGTCCTGGCACAGCACCGGCAACAGCCGCTGCACCGT

>31P1R_2

GGCGGCAAGCAGGCGCTGGAGACGGTGCAGCGGCTGTTGCCGGTGCTGTGCCAGGACCATGGCCTGACCCCGGACCAGGT

CGTGGCCATCGCCAGCAGTAGCGGCGGCAAGCAGGCGCTGGAGACGGTGCAGCGGCTGTTGCCGGTGCTGTGCCAGGCCC

ATGGCCTGACCCCGGACCAGGTGGTGGCCATCGCCAGCCACGATGGCGGCAAGCAGGCGCTGGAGACGGTGCAGCGGCTG

TTGCCGGTGCTGTGCCAGGACCATGGCCTGACCCCGGACCAGGTGGTGGCCATCGCCAGCAATATTGGCGGCAAGCAGGC

GCTGGAGACGGTGCAGCGGCTGTTGCCGGTGCTGTGCCAGGACCATGGCCTGACCCCGGACCAGGTGGTGGCCATCGCCA

GCAATATTGGCGGCAAGCAGGCGCTGGAGACGGTGCAGCGGCTGTTGCCGGTGCTGTGCCAGGACCATGGCCTGACCCCG

GACCAGGTGGTGGCCATCGCCAACAATAACGGCGGCAAGCAGGCGCTGGAGACGGTGCAGCGGCTGTTGCCGGTGCTGTG

CCAGGACCATGGCCTGACCCCGGACCAGGTGGTGGCCATCGCCAGCAATATTGGCGGCAAGCAGGCGCTGGAGACGGTGC

AGCGGCTGTTGCCGGTGCTGTGCCAGGACCATGGCCTGACCCCGGCCCAGGTGGTGGCCATCGCCAACAATAACGGCGGC

AAGCAGGCGCTGGAGACGGTGCAGCGGCTGTTGCCGGTGCTGTGCCAGGACCATGGCCTGACCCCGGACCAGGTGGTGGC

CATCGCCAGCAATATTGGCGGCAAGCAGGCGCTGGAGACGGTGCAGCGGCTGTTGCCGGTGCTGTGCCAGGACCATGGCC

TGACCCCGGACCAGGTGGTGGCCATCGCCAGCAATGGCGGCGGCAAGCAGGCGCTGGAGAGCATTGTTGCCCAGTTA

>31P2F

CGCGCACATCGTTGCGCTCAGCCAACACCCGGCAGCGTTAGGGACCGTTGCTGTCACGTATCAGGACATAATCAGGGCGT

TGCCAGAGGCGACACACGAAGACATCGTTGGCGTCGGCAAACAGTGGTCCGGCGCACGCGCCCTGGAGGCCTTGCTCACG

GAGGCGAGGGAGTTGAGAGGTCCGCCGTTACAGTTGGACACAGGCCAACTTCTCAAGATTGCAAAACGTGGAGGCGTGAC

CTCAGTGCAGGCAGTGCATGCATGGCGCAATGCACTGACGGGTGCCCCCCTGAACCTGACCCCGGACCAAGTGGTGGCCA

TCGCCAGCAATATTGGCGGCAAGCAGGCGCTGGAGACGGTACAGCGGCTGTTGCCGGTGCTGTGCCAGGCCCATGGCCTG

ACCCCGGACCAGGTGGTGGCCATCGCCAGCAATATTGGCGGCAAGCAGGCGCTGGAGACGGTGCAGCGGCTGTTGCCGGT

GCTGTGCCAGGACCATGGCCTGACCCCGGACCAGGTGGTGGCCATCGCCAGCCATGGCGGCGGCAAGCAGGCGCTGGAGA

CGGTGCAGCGGCTGTTGCCGGTGCTGTGCCAGGACCATGGCCTGACCCCGGACCAGGTGGTGGCCATCGCCAGCAATATT

GGCGGCAAGCAGGCGCTGGAGACGGTGCAGCGGCTGTTGCCGGTGCTGTGCCAGGACCATGGCCTGATCCCGGACCAGGT

GGTGGCCATCGCCAGCAATATTGGCGGCAAGCAGGCGCTGGAGACGGTGCAGCGGCTGTTGCCGGTGCTGTGCCAGGACC

ATGGCCTGACCCCGGACCAGGTGGTGGCCATCGCCAGCAATAGTGGCGGCAAGCAGGCGCTGGAGACGGTGCAGCGGCTG

TTGCCGGTGCTGTGCCAGGCCCATGGCCTGACCCCAGACCAAGTGGTGGCCATCGCCAGCCACGATGGCGGCAAGCAGGC

GCTGGAGACGGTGCAGCGGC

>31P2R

TGATTATGTCCTGATACGTGACAGCAACGGTCCCTAACGCTGCCGGGTGTTGGCTGAGCGCAACGATGTGCGCGTGTGTA

AACCCATGACCCACCAGTGCCTCGTGGTGCCGCGCCACTGTCGAACGCACCTTCGGTTTGATCTTCTCTTGCTGCTGCTG

ACTGTAGCCGAGCGTGCGTAGATCCACCTGCGCGGCCGGCGAAGCGTCGGAGGGTTGCGCCGCACGCCGTCGCGGGGCCG

GCTTGGCGCGCGGCGGCCGCGCGGCAGTGACAGCGACACGCACGGTGGGTGGCGGGTCATCGGCTGCACGCAGACCCGAT

TGCGCCTCATCCCATTCTGCTGGGGCAGCCGCTGTATGCGGCGTGCCGACGGCAGGCATCGAATCAAGAAGCGATGTATC

AAGAAGCGACGGATCGAACTGACGGAGCAGATCGCTGAAGCTGCCCGCCGAGAACGCAGGCGAGGGCGCAGGGGGAGATG

GCAGCCGGGTCCGGGACATCGTCCGCCGAGCGGGCAAGCCATCCAGGGGGCCGCCAGCAGGCGGAGCCCCCCCCCGATCT

GCAGTCGGCTGAACCCTATCCGGTTGGGGTCCGGGCAGAAGCTCGCGGGCAGGACTTGGCGTGCGCGAACGAATGGGATC

C

>31P3F

ACGCGCCGGAATTGATCAAAAGAATCAATCGCCGCATTCCCGAACGCACGTCCCATCGCGTTGCCGACCTCGCGCACGTG

GTGCGCGTGCTTGGTTTTTTCCAGAGCCACTCCCACCCAGCGCAAGCATTCGATGACGCCATGACGCAGTTCGGGATGAG

CAGGCACGGGTTGGTACAGCTCTTTCGCAGAGTGGGCGTCACCGAATTCGAAGCCCGCTGCGGAACACTCCCCCCAGCCT

CGCAGCGTTGGGACCGTATCCTCCAGGCATCAGGGATGAAAAGGGCCAAACCGTCCCCTACTTCAGCTCAAACGCCGGAT

CAGGCGTCTTTGCATGCATTCGCCGATTCGCTGGAGCGTGACCTTGATGCGCCCAGCCCAATGCACGAGGGAGATCAGAC

GCGGGCAAGCAGCCGTAAACGGTCCCGATCGGATCGTGCTGTCACCGGCCCCTCCACACAGCAATCTTTCGAGGTGCGCG

TTCCCGAACAGCACGATGCGCTGCATTTGCCCCTCAGCTGGAGGGTAAAACGCCCGCGTACCAGGATCGGGGGCGGCCTC

CCGGATCC

>31P3R

CGGTGACGCCCACTCTGCGAAAGAGCTGTACCAACCCGTGCCTGCTCATCCCGAACTGCGTCATGGCGTCATCGAATGCT

TGCGCTGGGTGGGAGTGGCTCTGGAAAAAACCAAGCACGCGCACCACGTGCGCGAGGTCGGCAACGCGATGGGACGTGCG

TTCGGGAATGCGGCGATTGATTCTTTTGATCAATTCCGGCGCGTGCGGCAATCCCTTTTTCACTGCATCCAGGGCAGGAC

GTCCGCCGAGGCAGGCCAAGGCGACGAGGTGGTCGTTGGTCAACGCGGCCAACGCCGGATCAGGGCGAGATAACTGGGCA

ACAATGCTCTCCAGCGCCTGCTTGCCGCCGCCATTGCTGGCGATGGCCACCACCTGGTCCGGGGTCAGGCCATGGTCCTG

GCACAGCACCGGCAACAGCCGCTGCACCGTCTCCAGCGCCTGCTTGCCGCCAATATTGCTGGCGATGGCCACCACCTGGT

CCGGGGTCAGGCCATGGTCCTGGCACAGCACCGGCAACAGCCGCTGCACCGTCTCCAGCGCCTGCTTGCCGCCGTTATTG

TTGGCGATGGCCACCACCTGGGCCGGGGTCAGGCCATGGTCCTGGCACAGCACCGGCAACAGCCGCTGCACCGTCTCCAG

CGCCTGCTTGCCGCCAATATTGCTGGCGATGGCCACCACCTGGTCCGGGGTCAGGCCATGGTCCTGGCACAGCACCGGCA

ACAGCCGCTGCACCGTCTCCAGCGCCTGCTTGCCGCCGTTATTGTTGGCGATGGCCACCACCTGGTCCGGGGTCAGGCCA

TGGTCCTGGCACAGCACCGGCAACAGCCGCTGCACCGTCTCCAGCGCCTGCTTGCCGCCAATATTGCTGGCGATGGCCAC

CACCTGGTCCGGGGTCAGGCCATGGTCCTGGCACAGCACCGGCAACAGCCGCTGCACCGTCTCCAGCGCCTGCTTGCCGC

CAATATTGC

>M13F

AGGTGGTGGCCATCGCCAACAATAACGGCGGCAAGCAGGCGCTGGAGACGGTGCAGCGGCTGTTGCCGGTGCTGTGCCAG

GACCATGGCCTGACCCCGGACCAGGTGGTGGCCATCGCCAGCAATATTGGCGGCAAGCAGGCGCTGGAGACGGTGCAGCG

GCTGTTGCCGGTGCTGTGCCAGGACCATGGCCTGACCCCGGACCAGGTGGTGGCCATCGCCAGCAATGGCGGCGGCAAGC

AGGCGCTGGAGAGCATTGTTGCCCAGTTATCTCGCCCTGATCCGGCGTTGGCCGCGTTGACCAACGACCACCTCGTCGCC

TTGGCCTGCCTCGGCGGACGTCCTGCCCTGGATGCAGTGAAAAAGGGATTGCCGCACGCGCCGGAATTGATCAAAAGAAT

CAATCGCCGCATTCCCGAACGCACGTCCCATCGCGTTGCCGACCTCGCGCACGTGGTGCGCGTGCTTGGTTTTTTCCAGA

GCCACTCCCACCCAGCGCAAGCATTCGATGACGCCATGACGCAGTTCGGGATGAGCAGGCACGGGTTGGTACAGCTCTTT

CGCAGAGTGGGCGTCACCGAATTCGAAGCCCGCTGCGGAACACTCCCCCCAGCCTCGCAGCGTTGGGACCGTATCCTCCA

GGCATCAGGGATGAAAAGGGCCAAACCGTCCCCTACTTCAGCTCAAACGCCGGATCAGGCGTCTTTGCATGCATTCGCCG

ATTCGCTGGAGCGTGACCTTGATGCGCCCAGCCCAATGCACGAGGGAGATCAGACGCGGGCAAGCAGCCGTAAACGGTCC

CGATCGGATCGTGCTGTCACCGGCCCCTCCACACAGCAATCTTTCGAGGTGCGCGTTCCCGAACAGCACGATGCGCTGCA

TTTGCCCCTCAGCTGGAGGGTAAAACGCCCGCGTACCAGGATCGGGGGCGGCCTCCCGGATCC

>M13R

GGATCCCATTCGTTCGCGCACGCCAAGTCCTGCCCGCGAGCTTCTGCCCGGACCCCAACCGGATAGGGTTCAGCCGACTG

CAGATCGGGGGGGGGCTCCGCCTGCTGGCGGCCCCCTGGATGGCTTGCCCGCTCGGCGGACGATGTCCCGGACCCGGCTG

CCATCTCCCCCTGCGCCCTCGCCTGCGTTCTCGGCGGGCAGCTTCAGCGATCTGCTCCGTCAGTTCGATCCGTCGCTTCT

TGATACATCGCTTCTTGATTCGATGCCTGCCGTCGGCACGCCGCATACAGCGGCTGCCCCAGCAGAATGGGATGAGGCGC

AATCGGGTCTGCGTGCAGCCGATGACCCGCCACCCACCGTGCGTGTCGCTGTCACTGCCGCGCGGCCGCCGCGCGCCAAG

CCGGCCCCGCGACGGCGTGCGGCGCAACCCTCCGACGCTTCGCCGGCCGCGCAGGTGGATCTACGCACGCTCGGCTACAG

TCAGCAGCAGCAAGAGAAGATCAAACCGAAGGTGCGTTCGACAGTGGCGCGGCACCACGAGGCACTGGTGGGTCATGGGT

TTACACACGCGCACATCGTTGCGCTCAGCCAACACCCGGCAGCGTTAGGGACCGTTGCTGTCACGTATCAGGACATAATC

AGGGCGTTGCCAGAGGCGACACACGAAGACATCGTTGGCGTCGGCAAACAGTGGTCCGGCGCACGCGCCCTGGAGGCCTT

GCTCACGGAGGCGAGGGAGTTGAGAGGTCCGCCGTTACAGTTGGACACAGGCCAACTTCTCAAGATTGCAAAACGTGGAG

GCGTGACCTCAGTGCAGGCAGTGCATGCATGGCGCAATGCACTGACGGGTGCCCCCCTGAACCTGACCCCGGACCAAGTG

GTGGCCATCGCCAGCAATATTGGCGGCAAGCAGGCGCTGG

**pTAL*_Bam_*_HI_-34**

> pCC2FOS-MscI-1

CCAGGACCATGGCCTGACCCCGGACCAGGTCGTGGCCATCGCCAGCAATATTGGCGGCAAGCAGGCGCTGGAGACGGTGC

AGCGGCTGTTGCCGGTGCTGTGCCAGGACCATGGCCTGACCCCGACCCAAGTGATGGCCATCGCCAACAATAACGGCGGC

AAGCAGGCACTGGAGACGGTGCAGCGGCTGTTGCCGGTGCTGTGCCAGGACCATGGCCTGACCCCGGACCAGGTCGTGGC

CATCGCCAGCCACGATGGCGGCAAGCAGGCGCTGGAGACGGTGCAGCGGCTGTTGCCGGTGCTGTGCCAGGACCATGGCC

TGACCCCGGCCCAGGTCGTGGCCATCGCCAGCAATAGTGGCGGCAAGCAGGCGCTGGAGACGGTGCAGCGGCTGTTGCCG

GTGCTGTGCCAGGACCATGGCCTGACCCCGGACCAGGTCGTGGCCATCGCCAGCCACGATGGCGGCAAGCAGGCGCTGGA

GACGGTGCAGCGGCTGTTGCCGGTGCTGTGCCAGGACCATGGCCTGACCCCGGACCAGGTGGTGGCCATCGCCAGCAATA

GTGGCGGCAAGCAGGCGCTGGAGACGGTGCAGCGGCTGTTGCCGGTGCTGTGCCAGGACCATGGCCTGACCCCGGACCAG

GTGGTGGCCATCGCCAGCAATAGTGGCGGCAAGCAGGCGCTGGAGACGGTGCAGCGGCTGTTGCCGGTGCTGTGCCAGGA

CC

> pCC2FOS-MscI-2

ACGGCGGCAAGCAGGCGCTGGAGACGGTGCAGCGGCTGTTGCCGGTGCTGTGCCAGGACCATGGCCTGACCCCGGACCAG

GTGATGGCCATCGCCAACAATAACGGCGGCAAGCAGGCGCTGGAGACGGTGCAGCGGCTGTTGCCGGTGCTGTGCCAGGA

CCATGGCCTGACCCCGGACCAGGTCGTGGCCATCGCCAGCAATATTGGCGGCAAGCAGGCGCTGGAGACGGTGCAGCGGC

TGTTGCCGGTGCTGTGCCAGGACCATGGCCTGACCCCGACCCAAGTGATGGCCATCGCCAACAATAACGGCGGCAAGCAG

GCACTGGAGACGGTGCAGCGGCTGTTGCCGGTGCTGTGCCAGGACCATGGCCTGACCCCGGACCAGGTCGTGGCCATCGC

CAGCCACGATGGCGGCAAGCAGGCGCTGGAGACGGTGCAGCGGCTGTTGCCGGTGCTGTGCCAGGACCATGGCCTGACCC

CGGCCCAGGTCGTGGCCATCGCCAGCAATAGTGGCGGCAAGCAGGCGCTGGAGACGGTGCAGCGGCTGTTGCCGGTGCTG

TGCCAGGACCATGGCCTGACCCCGGACCAGGTCGTGGCCATCGCCAGCCACGATGGCGGCAAGCAGGCGCTGGAGACGGT

GCAGCGGCTGTTGCCGGTGCTGTGCCAGGACCATGGCCTGACCCCGGACCAGGTGGTGGCCATCGCCAGCAATAGTGGCG

GCAAGCAGGCGCTGGAGACGGTGCAGCGGCTGTTGCCGGTGCTGTGCCAGGACCATGGCCTGACCCCGGACCAGGTGGTG

GCCATCGCCAGCAATAGTGGCGGCAAGCAGGCGCTGGAGACGGTGCAGCGGCTGTTGCCGGTGCTGTGCCAGGACCATGG

CCTGACCCCGGCCCAGGTGGTGGCCATCGCCAGCAATAGTGGCGGCAAGCAGGCGCTGGAGACGGTGCAGCGGCTGTTGC

CGGTGCTGTGCC

> pCC2FOS-MscI-3

ATGGCGGCAAGCAGGCGCTGGAGACGGTGCAGCGGCTGTTGCCGGTGCTGTGCCAGGACCATGGCCTGACCCCGGCCCAG

GTCGTGGCCATCGCCAGCAATAGTGGCGGCAAGCAGGCGCTGGAGACGGTGCAGCGGCTGTTGCCGGTGCTGTGCCAGGA

CCATGGCCTGACCCCGGACCAGGTCGTGGCCATCGCCAGCCACGATGGCGGCAAGCAGGCGCTGGAGACGGTGCAGCGGC

TGTTGCCGGTGCTGTGCCAGGACCATGGCCTGACCCCGGACCAGGTGGTGGCCATCGCCAGCAATAGTGGCGGCAAGCAG

GCGCTGGAGACGGTGCAGCGGCTGTTGCCGGTGCTGTGCCAGGACCATGGCCTGACCCCGGACCAGGTGGTGGCCATCGC

CAGCAATAGTGGCGGCAAGCAGGCGCTGGAGACGGTGCAGCGGCTGTTGCCGGTGCTGTGCCAGGACCATGGCCTGACCC

CGGCCCAGGTGGTGGCCATCGCCAGCAATAGTGGCGGCAAGCAGGCGCTGGAGACGGTGCAGCGGCTGTTGCCGGTGCTG

TGCCAGGACCATGGCCTGACCCCGGACCAAGTCGTGGCCATCGCCAGCCACGATGGCGGCAAGCAGGCGCTGGAGACGGT

GCAGCGGCTGTTGCCGGTGCTGTGCCAGGACCATGGCCTGACCCCGGACCAGGTCGTGGCCATCGCCAACAATAACGGCG

GCAAGCAGGCGCTGGAGACGCTGCAGCGGCTGTTGCCGGTGCTGTGCCAGGACCATGGCCTGACCCCGGACCAAGTGGTG

GCCATCGCCAGCCACGATGGCGGCAAGCAGGCGCTGGAGACGGTGCAGCGGCTGTTGCCGGTGCTGTGCCAGGACCATGG

CCTGACCCCGGACCAGGTCGTGGCCATCGCCAGCAATGGCGGCGGCAAGCAGGCGCTGGAGACGGTGCAACGGCTGTTGC

CGGT

>34P1F_1

AGGCGGGGGAGTTGAGAGGTCCGCCGTTACAGTTGGACACAGGCCAACTTCTCAAGATTGCAAAACGTGGCGGCGTGACC

GCAGTGGAGGCAGTGCATGCATGGCGCAATGCACTGACGGGTGCCCCCCTGAACCTGACCCCGGACCAAGTGGTGGCCAT

CGCCAGCAATATTGGCGGCAAGCAGGCGCTGGAGACGGTGCAGCGGCTGTTACCGGTGCTGTGCCAGGCCCATGGCCTGA

CCCCGGACCAGGTCGTGGCCATCGCCAGCCATGGCGGCGGCAAGCAGGCGCTGGAGACGGTGCAACGGCTGTTGCCGGTG

CTGTGCCAGGACCATGGCCTGACCCCGGACCAGGTCGTGGCCATCGCCAGCAATATTGGCGGCAAGCAGGCGCTGGAGAC

GGTGCAGCGGCTGTTGCCGGTGCTGTGCCAGGACCATGGCCTGACCCCGGACCAGGTGATGGCCATCGCCAACAATAACG

GCGGCAAGCAGGCGCTGGAGACGGTGCAGCGGCTGTTGCCGGTGCTGTGCCAGGACCATGGCCTGACCCCGGACCAGGTG

ATGGCCATCGCCAACAATAACGGCGGCAAGCAGGCGCTGGAGACGGTGCAGCGGCTGTTGCCGGTGCTGTGCCAGGACCA

TGGCCTGACCCCGGACCAGGTCGTGGCCATCGCCAGCAATATTGGCGGCAAGCAGGCGCTGGAGACGGTGCAGCGGCTGT

TGCCGGTGCTGTGCCAGGACCATGGCCTGACCCCGACCCAAGTGATGGCCATCGCCAACAATAACGGCGGCAAGCAGGCA

CTGGAGACGGTGCAGCGGCTGTTGCCGGTGCTGTGCCAGGACCATGGCCTGACCCCGGACCAGGTCGTGGCCATCGCCAG

CCACGATGGCGGCAAGCAGGCGCTGGAGACGGTGCAGCGGCTGTTGCCGGTGCTGTGCCAGGACCATGGCCTGACCCCGG

CCCAGGTCGTGGCCATCGCC

>34P1F_2

AGGCGGGGGAGTTGAGAGGTCCGCCGTTACAGTTGGACACAGGCCAACTTCTCAAGATTGCAAAACGTGGCGGCGTGACC

GCAGTGGAGGCAGTGCATGCATGGCGCAATGCACTGACGGGTGCCCCCCTGAACCTGACCCCGGACCAAGTGGTGGCCAT

CGCCAGCAATATTGGCGGCAAGCAGGCGCTGGAGACGGTGCAGCGGCTGTTACCGGTGCTGTGCCAGGCCCATGGCCTGA

CCCCGGACCAGGTCGTGGCCATCGCCAGCCATGGCGGCGGCAAGCAGGCGCTGGAGACGGTGCAACGGCTGTTGCCGGTG

CTGTGCCAGGACCATGGCCTGACCCCGGACCAGGTCGTGGCCATCGCCAGCAATATTGGCGGCAAGCAGGCGCTGGAGAC

GGTGCAGCGGCTGTTGCCGGTGCTGTGCCAGGACCATGGCCTGACCCCGGACCAGGTGATGGCCATCGCCAACAATAACG

GCGGCAAGCAGGCGCTGGAGACGGTGCAGCGGCTGTTGCCGGTGCTGTGCCAGGACCATGGCCTGACCCCGGACCAGGTG

ATGGCCATCGCCAACAATAACGGCGGCAAGCAGGCGCTGGAGACGGTGCAGCGGCTGTTGCCGGTGCTGTGCCAGGACCA

TGGCCTGACCCCGGACCAGGTCGTGGCCATCGCCAGCAATATTGGCGGCAAGCAGGCGCTGGAGACGGTGCAGCGGCTGT

TGCCGGTGCTGTGCCAGGACCATGGCCTGACCCCGACCCAAGTGATGGCCATCGCCAACAATAACGGCGGCAAGCAGGCA

CTGGAGACGGTGCAGCGGCTGTTGCCGGTGCTGTGCCAGGACCATGGCCTG

>34P1R_1

CTGGGCAACAATGCTCTCCAGCGCCTGCTTGCCGCCGCCATTGCTGGCGATGGCCACCACTTGGTCCGGGGTCAGGCCAT

GGTCCTGGCACAGCACCGGCAACAGCCGCTGCACCGTCTTCAGCGCCTGCTTGCCGCCGCCATTGCTGGCGATGGCCACC

ACCTGGTCCGGGGTCAGGCCATGGTCCTGGCACAGCACCGGCAACAGCCGCTGCACCGTCTCCAGCGCCTGCTTGCCGCC

ATCGTGGCTGGCGATGGCCACCACCTGGTCCGGGGTCAGGCCATGGTCCTGGCACAACACCGGCAACAGCCGCTGCACCG

TCTCCAGCGCCTGCTTGCCGCCATCGTGGCTGGCGATGGCCACCACTTGGGCCGGGGTCAGGCCATGGTCCTGGCACAGC

ACCGGCAACAGCCGCTGCACCGTCTCCAGCGCCTGCTTGCCGCCATCGTGGCTGGCGATGGCCACGACCTGGTCCGGGGT

CAGGCCATGGTCCTGGCACAGCACCGGCAACAGCCGTTGCACCGTCTCCAGCGCCTGCTTGCCGCCGCCATTGCTGGCGA

TGGCCACGACCTGGTCCGGGGTCAGGCCATGGTCCTGGCACAGCACCGGCAACAGCCGCTGCACCGTCTCCAGCGCCTGC

TTGCCGCCATCGTGGCTGGCGATGGCCACCACTTGGTCCGGGGTCAGGCCATGGTCCTGGCACAGCACCGGCAACAGCCG

CTGCAGCGTCTCCAGCGCCTGCTTGCCGCCGTTATTGTTGGCGATGGCCACGACCTGGTCCGGGGTCAGGCCATGGTCCT

GGCACAGCACCGGCAACAGCCGCTGCACCGTCTCCAGCGCCTGCTTGCCGCCATCGTGGCTGGCGATGGCCACGACTTGG

TCCGGGGTCAGGCCATGGTCCTGGCACAGCACCGGCAACAGCCGCTGCACCGTCTCCAGCGCCTGCTTGCCGCCACTATT

GCTGGCGATGGCCAC

>34P1R_2

TGGCCATCGCCAGCAATAGTGGCGGCAAGCAGGCGCTGGAGACGGTGCAGCGGCTGTTGCCGGTGCTGTGCCAGGACCAT

GGCCTGACCCCGGACCAAGTCGTGGCCATCGCCAGCCACGATGGCGGCAAGCAGGCGCTGGAGACGGTGCAGCGGCTGTT

GCCGGTGCTGTGCCAGGACCATGGCCTGACCCCGGACCAGGTCGTGGCCATCGCCAACAATAACGGCGGCAAGCAGGCGC

TGGAGACGCTGCAGCGGCTGTTGCCGGTGCTGTGCCAGGACCATGGCCTGACCCCGGACCAAGTGGTGGCCATCGCCAGC

CACGATGGCGGCAAGCAGGCGCTGGAGACGGTGCAGCGGCTGTTGCCGGTGCTGTGCCAGGACCATGGCCTGACCCCGGA

CCAGGTCGTGGCCATCGCCAGCAATGGCGGCGGCAAGCAGGCGCTGGAGACGGTGCAACGGCTGTTGCCGGTGCTGTGCC

AGGACCATGGCCTGACCCCGGACCAGGTCGTGGCCATCGCCAGCCACGATGGCGGCAAGCAGGCGCTGGAGACGGTGCAG

CGGCTGTTGCCGGTGCTGTGCCAGGACCATGGCCTGACCCCGGCCCAAGTGGTGGCCATCGCCAGCCACGATGGCGGCAA

GCAGGCGCTGGAGACGGTGCAGCGGCTGTTGCCGGTGTTGTGCCAGGACCATGGCCTGACCCCGGACCAGGTGGTGGCCA

TCGCCAGCCACGATGGCGGCAAGCAGGCGCTGGAGACGGTGCAGCGGCTGTTGCCGGTGCTGTGCCAGGACCATGGCCTG

ACCCCGGACCAGGTGGTGGCCATCGCCAGCAATGGCGGCGGCAAGCAGGCGCTGAAGACGGTGCAGCGGCTGTTGCCGGT

GCTGTGCCAGGACCATGGCCTGACCCCGGACCAAGTGGTGGCCATCGCCAGCAATGGCGGCGGCAAGCAGGCGCTGGAGA

GCATTGTTGCCCAGTT

>34P2F

CGCGCACATCGTTGCGCTCAGCCAACACCCGGCAGCGTTAGGGACCGTTGCTGTCACGTATCAGGACATAATCAGGGCGT

TGCCAGAGGCGACACACGAAGACATCGTTGGCGTCGGCAAACAGTGGTCCGGCGCACGCGCCCTGGAGGCCTTGCTCACG

AAGGCGGGGGAGTTGAGAGGTCCGCCGTTACAGTTGGACACAGGCCAACTTCTCAAGATTGCAAAACGTGGCGGCGTGAC

CGCAGTGGAGGCAGTGCATGCATGGCGCAATGCACTGACGGGTGCCCCCCTGAACCTGACCCCGGACCAAGTGGTGGCCA

TCGCCAGCAATATTGGCGGCAAGCAGGCGCTGGAGACGGTGCAGCGGCTGTTACCGGTGCTGTGCCAGGCCCATGGCCTG

ACCCCGGACCAGGTCGTGGCCATCGCCAGCCATGGCGGCGGCAAGCAGGCGCTGGAGACGGTGCAACGGCTGTTGCCGGT

GCTGTGCCAGGACCATGGCCTGACCCCGGACCAGGTCGTGGCCATCGCCAGCAATATTGGCGGCAAGCAGGCGCTGGAGA

CGGTGCAGCGGCTGTTGCCGGTGCTGTGCCAGGACCATGGCCTGACCCCGGACCAGGTGATGGCCATCGCCAACAATAAC

GGCGGCAAGCAGGCGCTGGAGACGGTGCAGCGGCTGTTGCCGGTGCTGTGCCAGGACCATGGCCTGACCCCGGACCAGGT

GATGGCCATCGCCAACAATAACGGCGGCAAGCAGGCGCTGGAGACGGTGCAGCGGCTGTTGCCGGTGCTGTGCCAGGACC

ATGGCCTGACCCCGGACCAGGTCGTGGCCATCGCCAGCAATATTGGCGGCAAGCAGGCGCTGGAGACGGTGCAGCGGCTG

TTGCCGGTGCTGTGCCAGGACCATGGCCTGACCCCGACCCAAGTGATGGCCATCGCCAACAATAACGGCGGCAAGCAGGC

ACTGGAGACGGTGCA

>34P2R

TGATTATGTCCTGATACGTGACAGCAACGGTCCCTAACGCTGCCGGGTGTTGGCTGAGCGCAACGATGTGCGCGTGTGTA

AACCCATGGCCCACCAGTGCCTCGTGGTGCTGCGCCACTGTCGAACGCACCTTCGGTTTGATCTTCTCTTGCTGCTGCTG

ACTGTAGCCGAGCGTGCGTAGATCCACCTGCGCGGCCGGCGAAGCGTCGGAGGGTTGCGCCGCACGCCGCCGCGGGGCCG

GCTTGGCGCGCGGCGGCCGCGCGGCAGTGACAGCGACACGCACGGTGGGTGGCGGGTCATCGGCTGCACGCAGACCCGAT

TGCACCTCATCGCACTCTGCTGGGGCAGCCGCTGTATGCGGCGTGCCGACGGCAGGCATCGAATCAAGAAGCGATGTATC

AAGAAGCGACGGATCGAACTGACGGAGCAGATCGCTGAAGCTGCCCGCCGAGAACGCAGGCGAGGGCGCAGGGGGAGATG

GCAGCCGGGTCCGGGACATCGTCCGCCGAGCGGGCAAGCCATCCAGGGGGCCGCCAGCAGGCGGAGCCCCCCCCCCCCGA

TCTGCAGTCGGCTGAACCCTATCCGGTTGGGGTCCGGGCAGAAGCTCGCGGGCAGGACTTGGCGTGCGCGAACGAATGGG

ATCC

>34P3F

CGCACGCGCCAGAATTGATCAGAAGAATCAATCGCCGCATTCCCGAACGCACGTCCCATCGCGTTGCCGACCTCGCGCAC

GTGGTTCGCGTGCTTGGTTTTTTCCAGAGCCACTCCCACCCAGCGCAAGCATTCGATGACGCCATGACGCAGTTCGGGAT

GAGCAGGCACGGGTTGGTACAGCTCTTTCGCAGAGTGGGCGTCACCGAATTCGAAGCCCGCTACGGAACGCTCCCCCCAG

CCTCGCAGCGTTGGGACCGTATCCTCCAGGCATCAGGGATGAAAAGGGCCAAACCGTCCCCTACTTCAGCTCAAACGCCG

GATCAGGCGTCTTTGCATGCATTCGCCGATTCGCTGGAGCGTGACCTTGATGCGCCCAGCCCAATGCACGAGGGAGATCA

GACGCGGGCAAGCAGCCGTAAACGGTCCCGATCGGATCGTGCTGTCACCGACCCCTCCGCACAGCAATCTTTCGAGGTGC

GCGTTCCCGAACAGCACGATACGCTGCATTTGCCCCTCAGCTGGAGGGTAAAACGCCCGCGTACCAGGATCGGGGGCGGC

CTCCCGGATCC

>34P3R

CGGTGACGCCCACTCTGCGAAAGAGCTGTACCAACCCGTGCCTGCTCATCCCGAACTGCGTCATGGCGTCATCGAATGCT

TGCGCTGGGTGGGAGTGGCTCTGGAAAAAACCAAGCACGCGAACCACGTGCGCGAGGTCGGCAACGCGATGGGACGTGCG

TTCGGGAATGCGGCGATTGATTCTTCTGATCAATTCTGGCGCGTGCGGCAATCCCTTTTTCACTGCATCCAGGGCAGGAC

GCCCGCCGAGGCAGGCCAAGGCGACGAGGTGGTCGTTGGTCAACGCGGCCAACGCCGGATCAGGGCAAGATAACTGGGCA

ACAATGCTCTCCAGCGCCTGCTTGCCGCCGCCATTGCTGGCGATGGCCACCACTTGGTCCGGGGTCAGGCCATGGTCCTG

GCACAGCACCGGCAACAGCCGCTGCACCGTCTTCAGCGCCTGCTTGCCGCCGCCATTGCTGGCGATGGCCACCACCTGGT

CCGGGGTCAGGCCATGGTCCTGGCACAGCACCGGCAACAGCCGCTGCACCGTCTCCAGCGCCTGCTTGCCGCCATCGTGG

CTGGCGATGGCCACCACCTGGTCCGGGGTCAGGCCATGGTCCTGGCACAACACCGGCAACAGCCGCTGCACCGTCTCCAG

CGCCTGCTTGCCGCCATCGTGGCTGGCGATGGCCACCACTTGGGCCGGGGTCAGGCCATGGTCCTGGCACAGCACCGGCA

ACAGCCGCTGCACCGTCTCCAGCGCCTGCTTGCCGCCATCGTGGCTGGCGATGGCCACGACCTGGTCCGGGGTCAGGCCA

TGGTCCTGGCACAGCACCGGCAACAGCCGTTGCACCGTCTCCAGCGCCTGCTTGCCGCCGCCATTGCTGGCGATGGCCAC

GACCTGGTCCGGGGTCAGGCCATGGTCCTGGCACAGCACCGGCAACAGCCGCTGCACCGTCTCCAGCGCCTGCTTGCCGC

CATCGTGGCTGGC

>M13F

GGATCCCATTCGTTCGCGCACGCCAAGTCCTGCCCGCGAGCTTCTGCCCGGACCCCAACCGGATAGGGTTCAGCCGACTG

CAGATCGGGGGGGGGGGGCTCCGCCTGCTGGCGGCCCCCTGGATGGCTTGCCCGCTCGGCGGACGATGTCCCGGACCCGG

CTGCCATCTCCCCCTGCGCCCTCGCCTGCGTTCTCGGCGGGCAGCTTCAGCGATCTGCTCCGTCAGTTCGATCCGTCGCT

TCTTGATACATCGCTTCTTGATTCGATGCCTGCCGTCGGCACGCCGCATACAGCGGCTGCCCCAGCAGAGTGCGATGAGG

TGCAATCGGGTCTGCGTGCAGCCGATGACCCGCCACCCACCGTGCGTGTCGCTGTCACTGCCGCGCGGCCGCCGCGCGCC

AAGCCGGCCCCGCGGCGGCGTGCGGCGCAACCCTCCGACGCTTCGCCGGCCGCGCAGGTGGATCTACGCACGCTCGGCTA

CAGTCAGCAGCAGCAAGAGAAGATCAAACCGAAGGTGCGTTCGACAGTGGCGCAGCACCACGAGGCACTGGTGGGCCATG

GGTTTACACACGCGCACATCGTTGCGCTCAGCCAACACCCGGCAGCGTTAGGGACCGTTGCTGTCACGTATCAGGACATA

ATCAGGGCGTTGCCAGAGGCGACACACGAAGACATCGTTGGCGTCGGCAAACAGTGGTCCGGCGCACGCGCCCTGGAGGC

CTTGCTCACGAAGGCGGGGGAGTTGAGAGGTCCGCCGTTACAGTTGGACACAGGCCAACTTCTCAAGATTGCAAAACGTG

GCGGCGTGACCGCAGTGGAGGCAGTGCATGCATGGCGCAATGCACTGACGGGTGCCCCCCTGAACCTGACCCCGGACCAA

GTGGTGGCCATCGCCAGCAATATTGGCGGCAAGCAGGCGCTGGAGACGGTGCAGCGGCTGTTACCGGTGCTGTGCCAGGC

CCA

>M13R

CCGGACCAGGTGGTGGCCATCGCCAGCCACGATGGCGGCAAGCAGGCGCTGGAGACGGTGCAGCGGCTGTTGCCGGTGCT

GTGCCAGGACCATGGCCTGACCCCGGACCAGGTGGTGGCCATCGCCAGCAATGGCGGCGGCAAGCAGGCGCTGAAGACGG

TGCAGCGGCTGTTGCCGGTGCTGTGCCAGGACCATGGCCTGACCCCGGACCAAGTGGTGGCCATCGCCAGCAATGGCGGC

GGCAAGCAGGCGCTGGAGAGCATTGTTGCCCAGTTATCTTGCCCTGATCCGGCGTTGGCCGCGTTGACCAACGACCACCT

CGTCGCCTTGGCCTGCCTCGGCGGGCGTCCTGCCCTGGATGCAGTGAAAAAGGGATTGCCGCACGCGCCAGAATTGATCA

GAAGAATCAATCGCCGCATTCCCGAACGCACGTCCCATCGCGTTGCCGACCTCGCGCACGTGGTTCGCGTGCTTGGTTTT

TTCCAGAGCCACTCCCACCCAGCGCAAGCATTCGATGACGCCATGACGCAGTTCGGGATGAGCAGGCACGGGTTGGTACA

GCTCTTTCGCAGAGTGGGCGTCACCGAATTCGAAGCCCGCTACGGAACGCTCCCCCCAGCCTCGCAGCGTTGGGACCGTA

TCCTCCAGGCATCAGGGATGAAAAGGGCCAAACCGTCCCCTACTTCAGCTCAAACGCCGGATCAGGCGTCTTTGCATGCA

TTCGCCGATTCGCTGGAGCGTGACCTTGATGCGCCCAGCCCAATGCACGAGGGAGATCAGACGCGGGCAAGCAGCCGTAA

ACGGTCCCGATCGGATCGTGCTGTCACCGACCCCTCCGCACAGCAATCTTTCGAGGTGCGCGTTCCCGAACAGCACGATA

CGCTGCATTTGCCCCTCAGCTGGAGGGTAAAACGCCCGCGTACCAGGATCGGGGGCGGCCTCCCGGATCC

**pTAL*_Bam_*_HI_-36**

> pCC2FOS-MscI-1

GGCACAGCACCGGCAACAGCCGTTGCACCGTCTCCAGCGCCTGCTTGCCGCCGCCATTGCTGGCGATGGCCACGACCTGG

TCCGGGGTCAGGCCATGGTCCTGGCACAGCACCGGCAACAGCCGCTGCACCGTCTCCAGCGCCTGCTTGCCGCCACTATT

GCTGGCGATGGCCACCACCTGGGCCGGGGTCAGGCCATGGTCCTGGCACAGCACCGGCAACAGCCGCTGCACCGTCTCCA

GCGCCTGCTTGCCGCCATCGTGGCTGGCGAGGGCCACCACTTGGGCCGGGGTCAGGCCATGGTCCTGGCACAGCACCGGC

AACAGCCGCTGCAGCGTCTCCAGCGCCTGCTTGCCGCCGCCATGGCTGGCGATGGCCAGCACCTGGTCCGGGGTCAGGCC

ATGGGCCTGGCACAGCACCGGCAACAGCCGTTGCAGCGTCTCCAGCGCCTGCTTGCCGCCGCCATTGCTGGCGATGGCCA

CCACCTGGTCCGGGGTCAGGCCATGGTCCTGGCACAGCACCGGCAACAGCCGCTGCACCGTCTCCAGCGCCTGCTTGCCG

CCAATATTGCTGGCGATGGCCACCACCTGGTCCGGGGTCAGGCCATGGTCCTGGCACAGCACCGGCAACAGCCGCTGCAG

CGTCTCCAGCGCCTGCTTGCCGCCGCCATGGCTGGCGATGGCCACGACCTGGTCCGGGGTCAGGCCATGGGCCTGGCACA

GCACCGGCAACAGCCGCTGCACCGTCTCCAGCGCCTGGTTGCCGCCAATATTGCTGGCGATGG

> pCC2FOS-MscI-2

CGGCGGCAAGCAGGCGCTGGAGACGGTGCAACGGCTGTTGCTGGTGCTGTGCCAGGCCCATGGCCTGACCCCGGACCAGG

TCGTGGCCATCGCCAGCAATGGCGGCGGCAAGCAGGCGCTGGAGACGGTGCAACGGCTGTTGCCGGTGCTGTGCCAGGAC

CATGGCCTGACCCCGGACCAGGTGGTGGCCATCGCCAGCCATGGCGGCGGCAAGCAGGCGCTGGAGACGGTGCAGCGGCT

GTTGCCGGTGCTGTGCCAGGACCATGGCCTGACCCCGGACCAGGTCGTGGCCATCGCCAGCAATGGCGGCGGCAAGCAGG

CGCTGGAGACGGTGCAGCGGCTGTTGCCGGTGCTGTGCCAGGACCATGGCCTGACCCCGGACCAGGTGGTGGCCATCGCC

AGCCACGATGGCGGCAAGCAGGCGCTGGAGACGGTGCAGCGGCTGTTGCCGGTGCTGTGCCAGGACCATGGCCTGACCCC

GGACCAGGTGGTGGCCATCGCCAGCCATGGCGGCGGCAAGCAGGCGCTGGAGACAGTGCAGCGGCTGTTGCCGGTGCTGT

GCCAGGACCATGGCCTGACCCCGGACCAGGTCGTGGCCATCGCCAGCCACGATGGCGGCAAGCAGGCGCTGGAGACGGTG

CAGCGGCTGTTGCCGGTGCTGTGCCAGGACCATGGCCTGACCCCGGACCAGGTGGTGGCCATCGCCAGCCACGATGGCGG

CAAGCAGGCGCTGGAGACGGTGCAGCGGCTGTTGCCGGTGCTGTGCCAGGACCATGGCCTGACCCTGGACCAGGTGGTGG

CCATCGCCAGCAATATTGGCGGCAAGCAGGCGCTGGAGACGGTGCAGCGGCTGTTGCCGGTGCTGTGCCAGGACCATGGC

CTGACCCCGGACCAGGTGGTGGCCATCGCCAACAATAACGGCGGCAAGCAGGCGCTGGAGACGGTGCAGCGGCTGTTGCC

GGTGCTGTGCCAGGACCA

> pCC2FOS-MscI-3

CCATCGCCAGCAATATTGGCGGCAACCAGGCGCTGGAGACGGTGCAGCGGCTGTTGCCGGTGCTGTGCCAGGCCCATGGC

CTGACCCCGGACCAGGTCGTGGCCATCGCCAGCCATGGCGGCGGCAAGCAGGCGCTGGAGACGCTGCAGCGGCTGTTGCC

GGTGCTGTGCCAGGACCATGGCCTGACCCCGGACCAGGTGGTGGCCATCGCCAGCAATATTGGCGGCAAGCAGGCGCTGG

AGACGGTGCAGCGGCTGTTGCCGGTGCTGTGCCAGGACCATGGCCTGACCCCGGACCAGGTGGTGGCCATCGCCAGCAAT

GGCGGCGGCAAGCAGGCGCTGGAGACGCTGCAACGGCTGTTGCCGGTGCTGTGCCAGGCCCATGGCCTGACCCCGGACCA

GGTGCTGGCCATCGCCAGCCATGGCGGCGGCAAGCAGGCGCTGGAGACGCTGCAGCGGCTGTTGCCGGTGCTGTGCCAGG

ACCATGGCCTGACCCCGGCCCAAGTGGTGGCCCTCGCCAGCCACGATGGCGGCAAGCAGGCGCTGGAGACGGTGCAGCGG

CTGTTGCCGGTGCTGTGCCAGGACCATGGCCTGACCCCGGCCCAGGTGGTGGCCATCGCCAGCAATAGTGGCGGCAAGCA

GGCGCTGGAGACGGTGCAGCGGCTGTTGCCGGTGCTGTGCCAGGACCATGGCCTGACCCCGGACCAGGTCGTGGCCATCG

CCAGCAATGGCGGCGGCAAGCAGGCGCTGGAGACGGTGCAACGGCTGTTGCCGGTGCTGTGCCAGGACCATGGCCTGACC

CCGGACCAGGTGGTGGCCATCGCCAGCCACGATGGCGGCAAGCAGGCGCTGGAGACGGTGCAGCGGCTGTTGCCGGTGCT

GTGCCAGGACC

> pCC2FOS-MscI-4

CCATCGCCAGCAATGGCGGCGGCAAGCAGGCGCTGGAGACGCTGCAACGGCTGTTGCCGGTGCTGTGCCAGGCCCATGGC

CTGACCCCGGACCAGGTGCTGGCCATCGCCAGCCATGGCGGCGGCAAGCAGGCGCTGGAGACGCTGCAGCGGCTGTTGCC

GGTGCTGTGCCAGGACCATGGCCTGACCCCGGCCCAAGTGGTGGCCCTCGCCAGCCACGATGGCGGCAAGCAGGCGCTGG

AGACGGTGCAGCGGCTGTTGCCGGTGCTGTGCCAGGACCATGGCCTGACCCCGGCCCAGGTGGTGGCCATCGCCAGCAAT

AGTGGCGGCAAGCAGGCGCTGGAGACGGTGCAGCGGCTGTTGCCGGTGCTGTGCCAGGACCATGGCCTGACCCCGGACCA

GGTCGTGGCCATCGCCAGCAATGGCGGCGGCAAGCAGGCGCTGGAGACGGTGCAACGGCTGTTGCCGGTGCTGTGCCAGG

ACCATGGCCTGACCCCGGACCAGGTGGTGGCCATCGCCAGCCACGATGGCGGCAAGCAGGCGCTGGAGACGGTGCAGCGG

CTGTTGCCGGTGCTGTGCCAGGACCATGGCCTGACCCCGGACCAGGTCGTGGCCATCGCCAACAATAACGGCGGCAAGCA

GGCGCTGGAGACGGTGCAACGGCTGTTGCTGGTGCTGTGCCAGGCCCATGGCCTGACCCCGGACCAGGTCGTGGCCATCG

CCAGCAATGGCGGCGGCAAGCAGGCGCTGGAGACGGTGCAACGGCTGTTGCCGGTGCTGTGCCAGGACCATGGCCTGACC

CCGGACCAGGTGGTGGCCATCGCCAGCCATGGCGGCGGCAAGCAGGCGCTGGAGACGGTGCAGCGGCTGTTGCCGGTGCT

GTGCCAGGACCA

> pCC2FOS-MscI-5

CCTGGCACAGCACCGGCAACAGCCGCTGCACCGTCTCCAGCGCCTGCTTGCCGCCATCGTGGCTGGCGATGGCCACGACC

TGGTCCGGGGTCAGGCCATGGTCCTGGCACAGCACCGGCAACAGCCGCTGCACTGTCTCCAGCGCCTGCTTGCCGCCGCC

ATGGCTGGCGATGGCCACCACCTGGTCCGGGGTCAGGCCATGGTCCTGGCACAGCACCGGCAACAGCCGCTGCACCGTCT

CCAGCGCCTGCTTGCCGCCATCGTGGCTGGCGATGGCCACCACCTGGTCCGGGGTCAGGCCATGGTCCTGGCACAGCACC

GGCAACAGCCGCTGCACCGTCTCCAGCGCCTGCTTGCCGCCGCCATTGCTGGCGATGGCCACGACCTGGTCCGGGGTCAG

GCCATGGTCCTGGCACAGCACCGGCAACAGCCGCTGCACCGTCTCCAGCGCCTGCTTGCCGCCGCCATGGCTGGCGATGG

CCACCACCTGGTCCGGGGTCAGGCCATGGTCCTGGCACAGCACCGGCAACAGCCGTTGCACCGTCTCCAGCGCCTGCTTG

CCGCCGCCATTGCTGGCGATGGCCACGACCTGGTCCGGGGTCAGGCCATGGGCCTGGCACAGCACCAGCAACAGCCGTTG

CACCGTCTCCAGCGCCTGCTTGCCGCCGTTATTGTTGGCGATGGCCACGACCTGGTCCGGGGTCAGGCCATGGTCCTGGC

ACAGCACCGGCAACAGCCGCTGCACCGTCTCCAGCGCCTGCTTGCCGCCATCGTGGCTGGCGATGGCCACCACCTGGTCC

GGGGTCAGGCCATGGTCCTGGCACAGCACCGGCAACAGCCGTTGCACCGTCTCCAGCGCCTGCTTGCCGCCGCCATTGCT

GGCGATGGCCACGACCTGGTCCGGGGTCAGGCCATGGTCCTGGCACAGCACCGGCAACAGCCGCTGCACCGTCTCCAGCG

CCTGCTTGCCGCCAC

> pCC2FOS-MscI-6

CCTGGCACAGCACCGGCAACAGCCGCTGCACTGTCTCCAGCGCCTGCTTGCCGCCGCCATGGCTGGCGATGGCCACCACC

TGGTCCGGGGTCAGGCCATGGTCCTGGCACAGCACCGGCAACAGCCGCTGCACCGTCTCCAGCGCCTGCTTGCCGCCATC

GTGGCTGGCGATGGCCACCACCTGGTCCGGGGTCAGGCCATGGTCCTGGCACAGCACCGGCAACAGCCGCTGCACCGTCT

CCAGCGCCTGCTTGCCGCCGCCATTGCTGGCGATGGCCACGACCTGGTCCGGGGTCAGGCCATGGTCCTGGCACAGCACC

GGCAACAGCCGCTGCACCGTCTCCAGCGCCTGCTTGCCGCCGCCATGGCTGGCGATGGCCACCACCTGGTCCGGGGTCAG

GCCATGGTCCTGGCACAGCACCGGCAACAGCCGTTGCACCGTCTCCAGCGCCTGCTTGCCGCCGCCATTGCTGGCGATGG

CCACGACCTGGTCCGGGGTCAGGCCATGGGCCTGGCACAGCACCAGCAACAGCCGTTGCACCGTCTCCAGCGCCTGCTTG

CCGCCGTTATTGTTGGCGATGGCCACGACCTGGTCCGGGGTCAGGCCATGGTCCTGGCACAGCACCGGCAACAGCCGCTG

CACCGTCTCCAGCGCCTGCTTGCCGCCATCGTGGCTGGCGATGGCCACCACCTGGTCCGGGGTCAGGCCATGGTCCTGGC

ACAGCACCGGCAACAGCCGTTGCACCGTCTCCAGCGCCTGCTTGCCGCCGCCATTGCTGGCGATGGCCACGACCTGGTCC

GGGGTCAGG

>36P1F_1

AGGCGGGGGAGTTGAGAGGTCCGCCGTTACAGTTGGACACAGGCCAACTTCTCAAGATTGCAAAACGTGGCGGCGTGACC

GCAGTGGAGGCAGTGCATGCATGGCGCAATGCACTGACGGGTGCCCCCCTGAACCTGACCCCGGACCAAGTGGTGGCCAT

CGCCAGCAATATTGGCGGCAACCAGGCGCTGGAGACGGTGCAGCGGCTGTTGCCGGTGCTGTGCCAGGCCCATGGCCTGA

CCCCGGACCAGGTCGTGGCCATCGCCAGCCATGGCGGCGGCAAGCAGGCGCTGGAGACGCTGCAGCGGCTGTTGCCGGTG

CTGTGCCAGGACCATGGCCTGACCCCGGACCAGGTGGTGGCCATCGCCAGCAATATTGGCGGCAAGCAGGCGCTGGAGAC

GGTGCAGCGGCTGTTGCCGGTGCTGTGCCAGGACCATGGCCTGACCCCGGACCAGGTGGTGGCCATCGCCAGCAATGGCG

GCGGCAAGCAGGCGCTGGAGACGCTGCAACGGCTGTTGCCGGTGCTGTGCCAGGCCCATGGCCTGACCCCGGACCAGGTG

CTGGCCATCGCCAGCCATGGCGGCGGCAAGCAGGCGCTGGAGACGCTGCAGCGGCTGTTGCCGGTGCTGTGCCAGGACCA

TGGCCTGACCCCGGCCCAAGTGGTGGCCCTCGCCAGCCACGATGGCGGCAAGCAGGCGCTGGAGACGGTGCAGCGGCTGT

TGCCGGTGCTGTGCCAGGACCATGGCCTGACCCCGGCCCAGGTGGTGGCCATCGCCAGCAATAGTGGCGGCAAGCAGGCG

CTGGAGACGGTGCAGCGGCTGTTGCCGGTGCTGTGCCAGGACCATGGCCTGACCCCGGACCAGGTCGTGGCCATCGCCAG

CAATGGCGGCGGCAAGCAGGCGCTGGAGACGGTGCAACGGCTGTTGCCGGTGCTGTGCCAGGACCATGGCCTGACCCCGG

ACCAGGTGGTGGCCATCGCCAG

>36P1F_2

GGCCATCGCCAGCAATGGCGGCGGCAAGCAGGCGCTGGAGACGGTGCAACGGCTGTTGCCGGTGCTGTGCCAGGACCATG

GCCTGACCCCGGACCAGGTGGTGGCCATCGCCAGCCATGGCGGCGGCAAGCAGGCGCTGGAGACGGTGCAGCGGCTGTTG

CCGGTGCTGTGCCAGGACCATGGCCTGACCCCGGACCAGGTCGTGGCCATCGCCAGCAATGGCGGCGGCAAGCAGGCGCT

GGAGACGGTGCAGCGGCTGTTGCCGGTGCTGTGCCAGGACCATGGCCTGACCCCGGACCAGGTGGTGGCCATCGCCAGCC

ACGATGGCGGCAAGCAGGCGCTGGAGACGGTGCAGCGGCTGTTGCCGGTGCTGTGCCAGGACCATGGCCTGACCCCGGAC

CAGGTGGTGGCCATCGCCAGCCATGGCGGCGGCAAGCAGGCGCTGGAGACAGTGCAGCGGCTGTTGCCGGTGCTGTGCCA

GGACCATGGCCTGACCCCGGACCAGGTCGTGGCCATCGCCAGCCACGATGGCGGCAAGCAGGCGCTGGAGACGGTGCAGC

GGCTGTTGCCGGTGCTGTGCCAGGACCATGGCCTGACCCCGGACCAGGTGGTGGCCATCGCCAGCCACGATGGCGGCAAG

CAGGCGCTGGAGACGGTGCAGCGGCTGTTGCCGGTGCTGTGCCAGGACCATGGCCTGACCCTGGACCAGGTGGTGGCCAT

CGCCAGCAATATTGGCGGCAAGCAGGCGCTGGAGACGGTGCAGCGGCTGTTGCCGGTGCTGTGCCAGGACCATGGCCTGA

CCCCGGACCAGGTGGTGGCCATCGCCAACAATAACGGCGGCAAGCAGGCGCTGGAGACGGTGCAGCGGCTGTTGCCGGTG

CTGTGCCAGGACCATGGCCTGACCCCGGACCAGGTGGTGGCCATCGCCAGCAATGGCGGCGGCAAGCAGGCGCTGGAGAG

CATTGTTGCCCAGTTAT

>36P1F_2

GGCGGGGGAGTTGAGAGGTCCGCCGTTACAGTTGGACACAGGCCAACTTCTCAAGATTGCAAAACGTGGCGGCGTGACCG

CAGTGGAGGCAGTGCATGCATGGCGCAATGCACTGACGGGTGCCCCCCTGAACCTGACCCCGGACCAAGTGGTGGCCATC

GCCAGCAATATTGGCGGCAACCAGGCGCTGGAGACGGTGCAGCGGCTGTTGCCGGTGCTGTGCCAGGCCCATGGCCTGAC

CCCGGACCAGGTCGTGGCCATCGCCAGCCATGGCGGCGGCAAGCAGGCGCTGGAGACGCTGCAGCGGCTGTTGCCGGTGC

TGTGCCAGGACCATGGCCTGACCCCGGACCAGGTGGTGGCCATCGCCAGCAATATTGGCGGCAAGCAGGCGCTGGAGACG

GTGCAGCGGCTGTTGCCGGTGCTGTGCCAGGACCATGGCCTGACCCCGGACCAGGTGGTGGCCATCGCCAGCAATGGCGG

CGGCAAGCAGGCGCTGGAGACGCTGCAACGGCTGTTGCCGGTGCTGTGCCAGGCCCATGGCCTGACCCCGGACCAGGTGC

TGGCCATCGCCAGCCATGGCGGCGGCAAGCAGGCGCTGGAGACGCTGCAGCGGCTGTTGCCGGTGCTGTGCCAGGACCAT

GGCCTGACCCCGGCCCAAGTGGTGGCCCTCGCCAGCCACGATGGCGGCAAGCAGGCGCTGGAGACGGTGCAGCGGCTGTT

GCCGGTGCTGTGCCAGGACCATGGCCTGACCCCGGCCCAGGTGGTGGCCATCGCCAGC

>36P1R_1

CTGGGCAACAATGCTCTCCAGCGCCTGCTTGCCGCCGCCATTGCTGGCGATGGCCACCACCTGGTCCGGGGTCAGGCCAT

GGTCCTGGCACAGCACCGGCAACAGCCGCTGCACCGTCTCCAGCGCCTGCTTGCCGCCGTTATTGTTGGCGATGGCCACC

ACCTGGTCCGGGGTCAGGCCATGGTCCTGGCACAGCACCGGCAACAGCCGCTGCACCGTCTCCAGCGCCTGCTTGCCGCC

AATATTGCTGGCGATGGCCACCACCTGGTCCAGGGTCAGGCCATGGTCCTGGCACAGCACCGGCAACAGCCGCTGCACCG

TCTCCAGCGCCTGCTTGCCGCCATCGTGGCTGGCGATGGCCACCACCTGGTCCGGGGTCAGGCCATGGTCCTGGCACAGC

ACCGGCAACAGCCGCTGCACCGTCTCCAGCGCCTGCTTGCCGCCATCGTGGCTGGCGATGGCCACGACCTGGTCCGGGGT

CAGGCCATGGTCCTGGCACAGCACCGGCAACAGCCGCTGCACTGTCTCCAGCGCCTGCTTGCCGCCGCCATGGCTGGCGA

TGGCCACCACCTGGTCCGGGGTCAGGCCATGGTCCTGGCACAGCACCGGCAACAGCCGCTGCACCGTCTCCAGCGCCTGC

TTGCCGCCATCGTGGCTGGCGATGGCCACCACCTGGTCCGGGGTCAGGCCATGGTCCTGGCACAGCACCGGCAACAGCCG

CTGCACCGTCTCCAGCGCCTGCTTGCCGCCGCCATTGCTGGCGATGGCCACGACCTGGTCCGGGGTCAGGCCATGGTCCT

GGCACAGCACCGGCAACAGCCGCTGCACCGTCTCCAGCGCCTGCTTGCCGCCGCCATGGCTGGCGATGGCCACCACCTGG

TCCGGGGTCAGGCCATGGTCCTGGCACAGCACCGGCAACAGCCGTTGCACCGTCTCCAGCGCCTGCTTGCCGCCGCCATT

GCTGGCGATGGCCAC

>36P2F

CGCGCACATCGTTGCGCTCAGCCAACACCCGGCAGCGTTAGGGACCGTTGCTGTCACGTATCAGGACATAATCAGGGCGT

TGCCAGAGGCGACACACGAAGACATCGTTGGCGTCGGCAAACAGTGGTCCGGCGCACGCGCCCTGGAGGCCTTGCTCACG

AAGGCGGGGGAGTTGAGAGGTCCGCCGTTACAGTTGGACACAGGCCAACTTCTCAAGATTGCAAAACGTGGCGGCGTGAC

CGCAGTGGAGGCAGTGCATGCATGGCGCAATGCACTGACGGGTGCCCCCCTGAACCTGACCCCGGACCAAGTGGTGGCCA

TCGCCAGCAATATTGGCGGCAACCAGGCGCTGGAGACGGTGCAGCGGCTGTTGCCGGTGCTGTGCCAGGCCCATGGCCTG

ACCCCGGACCAGGTCGTGGCCATCGCCAGCCATGGCGGCGGCAAGCAGGCGCTGGAGACGCTGCAGCGGCTGTTGCCGGT

GCTGTGCCAGGACCATGGCCTGACCCCGGACCAGGTGGTGGCCATCGCCAGCAATATTGGCGGCAAGCAGGCGCTGGAGA

CGGTGCAGCGGCTGTTGCCGGTGCTGTGCCAGGACCATGGCCTGACCCCGGACCAGGTGGTGGCCATCGCCAGCAATGGC

GGCGGCAAGCAGGCGCTGGAGACGCTGCAACGGCTGTTGCCGGTGCTGTGCCAGGCCCATGGCCTGACCCCGGACCAGGT

GCTGGCCATCGCCAGCCATGGCGGCGGCAAGCAGGCGCTGGAGACGCTGCAGCGGCTGTTGCCGGTGCTGTGCCAGGACC

ATGGCCTGACCCCGGCCCAAGTGGTGGCCCTCGCCAGCCACGATGGCGGCAAGCAGGCGCTGGAGACGGTGCAGCGGCTG

TTGCCGGTGCTGTGCCAGGACCATGGCCTGACCCCGGCCCAGGTGGTGGCCATCGCCAGCAATAGTGGCGGCAAGCAGGC

GCTGGAGACGGTGC

>36P2R

CCCTGATTATGTCCTGATACGTGACAGCAACGGTCCCTAACGCTGCCGGGTGTTGGCTGAGCGCAACGATGTGCGCGTGT

GTAAACCCATGGCCCACCAGTGCCTCGTGGTGCTGCGCCACTGTCGAACGCACCTTCGGTTTGATCTTCTCTTGCTGCTG

CTGACTGTAGCCGAGCGTGCGTAGATCCACCTGCGCGGCCGGCGAAGCGTCGGAGGGTTGCGCCGCACGCCGTCGCGGGG

CCGGCTTAGCGCGCGGCGGCCGCGCGGCAGTGACAGCGACACGCACGGTGGGTGGCGGGTCATCGGCTGCACGCAGACCC

GATTGCACCTCATCCCACTCTGCTGGGGCAGCCGCTGTATGCGGCGTGCCGACGGCAGGAATCGAATCAAGAAGCGATGT

ATCAAGAAGCGACGGATCGAACTGACGGAGCAGATCGCTGAAGCTGCCCGCCGAGAACGCAGGCGAGGGCGCAGGGGGAG

ATGGCAGCCGGGTCCGGGACATCGTCCGCCGAGCGGGCAAGCCATCCAGGGGGCCGCCAGCAGGCGGAGCCCCCCCCCGA

TCTGCAGTCGGCTGAACCCTATCCGGTTGGGGTCCGGGCAGAAGCTCGCGGGCAGGACTTGGCGTGCGCGAACGAATGGG

ATCC

>36P3F

ATTGCCGCACGCGCCGGAATTGATCAGAAGAATCAATCGCCGCATTCCCGAACGCACGTCCCATCGCGTTGCCGACCTCC

CCGAACGCACGTCCAATCGCGTTGCCGACCTCGCGCACGTGGTGCGCGTGCTTGGTTTTTTCCAGAGCCACTCCCACCCA

GCGCAAGCATTCGATGACGCCATGACGCAGTTCGGGATGAGCAGGCACGGGTTGGTACAGCTCTTTCGCAGAGTGGGCGT

CACCGAACTCGAAGCCCGCTGCGGAACGCTCCCCCCAGCCTCGCAGCGTTGGGACCGTATCCTCCAGGCATCAGGGATGA

AAAGGGCCAAACCGTCCCCTACTTCAGCTCAAACACCGGATCAGGCGTCTTTGCATGCATTCGCCGATTCGCTGGAGCGT

GACCTTGATGCGCCCAGCCCAATGCACGAGGGAGATCAGACGCGGGCAAGCAGCCGTAAACGGTCCCGATCGGATCGTGC

TGTCACCGGCCCCTCCGCACAGCAATCTTTCGAGGTACGCGTTCCCGAACAGCGCGATGCGCTGCATTTGCCCCTCAGCT

GGAGGGTAAAACGCCCGCGTACCAGGATCGGGGGCGGCCTCCCGGATCC

>36P3R

CTTCGAGTTCGGTGACGCCCACTCTGCGAAAGAGCTGTACCAACCCGTGCCTGCTCATCCCGAACTGCGTCATGGCGTCA

TCGAATGCTTGCGCTGGGTGGGAGTGGCTCTGGAAAAAACCAAGCACGCGCACCACGTGCGCGAGGTCGGCAACGCGATT

GGACGTGCGTTCGGGGAGGTCGGCAACGCGATGGGACGTGCGTTCGGGAATGCGGCGATTGATTCTTCTGATCAATTCCG

GCGCGTGCGGCAATCCCTTTTTCACTGCATCCAGGGCAGGACGTCCGCCGAGGCAGGCCAAGGCGACGAGGTGGTCGTTG

GTCAACGCGGCCAACGCCGGATCAGGGCAAGATAACTGGGCAACAATGCTCTCCAGCGCCTGCTTGCCGCCGCCATTGCT

GGCGATGGCCACCACCTGGTCCGGGGTCAGGCCATGGTCCTGGCACAGCACCGGCAACAGCCGCTGCACCGTCTCCAGCG

CCTGCTTGCCGCCGTTATTGTTGGCGATGGCCACCACCTGGTCCGGGGTCAGGCCATGGTCCTGGCACAGCACCGGCAAC

AGCCGCTGCACCGTCTCCAGCGCCTGCTTGCCGCCAATATTGCTGGCGATGGCCACCACCTGGTCCAGGGTCAGGCCATG

GTCCTGGCACAGCACCGGCAACAGCCGCTGCACCGTCTCCAGCGCCTGCTTGCCGCCATCGTGGCTGGCGATGGCCACCA

CCTGGTCCGGGGTCAGGCCATGGTCCTGGCACAGCACCGGCAACAGCCGCTGCACCGTCTCCAGCGCCTGCTTGCCGCCA

TCGTGGCTGGCGATGGCCACGACCTGGTCCGGGGTCAGGCCATGGTCCTGGCACAGCACCGGCAACAGCCGCTGCACTGT

CTCCAGCGCCTGCTTGCCGCCGCCATGGCTGGCGATGGCCACCACCTGGTCCGGGGTCAGGCCATGGTCCTGGCACAGCA

CCGGCAACAGCCGCTGCA

>M13F

GGATCCCATTCGTTCGCGCACGCCAAGTCCTGCCCGCGAGCTTCTGCCCGGACCCCAACCGGATAGGGTTCAGCCGACTG

CAGATCGGGGGGGGGCTCCGCCTGCTGGCGGCCCCCTGGATGGCTTGCCCGCTCGGCGGACGATGTCCCGGACCCGGCTG

CCATCTCCCCCTGCGCCCTCGCCTGCGTTCTCGGCGGGCAGCTTCAGCGATCTGCTCCGTCAGTTCGATCCGTCGCTTCT

TGATACATCGCTTCTTGATTCGATTCCTGCCGTCGGCACGCCGCATACAGCGGCTGCCCCAGCAGAGTGGGATGAGGTGC

AATCGGGTCTGCGTGCAGCCGATGACCCGCCACCCACCGTGCGTGTCGCTGTCACTGCCGCGCGGCCGCCGCGCGCTAAG

CCGGCCCCGCGACGGCGTGCGGCGCAACCCTCCGACGCTTCGCCGGCCGCGCAGGTGGATCTACGCACGCTCGGCTACAG

TCAGCAGCAGCAAGAGAAGATCAAACCGAAGGTGCGTTCGACAGTGGCGCAGCACCACGAGGCACTGGTGGGCCATGGGT

TTACACACGCGCACATCGTTGCGCTCAGCCAACACCCGGCAGCGTTAGGGACCGTTGCTGTCACGTATCAGGACATAATC

AGGGCGTTGCCAGAGGCGACACACGAAGACATCGTTGGCGTCGGCAAACAGTGGTCCGGCGCACGCGCCCTGGAGGCCTT

GCTCACGAAGGCGGGGGAGTTGAGAGGTCCGCCGTTACAGTTGGACACAGGCCAACTTCTCAAGATTGCAAAACGTGGCG

GCGTGACCGCAGTGGAGGCAGTGCATGCATGGCGCAATGCACTGACGGGTGCCCCCCTGAACCTGACCCCGGACCAAGTG

GTGGCCATCGCCAGCAATATTGGCGGCAACCAGGCGCTGGAGACGGTGCAGCGGCTGTTGCCGGTGCTGTGCCAGGCCCA

TGGCCTGA

>M13R

GCGCTGGAGACGGTGCAGCGGCTGTTGCCGGTGCTGTGCCAGGACCATGGCCTGACCCCGGACCAGGTGGTGGCCATCGC

CAACAATAACGGCGGCAAGCAGGCGCTGGAGACGGTGCAGCGGCTGTTGCCGGTGCTGTGCCAGGACCATGGCCTGACCC

CGGACCAGGTGGTGGCCATCGCCAGCAATGGCGGCGGCAAGCAGGCGCTGGAGAGCATTGTTGCCCAGTTATCTTGCCCT

GATCCGGCGTTGGCCGCGTTGACCAACGACCACCTCGTCGCCTTGGCCTGCCTCGGCGGACGTCCTGCCCTGGATGCAGT

GAAAAAGGGATTGCCGCACGCGCCGGAATTGATCAGAAGAATCAATCGCCGCATTCCCGAACGCACGTCCCATCGCGTTG

CCGACCTCCCCGAACGCACGTCCAATCGCGTTGCCGACCTCGCGCACGTGGTGCGCGTGCTTGGTTTTTTCCAGAGCCAC

TCCCACCCAGCGCAAGCATTCGATGACGCCATGACGCAGTTCGGGATGAGCAGGCACGGGTTGGTACAGCTCTTTCGCAG

AGTGGGCGTCACCGAACTCGAAGCCCGCTGCGGAACGCTCCCCCCAGCCTCGCAGCGTTGGGACCGTATCCTCCAGGCAT

CAGGGATGAAAAGGGCCAAACCGTCCCCTACTTCAGCTCAAACACCGGATCAGGCGTCTTTGCATGCATTCGCCGATTCG

CTGGAGCGTGACCTTGATGCGCCCAGCCCAATGCACGAGGGAGATCAGACGCGGGCAAGCAGCCGTAAACGGTCCCGATC

GGATCGTGCTGTCACCGGCCCCTCCGCACAGCAATCTTTCGAGGTACGCGTTCCCGAACAGCGCGATGCGCTGCATTTGC

CCCTCAGCTGGAGGGTAAAACGCCCGCGTACCAGGATCGGGGGCGGCCTCCCGGATCC

**pTAL*_Bam_*_HI_-39**

> pCC2FOS-MscI-1

ACCAGGTGGTGGCCATCGCCAACAATAACGGCGGCAAGCAGGCGCTGGAGACGGTGCAGCGGCTGTTGCCGGTGCTGTGC

CAGGACCATGGCCTGACCCCGGACCAAGTCGTGGCCATCGCCAGCCACGATGGCGGCAAGCAGGCGCTGGAGACGGTGCA

GCGGCTGTTGCCGGTGCTGTGCCAGGACCATGGCCTGACCCCGGACCAGGTCGTGGCCATCGCCAGCCACGATGGCGGCA

AGCAGGCGCTGGAGACGGTGCAGCGGCTGTTGCCGGTGCTGTGCCAGGAACATGGCCTGACCCCGGCCCAAGTGGTGGCC

ATCGCCAGCCACGATGGCGGCAAGCAGGCGCTGGAGACGGTGCAGCGGCTGTTGCCGGTGCTGTGCCAGGACCATGGCCT

GACCCCGGACCAGGTGGTGGCCATCGCCAGCAATGGCGGCGGCAAGCAGGCGCTGGCGACGGTGCAGCGGCTGTTGCCGG

TGCTGTGCCAGGCCCATGGCCTGACCCCGGACCAGGTCGTGGCCATCGCCAGCCACGATGGCGGCAAGCAGGCGCTGGAG

ACGGTGCAGCGGCTGTTGCCGGTGCTGTGCCAGGCCAATGGCCTGACCCCGGACCAGGTCGTGGCCATCGCCAGCAATGG

CGGCAAGCAGGCGCTGGAGACGGTGCAGCGGCTGTTGCCTGTACAGCGGCTGTTGCCGGTGCTGTGCCAGGACCATGGCC

TGACCCAGGACCAGGTGGTGGCCATCGCCAGCAATATTGGCGGCAAGCAGGCGCTGGAGACGGTGCAGCGGCTGTTGCCG

GTGCTGTGCCAGGC

> pCC2FOS-MscI-2

GGCAAGCAGGCGCTGGAGACGGTACAGCGGCTGTTGCCGGTGCTGTGCCAGGCCAATGGCCTGACCCCGGACCAGGTCGT

GGCCATCGCCAGCCATGGCGGCGGCAAGCAGGCGCTGGAGACGGTGCAGCGCCTGTTGCCGGTGCTGTGCCAGGACCATG

GCCTGACCCCGGACCAGGTCGTGGCCATCGCCAGCAATATTGGCGGCAAGCAGGCGCTGGAGACGGTGCAGCGGCTGTTG

CCGGTGCTGTGCCAGGACCATGGCCTGACCCCGGACCAGGTCGTGGCCATCGCCAGCCATGGCGGCGGCAAGCAGGCGCT

GGAGACGGTGCAGCGGCTGTTGCCGGTGCTGTGCCAGGACCATGGCCTGACCCCGGACCAGGTGGTGGCCATCGCCAGCA

ATATTGGCGGCAAGCAGGCGCTAGAGACGGTGCAGCGGCTGTTGCCGGTGCTGTGCCAGGCCCATGGCCTGACCCCGGAC

CAGGTGGTGGCCATCGCCAGCAATATTGGCGGCAAGCAGGCGCTGGAGACGGTGCAGCGGCTGTTGCCGGTGCTGTGCCA

GGACCATGGCCTGACCCCGGCCCAGGTGGTGGCCATCGCCAGCAATATTGGCGGCAAGCAGGCGCTGGAGACGGTGCAGC

GGCTGTTGCCGGTGCTGTGCCAGGACCATGGCCTGACCCCGGACCAAGTCGTGGCCATCGCCAGCCACGATGGCGGCAAG

CAGGCGCTGGAGACGGTGCAGCGGCTGTTGCCGGTGCTGTGCCAGGACCATGGCCTGACCCCGGACCAGGTGGTGGCCAT

CGCCAACAATAACGGCGGCAAGCAGGCGCTGGAGACGGTGCAGCGGCTGTTGCCGGTGCTGTGCCAGGACCATGGCCTGA

CCCCGGACCAAGTCGTGGCCATCGCCAGCCACGATGGCGGCAAGCAGGCGCTGGAGACGGTGCAGCGGCTGTTGCCGGTG

CTGTGCCAGGACCATGGCCTGA

> pCC2FOS-MscI-3

CCATCGCCAACAATAACGGCGGCAAGCAGGCGCTGGAGACGGTGCAGCGGCTGTTGCCGGTGCTGTGCCAGGACCATGGC

CTGACCCCGGACCAAGTCGTGGCCATCGCCAGCCACGATGGCGGCAAGCAGGCGCTGGAGACGGTGCAGCGGCTGTTGCC

GGTGCTGTGCCAGGACCATGGCCTGACCCCGGACCAGGTCGTGGCCATCGCCAGCCACGATGGCGGCAAGCAGGCGCTGG

AGACGGTGCAGCGGCTGTTGCCGGTGCTGTGCCAGGAACATGGCCTGACCCCGGCCCAAGTGGTGGCCATCGCCAGCCAC

GATGGCGGCAAGCAGGCGCTGGAGACGGTGCAGCGGCTGTTGCCGGTGCTGTGCCAGGACCATGGCCTGACCCCGGACCA

GGTGGTGGCCATCGCCAGCAATGGCGGCGGCAAGCAGGCGCTGGCGACGGTGCAGCGGCTGTTGCCGGTGCTGTGCCAGG

CCCATGGCCTGACCCCGGACCAGGTCGTGGCCATCGCCAGCCACGATGGCGGCAAGCAGGCGCTGGAGACGGTGCAGCGG

CTGTTGCCGGTGCTGTGCCAGGCCAATGGCCTGACCCCGGACCAGGTCGTGGCCATCGCCAGCAATGGCGGCAAGCAGGC

GCTGGAGACGGTGCAGCGGCTGTTGCCTGTACAGCGGCTGTTGCCGGTGCTGTGCCAGGACCATGGCCTGACCCAGGACC

AGGTGGTGGCCATCGCCAGCAATATTGGCGGCAAGCAGGCGCTGGAGACGGTGCAGCGGCTGTTGCCGGTGCTGTGCCAG

GCCAATGGCCTGACCCAGGACCAGGTGGTGGCCATCGCCAGCCACGATGGCGGCAAGCAGGCGCTGGAGACGGTGCAGCG

GCTGTTGCCGGTGCTGTGCCAGGACCA

> pCC2FOS-MscI-4

CGCTGGAGACGGTACAGCGGCTGTTGCCGGTGCTGTGCCAGGCCAATGGCCTGACCCCGGACCAGGTCGTGGCCATCGCC

AGCCATGGCGGCGGCAAGCAGGCGCTGGAGACGGTGCAGCGCCTGTTGCCGGTGCTGTGCCAGGACCATGGCCTGACCCC

GGACCAGGTCGTGGCCATCGCCAGCAATATTGGCGGCAAGCAGGCGCTGGAGACGGTGCAGCGGCTGTTGCCGGTGCTGT

GCCAGGACCATGGCCTGACCCCGGACCAGGTCGTGGCCATCGCCAGCCATGGCGGCGGCAAGCAGGCGCTGGAGACGGTG

CAGCGGCTGTTGCCGGTGCTGTGCCAGGACCATGGCCTGACCCCGGACCAGGTGGTGGCCATCGCCAGCAATATTGGCGG

CAAGCAGGCGCTAGAGACGGTGCAGCGGCTGTTGCCGGTGCTGTGCCAGGCCCATGGCCTGACCCCGGACCAGGTGGTGG

CCATCGCCAGCAATATTGGCGGCAAGCAGGCGCTGGAGACGGTGCAGCGGCTGTTGCCGGTGCTGTGCCAGGACCATGGC

CTGACCCCGGCCCAGGTGGTGGCCATCGCCAGCAATATTGGCGGCAAGCAGGCGCTGGAGACGGTGCAGCGGCTGTTGCC

GGTGCTGTGCCAGGACCATGGCCTGACCCCGGACCAAGTCGTGGCCATCGCCAGCCACGATGGCGGCAAGCAGGCGCTGG

AGACGGTGCAGCGGCTGTTGCCGGTGCTGTGCCAGGACCATGGCCTGACCCCGGACCAGGTGGTGGCCATCGCCAACAAT

AACGGCGGCAAGCAG

> pCC2FOS-MscI-5

CCATCGCCAGCCACGATGGCGGCAAGCAGGCGCTGGAGACGGTGCAGCGGCTGTTGCCGGTGCTGTGCCAGGACCATGGC

CTGACCCCGGACCAGGTGGTGGCCATCGCCAACAATAACGGCGGCAAGCAGGCGCTGGAGACGGTGCAGCGGCTGTTGCC

GGTGCTGTGCCAGGACCATGGCCTGACCCCGGACCAAGTCGTGGCCATCGCCAGCCACGATGGCGGCAAGCAGGCGCTGG

AGACGGTGCAGCGGCTGTTGCCGGTGCTGTGCCAGGACCATGGCCTGACCCCGGACCAGGTCGTGGCCATCGCCAGCCAC

GATGGCGGCAAGCAGGCGCTGGAGACGGTGCAGCGGCTGTTGCCGGTGCTGTGCCAGGAACATGGCCTGACCCCGGCCCA

AGTGGTGGCCATCGCCAGCCACGATGGCGGCAAGCAGGCGCTGGAGACGGTGCAGCGGCTGTTGCCGGTGCTGTGCCAGG

ACCATGGCCTGACCCCGGACCAGGTGGTGGCCATCGCCAGCAATGGCGGCGGCAAGCAGGCGCTGGCGACGGTGCAGCGG

CTGTTGCCGGTGCTGTGCCAGGCCCATGGCCTGACCCCGGACCAGGTCGTGGCCATCGCCAGCCACGATGGCGGCAAGCA

GGCGCTGGAGACGGTGCAGCGGCTGTTGCCGGTGCTGTGCCAGGCCAATGGCCTGACCCCGGACCAGGTCGTGGCCATCG

CCAGCAATGGCGGCAAGCAGGCGCTGGAGACGGTGCAGCGGCTGTTGCCTGTACAGCGGCTGTTGCCGGTGCTGTGCCAG

G

> pCC2FOS-MscI-6

CCATCGCCAGCAATATTGGCGGCAAGCAGGCGCTGGAGACGGTACAGCGGCTGTTGCCGGTGCTGTGCCAGGCCAATGGC

CTGACCCCGGACCAGGTCGTGGCCATCGCCAGCCATGGCGGCGGCAAGCAGGCGCTGGAGACGGTGCAGCGCCTGTTGCC

GGTGCTGTGCCAGGACCATGGCCTGACCCCGGACCAGGTCGTGGCCATCGCCAGCAATATTGGCGGCAAGCAGGCGCTGG

AGACGGTGCAGCGGCTGTTGCCGGTGCTGTGCCAGGACCATGGCCTGACCCCGGACCAGGTCGTGGCCATCGCCAGCCAT

GGCGGCGGCAAGCAGGCGCTGGAGACGGTGCAGCGGCTGTTGCCGGTGCTGTGCCAGGACCATGGCCTGACCCCGGACCA

GGTGGTGGCCATCGCCAGCAATATTGGCGGCAAGCAGGCGCTAGAGACGGTGCAGCGGCTGTTGCCGGTGCTGTGCCAGG

CCCATGGCCTGACCCCGGACCAGGTGGTGGCCATCGCCAGCAATATTGGCGGCAAGCAGGCGCTGGAGACGGTGCAGCGG

CTGTTGCCGGTGCTGTGCCAGGACCATGGCCTGACCCCGGCCCAGGTGGTGGCCATCGCCAGCAATATTGGCGGCAAGCA

GGCGCTGGAGACGGTGCAGCGGCTGTTGCCGGTGCTGTGCCAGGACCATGGCCTGACCCCGGACCAAGTCGTGGCCATCG

CCAGCCACGATGGCGGCAAGCAGGCGCTGGAGACGGTGCAGCGGCTGTTGCCGGTGCTGTGCCAGGACCATGGCCTGACC

CCGGACCAGGTGGTGGCCATCGCCAACAATAACGGCGGCAAGCAGGCGCTGGAGACGGTGCAGCGGCTGTTGCCGGTGCT

GTGCCAGG

> pCC2FOS-MscI-7

CCATCGCCAGCCACGATGGCGGCAAGCAGGCGCTGGAGACGGTGCAGCGGCTGTTGCCGGTGCTGTGCCAGGACCATGGC

CTGACCCCGGACCAGGTGGTGGCCATCGCCAACAATAACGGCGGCAAGCAGGCGCTGGAGACGGTGCAGCGGCTGTTGCC

GGTGCTGTGCCAGGACCATGGCCTGACCCCGGACCAAGTCGTGGCCATCGCCAGCCACGATGGCGGCAAGCAGGCGCTGG

AGACGGTGCAGCGGCTGTTGCCGGTGCTGTGCCAGGACCATGGCCTGACCCCGGACCAGGTCGTGGCCATCGCCAGCCAC

GATGGCGGCAAGCAGGCGCTGGAGACGGTGCAGCGGCTGTTGCCGGTGCTGTGCCAGGAACATGGCCTGACCCCGGCCCA

AGTGGTGGCCATCGCCAGCCACGATGGCGGCAAGCAGGCGCTGGAGACGGTGCAGCGGCTGTTGCCGGTGCTGTGCCAGG

ACCATGGCCTGACCCCGGACCAGGTGGTGGCCATCGCCAGCAATGGCGGCGGCAAGCAGGCGCTGGCGACGGTGCAGCGG

CTGTTGCCGGTGCTGTGCCAGGCCCATGGCCTGACCCCGGACCAGGTCGTGGCCATCGCCAGCCACGATGGCGGCAAGCA

GGCGCTGGAGACGGTGCAGCGGCTGTTGCCGGTGCTGTGCCAGGCCAATGGCCTGACCCCGGACCAGGTCGTGGCCATCG

CCAGCAATGGCGGCAAGCAGGCGCTGGAGACGGTGCAGCGGCTGTTGCCTGTACAGCGGCTGTTGCCGGTGCTGTGCCAG

G

> pCC2FOS-MscI-8

CCATCGCCAGCAATGGCGGCAAGCAGGCGCTGGAGACGGTGCAGCGGCTGTTGCCTGTACAGCGGCTGTTGCCGGTGCTG

TGCCAGGACCATGGCCTGACCCAGGACCAGGTGGTGGCCATCGCCAGCAATATTGGCGGCAAGCAGGCGCTGGAGACGGT

GCAGCGGCTGTTGCCGGTGCTGTGCCAGGCCAATGGCCTGACCCAGGACCAGGTGGTGGCCATCGCCAGCCACGATGGCG

GCAAGCAGGCGCTGGAGACGGTGCAGCGGCTGTTGCCGGTGCTGTGCCAGGACCATGGCCTGACCCCGGACCAGGTCGTG

GCCATCGCCAGCCACGATGGCGGCAAACAGGCGCTGGAGACGGTGCAGCGGCTGTTGCCGGTGCTGTGCCAGGACCATGG

CCTGACCCCGGCCCAGGTGGTGGCCATCGCCAACAATAACGGCGGCAAGCAGGCGCTGGAGACGGTGCAGCGGCTGTTGC

CGGTGCTGTGCCAGGACCATGGCCTGACCCCGGACCAGGTCGTGGCCATCGCCAGCAATATTGGCGGCAAGCAGGCGCTG

GCGACGGTGCAGCGGCTGTTGCCGGTGCTGTGCCAGGACCATGGCCTGACCCCGGACCAGGTCGTGGCCATCGCCAACAA

TAACGGCGGCAAGCAGGCGCTGGAGACGGTGCAGCGGCTGTTGCCGGTGCTGTGCCAGGACCATGGCCTGACCCCGGCGC

AGGTGGTGGCCATCGCCAGCAATAACGGCGGC

> pCC2FOS-MscI-9

CATGGCCTGACCCCGGACCAGGTGGTGGCCATCGCCAGCAATATTGGCGGCAAGCAGGCGCTGGAGACGGTGCAGCGGCT

GTTGCCGGTGCTGTGCCAGGACCATGGCCTGACCCCGGCCCAGGTGGTGGCCATCGCCAGCAATATTGGCGGCAAGCAGG

CGCTGGAGACGGTGCAGCGGCTGTTGCCGGTGCTGTGCCAGGACCATGGCCTGACCCCGGACCAAGTCGTGGCCATCGCC

AGCCACGATGGCGGCAAGCAGGCGCTGGAGACGGTGCAGCGGCTGTTGCCGGTGCTGTGCCAGGACCATGGCCTGACCCC

GGACCAGGTGGTGGCCATCGCCAACAATAACGGCGGCAAGCAGGCGCTGGAGACGGTGCAGCGGCTGTTGCCGGTGCTGT

GCCAGGACCATGGCCTGACCCCGGACCAAGTCGTGGCCATCGCCAGCCACGATGGCGGCAAGCAGGCGCTGGAGACGGTG

CAGCGGCTGTTGCCGGTGCTGTGCCAGGACCATGGCCTGACCCCGGACCAGGTCGTGGCCATCGCCAGCCACGATGGCGG

CAAGCAGGCGCTGGAGACGGTGCAGCGGCTGTTGCCGGTGCTGTGCCAGGAACATGGCCTGACCCCGGCCCAAGTGGTGG

CCATCGCCAGCCACGATGGCGGCAAGCAGGCGCTGGAGACGGTGCAGCGGCTGTTGCCGGTGCTGTGCCAGGACCA

> pCC2FOS-MscI-10

AATAACGGCGGCAAGCAGGCGCTGGAGACGGTGCAGCGGCTGTTGCCGGTGCTGTGCCAGGACCATGGCCTGACCCCGGA

CCAGGTCGTGGCCATCGCCAGCAATATTGGCGGCAAGCAGGCGCTGGCGACGGTGCAGCGGCTGTTGCCGGTGCTGTGCC

AGGACCATGGCCTGACCCCGGACCAGGTCGTGGCCATCGCCAACAATAACGGCGGCAAGCAGGCGCTGGAGACGGTGCAG

CGGCTGTTGCCGGTGCTGTGCCAGGACCATGGCCTGACCCCGGCGCAGGTGGTGGCCATCGCCAGCAATAACGGCGGCAA

GCAGGCGCTGGAGACGGTGCAGCGGCTGTTGCCGGTGCTGTGCCAGGACCATGGCCTGACCCTGGACCAGGTGGTGGCCA

TTGCCAGCAATGGCGGCAGCAAACAGGCGCTGGAGACGGTGCAGCGGCTGTTGCCGGTGCTGTGCCAGGACCATGGCCTG

ACCCCGGACCAAGTGGTGGCCATCGCCAACAATAACGGCGGCAAGCAGGCGCTGGAGACGGTGCAGCGGCTGTTGCCGGT

GCTGTGCCAGGACCATGGCCTGACCCTGGACCAGGTGGTGGCCATCGCCAGCCACGATGGCGGCAAGCAGGCGCTGGAGA

CGGTGCAGCGGCTGTTGCCGGTGCTGTGCCAGGACCATGGCCTGACCCTGGACAAGGTGGTGGCCATCGCCAGCAATGGC

GGCAAGCAGGCGCTGGAGACGGTGCAGCGGCTGTTGCCGGTGCTGTGCCAGGACCATGGCCTGACCCCGAACCAGGTGGT

GGCCATCGCCAGCAATAGTGGCGGCAAGCAGGCGCTGGAGACGGTGCAGCGGCTGTTGCCGGTG

> pCC2FOS-MscI-11

GTACAGCGGCTGTTGCCGGTGCTGTGCCAGGCCAATGGCCTGACCCCGGACCAGGTCGTGGCCATCGCCAGCCATGGCGG

CGGCAAGCAGGCGCTGGAGACGGTGCAGCGCCTGTTGCCGGTGCTGTGCCAGGACCATGGCCTGACCCCGGACCAGGTCG

TGGCCATCGCCAGCAATATTGGCGGCAAGCAGGCGCTGGAGACGGTGCAGCGGCTGTTGCCGGTGCTGTGCCAGGACCAT

GGCCTGACCCCGGACCAGGTCGTGGCCATCGCCAGCCATGGCGGCGGCAAGCAGGCGCTGGAGACGGTGCAGCGGCTGTT

GCCGGTGCTGTGCCAGGACCATGGCCTGACCCCGGACCAGGTGGTGGCCATCGCCAGCAATATTGGCGGCAAGCAGGCGC

TAGAGACGGTGCAGCGGCTGTTGCCGGTGCTGTGCCAGGCCCATGGCCTGACCCCGGACCAGGTGGTGGCCATCGCCAGC

AATATTGGCGGCAAGCAGGCGCTGGAGACGGTGCAGCGGCTGTTGCCGGTGCTGTGCCAGGACCATGGCCTGACCCCGGC

CCAGGTGGTGGCCATCGCCAGCAATATTGGCGGCAAGCAGGCGCTGGAGACGGTGCAGCGGCTGTTGCCGGTGCTGTGCC

AGGACCATGGCCTGACCCCGGACCAAGTCGTGGCCATCGCCAGCCACGATGGCGGCAAGCAGGCGCTGGAGACGGTGCAG

CGGCTGTTGCCGGTGCTGTGCCAGGACCATGGCCTGACCCCGGACCAGGTGGTGGCCATCGCCAACAATAACGGCGGCAA

GCAGGCGCTGGAGACGGTGCAGCGGCTGTTGCCGGTGCTGTGCCAGGACCATGGCCTGACCCCGGACCAAGTCGTGGCCA

TCGCCAGCCACGATGGCGGCAAGCAGGCGCTGGAGACGGTGCAGCGGCTGTTGCCGGTGCTGTGCCAGGACCATGGCCTG

ACC

> pCC2FOS-MscI-12

CATGGTCCTGGCACAGCACCGGCAACAGCCGCTGCACCGTCTCCAGCGCCTGCTTGCCGCCACTATTGCTGGCGATGGCC

ACCACCTGGTTCGGGGTCAGGCCATGGTCCTGGCACAGCACCGGCAACAGCCGCTGCACCGTCTCCAGCGCCTGCTTGCC

GCCATTGCTGGCGATGGCCACCACCTTGTCCAGGGTCAGGCCATGGTCCTGGCACAGCACCGGCAACAGCCGCTGCACCG

TCTCCAGCGCCTGCTTGCCGCCATCGTGGCTGGCGATGGCCACCACCTGGTCCAGGGTCAGGCCATGGTCCTGGCACAGC

ACCGGCAACAGCCGCTGCACCGTCTCCAGCGCCTGCTTGCCGCCGTTATTGTTGGCGATGGCCACCACTTGGTCCGGGGT

CAGGCCATGGTCCTGGCACAGCACCGGCAACAGCCGCTGCACCGTCTCCAGCGCCTGTTTGCTGCCGCCATTGCTGGCAA

TGGCCACCACCTGGTCCAGGGTCAGGCCATGGTCCTGGCACAGCACCGGCAACAGCCGCTGCACCGTCTCCAGCGCCTGC

TTGCCGCCGTTATTGCTGGCGATGGCCACCACCTGCGCCGGGGTCAGGCCATGGTCCTGGCACAGCACCGGCAACAGCCG

CTGCACCGTCTCCAGCGCCTGCTTGCCGCCGTTATTGTTGGCGATGGCCACGACCTGGTCCGGGGTCAGGCCATGGTCCT

GGCACAGCACCGGCAACAGCCGCTGCACCGTCGCCAGCGCCTGCTTGCCGCCAATATTGCTGGCGATGGCCACGACCTGG

TCCGGGGTCAGGCCATGGTCCTGGCACAGCACCGGCAACAGCCGCTGCACCGTCTCCAGCGCCTGCTTGCCGCCGTTATT

GTTGGCGATGGCCACCACCTGGGCCGGGGTCAGGCCATGGTCCTGGCACAGCACCGGCAACAGCCGCTGCACCGTCTCCA

GCGCCTGTTTGCCGCCATC

> pCC2FOS-MscI-13

TGGCGATGGCCACCACTTGGTCCGGGGTCAGGCCATGGTCCTGGCACAGCACCGGCAACAGCCGCTGCACCGTCTCCAGC

GCCTGTTTGCTGCCGCCATTGCTGGCAATGGCCACCACCTGGTCCAGGGTCAGGCCATGGTCCTGGCACAGCACCGGCAA

CAGCCGCTGCACCGTCTCCAGCGCCTGCTTGCCGCCGTTATTGCTGGCGATGGCCACCACCTGCGCCGGGGTCAGGCCAT

GGTCCTGGCACAGCACCGGCAACAGCCGCTGCACCGTCTCCAGCGCCTGCTTGCCGCCGTTATTGTTGGCGATGGCCACG

ACCTGGTCCGGGGTCAGGCCATGGTCCTGGCACAGCACCGGCAACAGCCGCTGCACCGTCGCCAGCGCCTGCTTGCCGCC

AATATTGCTGGCGATGGCCACGACCTGGTCCGGGGTCAGGCCATGGTCCTGGCACAGCACCGGCAACAGCCGCTGCACCG

TCTCCAGCGCCTGCTTGCCGCCGTTATTGTTGGCGATGGCCACCACCTGGGCCGGGGTCAGGCCATGGTCCTGGCACAGC

ACCGGCAACAGCCGCTGCACCGTCTCCAGCGCCTGTTTGCCGCCATCGTGGCTGGCGATGGCCACGACCTGGTCCGGGGT

CAGGCCATGGTCCTGGCACAGCACCGGCAACAGCCGCTGCACCGTCTCCAGCGCCTGCTTGCCGCCATCGTGGCTGGCGA

TGGCCACCACCTGGTCCTGGGTCAGGCCATTGGCCTGGCACAGCACCGGCAACAGCCGCTGCACCGTCTCCAGCGCCTGC

TTGCCGCCAATATTGCTGGCGATGGCCACCACCTGGTCCTGGGTCAGGCCATGGTCCTGGCACAGCACCGGCAACAGCCG

CTGTACAGGCAACAGCCGCTGCACCGTCTCCAG

> pCC2FOS-MscI-14

CTGGCACAGCACCGGCAACAGCCGCTGCACCGTCTCCAGCGCCTGCTTGCCGCCGTTATTGCTGGCGATGGCCACCACCT

GCGCCGGGGTCAGGCCATGGTCCTGGCACAGCACCGGCAACAGCCGCTGCACCGTCTCCAGCGCCTGCTTGCCGCCGTTA

TTGTTGGCGATGGCCACGACCTGGTCCGGGGTCAGGCCATGGTCCTGGCACAGCACCGGCAACAGCCGCTGCACCGTCGC

CAGCGCCTGCTTGCCGCCAATATTGCTGGCGATGGCCACGACCTGGTCCGGGGTCAGGCCATGGTCCTGGCACAGCACCG

GCAACAGCCGCTGCACCGTCTCCAGCGCCTGCTTGCCGCCGTTATTGTTGGCGATGGCCACCACCTGGGCCGGGGTCAGG

CCATGGTCCTGGCACAGCACCGGCAACAGCCGCTGCACCGTCTCCAGCGCCTGTTTGCCGCCATCGTGGCTGGCGATGGC

CACGACCTGGTCCGGGGTCAGGCCATGGTCCTGGCACAGCACCGGCAACAGCCGCTGCACCGTCTCCAGCGCCTGCTTGC

CGCCATCGTGGCTGGCGATGGCCACCACCTGGTCCTGGGTCAGGCCATTGGCCTGGCACAGCACCGGCAACAGCCGCTGC

ACCGTCTCCAGCGCCTGCTTGCCGCCAATATTGCTGGCGATGGCCACCACCTGGTCCTGGGTCAGGCCATGGTCCTGGCA

CAGCACCGGCAACAGCCGCTGTACAGGCAACAGCCGCTGCACCGTCTCCAGCGCCTGCTTGCCGCCATTGCTGGCGATGG

CCACGACCTGGTCCGGGGTCAGGCCATTGGCCTGG

> pCC2FOS-MscI-15

GCACCGGCAACAGCCGCTGCACCGTCTCCAGCGCCTGCTTGCCGCCATCGTGGCTGGCGATGGCCACGACTTGGTCCGGG

GTCAGGCCATGGTCCTGGCACAGCACCGGCAACAGCCGCTGCACCGTCTCCAGCGCCTGCTTGCCGCCGTTATTGTTGGC

GATGGCCACCACCTGGTCCGGGGTCAGGCCATGGTCCTGGCACAGCACCGGCAACAGCCGCTGCACCGTCTCCAGCGCCT

GCTTGCCGCCATCGTGGCTGGCGATGGCCACGACTTGGTCCGGGGTCAGGCCATGGTCCTGGCACAGCACCGGCAACAGC

CGCTGCACCGTCTCCAGCGCCTGCTTGCCGCCAATATTGCTGGCGATGGCCACCACCTGGGCCGGGGTCAGGCCATGGTC

CTGGCACAGCACCGGCAACAGCCGCTGCACCGTCTCCAGCGCCTGCTTGCCGCCAATATTGCTGGCGATGGCCACCACCT

GGTCCGGGGTCAGGCCATGGGCCTGGCACAGCACCGGCAACAGCCGCTGCACCGTCTCTAGCGCCTGCTTGCCGCCAATA

TTGCTGGCGATGGCCACCACCTGGTCCGGGGTCAGGCCATGGTCCTGGCACAGCACCGGCAACAGCCGCTGCACCGTCTC

CAGCGCCTGCTTGCCGCCGCCATGGCTGGCGATGGCCACGACCTGGTCCGGGGTCAGGCCATGGTCCTGGCACAGCACCG

GCAACAGCCGCTGCACCGTCTCCAGCGCCTGCTTGCCGCCAATATTGCTGGCGATGGCCACGACCTGGTCCGGGGTCAGG

CCATGGTCCTGGCACAGCACCGGCAACAGGCGCTGCACCGTCTCCAGCGCCTGCTTGCCGCCGCCATGGCTGGCGATGGC

CACGACCTGGTCCGGGGTCAGGCCATTGGCCTGG

> pCC2FOS-MscI-16

CTGGCACAGCACCGGCAACAGCCGCTGCACCGTCGCCAGCGCCTGCTTGCCGCCGCCATTGCTGGCGATGGCCACCACCT

GGTCCGGGGTCAGGCCATGGTCCTGGCACAGCACCGGCAACAGCCGCTGCACCGTCTCCAGCGCCTGCTTGCCGCCATCG

TGGCTGGCGATGGCCACCACTTGGGCCGGGGTCAGGCCATGTTCCTGGCACAGCACCGGCAACAGCCGCTGCACCGTCTC

CAGCGCCTGCTTGCCGCCATCGTGGCTGGCGATGGCCACGACCTGGTCCGGGGTCAGGCCATGGTCCTGGCACAGCACCG

GCAACAGCCGCTGCACCGTCTCCAGCGCCTGCTTGCCGCCATCGTGGCTGGCGATGGCCACGACTTGGTCCGGGGTCAGG

CCATGGTCCTGGCACAGCACCGGCAACAGCCGCTGCACCGTCTCCAGCGCCTGCTTGCCGCCGTTATTGTTGGCGATGGC

CACCACCTGGTCCGGGGTCAGGCCATGGTCCTGGCACAGCACCGGCAACAGCCGCTGCACCGTCTCCAGCGCCTGCTTGC

CGCCATCGTGGCTGGCGATGGCCACGACTTGGTCCGGGGTCAGGCCATGGTCCTGGCACAGCACCGGCAACAGCCGCTGC

ACCGTCTCCAGCGCCTGCTTGCCGCCAATATTGCTGGCGATGGCCACCACCTGGGCCGGGGTCAGGCCATGGTCCTGGCA

CAGCACCGGCAACAGCCGCTGCACCGTCTCCAGCGCCTGCTTGCCGCCAATATTGCTGGCGATGGCCACCACCTGGTCCG

GGGTCAGGCCATGGGCCTGGCACAGCACCGGCAACAGCCGCTGCACCGTCTCTAGCGCCTGCTTGCCGCCAATATTGCTG

GCGATGGCCACCACCTGGTCCGGGGTCAGGCCATGGTCCTGGCACAGCACCGGCAACAGCCGCTGCACCGTCTCCAGCGC

CTGCTTGCCGCC

>39P1F_1

AGGCGGGGGAGTTGAGAGGTCCGCCGTTACAGTTGGACACAGGCCAACTTCTCAAGATTGCAAAACGTGGCGGCTTGACC

TCAGTGCAGGCAGTGCATGCATGGCGCAATGCACTGACGGGTGCCCCCCTGAACCTGACCCCGGACCAAGTGGTGGCCAT

CGCCAGCAATATTGGCGGCAAGCAGGCGCTGGAGACGGTACAGCGGCTGTTGCCGGTGCTGTGCCAGGCCAATGGCCTGA

CCCCGGACCAGGTCGTGGCCATCGCCAGCCATGGCGGCGGCAAGCAGGCGCTGGAGACGGTGCAGCGCCTGTTGCCGGTG

CTGTGCCAGGACCATGGCCTGACCCCGGACCAGGTCGTGGCCATCGCCAGCAATATTGGCGGCAAGCAGGCGCTGGAGAC

GGTGCAGCGGCTGTTGCCGGTGCTGTGCCAGGACCATGGCCTGACCCCGGACCAGGTCGTGGCCATCGCCAGCCATGGCG

GCGGCAAGCAGGCGCTGGAGACGGTGCAGCGGCTGTTGCCGGTGCTGTGCCAGGACCATGGCCTGACCCCGGACCAGGTG

GTGGCCATCGCCAGCAATATTGGCGGCAAGCAGGCGCTAGAGACGGTGCAGCGGCTGTTGCCGGTGCTGTGCCAGGCCCA

TGGCCTGACCCCGGACCAGGTGGTGGCCATCGCCAGCAATATTGGCGGCAAGCAGGCGCTGGAGACGGTGCAGCGGCTGT

TGCCGGTGCTGTGCCAGGACCATGGCCTGACCCCGGCCCAGGTGGTGGCCATCGCCAGCAATATTGGCGGCAAGCAGGCG

CTGGAGACGGTGCAGCGGCTGTTGCCGGTGCTGTGCCAGGACCATGGCCTGACCCCGGACCAAGTCGTGGCCATCGCCAG

CCACGATGGCGGCAAGCAGGCGCTGGAGACGGTGCAGCGGCTGTTGCCGGTGCTGTGCCAGGACCATGGCCTGACCCCGG

ACCAGGTGGTGGCCATCGCCAA

>39P1F_2

TTGCTCACGAAGGCGGGGGAGTTGAGAGGTCCGCCGTTACAGTTGGACACAGGCCAACTTCTCAAGATTGCAAAACGTGG

CGGCTTGACCTCAGTGCAGGCAGTGCATGCATGGCGCAATGCACTGACGGGTGCCCCCCTGAACCTGACCCCGGACCAAG

TGGTGGCCATCGCCAGCAATATTGGCGGCAAGCAGGCGCTGGAGACGGTACAGCGGCTGTTGCCGGTGCTGTGCCAGGCC

AATGGCCTGACCCCGGACCAGGTCGTGGCCATCGCCAGCCATGGCGGCGGCAAGCAGGCGCTGGAGACGGTGCAGCGCCT

GTTGCCGGTGCTGTGCCAGGACCATGGCCTGACCCCGGACCAGGTCGTGGCCATCGCCAGCAATATTGGCGGCAAGCAGG

CGCTGGAGACGGTGCAGCGGCTGTTGCCGGTGCTGTGCCAGGACCATGGCCTGACCCCGGACCAGGTCGTGGCCATCGCC

AGCCATGGCGGCGGCAAGCAGGCGCTGGAGACGGTGCAGCGGCTGTTGCCGGTGCTGTGCCAGGACCATGGCCTGACCCC

GGACCAGGTGGTGGCCATCGCCAGCAATATTGGCGGCAAGCAGGCGCTAGAGACGGTGCAGCGGCTGTTGCCGGTGCTGT

GCCAGGCCCATGGCCTGACCCCGGACCAGGTGGTGGCCATCGCCAGCAATATTGGCGGCAAGCAGGCGCTGGAGACGGTG

CAGCGGCTGTTGCCGGTGCTGTGCCAGGACCATGGCCTGACCCCGGCCCAGGTGGTGGCCATCGCCAGCAATATTGGCGG

CAAGCAGGCGCTGGAGACGGTGCAGCGGCTGTTGCCGGTGCTGTGCCAGGACCATGGCCTGACCCCGGACCAAGTCGTGG

CCATCGCCAGCCACGATGGCGGCAAGCAGGCGCTGGAGACGGTGCAGCGGCTGTTGCCGGTGCTGTGCCAGGACCATGGC

CTGACCCCGGACCAGGTGGTGGCCATCGCC

>39P1R_1

GGGCAACAATGCTCTCCAGCGCCTGCTTGCCGCCATTGCTGGCGATGGCCACCACCTGGTTCGGGGTCAGGCCATGGTCC

TGGCACAGCACCGGCAACAGCCGCTGCACCGTCTCCAGCGCCTGCTTGCCGCCACTATTGCTGGCGATGGCCACCACCTG

GTTCGGGGTCAGGCCATGGTCCTGGCACAGCACCGGCAACAGCCGCTGCACCGTCTCCAGCGCCTGCTTGCCGCCATTGC

TGGCGATGGCCACCACCTTGTCCAGGGTCAGGCCATGGTCCTGGCACAGCACCGGCAACAGCCGCTGCACCGTCTCCAGC

GCCTGCTTGCCGCCATCGTGGCTGGCGATGGCCACCACCTGGTCCAGGGTCAGGCCATGGTCCTGGCACAGCACCGGCAA

CAGCCGCTGCACCGTCTCCAGCGCCTGCTTGCCGCCGTTATTGTTGGCGATGGCCACCACTTGGTCCGGGGTCAGGCCAT

GGTCCTGGCACAGCACCGGCAACAGCCGCTGCACCGTCTCCAGCGCCTGTTTGCTGCCGCCATTGCTGGCAATGGCCACC

ACCTGGTCCAGGGTCAGGCCATGGTCCTGGCACAGCACCGGCAACAGCCGCTGCACCGTCTCCAGCGCCTGCTTGCCGCC

GTTATTGCTGGCGATGGCCACCACCTGCGCCGGGGTCAGGCCATGGTCCTGGCACAGCACCGGCAACAGCCGCTGCACCG

TCTCCAGCGCCTGCTTGCCGCCGTTATTGTTGGCGATGGCCACGACCTGGTCCGGGGTCAGGCCATGGTCCTGGCACAGC

ACCGGCAACAGCCGCTGCACCGTCGCCAGCGCCTGCTTGCCGCCAATATTGCTGGCGATGGCCACGACCTGGTCCGGGGT

CAGGCCATGGTCCTGGCACAGCACCGGCAACAGCCGCTGCACCGTCTCCAGCGCCTGCTTGCCGCCGTTATTGTTGGCGA

TGGCCACCACCT

>39P1R_2

CCAGGTGGTGGCCATCGCCAACAATAACGGCGGCAAGCAGGCGCTGGAGACGGTGCAGCGGCTGTTGCCGGTGCTGTGCC

AGGACCATGGCCTGACCCCGGACCAGGTCGTGGCCATCGCCAGCAATATTGGCGGCAAGCAGGCGCTGGCGACGGTGCAG

CGGCTGTTGCCGGTGCTGTGCCAGGACCATGGCCTGACCCCGGACCAGGTCGTGGCCATCGCCAACAATAACGGCGGCAA

GCAGGCGCTGGAGACGGTGCAGCGGCTGTTGCCGGTGCTGTGCCAGGACCATGGCCTGACCCCGGCGCAGGTGGTGGCCA

TCGCCAGCAATAACGGCGGCAAGCAGGCGCTGGAGACGGTGCAGCGGCTGTTGCCGGTGCTGTGCCAGGACCATGGCCTG

ACCCTGGACCAGGTGGTGGCCATTGCCAGCAATGGCGGCAGCAAACAGGCGCTGGAGACGGTGCAGCGGCTGTTGCCGGT

GCTGTGCCAGGACCATGGCCTGACCCCGGACCAAGTGGTGGCCATCGCCAACAATAACGGCGGCAAGCAGGCGCTGGAGA

CGGTGCAGCGGCTGTTGCCGGTGCTGTGCCAGGACCATGGCCTGACCCTGGACCAGGTGGTGGCCATCGCCAGCCACGAT

GGCGGCAAGCAGGCGCTGGAGACGGTGCAGCGGCTGTTGCCGGTGCTGTGCCAGGACCATGGCCTGACCCTGGACAAGGT

GGTGGCCATCGCCAGCAATGGCGGCAAGCAGGCGCTGGAGACGGTGCAGCGGCTGTTGCCGGTGCTGTGCCAGGACCATG

GCCTGACCCCGAACCAGGTGGTGGCCATCGCCAGCAATAGTGGCGGCAAGCAGGCGCTGGAGACGGTGCAGCGGCTGTTG

CCGGTGCTGTGCCAGGACCATGGCCTGACCCCGAACCAGGTGGTGGCCATCGCCAGCAATGGCGGCAAGCAGGCGCTGGA

GAGCATTGTTGCCCAGTT

>39P2F

CGCGCACATCGTTGCGCTCAGCCAACACCCGGCAGCGTTAGGGACCGTCGCTGTCAAGTATCAGCACATAATCACGGCGT

TGCCAGAGGCGACACACGAAGACATCGTTGGCGTCGGCAAACAGTGGTCCGGCGCACGCGCCCTGGAGGCCTTGCTCACG

AAGGCGGGGGAGTTGAGAGGTCCGCCGTTACAGTTGGACACAGGCCAACTTCTCAAGATTGCAAAACGTGGCGGCTTGAC

CTCAGTGCAGGCAGTGCATGCATGGCGCAATGCACTGACGGGTGCCCCCCTGAACCTGACCCCGGACCAAGTGGTGGCCA

TCGCCAGCAATATTGGCGGCAAGCAGGCGCTGGAGACGGTACAGCGGCTGTTGCCGGTGCTGTGCCAGGCCAATGGCCTG

ACCCCGGACCAGGTCGTGGCCATCGCCAGCCATGGCGGCGGCAAGCAGGCGCTGGAGACGGTGCAGCGCCTGTTGCCGGT

GCTGTGCCAGGACCATGGCCTGACCCCGGACCAGGTCGTGGCCATCGCCAGCAATATTGGCGGCAAGCAGGCGCTGGAGA

CGGTGCAGCGGCTGTTGCCGGTGCTGTGCCAGGACCATGGCCTGACCCCGGACCAGGTCGTGGCCATCGCCAGCCATGGC

GGCGGCAAGCAGGCGCTGGAGACGGTGCAGCGGCTGTTGCCGGTGCTGTGCCAGGACCATGGCCTGACCCCGGACCAGGT

GGTGGCCATCGCCAGCAATATTGGCGGCAAGCAGGCGCTAGAGACGGTGCAGCGGCTGTTGCCGGTGCTGTGCCAGGCCC

ATGGCCTGACCCCGGACCAGGTGGTGGCCATCGCCAGCAATATTGGCGGCAAGCAGGCGCTGGAGACGGTGCAGCGGCTG

TTGCCGGTGCTGTGCCAGGACCATGGCCTGACCCCGGCCCAGGTGGTGGCCATCGCCAGCAATATTGGCGGCAAGCAGGC

GCTGGAGACGG

>39P2R

CGCCGTGATTATGTGCTGATACTTGACAGCGACGGTCCCTAACGCTGCCGGGTGTTGGCTGAGCGCAACGATGTGCGCGT

GTGTAAACCCATGGCCCACCAGTGCCTCGTGGTGCTGCGCCACTGTCGAACGCACCTTCGGTTTGATCTTCTCTTGCTGC

TGCTGACTGTAGCCGAGCGTGCGTAGATCCACCTGCGCGGCCGGCGAAGCGTCGGAGGGTTGTGCCGCACGCCGTCGCGG

GGCCGGCTTGGCGCGCGGCGGCCGCGCGGCAGTGACAGCGACACGCACGGTGGGTGGCGGGTCATCGGCTGCACGCAGAC

CCGATTGCACCTCATCCCACTCTGCTGGGGCAGCCGCTGTATGCGGCGTGCCGACGGCAGGCATCGAATCAAGAAGCGAT

GTATCAAGAAGCGACGGATCGAACTGACGGAGCAGATCGCTGAAGCTGCCCGCCGAGAACGCAGGCGAGGGCGCAGGGGG

AGATGGCAGCCGGGTCCGGGACATCGTCCGCCGAGCGGGCAAGCCATCCAGGGGGCCGCCAGCAGGCGGAGCCCCCCCCC

GATCTGCAGTCGGCTGAACCCTATCCGGTTGGGGTCCGGGCAGAAGCTCGCGGGCAGGACTTGGCGTGCGCGAACGAATG

GGATCC

>39P3F

GGGATTGCCGCACGCGCCGGAATTGATCAGAAGAATCAATCGCCGCATTCCCGAACGCACGTCCCATCGCGTTCCCGACC

TCGCGCACGTGGTTCGCGTGCTTGGTTTTTTCCAGAGCCACTCCCACCCAGCGCAAGCATTCGATGACGCCATGACGCAG

TTCGAGATGAGCAGGCACGGCTTGGTACAGCTCTTTCGCAGAGTGGGCGTCACCGAATTCGAAGCCCGCTACGGAACGCT

CCCCCCAGCCTCGCAGCGTTGGGACCGTATCCTCCAGGCATCAGGGATGAAAAGGGCCAAACCGTCCCCTACTTCAGCTC

AAACACCGGATCAGGCGTCTTTGCATGCATTCGCCGATTCGCTGGAGCGTGACCTTGATGCGCCTAGCCCAATGCACGAG

GGAGATCAGACAGGGGCAAGCAGCCGTAAACGGTCCCGATCGGATCGTGCTGTCACCGGCCCCTCCGCACAGCAATCTTT

CGAGGTGCGCGTTCCCGAACAGCGCGATGCGCTGCATTTGCCCCTCAGCTGGAGGGTAAAACGCCCGCGTACCAGGATCG

GGGGCGGCCTCCCGGATCC

>39P3R

TCGGTGACGCCCACTCTGCGAAAGAGCTGTACCAAGCCGTGCCTGCTCATCTCGAACTGCGTCATGGCGTCATCGAATGC

TTGCGCTGGGTGGGAGTGGCTCTGGAAAAAACCAAGCACGCGAACCACGTGCGCGAGGTCGGGAACGCGATGGGACGTGC

GTTCGGGAATGCGGCGATTGATTCTTCTGATCAATTCCGGCGCGTGCGGCAATCCCTTTTTCACTGCATCCAGGGCAGGA

CGTCCGCCGAGGCAGGCCAAGGCGACGAGGTGGTCGTTGGTCAACGCGGCCAACGCCGGATCAGGGCGAGATAACTGGGC

AACAATGCTCTCCAGCGCCTGCTTGCCGCCATTGCTGGCGATGGCCACCACCTGGTTCGGGGTCAGGCCATGGTCCTGGC

ACAGCACCGGCAACAGCCGCTGCACCGTCTCCAGCGCCTGCTTGCCGCCACTATTGCTGGCGATGGCCACCACCTGGTTC

GGGGTCAGGCCATGGTCCTGGCACAGCACCGGCAACAGCCGCTGCACCGTCTCCAGCGCCTGCTTGCCGCCATTGCTGGC

GATGGCCACCACCTTGTCCAGGGTCAGGCCATGGTCCTGGCACAGCACCGGCAACAGCCGCTGCACCGTCTCCAGCGCCT

GCTTGCCGCCATCGTGGCTGGCGATGGCCACCACCTGGTCCAGGGTCAGGCCATGGTCCTGGCACAGCACCGGCAACAGC

CGCTGCACCGTCTCCAGCGCCTGCTTGCCGCCGTTATTGTTGGCGATGGCCACCACTTGGTCCGGGGTCAGGCCATGGTC

CTGGCACAGCACCGGCAACAGCCGCTGCACCGTCTCCAGCGCCTGTTTGCTGCCGCCATTGCTGGCAATGGCCACCACCT

GGTCCAGGGTCAGGCCATGGTCCTGGCACAGCACCGGCAACAGCCGCTGCACCGTCTCCAGCGCCTGCTTGCCGCCGTTA

TTGCTGGCGATGGC

>M13F

GGATCCCATTCGTTCGCGCACGCCAAGTCCTGCCCGCGAGCTTCTGCCCGGACCCCAACCGGATAGGGTTCAGCCGACTG

CAGATCGGGGGGGGGCTCCGCCTGCTGGCGGCCCCCTGGATGGCTTGCCCGCTCGGCGGACGATGTCCCGGACCCGGCTG

CCATCTCCCCCTGCGCCCTCGCCTGCGTTCTCGGCGGGCAGCTTCAGCGATCTGCTCCGTCAGTTCGATCCGTCGCTTCT

TGATACATCGCTTCTTGATTCGATGCCTGCCGTCGGCACGCCGCATACAGCGGCTGCCCCAGCAGAGTGGGATGAGGTGC

AATCGGGTCTGCGTGCAGCCGATGACCCGCCACCCACCGTGCGTGTCGCTGTCACTGCCGCGCGGCCGCCGCGCGCCAAG

CCGGCCCCGCGACGGCGTGCGGCACAACCCTCCGACGCTTCGCCGGCCGCGCAGGTGGATCTACGCACGCTCGGCTACAG

TCAGCAGCAGCAAGAGAAGATCAAACCGAAGGTGCGTTCGACAGTGGCGCAGCACCACGAGGCACTGGTGGGCCATGGGT

TTACACACGCGCACATCGTTGCGCTCAGCCAACACCCGGCAGCGTTAGGGACCGTCGCTGTCAAGTATCAGCACATAATC

ACGGCGTTGCCAGAGGCGACACACGAAGACATCGTTGGCGTCGGCAAACAGTGGTCCGGCGCACGCGCCCTGGAGGCCTT

GCTCACGAAGGCGGGGGAGTTGAGAGGTCCGCCGTTACAGTTGGACACAGGCCAACTTCTCAAGATTGCAAAACGTGGCG

GCTTGACCTCAGTGCAGGCAGTGCATGCATGGCGCAATGCACTGACGGGTGCCCCCCTGAACCTGACCCCGGACCAAGTG

GTGGCCATCGCCAGCAATATTGGCGGCAAGCAGGCGCTGGAGACGGTACAGCGGCTGTTGCCGGTGCTGTGCCAGGCCAA

TG

>M13R

ACCCTGGACAAGGTGGTGGCCATCGCCAGCAATGGCGGCAAGCAGGCGCTGGAGACGGTGCAGCGGCTGTTGCCGGTGCT

GTGCCAGGACCATGGCCTGACCCCGAACCAGGTGGTGGCCATCGCCAGCAATAGTGGCGGCAAGCAGGCGCTGGAGACGG

TGCAGCGGCTGTTGCCGGTGCTGTGCCAGGACCATGGCCTGACCCCGAACCAGGTGGTGGCCATCGCCAGCAATGGCGGC

AAGCAGGCGCTGGAGAGCATTGTTGCCCAGTTATCTCGCCCTGATCCGGCGTTGGCCGCGTTGACCAACGACCACCTCGT

CGCCTTGGCCTGCCTCGGCGGACGTCCTGCCCTGGATGCAGTGAAAAAGGGATTGCCGCACGCGCCGGAATTGATCAGAA

GAATCAATCGCCGCATTCCCGAACGCACGTCCCATCGCGTTCCCGACCTCGCGCACGTGGTTCGCGTGCTTGGTTTTTTC

CAGAGCCACTCCCACCCAGCGCAAGCATTCGATGACGCCATGACGCAGTTCGAGATGAGCAGGCACGGCTTGGTACAGCT

CTTTCGCAGAGTGGGCGTCACCGAATTCGAAGCCCGCTACGGAACGCTCCCCCCAGCCTCGCAGCGTTGGGACCGTATCC

TCCAGGCATCAGGGATGAAAAGGGCCAAACCGTCCCCTACTTCAGCTCAAACACCGGATCAGGCGTCTTTGCATGCATTC

GCCGATTCGCTGGAGCGTGACCTTGATGCGCCTAGCCCAATGCACGAGGGAGATCAGACAGGGGCAAGCAGCCGTAAACG

GTCCCGATCGGATCGTGCTGTCACCGGCCCCTCCGCACAGCAATCTTTCGAGGTGCGCGTTCCCGAACAGCGCGATGCGC

TGCATTTGCCCCTCAGCTGGAGGGTAAAACGCCCGCGTACCAGGATCGGGGGCGGCCTCCCGGATCC

**pTAL*_Bam_*_HI_-40**

> pCC2FOS-MscI-1

CCATCGCCAGCAATGGCGGCGGCAAGCAGGCGCTGGAGACGGTGCAGCGGCTGTTGCCGGTGCTGTGCCAGGCCCATGGC

CTGACCCTGGACCAGGTCGTGGCCATTGCCAGCAATGGCGGCGGCAAGCAGGCGCTGGAGACGGTGCAGCGGCTGTTGCC

GGTGCTGTGCCAGGCCCATGGCCTGACCCCGGACCAGGTGGTGGCCATCGCCAGCCACGATGGCGGCAAGCAGGCGCTGG

AGACGGTGCAGCGGCTGTTGCCGGTGCTGTGCCAGGCCCATGGCCTGACCCCGGCCCAGGTGGTGGCCATCGCCAGCCAC

GATGGCGGCAAGCAGGCGCTGGAGACGGTGCAGCGGCTGTTGCCGGTGCTGTGCCAGGCCCATGGTCTGACCCTGGACCA

GGTAGTGGCCATTGCCAGCCACGATGGCGGCAAGCAGGCGCTGGAGACGGTGCAGCGGCTGTTGCCGGTGCTGTGCCAGG

CCCATGGTCTGACCCTGGACCAGGTAGTGGCCATTGCCAGCCACGATGGCGGCAAGCAGGCGCTGGAGACGGTGCAGCGG

CTGTTGCCGGTGCTGTGCCAGGACCATGGTCTGACCCCGGCGCAGGTGGTGGCCATCGCCAGCAATAACGGCGGCAAGCA

GGCGCTGGAGACGGTGCAGCGGCTGTTGCCGGTGCTGTGCCAGGACCATGGCCTGACCCCGGACCAGGTGGTGGCCATCG

CCAGCCACGATGGCGGCAAGCAGGCGCTGGAGACGGTGCAGCGGCTGTTGCCGGTGCTGTGCC

>40P1F_1

AAGGCGGGGGAGTTGAGAGGTCCGCCGTTACAGTTGGACACAGGCCAACTTCTCAAGATTGCAAGACGTGGCGGCGTGAC

CGCAGTGGAGGCAGTGCATGCATGGCGCAATGCACTGACGGGTGCCCCCCTGAACCTGACCCCGGACCAAGTGGTGGCCA

TCGCCAGCAATAGTGGCGGCAAGCAGGCGCTGGAGACGGTGCAGCGGCTGTTGCCGGTGCTGTGCCAGGACCATGGCCTG

ACCCCGGACCAGGTCGTGGCCATCGCCAGCCACGATGGCGGCAAGCAGGCGCTGGAGACGGTGCAGCGGCTGTTGCCGGT

GCTGTGCCAGGACCATGGCCTGACCCCGGACCAGGTCGTGGCCATCGCCAGCAATGGCGGCGGCAAGCAGGCGCTGGAGA

CGGTGCAGCGGCTGTTGCCGGTGCTGTGCCAGGACCATGGCCTGACCCCGGACCAGGTGGTGGCCATCGCCAGCAATGGC

GGCGGCAAGCAGGCGCTGGAGACGGTACTGTGCCAGGCCCATGGCCTGACCCCGGCGCAGGTGGTGGCCATCGCCAGCAA

TGGCGGCGGCAAGCAGGCGCTGGAGACGGTGCAGCGGCTGTTGCCGGTGCTGTGCCAGGCCCATGGCCTGACCCTGGACC

AGGTCGTGGCCATTGCCAGCAATGGCGGCGGCAAGCAGGCGCTGGAGACGGTGCAGCGGCTGTTGCCGGTGCTGTGCCAG

GCCCATGGCCTGACCCCGGACCAGGTGGTGGCCATCGCCAGCCACGATGGCGGCAAGCAGGCGCTGGAGACGGTGCAGCG

GCTGTTGCCGGTGCTGTGCCAGGCCCATGGCCTGACCCCGGCCCAGGTGGTGGCCATCGCCAGCCACGATGGCGGCAAGC

AGGCGCTGGAGACGGTGCAGCGGCTGTTGCCGGTGCTGTGCCAGGCCCATGGTCTGACCCTGGACCAGGTAGTGGCCATT

GCCAGCCACGATGGCGGCA

>40P1F_2

AGGCGGGGGAGTTGAGAGGTCCGCCGTTACAGTTGGACACAGGCCAACTTCTCAAGATTGCAAGACGTGGCGGCGTGACC

GCAGTGGAGGCAGTGCATGCATGGCGCAATGCACTGACGGGTGCCCCCCTGAACCTGACCCCGGACCAAGTGGTGGCCAT

CGCCAGCAATAGTGGCGGCAAGCAGGCGCTGGAGACGGTGCAGCGGCTGTTGCCGGTGCTGTGCCAGGACCATGGCCTGA

CCCCGGACCAGGTCGTGGCCATCGCCAGCCACGATGGCGGCAAGCAGGCGCTGGAGACGGTGCAGCGGCTGTTGCCGGTG

CTGTGCCAGGACCATGGCCTGACCCCGGACCAGGTCGTGGCCATCGCCAGCAATGGCGGCGGCAAGCAGGCGCTGGAGAC

GGTGCAGCGGCTGTTGCCGGTGCTGTGCCAGGACCATGGCCTGACCCCGGACCAGGTGGTGGCCATCGCCAGCAATGGCG

GCGGCAAGCAGGCGCTGGAGACGGTACTGTGCCAGGCCCATGGCCTGACCCCGGCGCAGGTGGTGGCCATCGCCAGCAAT

GGCGGCGGCAAGCAGGCGCTGGAGACGGTGCAGCGGCTGTTGCCGGTGCTGTGCCAGGCCCATGGCCTGACCCTGGACCA

GGTCGTGGCCATTGCCAGCAATGGCGGCGGCAAGCAGGCGCTGGAGACGGTGCAGCGGCTGTTGCCGGTGCTGTGCCAGG

CCCATGGCCTGACCCCGGACCAGGTGGTGGCCATCGCCAGCCACGATGGCGGCAAGCAGGCGCTGGAGACGGTGCAGCGG

CTGTTGCCGGTGCTGTGCCAGGCCCATGGCCTGACCCCGGCCCAGGTGGTGGCCATCGCCAGCCACGATGGCGGCAAGCA

GGCGCTGGAGACGGTGCAGCGGCTGTTGCCGGTGCTGTGCCAGGCCCATGGTCTGACCCTGGACCAGGTAGTGGCCATTG

CCAGCCACGATGGCGGCAA

>40P1R_1

GGGCAACAATGCTCTCCAGCGCCTGCTTGCCGCCATGGCTGGCGATGGCCACCACCTGGTCCGGGGTCAGGCCATGGGCC

TGGCACAGCACCGGCAACAGCCGCTGCACCGTCTCCAGCGCCTGCTTGCCGCCGTTATTGTTGGCGATGGCCACGACCTG

GTCCGGGGTCAGGCCATGGTCCTGGCACAGCACCGGCAACAGCCGCTGCACCGTCTCCAGCGCCTGCTTGCCGCCATCGT

GGCTGGCGATGGCCACGACCTGGTCCGGGGTCAGGCCATGGTCCTGGCACAGCACCTGCAACAGCCGTTGCACCGTCTCC

AGCGCCTGCTTGCCGCCATCGTGGCTGGCGATGGCCACCACCTGCGCCGGGGTCAGACCATGGTCCTGGCACAGCACCGG

CAACAGCCGCTGCACCGTCTCCAGCGCCTGCTTGCCGCCATCGTGGCTGGCGATGGCCACGACCTGGTCCGGGGTCAGGC

CATGGGCCTGGCACAGCACCGGCAACAGCCGCTGCACCGTCTCCAGCGCCTGCTTGCCGCCATCGTGGCTGGCGATGGCC

ACCACCTGGTCCGGGGTCAGGCCATGGTCCTGGCACAGCACCGGCAACAGCCGCTGCACCGTCTCCAGCGCCTGCTTGCC

GCCGTTATTGCTGGCGATGGCCACCACCTGCGCCGGGGTCAGACCATGGTCCTGGCACAGCACCGGCAACAGCCGCTGCA

CCGTCTCCAGCGCCTGCTTGCCGCCATCGTGGCTGGCAATGGCCACTACCTGGTCCAGGGTCAGACCATGGGCCTGGCAC

AGCACCGGCAACAGCCGCTGCACCGTCTCCAGCGCCTGCTTGCCGCCATCGTGGCTGGCAATGGCCACTACCTGGTCCAG

GGTCAGACCATGGGCCTGGCACAGCACCGGCAACAGCCGCTGCACCGTCTCCAGCGCCTGCTTGCCGCCATCGTGGCTGG

CGATGGCCA

>40P1R_2

GTGGTGGCCATCGCCAGCCACGATGGCGGCAAGCAGGCGCTGGAGACGGTGCAGCGGCTGTTGCCGGTGCTGTGCCAGGC

CCATGGTCTGACCCTGGACCAGGTAGTGGCCATTGCCAGCCACGATGGCGGCAAGCAGGCGCTGGAGACGGTGCAGCGGC

TGTTGCCGGTGCTGTGCCAGGCCCATGGTCTGACCCTGGACCAGGTAGTGGCCATTGCCAGCCACGATGGCGGCAAGCAG

GCGCTGGAGACGGTGCAGCGGCTGTTGCCGGTGCTGTGCCAGGACCATGGTCTGACCCCGGCGCAGGTGGTGGCCATCGC

CAGCAATAACGGCGGCAAGCAGGCGCTGGAGACGGTGCAGCGGCTGTTGCCGGTGCTGTGCCAGGACCATGGCCTGACCC

CGGACCAGGTGGTGGCCATCGCCAGCCACGATGGCGGCAAGCAGGCGCTGGAGACGGTGCAGCGGCTGTTGCCGGTGCTG

TGCCAGGCCCATGGCCTGACCCCGGACCAGGTCGTGGCCATCGCCAGCCACGATGGCGGCAAGCAGGCGCTGGAGACGGT

GCAGCGGCTGTTGCCGGTGCTGTGCCAGGACCATGGTCTGACCCCGGCGCAGGTGGTGGCCATCGCCAGCCACGATGGCG

GCAAGCAGGCGCTGGAGACGGTGCAACGGCTGTTGCAGGTGCTGTGCCAGGACCATGGCCTGACCCCGGACCAGGTCGTG

GCCATCGCCAGCCACGATGGCGGCAAGCAGGCGCTGGAGACGGTGCAGCGGCTGTTGCCGGTGCTGTGCCAGGACCATGG

CCTGACCCCGGACCAGGTCGTGGCCATCGCCAACAATAACGGCGGCAAGCAGGCGCTGGAGACGGTGCAGCGGCTGTTGC

CGGTGCTGTGCCAGGCCCATGGCC

>40P2F

CGCGCACATCGTTGCGCTCAGCCAACACCCGGCAGCGTTAGGGACCGTTGCTGTCACGTATCAAGACATAATCACGGCGT

TGCCAGAGGCGACACACGAAGACATCGTTGGCGTCGGCAAACAGTTGTCCGGCGCACGCGCCCTGGAGGCCTTGCTCACG

AAGGCGGGGGAGTTGAGAGGTCCGCCGTTACAGTTGGACACAGGCCAACTTCTCAAGATTGCAAGACGTGGCGGCGTGAC

CGCAGTGGAGGCAGTGCATGCATGGCGCAATGCACTGACGGGTGCCCCCCTGAACCTGACCCCGGACCAAGTGGTGGCCA

TCGCCAGCAATAGTGGCGGCAAGCAGGCGCTGGAGACGGTGCAGCGGCTGTTGCCGGTGCTGTGCCAGGACCATGGCCTG

ACCCCGGACCAGGTCGTGGCCATCGCCAGCCACGATGGCGGCAAGCAGGCGCTGGAGACGGTGCAGCGGCTGTTGCCGGT

GCTGTGCCAGGACCATGGCCTGACCCCGGACCAGGTCGTGGCCATCGCCAGCAATGGCGGCGGCAAGCAGGCGCTGGAGA

CGGTGCAGCGGCTGTTGCCGGTGCTGTGCCAGGACCATGGCCTGACCCCGGACCAGGTGGTGGCCATCGCCAGCAATGGC

GGCGGCAAGCAGGCGCTGGAGACGGTACTGTGCCAGGCCCATGGCCTGACCCCGGCGCAGGTGGTGGCCATCGCCAGCAA

TGGCGGCGGCAAGCAGGCGCTGGAGACGGTGCAGCGGCTGTTGCCGGTGCTGTGCCAGGCCCATGGCCTGACCCTGGACC

AGGTCGTGGCCATTGCCAGCAATGGCGGCGGCAAGCAGGCGCTGGAGACGGTGCAGCGGCTGTTGCCGGTGCTGTGCCAG

GCCCATGGCCTGACCCCGGACCAGGTGGTGGCCATCGCCAGCCACGATGGCGGCAAGCAGGCGCTGGAGACGGTGCAGCG

GCTGTTGCCGGTGCTG

>40P2R

ATTATGTCTTGATACGTGACAGCAACGGTCCCTAACGCTGCCGGGTGTTGGCTGAGCGCAACGATGTGCGCGTGTGTAAA

CCCATGGCCCACCAGTGCCTCGTGGTGCTGCGCCACTGTCGCGAGCGTGAGTAGATCCACCTGCGCGGCCGGCGAAGTGT

CTGCTGGGGCAGCCTCTGTATGAGGCGTGCCGACGGCAGGCATCGAATCAAAAAGCGATGTATCAGGAAGCGACGGATCG

AACGGACGGAGCCGATCGGTGGAGCTGCCCGCCGAGAACGCAGGCAAGGGGGCAGGGGGAGATGGCAGCCGGGTCCGGGA

CACCGTCCGCCGAGCGGGCAAGCCATCCAGAGGGCTGCCAGCAGGCGCAGACACCCCACGATCTGCAGTCGGCTGAACCC

TATCCGGTTGGGGTCCGGGCAGAGGCTCGCGGGCAGGACTTGGCGTGCGCGAACGAATGGGATCC

>40P3F

CGCATGATTGATGGAGCTATTGCCTCAGTGAGGCTCAGTCGGGGGACTATCTGAGCGACGTCCCCCCATCCTGAGTAGCA

GTCGGGTTTAGAGTCCGGGGTTGATGATATCGCTATTGGCCAACTGCTTGGCATACGTTGCCGGCGTCATTGCGCCGATT

GCTTTCTTTGGGCGGTGTTGGTTTTATTCGCGGCGCCAGCGTTTGATACGCCCAGGGTTCCGTAGACACTCAAGACCCTC

GTTGCGCGTAGCGCCGTTGAAACTCTACAGGCGATAGGTTGCCGGTTGAACCATGACGGCGTTGTGGGTTGTAGAACATT

TCGATGTCGTCGAACACCTCGGCGCGTGCGGCGTCCTTGGTGGGGTAGATCCGCCGCCTGATCCGCTTGCGTTTGAGCAG

ACCGAAGAAGCTCTCCACCGGGGCATTGTCATGGCAGTTGCCACGTCGACTCATGCTGCACACCAAACCGTGGGACACCA

GGAAACTGCGCCAGTCATCGCTGGTGTAGACCGACCCTTGGTCCGAGTGAACCAGGCAACCGGCGCTGGGCTTGCGCCGC

CACACCGCCGACAGCAAGGCCTGCACGACCAACTCGGTATCGGCTCGATCGCGCATCGCCCAGCCGACGACCTGCCTGGA

AAACAGATCGATCACCACAGCCAGGTACATCCAGCCTTCATGCGTACGGATAAAGGTGAAGTCGCTCGCCCAGGCCGTAT

CCGGCTCGGTCACGTCGAACTGCCGATCCAGCAGGTTGGCTGCCGCCTTGCACGGCATTCCGCCATGGAAGCGCGGTTTG

CGACCATAGCCCACCTGGGCACGCAGCCCATCGGCGCGCATCAGCCGATGCACCCGATGGCGACTGCAACGCTCACCCAG

ATCGCGCAGATCCTTGGCGATCTTGCGATGCCCATACACACTGCCGCTGGCCAGCCAGTGGTGCTTGATCAGCCCCA

>Ct seqF2

AGTGAGGCTCAGTCGGGGGACTATCTGAGCGACGTCCCCCCATCCTGAGTAGCAGTCGGGTTTAGAGTCCGGGGTTGATG

ATATCGCTATTGGCCAACTGCTTGGCATACGTTGCCGGCGTCATTGCGCCGATTGCTTTCTTTGGGCGGTGTTGGTTTTA

TTCGCGGCGCCAGCGTTTGATACGCCCAGGGTTCCGTAGACACTCAAGACCCTCGTTGCGCGTAGCGCCGTTGAAACTCT

ACAGGCGATAGGTTGCCGGTTGAACCATGACGGCGTTGTGGGTTGTAGAACATTTCGATGTCGTCGAACACCTCGGCGCG

TGCGGCGTCCTTGGTGGGGTAGATCCGCCGCCTGATCCGCTTGCGTTTGAGCAGACCGAAGAAGCTCTCCACCGGGGCAT

TGTCATGGCAGTTGCCACGTCGACTCATGCTGCACACCAAACCGTGGGACACCAGGAAACTGCGCCAGTCATCGCTGGTG

TAGACCGACCCTTGGTCCGAGTGAACCAGGCAACCGGCGCTGGGCTTGCGCCGCCACACCGCCGACAGCAAGGCCTGCAC

GACCAACTCGGTATCGGCTCGATCGCGCATCGCCCAGCCGACGACCTGCCTGGAAAACAGATCGATCACCACAGCCAGGT

ACATCCAGCCTTCATGCGTACGGATAAAGGTGAAGTCGCTCGCCCAGGCCGTATCCGGCTCGGTCACGTCGAACTGCCGA

TCCAGCAGGTTGGCTGCCGCCTTGCACGGCATTCCGCCATGGAAGCGCGGTTTGCGACCATAGCCCACCTGGGCACGCAG

CCCATCGGCGCGCATCAGCCGATGCACCCGATGGCGACTGCAACGCTCACCCAGATCGCGCAGATCCTTGGCGATCTTGC

GATGCCCATACACACTGCCGCTGGCCAGCCAGTGGTGCTTGATCAGCCCCAGCAGGCGTTCGTCTTCCTTGGCG

>IS 2

TCGTCGAACACCTCGGCGCGTGCGGCGTCCTTGGTGGGGTAGATCCGCCGCCTGATCCGCTTGCGTTTGAGCAGACCGAA

GAAGCTCTCCACCGGGGCATTGTCATGGCAGTTGCCACGTCGACTCATGCTGCACACCAAACCGTGGGACACCAGGAAAC

TGCGCCAGTCATCGCTGGTGTAGACCGACCCTTGGTCCGAGTGAACCAGGCAACCGGCGCTGGGCTTGCGCCGCCACACC

GCCGACAGCAAGGCCTGCACGACCAACTCGGTATCGGCTCGATCGCGCATCGCCCAGCCGACGACCTGCCTGGAAAACAG

ATCGATCACCACAGCCAGGTACATCCAGCCTTCATGCGTACGGATAAAGGTGAAGTCGCTCGCCCAGGCCGTATCCGGCT

CGGTCACGTCGAACTGCCGATCCAGCAGGTTGGCTGCCGCCTTGCACGGCATTCCGCCATGGAAGCGCGGTTTGCGACCA

TAGCCCACCTGGGCACGCAGCCCATCGGCGCGCATCAGCCGATGCACCCGATGGCGACTGCAACGCTCACCCAGATCGCG

CAGATCCTTGGCGATCTTGCGATGCCCATACACACTGCCGCTGGCCAGCCAGTGGTGCTTGATCAGCCCCAGCAGGCGTT

CGTCTTCCTTGGCGCGCTCACTGTCGGGCGACTTTAGCCACGCGTCGTATCCAGCCCGGTTCACCCGCAACACCCGGCAC

ATCGCGCACACCCTGAATTCTTCGCGGTGGGCTTGCATGAGGACGTACTTTGCCTTTACCCCTTGGCAAAGTACGCGACG

GCCTTTTTTAGGATGTCGCGCTCCTCCGTCACCCGGCGCAACTCGGTCTTCAGCCGCCGAACCTCGGCGCTCTGGTCCAC

TTCGGCACGCTGCACCACGCCGGGCTTGCCGAACGTACGCAGCCAGGCGTACAGGCTGTGCGTGGTGACACCCAGCCGCT

GTG

>IS seqR

CGATGGCGGCAAGCAGGCGCTGGAGACGGTGCAGCGGCTGTTGCCGGTGCTGTGCCAGGCCCATGGCCTGACCCCGGACC

AGGTCGTGGCCATCGCCAGCCACGATGGCGGCAAGCAGGCGCTGGAGACGGTGCAGCGGCTGTTGCCGGTGCTGTGCCAG

GACCATGGTCTGACCCCGGCGCAGGTGGTGGCCATCGCCAGCCACGATGGCGGCAAGCAGGCGCTGGAGACGGTGCAACG

GCTGTTGCAGGTGCTGTGCCAGGACCATGGCCTGACCCCGGACCAGGTCGTGGCCATCGCCAGCCACGATGGCGGCAAGC

AGGCGCTGGAGACGGTGCAGCGGCTGTTGCCGGTGCTGTGCCAGGACCATGGCCTGACCCCGGACCAGGTCGTGGCCATC

GCCAACAATAACGGCGGCAAGCAGGCGCTGGAGACGGTGCAGCGGCTGTTGCCGGTGCTGTGCCAGGCCCATGGCCTGAC

CCCGGACCAGGTGGTGGCCATCGCCAGCCATGGCGGCAAGCAGGCGCTGGAGAGCATTGTTGCCCAGTTATCTCGCCGTG

ATCCGGCGTTGGCCGCGTTGACCAACGACCAACTCGTCGCCTTGGCCTGCCTCGGCGGACGTCCTGCCCCGCATTCAAGG

AAGAGGAAATCGCATGATTGATGGAGCTATTGCCTCAGTGAGGCTCAGTCGGGGGACTATCTGAGCGACGTCCCCCCATC

CTGAGTAGCAGTCGGGTTTAGAGTCCGGGGTTGATGATATCGCTATTGGCCAACTGCTTGGCATACGTTGCCGGCGTCAT

TGCGCCGATTGCTTTCTTTGGGCGGTGTTGGTTTTATTCGCGGCGCCAGCGTTTGATACGCCCAGGGTT

>M13F

GGATCCCATTCGTTCGCGCACGCCAAGTCCTGCCCGCGAGCCTCTGCCCGGACCCCAACCGGATAGGGTTCAGCCGACTG

CAGATCGTGGGGTGTCTGCGCCTGCTGGCAGCCCTCTGGATGGCTTGCCCGCTCGGCGGACGGTGTCCCGGACCCGGCTG

CCATCTCCCCCTGCCCCCTTGCCTGCGTTCTCGGCGGGCAGCTCCACCGATCGGCTCCGTCCGTTCGATCCGTCGCTTCC

TGATACATCGCTTTTTGATTCGATGCCTGCCGTCGGCACGCCTCATACAGAGGCTGCCCCAGCAGACACTTCGCCGGCCG

CGCAGGTGGATCTACTCACGCTCGCGACAGTGGCGCAGCACCACGAGGCACTGGTGGGCCATGGGTTTACACACGCGCAC

ATCGTTGCGCTCAGCCAACACCCGGCAGCGTTAGGGACCGTTGCTGTCACGTATCAAGACATAATCACGGCGTTGCCAGA

GGCGACACACGAAGACATCGTTGGCGTCGGCAAACAGTTGTCCGGCGCACGCGCCCTGGAGGCCTTGCTCACGAAGGCGG

GGGAGTTGAGAGGTCCGCCGTTACAGTTGGACACAGGCCAACTTCTCAAGATTGCAAGACGTGGCGGCGTGACCGCAGTG

GAGGCAGTGCATGCATGGCGCAATGCACTGACGGGTGCCCCCCTGAACCTGACCCCGGACCAAGTGGTGGCCATCGCCAG

CAATAGTGGCGGCAAGCAGGCGCTGGAGACGGTGCAGCGGCTGTTGCCGGTGCTGTGCCAGGACCATGGCCTGACCCCGG

ACCAGGTCGTGGCCATCGCCAGCCACGATGGCGGCAAGCAGGCGCTGGAGACGGTGCAGCGGCTGTTGCCGGTGCTGTGC

CAGGACCATGGCCTGACCCCGGACCAGGTCGTGGCCATCGCCAGCAATGGCGGCGGCAAGCAGGCGC

>M13R

GTACTTTGCCTTTACCCCTTGGCAAAGTACGCGACGGCCTTTTTTAGGATGTCGCGCTCCTCCGTCACCCGGCGCAACTC

GGTCTTCAGCCGCCGAACCTCGGCGCTCTGGTCCACTTCGGCACGCTGCACCACGCCGGGCTTGCCGAACGTACGCAGCC

AGGCGTACAGGCTGTGCGTGGTGACACCCAGCCGCTGTGCGACCTCTGCCACCTTGAAGCCACGATCGGTCACTTACCGG

ACCGCTTCGATCTTGAACTCATCCGTATACCGCTTGCTGCTCATGGACACCTCCGAATCAGTCATTTTCTATGACCTTGA

GATGTCTAGGAAACACTGGGCGTATCACTTCTGTCATCCCGCCTAACGGGTTGTCAGAGCTTCCGCTCCTTCCTACCGGC

ATGGCATGGCGGAATCCGGTGCGGGACTACGACTATGATCGGTCGGAGTGGCAGATCTACATTGTCTTACGACACGCATC

GGTAGATCTGGCGCGCGTCAATCAGCTTGCCGAACCATCCGGCACCACGGCGGCAAGCGCAAGGTCAACGGCGTGGTGAC

CGCCGTGCGGCAGTTGGGCGCCGATGGCGGCTTGTCCAGCTACCGCTTGCGGATACAGCCCGCGCTGGCGCTGTTGGCCT

ATCGCCGCACCTGCCGCATCTTCCAGGAGGAGAGCGTGCCGGACATCGTCGCGCAGATCGTGCAGGAACATCGCGCCAGC

AATCCCCCGATCGCCGCCAGCTTCCGCCTGGACCAGCAGCTGCGCCAGCGACGTCCCCCCGAAGTTGCGTACTGCCATGC

GTACTCGGAAGAGCATGTAGGGACCACAGACCGTTAGTGTTGAGGCAACCCAGGTTTCTGCAAAGAGGTATGCCTTATGG

ATCC

**pTAL*_Bam_*_HI_-41**

> pCC2FOS-MscI-1

GCGGCAACCAGGCGCTGGAGACGGTGCAGCGGCTGTTGCCGGTGCTGTGCCAGGCCCATGGCCTGACCCCGGACCAGGTC

GTGGCCATCGCCAGCCATGGCGGCGGCAAGCAGGCGCTGGAGACGGTGCAGCGGCTGTTGCCGGTGCTGTGCCAGGACCA

TGGCCTGACCCCGGACCAGGTGGTGGCCATCGCCAGCAATATTGGCGGCAAGCAGGCGCTGGAGACGGTGCAACGGCTGT

TGCCGGTGCTGTGCCAGGACCATGGCCTGACCCCGGACCAGGTGGTGGCCATCGCCAGCAATATTGGCGGCAAGCAGGCG

CTGGAGACGGTGCAGCGGCTGTTGCCGGTGCTGTGCCAGGACCATGGCCTGACCCCGGACCAGGTGGTGGCCATCGCCAG

CAATATTGGCGGCAAGCAGGCGCTGGAGACGGTGCAGCGGCTGTTGCCGGTGCTGTGCCAGGACCATGGCCTGACCCCGG

ACCAGGTGGTGGCCATCGCCAACAATAACGGCGGCAAGCAGGCGCTGGAGACGGTGCAACGGCTGTTGCCGGTGCTGTGC

CAGGACCATGGCCTGACCCCGGACCAGGTGGTGGCCATCGCCAGCCACGATGGCGGCAAGCAGGCGCTGGAGACGGTGCA

GCGGCTGTTGCCGGTGCTGTGCCAGGGCCATGGCCTGACCCCGGACAAGGTGGTGGCCATCGCCAGCAATAGTGGCGGCA

AGCAGGCGCTGGAGACGGTGCAGCGGCTGTTGCCAGTGCTGTGCCAGGCCCATGGCCTGACCCCGGACAAGGTGGTGGCC

ATCGCCAACAATAACGGCGGCAAGCAGGCGCTGGAGACGGTGCAGCGGCTGTTGCCGGTGCTGTGCCAGGACCATGGCCT

GACCCCGGACCAGGTGGTGGCCATCGCCAGCAATAGTGGCGGCAAGCAGGCGCTGGAGACGGTGCAGCGGCTGTTGCCGG

TGCTGTGCCA

> pCC2FOS-MscI-2

CCATCGCCAGCAATATTGGCGGCAAGCAGGCGCTGGAGACGGTGCAGCGGCTGTTGCCGGTGCTGTGCCAGGACCATGGC

CTGACCCCGGACCAGGTGGTGGCCATCGCCAACAATAACGGCGGCAAGCAGGCGCTGGAGACGGTGCAACGGCTGTTGCC

GGTGCTGTGCCAGGACCATGGCCTGACCCCGGACCAGGTGGTGGCCATCGCCAGCCACGATGGCGGCAAGCAGGCGCTGG

AGACGGTGCAGCGGCTGTTGCCGGTGCTGTGCCAGGGCCATGGCCTGACCCCGGACAAGGTGGTGGCCATCGCCAGCAAT

AGTGGCGGCAAGCAGGCGCTGGAGACGGTGCAGCGGCTGTTGCCAGTGCTGTGCCAGGCCCATGGCCTGACCCCGGACAA

GGTGGTGGCCATCGCCAACAATAACGGCGGCAAGCAGGCGCTGGAGACGGTGCAGCGGCTGTTGCCGGTGCTGTGCCAGG

ACCATGGCCTGACCCCGGACCAGGTGGTGGCCATCGCCAGCAATAGTGGCGGCAAGCAGGCGCTGGAGACGGTGCAGCGG

CTGTTGCCGGTGCTGTGCCAGGACCATGGTCTGACCCCGGCCCAGGTGGTGGCCATCGCCAACAATAACGGCGGCAAGCA

GGCGCTGGAGACGGTGCGGCGGCTGTTGCCGGTGCTGTGCCAGGACCATGGCCTGACCCCGGACCAGGTCGTGGCCATCG

CCAGCCACGATGGCGGCAAGCAGGCGCTGGAGACGGTGCAGCGGCTGTTGCCGGTACTGTGCCAGGACCATGGCCTGACC

CCGGACCAGGTGGTGGCCATCGCCAACAATAACGGCGGCAAGCAGGCGCTGGAGACGGTGCAGCGGCTGTTGCCGGTGCT

GTGCCAGGACCA

> pCC2FOS-MscI-3

GGCGGCAAGCAGGCGCTGGAGACGGTGCAGCGGCTGTTGCCGGTGCTGTGCCAGGGCCATGGCCTGACCCCGGACAAGGT

GGTGGCCATCGCCAGCAATAGTGGCGGCAAGCAGGCGCTGGAGACGGTGCAGCGGCTGTTGCCAGTGCTGTGCCAGGCCC

ATGGCCTGACCCCGGACAAGGTGGTGGCCATCGCCAACAATAACGGCGGCAAGCAGGCGCTGGAGACGGTGCAGCGGCTG

TTGCCGGTGCTGTGCCAGGACCATGGCCTGACCCCGGACCAGGTGGTGGCCATCGCCAGCAATAGTGGCGGCAAGCAGGC

GCTGGAGACGGTGCAGCGGCTGTTGCCGGTGCTGTGCCAGGACCATGGTCTGACCCCGGCCCAGGTGGTGGCCATCGCCA

ACAATAACGGCGGCAAGCAGGCGCTGGAGACGGTGCGGCGGCTGTTGCCGGTGCTGTGCCAGGACCATGGCCTGACCCCG

GACCAGGTCGTGGCCATCGCCAGCCACGATGGCGGCAAGCAGGCGCTGGAGACGGTGCAGCGGCTGTTGCCGGTACTGTG

CCAGGACCATGGCCTGACCCCGGACCAGGTGGTGGCCATCGCCAACAATAACGGCGGCAAGCAGGCGCTGGAGACGGTGC

AGCGGCTGTTGCCGGTGCTGTGCCAGGACCATGGCCTGACCCAGGACCAGGTGGTGGCCATCGCCAGCAATATTGGCGGC

AAGCAGGCGCTGGAGACGGTGCAGCGGCTGTTGCCGGTGCTGTGCCAGGACCATGGCCTGACCCTGGACCAGGTGGTGGC

CATCGCCAGCCACGATGGCGGCAAACAGGCGCTGGAGACGGTGCAGCGGCTGTTGCCGGTGCTGTGCCAGGACCATGGCC

TGACCCCGGCCCAGGTGGTGGCCATCGCCAACAATAACGGCGGCAAGCAGGCGCTGGAGACGGTGCAGCGGCTGTTGCCG

GTG

> pCC2FOS-MscI-4

CCATCGCCAGCAATAGTGGCGGCAAGCAGGCGCTGGAGACGGTGCAGCGGCTGTTGCCGGTGCTGTGCCAGGACCATGGT

CTGACCCCGGCCCAGGTGGTGGCCATCGCCAACAATAACGGCGGCAAGCAGGCGCTGGAGACGGTGCGGCGGCTGTTGCC

GGTGCTGTGCCAGGACCATGGCCTGACCCCGGACCAGGTCGTGGCCATCGCCAGCCACGATGGCGGCAAGCAGGCGCTGG

AGACGGTGCAGCGGCTGTTGCCGGTACTGTGCCAGGACCATGGCCTGACCCCGGACCAGGTGGTGGCCATCGCCAACAAT

AACGGCGGCAAGCAGGCGCTGGAGACGGTGCAGCGGCTGTTGCCGGTGCTGTGCCAGGACCATGGCCTGACCCAGGACCA

GGTGGTGGCCATCGCCAGCAATATTGGCGGCAAGCAGGCGCTGGAGACGGTGCAGCGGCTGTTGCCGGTGCTGTGCCAGG

ACCATGGCCTGACCCTGGACCAGGTGGTGGCCATCGCCAGCCACGATGGCGGCAAACAGGCGCTGGAGACGGTGCAGCGG

CTGTTGCCGGTGCTGTGCCAGGACCATGGCCTGACCCCGGCCCAGGTGGTGGCCATCGCCAACAATAACGGCGGCAAGCA

GGCGCTGGAGACGGTGCAGCGGCTGTTGCCGGTGCTGTGCCAGGACCATGGCCTGAGCCCGGACCAGGTCGTGGCCATCG

CCAGCAATATTGGCGGCAAGCAGGCGCTGGAGACGGTGCAGCGGCTGTTGCCGGTGCTGTGCCAGGACCATGGCCTGACC

CCGGACCAGGTGGTGGCCATCGCCAGCAATGGCGGCGGCAAGCAGGCGCTGGAGACGGTGCAGCGGCTGTTGCCGGT

> pCC2FOS-MscI-5

CCATCGCCAGCAATATTGGCGGCAAGCAGGCGCTGGAGACGGTGCAGCGGCTGTTGCCGGTGCTGTGCCAGGACCATGGC

CTGACCCCGGACCAGGTGGTGGCCATCGCCAACAATAACGGCGGCAAGCAGGCGCTGGAGACGGTGCAACGGCTGTTGCC

GGTGCTGTGCCAGGACCATGGCCTGACCCCGGACCAGGTGGTGGCCATCGCCAGCCACGATGGCGGCAAGCAGGCGCTGG

AGACGGTGCAGCGGCTGTTGCCGGTGCTGTGCCAGGGCCATGGCCTGACCCCGGACAAGGTGGTGGCCATCGCCAGCAAT

AGTGGCGGCAAGCAGGCGCTGGAGACGGTGCAGCGGCTGTTGCCAGTGCTGTGCCAGGCCCATGGCCTGACCCCGGACAA

GGTGGTGGCCATCGCCAACAATAACGGCGGCAAGCAGGCGCTGGAGACGGTGCAGCGGCTGTTGCCGGTGCTGTGCCAGG

ACCATGGCCTGACCCCGGACCAGGTGGTGGCCATCGCCAGCAATAGTGGCGGCAAGCAGGCGCTGGAGACGGTGCAGCGG

CTGTTGCCGGTGCTGTGCCAGGACCATGGTCTGACCCCGGCCCAGGTGGTGGCCATCGCCAACAATAACGGCGGCAAGCA

GGCGCTGGAGACGGTGCGGCGGCTGTTGCCGGTGCTGTGCCAGGACCATGGCCTGACCCCGGACCAGGTCGTGGCCATCG

CCAGCCACGATGGCGGCAAGCAGGCGCTGGAGACGGTGCAGCGGCTGTTGCCGGTACTGTGCCAGGACCATGGCCTGACC

CCGGACCAGGTGGTGGCCATCGCCAACAATAACGGCGGCAAGCAGGCGCTGGAGACGGTGCAGCGGCTGTTGCCGGTGCT

GTGCCAGGACC

> pCC2FOS-MscI-6

CCATCGCCAGCAATAGTGGCGGCAAGCAGGCGCTGGAGACGGTGCAGCGGCTGTTGCCAGTGCTGTGCCAGGCCCATGGC

CTGACCCCGGACAAGGTGGTGGCCATCGCCAACAATAACGGCGGCAAGCAGGCGCTGGAGACGGTGCAGCGGCTGTTGCC

GGTGCTGTGCCAGGACCATGGCCTGACCCCGGACCAGGTGGTGGCCATCGCCAGCAATAGTGGCGGCAAGCAGGCGCTGG

AGACGGTGCAGCGGCTGTTGCCGGTGCTGTGCCAGGACCATGGTCTGACCCCGGCCCAGGTGGTGGCCATCGCCAACAAT

AACGGCGGCAAGCAGGCGCTGGAGACGGTGCGGCGGCTGTTGCCGGTGCTGTGCCAGGACCATGGCCTGACCCCGGACCA

GGTCGTGGCCATCGCCAGCCACGATGGCGGCAAGCAGGCGCTGGAGACGGTGCAGCGGCTGTTGCCGGTACTGTGCCAGG

ACCATGGCCTGACCCCGGACCAGGTGGTGGCCATCGCCAACAATAACGGCGGCAAGCAGGCGCTGGAGACGGTGCAGCGG

CTGTTGCCGGTGCTGTGCCAGGACCATGGCCTGACCCAGGACCAGGTGGTGGCCATCGCCAGCAATATTGGCGGCAAGCA

GGCGCTGGAGACGGTGCAGCGGCTGTTGCCGGTGCTGTGCCAGGACCATGGCCTGACCCTGGACCAGGTGGTGGCCATCG

CCAGCCACGATGGCGGCAAACAGGCGCTGGAGACGGTGCAGCGGCTGTTGCCGGTGCTGTGCCAGGACCATGGCCTGACC

CCGGCCCAGGTGGTGGCCATCGCCAACAATAACGGCGGCAAGCAGGCGCTGGAGACGGTGCAGCGGCTGTTGCCGGTGCT

GTGCC

>41P1F_1

CGGGGGAGTTGAGAGGTCCGCCGTTACAGTTGGACACAGGCCAACTTCTCAAGATTGCAAAACGTGGCGGCGTGACCGCA

GTGGAGGCAGTGCATGCATGGCGCAATGCACTGACGGGTGCCCCCCTGAACCTGACCCCGGACCAAGTGGTGGCCATCGC

CAGCAATATTGGCGGCAACCAGGCGCTGGAGACGGTGCAGCGGCTGTTGCCGGTGCTGTGCCAGGCCCATGGCCTGACCC

CGGACCAGGTCGTGGCCATCGCCAGCCATGGCGGCGGCAAGCAGGCGCTGGAGACGGTGCAGCGGCTGTTGCCGGTGCTG

TGCCAGGACCATGGCCTGACCCCGGACCAGGTGGTGGCCATCGCCAGCAATATTGGCGGCAAGCAGGCGCTGGAGACGGT

GCAACGGCTGTTGCCGGTGCTGTGCCAGGACCATGGCCTGACCCCGGACCAGGTGGTGGCCATCGCCAGCAATATTGGCG

GCAAGCAGGCGCTGGAGACGGTGCAGCGGCTGTTGCCGGTGCTGTGCCAGGACCATGGCCTGACCCCGGACCAGGTGGTG

GCCATCGCCAGCAATATTGGCGGCAAGCAGGCGCTGGAGACGGTGCAGCGGCTGTTGCCGGTGCTGTGCCAGGACCATGG

CCTGACCCCGGACCAGGTGGTGGCCATCGCCAACAATAACGGCGGCAAGCAGGCGCTGGAGACGGTGCAACGGCTGTTGC

CGGTGCTGTGCCAGGACCATGGCCTGACCCCGGACCAGGTGGTGGCCATCGCCAGCCACGATGGCGGCAAGCAGGCGCTG

GAGACGGTGCAGCGGCTGTTGCCGGTGCTGTGCCAGGGCCATGGCCTGACCCCGGACAAGGTGGTGGCCATCGCCAGCAA

TAGTGGCGGCAAGCAGGCGCTGGAGACGGTGCAGCGGCTGTTGCCAGTGCTGTGCCAGGCCCATGGCCTGACCCCGGACA

AGGTGGTGGCCATCGC

>41P1F_2

GGCGGGGGAGTTGAGAGGTCCGCCGTTACAGTTGGACACAGGCCAACTTCTCAAGATTGCAAAACGTGGCGGCGTGACCG

CAGTGGAGGCAGTGCATGCATGGCGCAATGCACTGACGGGTGCCCCCCTGAACCTGACCCCGGACCAAGTGGTGGCCATC

GCCAGCAATATTGGCGGCAACCAGGCGCTGGAGACGGTGCAGCGGCTGTTGCCGGTGCTGTGCCAGGCCCATGGCCTGAC

CCCGGACCAGGTCGTGGCCATCGCCAGCCATGGCGGCGGCAAGCAGGCGCTGGAGACGGTGCAGCGGCTGTTGCCGGTGC

TGTGCCAGGACCATGGCCTGACCCCGGACCAGGTGGTGGCCATCGCCAGCAATATTGGCGGCAAGCAGGCGCTGGAGACG

GTGCAACGGCTGTTGCCGGTGCTGTGCCAGGACCATGGCCTGACCCCGGACCAGGTGGTGGCCATCGCCAGCAATATTGG

CGGCAAGCAGGCGCTGGAGACGGTGCAGCGGCTGTTGCCGGTGCTGTGCCAGGACCATGGCCTGACCCCGGACCAGGTGG

TGGCCATCGCCAGCAATATTGGCGGCAAGCAGGCGCTGGAGACGGTGCAGCGGCTGTTGCCGGTGCTGTGCCAGGACCAT

GGCCTGACCCCGGACCAGGTGGTGGCCATCGCCAACAATAACGGCGGCAAGCAGGCGCTGGAGACGGTGCAACGGCTGTT

GCCGGTGCTGTGCCAGGACCATGGCCTGACCCCGGACCAGGTGGTGGCCATCGCCAGCCACGATGGCGGCAAGCAGGCGC

TGGAGACGGTGCAGCGGCTGTTGCCGGTGCTGTGCCAGGGCCATGGCCTGACCCCGGACAAGGTGGTGGCCATCGCCAGC

AATAGTGGCGGCAAGCAGGCGCTGGAGACGGTGCAGCGGCTGTTGCCAGTGCTGTGCCAGGCCCATGGCCTGACCCCGGA

CAAGGTGGTGGCCATCGCCA

>41P1R_1

GGGCAACAATGCTCTCCAGCGCCTGCTTGCCGCCGCCATTGCTGGCGATGGCCACCACCTGGTCCGGGGTCAGGCCATGG

TCCTGGCACAGCACCGGCAACAGCCGCTGCACCGTCTCCAGCGCCTGCTTGCCGCCATCATGGCTGGCGATGGCCACGAC

CTGGTCCGGGCTCAGGCCATGGTCCTGGCACAGCACCGGCAACAGCCGCTGCACCGTCTCCAGCGCCTGCTTGCCGCCGC

CATTGCTGGCGATGGCCACCACCTGGTCCGGGGTCAGGCCATGGTCCTGGCACAGCACCGGCAACAGCCGCTGCACCGTC

TCCAGCGCCTGCTTGCCGCCAATATTGCTGGCGATGGCCACGACCTGGTCCGGGCTCAGGCCATGGTCCTGGCACAGCAC

CGGCAACAGCCGCTGCACCGTCTCCAGCGCCTGCTTGCCGCCGTTATTGTTGGCGATGGCCACCACCTGGGCCGGGGTCA

GGCCATGGTCCTGGCACAGCACCGGCAACAGCCGCTGCACCGTCTCCAGCGCCTGTTTGCCGCCATCGTGGCTGGCGATG

GCCACCACCTGGTCCAGGGTCAGGCCATGGTCCTGGCACAGCACCGGCAACAGCCGCTGCACCGTCTCCAGCGCCTGCTT

GCCGCCAATATTGCTGGCGATGGCCACCACCTGGTCCTGGGTCAGGCCATGGTCCTGGCACAGCACCGGCAACAGCCGCT

GCACCGTCTCCAGCGCCTGCTTGCCGCCGTTATTGTTGGCGATGGCCACCACCTGGTCCGGGGTCAGGCCATGGTCCTGG

CACAGTACCGGCAACAGCCGCTGCACCGTCTCCAGCGCCTGCTTGCCGCCATCGTGGCTGGCGATGGCCACGACCTGGTC

CGGGGTCAGGCCATGGTCCTGGCACAGCACCGGCAACAGCC

>41P1R_2

GGTGGCCATCGCCAACAATAACGGCGGCAAGCAGGCGCTGGAGACGGTGCGGCGGCTGTTGCCGGTGCTGTGCCAGGACC

ATGGCCTGACCCCGGACCAGGTCGTGGCCATCGCCAGCCACGATGGCGGCAAGCAGGCGCTGGAGACGGTGCAGCGGCTG

TTGCCGGTACTGTGCCAGGACCATGGCCTGACCCCGGACCAGGTGGTGGCCATCGCCAACAATAACGGCGGCAAGCAGGC

GCTGGAGACGGTGCAGCGGCTGTTGCCGGTGCTGTGCCAGGACCATGGCCTGACCCAGGACCAGGTGGTGGCCATCGCCA

GCAATATTGGCGGCAAGCAGGCGCTGGAGACGGTGCAGCGGCTGTTGCCGGTGCTGTGCCAGGACCATGGCCTGACCCTG

GACCAGGTGGTGGCCATCGCCAGCCACGATGGCGGCAAACAGGCGCTGGAGACGGTGCAGCGGCTGTTGCCGGTGCTGTG

CCAGGACCATGGCCTGACCCCGGCCCAGGTGGTGGCCATCGCCAACAATAACGGCGGCAAGCAGGCGCTGGAGACGGTGC

AGCGGCTGTTGCCGGTGCTGTGCCAGGACCATGGCCTGAGCCCGGACCAGGTCGTGGCCATCGCCAGCAATATTGGCGGC

AAGCAGGCGCTGGAGACGGTGCAGCGGCTGTTGCCGGTGCTGTGCCAGGACCATGGCCTGACCCCGGACCAGGTGGTGGC

CATCGCCAGCAATGGCGGCGGCAAGCAGGCGCTGGAGACGGTGCAGCGGCTGTTGCCGGTGCTGTGCCAGGACCATGGCC

TGAGCCCGGACCAGGTCGTGGCCATCGCCAGCCATGATGGCGGCAAGCAGGCGCTGGAGACGGTGCAGCGGCTGTTGCCG

GTGCTGTGCCAGGACCATGGCCTGACCCCGGACCAGGTGGTGGCCATCGCCAGCAATGGCGGCGGCAAGCAGGCGCTGGA

GAGCATTGTTGCCCAGT

>41P2F

CGCGCACATCGTTGCGCTCAGCCAACACCCGGCAGCGTTAGGGACCGTTGCTGTCACGTATCAGGACATAATCAGGGCGT

TGCCAGAGGCGACACACGAAGACATCGTTGGCGTCGGCAAACAGTGGTCCGGCGCACGCGCCCTGGAGGCCTTGCTCACG

GAGGCGGGGGAGTTGAGAGGTCCGCCGTTACAGTTGGACACAGGCCAACTTCTCAAGATTGCAAAACGTGGCGGCGTGAC

CGCAGTGGAGGCAGTGCATGCATGGCGCAATGCACTGACGGGTGCCCCCCTGAACCTGACCCCGGACCAAGTGGTGGCCA

TCGCCAGCAATATTGGCGGCAACCAGGCGCTGGAGACGGTGCAGCGGCTGTTGCCGGTGCTGTGCCAGGCCCATGGCCTG

ACCCCGGACCAGGTCGTGGCCATCGCCAGCCATGGCGGCGGCAAGCAGGCGCTGGAGACGGTGCAGCGGCTGTTGCCGGT

GCTGTGCCAGGACCATGGCCTGACCCCGGACCAGGTGGTGGCCATCGCCAGCAATATTGGCGGCAAGCAGGCGCTGGAGA

CGGTGCAACGGCTGTTGCCGGTGCTGTGCCAGGACCATGGCCTGACCCCGGACCAGGTGGTGGCCATCGCCAGCAATATT

GGCGGCAAGCAGGCGCTGGAGACGGTGCAGCGGCTGTTGCCGGTGCTGTGCCAGGACCATGGCCTGACCCCGGACCAGGT

GGTGGCCATCGCCAGCAATATTGGCGGCAAGCAGGCGCTGGAGACGGTGCAGCGGCTGTTGCCGGTGCTGTGCCAGGACC

ATGGCCTGACCCCGGACCAGGTGGTGGCCATCGCCAACAATAACGGCGGCAAGCAGGCGCTGGAGACGGTGCAACGGCTG

TTGCCGGTGCTGTGCCAGGACCATGGCCTGACCCCGGACCAGGTGGTGGCCATCGCCAGCCACGATGGCGGCAAGCAGGC

GCTGGAGACGGTGCAGCGG

>41P2R

TGTCCTGATACGTGACAGCAACGGTCCCTAACGCTGCCGGGTGTTGGCTGAGCGCAACGATGTGCGCGTGTGTAAACCCA

TGGCCCACCAGTGCCTCGTGGTGCTGCGCCACTGTCGAACGCACCTTCGGTTTGATCTTCTCTTGCTGCTGCTGACTGTA

GCCGAGCGTGCGTAGATCCACCTGCGCGGCCGGCGAAGCGTCGGAGGGTTGCGCCGCACGCCGTCGCGGGGCCGGCTTGG

CGCGCGGCGGCCGCGCGGCAGTGACAGCGACACGCACGGTGGGTGGCGGGTCATCGGCTGCACGCAGACCCGATTGCACC

TCATCGCACTCTGCTGGGGCAGCCGCTGTATGCGGCGTGCCGACGGCAGGCATCGAATCAAGAAGCGATGTATCAAGAAG

CGACGGATCGAACTGACGGAGCAGATCGCTGAAGCTGCCCGCCGAGAACGCAGGCGAGGGCGCAGGGGGAGATGGCAGCC

GGGTCCGGGACATCGTCCGCCGAGCGGGCAAGCCATCCAGGGGGCCGCCAGCAGGCGGAGCCCCCCCCCGATCTGCAGTC

GGCTGAACCCTATCCGGTTGGGGTCCGGGCAGAAGCTCGCGGGCAGGACTTGGCGTACGCGAACGAATGGGATCC

>41P3F

GCCGCACGCGCCGGAATTGATCAGAAGAGTCAATAGCCGTATTGGCGAACGCACGTCCCATCGCGTTGCCGACTACGCGC

AAGTGGTTCGCGTGCTGGAGTTTTTCCAGTGCCACTCCCACCCAGCGTACGCATTTGATGAGGCCATGACGCAGTTCGGG

ATGAGCAGGAACGGGTTGGTACAGCTCTTTCGCAGAGTGGGCGTCACCGAACTCGAAGCCCGCTGCGGAACGCTCCCCCC

AGCCTCGCAGCGTTGGGACCGTATCCTCCAGGCATCAGGGATGAAAAGGGCCAAACCGTCCCCTACTTCAGCTCAAACAC

CGGATCAGGCGTCTTTGCATGCATTCGCCGATTCGCTGGAGCGTGACCTTGATGCGCCCAGCCCAATGCACGAGGGAGAT

CAGACGCGGGCAAGCAGCCGTAAACGGTCCCGATCGGATCGTGCTGTCACCGGCCCCTCCGCACAGCAATCTTTCGAGGT

GCGCGTTCCCGAACAGCGCGATGCGCTGCATTTGCCCCTCAGCTGGAGGGTAAAACGCCCGCGTACCAGGATCGGGGGCG

GCCTCCCGGATCC

>41P3R

GGGCTTCGAGTTCGGTGACGCCCACTCTGCGAAAGAGCTGTACCAACCCGTTCCTGCTCATCCCGAACTGCGTCATGGCC

TCATCAAATGCGTACGCTGGGTGGGAGTGGCACTGGAAAAACTCCAGCACGCGAACCACTTGCGCGTAGTCGGCAACGCG

ATGGGACGTGCGTTCGCCAATACGGCTATTGACTCTTCTGATCAATTCCGGCGCGTGCGGCAATCCCTTTTTCACTGCAT

CCAGGGCAGGACGTCCGCCGAGGCAGGCCAAGGCGACGAGGTGGTCGTTGGTCAACGCGGCCAACGCCGGATCAGGGCGA

GATAACTGGGCAACAATGCTCTCCAGCGCCTGCTTGCCGCCGCCATTGCTGGCGATGGCCACCACCTGGTCCGGGGTCAG

GCCATGGTCCTGGCACAGCACCGGCAACAGCCGCTGCACCGTCTCCAGCGCCTGCTTGCCGCCATCATGGCTGGCGATGG

CCACGACCTGGTCCGGGCTCAGGCCATGGTCCTGGCACAGCACCGGCAACAGCCGCTGCACCGTCTCCAGCGCCTGCTTG

CCGCCGCCATTGCTGGCGATGGCCACCACCTGGTCCGGGGTCAGGCCATGGTCCTGGCACAGCACCGGCAACAGCCGCTG

CACCGTCTCCAGCGCCTGCTTGCCGCCAATATTGCTGGCGATGGCCACGACCTGGTCCGGGCTCAGGCCATGGTCCTGGC

ACAGCACCGGCAACAGCCGCTGCACCGTCTCCAGCGCCTGCTTGCCGCCGTTATTGTTGGCGATGGCCACCACCTGGGCC

GGGGTCAGGCCATGGTCCTGGCACAGCACCGGCAACAGCCGCTGCACCGTCTCCAGCGCCTGTTTGCCGCCATCGTGGCT

GGCGATGGCCACCACCTGGTCCAGGGTCAGGCCATGGTCCTGGCACAGCACCGGCAACAGCCGCTGCACCGTCTCCAGCG

CCTGCTTGCCGCCAATATTGCTGG

>M13F

GGATCCCATTCGTTCGCGTACGCCAAGTCCTGCCCGCGAGCTTCTGCCCGGACCCCAACCGGATAGGGTTCAGCCGACTG

CAGATCGGGGGGGGGCTCCGCCTGCTGGCGGCCCCCTGGATGGCTTGCCCGCTCGGCGGACGATGTCCCGGACCCGGCTG

CCATCTCCCCCTGCGCCCTCGCCTGCGTTCTCGGCGGGCAGCTTCAGCGATCTGCTCCGTCAGTTCGATCCGTCGCTTCT

TGATACATCGCTTCTTGATTCGATGCCTGCCGTCGGCACGCCGCATACAGCGGCTGCCCCAGCAGAGTGCGATGAGGTGC

AATCGGGTCTGCGTGCAGCCGATGACCCGCCACCCACCGTGCGTGTCGCTGTCACTGCCGCGCGGCCGCCGCGCGCCAAG

CCGGCCCCGCGACGGCGTGCGGCGCAACCCTCCGACGCTTCGCCGGCCGCGCAGGTGGATCTACGCACGCTCGGCTACAG

TCAGCAGCAGCAAGAGAAGATCAAACCGAAGGTGCGTTCGACAGTGGCGCAGCACCACGAGGCACTGGTGGGCCATGGGT

TTACACACGCGCACATCGTTGCGCTCAGCCAACACCCGGCAGCGTTAGGGACCGTTGCTGTCACGTATCAGGACATAATC

AGGGCGTTGCCAGAGGCGACACACGAAGACATCGTTGGCGTCGGCAAACAGTGGTCCGGCGCACGCGCCCTGGAGGCCTT

GCTCACGGAGGCGGGGGAGTTGAGAGGTCCGCCGTTACAGTTGGACACAGGCCAACTTCTCAAGATTGCAAAACGTGGCG

GCGTGACCGCAGTGGAGGCAGTGCATGCATGGCGCAATGCACTGACGGGTGCCCCCCTGAACCTGACCCCGGACCAAGTG

GTGGCCATCGCCAGCAATATTGGCGGCAACCAGGCGCTGGAGA

>M13R

GACCAGGTGGTGGCCATCGCCAGCAATGGCGGCGGCAAGCAGGCGCTGGAGACGGTGCAGCGGCTGTTGCCGGTGCTGTG

CCAGGACCATGGCCTGAGCCCGGACCAGGTCGTGGCCATCGCCAGCCATGATGGCGGCAAGCAGGCGCTGGAGACGGTGC

AGCGGCTGTTGCCGGTGCTGTGCCAGGACCATGGCCTGACCCCGGACCAGGTGGTGGCCATCGCCAGCAATGGCGGCGGC

AAGCAGGCGCTGGAGAGCATTGTTGCCCAGTTATCTCGCCCTGATCCGGCGTTGGCCGCGTTGACCAACGACCACCTCGT

CGCCTTGGCCTGCCTCGGCGGACGTCCTGCCCTGGATGCAGTGAAAAAGGGATTGCCGCACGCGCCGGAATTGATCAGAA

GAGTCAATAGCCGTATTGGCGAACGCACGTCCCATCGCGTTGCCGACTACGCGCAAGTGGTTCGCGTGCTGGAGTTTTTC

CAGTGCCACTCCCACCCAGCGTACGCATTTGATGAGGCCATGACGCAGTTCGGGATGAGCAGGAACGGGTTGGTACAGCT

CTTTCGCAGAGTGGGCGTCACCGAACTCGAAGCCCGCTGCGGAACGCTCCCCCCAGCCTCGCAGCGTTGGGACCGTATCC

TCCAGGCATCAGGGATGAAAAGGGCCAAACCGTCCCCTACTTCAGCTCAAACACCGGATCAGGCGTCTTTGCATGCATTC

GCCGATTCGCTGGAGCGTGACCTTGATGCGCCCAGCCCAATGCACGAGGGAGATCAGACGCGGGCAAGCAGCCGTAAACG

GTCCCGATCGGATCGTGCTGTCACCGGCCCCTCCGCACAGCAATCTTTCGAGGTGCGCGTTCCCGAACAGCGCGATGCGC

TGCATTTGCCCCTCAGCTGGAGGGTAAAACGCCCGCGTACCAGGATCGGGGGCGGCCTCCCGGATCC

**pTAL*_Bam_*_HI_-47**

> pCC2FOS-MscI-1

TGGCACAGCACCGGCAACAGCCGCTGCACCGTCTCCAGCGCCTGCTTGCCGCCAATATTGCTGGCGATGGCCACCACCTG

GTCCGGGGTCAGGCCATGGTCCTGGCACAGCACCGGCAACAGCCGCTGCACCGTCTCCAGCGCCTGCTTGCCGCCAATAT

TGCTGGCGATGGCCACGACCTGGTCCGGGGTCAGGCCATGGTCCTGGCACAGCACCGGCAACAGCCGTTGCACCGTCTCC

AGCGCCTGCTTGCCGCCGTTATTGCTGGCGATGGTCACCACCTGGTCCGTGGTCAGGCCATGGGCCTGGCACAGCACCGG

CAACAGCCGCTGCACCGTCTCCAGCGCCTGCTTGCCGCCGTTATTGTTGGCGATGGCCACCACCTGGTCCGGGATCAGGC

CATGGTCCTGGCACAGCACCGGCAACAGCCGCTGCACCGTCTCCAGCGCCTGCTTGCCGCCGTTATTGCTGGCGATGGCC

ACCACCTGGTCCGGGGTCAGGCCATGGTCCTGGCACAGCACCGGCAACAGCCGCTGCACCGTCTCCAGCGCCTGCTTGCC

GCCGCCATGG

> pCC2FOS-MscI-2

CCAGGCCCATGGCCTGACCCCGGCGCAGGTGGTGGCCATCGCCAGCAATAACGGCGGCAAGCAGGCGCTGGAGACGGTGC

AGCGGCTGTTGCCGGTGCTGTGCCAGGACCATGGCCTGACCCCGGACCAAGTGGTGGCCATCGCCAGCAATATTGGCGGC

AAGCAGGCGCTGGAGACGGTGCAACGGCTGTTGCCGGTGCTGTGCCAGGACCATGGCCTGACCCCGGACCAGGTCGTGGC

CATCGCCAGCAATATTGGCGGCAAGCAGGCGCTGGAGACGGTGCAGCGGCTGTTGCCGGTGCTGTGCCAGACCCATGCCC

TGACCCCGGACCAGGTGGTGGCCATCGCCAGCAATATTGGCGGCAAGCAGGCGCTGGAGACGGTGCAGCGGCTGTTGCCG

GTGCTGTGCCAGGACCACGGCCTGACCCCGGCGCAGGTGGTGGCCATCGCCAGCCACGATGGCGGCAAGCAGGCGCTGGA

GACGGTGCAGCGGCTGTTGCCGGTGCTGTGCCAGGACCATGGCCTGACCCCGGACCAGGTGGTGGCCATCGCCAGCAATA

GTGGCGGCAAGCAGGCGCTGGAGACGGTGCAGCGGCTGTTGCCGGTGCTGTGCCAGGCCCATGGCCTGACCCTGGACCAG

GTGGTGGCCATCGCCAGCCATGGCGGCGGCAAGCAGGCGCTGGAGACGGTGCAGCGGCTGTTGCCGGTGCTGTGCCAGGA

CCATGGCCTGACCCCGGACCAGGTGGTGGCCATCGCCAGCAATAACGGCGGCAAGCAGGCGCTGGAGACGGTGCAGCGGC

TGTTGCCGGTGCTGTGCC

> pCC2FOS-MscI-3

CCATCGCCAGCAATATTGGCGGCAAGCAGGCGCTGGAGACGGTGCAGCGGCTGTTGCCGGTGCTGTGCCAGGCCCATGGC

CTGACCCCGGACCAGGTGGTGGCCATCGCCAACAATAACGGCGGCAAGCAGGCACTGGAGACGGTGCAGCGGCTGTTGCC

GGTGCTGTGCCAGGCCCATGGCCTGACCCCGGCGCAGGTGGTGGCCATCGCCAGCAATAACGGCGGCAAGCAGGCGCTGG

AGACGGTGCAGCGGCTGTTGCCGGTGCTGTGCCAGGACCATGGCCTGACCCCGGACCAAGTGGTGGCCATCGCCAGCAAT

ATTGGCGGCAAGCAGGCGCTGGAGACGGTGCAACGGCTGTTGCCGGTGCTGTGCCAGGACCATGGCCTGACCCCGGACCA

GGTCGTGGCCATCGCCAGCAATATTGGCGGCAAGCAGGCGCTGGAGACGGTGCAGCGGCTGTTGCCGGTGCTGTGCCAGA

CCCATGCCCTGACCCCGGACCAGGTGGTGGCCATCGCCAGCAATATTGGCGGCAAGCAGGCGCTGGAGACGGTGCAGCGG

CTGTTGCCGGTGCTGTGCCAGGACCACGGCCTGACCCCGGCGCAGGTGGTGGCCATCGCCAGCCACGATGGCGGCAAGCA

GGCGCTGGAGACGGTGCAGCGGCTGTTGCCGGTGCTGTGCCAGGACCATGGCCTGACCCCGGACCAGGTGGTGGCCATCG

CCAGCAATAGTGGCGGCAAGCAGGCGCTGGAGACGGTGCAGCGGCTGTTGCCGGTGCTGTGCCAGGCCCATGGCCTGACC

CTGGACCAGGTGGTGGCCATCGCCAGCCATGGCGGCGGCAAGCAGGCGCTGGAGACGGTGCAGCGGCTGTTGCCGGTG

> pCC2FOS-MscI-4

GGTGGCCATCGCCAGCAATATTGGCGGCAAGCAGGCGCTGGAGACGGTGCAGCGGCTGTTGCCGGTGCTGTGCCAGGACC

ACGGCCTGACCCCGGCGCAGGTGGTGGCCATCGCCAGCCACGATGGCGGCAAGCAGGCGCTGGAGACGGTGCAGCGGCTG

TTGCCGGTGCTGTGCCAGGACCATGGCCTGACCCCGGACCAGGTGGTGGCCATCGCCAGCAATAGTGGCGGCAAGCAGGC

GCTGGAGACGGTGCAGCGGCTGTTGCCGGTGCTGTGCCAGGCCCATGGCCTGACCCTGGACCAGGTGGTGGCCATCGCCA

GCCATGGCGGCGGCAAGCAGGCGCTGGAGACGGTGCAGCGGCTGTTGCCGGTGCTGTGCCAGGACCATGGCCTGACCCCG

GACCAGGTGGTGGCCATCGCCAGCAATAACGGCGGCAAGCAGGCGCTGGAGACGGTGCAGCGGCTGTTGCCGGTGCTGTG

CCAGGACCATGGCCTGATCCCGGACCAGGTGGTGGCCATCGCCAACAATAACGGCGGCAAGCAGGCGCTGGAGACGGTGC

AGCGGCTGTTGCCGGTGCTGTGCCAGGCCCATGGCCTGACCACGGACCAGGTGGTGACCATCGCCAG

> pCC2FOS-MscI-5

CAAGCAGGCGCTGGAGACGGTGCAGCGGCTGTTGCCGGTGCTGTGCCAGGACCATGGCCTGATCCCGGACCAGGTGGTGG

CCATCGCCAACAATAACGGCGGCAAGCAGGCGCTGGAGACGGTGCAGCGGCTGTTGCCGGTGCTGTGCCAGGCCCATGGC

CTGACCACGGACCAGGTGGTGACCATCGCCAGCAATAACGGCGGCAAGCAGGCGCTGGAGACGGTGCAACGGCTGTTGCC

GGTGCTGTGCCAGGACCATGGCCTGACCCCGGACCAGGTCGTGGCCATCGCCAGCAATATTGGCGGCAAGCAGGCGCTGG

AGACGGTGCAGCGGCTGTTGCCGGTGCTGTGCCAGGACCATGGCCTGACCCCGGACCAGGTGGTGGCCATCGCCAGCAAT

ATTGGCGGCAAGCAGGCGCTGGAGACGGTGCAGCGGCTGTTGCCGGTGCTGTGCCAGGACCATGGCCTGACCCCGGACCA

GGTCGTGGCCATCGCCAGCAATGGCGGCGGCAAGCAGGCGCTGGAGACGGTGCAGCGGCTGTTGCCGGTGCTGTGCCA

> pCC2FOS-MscI-6

CGGCAAGCAGGCGCTGGAGACGGTGCAGCGGCTGTTGCCGGTGCTGTGCCAGGACCACGGCCTGACCCCGGCGCAGGTGG

TGGCCATCGCCAGCCACGATGGCGGCAAGCAGGCGCTGGAGACGGTGCAGCGGCTGTTGCCGGTGCTGTGCCAGGACCAT

GGCCTGACCCCGGACCAGGTGGTGGCCATCGCCAGCAATAGTGGCGGCAAGCAGGCGCTGGAGACGGTGCAGCGGCTGTT

GCCGGTGCTGTGCCAGGCCCATGGCCTGACCCTGGACCAGGTGGTGGCCATCGCCAGCCATGGCGGCGGCAAGCAGGCGC

TGGAGACGGTGCAGCGGCTGTTGCCGGTGCTGTGCCAGGACCATGGCCTGACCCCGGACCAGGTGGTGGCCATCGCCAGC

AATAACGGCGGCAAGCAGGCGCTGGAGACGGTGCAGCGGCTGTTGCCGGTGCTGTGCCAGGACCATGGCCTGATCCCGGA

CCAGGTGGTGGCCATCGCCAACAATAACGGCGGCAAGCAGGCGCTGGAGACGGTGCAGCGGCTGTTGCCGGTGCTGTGCC

AGGCCCATGGCCTGACCACGGACCAGGTGGTGACCATCGCCAGCAATAACGGCGGCAAGCAGGCGCTGGAGACGGTGCAA

CGGCTGTTGCCGGTGCTGTGCCAGGACCATGGCCTGACCCCGGACCAGGTCGTGGCCATCGCCAGCAATATTGGCGGCAA

GCAGGCGCTGGAGACGGTGCAGCGGCTGTTGCCGGTGCTGT

> pCC2FOS-MscI-7

GTGGCCATCGCCAGCAATATTGGCGGCAAGCAGGCGCTGGAGACGGTGCAGCGGCTGTTGCCGGTGCTGTGCCAGGACCA

CGGCCTGACCCCGGCGCAGGTGGTGGCCATCGCCAGCCACGATGGCGGCAAGCAGGCGCTGGAGACGGTGCAGCGGCTGT

TGCCGGTGCTGTGCCAGGACCATGGCCTGACCCCGGACCAGGTGGTGGCCATCGCCAGCAATAGTGGCGGCAAGCAGGCG

CTGGAGACGGTGCAGCGGCTGTTGCCGGTGCTGTGCCAGGCCCATGGCCTGACCCTGGACCAGGTGGTGGCCATCGCCAG

CCATGGCGGCGGCAAGCAGGCGCTGGAGACGGTGCAGCGGCTGTTGCCGGTGCTGTGCCAGGACCATGGCCTGACCCCGG

ACCAGGTGGTGGCCATCGCCAGCAATAACGGCGGCAAGCAGGCGCTGGAGACGGTGCAGCGGCTGTTGCCGGTGCTGTGC

CAGGACCATGGCCTGATCCCGGACCAGGTGGTGGCCATCGCCAACAATAACGGCGGCAAGCAGGCGCTGGAGACGGTGCA

GCGGCTGTTGCCGGTGCTGTGCCAGGCCCATGGCCTGACCACGGACCAGGTGGTGACCATCGCCAGCAATAACGGCGGCA

AGCAGGCGCTGGAGACGGTGCAACGGCTGTTGCCGGTGCTGTGCCAGGACCATGGCCTGACCCCGGACCAGGTCGTGGCC

ATCGCCAGCAATATTGGCGGCAAGCAGGCGCTGGAGACGGTGCAGCGGCTGTTGCCGGTGCTGTGCCAGGACCATGGCCT

GACCCCGGACCAGGTGGTGGCCATCGCCAGCAATATTGGCGGCAAGCAGGCGCTGGAGACGGTGCAGCGGCTGTTGCCGG

TGCTGTGCCAGG

>47P1F_1

CGGGGGAGTTGAGAGGTCCGCCGTTACAGTTGGACACAGGCCAACTTCTCAAGATTGCAAAACGTGGCGGCGTGACCGCA

GTGGAGGCAGTGCATGCATGGCGCAATGCACTGACGGGTGCCCCCCTGAACCTGACCCCGGACCAAGTGGTGGCCATCGC

CAGCAATATTGGCGGCAAGCAGGCGCTGGAGACGGTGCAGCGGCTGTTGCCGGTGCTGTGCCAGGCCCATGGCCTGACCC

CGGACCAGGTGGTGGCCATCGCCAACAATAACGGCGGCAAGCAGGCACTGGAGACGGTGCAGCGGCTGTTGCCGGTGCTG

TGCCAGGCCCATGGCCTGACCCCGGCGCAGGTGGTGGCCATCGCCAGCAATAACGGCGGCAAGCAGGCGCTGGAGACGGT

GCAGCGGCTGTTGCCGGTGCTGTGCCAGGACCATGGCCTGACCCCGGACCAAGTGGTGGCCATCGCCAGCAATATTGGCG

GCAAGCAGGCGCTGGAGACGGTGCAACGGCTGTTGCCGGTGCTGTGCCAGGACCATGGCCTGACCCCGGACCAGGTCGTG

GCCATCGCCAGCAATATTGGCGGCAAGCAGGCGCTGGAGACGGTGCAGCGGCTGTTGCCGGTGCTGTGCCAGACCCATGC

CCTGACCCCGGACCAGGTGGTGGCCATCGCCAGCAATATTGGCGGCAAGCAGGCGCTGGAGACGGTGCAGCGGCTGTTGC

CGGTGCTGTGCCAGGACCACGGCCTGACCCCGGCGCAGGTGGTGGCCATCGCCAGCCACGATGGCGGCAAGCAGGCGCTG

GAGACGGTGCAGCGGCTGTTGCCGGTGCTGTGCCAGGACCATGGCCTGACCCCGGACCAGGTGGTGGCCATCGCCAGCAA

TAGTGGCGGCAAGCAGGCGCTGGAGACGGTGCAGCGGCTGTTGCCGGTGCTGTGCCAGGCCCATGGCCTGACCCTGGACC

AGGTGGTGGCCATCGCCAG

>47P1F_2

GGGGGAGTTGAGAGGTCCGCCGTTACAGTTGGACACAGGCCAACTTCTCAAGATTGCAAAACGTGGCGGCGTGACCGCAG

TGGAGGCAGTGCATGCATGGCGCAATGCACTGACGGGTGCCCCCCTGAACCTGACCCCGGACCAAGTGGTGGCCATCGCC

AGCAATATTGGCGGCAAGCAGGCGCTGGAGACGGTGCAGCGGCTGTTGCCGGTGCTGTGCCAGGCCCATGGCCTGACCCC

GGACCAGGTGGTGGCCATCGCCAACAATAACGGCGGCAAGCAGGCACTGGAGACGGTGCAGCGGCTGTTGCCGGTGCTGT

GCCAGGCCCATGGCCTGACCCCGGCGCAGGTGGTGGCCATCGCCAGCAATAACGGCGGCAAGCAGGCGCTGGAGACGGTG

CAGCGGCTGTTGCCGGTGCTGTGCCAGGACCATGGCCTGACCCCGGACCAAGTGGTGGCCATCGCCAGCAATATTGGCGG

CAAGCAGGCGCTGGAGACGGTGCAACGGCTGTTGCCGGTGCTGTGCCAGGACCATGGCCTGACCCCGGACCAGGTCGTGG

CCATCGCCAGCAATATTGGCGGCAAGCAGGCGCTGGAGACGGTGCAGCGGCTGTTGCCGGTGCTGTGCCAGACCCATGCC

CTGACCCCGGACCAGGTGGTGGCCATCGCCAGCAATATTGGCGGCAAGCAGGCGCTGGAGACGGTGCAGCGGCTGTTGCC

GGTGCTGTGCCAGGACCACGGCCTGACCCCGGCGCAGGTGGTGGCCATCGCCAGCCACGATGGCGGCAAGCAGGCGCTGG

AGACGGTGCAGCGGCTGTTGCCGGTGCTGTGCCAGGACCATGGCCTGACCCCGGACCAGGTGGTGGCCATCGCCAGCAAT

AGTGGCGGCAAGCAGGCGCTGGAGACGGTGCAGCGGCTGTTGCCGGTGCTGTGCCAGGCCCATGGCCTGACCCTGGACCA

GGTGGTGGCCATCGC

>47P1R_1

GGGCAACAATGCTCTCCAGCGCCTGCTTGCCACCATCGTGGCTGGCGATGGCCACCACCTGGTCCGGGGTCAGGCCATGG

TCCTGGCACAGCACCGGCAACAGCCGCTGCACCGTCTCCAGCGCCTGCTTGCCGCCGCCATTGCTGGCGATGGCCACGAC

CTGGTCCGGGGTCAGGCCATGGTCCTGGCACAGCACCGGCAACAGCCGCTGCACCGTCTCCAGCGCCTGCTTGCCGCCAA

TATTGCTGGCGATGGCCACCACCTGGTCCGGGGTCAGGCCATGGTCCTGGCACAGCACCGGCAACAGCCGCTGCACCGTC

TCCAGCGCCTGCTTGCCGCCAATATTGCTGGCGATGGCCACGACCTGGTCCGGGGTCAGGCCATGGTCCTGGCACAGCAC

CGGCAACAGCCGTTGCACCGTCTCCAGCGCCTGCTTGCCGCCGTTATTGCTGGCGATGGTCACCACCTGGTCCGTGGTCA

GGCCATGGGCCTGGCACAGCACCGGCAACAGCCGCTGCACCGTCTCCAGCGCCTGCTTGCCGCCGTTATTGTTGGCGATG

GCCACCACCTGGTCCGGGATCAGGCCATGGTCCTGGCACAGCACCGGCAACAGCCGCTGCACCGTCTCCAGCGCCTGCTT

GCCGCCGTTATTGCTGGCGATGGCCACCACCTGGTCCGGGGTCAGGCCATGGTCCTGGCACAGCACCGGCAACAGCCGCT

GCACCGTCTCCAGCGCCTGCTTGCCGCCGCCATGGCTGGCGATGGCCACCACCTGGTCCAGGGTCAGGCCATGGGCCTGG

CACAGCACCGGCAACAGCCGCTGCACCGTCTCCAGCGCCTGCTTGCCGCCACTATTGCTGGCGATGGCCACCACCTGGTC

CGGGGTCAGGCCATGGTCCTGGCACAGCACCGGCAACAGCCGCTGCACCGTCTCCAGCGCCTGCTTGCCGCCATC

>47P1R_2

CATCGCCAGCCACGATGGCGGCAAGCAGGCGCTGGAGACGGTGCAGCGGCTGTTGCCGGTGCTGTGCCAGGACCATGGCC

TGACCCCGGACCAGGTGGTGGCCATCGCCAGCAATAGTGGCGGCAAGCAGGCGCTGGAGACGGTGCAGCGGCTGTTGCCG

GTGCTGTGCCAGGCCCATGGCCTGACCCTGGACCAGGTGGTGGCCATCGCCAGCCATGGCGGCGGCAAGCAGGCGCTGGA

GACGGTGCAGCGGCTGTTGCCGGTGCTGTGCCAGGACCATGGCCTGACCCCGGACCAGGTGGTGGCCATCGCCAGCAATA

ACGGCGGCAAGCAGGCGCTGGAGACGGTGCAGCGGCTGTTGCCGGTGCTGTGCCAGGACCATGGCCTGATCCCGGACCAG

GTGGTGGCCATCGCCAACAATAACGGCGGCAAGCAGGCGCTGGAGACGGTGCAGCGGCTGTTGCCGGTGCTGTGCCAGGC

CCATGGCCTGACCACGGACCAGGTGGTGACCATCGCCAGCAATAACGGCGGCAAGCAGGCGCTGGAGACGGTGCAACGGC

TGTTGCCGGTGCTGTGCCAGGACCATGGCCTGACCCCGGACCAGGTCGTGGCCATCGCCAGCAATATTGGCGGCAAGCAG

GCGCTGGAGACGGTGCAGCGGCTGTTGCCGGTGCTGTGCCAGGACCATGGCCTGACCCCGGACCAGGTGGTGGCCATCGC

CAGCAATATTGGCGGCAAGCAGGCGCTGGAGACGGTGCAGCGGCTGTTGCCGGTGCTGTGCCAGGACCATGGCCTGACCC

CGGACCAGGTCGTGGCCATCGCCAGCAATGGCGGCGGCAAGCAGGCGCTGGAGACGGTGCAGCGGCTGTTGCCGGTGCTG

TGCCAGGACCATGGCCTGACCCCGGACCAGGTGGTGGCCATCGCCAGCCACGATGGTGGCAAGCAGGCGCTGGAGAGCAT

TGTTGCCCAGTTATCTCG

>47P2F

CGCGCACATCGTTGCGCTCAGCCAACACCCGGCAGCGTTAGGGACCGTCGCTGTCAAGTATCAGCACATAATCACGGCGT

TGCCAGAGGCGACACACGAAGACATCGTTGGCGTCGGCAAACAGTGGTCCGGCGCACGCGCCCTGGAGGCCTTGCTCACG

AAGGCGGGGGAGTTGAGAGGTCCGCCGTTACAGTTGGACACAGGCCAACTTCTCAAGATTGCAAAACGTGGCGGCGTGAC

CGCAGTGGAGGCAGTGCATGCATGGCGCAATGCACTGACGGGTGCCCCCCTGAACCTGACCCCGGACCAAGTGGTGGCCA

TCGCCAGCAATATTGGCGGCAAGCAGGCGCTGGAGACGGTGCAGCGGCTGTTGCCGGTGCTGTGCCAGGCCCATGGCCTG

ACCCCGGACCAGGTGGTGGCCATCGCCAACAATAACGGCGGCAAGCAGGCACTGGAGACGGTGCAGCGGCTGTTGCCGGT

GCTGTGCCAGGCCCATGGCCTGACCCCGGCGCAGGTGGTGGCCATCGCCAGCAATAACGGCGGCAAGCAGGCGCTGGAGA

CGGTGCAGCGGCTGTTGCCGGTGCTGTGCCAGGACCATGGCCTGACCCCGGACCAAGTGGTGGCCATCGCCAGCAATATT

GGCGGCAAGCAGGCGCTGGAGACGGTGCAACGGCTGTTGCCGGTGCTGTGCCAGGACCATGGCCTGACCCCGGACCAGGT

CGTGGCCATCGCCAGCAATATTGGCGGCAAGCAGGCGCTGGAGACGGTGCAGCGGCTGTTGCCGGTGCTGTGCCAGACCC

ATGCCCTGACCCCGGACCAGGTGGTGGCCATCGCCAGCAATATTGGCGGCAAGCAGGCGCTGGAGACGGTGCAGCGGCTG

TTGCCGGTGCTGTGCCAGGACCACGGCCTGACCCCGGCGCAGGTGGTGGCCATCGCCAGCCACGATGGCGGCAAGCAGGC

GCTGGAGACGGTGCA

>47P2R

GTGATTATGTGCTGATACTTGACAGCGACGGTCCCTAACGCTGCCGGGTGTTGGCTGAGCGCAACGATGTGCGCGTGTGT

AAACCCATGGCCCACCAGTGCCTCGTGGTGCTGCGCCACTGTCGAACGCACCTTCGGTTTGATCTTCTCTTGCTGCTGCT

GACTGTAGCCGAGCGTGCGTAGATCCACCTGCGCGGCCGGCGAAGCGTCGGAGGGTTGCGCCGCACGCCGTCGCGGGGCC

GGCTTGGCGCGCGGCGGCCGCGCGGCAGTGACAGCGACACGCACGGTGGGTGGCGGGTCATCGGCTGCACGCAGACCCGA

TTGCATCTCATCCCACTCTGCTGGGGCAGCCGCTGTATGCGGCGTGCCGACGGCAGGCATCGAATCAAGAAGCGATGTAT

CGACTTGACGGAGCAGATCGCTGAAGCTGCCCGCCGGGAACGCAGGCGAGGGCGCAGGGGGAGATGGCAGCCGGGTCCGG

GACATCGTCCGCCGAGCGGGCAAGCCATCCAGGGGGCCGCCAGCAGGCGGAGCCCCCCCCCGATCTGCAGTCGGCTGAAC

CCTATCCGGTTGGGGTCCGGGCAGAAGCTCGCGGGCAGGACTTGGCGTACGCGAACGAATGGGATCC

>47P3F

GGATTGCCGCACGCGCCGGAATTGATCAGAAGAATCAATCGCCGTATTCCCGAACGCACGTCCCATCGCGTTGCCGACTA

CGCGCAAGTGGTTCGCGTGCTGGAGTTTTTCCAGTGCCACTCCCACCCAGCGTACGCATTTGATGAGGCCATGACGCAGT

TCGGGATGAGCAGGAACGGGTTGGTACAGCTCTTTCGCAGAGTGGGCGTCACCGAACTCGAAGCCCGCGGTGGAACGCTC

CCCCCAGCCTCGCAGCGTTGGGACCGTATCCTCCAGGCATCAGGGATGAAAAGGGCCAAACCGTCCCCTACTTCAGCTCA

AACACCGGATCAGGCGTCTTTGCATGCATTCGCCGATTCGCTGGAGCGTGACCTTGATGCGCCCAGCCCAATGCACGAGG

GAGATCAGACGCGGGCAAGCAGCCGTAAACGGTCCCGATCGGATCGTGCTGTCACCGGCCCCTCCACACAGCAATCTTTC

GAGGTGCGCGTTCCCGAACAGCGCGATGCGCTGCATTTGCCCCTCAGCTGGAGGGTAAAACGCCCGCGTACCAGGATCGG

GGGCGGCCTCCCGGATCC

>47P3R

CTTCGAGTTCGGTGACGCCCACTCTGCGAAAGAGCTGTACCAACCCGTTCCTGCTCATCCCGAACTGCGTCATGGCCTCA

TCAAATGCGTACGCTGGGTGGGAGTGGCACTGGAAAAACTCCAGCACGCGAACCACTTGCGCGTAGTCGGCAACGCGATG

GGACGTGCGTTCGGGAATACGGCGATTGATTCTTCTGATCAATTCCGGCGCGTGCGGCAATCCCTTTTTCACTGCATCCA

GGGCAGGACGTCCGCCGAGGCAGGCCAAGGCGACGAGGTGGTCGTTGGTCAACGCGGCCAACGCCGGATCAGGGCGAGAT

AACTGGGCAACAATGCTCTCCAGCGCCTGCTTGCCACCATCGTGGCTGGCGATGGCCACCACCTGGTCCGGGGTCAGGCC

ATGGTCCTGGCACAGCACCGGCAACAGCCGCTGCACCGTCTCCAGCGCCTGCTTGCCGCCGCCATTGCTGGCGATGGCCA

CGACCTGGTCCGGGGTCAGGCCATGGTCCTGGCACAGCACCGGCAACAGCCGCTGCACCGTCTCCAGCGCCTGCTTGCCG

CCAATATTGCTGGCGATGGCCACCACCTGGTCCGGGGTCAGGCCATGGTCCTGGCACAGCACCGGCAACAGCCGCTGCAC

CGTCTCCAGCGCCTGCTTGCCGCCAATATTGCTGGCGATGGCCACGACCTGGTCCGGGGTCAGGCCATGGTCCTGGCACA

GCACCGGCAACAGCCGTTGCACCGTCTCCAGCGCCTGCTTGCCGCCGTTATTGCTGGCGATGGTCACCACCTGGTCCGTG

GTCAGGCCATGGGCCTGGCACAGCACCGGCAACAGCCGCTGCACCGTCTCCAGCGCCTGCTTGCCGCCGTTATTGTTGGC

GATGGCCACCACCTGGTCCGGGATCAGGCCATGGTCCTGGCACAGCACCGGCAACAGCCGCTGCACCGTCTCCAGCGCCT

GCTTGCCGCCGTTATTGCTG

>M13F

GGATCCCATTCGTTCGCGTACGCCAAGTCCTGCCCGCGAGCTTCTGCCCGGACCCCAACCGGATAGGGTTCAGCCGACTG

CAGATCGGGGGGGGGCTCCGCCTGCTGGCGGCCCCCTGGATGGCTTGCCCGCTCGGCGGACGATGTCCCGGACCCGGCTG

CCATCTCCCCCTGCGCCCTCGCCTGCGTTCCCGGCGGGCAGCTTCAGCGATCTGCTCCGTCAAGTCGATACATCGCTTCT

TGATTCGATGCCTGCCGTCGGCACGCCGCATACAGCGGCTGCCCCAGCAGAGTGGGATGAGATGCAATCGGGTCTGCGTG

CAGCCGATGACCCGCCACCCACCGTGCGTGTCGCTGTCACTGCCGCGCGGCCGCCGCGCGCCAAGCCGGCCCCGCGACGG

CGTGCGGCGCAACCCTCCGACGCTTCGCCGGCCGCGCAGGTGGATCTACGCACGCTCGGCTACAGTCAGCAGCAGCAAGA

GAAGATCAAACCGAAGGTGCGTTCGACAGTGGCGCAGCACCACGAGGCACTGGTGGGCCATGGGTTTACACACGCGCACA

TCGTTGCGCTCAGCCAACACCCGGCAGCGTTAGGGACCGTCGCTGTCAAGTATCAGCACATAATCACGGCGTTGCCAGAG

GCGACACACGAAGACATCGTTGGCGTCGGCAAACAGTGGTCCGGCGCACGCGCCCTGGAGGCCTTGCTCACGAAGGCGGG

GGAGTTGAGAGGTCCGCCGTTACAGTTGGACACAGGCCAACTTCTCAAGATTGCAAAACGTGGCGGCGTGACCGCAGTGG

AGGCAGTGCATGCATGGCGCAATGCACTGACGGGTGCCCCCCTGAACCTGACCCCGGACCAAGTGGT

>M13R

ACCATGGCCTGACCCCGGACCAGGTGGTGGCCATCGCCAGCAATATTGGCGGCAAGCAGGCGCTGGAGACGGTGCAGCGG

CTGTTGCCGGTGCTGTGCCAGGACCATGGCCTGACCCCGGACCAGGTCGTGGCCATCGCCAGCAATGGCGGCGGCAAGCA

GGCGCTGGAGACGGTGCAGCGGCTGTTGCCGGTGCTGTGCCAGGACCATGGCCTGACCCCGGACCAGGTGGTGGCCATCG

CCAGCCACGATGGTGGCAAGCAGGCGCTGGAGAGCATTGTTGCCCAGTTATCTCGCCCTGATCCGGCGTTGGCCGCGTTG

ACCAACGACCACCTCGTCGCCTTGGCCTGCCTCGGCGGACGTCCTGCCCTGGATGCAGTGAAAAAGGGATTGCCGCACGC

GCCGGAATTGATCAGAAGAATCAATCGCCGTATTCCCGAACGCACGTCCCATCGCGTTGCCGACTACGCGCAAGTGGTTC

GCGTGCTGGAGTTTTTCCAGTGCCACTCCCACCCAGCGTACGCATTTGATGAGGCCATGACGCAGTTCGGGATGAGCAGG

AACGGGTTGGTACAGCTCTTTCGCAGAGTGGGCGTCACCGAACTCGAAGCCCGCGGTGGAACGCTCCCCCCAGCCTCGCA

GCGTTGGGACCGTATCCTCCAGGCATCAGGGATGAAAAGGGCCAAACCGTCCCCTACTTCAGCTCAAACACCGGATCAGG

CGTCTTTGCATGCATTCGCCGATTCGCTGGAGCGTGACCTTGATGCGCCCAGCCCAATGCACGAGGGAGATCAGACGCGG

GCAAGCAGCCGTAAACGGTCCCGATCGGATCGTGCTGTCACCGGCCCCTCCACACAGCAATCTTTCGAGGTGCGCGTTCC

CGAACAGCGCGATGCGCTGCATTTGCCCCTCAGCTGGAGGGTAAAACGCCCGCGTACCAGGATCGGGGGCGGCCTCCCGG

ATCC

**pTAL*_Bam_*_HI_-86**

> pCC2FOS-MscI-1

GGGCCTGGCACAGCACCGGCAACAGCCGCTGCACCGTCTCCAGCGCCTGCTTGCCGCCATCGTGCCTGGCGATGGCCACG

ACTTGGTCCGGGGTCAGGCCATGGGCCTGGCACAGCACCGGCAACAGCCGTTGCACCGTCTCCAGCGCCTGCTTGCCGCC

GCCATGGCTGGCGATGGCCACGACCTGGTCCGGGGTCAGGCCATGGTCCTGGCACAGCACCGGCAACAGCCGCTGCACCG

TCTCCAGCGCCTGCTTGCCGCCAATATTGCTGGCGATGGCCACCACCTGGTCCGGGGTCAGGCCATGGTCCTGGCACAGC

ACCGGCAACAGCCGCTGCACCGTCTCCAGCGCCTGCTTGCCGCCAATATTGCTGGCGATGGCCACCACCTGGTCCGGGGT

CAGGCCATGGTCCTGGCACAGCACCGGCAACAGCCGCTGCACCGTCTCCAGCGCCTGCTTGCCGCCGCCATGGCTGGCGA

TGGCCACGACCTGGTCCGGGGTCAGGCCATGGTCCTGGCACAGCACCGGCAACAGCCGCTGCACCGTCTCCAGCGCCTGC

TTGCCGCCAATATTGCTGGCGATGG

> pCC2FOS-MscI-2

CCATCGCCAGCCACGATGGCGGCAAGCAGGCGCTGGAGACGGTGCAGCGGCTGTTGCCGGTGCTGTGCCAGGACCATGGC

CTGACCCCGGACCAGGTGGTGGCCATCGCCAGCCATGGCGGCGGCAAGCAGGCGCTGGGGACGGTGCAACGGCTGTTGCC

GGTGCTGTGCCAGGACCATGGCCTGACCCCGGACCAAGTGGTGGCCATCGCCAACAATAACGGCGGCAAGCAGGCGCTGG

AGACGGTGCAGCGGCTGTTGCCGGTGCTGTGCCAGGACCATGGCCTGACCCCGGACCAAGTGGTGGCCATCGCCAACAAT

AACGGCGGCAAGCAGGCGCTGGAGACGGTGCAGCGGCTGTTGCCGGTGCTGTGCCAGGCCCATGGCCTGACCCCGGACCA

AGTGGTGGCCATCGCCAGCCACGATGGCGGCAAGCAGGCGCTGGAGACGGTGCAGCGGCTGTTGCCGGTGCTGTGCCAGG

ACCATGGCCTGACCCCGGCCCAGGTGGTGGCCATCGCCAGCAATAGCGGCGGCAAGCAGGCGCTGGAGACGGTGCAACGG

CTGTTGCCGGTGCTGTGCCAGGACCA

> pCC2FOS-MscI-3

CCATCGCCAACAATAACGGCGGCAAGCAGGCGCTGGAGACGGTGCAGCGGCTGTTGCCGGTACAGCGGCTGGTGCCGGTG

CTGTGCCAGGACCATGGCCTGACCCAGGACCAGGTGGTGGCCATCGCCAGCAATATTGGCGGCAAGCAGGCGCTGGAGAC

GGTGCAGCGGCTGTTGCCGGTGCTGTACCAGGACCATGGCCTGACCCAGGACCAGGTGGTGGCCATCGCCAGCCACGATG

GCGGCAAGCAGGCGCTGGAGACGGTGCAGCGGCTGTTGCCGGTGCTGTGCCAGGACCATGGCCTGACCCCGGACCAAGTG

GTGGCCATCGCCAGCCACGATGGCGGCAAACAGGCGCTGGAGACGGTGCAGCGGCTGTTGCCGGTGCTGTGCCAGGAACA

TGGCCTGACCCCGGACCAAGTGGTGGCCATCGCCAGCCACGATGGCGGCAAGCAGGCGCTGGAGACGGTGCAGCGGCTGT

TGCCGGTGCTGTGCCAGGACCATGGCCTGACCCCGGACCAGGTGGTGGCCATCGCCAGCCATGGCGGCGGCAAGCAGGCG

CTGGGGACGGTGCAACGGCTGTTGCCGGTGCTGTGCCAGGACCATGGCCTGACCCCGGACCAAGTGGTGGCCATCGCCAA

CAATAACGGCGGCAAGCAGGCGCTGGAGACGGTGCAGCGGCTGTTGCCGGTGCTGTGCCAGGACCATGGCCTGACCCCGG

ACCAAGTGGTGGCCATCGCCAACAATAACGGCGGCAAGCAGGCGCTGGAGACGGTGCAGCGGCTGTTGCCGGTGCTGTGC

CAGGC

> pCC2FOS-MscI-4

CCATCGCCAGCCATGGCGGCGGCAAGCAGGCGCTGGGGACGGTGCAACGGCTGTTGCCGGTGCTGTGCCAGGACCATGGC

CTGACCCCGGACCAAGTGGTGGCCATCGCCAACAATAACGGCGGCAAGCAGGCGCTGGAGACGGTGCAGCGGCTGTTGCC

GGTGCTGTGCCAGGACCATGGCCTGACCCCGGACCAAGTGGTGGCCATCGCCAACAATAACGGCGGCAAGCAGGCGCTGG

AGACGGTGCAGCGGCTGTTGCCGGTGCTGTGCCAGGCCCATGGCCTGACCCCGGACCAAGTGGTGGCCATCGCCAGCCAC

GATGGCGGCAAGCAGGCGCTGGAGACGGTGCAGCGGCTGTTGCCGGTGCTGTGCCAGGACCATGGCCTGACCCCGGCCCA

GGTGGTGGCCATCGCCAGCAATAGCGGCGGCAAGCAGGCGCTGGAGACGGTGCAACGGCTGTTGCCGGTGCTGTGCCAGG

ACCATGGCCTGACCCCGGCCCAGGTCGTGGCCATCGCCAACAATAACGGCGGCAAGCAGGCGCTGGAGACGGTGCAGCGG

CTGTTGCCGGTGCTGTGCCAGGACCATGGCCTGACCCCGGACCAGGTCGTGGCCATCGCCAGCCACGATGGCGGCAAGCA

GGCGCTGGAGACGGTGCAGCGGCTGTTGCCGGTGCTGTGCCAGGACCATGGCCTGACCCCGGACCAGGTCGTGGCCATCG

CCAGCAATGGCGGCGGCAAGCAGGCGCTGGCGACGGTGCAGCGGCTGTTGCCGGTGCTGTGCCAGGCCCATGGCCTGACC

CCGGACCAGGTCGTGGCCATCGCCAGCAATAGTGGCGGCAAGCAGGCGCTGGAGACGGTGCAGCGGCTGTTGCCGGTGCT

GTGCCAGGACCA

> pCC2FOS-MscI-5

CCATCGCCAGCAATATTGGCGGCAAGCAGGCGCTGGAGACGGTGCAGCGGCTGTTGCCGGTGCTGTACCAGGACCATGGC

CTGACCCAGGACCAGGTGGTGGCCATCGCCAGCCACGATGGCGGCAAGCAGGCGCTGGAGACGGTGCAGCGGCTGTTGCC

GGTGCTGTGCCAGGACCATGGCCTGACCCCGGACCAAGTGGTGGCCATCGCCAGCCACGATGGCGGCAAACAGGCGCTGG

AGACGGTGCAGCGGCTGTTGCCGGTGCTGTGCCAGGAACATGGCCTGACCCCGGACCAAGTGGTGGCCATCGCCAGCCAC

GATGGCGGCAAGCAGGCGCTGGAGACGGTGCAGCGGCTGTTGCCGGTGCTGTGCCAGGACCATGGCCTGACCCCGGACCA

GGTGGTGGCCATCGCCAGCCATGGCGGCGGCAAGCAGGCGCTGGGGACGGTGCAACGGCTGTTGCCGGTGCTGTGCCAGG

ACCATGGCCTGACCCCGGACCAAGTGGTGGCCATCGCCAACAATAACGGCGGCAAGCAGGCGCTGGAGACGGTGCAGCGG

CTGTTGCCGGTGCTGTGCCAGGACCATGGCCTGACCCCGGACCAAGTGGTGGCCATCGCCAACAATAACGGCGGCAAGCA

GGCGCTGGAGACGGTGCAGCGGCTGTTGCCGGTGCTGTGCCAGGCCCATGGCCTGACCCCGGACCAAGTGGTGGCCATCG

CCAGCCACGATGGCGGCAAGCAGGCGCTGGAGACGGTGCAGCGGCTGTTGCCGGTGCTGTGCCAGGACCATGGCCTGACC

CCGGCCCAGGTGGTGGCCATCGCCAGCAATAGCGGCGGCAAGCAGGCGCTGGAGACGGTGCAACGGCTGTTGCCGGTGCT

GTGCCAGGACCATG

> pCC2FOS-MscI-6

CCATCGCCAGCCACGATGGCGGCAAGCAGGCGCTGGAGACGGTGCAGCGGCTGTTGCCGGTGCTGTGCCAGGACCATGGC

CTGACCCCGGACCAGGTGGTGGCCATCGCCAGCCATGGCGGCGGCAAGCAGGCGCTGGGGACGGTGCAACGGCTGTTGCC

GGTGCTGTGCCAGGACCATGGCCTGACCCCGGACCAAGTGGTGGCCATCGCCAACAATAACGGCGGCAAGCAGGCGCTGG

AGACGGTGCAGCGGCTGTTGCCGGTGCTGTGCCAGGACCATGGCCTGACCCCGGACCAAGTGGTGGCCATCGCCAACAAT

AACGGCGGCAAGCAGGCGCTGGAGACGGTGCAGCGGCTGTTGCCGGTGCTGTGCCAGGCCCATGGCCTGACCCCGGACCA

AGTGGTGGCCATCGCCAGCCACGATGGCGGCAAGCAGGCGCTGGAGACGGTGCAGCGGCTGTTGCCGGTGCTGTGCCAGG

ACCATGGCCTGACCCCGGCCCAGGTGGTGGCCATCGCCAGCAATAGCGGCGGCAAGCAGGCGCTGGAGACGGTGCAACGG

CTGTTGCCGGTGCTGTGCCAGGACCATGGCCTGACCCCGGCCCAGGTCGTGGCCATCGCCAACAATAACGGCGGCAAGCA

GGCGCTGGAGACGGTGCAGCGGCTGTTGCCGGTGCTGTGCCAGGACCATGGCCTGACCCCGGACCAGGTCGTGGCCATCG

CCAGCCACGATGGCGGCAAGCAGGCGCTGGAGACGGTGCAGCGGCTGTTGCCGGTGCTGTGCCAGGA

> pCC2FOS-MscI-7

CCCGGACCAGGTCGTGGCCATCGCCAGCCATGGCGGCGGCAAGCAGGCGCTGGAGACGGTGCAACGGCTGTTGCCGGTGC

TGTGCCAGGCCCATGGCCTGACCCCGGACCAAGTCGTGGCCATCGCCAGGCACGATGGCGGCAAGCAGGCGCTGGAGACG

GTGCAGCGGCTGTTGCCGGTGCTGTGCCAGGCCCATGGCCTGACCCCAGACCAAGTGGTGGCCATCGCCAACAATAACGG

CGGCAAGCAGGCGCTGGAGACGGTGCAGCGGCTGTTGCCGGTGCTGTGCCAGGCCCATGGCCTGACCCCGGACCAAGTGG

TGGCCATCGCCAGCCACGATGGCGGCAAGCAGGCGCTGGAGACGGTGCAGCGGCTGTTGCCGGTGCTGTGCCAGGCCCAT

GGCCTGACCCCGGACCAGGTGGTGGCCATCGCCAGCCACGATGGCGGCAAGCAGGCGCTGGAGACGGTGCAGCGGCTGTT

GCCGGTGCTGTGCCAGGACCATGGCCTGACCCCGGACCAGGTGGTGGCCATCGCCAGCCACGATGGCGGCAAGCAGGCGT

TGGAGACGGTGCAGCGGCTGTTGCCGGTGCTGTGCCAGGACCATGGCCTGACCCCGGACCAGGTCGTGGCCATCGCCAGC

AATATTGGCGGCAAGCAGGCGCTGGAGACGGTGCAGCGGCTGTTGCCGGTGCTGTGCCAGGACCATGGCCTGACCCCGGA

CCAGGTGGTGGCCATCGCCAGCAATATTGGCGGCAAGCAGGCGCTGGAGACGGTACAGCGGCTGTTGCCGGTGCTGTGCC

> pCC2FOS-MscI-8

GCCATCGCCAGCAATATTGGCGGCAAGCAGGCGCTGGAGACGGTACAGCGGCTGTTGCCGGTGCTGTGCCAGGACCATGG

CCTGACCCCGGACCAGGTGGTGGCCATCGCCAACAATAACGGCGGCAAGCAGGCGCTGGAGACGGTGCAGCGGCTGTTGC

CGGTACAGCGGCTGGTGCCGGTGCTGTGCCAGGACCATGGCCTGACCCAGGACCAGGTGGTGGCCATCGCCAGCAATATT

GGCGGCAAGCAGGCGCTGGAGACGGTGCAGCGGCTGTTGCCGGTGCTGTACCAGGACCATGGCCTGACCCAGGACCAGGT

GGTGGCCATCGCCAGCCACGATGGCGGCAAGCAGGCGCTGGAGACGGTGCAGCGGCTGTTGCCGGTGCTGTGCCAGGACC

ATGGCCTGACCCCGGACCAAGTGGTGGCCATCGCCAGCCACGATGGCGGCAAACAGGCGCTGGAGACGGTGCAGCGGCTG

TTGCCGGTGCTGTGCCAGGAACATGGCCTGACCCCGGACCAAGTGGTGGCCATCGCCAGCCACGATGGCGGCAAGCAGGC

GCTGGAGACGGTGCAGCGGCTGTTGCCGGTGCTGTGCCAGGACCATGGCCTGACCCCGGACCAGGTGGTGGCCATCGCCA

GCCATGGCGGCGGCAAGCAGGCGCTGGGGACGGTGCAACGGCTGTTGCCGGTGCTGTGCCAG

> pCC2FOS-MscI-9

CCATCGCCAACAATAACGGCGGCAAGCAGGCGCTGGAGACGGTGCAGCGGCTGTTGCCGGTGCTGTGCCAGGACCATGGC

CTGACCCCGGACCAAGTGGTGGCCATCGCCAACAATAACGGCGGCAAGCAGGCGCTGGAGACGGTGCAGCGGCTGTTGCC

GGTGCTGTGCCAGGCCCATGGCCTGACCCCGGACCAAGTGGTGGCCATCGCCAGCCACGATGGCGGCAAGCAGGCGCTGG

AGACGGTGCAGCGGCTGTTGCCGGTGCTGTGCCAGGACCATGGCCTGACCCCGGCCCAGGTGGTGGCCATCGCCAGCAAT

AGCGGCGGCAAGCAGGCGCTGGAGACGGTGCAACGGCTGTTGCCGGTGCTGTGCCAGGACCATGGCCTGACCCCGGCCCA

GGTCGTGGCCATCGCCAACAATAACGGCGGCAAGCAGGCGCTGGAGACGGTGCAGCGGCTGTTGCCGGTGCTGTGCCAGG

ACCATGGCCTGACCCCGGACCAGGTCGTGGCCATCGCCAGCCACGATGGCGGCAAGCAGGCGCTGGAGACGGTGCAGCGG

CTGTTGCCGGTGCTGTGCCAGGACCATGGCCTGACCCCGGACCAGGTCGTGGCCATCGCCAGCAATGGCGGCGGCAAGCA

GGCGCTGGCGACGGTGCAGCGGCTGTTGCCGGTGCTGTGCCAGGCCCATGGCCTGACCCCGGACCAGGTCGTGGCCATCG

CCAGCAATAGTGGCGGCAAGCAGGCGCTGGAGACGGTGCAGCGGCTGTTGCCGGTGCTGTGCCAGGACCATG

> pCC2FOS-MscI-10

CCATCGCCAGCAATATTGGCGGCAAGCAGGCGCTGGAGACGGTGCAGCGGCTGTTGCCGGTGCTGTGCCAGGACCATGGC

CTGACCCCGGACCAGGTGGTGGCCATCGCCAGCAATATTGGCGGCAAGCAGGCGCTGGAGACGGTACAGCGGCTGTTGCC

GGTGCTGTGCCAGGACCATGGCCTGACCCCGGACCAGGTGGTGGCCATCGCCAACAATAACGGCGGCAAGCAGGCGCTGG

AGACGGTGCAGCGGCTGTTGCCGGTACAGCGGCTGGTGCCGGTGCTGTGCCAGGACCATGGCCTGACCCAGGACCAGGTG

GTGGCCATCGCCAGCAATATTGGCGGCAAGCAGGCGCTGGAGACGGTGCAGCGGCTGTTGCCGGTGCTGTACCAGGACCA

TGGCCTGACCCAGGACCAGGTGGTGGCCATCGCCAGCCACGATGGCGGCAAGCAGGCGCTGGAGACGGTGCAGCGGCTGT

TGCCGGTGCTGTGCCAGGACCATGGCCTGACCCCGGACCAAGTGGTGGCCATCGCCAGCCACGATGGCGGCAAACAGGCG

CTGGAGACGGTGCAGCGGCTGTTGCCGGTGCTGTGCCAGGAACATGGCCTGACCCCGGACCAAGTGGTGGCCATCGCCAG

CCACGATGGCGGCAAGCAGGCGCTGGAGACGGTGCAGCGGCTGTTGCCGGTGCTGTGCCAGGACCATGGCCTGACCCCGG

ACCAGGTGGTGGCCATCGCCAGCCATGGCGGCGGCAAGCAGGCGCTGGGGACGGTGCAACGGCTGTTGCCGGTGCTGTGC

C

> pCC2FOS-MscI-11

TGGTCCTGGCACAGCACCGGCAACAGCCGCTGCACCGTCTCCAGCGCCTGCTTGCCGCCAATATTGCTGGCGATGGCCAC

GACCTGGTCCGGGGTCAGGCCATGGTCCTGGCACAGCACCGGCAACAGCCGCTGCACCGTCTCCAACGCCTGCTTGCCGC

CATCGTGGCTGGCGATGGCCACCACCTGGTCCGGGGTCAGGCCATGGTCCTGGCACAGCACCGGCAACAGCCGCTGCACC

GTCTCCAGCGCCTGCTTGCCGCCATCGTGGCTGGCGATGGCCACCACCTGGTCCGGGGTCAGGCCATGGGCCTGGCACAG

CACCGGCAACAGCCGCTGCACCGTCTCCAGCGCCTGCTTGCCGCCATCGTGGCTGGCGATGGCCACCACTTGGTCCGGGG

TCAGGCCATGGGCCTGGCACAGCACCGGCAACAGCCGCTGCACCGTCTCCAGCGCCTGCTTGCCGCCGTTATTGTTGGCG

ATGGCCACCACTTGGTCTGGGGTCAGGCCATGGGCCTGGCACAGCACCGGCAACAGCCGCTGCACCGTCTCCAGCGCCTG

CTTGCCGCCATCGTGCCTGGCGATGGCCACGACTTGGTCCGGGGTCAGGCCATGGGCCTGGCACAGCACCGGCAACAGCC

GTTGCACCGTCTCCAGCGCCTGCTTGCCGCCGCCATGGCTGGCGATGGCCACGACCTGGTCCGGGGTCAGGCCATGGTCC

TGGCACAGCACCGGCAACAGCCGCTGCACCGTCTCCAGCGCCTGCTTGCCGCCAATATTGCTGGCGATGGCCACCACCTG

GTCCGGGGTCAGGCCATGGTCCTGGCACAGCACCGGCAACAGCCGCTGCACCGTCTCCAGCGCCTGCTTGCCGCCAATAT

TGCTGGCGATGGCCACCACCTGGTCCGGGGTCAGGCCATGGTCCTGGCACAGCACCGGCAACAGCCGCTGCACCGTCTCC

AGCGCCTGCTTGCCGCCGCC

> pCC2FOS-MscI-12

TGGTCCTGGCACAGCACCGGCAACAGCCGCTGCACCGTCTCCAGCGCCTGCTTGCCGCCGTTATTGTTGGCGATGGCCAC

CACTTGGTCCGGGGTCAGGCCATGGTCCTGGCACAGCACCGGCAACAGCCGTTGCACCGTCCCCAGCGCCTGCTTGCCGC

CGCCATGGCTGGCGATGGCCACCACCTGGTCCGGGGTCAGGCCATGGTCCTGGCACAGCACCGGCAACAGCCGCTGCACC

GTCTCCAGCGCCTGCTTGCCGCCATCGTGGCTGGCGATGGCCACCACTTGGTCCGGGGTCAGGCCATGTTCCTGGCACAG

CACCGGCAACAGCCGCTGCACCGTCTCCAGCGCCTGTTTGCCGCCATCGTGGCTGGCGATGGCCACCACTTGGTCCGGGG

TCAGGCCATGGTCCTGGCACAGCACCGGCAACAGCCGCTGCACCGTCTCCAGCGCCTGCTTGCCGCCATCGTGGCTGGCG

ATGGCCACCACCTGGTCCTGGGTCAGGCCATGGTCCTGGTACAGCACCGGCAACAGCCGCTGCACCGTCTCCAGCGCCTG

CTTGCCGCCAATATTGCTGGCGATGGCCACCACCTGGTCCTGGGTCAGGCCATGGTCCTGGCACAGCACCGGCACCAGCC

GCTGTACCGGCAACAGCCGCTGCACCGTCTCCAGCGCCTGCTTGCCGCCGTTATTGTTGGCGATGGCCACCACCTGGTCC

GGGGTCAGGCCATGGTCCTGGCACAGCACCGGCAACAGCCGCTGTACCGTCTCCAGCGCCTGCTTGCCGCCAATATTGCT

GGCGATGGCCACCACCTGGTCCGGGGTCAGGCCATGGTCCTGGCACAGCACCGGCAACAGCCGCTGCACCGTCTCCAGCG

CCTGCTTGCCGCCAATATTGCTGGCGATGG

> pCC2FOS-MscI-13

TGGTCCTGGCACAGCACCGGCAACAGCCGCTGCACCGTCTCCAGCGCCTGCTTGCCGCCATCGTGGCTGGCGATGGCCAC

CACCTGGTCCGGGGTCAGGCCATGGGCCTGGCACAGCACCGGCAACAGCCGCTGCACCGTCTCCAGCGCCTGCTTGCCGC

CATCGTGGCTGGCGATGGCCACCACTTGGTCCGGGGTCAGGCCATGGGCCTGGCACAGCACCGGCAACAGCCGCTGCACC

GTCTCCAGCGCCTGCTTGCCGCCGTTATTGTTGGCGATGGCCACCACTTGGTCTGGGGTCAGGCCATGGGCCTGGCACAG

CACCGGCAACAGCCGCTGCACCGTCTCCAGCGCCTGCTTGCCGCCATCGTGCCTGGCGATGGCCACGACTTGGTCCGGGG

TCAGGCCATGGGCCTGGCACAGCACCGGCAACAGCCGTTGCACCGTCTCCAGCGCCTGCTTGCCGCCGCCATGGCTGGCG

ATGGCCACGACCTGGTCCGGGGTCAGGCCATGGTCCTGGCACAGCACCGGCAACAGCCGCTGCACCGTCTCCAGCGCCTG

CTTGCCGCCAATATTGCTGGCGATGGCCACCACCTGGTCCGGGGTCAGGCCATGGTCCTGGCACAGCACCGGCAACAGCC

GCTGCACCGTCTCCAGCGCCTGCTTGCCGCCAATATTGCTGGCGATGGCCACCACCTGGTCCGGGGTCAGGCCATGGTCC

TGGCACAGCACCGGCAACAGCCGCTGCACCGTCTCCAGCGCCTGCTTGCCGCCGCCATGGCTGGCGATGGCCACGACCTG

GTCCGGGGTCAGGCCAT

> pCC2FOS-MscI-14

GGCGATGGCCACCACTTGGTCCGGGGTCAGGCCATGGTCCTGGCACAGCACCGGCAACAGCCGCTGCACCGTCTCCAGCG

CCTGCTTGCCGCCATCGTGGCTGGCGATGGCCACCACCTGGTCCTGGGTCAGGCCATGGTCCTGGTACAGCACCGGCAAC

AGCCGCTGCACCGTCTCCAGCGCCTGCTTGCCGCCAATATTGCTGGCGATGGCCACCACCTGGTCCTGGGTCAGGCCATG

GTCCTGGCACAGCACCGGCACCAGCCGCTGTACCGGCAACAGCCGCTGCACCGTCTCCAGCGCCTGCTTGCCGCCGTTAT

TGTTGGCGATGGCCACCACCTGGTCCGGGGTCAGGCCATGGTCCTGGCACAGCACCGGCAACAGCCGCTGTACCGTCTCC

AGCGCCTGCTTGCCGCCAATATTGCTGGCGATGGCCACCACCTGGTCCGGGGTCAGGCCATGGTCCTGGCACAGCACCGG

CAACAGCCGCTGCACCGTCTCCAGCGCCTGCTTGCCGCCAATATTGCTGGCGATGGCCACGACCTGGTCCGGGGTCAGGC

CATGGTCCTGGCACAGCACCGGCAACAGCCGCTGCACCGTCTCCAACGCCTGCTTGCCGCCATCGTGGCTGGCGATGGCC

ACCACCTGGTCCGGGGTCAGGCCATGGTCCTGGCACAGCACCGGCAACAGCCGCTGCACCGTCTCCAGCGCCTGCTTGCC

GCCATCGTGGCTGGCGATGGCCACCACCTGGTCCGGGGTCAGGCCATGGGCCTGGCACAGCACCGGCAACAGCCGCTGCA

CCGTCTCCAGCGCCTGCTTGCCGCCATCGTGGCTGGCGATGGCCACCACTTGGTCCGGGGTCAGGCCATGGGCCTGGCAC

AGCACCGGCAACAGCCGCTGCACCGTCT

> pCC2FOS-MscI-15

AACAGCCGCTGCACCGTCTCCAGCGCCTGCTTGCCGCCATCGTGGCTGGCGATGGCCACCACCTGGTCCGGGGTCAGGCC

ATGGGCCTGGCACAGCACCGGCAACAGCCGCTGCACCGTCTCCAGCGCCTGCTTGCCGCCATCGTGGCTGGCGATGGCCA

CCACTTGGTCCGGGGTCAGGCCATGGGCCTGGCACAGCACCGGCAACAGCCGCTGCACCGTCTCCAGCGCCTGCTTGCCG

CCGTTATTGTTGGCGATGGCCACCACTTGGTCTGGGGTCAGGCCATGGGCCTGGCACAGCACCGGCAACAGCCGCTGCAC

CGTCTCCAGCGCCTGCTTGCCGCCATCGTGCCTGGCGATGGCCACGACTTGGTCCGGGGTCAGGCCATGGGCCTGGCACA

GCACCGGCAACAGCCGTTGCACCGTCTCCAGCGCCTGCTTGCCGCCGCCATGGCTGGCGATGGCCACGACCTGGTCCGGG

GTCAGGCCATGGTCCTGGCACAGCACCGGCAACAGCCGCTGCACCGTCTCCAGCGCCTGCTTGCCGCCAATATTGCTGGC

GATGGCCACCACCTGGTCCGGGGTCAGGCCATGGTCCTGGCACAGCACCGGCAACAGCCGCTGCACCGTCTCCAGCGCCT

GCTTGCCGCCAATATTGCTGGCGATGGCCACCACCTGGTCCGGGGTCAGGCCATGGTCCTGGCACAGCACCGGCAACAGC

CGCTGCACCGTCTCCAGCGCCTGCTTGCCGCCGCCATGGCTGGCGATGGCCACGACCTGGTCCGGGGTCAGGCCATGGTC

CTGGCACAGCACCGGCAACAGCCGCTGCACCGTCTCCAGCGCCTGCTTGCCGCCAATATTGCTGGCGATGG

> pCC2FOS-MscI-16

CAGCACCGGCAACAGCCGCTGCACCGTCTCCAGCGCCTGCTTGCCGCCGTTATTGTTGGCGATGGCCACGACCTGGGCCG

GGGTCAGGCCATGGTCCTGGCACAGCACCGGCAACAGCCGTTGCACCGTCTCCAGCGCCTGCTTGCCGCCGCTATTGCTG

GCGATGGCCACCACCTGGGCCGGGGTCAGGCCATGGTCCTGGCACAGCACCGGCAACAGCCGCTGCACCGTCTCCAGCGC

CTGCTTGCCGCCATCGTGGCTGGCGATGGCCACCACTTGGTCCGGGGTCAGGCCATGGGCCTGGCACAGCACCGGCAACA

GCCGCTGCACCGTCTCCAGCGCCTGCTTGCCGCCGTTATTGTTGGCGATGGCCACCACTTGGTCCGGGGTCAGGCCATGG

TCCTGGCACAGCACCGGCAACAGCCGCTGCACCGTCTCCAGCGCCTGCTTGCCGCCGTTATTGTTGGCGATGGCCACCAC

TTGGTCCGGGGTCAGGCCATGGTCCTGGCACAGCACCGGCAACAGCCGTTGCACCGTCCCCAGCGCCTGCTTGCCGCCGC

CATGGCTGGCGATGGCCACCACCTGGTCCGGGGTCAGGCCATGGTCCTGGCACAGCACCGGCAACAGCCGCTGCACCGTC

TCCAGCGCCTGCTTGCCGCCATCGTGGCTGGCGATGGCCACCACTTGGTCCGGGGTCAGGCCATGTTCCTGGCACAGCAC

CGGCAACAGCCGCTGCACCGTCTCCAGCGCCTGTTTGCCGCCATCGTGGCTGGCGATGGCCACCACTTGGTCCGGGGTCA

GGCCATGGTCCTGGCACAGCACCGGCAACAGCCGCTGCACCGTCTCCAGCGCCTGCTTGCCGCCATCGTGGCTGGCGATG

G

> pCC2FOS-MscI-17

CCATTGCTGGCGATGGCCACGACCTGGTCCGGGGTCAGGCCATGGTCCTGGCACAGCACCGGCAACAGCCGCTGCACCGT

CTCCAGCGCCTGCTTGCCGCCATCGTGGCTGGCGATGGCCACGACCTGGTCCGGGGTCAGGCCATGGTCCTGGCACAGCA

CCGGCAACAGCCGCTGCACCGTCTCCAGCGCCTGCTTGCCGCCGTTATTGTTGGCGATGGCCACGACCTGGGCCGGGGTC

AGGCCATGGTCCTGGCACAGCACCGGCAACAGCCGTTGCACCGTCTCCAGCGCCTGCTTGCCGCCGCTATTGCTGGCGAT

GGCCACCACCTGGGCCGGGGTCAGGCCATGGTCCTGGCACAGCACCGGCAACAGCCGCTGCACCGTCTCCAGCGCCTGCT

TGCCGCCATCGTGGCTGGCGATGGCCACCACTTGGTCCGGGGTCAGGCCATGGGCCTGGCACAGCACCGGCAACAGCCGC

TGCACCGTCTCCAGCGCCTGCTTGCCGCCGTTATTGTTGGCGATGGCCACCACTTGGTCCGGGGTCAGGCCATGGTCCTG

GCACAGCACCGGCAACAGCCGCTGCACCGTCTCCAGCGCCTGCTTGCCGCCGTTATTGTTGGCGATGGCCACCACTTGGT

CCGGGGTCAGGCCATGGTCCTGGCACAGCACCGGCAACAGCCGTTGCACCGTCCCCAGCGCCTGCTTGCCGCCGCCATGG

CTGGCGATGGCCACCACCTGGTCCGGGGTCAGGCCATGGTCCTGGCACAGCACCGGCAACAGCCGCTGCACCGTCTCCAG

CGCCTGCTTGCCGCCATCGTGGCTGGCGATGG

>86P1F_1

CGGGGGAGTTGAGAGGTCCGCCGTTACAGTTGGACACAGGCCAACTTCTCAAGATTGCAAAACGTGGCGGCGTGACCGCA

GTGGAGGCAGTGCATGCATGGCGCAATGCACTGACGGGTGCCCCCCTGAACCTGACCCCGGACCAAGTGGTGGCCATCGC

CAGCAATATTGGCGGCAAGCAGGCGCTGGAGACGGTGCAGCGGCTGTTGCCGGTGCTGTGCCAGGACCATGGCCTGACCC

CGGACCAGGTCGTGGCCATCGCCAGCCATGGCGGCGGCAAGCAGGCGCTGGAGACGGTGCAGCGGCTGTTGCCGGTGCTG

TGCCAGGACCATGGCCTGACCCCGGACCAGGTGGTGGCCATCGCCAGCAATATTGGCGGCAAGCAGGCGCTGGAGACGGT

GCAGCGGCTGTTGCCGGTGCTGTGCCAGGACCATGGCCTGACCCCGGACCAGGTGGTGGCCATCGCCAGCAATATTGGCG

GCAAGCAGGCGCTGGAGACGGTGCAGCGGCTGTTGCCGGTGCTGTGCCAGGACCATGGCCTGACCCCGGACCAGGTCGTG

GCCATCGCCAGCCATGGCGGCGGCAAGCAGGCGCTGGAGACGGTGCAACGGCTGTTGCCGGTGCTGTGCCAGGCCCATGG

CCTGACCCCGGACCAAGTCGTGGCCATCGCCAGGCACGATGGCGGCAAGCAGGCGCTGGAGACGGTGCAGCGGCTGTTGC

CGGTGCTGTGCCAGGCCCATGGCCTGACCCCAGACCAAGTGGTGGCCATCGCCAACAATAACGGCGGCAAGCAGGCGCTG

GAGACGGTGCAGCGGCTGTTGCCGGTGCTGTGCCAGGCCCATGGCCTGACCCCGGACCAAGTGGTGGCCATCGCCAGCCA

CGATGGCGGCAAGCAGGCGCTGGAGACGGTGCAGCGGCTGTTGCCGGTGCTGTGCCAGGCCCATGGCCTGACCCCGGACC

AGGTGGTGGCCATCGCCAGC

>86P1F_2

CGCCGTTACAGTTGGACACAGGCCAACTTCTCAAGATTGCAAAACGTGGCGGCGTGACCGCAGTGGAGGCAGTGCATGCA

TGGCGCAATGCACTGACGGGTGCCCCCCTGAACCTGACCCCGGACCAAGTGGTGGCCATCGCCAGCAATATTGGCGGCAA

GCAGGCGCTGGAGACGGTGCAGCGGCTGTTGCCGGTGCTGTGCCAGGACCATGGCCTGACCCCGGACCAGGTCGTGGCCA

TCGCCAGCCATGGCGGCGGCAAGCAGGCGCTGGAGACGGTGCAGCGGCTGTTGCCGGTGCTGTGCCAGGACCATGGCCTG

ACCCCGGACCAGGTGGTGGCCATCGCCAGCAATATTGGCGGCAAGCAGGCGCTGGAGACGGTGCAGCGGCTGTTGCCGGT

GCTGTGCCAGGACCATGGCCTGACCCCGGACCAGGTGGTGGCCATCGCCAGCAATATTGGCGGCAAGCAGGCGCTGGAGA

CGGTGCAGCGGCTGTTGCCGGTGCTGTGCCAGGACCATGGCCTGACCCCGGACCAGGTCGTGGCCATCGCCAGCCATGGC

GGCGGCAAGCAGGCGCTGGAGACGGTGCAACGGCTGTTGCCGGTGCTGTGCCAGGCCCATGGCCTGACCCCGGACCAAGT

CGTGGCCATCGCCAGGCACGATGGCGGCAAGCAGGCGCTGGAGACGGTGCAGCGGCTGTTGCCGGTGCTGTGCCAGGCCC

ATGGCCTGACCCCAGACCAAGTGGTGGCCATCGCCAACAATAACGGCGGCAAGCAGGCGCTGGAGACGGTGCAGCGGCTG

TTGCCGGTGCTGTGCCAGGCCCATGGCCTGACCCCGGACCAAGTGGTGGCCATCGCCAGCCACGATGGCGGCAA

>86P1R_1

TGGGCAACAATGCTCTCCAGCGCCTGCTTGCCGCCATTGCTGGCGATGGCCACCACCTGGTTCGGGGTCAGGCCATGGTC

CTGGCACAGCACCGGCAACAGCCGCTGCACCGTCTCCAGCGCCTGCTTGCCGCCACTATTGCTGGCGATGGCCACGACCT

GGTCCGGGGTCAGGCCATGGGCCTGGCACAGCACCGGCAACAGCCGCTGCACCGTCGCCAGCGCCTGCTTGCCGCCGCCA

TTGCTGGCGATGGCCACGACCTGGTCCGGGGTCAGGCCATGGTCCTGGCACAGCACCGGCAACAGCCGCTGCACCGTCTC

CAGCGCCTGCTTGCCGCCATCGTGGCTGGCGATGGCCACGACCTGGTCCGGGGTCAGGCCATGGTCCTGGCACAGCACCG

GCAACAGCCGCTGCACCGTCTCCAGCGCCTGCTTGCCGCCGTTATTGTTGGCGATGGCCACGACCTGGGCCGGGGTCAGG

CCATGGTCCTGGCACAGCACCGGCAACAGCCGTTGCACCGTCTCCAGCGCCTGCTTGCCGCCGCTATTGCTGGCGATGGC

CACCACCTGGGCCGGGGTCAGGCCATGGTCCTGGCACAGCACCGGCAACAGCCGCTGCACCGTCTCCAGCGCCTGCTTGC

CGCCATCGTGGCTGGCGATGGCCACCACTTGGTCCGGGGTCAGGCCATGGGCCTGGCACAGCACCGGCAACAGCCGCTGC

ACCGTCTCCAGCGCCTGCTTGCCGCCGTTATTGTTGGCGATGGCCACCACTTGGTCCGGGGTCAGGCCATGGTCCTGGCA

CAGCACCGGCAACAGCCGCTGCACCGTCTCCAGCGCCTGCTTGCCGCCGTTATTGTTGGCGATGGCCACCACTTGGTCCG

GGGTCAGGCCATGGTCCTGGCACAGCACCGGCAACAGCC

>86P1R_2

GACCCCGGACCAAGTGGTGGCCATCGCCAACAATAACGGCGGCAAGCAGGCGCTGGAGACGGTGCAGCGGCTGTTGCCGG

TGCTGTGCCAGGACCATGGCCTGACCCCGGACCAAGTGGTGGCCATCGCCAACAATAACGGCGGCAAGCAGGCGCTGGAG

ACGGTGCAGCGGCTGTTGCCGGTGCTGTGCCAGGCCCATGGCCTGACCCCGGACCAAGTGGTGGCCATCGCCAGCCACGA

TGGCGGCAAGCAGGCGCTGGAGACGGTGCAGCGGCTGTTGCCGGTGCTGTGCCAGGACCATGGCCTGACCCCGGCCCAGG

TGGTGGCCATCGCCAGCAATAGCGGCGGCAAGCAGGCGCTGGAGACGGTGCAACGGCTGTTGCCGGTGCTGTGCCAGGAC

CATGGCCTGACCCCGGCCCAGGTCGTGGCCATCGCCAACAATAACGGCGGCAAGCAGGCGCTGGAGACGGTGCAGCGGCT

GTTGCCGGTGCTGTGCCAGGACCATGGCCTGACCCCGGACCAGGTCGTGGCCATCGCCAGCCACGATGGCGGCAAGCAGG

CGCTGGAGACGGTGCAGCGGCTGTTGCCGGTGCTGTGCCAGGACCATGGCCTGACCCCGGACCAGGTCGTGGCCATCGCC

AGCAATGGCGGCGGCAAGCAGGCGCTGGCGACGGTGCAGCGGCTGTTGCCGGTGCTGTGCCAGGCCCATGGCCTGACCCC

GGACCAGGTCGTGGCCATCGCCAGCAATAGTGGCGGCAAGCAGGCGCTGGAGACGGTGCAGCGGCTGTTGCCGGTGCTGT

GCCAGGACCATGGCCTGACCCCGAACCAGGTGGTGGCCATCGCCAGCAATGGCGGCAAGCAGGCGCTGGAGAGCA

>86P2F

CGCGCACATCGTTGCGCTCAGCCAACACCCGGCAGCGTTAGGGACCGTCGCTGTCAAGTATCAGCACATAATCACGGCGT

TGCCAGAGGCGACACACGAAGACATCGTTGGCGTCGGCAAACAGTGGTCCGGCGCACGCGCCCTGGAGGCCTTGCTCACG

GAGGCGGGGGAGTTGAGAGGTCCGCCGTTACAGTTGGACACAGGCCAACTTCTCAAGATTGCAAAACGTGGCGGCGTGAC

CGCAGTGGAGGCAGTGCATGCATGGCGCAATGCACTGACGGGTGCCCCCCTGAACCTGACCCCGGACCAAGTGGTGGCCA

TCGCCAGCAATATTGGCGGCAAGCAGGCGCTGGAGACGGTGCAGCGGCTGTTGCCGGTGCTGTGCCAGGACCATGGCCTG

ACCCCGGACCAGGTCGTGGCCATCGCCAGCCATGGCGGCGGCAAGCAGGCGCTGGAGACGGTGCAGCGGCTGTTGCCGGT

GCTGTGCCAGGACCATGGCCTGACCCCGGACCAGGTGGTGGCCATCGCCAGCAATATTGGCGGCAAGCAGGCGCTGGAGA

CGGTGCAGCGGCTGTTGCCGGTGCTGTGCCAGGACCATGGCCTGACCCCGGACCAGGTGGTGGCCATCGCCAGCAATATT

GGCGGCAAGCAGGCGCTGGAGACGGTGCAGCGGCTGTTGCCGGTGCTGTGCCAGGACCATGGCCTGACCCCGGACCAGGT

CGTGGCCATCGCCAGCCATGGCGGCGGCAAGCAGGCGCTGGAGACGGTGCAACGGCTGTTGCCGGTGCTGTGCCAGGCCC

ATGGCCTGACCCCGGACCAAGTCGTGGCCATCGCCAGGCACGATGGCGGCAAGCAGGCGCTGGAGACGGTGCAGCGGCTG

TTGCCGGTGCTGTGCCAGGCCCATGGCCTGACCCCAGACCAAGTGGTGGCCATCGCCAACAATAACGGCGGCAAGCA

>86P2R

CGCCGTGATTATGTGCTGATACTTGACAGCGACGGTCCCTAACGCTGCCGGGTGTTGGCTGAGCGCAACGATGTGCGCGT

GTGTAAACCCATGGCCCACCAGTGCCTCGTGGTGCTGCGCCACTGTCGAACGCACCTTCGGTTTGATCTTCTCTTGCTGC

TGCTGACTGTAGCCGAGCGTGCGTAGATCCACCTGCGCGGCCGGCGAAGCGTCGGAGGGTTGCGCCGCACGCCGTCGCGG

GGCCGGCTTGGCGCGCGGCGGCCGCGCGGCAGTGACAGCGACACGCACGGTGGGTGGCGGGTCATCGGCTGCACGCAGAC

CCGATTGCACCTCATCCCACTCTGCTGGGGCAGCCGCTGTATGCGGTGTGCCGACGGCAGGCATCGAATCAAGAAGCGAT

GTATCAAGAAGCGACGGATCGAACTGACGGAGCAGATCGCTGAAGCTGCCCGCCGAGAACGCAGGCGAGGGCGCAGGGGG

AGATGGCAGCCGGGTCCGGGACATCGTCCGCCGAGCGGGCAAGCCATCCAGGGGGCCGCCAGCAGGCGGAGCCCCCCCCC

GATCTGCAGTCGGCTGAACCCTATCCGGTTGGGGTCCGGGCAGAAGCTCGCGGGCAGGACTTGGCGTGCGCGAACGAATG

GGATCC

>86P3F

GGGATTGCCGCACGCGCCGGAATTGATCAGAAGAATCAATCGCCGTATTCCCGAACGCACGTCCCATCGCGTTCCCGACC

TCGCGCACGTGGTGCGCGTGCTTGGTTTTTTCCAGAGCCACTCCCACCCAGCGCAAGCATTCGATGACGCCATGACGCAG

TTCGGGATGAGCAGGCACGGCTTGGTACAGCTCTTTCGCAGAGTGGGCGTCACCGAATTCGAAGCCCGCTACGGAACGCT

CCCCCCAGCCTCGCAGCGTTGGGACCGTATCCTCCAGGCATCAGGGATGAAAAGGGCCAAACCGTCCCCTACTTCAGCTC

AAACACCGGATCAGGCGTCTTTGCATGCATTCGCCGATTCGCTGGAGCGTGACCTTGATGCGCCCAGCCCAATGCACGAG

GGAGATCAGACGCGGGCAAGCAGCCGTAAACGGTCCCGATCGGATCGTGCTGTCACCGACCCCTCCACACAGCAATCTTT

CGAGGTGCGCGTTCCCGAACAGCGCGATGCGCTGCATTTGCCCCTCAGCTGGAGGGTAAAACGCCCGCGTACCAGGATCG

GGGGCGGCCTCCCGGATCC

>86P3R

ATTCGGTGACGCCCACTCTGCGAAAGAGCTGTACCAAGCCGTGCCTGCTCATCCCGAACTGCGTCATGGCGTCATCGAAT

GCTTGCGCTGGGTGGGAGTGGCTCTGGAAAAAACCAAGCACGCGCACCACGTGCGCGAGGTCGGGAACGCGATGGGACGT

GCGTTCGGGAATACGGCGATTGATTCTTCTGATCAATTCCGGCGCGTGCGGCAATCCCTTTTTCACTGCATCCAGGGCAG

GACGTCCGCCGAGGCAGGCCAAGGCGACGAGGTGGTCGTTGGTCAACGCGGCCAACGCCGGATCAGGGCGAGATAACTGG

GCAACAATGCTCTCCAGCGCCTGCTTGCCGCCATTGCTGGCGATGGCCACCACCTGGTTCGGGGTCAGGCCATGGTCCTG

GCACAGCACCGGCAACAGCCGCTGCACCGTCTCCAGCGCCTGCTTGCCGCCACTATTGCTGGCGATGGCCACGACCTGGT

CCGGGGTCAGGCCATGGGCCTGGCACAGCACCGGCAACAGCCGCTGCACCGTCGCCAGCGCCTGCTTGCCGCCGCCATTG

CTGGCGATGGCCACGACCTGGTCCGGGGTCAGGCCATGGTCCTGGCACAGCACCGGCAACAGCCGCTGCACCGTCTCCAG

CGCCTGCTTGCCGCCATCGTGGCTGGCGATGGCCACGACCTGGTCCGGGGTCAGGCCATGGTCCTGGCACAGCACCGGCA

ACAGCCGCTGCACCGTCTCCAGCGCCTGCTTGCCGCCGTTATTGTTGGCGATGGCCACGACCTGGGCCGGGGTCAGGCCA

TGGTCCTGGCACAGCACCGGCAACAGCCGTTGCACCGTCTCCAGCGCCTGCTTGCCGCCGCTATTGCTGGCGATGGCCAC

CACCTGGGCCGGGGTCAGGCCATGGTCCTGGCACAGCACCGGCAACAGCCGCTGCACCGTCTCCAGCGCCTGCTTGCCGC

CATCGTGGCTGGC

>M13F

GGATCCCATTCGTTCGCGCACGCCAAGTCCTGCCCGCGAGCTTCTGCCCGGACCCCAACCGGATAGGGTTCAGCCGACTG

CAGATCGGGGGGGGGCTCCGCCTGCTGGCGGCCCCCTGGATGGCTTGCCCGCTCGGCGGACGATGTCCCGGACCCGGCTG

CCATCTCCCCCTGCGCCCTCGCCTGCGTTCTCGGCGGGCAGCTTCAGCGATCTGCTCCGTCAGTTCGATCCGTCGCTTCT

TGATACATCGCTTCTTGATTCGATGCCTGCCGTCGGCACACCGCATACAGCGGCTGCCCCAGCAGAGTGGGATGAGGTGC

AATCGGGTCTGCGTGCAGCCGATGACCCGCCACCCACCGTGCGTGTCGCTGTCACTGCCGCGCGGCCGCCGCGCGCCAAG

CCGGCCCCGCGACGGCGTGCGGCGCAACCCTCCGACGCTTCGCCGGCCGCGCAGGTGGATCTACGCACGCTCGGCTACAG

TCAGCAGCAGCAAGAGAAGATCAAACCGAAGGTGCGTTCGACAGTGGCGCAGCACCACGAGGCACTGGTGGGCCATGGGT

TTACACACGCGCACATCGTTGCGCTCAGCCAACACCCGGCAGCGTTAGGGACCGTCGCTGTCAAGTATCAGCACATAATC

ACGGCGTTGCCAGAGGCGACACACGAAGACATCGTTGGCGTCGGCAAACAGTGGTCCGGCGCACGCGCCCTGGAGGCCTT

GCTCACGGAGGCGGGGGAGTTGAGAGGTCCGCCGTTACAGTTGGACACAGGCCAACTTCTCAAGATTGCAAAACGTGGCG

GCGTGACCGCAGTGGAGGCAGTGCATGCATGGCGCAATGCACTGACGGGTGCCCCCCTGAACCTGACCCCGGACCAAGTG

GTGGCCATCGCCAGCAATATTGGCGGCAAGCAGGCGCTGGAGACGGTGCAGCGGCTGTTGCCGGTGCTGTGCCAGGACCA

TGG

>M13R (reversed)

ACCCCGGACCAGGTCGTGGCCATCGCCAGCAATGGCGGCGGCAAGCAGGCGCTGGCGACGGTGCAGCGGCTGTTGCCGGT

GCTGTGCCAGGCCCATGGCCTGACCCCGGACCAGGTCGTGGCCATCGCCAGCAATAGTGGCGGCAAGCAGGCGCTGGAGA

CGGTGCAGCGGCTGTTGCCGGTGCTGTGCCAGGACCATGGCCTGACCCCGAACCAGGTGGTGGCCATCGCCAGCAATGGC

GGCAAGCAGGCGCTGGAGAGCATTGTTGCCCAGTTATCTCGCCCTGATCCGGCGTTGGCCGCGTTGACCAACGACCACCT

CGTCGCCTTGGCCTGCCTCGGCGGACGTCCTGCCCTGGATGCAGTGAAAAAGGGATTGCCGCACGCGCCGGAATTGATCA

GAAGAATCAATCGCCGTATTCCCGAACGCACGTCCCATCGCGTTCCCGACCTCGCGCACGTGGTGCGCGTGCTTGGTTTT

TTCCAGAGCCACTCCCACCCAGCGCAAGCATTCGATGACGCCATGACGCAGTTCGGGATGAGCAGGCACGGCTTGGTACA

GCTCTTTCGCAGAGTGGGCGTCACCGAATTCGAAGCCCGCTACGGAACGCTCCCCCCAGCCTCGCAGCGTTGGGACCGTA

TCCTCCAGGCATCAGGGATGAAAAGGGCCAAACCGTCCCCTACTTCAGCTCAAACACCGGATCAGGCGTCTTTGCATGCA

TTCGCCGATTCGCTGGAGCGTGACCTTGATGCGCCCAGCCCAATGCACGAGGGAGATCAGACGCGGGCAAGCAGCCGTAA

ACGGTCCCGATCGGATCGTGCTGTCACCGACCCCTCCACACAGCAATCTTTCGAGGTGCGCGTTCCCGAACAGCGCGATG

CGCTGCATTTGCCCCTCAGCTGGAGGGTAAAACGCCCGCGTACCAGGATCGGGGGCGGCCTCCCGGATCC

**pTAL*_Bam_*_HI_-*talC***

>pCC2FOS-MscI-talC_1

CAATATTGGCGGCAAGCAGGCGCTGGAGACGGTGCAGCGGCTGTTGCCGGTGCTGTGCCAGGCCCATGGCCTGACCCCGG

AGCAGGTGGTGGCCATCGCCAGCAATGGCGGCGGCAAGCAGGCGCTGGAGACGGTGCAGCGGTTGTTGCCGATGCTGTGC

CAGGCCCATGGCCTGACCCCGGAGCAGGTGGTGGCCATCGCCAGCAATAACGGCGGCAAGCAGGCGCTGGAGACGGTGCA

GCGGCTGTTGCCGGTGCTGTGCCAGGCCCATGGCCTGACCCCGGAGCAGGTGGTGGCCATCGCCAGCAATGGCGGCGGCA

AGCAGGCGCTGGAGACGGTGCAGCGGCTGTTGCCGGTGCTGTGCCAGGCCCATGGCCTGACCCCGGACCAAGTGGTGGCC

ATCGCCAGCCACGATGGCGGCAAGCAGGCGCTGGAGACGGTGCAGCGGCTGTTGCCGGTGCTGTGCCAGGCCCATGGCCT

GACCCCGGCGCAGGTGGTGGCCATCGCCAGCAATATTGGCGGCAAGCAGGCGCTGGAGACGGTGCGGCGGCTGTTGCCGG

TGCTGTGCCAGGCCCATGGCCTGACCCCGGCGCAGGTGGTGGCCATCGCCAACAATAACGGCGGCAAGCAGGCGCTGGAG

ACGGTGCAGCGGCTGTTGCCGGTGCTGTGCCAGGCCCATGGCCTGACCCCGGAGCAGGTGGTGGCCATCGCCAGCAATGG

CGGCAAGCAGGCGCTGGAGACGGTGCAGCGGCTGTTGCCGGTGCTGTGCCAGGCCCATGGCCTGACCCCGGAGCAGGTGG

TGGCCATCGCCAGCAATATTGGCGGCAAGCAGGCGCTGGAGACGGTGCAGCGGCTGTTGCCGGTGCTGTGCCAGGCCCAT

GGCCTGACCCCGGAGCAGGTGGTGGCCATCGCCAGCAATAACGGCGGCAAGCAGGCCCTGGAGACGGTGCAGCGGCTGTT

GCCGGTGCTGTGCCAGGCCC

>pCC2FOS-MscI-talC_2

CCATCGCCAGCAATAACGGCGGCAAGCAGGCGCTGGAGACGGTGCAGCGGCTGTTGCCGGTGCTGTGCCAGGCCCATGGC

CTGACCCCGGAGCAGGTGGTGGCCATCGCCAGCAATGGCGGCGGCAAGCAGGCGCTGGAGACGGTGCAGCGGCTGTTGCC

GGTGCTGTGCCAGGCCCATGGCCTGACCCCGGACCAAGTGGTGGCCATCGCCAGCCACGATGGCGGCAAGCAGGCGCTGG

AGACGGTGCAGCGGCTGTTGCCGGTGCTGTGCCAGGCCCATGGCCTGACCCCGGCGCAGGTGGTGGCCATCGCCAGCAAT

ATTGGCGGCAAGCAGGCGCTGGAGACGGTGCGGCGGCTGTTGCCGGTGCTGTGCCAGGCCCATGGCCTGACCCCGGCGCA

GGTGGTGGCCATCGCCAACAATAACGGCGGCAAGCAGGCGCTGGAGACGGTGCAGCGGCTGTTGCCGGTGCTGTGCCAGG

CCCATGGCCTGACCCCGGAGCAGGTGGTGGCCATCGCCAGCAATGGCGGCAAGCAGGCGCTGGAGACGGTGCAGCGGCTG

TTGCCGGTGCTGTGCCAGGCCCATGGCCTGACCCCGGAGCAGGTGGTGGCCATCGCCAGCAATATTGGCGGCAAGCAGGC

GCTGGAGACGGTGCAGCGGCTGTTGCCGGTGCTGTGCCAGGCCCATGGCCTGACCCCGGAGCAGGTGGTGGCCATCGCCA

GCAATAACGGCGGCAAGCAGGCCCTGGAGACGGTGCAGCGGCTGTTGCCGGTGCTGTGCCAGGCCC

>pCC2FOS-MscI-talC_3

CCATCGCCAACAATAACGGCGGCAAGCAGGCGCTGGAGACGGTGCAGCGGCTGTTGCCGGTGCTGTGCCAGGCCCATGGC

CTGACCCCGGAGCAGGTGGTGGCCATCGCCAGCAATGGCGGCAAGCAGGCGCTGGAGACGGTGCAGCGGCTGTTGCCGGT

GCTGTGCCAGGCCCATGGCCTGACCCCGGAGCAGGTGGTGGCCATCGCCAGCAATATTGGCGGCAAGCAGGCGCTGGAGA

CGGTGCAGCGGCTGTTGCCGGTGCTGTGCCAGGCCCATGGCCTGACCCCGGAGCAGGTGGTGGCCATCGCCAGCAATAAC

GGCGGCAAGCAGGCCCTGGAGACGGTGCAGCGGCTGTTGCCGGTGCTGTGCCAGGCCCATGGCCTGACCCCGGACCAGGT

GGTGGCCATCGCCAGCCACGATGGCGGCAAGCAGGCGCTGGAGACGGTGCAGCGGCTGTTGCCGGTGCTGTGCCAGGCCC

ATGGCCTGACCCTGGAGCAGGTGGTGGCCATCGCCAGCAATGGCGGCGGCAAGCAGGCGCTGGAGACGGTGCAGCGGCTG

TTGCCGGTGCTGTGCCAGGCCCATGGCCTGACCCCGGCGCAGGTGGTGGCCATCGCCTGCAATATTGGCGGCAAGCAGGC

GCTGGAGACGGTGCGGCGGCTGTTGCCGGTGCTGTGCCAGGCCCATGGCCTGACCCCGGCGCAGGTGGTGGCCATCGCCA

ACAATAACGGCGGCAAGCAGGCGCTGGAGACGGTGCAGCGGCTGTTGCCGGTGCTGTGCCAGGCCCATGGCCTGACCCCG

GCGCAGGTGGTGGCCATCGCCAGCAATGGCGGCAAGCAGGCGCTGGAGACGGTGCAGCGGCTGTTGCCGGTGCTGTGCCA

GGCCC

>pCC2FOS-MscI-talC_4

CCATCGCCAGCAATAGCGGCGGCAAGCAGGCGCTGGAGACGGTGCAGCGGCTGTTGCCGGTGCTGTGCCAGGCCCATGGC

CTGACCCCGGAGCAGGTGGTGGCCATCGCCAGCAATGGCGGCGGCAAGCAGGCGCTGGAGACGGTGCAGCGGCTGTTGCC

GGTGCTGTGCCAGGCCCATGGCCTGACCCCGGCGCAGGTGGTGGCCATCGCCAGCAATAGCGGCGGCAAGCAGGCGCTGG

AGACGGTGCAGCGGCTGTTGCCGGTGCTGTGCCAGGCCCATGGCCTGACCCCGGACCAGGTGGTGGCCATCGCCAGCCAC

GATGGCGGCAAGCAGGCGCTGGAGACGGTGCAGCGACTGTTGCCGGTGCTGTGCCAGGCCCATGGCCTGACCCCGGAGCA

GGTGGTGGCCATCGCCAGCAATATTGGCGGCAAGCAGGCGCTGGAGACGGTGCAGCGGCTGTTGCCGGTGCTGTGCCAGG

CCCATGGCCTGACCCCGGAGCAGGTGGTGGCCATCGCCAGCAATGGCGGCGGCAAGCAGGCGCTGGAGACGGTGCAGCGG

TTGTTGCCGATGCTGTGCCAGGCCCATGGCCTGACCCCGGAGCAGGTGGTGGCCATCGCCAGCAATAACGGCGGCAAGCA

GGCGCTGGAGACGGTGCAGCGGCTGTTGCCGGTGCTGTGCCAGGCCCATGGCCTGACCCCGGAGCAGGTGGTGGCCATCG

CCAGCAATGGCGGCGGCAAGCAGGCGCTGGAGACGGTGCAGCGGCTGTTGCCGGTGCTGTGCCAGGCCCATGGCCTGACC

CCGGACCAAGTGGTGGCCATCGCCAGCCACGATGGCGGCAAGCAGGCGCTGGAGACGGTGCAGCGGCTGTTGCCGGTGCT

GTGCCAGGCCC

>pCC2FOS-MscI-talC_5

CCATCGCCAGCAATGGCGGCAAGCAGGCGCTGGAGACGGTGCAGCGGCTGTTGCCGGTGCTGTGCCAGGCCCATGGCCTG

ACCCCGGAGCAGGTGGTGGCCATCGCCAGCAATATTGGCGGCAAGCAGGCGCTGGAGACGGTGCAGCGGCTGTTGCCGGT

GCTGTGCCAGGCCCATGGCCTGACCCCGGAGCAGGTGGTGGCCATCGCCAGCAATAACGGCGGCAAGCAGGCCCTGGAGA

CGGTGCAGCGGCTGTTGCCGGTGCTGTGCCAGGCCCATGGCCTGACCCCGGACCAGGTGGTGGCCATCGCCAGCCACGAT

GGCGGCAAGCAGGCGCTGGAGACGGTGCAGCGGCTGTTGCCGGTGCTGTGCCAGGCCCATGGCCTGACCCTGGAGCAGGT

GGTGGCCATCGCCAGCAATGGCGGCGGCAAGCAGGCGCTGGAGACGGTGCAGCGGCTGTTGCCGGTGCTGTGCCAGGCCC

ATGGCCTGACCCCGGCGCAGGTGGTGGCCATCGCCTGCAATATTGGCGGCAAGCAGGCGCTGGAGACGGTGCGGCGGCTG

TTGCCGGTGCTGTGCCAGGCCCATGGCCTGACCCCGGCGCAGGTGGTGGCCATCGCCAACAATAACGGCGGCAAGCAGGC

GCTGGAGACGGTGCAGCGGCTGTTGCCGGTGCTGTGCCAGGCCCATGGCCTGACCCCGGCGCAGGTGGTGGCCATCGCCA

GCAATGGCGGCAAGCAGGCGCTGGAGACGGTGCAGCGGCTGTTGCCGGTGCTGTGCCAGGCCCATGGTCTGACCCCGGCG

CAGGTGGTGGCCATCGCCAGCCACGATGGCGGCAAGCAGGCGTTGGAGACGGTGCAGCGGCTGTTGCCGGTGCTGTGCCA

GGCCC

>pCC2FOS-MscI-talC_6

CCATCGCCAGCAATAACGGCGGCAAGCAGGCCCTGGAGACGGTGCAGCGGCTGTTGCCGGTGCTGTGCCAGGCCCATGGC

CTGACCCCGGACCAGGTGGTGGCCATCGCCAGCCACGATGGCGGCAAGCAGGCGCTGGAGACGGTGCAGCGGCTGTTGCC

GGTGCTGTGCCAGGCCCATGGCCTGACCCTGGAGCAGGTGGTGGCCATCGCCAGCAATGGCGGCGGCAAGCAGGCGCTGG

AGACGGTGCAGCGGCTGTTGCCGGTGCTGTGCCAGGCCCATGGCCTGACCCCGGCGCAGGTGGTGGCCATCGCCTGCAAT

ATTGGCGGCAAGCAGGCGCTGGAGACGGTGCGGCGGCTGTTGCCGGTGCTGTGCCAGGCCCATGGCCTGACCCCGGCGCA

GGTGGTGGCCATCGCCAACAATAACGGCGGCAAGCAGGCGCTGGAGACGGTGCAGCGGCTGTTGCCGGTGCTGTGCCAGG

CCCATGGCCTGACCCCGGCGCAGGTGGTGGCCATCGCCAGCAATGGCGGCAAGCAGGCGCTGGAGACGGTGCAGCGGCTG

TTGCCGGTGCTGTGCCAGGCCCATGGTCTGACCCCGGCGCAGGTGGTGGCCATCGCCAGCCACGATGGCGGCAAGCAGGC

GTTGGAGACGGTGCAGCGGCTGTTGCCGGTGCTGTGCCAGGCCCATGGCCTGACCCCGGACCAGGTGGTGGCCATCGCCA

GCAATAACGGCGGCAAGCAGGCGCTGGAGACGGTGCAGCGGCTGTTGCCGGTGCTGTGCCAGGCC

>pCC2FOS-MscI-talC_7

CCATCGCCAGCAATAGCGGCGGCAAGCAGGCGCTGGAGACGGTGCAGCGGCTGTTGCCGGTGCTGTGCCAGGCCCATGGC

CTGACCCCGGACCAGGTGGTGGCCATCGCCAGCCACGATGGCGGCAAGCAGGCGCTGGAGACGGTGCAGCGACTGTTGCC

GGTGCTGTGCCAGGCCCATGGCCTGACCCCGGAGCAGGTGGTGGCCATCGCCAGCAATATTGGCGGCAAGCAGGCGCTGG

AGACGGTGCAGCGGCTGTTGCCGGTGCTGTGCCAGGCCCATGGCCTGACCCCGGAGCAGGTGGTGGCCATCGCCAGCAAT

GGCGGCGGCAAGCAGGCGCTGGAGACGGTGCAGCGGTTGTTGCCGATGCTGTGCCAGGCCCATGGCCTGACCCCGGAGCA

GGTGGTGGCCATCGCCAGCAATAACGGCGGCAAGCAGGCGCTGGAGACGGTGCAGCGGCTGTTGCCGGTGCTGTGCCAGG

CCCATGGCCTGACCCCGGAGCAGGTGGTGGCCATCGCCAGCAATGGCGGCGGCAAGCAGGCGCTGGAGACGGTGCAGCGG

CTGTTGCCGGTGCTGTGCCAGGCCCATGGCCTGACCCCGGACCAAGTGGTGGCCATCGCCAGCCACGATGGCGGCAAGCA

GGCGCTGGAGACGGTGCAGCGGCTGTTGCCGGTGCTGTGCCAGGCCCATGGCCTGACCCCGGCGCAGGTGGTGGCCATCG

CCAGCAATATTGGCGGCAAGCAGGCGCTGGAGACGGTGCGGCGGCTGTTGCCGGTGCTGTGCCAGGCCCATGGCCTGACC

CCGGCGCAGGTGGTGGCCATCGCCAACAATAACGGCGGCAAGCAGGCGCTGGAGACGGTGCAGCGGCTGTTGCCGGTGCT

GTGCCAGGCCC

>pCC2FOS-MscI-talC_8

CCATCGCCAGCAATAGCGGCGGCAAGCAGGCGCTGGAGACGGTGCAGCGGCTGTTGCCGGTGCTGTGCCAGGCCCATGGC

CTGACCCCGGACCAGGTGGTGGCCATCGCCAGCCACGATGGCGGCAAGCAGGCGCTGGAGACGGTGCAGCGACTGTTGCC

GGTGCTGTGCCAGGCCCATGGCCTGACCCCGGAGCAGGTGGTGGCCATCGCCAGCAATGGCGGCGGCAAGCAGGCGCTGG

AGACGGTGCAGCGGCTGTTGCCGGTGCTGTGCCAGGCCCATGGCCTGACCCCGGACCAAGTGGTGGCCATCGCCAGCCAC

GATGGCGGCAAGCAGGCGCTGGAGACGGTGCAGCGGCTGTTGCCGGTGCTGTGCCAGGCCCATGGCCTGACCCCGGAGCA

GGTGGTGGCCATCGCCAGCAATAACGGCGGCAAGCAGGCGCTGGAGACGGTGCAGCGGCTGTTGCCGGTGCTGTGCCAGG

CCCATGGCCTGACCCCGGAGCAGGTGGTGGCCATCGCCAGCAATGGCGGCGGCAAGCAGGCGCTGGAGACGGTGCAGCGG

CTGTTGCCGGTGCTGTGCCAGGCCCATGGCCTGACCCCGGACCAAGTGGTGGCCATCGCCAGCCACGATGGCGGCAAGCA

GGCGCTGGAGACGGTGCAGCGGCTGTTGCCGGTGCTGTGCCAGGCCCATGGCCTGACCCCGGCGCAGGTGGTGGCCATCG

CCAGCAATATTGGCGGCAAGCAGGCGCTGGAGACGGTGCGGCGGCTGTTGCCGGTGCTGTGCCAGGCCC

>pCC2FOS-MscI-talC_8

CCATCGCCAGCAATGGCGGCGGCAAGCAGGCGCTGGAGACGGTGCAGCGGCTGTTGCCGGTGCTGTGCCAGGCCCATGGC

CTGACCCCGGCGCAGGTGGTGGCCATCGCCAGCAATAGCGGCGGCAAGCAGGCGCTGGAGACGGTGCAGCGGCTGTTGCC

GGTGCTGTGCCAGGCCCATGGCCTGACCCCGGACCAGGTGGTGGCCATCGCCAGCCACGATGGCGGCAAGCAGGCGCTGG

AGACGGTGCAGCGACTGTTGCCGGTGCTGTGCCAGGCCCATGGCCTGACCCCGGAGCAGGTGGTGGCCATCGCCAGCAAT

ATTGGCGGCAAGCAGGCGCTGGAGACGGTGCAGCGGCTGTTGCCGGTGCTGTGCCAGGCCCATGGCCTGACCCCGGAGCA

GGTGGTGGCCATCGCCAGCAATGGCGGCGGCAAGCAGGCGCTGGAGACGGTGCAGCGGTTGTTGCCGATGCTGTGCCAGG

CCCATGGCCTGACCCCGGAGCAGGTGGTGGCCATCGCCAGCAATAACGGCGGCAAGCAGGCGCTGGAGACGGTGCAGCGG

CTGTTGCCGGTGCTGTGCCAGGCCCATGGCCTGACCCCGGAGCAGGTGGTGGCCATCGCCAGCAATGGCGGCGGCAAGCA

GGCGCTGGAGACGGTGCAGCGGCTGTTGCCGGTGCTGTGCCAGGCCCATGGCCTGACCCCGGACCAAGTGGTGGCCATCG

CCAGCCACGATGGCGGCAAGCAGGCGCTGGAGACGGTGCAGCGGCTGTTGCCGG

>pCC2FOS-MscI-talC_9

CCATCGCCAGCAATAACGGCGGCAAGCAGGCCCTGGAGACGGTGCAGCGGCTGTTGCCGGTGCTGTGCCAGGCCCATGGC

CTGACCCCGGACCAGGTGGTGGCCATCGCCAGCCACGATGGCGGCAAGCAGGCGCTGGAGACGGTGCAGCGGCTGTTGCC

GGTGCTGTGCCAGGCCCATGGCCTGACCCTGGAGCAGGTGGTGGCCATCGCCAGCAATGGCGGCGGCAAGCAGGCGCTGG

AGACGGTGCAGCGGCTGTTGCCGGTGCTGTGCCAGGCCCATGGCCTGACCCCGGCGCAGGTGGTGGCCATCGCCTGCAAT

ATTGGCGGCAAGCAGGCGCTGGAGACGGTGCGGCGGCTGTTGCCGGTGCTGTGCCAGGCCCATGGCCTGACCCCGGCGCA

GGTGGTGGCCATCGCCAACAATAACGGCGGCAAGCAGGCGCTGGAGACGGTGCAGCGGCTGTTGCCGGTGCTGTGCCAGG

CCCATGGCCTGACCCCGGCGCAGGTGGTGGCCATCGCCAGCAATGGCGGCAAGCAGGCGCTGGAGACGGTGCAGCGGCTG

TTGCCGGTGCTGTGCCAGGCCCATGGTCTGACCCCGGCGCAGGTGGTGGCCATCGCCAGCCACGATGGCGGCAAGCAGGC

GTTGGAGACGGTGCAGCGGCTGTTGCCGGTGCTGTGCCAGGCCCATGGCCTGACCCCGGACCAGGTGGTGGCCATCGCCA

GCAATAACGGCGGCAAGCAGGCGCTGGAGACGGTGCAGCGGCTGTTGCCGGTGCT

>pCC2FOS-MscI-talC_10

CCATCGCCAGCAATGGCGGCAAGCAGGCGCTGGAGACGGTGCAGCGGCTGTTGCCGGTGCTGTGCCAGGCCCATGGCCTG

ACCCCGGAGCAGGTGGTGGCCATCGCCAGCAATATTGGCGGCAAGCAGGCGCTGGAGACGGTGCAGCGGCTGTTGCCGGT

GCTGTGCCAGGCCCATGGCCTGACCCCGGAGCAGGTGGTGGCCATCGCCAGCAATAACGGCGGCAAGCAGGCCCTGGAGA

CGGTGCAGCGGCTGTTGCCGGTGCTGTGCCAGGCCCATGGCCTGACCCCGGACCAGGTGGTGGCCATCGCCAGCCACGAT

GGCGGCAAGCAGGCGCTGGAGACGGTGCAGCGGCTGTTGCCGGTGCTGTGCCAGGCCCATGGCCTGACCCTGGAGCAGGT

GGTGGCCATCGCCAGCAATGGCGGCGGCAAGCAGGCGCTGGAGACGGTGCAGCGGCTGTTGCCGGTGCTGTGCCAGGCCC

ATGGCCTGACCCCGGCGCAGGTGGTGGCCATCGCCTGCAATATTGGCGGCAAGCAGGCGCTGGAGACGGTGCGGCGGCTG

TTGCCGGTGCTGTGCCAGGCCCATGGCCTGACCCCGGCGCAGGTGGTGGCCATCGCCAACAATAACGGCGGCAAGCAGGC

GCTGGAGACGGTGCAGCGGCTGTTGCCGGTGCTGTGCCAGGCCCATGGCCTGACCCCGGCGCAGGTGGTGGCCATCGCCA

GCAATGGCGGCAAGCAGGCGCTGGAGACGGTGCAGCGGCTGTTGCCGGTGCTGTGCCAGGCCCATGGTCTGACCCCGGCG

CAGGTGGTGGCCATCGCCAGCCACGATGGCGGCAAGCAGGCGTTGGAGACGGTGCAGCGGCTGTTGCCGGTGCT

>pCC2FOS-MscI-talC_11

CGGCGGCAAGCAGGCGCTGGAGACGGTGCAGCGGCTGTTGCCGGTGCTGTGCCAGGCCCATGGCCTGACCCCGGAGCAGG

TGGTGGCCATCGCCAGCAATGGCGGCGGCAAGCAGGCGCTGGAGACGGTGCAGCGGCTGTTGCCGGTGCTGTGCCAGGCC

CATGGCCTGACCCCGGCGCAGGTGGTGGCCATCGCCAGCAATAGCGGCGGCAAGCAGGCGCTGGAGACGGTGCAGCGGCT

GTTGCCGGTGCTGTGCCAGGCCCATGGCCTGACCCCGGACCAGGTGGTGGCCATCGCCAGCCACGATGGCGGCAAGCAGG

CGCTGGAGACGGTGCAGCGACTGTTGCCGGTGCTGTGCCAGGCCCATGGCCTGACCCCGGAGCAGGTGGTGGCCATCGCC

AGCAATATTGGCGGCAAGCAGGCGCTGGAGACGGTGCAGCGGCTGTTGCCGGTGCTGTGCCAGGCCCATGGCCTGACCCC

GGAGCAGGTGGTGGCCATCGCCAGCAATGGCGGCGGCAAGCAGGCGCTGGAGACGGTGCAGCGGTTGTTGCCGATGCTGT

GCCAGGCCCATGGCCTGACCCCGGAGCAGGTGGTGGCCATCGCCAGCAATAACGGCGGCAAGCAGGCGCTGGAGACGGTG

CAGCGGCTGTTGCCGGTGCTGTGCCAGGCCCATGGCCTGACCCCGGAGCAGGTGGTGGCCATCGCCAGCAATGGCGGCGG

CAAGCAGGCGCTGGAGACGGTGCAGCGGCTGTTGCCGGTGCTGTGCCAGGCCCATGGCCTGACCCCGGACCAAGTGGTGG

CCATCGCCAGCCACGATGGCGGCAAGCAGGCGCTGGAGACGGTGCAGCGGCTGTTGCCGGTGCTGTGCCAGGCCCATGGC

CTGACCCCGGCGCAGGTGGTGGCCATCGCCAGCAATATTGGCGGCAAGCAGGCGCTGGAGACGGTGCAGCGGCTGTTGCC

GGTGCTGTGCCAGGCCC

>pCC2FOS-MscI-talC_11

CCATCGCCAGCAATGGCGGCAAGCAGGCGCTGGAGACGGTGCAGCGGCTGTTGCCGGTGCTGTGCCAGGCCCATGGCCTG

ACCCCGGAGCAGGTGGTGGCCATCGCCAGCAATATTGGCGGCAAGCAGGCGCTGGAGACGGTGCAGCGGCTGTTGCCGGT

GCTGTGCCAGGCCCATGGCCTGACCCCGGAGCAGGTGGTGGCCATCGCCAGCAATAACGGCGGCAAGCAGGCCCTGGAGA

CGGTGCAGCGGCTGTTGCCGGTGCTGTGCCAGGCCCATGGCCTGACCCCGGACCAGGTGGTGGCCATCGCCAGCCACGAT

GGCGGCAAGCAGGCGCTGGAGACGGTGCAGCGGCTGTTGCCGGTGCTGTGCCAGGCCCATGGCCTGACCCTGGAGCAGGT

GGTGGCCATCGCCAGCAATGGCGGCGGCAAGCAGGCGCTGGAGACGGTGCAGCGGCTGTTGCCGGTGCTGTGCCAGGCCC

ATGGCCTGACCCCGGCGCAGGTGGTGGCCATCGCCTGCAATATTGGCGGCAAGCAGGCGCTGGAGACGGTGCGGCGGCTG

TTGCCGGTGCTGTGCCAGGCCCATGGCCTGACCCCGGCGCAGGTGGTGGCCATCGCCAACAATAACGGCGGCAAGCAGGC

GCTGGAGACGGTGCAGCGGCTGTTGCCGGTGCTGTGCCAGGCCCATGGCCTGACCCCGGCGCAGGTGGTGGCCATCGCCA

GCAATGGCGGCAAGCAGGCGCTGGAGACGGTGCAGCGGCTGTTGCCGGTGCTGTGCCAGGCCCATGGTCTGACCCCGGCG

CAGGTGGTGGCCATCGCCAGCCACGATGGCGGCAAGCAGGCGTTGGAGACGGTGCAGCGGCTGTTGCCGGTGCT

>pCC2FOS-MscI-talC_12

CCATCGCCAGCAATGGCGGCGGCAAGCAGGCGCTGGAGACGGTGCAGCGGTTGTTGCCGATGCTGTGCCAGGCCCATGGC

CTGACCCCGGAGCAGGTGGTGGCCATCGCCAGCAATAACGGCGGCAAGCAGGCGCTGGAGACGGTGCAGCGGCTGTTGCC

GGTGCTGTGCCAGGCCCATGGCCTGACCCCGGAGCAGGTGGTGGCCATCGCCAGCAATGGCGGCGGCAAGCAGGCGCTGG

AGACGGTGCAGCGGCTGTTGCCGGTGCTGTGCCAGGCCCATGGCCTGACCCCGGACCAAGTGGTGGCCATCGCCAGCCAC

GATGGCGGCAAGCAGGCGCTGGAGACGGTGCAGCGGCTGTTGCCGGTGCTGTGCCAGGCCCATGGCCTGACCCCGGCGCA

GGTGGTGGCCATCGCCAGCAATATTGGCGGCAAGCAGGCGCTGGAGACGGTGCGGCGGCTGTTGCCGGTGCTGTGCCAGG

CCCATGGCCTGACCCCGGCGCAGGTGGTGGCCATCGCCAACAATAACGGCGGCAAGCAGGCGCTGGAGACGGTGCAGCGG

CTGTTGCCGGTGCTGTGCCAGGCCCATGGCCTGACCCCGGAGCAGGTGGTGGCCATCGCCAGCAATGGCGGCAAGCAGGC

GCTGGAGACGGTGCAGCGGCTGTTGCCGGTGCTGTGCCAGGCCCATGGCCTGACCCCGGAGCAGGTGGTGGCCATCGCCA

GCAATATTGGCGGCAAGCAGGCGCTGGAGACGGTGCAGCGGCTGTTGCCGGTGCTGTGCCAGGCCCATGGCCTGACCCCG

GAGCAGGTGGTGGCCATCGCCAGCAATAACGGCGGCAAGCAGGCCCTGGAGACGGTGCAGCGGCTGTTGCCGGTGC

>pCC2FOS-MscI-talC_13

CCATCGCCAGCAATATTGGCGGCAAGCAGGCGCTGGAGACGGTGCGGCGGCTGTTGCCGGTGCTGTGCCAGGCCCATGGC

CTGACCCCGGCGCAGGTGGTGGCCATCGCCAACAATAACGGCGGCAAGCAGGCGCTGGAGACGGTGCAGCGGCTGTTGCC

GGTGCTGTGCCAGGCCCATGGCCTGACCCCGGAGCAGGTGGTGGCCATCGCCAGCAATGGCGGCAAGCAGGCGCTGGAGA

CGGTGCAGCGGCTGTTGCCGGTGCTGTGCCAGGCCCATGGCCTGACCCCGGAGCAGGTGGTGGCCATCGCCAGCAATATT

GGCGGCAAGCAGGCGCTGGAGACGGTGCAGCGGCTGTTGCCGGTGCTGTGCCAGGCCCATGGCCTGACCCCGGAGCAGGT

GGTGGCCATCGCCAGCAATAACGGCGGCAAGCAGGCCCTGGAGACGGTGCAGCGGCTGTTGCCGGTGCTGTGCCAGGCCC

ATGGCCTGACCCCGGACCAGGTGGTGGCCATCGCCAGCCACGATGGCGGCAAGCAGGCGCTGGAGACGGTGCAGCGGCTG

TTGCCGGTGCTGTGCCAGGCCCATGGCCTGACCCTGGAGCAGGTGGTGGCCATCGCCAGCAATGGCGGCGGCAAGCAGGC

GCTGGAGACGGTGCAGCGGCTGTTGCCGGTGCTGTGCCAGGCCCATGGCCTGACCCCGGCGCAGGTGGTGGCCATCGCCT

GCAATATTGGCGGCAAGCAGGCGCTGGAGACGGTGCGGCGGCTGTTGCCGGTGCTGTGCCAGGCCCATG

>pCC2FOS-MscI-talC_14

CCATCGCCAGCCACGATGGCGGCAAGCAGGCGCTGGAGACGGTGCAGCGGCTGTTGCCGGTGCTGTGCCAGGCCCATGGC

CTGACCCCGGCGCAGGTGGTGGCCATCGCCAGCAATATTGGCGGCAAGCAGGCGCTGGAGACGGTGCGGCGGCTGTTGCC

GGTGCTGTGCCAGGCCCATGGCCTGACCCCGGCGCAGGTGGTGGCCATCGCCAACAATAACGGCGGCAAGCAGGCGCTGG

AGACGGTGCAGCGGCTGTTGCCGGTGCTGTGCCAGGCCCATGGCCTGACCCCGGAGCAGGTGGTGGCCATCGCCAGCAAT

GGCGGCAAGCAGGCGCTGGAGACGGTGCAGCGGCTGTTGCCGGTGCTGTGCCAGGCCCATGGCCTGACCCCGGAGCAGGT

GGTGGCCATCGCCAGCAATATTGGCGGCAAGCAGGCGCTGGAGACGGTGCAGCGGCTGTTGCCGGTGCTGTGCCAGGCCC

ATGGCCTGACCCCGGAGCAGGTGGTGGCCATCGCCAGCAATAACGGCGGCAAGCAGGCCCTGGAGACGGTGCAGCGGCTG

TTGCCGGTGCTGTGCCAGGCCCATGGCCTGACCCCGGACCAGGTGGTGGCCATCGCCAGCCACGATGGCGGCAAGCAGGC

GCTGGAGACGGTGCAGCGGCTGTTGCCGGTGCTGTGCCAGGCCCATGGCCTGACCCTGGAGCAGGTGGTGGCCATCGCCA

GCAATGGCGGCGGCAAGCAGGCGCTGGAGACGGTGCAGCGGCTGTTGCCGGTGCTGTGCCAGGCCCATGGC

>pCC2FOS-MscI-talC_15

CCATCGCCAGCAATGGCGGCGGCAAGCAGGCGCTGGAGACGGTGCAGCGGTTGTTGCCGATGCTGTGCCAGGCCCATGGC

CTGACCCCGGAGCAGGTGGTGGCCATCGCCAGCAATAACGGCGGCAAGCAGGCGCTGGAGACGGTGCAGCGGCTGTTGCC

GGTGCTGTGCCAGGCCCATGGCCTGACCCCGGAGCAGGTGGTGGCCATCGCCAGCAATGGCGGCGGCAAGCAGGCGCTGG

AGACGGTGCAGCGGCTGTTGCCGGTGCTGTGCCAGGCCCATGGCCTGACCCCGGACCAAGTGGTGGCCATCGCCAGCCAC

GATGGCGGCAAGCAGGCGCTGGAGACGGTGCAGCGGCTGTTGCCGGTGCTGTGCCAGGCCCATGGCCTGACCCCGGCGCA

GGTGGTGGCCATCGCCAGCAATATTGGCGGCAAGCAGGCGCTGGAGACGGTGCGGCGGCTGTTGCCGGTGCTGTGCCAGG

CCCATGGCCTGACCCCGGCGCAGGTGGTGGCCATCGCCAACAATAACGGCGGCAAGCAGGCGCTGGAGACGGTGCAGCGG

CTGTTGCCGGTGCTGTGCCAGGCCCATGGCCTGACCCCGGAGCAGGTGGTGGCCATCGCCAGCAATGGCGGCAAGCAGGC

GCTGGAGACGGTGCAGCGGCTGTTGCCGGTGCTGTGCCAGGCCCATGGCCTGACCCCGGAGCAGGTGGTGGCCATCGCCA

GCAATATTGGCGGCAAGCAGGCGCTGGAGACGGTGCAGCGGCTGTTGCCGGTGCTGTGCCAGGCCCATGGCCTGACCCCG

GAGCAGGTGGTGGCCATCGCCAGCAATAACGGCGGCAAGCAGGCCCTGGAGACGGTGCAGCGGCTGTTGCCGGTGCTGTG

CCAGGCCCATGGCC

>talC_M13F

GGATCCCATTCGTCCGCGCGCGCCAAGTCCTGCCCGCGAGGTTCTGCCCGGCCCCCAACCGGATAGGGTTCAGCCGACTG

CAGATCGTGGGGTGTCTGCGCCTGCTGGCAGCCCTCTGGATGGCTTGCCCGCTCGGCGGACGATGTCCCGGACCCGGCTG

CCATCTCCCCCTGCCCCCTTGCCTGCGTTCTCGGCGGGCAGCTTCAGCGATCTGCTCCGTCAGTTCGATCCGTCGCTTCT

TGATACATCGCTTTTTGATTCGATGCCTGCCGTCGGCACGCCTCATACAGAGGCTGCCCCAGCAGAGGGGGATGAGGTGC

AATCGGCTCTGCGTGCAGCCGATGACCCGCCACCCACCGTGCGTGTCGCTGTCACTGCCGCGCAGGTGGATCTACGCACG

CTCGGCTACAGTCAGCAGCAAGAGAAGATCAAACCGAATGTTCGTTCGACAGTGGCGCAGCACCACGAGGCACTGGTGGG

CCATGGGTTTACACACGCGCACATCGTTGCGCTCAGCCGACACCCGGCAGCGTTAGGGACCGTCGCTGTCAAGTATCAGG

ACATGATCGCGGCGTTACCAGAGGCGACACACGAAGACATCGTTGGGGTCGGCAAACAGTGTTCCGGCGCACGCGCCCTG

GAGGCCTTGCTCACGGTGGCGGGAGAGTTGAGAGGTCCACCGTTACAGTTGGACACAGGCCAACTTGTCAAGATTGCAAA

ACGTGGCGGCGTGACCGCAGTGGAGGCAGTGCATGCATCGCGCAATGCACTGACGGGTGCCCCCCTGAACCTGACCCCGG

CACAGGTGGTGGCCATCGCCAGCAATAGCGGCGGCAAGCAGGCGCTGGAGACGGTGCAGCGGCTGTTGCCGGTGCTGTGC

CAGGCCCATGGCCTGACCCCGGAGCAGGTGGTGGCCATCGCCAGCAATGGCGGCGGCAAGCAGGCGCTGGAGACGGTGCA

GCGGC

>talC_M13R

CAGGTGGTGGCCATCGCCAGCCACGATGGCGGCAAGCAGGCGTTGGAGACGGTGCAGCGGCTGTTGCCGGTGCTGTGCCA

GGCCCATGGCCTGACCCCGGACCAGGTGGTGGCCATCGCCAGCAATAACGGCGGCAAGCAGGCGCTGGAGACGGTGCAGC

GGCTGTTGCCGGTGCTGTGCCAGGCCCATGGCCTGACCCCGGAGCAGGTGGTGGCCATCGCCAGCAATGGCGGCGGCAAG

CAGGCGCTGGAGAGCATTGTTGCCCAGTTATCTCGCCCTGATCCGGCGTTGGCCGCGTTGACCAACGACCACCTCGTCGC

CTTGGCCTGCCTCGGCGGACGTCCTGCCCTGGATGCAGTGAAAAAGGGATTGCCGCACGCGCCGGAATTGATCAGAAGAG

TCAATAGCCGTATTGCCGAACGCACGTCCGATCGCGTTACCGACTACGCGCAAGTGGTTCGCGTGCTGGAGTTTTTCCAG

TGCCACTCCCACCCAGCGCACGCATTTGATGAGGCCATGACGCAGTTCGGTATGAGCAGGAACGGATTGTTACAGCTCTT

TCGCAGAGTGGGCGTCACCGAACTCGAAGCCTGCGGTGGAACGCTCCCCCCAGCCTCGCAGCGTTGGCACCGTATCCTCC

AAGCATCAGGGATGAAAAGTGCCAAACCGTCCTGTGCTTCGGCTCAAACGCCGGATCAGGCGTCTTTGCATGCATTCGCC

GATTCGCCGGAGCGTGACCTTGATGCGCCCAGCCCAATGCACGAGGGAGATCAGACGCGGGCAAGCAGCCGTAAACGGTC

CCGATCGGATCGTGCTGTCACCGGCCCCTCCGCACAGCAGGCTGTCGAGGTGCGCGTTCCCGAACAGCGCGATGCGCTGC

ATTTGCCCCTCAGCTGGAGTGTAAAACGCCCGCGTACCAGGATCGGGGGCGGCCTCCCGGATCC

>talC_R_seqF

AGTTGAGAGGTCCACCGTTACAGTTGGACACAGGCCAACTTGTCAAGATTGCAAAACGTGGCGGCGTGACCGCAGTGGAG

GCAGTGCATGCATCGCGCAATGCACTGACGGGTGCCCCCCTGAACCTGACCCCGGCACAGGTGGTGGCCATCGCCAGCAA

TAGCGGCGGCAAGCAGGCGCTGGAGACGGTGCAGCGGCTGTTGCCGGTGCTGTGCCAGGCCCATGGCCTGACCCCGGAGC

AGGTGGTGGCCATCGCCAGCAATGGCGGCGGCAAGCAGGCGCTGGAGACGGTGCAGCGGCTGTTGCCGGTGCTGTGCCAG

GCCCATGGCCTGACCCCGGCGCAGGTGGTGGCCATCGCCAGCAATAGCGGCGGCAAGCAGGCGCTGGAGACGGTGCAGCG

GCTGTTGCCGGTGCTGTGCCAGGCCCATGGCCTGACCCCGGACCAGGTGGTGGCCATCGCCAGCCACGATGGCGGCAAGC

AGGCGCTGGAGACGGTGCAGCGACTGTTGCCGGTGCTGTGCCAGGCCCATGGCCTGACCCCGGAGCAGGTGGTGGCCATC

GCCAGCAATATTGGCGGCAAGCAGGCGCTGGAGACGGTGCAGCGGCTGTTGCCGGTGCTGTGCCAGGCCCATGGCCTGAC

CCCGGAGCAGGTGGTGGCCATCGCCAGCAATGGCGGCGGCAAGCAGGCGCTGGAGACGGTGCAGCGGTTGTTGCCGATGC

TGTGCCAGGCCCATGGCCTGACCCCGGAGCAGGTGGTGGCCATCGCCAGCAATAACGGCGGCAAGCAGGCGCTGGAGACG

GTGCAGCGGCTGTTGCCGGTGCTGTGCCAGGCCCATGGCCTGACCCCGGAGCAGGTGGTGGCCATCGCCAGCAATGGCGG

CGGCAAGCAGGCGCTGGAGACGGTGCAGCGGCTGTTGCCGGTGCTGTGCCAGGCCCATGGCCTGACCCCGGACCAAGTGG

TGGCCATCGCC

>talC_R_seqR

GTGGCCATCGCCAGCAATATTGGCGGCAAGCAGGCGCTGGAGACGGTGCAGCGGCTGTTGCCGGTGCTGTGCCAGGCCCA

TGGCCTGACCCCGGAGCAGGTGGTGGCCATCGCCAGCAATAACGGCGGCAAGCAGGCCCTGGAGACGGTGCAGCGGCTGT

TGCCGGTGCTGTGCCAGGCCCATGGCCTGACCCCGGACCAGGTGGTGGCCATCGCCAGCCACGATGGCGGCAAGCAGGCG

CTGGAGACGGTGCAGCGGCTGTTGCCGGTGCTGTGCCAGGCCCATGGCCTGACCCTGGAGCAGGTGGTGGCCATCGCCAG

CAATGGCGGCGGCAAGCAGGCGCTGGAGACGGTGCAGCGGCTGTTGCCGGTGCTGTGCCAGGCCCATGGCCTGACCCCGG

CGCAGGTGGTGGCCATCGCCTGCAATATTGGCGGCAAGCAGGCGCTGGAGACGGTGCGGCGGCTGTTGCCGGTGCTGTGC

CAGGCCCATGGCCTGACCCCGGCGCAGGTGGTGGCCATCGCCAACAATAACGGCGGCAAGCAGGCGCTGGAGACGGTGCA

GCGGCTGTTGCCGGTGCTGTGCCAGGCCCATGGCCTGACCCCGGCGCAGGTGGTGGCCATCGCCAGCAATGGCGGCAAGC

AGGCGCTGGAGACGGTGCAGCGGCTGTTGCCGGTGCTGTGCCAGGCCCATGGTCTGACCCCGGCGCAGGTGGTGGCCATC

GCCAGCCACGATGGCGGCAAGCAGGCGTTGGAGACGGTGCAGCGGCTGTTGCCGGTGCTGTGCCAGGCCCATGGCCTGAC

CCCGGACCAGGTGGTGGCCATCGCCAGCAATAACGGCGGCAAGCAGGCGCTGGAGACGGTGCAGCGGCTGTTGCCGGTGC

TGTGCCAGGCCCATGGCCTGACCCCGGAGCAGGTGGTGGCCATCGCCAGCAATGGCGGCGGCAAGCAGGCGCTGGAGAGC

ATTGTTGCCCAG
