# Supplementary material for: Rapid and efficient genome-wide characterization of Xanthomonas TAL effector genes
Source: Sci Rep. 2015 Aug 14;5:13162. doi: 10.1038/srep13162 (PMC4536657; doi:10.1038/srep13162)
Supplement: Supplementary Data 2 [file srep13162-s3.doc]

**Supplementary DNA sequence data 2**. Assembled DNA sequences of the 18 unique *tal*-*Bam*HI fragments of *Xoo* strain K74 and the *tal-Bam*HI fragment of *talC*.

>***tal*-*Bam*HI-1 (*tal_Xoo_*_K74_-6)**

GGATCCCATTCGTTCGCGCACGCCAAGTCCTGCCCGCGAGCTTCTGCCCGGACCCCAACCGGATAGGGTTCAGCCGACTGCAGATCGGGGGGGGGCTCCGCCTGCTGGCGGCCCCCTGGATGGCTTGCCCGCTCGGCGGACGATGTCCCGGACCCGGCTGCCATCTCCCCCTGCGCCCTCGCCTGCGTTCTCGGCGGGCAGCTTCAGCGATCTGCTCCGTCAGTTCGATCCGTCGCTTCTTGATACATCGCTTCTTGATTCGATGCCTGCCGTCGGCACGCCGCATACAGCGGCTGCCCCAGCAGAATGGGATGAGGCGCAATCGGGTCTGCGTGCAGCCGATGACCCGCCACCCACCGTGCCTGTCGCTGTCACTGCCGCGCGGCCGCCGCGCGCCAAGCCGGCCCCGCGACGGCGTGCGGCGCAACCCTCCGACGCTTCGCCGGCCGCGCAGGTGGATCTACGCACGCTCGGCTACAGTCAGCAGCAGCAAGAGAAGATCAAACCGAAGGTGCGTTCGACAGTGGCGCAGCACCACGAGGCACTGGTGGGCCATGGGTTTACACACGCGCACATCGTTGCGCTCAGCCAACACCCGGCAGCGTTAGGGACCGTTGCTGTCACGTATCAGGACATAATCAGGGCGTTGCCAGAGGCGACACACGAAGACATCGTTGGCGTCGGCAAACAGTGGTCCGGCGCACGCGCCCTGGAGGCCTTGCTCACGAAGGCGGGGGAGTTGAGAGGTCCACCGTTACAGTTGGACACAGGCCAACTTGTCAAGATTGCAAAACGTGGCGGCGTGACCGCAGTGGAGGCAGTGCATGCATCGCGCAATGCACTGACGGGTGCCCCCCTGAACCTGACCCCGGACCAAGTGGTGGCCATCGCCAGCAATATTGGCGGCAAGCAGGCGCTGGAGACGGTGCAGCGCCTGTTGCCGGTGCTGTGCCAGGACCATGGCCTGACCCCGGACCAGGTGGTGGCCATCGCCAACAATAACGGCGGCAAGCAGGCGCTGGAGACGGTGCAGCGGCTGTTGCCGGTGCTGTGCCAGGACCATGGCCTGACCCCGGACCAGGTGGTGGCCATCGCCAGCAATATTGGCGGCAAGCAGGCGCTGGAGACGGTGCAGCGGCTGTTGCCGGTGCTGTGCCAGGCCCATGGCCTGACCCCGGCCCAGGTGGTGGCCATCGCCAGCAATAACGGCGGCAAGCAGGCGCTGGAGACGGTGCAGCGGCTGTTGCCGGTGCTGTGCCAGGCCCATGGCCTGACCCCGGACCAGGTGGTGGCTATCGCCAGCAATAACGGCGGCAAGCAGGCGCTGGAGACGGTGCAACGGCTGTTGCCGGTGCTGTGCCAGGCCCATGGCCTGACCCCGGACCAAGTGGTGGCCATCGCCAACAATAACGGCGGCAAGCAGGCGCTGGAGACGGTGCAGCGGCTGTTGCCGGTGCTGTGCCAGGACCATGGCCTGAGTCCGGACCAGGTGGTGGCCATCGCCAACAATAACGGCGGCAAGCAGGCGCTGGAGACGCTGCAGCGGCTGTTGCCGGTGCTGTGCCAGACCCATGCCCTGACCCCGGACCAGGTGGTGGCCATCGCCAACAATAACGGCGGCAAGCAGGCGCTGGAGACGGTGCAGCGGCTGTTGCCGGTGCTGTGCCAGGACCATGGCCTGACCCCGGACCAGGTGGTGGCCATCGCCAGCCACGATGGCGGCAAGCAGGCGCTGGAGACGGTGCAGCGGCTGTTGCCGGTGCTGTGCCAGGACCATGGCCTGACCCCGGACCAAGTGGTGGCCATCGCCAGCAATATTGGCGGCAAGCAGGCGCTGGAGACGGTGCAGCGGCTGTTGCCGGTGCTGTGCCAGGACCATGGCCTGACCCCGGACCAGGTGGTGGCCATCGCCAGCAATAGTGGCGGCAAGCAGGCGCTGGAGACGGTGCAGCGGCTGTTGCCGGTGCTGTGCCAGGACCATGGCCTGACCCCGGACCAGGTGGTGGCCATCGCCAGCCATGGCGGCGGCAAGCAGGCGCTGGAGACGGTGCAGCGGCTGTTGCCGGTGCTGTGCCAGGACCATGGCCTGACCCCAGACCAGGTCGTGGCCATCGCCAGCCACGATGGCGGCAAGCAGGCGCTGGAGACGGTGCAGCGGCTGTTGCCGGTGCTGTGCCAGGCCCATGGCCTGACCCCGAACCAGGTGGTGGCCATCGCCAGCAATATTGGCGGCAAGCAGGCGCTGGAGACGGTGCAACGGCTGTTGCCGGTGCTGTGCCAGGACCATGGCCTGACCCCGGACCAGGTCGTGGCCATCGCCAGCAATGGCGGCAAGCAGGCGCTGGAGACGGTGCAGCGGCTGTTGCCGGTGCTGTGCCAGGCTCATGGCCTGACCCCGGACCAAGTGGTGGCCATCGCCAGTAATAGTGGCGGCAAGCAGGCGCTGGAGACGGTGCAGCGGCTGTTGCCGGTGCTGTGCCAGGACCATGGCCTGACCCCGAACCAAGTGGTGGCCATCGCCAGCAATATTGGCGGCAAGCAGGCGCTGGAGACGGTGCAGCGGCTGTTGCCGGTGCTATGCCAGGACCATGGCCTGACCCCGGACCAGGTGGTGGCCATCGCCAGCAATATTGGCGGCAAGCAGGCGCTGGAGACGGTGCAGCGGCTGTTGCCGGTGCTGTGCCAGGACCATGGCCTGACCCTGGACCAGGTGGTGGCCATCGCCAGCCACGATGGCGGCAAACAGGCGCTGGAGACGGTGCAGCGGCTGTTGCCGGTGCTGTGCCAGGCCCATGGCCTGACCCCGGACCAGGTCGTGGCCATCGCCAGCCACGATGGCGGCAAGCAGGCGCTGGAGACGGTGCAGCGGCTGTTGCCGGTGCTGTGCCAGGCCCATGGCCTGACCCTGGACAAGGTGGTGGCCATCGCCAGCAATGGCGGCAAGCAGGCGCTGGAGACGGTGCAGCGGCTGTTGCCGGTGCTGTGCCAGGACCATGGCCTGACCCCGAACCAGGTGGTGGCCATCGCCAGCAATAGTGGCGGCAAGCAGGCGCTGGAGACGGTGCAGCGGCTGTTGCCGGTGCTGTGCCAGGACCATGGCCTGACCCCGAACCAGGTGGTGGCCATCGCCAGCAATGGCGGCAAGCAGGCGCTGGAGAGCATTGTTGCCCAGTTATCTCGCCCTGATCCGGCGTTGGCCGCGTTGACCAACGACCACCTCGTCGCCTTGGCCTGCCTCGGCGGACGTCCTGCCCTGGATGCAGTGAAAAAGGGATTGCCGCACGCGCCGGAATTGATCAGAAGAATCAATCGCCGCATTCCCGAACGCACGTCCCATCGCGTTCCCGACCTCGCGCACGTGGTTCGCGTGCTTGGTTTTTTCCAGAGCCACTCCCACCCAGCGCAAGCATTCGATGACGCCATGACGCAGTTCGAGATGAGCAGGCACGGCTTGGTACAGCTCTTTCGCAGAGTGGGCGTCACCGAATTCGAAGCCCGCTACGGAACGCTCCCCCCAGCCTCGCAGCGTTGGGACCGTATCCTCCAGGCATCAGGGATGAAAAGGGCCAAACCGTCCCCTACTTCAGCTCAAACACCGGATCAGGCGTCTTTGCATGCATTCGCCGATTCGCTGGAGCGTGACCTTGATGCGCCTAGCCCAATGCACGAGGGAGATCAGACAGGGGCAAGCAGCCGTAAACGGTCCCGATCGGATCGTGCTGTCACCGGCCCCTCCGCACAGCAATCTTTCGAGGTGCGCGTTCCCGAACAGCGCGATGCGCTGCATTTGCCCCTCAGCTGGAGGGTAAAACGCCCGCGTACCAGGATCGGGGGCGGCCTCCCGGATCC

>***tal*-*Bam*HI-6 (*tal_Xoo_*_K74_-12)**

GGATCCCATTCGTTCGCGCACGCCAAGTCCTGCCCGCGAGCTTCTGCCCGGACCCCAACCGGATAGGGTTCAGCCGACTGCAGATCGGGGGGGGGCTCCGCCTGCTGGCGGCCCCCTGGATGGCTTGCCCGCTCGGCGGACGATGTCCCGGACCCGGCTGCCATCTCCCCCTGCGCCCTCGCCTGCGTTCTCGGCGGGCAGCTTCAGCGATCTGCTCCGTCAGTTCGATCCGTCGCTTCTTGATACATCGCTTCTTGATTCGATGCCTGCCGTCGGCACGCCGCATACAGCGGCTGCCCCAGCAGAATGGGATGAGGCGCAATCGGGTCTGCGTGCAGCCGATGACCCGCCACCCACCGTGCGTGTCGCTGTCACTGCCGCGCGGCCGCCGCGCGCCAAGCCGGCCCCGCGACGGCGTGCGGCGCAACCCTCCGACGCTTCGCCGGCCGCGCAGGTGGATCTACGCACGCTCGGCTACAGTCAGCAGCAGCAAGAGAAGATCAAACCGAAGGTGCGTTCGACAGTGGCGCGGCACCACGAGGCACTGGTGGGTCATGGGTTTACACACGCGCACATCGTTGCGCTCAGCCAACACCCGGCAGCGTTAGGGACCGTTGCTGTCACGTATCAGGACATAATCAGGGCGTTGCCAGAGGCGACACACGAAGACATCGTTGGCGTCGGCAAACAGTGGTCCGGCGCACGCGCCCTGGAGGCCTTGCTCACGGAGGCGGGGGAGTTGAGAGGTCCGCCGTTACAGTTGGACACAGGCCAACTTCTCAAGATTGCAAAACGTGGCGGCGTGACCGCAGTGAAGGCAGTGCATGCATGGCGCAATGCACTGACGGGTGCCCCCCTGAACCTGACCCCGGCACAGGTGGTGGCCATCGCCAGCCACGATGGCGGCAATCAGGCGCTGGAGACGGTGCAGCGGCTGTTGCCGGTGCTGTGCCAGGACCATGGCCTGACCCCGGCGCAGGTGGTGGCCATCGCCAGCCACGATGGCGGCAAGCAGGCTCTGGAGACGGTGCAGCGGCTGTTGCCGGTGCTGTGCCAGGACCATGGCCTGACCCCGGACCAGGTCGTGGCCATCGCCAGCCACGATGGCGGCAAGCAGGCGCTGGAGACGGTGCAGCGGCTGTTGCCGGTGCTGTGCCAGGACCATGGCCTGACCCCGGCGCAGGCGGTGGCCATCGCCAGCAATGGCGGCGGCAAGCAGGCGCTGGAGACGGTGCAACGGCTGTTGCCGGTGCTGTGCCAGGACCATGGCCTGACCCCGGACCAGGTGGTGGCCATCGCCAGCAATGGCGGCAAGCAGGCGCTGGAGACGGTGCAGCGGCTGTTGCCGGTGCTGTGCCAGGACCATGGCCTGACCCCGGACCAGGTCGTGGCCATTGCCAGCAATAACGGCGGCAAGCAGGCGCTGGAGACGGTGCAGCGGCTGTTGCCGGTGCTGTGCCAGGACCATGGCCTGACCCCGGACCAGGTCGTGGCCATCGCCAGCCACGATGGCGGCAAGCAGGCGCTGGAGACGGTGCAGCGGCTGTTGCCGGTGCTGTGCCAGGACCATGGCCTGACCCCGGACCAGGTCGTGGCCATCGCCAGCCACGATGGCGGCAAGCAGGCGCTGGAGACGGTGCAGCGGCTGTTGCCGGTGCTGTGCCAGGACCATGGCCTGACCCCGGACCAGGTGGTGGCCATCGCCAGCAATGGCGGCAAGCAGGCGCTGGAGACGGTGCAGCGGCTGTTGCCGGTGCTGTGCCAGGACCATGGCCTGACCCTGGCGCAGGTGGTGGCCATCGCCAGCAATATTGGCGGCAAGCAGGCGCTGGAGACGGTGCAGCGGCTGTTGCCGGTGCTGTGCCAGGACCATGGCCTGACCCCGGACCAGGTCGTGGCCATCGCCAGTAATATTGGCGGCAAGCAGGCGCTGGAGACGGTGCAGCGGCTGTTGCCGGTGCTGTGCCAGGACCATGGCCTGACCCCGGACCAGGTCGTGGCCATCGCCAGCAATAACGGCGGCAAGCAGGCGCTGGAGACGGTGCAGCGGCTGTTGCCGGTGCTGTGCCAGGACCATGGCCTGACCCCGGACCAGGTCGTGGCCATCGCCAGCCACGATGGCGGCAAGCAGGCGCTGGAGACGGTGCAGCGGCTGTTGCCGGTGCTGTGCCAGGACCATGGCCTGACCCCGGCGCAGGTGGTGGCCATCGCCAGCCATATTGGCGGCAAGCAGGCGCTGGAGACGGTGCAGCGGCTGTTGCCGGTGCTGTGCCAGGACCATGGCCTGACCCTGGACCAGGTGGTGGCCATTGCCAGCAATGACGGCAGCAAGCAGGCGCTGGAGACGGTGCAGCGGCTGTTGCCGGTGCTGTGCCAGGACCATGGCCTGACCCCGGACCAGGTGGTGGCCATCGCCAGCCACGATGGCGGCAAGCAGGCGCTGGAGACGGTGCAGCGGCTGTTGCCGGTGCTGTGCCAGGACCATGGCCTGACCCCGAACCAAGTGGTGGCCATCGCCAGCAATATTGGCGGCAAGCAGGCGCTGGAGACGGTGCAGCGGCTGTTGCCGGTGCTGTGCCAGGCCCATGGCCTGACCCCGGACCAGGTCGTGGCCATCGCCAGCCACGATGGCGGCAAGCAGGCGCTGGAGACGGTGCAGCGGCTGTTGCCGGTGCTGTGCCAGAACCATGGCCTGACCCCGGACCAGGTCGTGGCCATCGCCAGCAATGGCGGCGGCAAGCAGGCGCTGGAGACGGTGCAACGGCTGTTGCCGGTGCTGTGCCAGGACCATGGCCTGACCCCGGACCAGGTCGTGGCCATCGCCAGCAATGGCGGCGGCAAGCAGGCGCTGGAGAGCATTGTTGCCCAGTTATCTCGCCCTGATCCGGCGTTGGCCGCGTTGACCAACGACCACCTCGTCGCCTTGGCCTGCCTCGGCGGACGTCCTGCCCTGGATGCAGTGAAAAAGGGATTGCCGCACGCGCCGGAATTGATCAGAAGAATCAATCGCCGTATTCCCGAACGCACGTCCCATCGCGTTGCCGACTACGCGCAAGTGGTTCGCGTGCTGGAGTTTTTCCAGTGCCACTCCCACCCAGCGTACGCATTTGATGAGGCCATGACGCAGTTCGGGATGAGCAGGAACGGGTTGGTACAGCTCTTTCGCAGAGTGGGCGTCACCGAACTCGAAGCCCGCTGCGGAACGCTCCCCCCAGCCTCGCAGCGTTGGGACCGTATCCTCCAGGCATCAGGGATGAAAAGGGCCAAACCGTCCCCTACTTCAGCTCAAACGCCGGATCAGGCGTCTTTGCATGCATTCGCCGATTCGCTGGAGCGTGACCTTGATGCGCCCAGCCCAATGCACGAGGGAGATCAGACGCGGGCAAGCAGCCGTAAACGGTCCCGATCGGATCGTGCTGTCACCGGCCCCTCCGCACAGCAATCTTTCGAGGTGCGCGTTCCCGAACAGCGCGATGCGCTGCATTTGCCCCTCAGCTGGAGGGTAAAACGCCCGCGTACCAGGATCGGGGGCGGCCTCCCGGATCC

>***tal*-*Bam*HI-11 (*tal_Xoo_*_K74_-14)**

GGATCCCATTCGTTCGCGCACGCCAAGTCCTGCCCGCGAGCTTCTGCCCGGACCCCAACCGGATAGGGTTCAGCCGACTGCAGATCGGGGGGGGGCTCCGCCTGCTGGCGGCCCCCTGGATGGCTTGCCCGCTCGGCGGACGATGTCCCGGACCCGGCTGCCATCTCCCCCTGCGCCCTCGCCTGCGTTCTCGGCGGGCAGCTTCAGCGATCTGCTCCGTCAGTTCGATCCGTCGCTTCTTGATACATCGCTTCTTGATTCGATGCCTGCCGTCGGCACGCCGCATACAGCGGCTGCCCCAGCAGAGTGGGATGAGGTGCAATCGGGTCTGCGTGCAGCCGATGACCCGCCACCCACCGTGCGTGTCGCTGTCACTGCCGCGCGGCCGCCGCGCGCCAAGCCGGCCCCGCGACGGCGTGCGGCGCAACCCTCCGACGCTTCGCCGGCCGCGCAGGTGGATCTACGCACGCTCGGCTACAGTCAGCAGCAGCAAGAGAAGATCAAACCGAAGGTGCGTTCGACAGTGGCGCAGCACCACGAGGCACTGGTGGGCCATGGGTTTACACACGCGCACATCGTTGCGCTCAGCCAACACCCGGCAGCGTTAGGGACCGTCGCTGTCAAGTATCAGCACATAATCACGGCGTTGCCAGAGGCGACACACGAAGACATCGTTGGCGTCGGCAAACAGTGGTCCGGCGCACGCGCCCTGGAGGCCTTGCTCACGAAGGCGGGGGAGTTGAGAGGTCCGCCGTTACAGTTGGACACAGGCCAACTTCTCAAGATTGCAAAACGTGGCGGCGTGACCGCAGTGGAGGCAGTGCATGCATGGCGCAATGCACTGACGGGTGCCCCCCTGAACCTGACCCCGGACCAAGTGGTGGCCATCGCCAGCAATATTGGCGGCAAGCAGGCGCTGGAGACGGTGCAGCGCCTGTTGCCGGTGCTGTGCCAGGCCCATGGCCTGAACCCGGACCAAGTGGTGGCCATCGCCAGCAATAGTGGCGGCAAGCAGGCGCTGGAGACGGTGCAGCGGCTGTTGCCGGTGCTGTGCCAGGACCATGGCCTGACCCCGGACCAAGTGGTGGCCATCGCCAGCCACGATGGCGGCAAGCAGGCGCTGGAGACGGTGCAGCGGCTGTTGCCGGTGCTGTGCCAGGACCATGGCCTGACCCCAGACCAGGTCGTGGCCATCGCCAGCAATGGCGGCGGCAAGCAGGCGCTGGAGACGGTGCAGCGGCTGTTGCCGGTGCTGTGCCAGGCCCATGGCCTGAACCCGGACCAGGTGGTGGCCATCGCCAGCAATAGTGGCGGCAAGCAGGCGCTGGAGACGGTGCAGCGGCTGTTGCCGGTGCTGTGCCAGGCCCATGGCCTGAACCCGGACCAAGTGGTGGCCATCGCCAGCAATAACGGCGGCAAGCAGGCGCTGGAGACGGTGCAGCGGCTGTTGCCGGTGCTGTGCCAGGACCATGGCCTGAGTCCGGACCAGGTGGTGGCCATCGCCAGCCACGATGGCGGCAAGCAGGCGCTGGAGACGGTGCAGCGGCTGTTGCCGGTGCTGTGCCAGGACCATGGCCTGACCCCGGACCAGGTCGTGGCCATCGCCAGCAATGGCGGCAAGCAGGCGCTGGAGACGGTGCAGCGCCTGTTGCCGGTGCTGTGCCAGGACCATGGCCTGACCCCGGACCAGGTGGTGGCCATCGCCAACAATAACGGCGGCAAGCAGGCGCTGGAGACGGTGCAACGGCTGTTGCCGGTGCTGTGCCAGGACCATGGCCTGACCCCGGACCAGGTGGTGGCCATCGCCAACAATAACGGCGGCAAGCAGGCGCTGGAGACGGTGCAGCGGCTGTTGCCGGTGCTGTGCCAGGCCCATGGCCTGCCCCCGGACCAGGTGGTGGCCATCGCCAGCAATATTGGCGGCAAGCAGGCGCTGGAGACGGTGCAGCGGCTGTTGCCGGTGCTGTGCCAGGACCATGGCCTGACCCCGGACCAAGTGGTGGCCATCGCCAACAATAACGGCGGCAAGCAGGCGCTGGAGACGGTGCAGCGGCTGTTGCCGGTGCTGTGCCAGGACCATGGCCTGACCCCGGACCAGGTGGTGGCCATCGCCAGCCACGATGGCGGCAAGCAGGCGCTGGAGACGGTGCAGCGGCTGTTGCCGGTGCTGTGCCAGGACCATGGCCTGACCCCGGACCAGGTGGTGGCCATCGCCAGCCATGGCGGCGGCAAGCAGGCGCTGGAGACGGTGCAGCGGCTGTTGCCGGTGCTGTGCCAGGACCATGGCCTGAGTCCGGACCAGGTGGTGGCCATCGCCAGCCACGATGGCGGCAAGCAGGCGCTGGAGACGGTGCAGCGGCTGTTGCCGGTGCTGTGCCAGGACCATGGCCTGACCCTGGACCAGGTGGTGGCCATCGCCAGCCACGATGGCGGCAAGCAGGCGCTGGAGACGGTGCAACGGCTGTTGCCGGTGCTGTGCCAGGACCATGGCCTGACCCCGGCCCAGGTGGTGGCCATCGCCAACAATAACGGCGGCAAGCAGGCGCTGGAGACGGTGCAGCGGCTGTTGCCGGTGCTGTGCCAGGACCATGGCCTGACCCCGGACCAGGTGGTGGCCATCGCCAGCAATGGCGGCGGCAAGCAGGCGCTGGAGAGCATTGTTGCCCAGTTATATCGCCCTGATCCGGCGTTGGCCGCGTTGACCAACGACCACCTCGTCGCCTTGGCCTGCCTCGGCGGACGTCCTGCCCTGGATGCAGTGAAAAAGGGATTGCCGCACGCGCCGGAATTGATCAGAAGAATCAATCGCCGTATTCCCGAACGCACGTCCCATCGCGTTGCCGACCTCGCGCACGTGGTGCGCGTGCTTGGTTTTTTCCAGAGCCACTCCCACCCAGCGCAAGCATTCGATGACGCCATGACGCAGTTCGGGATGAGCAGGCACGGGTTGGTACAGCTCTTTCGCAGAGTGGGCGTCACCGAATTCGAAGCCCGCTGCGGAACGCTCCCCCCAGCCTCGCAGCGTTGGGACCGTATCCTCCAGGCATCAGGGATGAAAAGGGCCAAACCGTCCCCTACTTCAGCTCAAACACCGGATCAGGCGTCTTTGCATGCATTCGCCGATTCGCTGGAGCGTGACCTTGATGCGCCCGGCCCAATGCACGAGGGAGATCAGACGCGGGCAAGCAGCCGTAAACGGTCCCGATCGGATCGTGCTGTCACCGGCCCCTCCGCACAGCAATCTTTCGAGGTACGCGTTCCCGAACAGCGCGATGCTCTGCATTTGCCCCTCAGCTGGAGGGTAAAACGCCCGCGTACCAGGATCGGGGGCGGCCTCCCGGATCC

>***tal*-*Bam*HI-14 (*tal_Xoo_*_K74_-18)**

GGATCCCATTCGTTCGCGCACGCCAAGTCCTGCCCGCGAGCTTCTGCCCGGACCCCAACCGGATAGGGTTCAGCCGACTGCAGATCGGGGGGGGGCTCCGCCTGCTGGCGGCCCCCTGGATGGCTTGCCCGCTCGGCGGACGATGTCCCGGACCCGGCTGCCATCTCCCCCTGCGCCCTCGCCTGCGTTCTCGGCGGGCAGCTTCAGCGATCTGCTCCGTCAGTTCGATCCGTCGCTTCTTGATACATCGCTTCTTGATTCGATGCCTGCCGTCGGCACGCCGCATACAGCGGCTGCCCCAGCAGAATGGGATGAGGCGCAATCGGGTCTGCGTGCAGCCGATGACCCGCCACCCACCGTGCCTGTCGCTGTCACTGCCGCGCGGCCGCCGCGCGCCAAGCCGGCCCCGCGACGGCGTGCGGCGCAACCCTCCGACGCTTCGCCGGCCGCGCAGGTGGATCTACGCACGCTCGGCTACAGTCAGCAGCAGCAAGAGAAGATCAAACCGAAGGTGCGTTCGACAGTGGCGCAGCACCACGAGGCACTGGTGGGCCATGGGTTTACACACGCGCACATCGTTGCGCTCAGCCAACACCCGGCAGCGTTAGGGACCGTTGCTGTCACGTATCAGGACATAATCAGGGCGTTGCCAGAGGCGACACACGAAGACATCGTTGGCGTCGGCAAACAGTGGTCCGGCGCACGCGCCCTGGAGGCCTTGCTCACGAAGGCGGGGGAGTTGAGAGGTCCACCGTTACAGTTGGACACAGGCCAACTTGTCAAGATTGCAAAACGTGGCGGCGTGACCGCAGTGGAGGCAGTGCATGCATCGCGCAATGCACTGACGGGTGCCCCCCTGAACCTGACCCCGGACCAAGTGGTGGCCATCGCCAGCAATATTGGCGGCAAGCAGGCGCTGGAGACGGTGCAGCGCCTGTTGCCGGTGCTGTGCCAGGACCATGGCCTGACCCCGGACCAGGTGGTGGCCATCGCCAACAATAACGGCGGCAAGCAGGCGCTGGAGACGGTGCAGCGGCTGTTGCCGGTGCTGTGCCAGGACCATGGCCTGACCCCGGACCAGGTGGTGGCCATCGCCAGCAATATTGGCGGCAAGCAGGCGCTGGAGACGGTGCAGCGGCTGTTGCCGGTGCTGTGCCAGGACCATGGCCTGACCCCGGACCAGGTGGTGGCCATCGCCAGCCATGGCGGCGGCAAGCAGGCGCTGGAGACGGTGCAGCGGCTGTTGCCGGTGCTGTGCCAGGACCATGGCCTGACCCCGGACCAGGTGGTGGCCATCGCCAGCCATGGCGGCGGCAAGCAGGCGCTGGAGACGCTGCAGCGGCTGTTGCCGGTGCTGTGCCAGGACCATGGCCTGACCCCGGACCAGGTCGTGGCCATCGCCAGCCACGATGGCGGCAAGCAGGCGCTGGAGACGGTGCAGCGGCTGTTGCCGGTGCTGTGCCAGGACCATGGCCTGACCCCGGACCAGGTCGTGGCCATCGCCAGCAATGGCGGCGGCAAGCAGGCGCTGGAGACGCTGCAACGGCTGTTGCCGGTGCTGTGCCAGGACCATGGCCTGACCCCGGACCAGGTCGTGGCCATCGCCAGCCACGATGGCGGCAAGCAGGCGCTGGAGACGGTGCAGCGGCTGTTGCCGATGCTGTGCCAGGACCATGGCCTGACCCCGGACCAGGTGGTGGCCATCGCCAGCCATGGCGGCGGCAAGCAGGCGCTGGAGACAGTGCAGCGGCTGTTGCCGGTGCTGTGCCAGGACCATGGCCTGACCCCGGACCAGGTGGTGGCCATCGCCAGCCACGATGGCGGCAAGCAGGCGCTGGAGACGGTGCAGCGGCTGTTGCCGGTGCTGTGCCAGGACCATGGCCTGACCCTGGACCAGGTGGTGGCCATCGCCAGCCACGATGGCGGCAAGAAGGCGCTGGAGACGGTGCAACGGCTGTTGCCGGTGCTGTGCCAGGACCATGGCCTGACCCCGGACCAGGTCGTGGCCATCGCCAGCCACGATGGCGGCAAGCAGGCGCTGGAGACGGTGCAGCGGCTGTTGCCGGTGCTGTGCCAGGACCATGGCCTGACCCCGGACCAGGTCGTGGCCATCGCCAGCAATGGCGGCGGCAAGCAGGCGCTGGAGAGCATTGTTGCCCAGTTATCTCGCCCTGATCCGGCGTTGGCCGCGTTGACCAACGACCACCTCGTCGCCTTGGCCTGCCTCGGCGGACGTCCTGCCCTGGATGCAGTGAAAAAGGGATTGCCGCACGCGCCGGAATTGATCAGAAGAGTCAATCGCCGTATTGGCGAACGCACGTCCCATCGCGTTGCCGACTACGCGCAAGTGGTTCGCGTGCTGGAGTTTTTCCAGTGCCACTCCCACCCAGCGCAAGCATTCGATGACGCCATGACGCAGTTCGGGATGAGCAGGCACGGGTTGGTACAGCTCTTTCGCAGAGTGGGCGTCACCGAATTCGAAGCCCGCTGCGGAACGCTCCCCCCAGCCTCGCAGCGTTGGGACCGTATCCTCCAGGCATCAGGGATGAAAAGGGCCAAACCGTCCCCTACTTCAGCTCAAACACCGGATCAGGCGTCTTTGCATGCATTCGCCGATTCGCTGGAGCGTGACCTTGATGCGCCCAGCCCAATGCACGAGGGAGATCAGACGCGGGCAAGCAGCCGTAAACGGTCCCGATCGGATCGTGCTGTCACCGGCCCCTCCACACAGCAATCTTTCGAGGTGCGCGTTCCCGAACAGCGCGATGCGCTGCATTTGCCCCTCAGCTGGAGGGTAAAACGCCCGCGTACCAGGATCGGGGGCGGCCTCCCGGATCC

>***tal*-*Bam*HI-16 (*tal_Xoo_*_K74_-13)**

GGATCCCATTCGTTCGCGCACGCCAAGTCCTGCCCGCGAGCTTCTGCCCGGACCCCAACCGGATAGGGTTCAGCCGACTGCAGATCGGGGGGGGACTCCGCCTGCTGGCGGCCCCCTGGATGGCTTGCCCGTTCGGCGGACGATGTCCCGGACCCGGCTGCCATCTCCCCCTGCACCCTCGCCTGCGTTCTCGGCGGGCAGCTTCAGCGATCTGCTCCGTCAGTTCGATCCGTCGCTTCTTGATACATCGCTTCTTGATTCGATGCCTGCCGTCGGCACGCCGCATACAGCGGCTGCCCCAGCAGAGTGGGATGAGGTGCAATCGGGTCTGCGTGCAGCCGATGACCCGCCACCCACTGTGCGTGTCGCTGTCACTGCCGCGCGGCCGCCGCGCGCCAAGCCGGCCCCGCGACGGCGTGCGGCGCAACCCTCCGACGCTTCGCCGGCCGCGCAGGTGGATCTACGCACGCTCGGCTACAGTCAGCAGCAGCAAGAGAAGATCAAACCGAAGGTGCGTTCGACAGTGGCGCAGCACCACGAGGCACTGGTGGGCCATGGGTTTACACACGCGCACATCGTTGCGCTCAGCCAACACCCGGCAGCGTTAGGGACCGTTGCTGTCACGTATCAGGACATAATCAGGGCGTTGCCAGAGGCGACACACGAAGACATCGTTGGCGTCGGCAAACAGTGGTCCGGCGCACGCGCCCTGGAGGCCTTGCTCACGGAGGCGAGGGAGTTGAGAGGTCCGCCGTTACAGTTGGACACAGGCCAACTTCTCAAGATTGCAAAACGTGGCGGCGTGACCGCAGTGGAGGCAGTGCATGCATGGCGCAATGCACTGACGGGTGCCCCCCTGAACCTGACCCCGGACCAAGTGGTGGCCATCGCCAGCAATATTGGCGGCAACCAGGCGCTGGAGACGGTACAGCGGCTGTTGCCGGTGCTGTGCCAGGCCCATGGCCTGACCCCGGACCAGGTCGTGGCCATCGCCAGCAATGGCGGCAAGCAGGCGCTGGAGACGGTGCAGCGGCTGTTGCCGGTGCTGTGCCAGGACCATAGCCTGACCCCGGACCAGGTCGTGGCCATCGCCAGCAATATAGGCGGCAAGCAGGCGCTGGAGACGGTGCAGCGGCTGTTGCCGGTGCTGTGCCAGGACCATGGCCTGACCCCGGACCAGGTGGTGGCCATCGCCAGCAATAGTGGCGGCAAGCAGGCGCTGGAGACGGTGCAGCGGCTGTTGCCGGTGCTGTGCCAGGACCATGGCCTGACCCCGGACCAGGTGATGACCATCGCCAGCAATAACGGCGGCAAGCAGGCGCTGGAGACGGTGCAACGGCTGTTGCCGGTGCTGTGCCAGGACCATGGCCTGACCCCGGACCAGGTGGTGACCATCGCCAGCAATGGCGGCGGCAAGCAGGCGCTGGAGACGGTGCAGCGGCTGTTGCCGGTGCTGTGCCAGGCCCATGGCCTGACCCCGGACCAAGTGGTGGCCATCGCCAACAATAACGGCGGCAAGCAGGCGCTGGAGACGGTGCAACGGCTGTTGCCGGTGCTATGCCAGGCCCATGGCCTGACCCCGGCCCAGGTCGTGGCCATCGCCAGCAATAGCGGCGGCAAGCAGGCGCTGGAGACGGTGCAGCGGCTGTTGCCGGTGCTGTGCCAGGCCCATGGCCTGACCCCGGACCAGGTGGTGGCCATCGCCAGCAATGGCGGCAAGCAGGCGCTGGAGACGGTGCAGCGGCTGTTGCCGGTGCTGTGCCAGGCCCATGGCCTGACCCCGGACCAGGTGGTGGCCATCGCCAGCAATAGTGGCGGCAAGCAGGCGCTGGAGACGGTGCAGCGGCTGTTGCCGGTGCTGTGCCAGGCCCATGGCCTGACCCCGGACCAGGTGGTGGCCATCGCCAACAATAACGGCGGCAAGCAGGCGCTGGAGACGGTGCAGCGGCTGTTGCCGGTGCTGTGCCAGGCCCATGGCCTGACCCCGGACCAGGTGGTGGCCATCGCCAGCAATAGTGGCGGCAAGCAGGCGCTGGAGACGGTGCAGCGGCTGTTGCCGGTGCTGTGCCAGGACCATGGCCTGACCCCGGACCAGGTGGTGGCCATCGCCAGCAATGGCGGCAAGCAGGCGCTGGAGACGGTGCAGCGGCTGTTGCCGGTGCTGTGCCAGGACCATGGCCTGACCCCGGACCAGGTGGTGGCCATCGCCAGCCACGATGGCGGCAAGCAGGCGCTGGAGACGGTGCAGCGGCTGTTGCCGGTGCTGTGCCAGGACCATGGCCTGACCCTGGACCAGGTGGTGGCCATCGCCAGCCATGGCGGCGGCAAGCAGGCGCTGGAGACGGTGCAGCGGCTGTTGCCGGTGCTGTGCCAGGACCATGGCCTGACCTCGGACCAGGTGGTGGCCATCGCCAGCCACGATGGCGGCAAGCAGGCGCTGGAGACGGTGCAGCGGCTGTTGCCGGTGCTGTGCCAGGACCATGGCCTGACCCCGGACCAGGTGGTGGCCATTGCCAGCAATATTGGCGGCAAGCAGGCGCTGGAGACGGTGCAGCGGCTGTTGCCGGTGCTGTGCCAGGACCATGGCCTGACCCTGGACCAGGTGGTGGCCATCGCCAGCCACGATGGCGGCAAGCAGGCGCTGGAGACGGTGCAACGGCTGTTGCCGGTGCTGTGCCAGGACCATGGCCTGACCCCGGACCAGGTCGTGGCCATCGCCAGCCACGATGGCGGCAAGCAGGCGCTGGAGACGGTGCAGCGGCTGTTGCCGGTGCTGTGCCAGGACCATGGCCTGACCCCGGCCCAGGTGGTGGCCATCGCCAGCAATGGCGGCGGCAAGCAGGCGCTGGAGAGCATTGTTGCCCAGTTATCTCGCCCTGATCCGGCGTTGGCCGCGTTGACCAACGACCACCTCGTCGCCTTGGCCTGCCTCGGCGGACGTCCTGCCCTGGATGCAGTGAAAAAGGGATTGCCGCACGCGCCGGAATTGATCAGAAGAATCAATCGCCGTATTCCCGAACGCACGTCCCATCGCGTTGCCGACTACGCGCAAGTGGTTCGCGTGCTGGAGTTTTTCCAGTGCCACTCCCACCCAGCGTACGCATTTGATGAGGCCATGACGCAGTTCGGGATGAGCAGGCACGGGTTGGTACAGCTCTTTCGCAGAGTGGGCGTCACCGAATTCGAAGCCCGCTGCGGAACGCTCCCCCCAGCCTCGCAGCGTTGGGACCGTATCCTCCAGGCATCAGGGATGAAAAGGGCCAAACCGTCCCCTACTTCAGCTCAAACACCGGATCAGGCGTCTTTGCATGCATTCGCCGATTCGCTGGAGCGTGACCTTGATGCGCCCAGCCCAATGCACGAGGGAGATCAGACGCGGGCAAGCAGCCGTAAACGGTCCCGATCGGATCGTGCTGTCACCGGCCCCTCCACACAGCAATCTTTCGAGGTGCGCGTTCCCGAACAGCGCGATGCGCTGCATTTGCCCCTCAGCTGGAGGGTAAAACGCCCGCGTACCAGGATCGGGGGCGGCCTCCCGGATCC

>***tal*-*Bam*HI-21 (*tal_Xoo_*_K74_-11)**

GGATCCCATTCGTTCGCGCACGCCAAGTCCTGCCCGCGAGCTTCTGCCCGGACCCCAACCGGATAGGGTTCAGCCGACTGCAGATCGGGGGGGGGCTCCGCCTGCTGGCGGCCCCCTGGATGGCTTGCCCGCTCGGCGGACGATGTCCCGGACCCGGCTGCCATCTCCCCCTGCGCCCTCGCCTGCGTTCTCGGCGGGCAGCTTCAACGATCTGCTCCGTCAGTTCGATCCGTCGCTTCTTGATACATCGCTTCTTGATTCGATGCCTGCCGTCGGCACGCCGCATACAGCGGCTGCCCCAGCAGAGTGGGATGAGGTGCAATCGGGTCTGCGTGCAGCCGATGACCCGCCACCCACCGTGCGTGTCGCTGTCACTGCCGCGCGGCCGCCGCGCGCCAAGCCGGCCCCGCGACGGCGTGCGGCGCAACCCTCCGACGCTTCGCCGGCCGCGCAGGTGGATCTACGCACGCTCGGCTACAGTCAGCAGCAGCAAGAGAAGATCAAACCGAAGGTGCGTTCGACAGTGGCGCAGCACCACGAGGCACTGGTGGGCCATGGGTTTACACACGCGCACATCGTTGCGCTCAGCCAACACCCGGCAGCGTTAGGGACCGTCGCTGTCAAGTATCAGCACATAATCACGGCGTTGCCAGAGGCGACACACGAAGACATCGTTGGCGTCGGCAAACAGTGGTCCGGCGCACGCGCCCTGGAGGCCTTGCTCACGGAGGCGAGGGAGTTGAGAGGTCCGCCGTTACAGTTGGACACAGGCCAACTTCTCAAGATTGCAAAACGTGGCGGCGTGACCGCAGTGGAGGCAGTGCATGCATGGCGCAATGCACTGACGGGTGCCCCCCTGAACCTGACCCCGGACCAAGTGGTGGCCATCGCCAGCAATATTGGCGGCAAGCAGGCGCTGGAGACGGTGCAGCGGCTGTTGCCGGTGCTGTGCCAGGACCATGGCCTGACCCCGGACCAGGTCGTGGCCATCGCCAGCAATGGCGGCGGCAAGCAGGCGCTGGAGACGGTGCAGCGGCTGTTGCCGGTGCTGTGCCAGGACCATGGCCTGACCCCGGACCAGGTCGTGGCCATCGCCAGCAATAACGGCGGCAAGCAGGCGCTGGAGACGGTGCAGCGGCTGTTGCCGGTGCTGTGCCAGGACCATGGCCTGACCCCGGACCAGGTCGTGGCCATCGCCAGCAATGGCGGCGGCAAGCAGGCGCTGGAGACGGTGCAGCGGCTGTTGCCGGTGCTGTGCCAGGACCATGGCCTGACCCCGGACCAAGTGGTGGCCATCGCCAACAATAAAGGCGGCAAGCAGGCGCTGGAGACGCTGCAGCGGCTGTTGCCGGTGCTGTGCCAGGCCCATGGCCTGACCCCGGACCAGGTGGTGGCCATCGCCAGCAATGGCGGCGGCAAGCAGGCGCTGGAGACGGTGCAACGGCTGTTGCCGGTGCTGTGCCAGGATCATGGCCTGACCCCGGCCCAGGTCGTGGCCATCGCCAGCAATATTGGCGGCAAGCAGGCGCTGGAGACGGTGCAGCGGCTGTTGCCGGTGCTGTGCCAGGACCATGGCCTGACCCCGGACCAAGTGGTGGCCATCGCCAACAATAACGGCGGCAAGCAGGCGCTGGAGACGGTGCAGCGGCTGTTGCCGGTGCTGTGCCAGGACCATGGCCTGACCCCGGACCAGGTCGTGACCATCGCCAGCAATATTGGCGGCAAGCAGGCGCTGGAGACGGTGCAGCGGCTGTTGCCGGTGCTGTGCCAGGACCATGGCCTGACCCCGGACCAAGTGGTGGCCATCGCCAACAATAACGGCGGCAAGCAGGCGCTGGAGACGGTGCAGCGGCTGTTGCCGGTGCTGTGCCAGGCCCATGGCCTGACCCCGGACCAGGTCGTGGCCATCGCCAGCAATATTGGCGGCAAGCAGACGCTGGAGACGGTGCAGCGGCTGTTGCCGGTGCTGTGCCAGGACCATGGCCTAACCCCGGACCAGGTCGTGGCCATCGCCAACAATAACGGCGGCAAGCAGGCGCTGGAGACGGTGCAGCGGCTGTTGCCGGTGCTGTGCCAGGACCATGGCCTGACCCCGGACCAGGTCGTGGCCATCGCCAGCAATAGTGGCGGCAAGCAGGCGCTGGAGACGGTGCAGCGGCTGTTGCCGGTGCTGTGCCAGGACCATGGCCTGACCCCGAACCAGGTGGTGGCCATCGCCAGCAATGGCGGCGGCAAGCAGGCGCTGGAGACGGTGCAGCGGCTGTTGCCGGTGCTGTGCCAGGACCATGGCCTGACCCCGGACCAGGTCGTGGGCATCGCCAGCAATAGTGGCGGCAAGCAGGCGCTGGAGACGGTGCAGCGGCTGTTGCCGGTGCTGTGCCAGGACCATGGCCTGACCCCGGACCAGGTCGTGGCCATCGCCAACAATAACGGTGGCAAGCAGGCGCTGGAGACGGTGCAGCGGCTGTTGCCGGTGCTGTGCCAGGACCATGGCCTGACCCCGGACCAGGTGGTGGCCATCGCCAGCAATATTGGCGGCAAGCAGGCGCTGGAGACGGTGCAGCGGCTGTTGCCGGTGCTGTGCCAGGACCATGGCCTGACCCTGGACAAGGTGGTGGCCATCGCCAGCAATGGCGGCAAGCAGGCGCTGGAGACGGTGCAGCGGCTGTTGCCGGTGCTGTGCCAGGACCATGGCCTGACCCCGGACCAGGTCGTGGCCATCGCCAGCAATAGTGGCGGCAAGCAGGCGCTGGAGACGGTGCAGCGGCTGTTGCCGGTGCTGTGCCAGGACCATGGCCTGACCCCGGACCAGGTCGTGGCCATCGCCAGCAATGGCGGCGGCAAGCAGGCGCTGGAGAGCATTGTTGCCCAGTTATCTCGCCCTGATCCGGCGTTGGCCGCGTTGACCAACGACCACCTCGTCGCCTTGGCCTGCCTCGGCGGACGTCCTGCCATGGATGCAGTGAAAAAGGGATTGCCGCACGCGCCGGAATTGATCAGAAGAGTCAATCGCCGTATTGGCGAACGCACGTCCCATCGCGTTGCCGACTACGCGCAAGTGGTTCGCGTGCTGGAGTTTTTCCAGTGCCACTCCCACCCAGCGTACGCATTTGATGAGGCCATGACGCAGTTCGGGATGAGCAGGAACGGGTTGGTACAGCTCTTTCGCAGAGTGGGCGTCACCGAACTCGAAGCCCGCGGTGGAACGCTCCCCCCAGCCTCGCAGCGTTGGGACCGTATCCTCCAGGCATCAGGGATGAAAAGGGCCAAACCGTCCCCTACTTCAGCTCAAACACCGGATCAGGCGTCTTTGCATGCATTCGCCGATTCGCTGGAGCGTGACCTTGATGCGCCTAGCCCAATGCACGAGGGAGATCAGACAGGGGCAAGCAGCCGTAAACGGTCCCGATCGGATCGTGCTGTCACCGGCCCCTCCGCACAGCAATCTTTCGAGGTGCGCGTTCCCGAACAGCGCGATGCGCTGCATTTGCCCCTCAGCTGGAGGGTAAAACGCCCGCGTACCAGGATCGGGGGCGGCCTCCCGGATCC

>***tal*-*Bam*HI-22 (*tal_Xoo_*_K74_-4)**

GGATCCCATTCGTTCGCGCACGCCAAGTCCTGCCCGCGAGCTTCTGCCCGGACCCCAACCGGATAGGGTTCAGCCGACTGCAGATCGGGGGGGGGCTCCGCCTGCTGGCGGCCCCCTGGATGGCTTGCCCGCTCGGCGGACGATGTCCCGGACCCGGCTGCCATCTCCCCCTGCGCCCTCGCCTGCGTTCTCGGCGGGCAGCTTCAGCGATCTGCTCCGTCAGTTCGATCCGTCGCTTCTTGATACATCGCTTCTTGATTCGATGCCTGCCGTCGGCACGCCGCATACAGCGGCTGCCCCAGCAGAATGGGATGAGGCGCAATCGGGTCTGCGTGCAGCCGATGACCCGCCACCCACCGTGCCTGTCGCTGTCACTGCCGCGCGGCCGCCGCGCGCCAAGCCGGCCCCGCGACGGCGTGCGGCGCAACCCTCCGACGCTTCGCCGGCCGCGCAGGTGGATCTACGCACGCTCGGCTACAGTCAGCAGCAGCAAGAGAAGATCAAACCGAAGGTGCGTTCGACAGTGGCGCAGCACCACGAGGCACTGGTGGGCCATGGGTTTACACACGCGCACATCGTTGCGCTCAGCCAACACCCGGCAGCGTTAGGGACCGTTGCTGTCACGTATCAGGACATAATCAGGGCGTTGCCAGAGGCGACACACGAAGACATCGTTGGCGTCGGCAAACAGTGGTCCGGCGCACGCGCCCTGGAGGCCTTGCTCACGAAGGCGGGGGAGTTGAGAGGTCCGCCGTTACAGTTGGACACAGGCCAACTTCTCAAGATTGCAAAACGTGGCGGCGTGACCGCAGTGGAGGCAGTGCATGCATCGCGCAATGCACTGACGGGTGCCCCCCTGAACCTGACCCCGGACCAAGTGGTGGCCATCGCCAGCCACGATGGCGGCAAGCAGGCACTGGAGACGGTGCAGCGGCTGTTGCCGGTGCTGTGCCAGGCCCATGGCTTGACCCCGGCGCAGGTGGTGGCCATCGCCAGCCACGATGGCGGCAAGCAGGCGCTGGAGACGGTGCAGCGGCTGTTGCCGGTGCTGTGCCAGGCCCATGGTCTGACCCCAGCGCAGGTGGTGGCCATCGCCAGCAATAACGGCGGCAAGCAGGCGCTGGAGACGGTGCAGCGGCTGTTGCCGGTGCTGTGCCAGGCCCATGGCCTGACCCCGGACCAGGTCGTGGCCATCGCCAGCAATAACGGCGGCAAGCAGGCGCTGGAGACGGTGCAGCGGCTGTTGCCGGTGCTGTGCCAGGCCCATGGCCTGACCCCAGACCAGGTCGTGGCCATCGCCAGCAATATTGGCGGCAAGCAGGCGCTGGAGACGGTGCAGCGGCTGTTGCCGGTGCTGTGCCAGGACCATGGCCTGACCCCGGACCAGGTCGTGGCCATCGCCAGCAATGGCGGCGGCAAGCAGGCGCTGGAGACGGTGCAGCGGCTGTTGCCGGTGCTGTGCCAGGCCCATGGCCTGCCCCCGGCGCAGGTGGTGGCCATCGCCAGCCACGATGGCGGCAAGCAGGCGCTGGAGACGGTGCAGCGGCTGTTGCCGGTGCTGTGCCAGGACCATGGCCTGACCCCGGCGCAGGTGGTGGCCATCGCCAGCAGTGGCGGCAAGCAGGCGCTGGAGACGGTGCAGCGGCTGTTGCCGGTGCTGTGCCAGGCCCATGGCCTGACCCTGGACCAGGTGGTGGCCATCGCCAGCCATGGCGGCAGCAAGCAGGCGCTGGAGACGGTGCAGCGGCTGTTGCCGGTGCTGTGCCAGGACCATGGCCTGACCCCGGACCAGGTGGTGGCCATCGCCAGCCACGATGGCGGCAAGCAGGCGCTGGAGACGGTGCAGCGGCTGTTGCCGGTGCTGTGCCAGGCCCATGGCCTGACCCCGGACCAGGTCGTGGCCATCGCCAGCAATGGCGGCGGCAAGCAGGCGCTGGAGACGGTGCAACGGCTGTTGCCGGTGCTGTGCCAGGACCATGGCCTGACCCCGGACCAGGTGGTGGCCATCGCCAGCAATGGCGGCAAGCAGGCGCTGGAGACGGTGCAGCGGCTGTTGCCGGTGCTGTGCCAGGACCATGGCCTGACCCCGGACCAGGTCGTGGCCATCGCCAGCAATGGCGGCGGCAAGCAGGCGCTGGCGACGGTGCAGCGGCTGTTGCCGGTGCTGTGCCAGGCCCATGGCCTGACCCCGGACCAGGTCGTGGCCATCGCCAGCCACGATGGCGGCAAGCAGGCGCTGGAGACGGTGCAGCGGCTGTTGCCGGTGCTGTGCCAGGACCATGGCCTGACCCCGGACCAGGTGGTGGCCATCGCCAGCCACGATGGCGGCAAGCAGGCGCTGGAGACGGTGCAGCGGCTGTTGCCGGTGCTGTGCCAGGCCCATGGCCTGACCCCGGACCAGGTGGTGGCCATCGCCAGCAATGGCGGCAAGCAGGCGCTGGAGACGGTGCAGCGGCTGTTGCCGGTGCTGTGCCAGGCCCATGGCCTGACCCTGGCGCAGGTGGTGGCCATCGCCAGCAATATTGGCGGCAAGCAGGCGCTGGAGACGGTGCAGCGGCTGTTGCCGGTGCTGTGCCAGGACCATGGCCTGACCCCGGACCAGGTGGTGGCCATCGCCAGTAATATTGGCGGCAAGCAGGCGCTGGAGACGGTGCAGCGGCTGTTGCCGGTGCTGTGCCAGGACCATGGCCTGACCCCGGACCAGGTGGTGGCCATCGCCAGCAATAACGGCGGCAAGCAGGCGCTGGAGACGGTGCAGCGGCTGTTGCCGGTGCTGTGCCAGGACCATGGCCTGACCCTGGATCAGGTCGTGGCCATCGCCAGCCACGATGGCGGCAAGCAGGCGCTGGAGACGGTGCAGCGGCTGTTGCCGGTGCTGTGCCAGGCCCATGGCCTGACCCCGGCGCAGGTGGTGGCCATCGCCAGCCATATTGGCGGCAAGCAGGCGCTGGAGACGGTGCAGCGGCTGTTGCCGGTGCTGTGCCAGGACCATGGCCTGACCCTGGACCAGGTGGTGGCCATTGCCAGCAATGACGGCAGCAAGCAGGCGCTGGAGACGGTGCAGCGGCTGTTGCCGGTGCTGTGCCAGGACCATGGCCTGACCCCGGACCAGGTGGTGGCCATCGCCAGCCACGATGGCGGCAAGCAGGCGCTGGAGACGGTGCAGCGGCTGTTGCCGGTGCTGTGCCAGGACCATGGCCTGACCCCGGACCAGGTGGTGGCCATCGCCAGCAATGGCGGCGGCAAGCAGGCGCTGGAGACGGTGCAACGGCTGTTGCCGGTGCTGTGCCAGGACCATGGCCTGACCCCGGCCCAGGTCGTGGCCATCGCCAACAATAACGGCGGCAAGCAGGCGCTGGAGACGGTGCAGCGGCTGTTGCCGGTGCTGTGCCAGGACCATGGCCTGACCCCGGACCAGGTCGTGGCCATCGCCAGCCATGGCGGCGGCAAGCAGGCGCTGGAGACGGTGCAGCGGCTGTTGCCGGTGCTGTGCCAGGACCATGGCCTGACCCCGGACCAGGTGGTGGCCATCGCCAGCAATGGCGGCAAGCAGGCGCTGGAGAGCATTGTTGCCCAGTTATCTCGCCCTGATCCGGAGTTGGCCGCGTTGACCAACGACCACCTCGTCGCCTTGGCCTGCCTCGGCGGACGTCCTGCCCTGGATGCAGTGAAAAAGGGATTGCCGCACGCGCCGGAATTGATCAGAAGAATCAATCGCCGCATTCCCGAACGCACGTCCCATCGCGTTCCCGACCTCGCGCACGTGGTGCGCGTGCTTGGTTTTTTCCAGAGCCACTCCCACCCAGCGCAAGCATTCGATGACGCCATGACGCAGTTCGGGATGAGCAGGAACGGGTTGGTACAGCTCTTTCGCAGAGTGGGCGTCACCGAACTCGAAGCCCGCGGTGGAACGCTCCCCCCAGCCTCGCAGCGTTGGGACCGTATCCTCCAGGCATCAGGGATGAAAAGGGCCAAACCGTCCCCTACTTCAGCTCAAACACCGGATCAGGCGTCTTTGCATGCATTCGCCGATTCGCTGGAGCGTGACCTTGATGCGCCTAGCCCAATGCACGAGGGAGATCAGACAGGGGCAAGCAGCCGTAAACGGTCCCGATCGGATCGTGCTGTCACCAGCCCCTCCGCACAGCAATCTTTCGAGGTGCGCGTTCCCGAACAGCACGATGCGCTGCATTTGCCCCTCAGCTGGAGGGTAAAACGCCCGCGTACCAGGATCGGGGGCGGCCTCCCGGATCC

>***tal*-*Bam*HI-24 (*tal_Xoo_*_K74_-15)**.

GGATCCCATTCGTTCGCGCACGCCAAGTCCTGCCCGCGAGCCTCTGCCCGGACCCCAACCGGATAGGGTTCAGCCGACTGCAGATCGTGGGGTGTCTGCGCCTGCTGGCAGCCCTCTGGATGGCTTGCCCGCTCGGCGGACGGTGTCCCGGACCCGGCTGCCATCTCCCCCTGCCCCCTTGCCTGCGTTCTCGGCGGGCAGCTCCACCGATCGGCTCCGTCCGTTCGATCCGTCGCTTCCTGATACATCGCTTTTTGATTCGATGCCTGCCGTCGGCACGCCTCATACAGAGGCTGCCCCAGCAGACACTTCGCCGGCCGCGCAGGTGGATCTACTCACGCTCGCGACAGTGGCGCAGCACCACGAGGCACTGGTGGGCCATGGGTTTACACACGCGCACATCGTTGCGCTCAGCCAACACCCGGCAGCGTTAGGGACCGTTGCTGTCACGTATCAAGACATAATCACGGCGTTGCCAGAGGCGACACACGAAGACATCGTTGGCGTCGGCAAACAGTTGTCCGGCGCACGCGCCCTGGAGGCCTTGCTCACGAAGGCGGGGGAGTTGAGAGGTCCGCCGTTACAGTTGGACACAGGCCAACTTCTCAAGATTGCAAGACGTGGCGGCGTGACCGCAGTGGAGGCAGTGCATGCATGGCGCAATGCACTGACGGGTGCCCCCCTGAACCTGACCCCGGACCAAGTGGTGGCCATCGCCAGCAATAGTGGCGGCAAGCAGGCGCTGGAGACGGTGCAGCGGCTGTTGCCGGTGCTGTGCCAGGACCATGGCCTGACCCCGGACCAGGTCGTGGCCATCGCCAGCCACGATGGCGGCAAGCAGGCGCTGGAGACGGTGCAGCGGCTGTTGCCGGTGCTGTGCCAGGACCATGGCCTGACCCCGGACCAGGTGGTGGCCATCGCCAGCAATGGCGGCGGCAAGCAGGCGCTGGAGACGGTGCAGCGGCTGTTGCCGGTGCTGTGCCAGGACCATGGCCTGACCCCGGACCAGGTGGTGGCCATCGCCAGCAATGGCGGCGGCAAGCAGGCGCTGGAGACGGTACTGTGCCAGGCCCATGGCCTGACCCCGGCGCAGGTGGTGGCCATCGCCAGCAATGGCGGCGGCAAGCAGGCGCTGGAGACGGTGCAGCGGCTGTTGCCGGTGCTGTGCCAGGCCCATGGCCTGACCCTGGACCAGGTCGTGGCCATTGCCAGCAATGGCGGCGGCAAGCAGGCGCTGGAGACGGTGCAGCGGCTGTTGCCGGTGCTGTGCCAGGCCCATGGCCTGACCCCGGACCAGGTGGTGGCCATCGCCAGCCACGATGGCGGCAAGCAGGCGCTGGAGACGGTGCAGCGGCTGTTGCCGGTGCTGTGCCAGGCCCATGGCCTGACCCCGGCCCAGGTGGTGGCCATCGCCAGCCACGATGGCGGCAAGCAGGCGCTGGAGACGGTGCAGCGGCTGTTGCCGGTGCTGTGCCAGGCCCATGGTCTGACCCTGGACCAGGTAGTGGCCATTGCCAGCCACGATGGCGGCAAGCAGGCGCTGGAGACGGTGCAGCGGCTGTTGCCGGTGCTGTGCCAGGCCCATGGTCTGACCCTGGACCAGGTAGTGGCCATTGCCAGCCACGATGGCGGCAAGCAGGCGCTGGAGACGGTGCAGCGGCTGTTGCCGGTGCTGTGCCAGGACCATGGTCTGACCCCGGCGCAGGTGGTGGCCATCGCCAGCAATAACGGCGGCAAGCAGGCGCTGGAGACGGTGCAGCGGCTGTTGCCGGTGCTGTGCCAGGACCATGGCCTGACCCCGGACCAGGTGGTGGCCATCGCCAGCCACGATGGCGGCAAGCAGGCGCTGGAGACGATGCAGCGGCTGTTGCCGGTGCTGTGCCAGGCCCATGGCCTGACCCCGGACCAGGTCGTGGCCATCGCCAGCAATGGCGGCGGCAAGCAGGCGCTGGAGACGGTGCAGCGGCTGTTGCCGGTGCTGTGCCAGGCCCATGGCCTGACCCCGGACCAGGTCGTGGCCATCGCCAGCCACGATGGCGGCAAGCAGGCGCTGGAGACGGTGCAGCGGCTGTTGCCGGTGCTGTGCCAGGACCATGGCCTGACCCCGGACCAGGTCGTGGCCATCGCCAGCAATATTGGCGGCAAGCAGGCGCTGGAGACGGTGCAGCGGCTGTTGCCGGTGCTGTGCCAGGACCATGGCCTGACCCCGGACCAGGTCGTGGCCATCGCCAGCCACGATGGCGGCAAGCAGGCGCTGGAGACGGTGCAGCGGCTGTTGCCGGTGCTGTGCCAGGACCATGGCCTGACCCCGGACCAGGTCGTGGCCATCGCCAACAATAACGGCGGCAAGCAGGCGCTGGAGACGGTGCAGCGGCTGTTGCCGGTGCTGTGCCAGGACCATGGCCTGACCCCGGACCAGGTCGTGGCCATCGCCAGCAATGGCGGCAAGCAGGCGCTGGAGAGCATTGTTGCCCAGTTATCTCGCCCTGATCCGGCGTTGGCCGCGTTGACCAACGACCACCTCGTCGCCTTGGCCTGCCTCGGCGGACGTCCTGCCCTGGATGCAGTGAAAAAGGGATTGCCGCACGCGCCGGAATTGATCAGAAGAGTCAATAGCCGTATTGGCGAACGCACGTCCCATCGCGTTGCCGACCTCGCGCACGTGGTGCGCGTGCTTGGTTTTTTCCAGAGCCACTCCCACCCAGCGCAAGCATTCGATGACGCCATGACGCAGTTCGGGATGAGCAGGCACGGGTTGGTACAGCTCTTTCGCAGAGTGGGCGTCACCGAATTCGAAGCCCGCTGCGGAACTATCCCCCCAGCCTCGCAGCGTTGGGACCGTATCCTCCAGGCATCAGGGACGAAAAGGGCCAAACCGTCCCCTACTTCAGCTCAGACGCCGGATCAGGCGTCTTTGCATGCATTCCCCGACTCGCTGGAGCGTGACCTTGATGCGCCCAGCCCAATGCACGAGGGAGATCAGACGCGGGCAAGCAGACGTAAACGGTCCTGATCGGATCGTGCTGTCACCGACCCCTCCGCACAGCAATCTTTCGAGGTGCGCGTTCCCGAACAGCACGATGCGCTGCATTTGCCCCTCAGCTGGAGGGTAAAACGCCCGCGTACCAGGATCGGGGGCGGCCTCCCGGATCC

>***tal*-*Bam*HI-26 (*tal_Xoo_*_K74_-16)**.

GGATCCCATTCGTTCGCGCACGCCAAGTCCTGCCCGCGAGCTTCTGCCCGGACCCCAACCGGATAGGGTTCAGCCGACTGCAGATCGGGGGGGGGCTCCGCCTGCTGGCGGCCCCCTGGATGGCTTGCCCGCTCGGCGGACGATGTCCCGGACCCGGCTGCCATCTCCCCCTGCGCCCTCGCCTGCGTTCTCGGCGGGCAGCTTCAACGATCTGCTCCGTCAGTTCGATCCGTCGCTTCTTGATACATCGCTTCTTGATTCGATGCCTGCCGTCGGCACGCCGCATACAGCGGCTGCCCCAGCAGAGTGGGATGAGGTGCAATCGGGTCTGCGTGCAGCCGATGACCCGCCACCCACCGTGCGTGTCGCTGTCACTGCCGCGCGGCCGCCGCGCGCAAAGCCGGCCCCGCGACGGCGTGCGGCGCAACCCTCCGACGCTTCGCCGGCCGCGCAGGTGGATCTACGCACGCTCGGCTACAGTCAGCAGCAGCAAGAGAAGATCAAACCGAAGGTGCGTTCGACAGTGGCGCAGCACCACGAGGCACTGGTGGGCCATGGTTTTACACACGCGCACATCGTTGCGCTCAGCCAACACCCGGCAGCGTTAGGGACCGTCGCTGTCAAGTATCAGCACATAATCACGGCGTTGCCAGAGGCGACACACGAAGACATCGTTGGCGTCGGCAAACAGTGGTCCGGCGCACGCGCCCTGGAGGCCTTGCTCACGAAGGCGGGGGAGTTGAGAGGTCCGCCGTTACAGTTGGACACAGGCCAACTTCTCAAGATTGCAAAACGTGGCGGCGTGACCGCAGTGGAGGCAGTGCATGCATCGCGCAATGCACTGACGGGTGCCCCCCTGAACCTGACCCCGGACCAAGTGGTGGCCATCGCCAGCAATATTGGCGGCAACCAGGCGCTGGAGACGGTGCAGCGGCTGTTGCCGGTGCTGTGCCAGGACCATGGCCTGACCCCGGACCAAGTGGTGGCCATCGCCAACAATAACGGCGGCAAGCAGGCGCTGGAGACGGTGCAGCGGCTGTTGCCGGTGCTGTGCCAGGCCCATGGCCTGACCCCGGACCAAGTGGTGGCCATCGCCAGCAATGGCGGCAAGCAGGCGCTGGAGACGGTGCAGCGGCTGTTGCCGGTGCTGTGCCAGGCCCATGGCCTGACCCCGGACCAGGTCGTGGCCATCGCCAGCAATGGCGGCGGCAAGCAGGCGCTGGAGACGGTGCAGCGGCTGTTGCCGGTGCTGTGCCAGGCCCATGGCCTGACCCCGGCCCAGGTGGTGGCCATCGCCAGCAATAGTGGCGGCAAGCAGGCGCTGGAGACGGTGCAGCGGCTGTTGCCGGTGCTGTGCCAGGACCATGGCCTGACCCCGGCCCAAGTGGTGGCCATCGCCAACAATAACGGCGGCAAGCAGGCGCTGGAGACGGTGCAGCGGCTGTTTCCGGTGCTGTGCCAGGACCATGGCCTGACCCCGGACCAGGTGGTGACCATCGCCAACAATAACGGCGGCAAGCAGGCGCTGGAGACGGTGCAGCGGCTGTTGCCGGTGCTGTGCCAGGCCCATGGCTTGATCCCGGACCAGGTGGTGGCCATCGCCAACAATAACGGCGGCAAGCAGGCGCTGGAGACGGTGCAGCGGCTGTTGCCGGTGCTGTGCCAGGCCCATGGCCTGACCCCGGCCCAAGTGGTGGCCATCGCCAGCAATATTGGCGGCAAGCAGGCGCTGGAGACGGTGCAGCGGCTGTTGCCGGTGCTGTGCCGGGCCCATGGCCTGACCCCGGCCCAAGTGGTGGCCATCGCCAACAATAACGGCGGCAAGCAGGCGCTGGAGACGGTGCAGCGGCTGTTGCCGGTGCTGTGCCAGGACCATGGCCTGACCCCGGATCAAGTGGTGGCCATCGCCAGCAATATTGGCGGCAAGCAGGCGCTGGAGACGGTGCAGCGCCTGTTGCCGGTGCTGTGCCAGGCCCATGGCCTGACCCCGGACCAGGTCGTGGCCATCGCCAGCAATGGCGGCGGCAAGCAGGCGCTGGAGACGGTGCAGCGGCTGTTGCCGGTGCTGTGCCAGGACCATGGCCTGACCCCGGACCAGGTCGTGGCCATCGCCGGCCACGATGGCGGCAAGCAGGCGCTGGAGACGGTGCAGCGGCTGTTGCCGGTGCTGTGCCAGGACCATGGCCTGACCCCGGACCAGGTCGTGGCCATCGCCAGCCACGATGGCGGCAAGCAGGCGCTGGAGACGGTGCAGCGGCTGTTGCCGGTGCTGTGCCAGGACCATGGCCTGACCCTGGACCAGGTGGTGGCCATCGCCAGCAATATTGGCGGCAAGCAGGCGCTGGAGACGGTGCAGCGGCTGTTGCCGGTGCTGTGCCAGGACCATGGCCTGACCCCGGACCAGGTCGTGGCCATCGCCAGCAATGGCGGCGGCAAGCAGGCGCTGGAGAGCATTGTTGCCCAGTTATCTCGCCCTGATCCGGCGTTGGCCGCGTTGACCAACGACCACCTCGTCGCCTTGGCCTGCCTCGGCGGACGTCCTGCCCTGGATGCAGTGAAAAAGGGATTGCCGCACGCGCCGGAATTGATCAGAAGAATCAATCGCCGTATTCCCGAACGCACGTCCCATCGCGTTGCCGACTACGCGCAAGTGGTTCGCGTGCTGGAGTTTTTCCAGTGCCACTCCCACCCAGCGTACGCATTTGATGAGGCCATGACGCAGTTCGGGATGAGCAGGAACGGGTTGGTACAGCTCTTTCGCAGAGTGGGCGTCACCGAACTCGAAGCCCGCGGTGGAACGCTCCCCCCAGCCTCGCAGCGTTGGGACCGTATCCTCCAGGCATCAGGGATGAAAAGGGCCAAACCGTCCCCTACTTCAGCTCAAACACCGGATCAGGCGTCTTTGCATGCATTCGCCGATTCGCTGGAGCGTGACCTTGATGCGCCTAGCCCAATGCACGAGGGAGATCAGACAGGGGCAAGCAGCCGTAAACGGTCCCGATCGGATCGTGCTGTCACCGGCCCCTCCGCACAGCAATCTTTCGAGGTGCGCGTTCCCGAACAGCGCGATGCGCTGCATTTGCCCCTCAGCTGGAGGGTAAAACGCCCGCGTACCAGGATCGGGGGCGGCCTCCCGGATCC

>***tal*-*Bam*HI-27 (*tal_Xoo_*_K74_-5)**.

GGATCCCATTCGTTCGCGCACGCCAAGTCCTGCCCGCGAGCTTCTGCCCGGACCCCAACCGGATAGGGTTCAGCCGACTGCAGATCGGGGGGGGGCTCCGCCTGCTGGCGGCCCCCTGGATGGCTTGCCCGCTCGGCGGACGATGTCCCGGACCCGGCTGCCATCTCCCCCTGCGCCCTCGCCTGCGTTCTCGGCGGGCAGCTTCAGCGATCCGCTGCGTCAGTTCGATCCGTCGCTTCTTGATACATCGCTTTTTGATTCGATGCCTGCCGTCGGCACGCCGCATACAGCGGCTGCCCCAGCAGAGTGGGATGAGGCGCAATCGGCTCTGCGTGCAGCCGATGACCCGCCACCCACCGTGCGTGTCGCTGTCACTGCCGCGCGGCCGCCGCGCGCCAAGCCGGCCCCGCGACGGCGTGCGGCGCAACCCTCCGACGCTTCGCCGGCCGCGCAGGTGGATCTACGCACGCTCGGCTACAGTCAGCAGCAGCAAGAGAAGATCAAACCGAAGGTGCGTTCGACAGTGGCGCAGCACCACGAGGCACTGGTGGGCCATGGGTTTACACACGCGCACATCGTTGCGCTCAGCAAACACCCGGCAGCGTTAGGGACCGTTGCTGTCACGTATCAGCACATAATCACGGCGTTGCCAGAGGCGACACACGAAGACATCGTTGGCGTCGGCAAACAGTGGTCCGGCGCACGCGCCCTGGAGGCCTTGCTCACGGATGCGGGGGAGTTGAGAGGTCCGCCGTTACAGTTGGACACAGGCCAACTTCTCAAGATTGCAAAACGTGGCGGCGTGACCGCAGTGGAGGCAGTGCATGCATCGCGCAATGCACTGACGGGTGCCCCCCTGAACCTGACCCCGGCACAGGTGGTGGCCATCGCCAGCAATAACGGCGGCAAGCAGGCGCTGGAGACGGTGCAGCGGCTGTTGCCGTTGCTGTGCCAGGCCCATGGCCTGACCCCGGCGCAGGTGGTGGCCATCGCCAGCCACGATGGCGGCAAGCAGGCACTGGAGACGGTGCAGCGGCTGTTGCCGGTGCTGTGCCAGGACCATGGCCTGACCCCGGACCAGGTGGTGGCCATCGCCAGCAATAGTGGCGGCAAGCAGGCGCTGGAGACGGTGCAGCGGCTGTTGCCGGTGCTGTGCCAGGACCATGGCCTGACACCGGACCAGGTGGTGGCCATCGCCAGCAATGGCGGCGGCAAGCAGGCGCTGGAGACGGTGCAGCGGCTGTTGCCGGTGCTGTGCCAGGACCATGGCCTGACCCCGGACCAGGTGGTGGCCATCGCCAGCCACGATGGCGGCAAGCAGGCGCTGGAGACGGTGCAGCGGCTGTTGCCGGTGCTGTGCCAGGACCATGGCCTGACCCCGGACCAATTGGTGGCCATCGCCAACAATAACGGCGGCAAGCAGGCGCTGGAGACGGTGCAGCGGCTGTTGCCGGTGCTGTGCCAGGACCATGGCCTGACCCCGGACCAGGTCGTGGCCATCGCCAGCAATGGCGGCAAGCAGGCGCTGGAGACAGTGCAGCGGCTGTTGCCGGTGCTGTGCCAGGACCATGGTCTGACCCCGGACCAGGTCGTGGCCATCGCCAGCAATATTGGCGGCAAGCAGGCGCTGGAGACGGTGCAGCGGCTGTTGCCGGTGCTGTGCCAGGCCCATGGTCTGACCCCGGCGCAGGTGGTGGCCATCGCCAGCCACGATGGCGGCAAGCAGGCGCTGGAGACGATGCAGCGGCTGTTGCCGGTGCTGTGCCAGGCCCATGGCCTGACCCCGGCGCAGGTGGTGGCCATCGCCAGCAATAGCGGCGGCAAGCAGGCGCTGGAGACGGTGCAGCGGCTGTTGCCGGTGCTGTGCCAGGCCCATGGCCTGACCCCGGCGCAGGTGGTGGCCATCGTCAGCCACGATGGCGGCAAGCAGGCGCTGGAGACGGTGCAGCGGCTGTTGCCGGTGCTGTGCCAGGCCCATGGTCTGACCCCGGACCAGGTGGTGGCCATCGCCAGCAATAACGGCGGCAAGCAGGCGCTGGAGACGGTGCAGCGGCTGTTGCCGGTGCTGTGCCAGGACCATGGCCTGACCCCGGACCAGGTGGTGGCCATCGCCAGCCACGATGGCGGCAAGCAGGCGCTGGGGACGGTGCAGCGGCTGTTGCCGGTGCTGTGCCAGGACCATGGCCTGACCCCGGACCAGGTGGTGGCCATCGCCAGCAATAACGGCGGCAAGCAGGCGCTGGAGACGGTGCAGCGGCTGTTGCCGGTGCTGTGCCAGGACCATGGCCTGACCCCGGACCAGGTGGTGGCCATCGCCAGCCACGATGGCGGTAAGCAGGCGCTGGAGACGGTGCAGCGGCTGTTGCCGGTGCTGTGCCAGGCCCATGGCCTGACCCCGGCGCAGGTGGTGGCCATCGCCAGCAATAACGGCGGCAAGCAGGCGCTGGAGACGGTGCAGCGGCTGTTGCCGGTGCTGTGCCAGGACCATGGTCTGACCCCGGACCAGGTGGTAGCCATCGCCAACAATAACGGCGGCAAGCAGGCGCTGGAGACGGTGCAGCGGCTGTTGCCAGTGCTGTGCCAGGCCCATGGCCTGACCCCGGCGCAGGTGGTGGCCATCGCCAGCAATAACGGCAGCAAGCAGGCGCTGGAGACGGTGCAGCGGCTGTTGCCGGTGCTGTGCCAGGCCCATGGCCTGACCCCGGCGCAGGTGGTGGCCATCGCCAGCAATAACGGCGGCAAGCAGGCGCTGGAGACGGTGCAGCGGCTGTTGCCGGTGCTGTGCCAGGCCCATGGTCTGACCCCGGACCAGGTGGTGGCCATCGCCAGCCACGATGGCGGCAAGCAGGCGCTGGAGACGGTGCAGCGGCTGTTGCCGGTGCTGTGCCAGGCCCATGGCCTGACCCCGGACCAGGTGGTGGCCATCGCCAGCAATAACGGCGGCAAGCAGGCGCTGGAGACGGTGCAGCGGCTGTTGCCGGTGCTGTGCCAGGCCCATGGCCTGACCCCGGACCAGGTGGTGGCCATCGCCAGCAATAACGGCGGCAAGCAGGCGCTGGAGACGGTGCAGCGGCTGTTGCCGGTGCTGTGCCAGGACCATGGCCTGACCCCGGCGCAGGTGGTGGCCATCGCCAGCCACGATGGCGGCAAGCAGGCGCTGGAGACGGTGCAGCGGCTGTTGCCGGTGCTGTGCCAGGACCATGGCCTGACCCCGGCGCAGGTGGTGGCCATCGCCAGCAATGGCGGCGGCAAGCAGGCGCTGGAGAGCATTGTTGCCCAGTTATCTCGCCCTGATCCGGCGTTGGCCGCGTTGACCAACGACCACCTCGTCGCCTTGGCCTGCCTCGGCGGACGTCCTGCCCTGGATGCAGTGAAAAAAGGATTGCCGCACGCGCCGGAATTGATCAGAAGAGTCAATAGCCGTATTGGCGAACGCACGTCCCATCGCGTTGCCGACTACGCGCAAGTGGTTCGCGTGCTGGAGTTTTTCCAGTGCCACTCCCACCCAGCGTACGCATTTGATGAGGCCATGACGCAGTTCGGGATGAGCAGGAACGGGTTGTTACAGCTCTTTCGCAGAGTGGGCGTCACCGAACTCGAAGCCCGCGGTGGAACGCTCCCCCCAGCCTCGCAGCGTTGGGACCGTATCCTCCAGGCATCAGGGATGAAAAGGGCCAAACCGTCCCCTACTTCAGCTCAAACGCCGGATCAGGCGTCTTTGCATGCATTCGCCGATTCGCTGGAGCGTGACCTTGATGCGCCCAGCCCAATGCACGAGGGAGATCAGACGCGGGCAAGCAGCCGTAAACGGTCCCGATCGGATCGTGCTGTCACCGGCCCCTCCGCACAGCAATCTTTCGAGGTGCGCGTTCCCGAACAGCGCGATGCGCTGCATTTGCCCCTCAGCTGGAGGGTAAAACGCCCGCGTACCAGGATCGGGGGCGGCCTCCCGGATCC

>***tal*-*Bam*HI-31 (*tal_Xoo_*_K74_-9)**.

GGATCCCATTCGTTCGCGCACGCCAAGTCCTGCCCGCGAGCTTCTGCCCGGACCCCAACCGGATAGGGTTCAGCCGACTGCAGATCGGGGGGGGGCTCCGCCTGCTGGCGGCCCCCTGGATGGCTTGCCCGCTCGGCGGACGATGTCCCGGACCCGGCTGCCATCTCCCCCTGCGCCCTCGCCTGCGTTCTCGGCGGGCAGCTTCAGCGATCTGCTCCGTCAGTTCGATCCGTCGCTTCTTGATACATCGCTTCTTGATTCGATGCCTGCCGTCGGCACGCCGCATACAGCGGCTGCCCCAGCAGAATGGGATGAGGCGCAATCGGGTCTGCGTGCAGCCGATGACCCGCCACCCACCGTGCGTGTCGCTGTCACTGCCGCGCGGCCGCCGCGCGCCAAGCCGGCCCCGCGACGGCGTGCGGCGCAACCCTCCGACGCTTCGCCGGCCGCGCAGGTGGATCTACGCACGCTCGGCTACAGTCAGCAGCAGCAAGAGAAGATCAAACCGAAGGTGCGTTCGACAGTGGCGCGGCACCACGAGGCACTGGTGGGTCATGGGTTTACACACGCGCACATCGTTGCGCTCAGCCAACACCCGGCAGCGTTAGGGACCGTTGCTGTCACGTATCAGGACATAATCAGGGCGTTGCCAGAGGCGACACACGAAGACATCGTTGGCGTCGGCAAACAGTGGTCCGGCGCACGCGCCCTGGAGGCCTTGCTCACGGAGGCGAGGGAGTTGAGAGGTCCGCCGTTACAGTTGGACACAGGCCAACTTCTCAAGATTGCAAAACGTGGAGGCGTGACCTCAGTGCAGGCAGTGCATGCATGGCGCAATGCACTGACGGGTGCCCCCCTGAACCTGACCCCGGACCAAGTGGTGGCCATCGCCAGCAATATTGGCGGCAAGCAGGCGCTGGAGACGGTACAGCGGCTGTTGCCGGTGCTGTGCCAGGCCCATGGCCTGACCCCGGACCAGGTGGTGGCCATCGCCAGCAATATTGGCGGCAAGCAGGCGCTGGAGACGGTGCAGCGGCTGTTGCCGGTGCTGTGCCAGGACCATGGCCTGACCCCGGACCAGGTGGTGGCCATCGCCAGCCATGGCGGCGGCAAGCAGGCGCTGGAGACGGTGCAGCGGCTGTTGCCGGTGCTGTGCCAGGACCATGGCCTGACCCCGGACCAGGTGGTGGCCATCGCCAGCAATATTGGCGGCAAGCAGGCGCTGGAGACGGTGCAGCGGCTGTTGCCGGTGCTGTGCCAGGACCATGGCCTGATCCCGGACCAGGTGGTGGCCATCGCCAGCAATATTGGCGGCAAGCAGGCGCTGGAGACGGTGCAGCGGCTGTTGCCGGTGCTGTGCCAGGACCATGGCCTGACCCCGGACCAGGTGGTGGCCATCGCCAGCAATAGTGGCGGCAAGCAGGCGCTGGAGACGGTGCAGCGGCTGTTGCCGGTGCTGTGCCAGGCCCATGGCCTGACCCCAGACCAAGTGGTGGCCATCGCCAGCCACGATGGCGGCAAGCAGGCGCTGGAGACGGTGCAGCGGCTGTTGCCGGTGCTGTGCCAGGACCATGGCCTGACCCCGGCCCAAGTGGTGGCCATCGCCAGCAATAACGGCGGCAAGCAGGCGCTGGAGACGGTGCAGCGGCTGTTGCCGGTGCTGTGCCAGGCCCATGGCCTGACCCCAGACCAAGTGGTGGCCATCGCCAGCCACGATGGCGGCAAGCAGGCGCTGGAGACGGTGCAGCGGCTGTTGCCGGTGCTGTGCCAGGACCATGGCCTGACCCCGGCCCAAGTGGTGGCCATCGCCAGCAATAGTGGCGGCAAGCAGGCGCTGGAGACGGTGCAGCGGCTGTTGCCGGTGCTGTGCCAGGACCATGGCCTGACCCCGGACCAGGTGGTGGCCATCGCCAGCAATGGCGGCGGCAAGCAGGCGCTGGAGACGGTGCAGCGGCTGTTGCCGGTGCTGTGCCAGGACCATGGCCTGACCCCGGACCAGGTCGTGGCCATCGCCAGCAGTAGCGGCGGCAAGCAGGCGCTGGAGACGGTGCAGCGGCTGTTGCCGGTGCTGTGCCAGGCCCATGGCCTGACCCCGGACCAGGTGGTGGCCATCGCCAGCCACGATGGCGGCAAGCAGGCGCTGGAGACGGTGCAGCGGCTGTTGCCGGTGCTGTGCCAGGACCATGGCCTGACCCCGGACCAGGTGGTGGCCATCGCCAGCAATATTGGCGGCAAGCAGGCGCTGGAGACGGTGCAGCGGCTGTTGCCGGTGCTGTGCCAGGACCATGGCCTGACCCCGGACCAGGTGGTGGCCATCGCCAGCAATATTGGCGGCAAGCAGGCGCTGGAGACGGTGCAGCGGCTGTTGCCGGTGCTGTGCCAGGACCATGGCCTGACCCCGGACCAGGTGGTGGCCATCGCCAACAATAACGGCGGCAAGCAGGCGCTGGAGACGGTGCAGCGGCTGTTGCCGGTGCTGTGCCAGGACCATGGCCTGACCCCGGACCAGGTGGTGGCCATCGCCAGCAATATTGGCGGCAAGCAGGCGCTGGAGACGGTGCAGCGGCTGTTGCCGGTGCTGTGCCAGGACCATGGCCTGACCCCGGCCCAGGTGGTGGCCATCGCCAACAATAACGGCGGCAAGCAGGCGCTGGAGACGGTGCAGCGGCTGTTGCCGGTGCTGTGCCAGGACCATGGCCTGACCCCGGACCAGGTGGTGGCCATCGCCAGCAATATTGGCGGCAAGCAGGCGCTGGAGACGGTGCAGCGGCTGTTGCCGGTGCTGTGCCAGGACCATGGCCTGACCCCGGACCAGGTGGTGGCCATCGCCAGCAATGGCGGCGGCAAGCAGGCGCTGGAGAGCATTGTTGCCCAGTTATCTCGCCCTGATCCGGCGTTGGCCGCGTTGACCAACGACCACCTCGTCGCCTTGGCCTGCCTCGGCGGACGTCCTGCCCTGGATGCAGTGAAAAAGGGATTGCCGCACGCGCCGGAATTGATCAAAAGAATCAATCGCCGCATTCCCGAACGCACGTCCCATCGCGTTGCCGACCTCGCGCACGTGGTGCGCGTGCTTGGTTTTTTCCAGAGCCACTCCCACCCAGCGCAAGCATTCGATGACGCCATGACGCAGTTCGGGATGAGCAGGCACGGGTTGGTACAGCTCTTTCGCAGAGTGGGCGTCACCGAATTCGAAGCCCGCTGCGGAACACTCCCCCCAGCCTCGCAGCGTTGGGACCGTATCCTCCAGGCATCAGGGATGAAAAGGGCCAAACCGTCCCCTACTTCAGCTCAAACGCCGGATCAGGCGTCTTTGCATGCATTCGCCGATTCGCTGGAGCGTGACCTTGATGCGCCCAGCCCAATGCACGAGGGAGATCAGACGCGGGCAAGCAGCCGTAAACGGTCCCGATCGGATCGTGCTGTCACCGGCCCCTCCACACAGCAATCTTTCGAGGTGCGCGTTCCCGAACAGCACGATGCGCTGCATTTGCCCCTCAGCTGGAGGGTAAAACGCCCGCGTACCAGGATCGGGGGCGGCCTCCCGGATCC

>***tal*-*Bam*HI-34 (*tal_Xoo_*_K74_-7)**.

GGATCCCATTCGTTCGCGCACGCCAAGTCCTGCCCGCGAGCTTCTGCCCGGACCCCAACCGGATAGGGTTCAGCCGACTGCAGATCGGGGGGGGGGGGCTCCGCCTGCTGGCGGCCCCCTGGATGGCTTGCCCGCTCGGCGGACGATGTCCCGGACCCGGCTGCCATCTCCCCCTGCGCCCTCGCCTGCGTTCTCGGCGGGCAGCTTCAGCGATCTGCTCCGTCAGTTCGATCCGTCGCTTCTTGATACATCGCTTCTTGATTCGATGCCTGCCGTCGGCACGCCGCATACAGCGGCTGCCCCAGCAGAGTGCGATGAGGTGCAATCGGGTCTGCGTGCAGCCGATGACCCGCCACCCACCGTGCGTGTCGCTGTCACTGCCGCGCGGCCGCCGCGCGCCAAGCCGGCCCCGCGGCGGCGTGCGGCGCAACCCTCCGACGCTTCGCCGGCCGCGCAGGTGGATCTACGCACGCTCGGCTACAGTCAGCAGCAGCAAGAGAAGATCAAACCGAAGGTGCGTTCGACAGTGGCGCAGCACCACGAGGCACTGGTGGGCCATGGGTTTACACACGCGCACATCGTTGCGCTCAGCCAACACCCGGCAGCGTTAGGGACCGTTGCTGTCACGTATCAGGACATAATCAGGGCGTTGCCAGAGGCGACACACGAAGACATCGTTGGCGTCGGCAAACAGTGGTCCGGCGCACGCGCCCTGGAGGCCTTGCTCACGAAGGCGGGGGAGTTGAGAGGTCCGCCGTTACAGTTGGACACAGGCCAACTTCTCAAGATTGCAAAACGTGGCGGCGTGACCGCAGTGGAGGCAGTGCATGCATGGCGCAATGCACTGACGGGTGCCCCCCTGAACCTGACCCCGGACCAAGTGGTGGCCATCGCCAGCAATATTGGCGGCAAGCAGGCGCTGGAGACGGTGCAGCGGCTGTTACCGGTGCTGTGCCAGGCCCATGGCCTGACCCCGGACCAGGTCGTGGCCATCGCCAGCCATGGCGGCGGCAAGCAGGCGCTGGAGACGGTGCAACGGCTGTTGCCGGTGCTGTGCCAGGACCATGGCCTGACCCCGGACCAGGTCGTGGCCATCGCCAGCAATATTGGCGGCAAGCAGGCGCTGGAGACGGTGCAGCGGCTGTTGCCGGTGCTGTGCCAGGACCATGGCCTGACCCCGGACCAGGTGATGGCCATCGCCAACAATAACGGCGGCAAGCAGGCGCTGGAGACGGTGCAGCGGCTGTTGCCGGTGCTGTGCCAGGACCATGGCCTGACCCCGGACCAGGTGATGGCCATCGCCAACAATAACGGCGGCAAGCAGGCGCTGGAGACGGTGCAGCGGCTGTTGCCGGTGCTGTGCCAGGACCATGGCCTGACCCCGGACCAGGTCGTGGCCATCGCCAGCAATATTGGCGGCAAGCAGGCGCTGGAGACGGTGCAGCGGCTGTTGCCGGTGCTGTGCCAGGACCATGGCCTGACCCCGACCCAAGTGATGGCCATCGCCAACAATAACGGCGGCAAGCAGGCACTGGAGACGGTGCAGCGGCTGTTGCCGGTGCTGTGCCAGGACCATGGCCTGACCCCGGACCAGGTCGTGGCCATCGCCAGCCACGATGGCGGCAAGCAGGCGCTGGAGACGGTGCAGCGGCTGTTGCCGGTGCTGTGCCAGGACCATGGCCTGACCCCGGCCCAGGTCGTGGCCATCGCCAGCAATAGTGGCGGCAAGCAGGCGCTGGAGACGGTGCAGCGGCTGTTGCCGGTGCTGTGCCAGGACCATGGCCTGACCCCGGACCAGGTCGTGGCCATCGCCAGCCACGATGGCGGCAAGCAGGCGCTGGAGACGGTGCAGCGGCTGTTGCCGGTGCTGTGCCAGGACCATGGCCTGACCCCGGACCAGGTGGTGGCCATCGCCAGCAATAGTGGCGGCAAGCAGGCGCTGGAGACGGTGCAGCGGCTGTTGCCGGTGCTGTGCCAGGACCATGGCCTGACCCCGGACCAGGTGGTGGCCATCGCCAGCAATAGTGGCGGCAAGCAGGCGCTGGAGACGGTGCAGCGGCTGTTGCCGGTGCTGTGCCAGGACCATGGCCTGACCCCGGCCCAGGTGGTGGCCATCGCCAGCAATAGTGGCGGCAAGCAGGCGCTGGAGACGGTGCAGCGGCTGTTGCCGGTGCTGTGCCAGGACCATGGCCTGACCCCGGACCAAGTCGTGGCCATCGCCAGCCACGATGGCGGCAAGCAGGCGCTGGAGACGGTGCAGCGGCTGTTGCCGGTGCTGTGCCAGGACCATGGCCTGACCCCGGACCAGGTCGTGGCCATCGCCAACAATAACGGCGGCAAGCAGGCGCTGGAGACGCTGCAGCGGCTGTTGCCGGTGCTGTGCCAGGACCATGGCCTGACCCCGGACCAAGTGGTGGCCATCGCCAGCCACGATGGCGGCAAGCAGGCGCTGGAGACGGTGCAGCGGCTGTTGCCGGTGCTGTGCCAGGACCATGGCCTGACCCCGGACCAGGTCGTGGCCATCGCCAGCAATGGCGGCGGCAAGCAGGCGCTGGAGACGGTGCAACGGCTGTTGCCGGTGCTGTGCCAGGACCATGGCCTGACCCCGGACCAGGTCGTGGCCATCGCCAGCCACGATGGCGGCAAGCAGGCGCTGGAGACGGTGCAGCGGCTGTTGCCGGTGCTGTGCCAGGACCATGGCCTGACCCCGGCCCAAGTGGTGGCCATCGCCAGCCACGATGGCGGCAAGCAGGCGCTGGAGACGGTGCAGCGGCTGTTGCCGGTGTTGTGCCAGGACCATGGCCTGACCCCGGACCAGGTGGTGGCCATCGCCAGCCACGATGGCGGCAAGCAGGCGCTGGAGACGGTGCAGCGGCTGTTGCCGGTGCTGTGCCAGGACCATGGCCTGACCCCGGACCAGGTGGTGGCCATCGCCAGCAATGGCGGCGGCAAGCAGGCGCTGAAGACGGTGCAGCGGCTGTTGCCGGTGCTGTGCCAGGACCATGGCCTGACCCCGGACCAAGTGGTGGCCATCGCCAGCAATGGCGGCGGCAAGCAGGCGCTGGAGAGCATTGTTGCCCAGTTATCTTGCCCTGATCCGGCGTTGGCCGCGTTGACCAACGACCACCTCGTCGCCTTGGCCTGCCTCGGCGGGCGTCCTGCCCTGGATGCAGTGAAAAAGGGATTGCCGCACGCGCCAGAATTGATCAGAAGAATCAATCGCCGCATTCCCGAACGCACGTCCCATCGCGTTGCCGACCTCGCGCACGTGGTTCGCGTGCTTGGTTTTTTCCAGAGCCACTCCCACCCAGCGCAAGCATTCGATGACGCCATGACGCAGTTCGGGATGAGCAGGCACGGGTTGGTACAGCTCTTTCGCAGAGTGGGCGTCACCGAATTCGAAGCCCGCTACGGAACGCTCCCCCCAGCCTCGCAGCGTTGGGACCGTATCCTCCAGGCATCAGGGATGAAAAGGGCCAAACCGTCCCCTACTTCAGCTCAAACGCCGGATCAGGCGTCTTTGCATGCATTCGCCGATTCGCTGGAGCGTGACCTTGATGCGCCCAGCCCAATGCACGAGGGAGATCAGACGCGGGCAAGCAGCCGTAAACGGTCCCGATCGGATCGTGCTGTCACCGACCCCTCCGCACAGCAATCTTTCGAGGTGCGCGTTCCCGAACAGCACGATACGCTGCATTTGCCCCTCAGCTGGAGGGTAAAACGCCCGCGTACCAGGATCGGGGGCGGCCTCCCGGATCC

>***tal*-*Bam*HI-36 (*tal_Xoo_*_K74_-8)**.

GGATCCCATTCGTTCGCGCACGCCAAGTCCTGCCCGCGAGCTTCTGCCCGGACCCCAACCGGATAGGGTTCAGCCGACTGCAGATCGGGGGGGGGCTCCGCCTGCTGGCGGCCCCCTGGATGGCTTGCCCGCTCGGCGGACGATGTCCCGGACCCGGCTGCCATCTCCCCCTGCGCCCTCGCCTGCGTTCTCGGCGGGCAGCTTCAGCGATCTGCTCCGTCAGTTCGATCCGTCGCTTCTTGATACATCGCTTCTTGATTCGATTCCTGCCGTCGGCACGCCGCATACAGCGGCTGCCCCAGCAGAGTGGGATGAGGTGCAATCGGGTCTGCGTGCAGCCGATGACCCGCCACCCACCGTGCGTGTCGCTGTCACTGCCGCGCGGCCGCCGCGCGCTAAGCCGGCCCCGCGACGGCGTGCGGCGCAACCCTCCGACGCTTCGCCGGCCGCGCAGGTGGATCTACGCACGCTCGGCTACAGTCAGCAGCAGCAAGAGAAGATCAAACCGAAGGTGCGTTCGACAGTGGCGCAGCACCACGAGGCACTGGTGGGCCATGGGTTTACACACGCGCACATCGTTGCGCTCAGCCAACACCCGGCAGCGTTAGGGACCGTTGCTGTCACGTATCAGGACATAATCAGGGCGTTGCCAGAGGCGACACACGAAGACATCGTTGGCGTCGGCAAACAGTGGTCCGGCGCACGCGCCCTGGAGGCCTTGCTCACGAAGGCGGGGGAGTTGAGAGGTCCGCCGTTACAGTTGGACACAGGCCAACTTCTCAAGATTGCAAAACGTGGCGGCGTGACCGCAGTGGAGGCAGTGCATGCATGGCGCAATGCACTGACGGGTGCCCCCCTGAACCTGACCCCGGACCAAGTGGTGGCCATCGCCAGCAATATTGGCGGCAACCAGGCGCTGGAGACGGTGCAGCGGCTGTTGCCGGTGCTGTGCCAGGCCCATGGCCTGACCCCGGACCAGGTCGTGGCCATCGCCAGCCATGGCGGCGGCAAGCAGGCGCTGGAGACGCTGCAGCGGCTGTTGCCGGTGCTGTGCCAGGACCATGGCCTGACCCCGGACCAGGTGGTGGCCATCGCCAGCAATATTGGCGGCAAGCAGGCGCTGGAGACGGTGCAGCGGCTGTTGCCGGTGCTGTGCCAGGACCATGGCCTGACCCCGGACCAGGTGGTGGCCATCGCCAGCAATGGCGGCGGCAAGCAGGCGCTGGAGACGCTGCAACGGCTGTTGCCGGTGCTGTGCCAGGCCCATGGCCTGACCCCGGACCAGGTGCTGGCCATCGCCAGCCATGGCGGCGGCAAGCAGGCGCTGGAGACGCTGCAGCGGCTGTTGCCGGTGCTGTGCCAGGACCATGGCCTGACCCCGGCCCAAGTGGTGGCCCTCGCCAGCCACGATGGCGGCAAGCAGGCGCTGGAGACGGTGCAGCGGCTGTTGCCGGTGCTGTGCCAGGACCATGGCCTGACCCCGGCCCAGGTGGTGGCCATCGCCAGCAATAGTGGCGGCAAGCAGGCGCTGGAGACGGTGCAGCGGCTGTTGCCGGTGCTGTGCCAGGACCATGGCCTGACCCCGGACCAGGTCGTGGCCATCGCCAGCAATGGCGGCGGCAAGCAGGCGCTGGAGACGGTGCAACGGCTGTTGCCGGTGCTGTGCCAGGACCATGGCCTGACCCCGGACCAGGTGGTGGCCATCGCCAGCCACGATGGCGGCAAGCAGGCGCTGGAGACGGTGCAGCGGCTGTTGCCGGTGCTGTGCCAGGACCATGGCCTGACCCCGGACCAGGTCGTGGCCATCGCCAACAATAACGGCGGCAAGCAGGCGCTGGAGACGGTGCAACGGCTGTTGCTGGTGCTGTGCCAGGCCCATGGCCTGACCCCGGACCAGGTCGTGGCCATCGCCAGCAATGGCGGCGGCAAGCAGGCGCTGGAGACGGTGCAACGGCTGTTGCCGGTGCTGTGCCAGGACCATGGCCTGACCCCGGACCAGGTGGTGGCCATCGCCAGCCATGGCGGCGGCAAGCAGGCGCTGGAGACGGTGCAGCGGCTGTTGCCGGTGCTGTGCCAGGACCATGGCCTGACCCCGGACCAGGTCGTGGCCATCGCCAGCAATGGCGGCGGCAAGCAGGCGCTGGAGACGGTGCAGCGGCTGTTGCCGGTGCTGTGCCAGGACCATGGCCTGACCCCGGACCAGGTGGTGGCCATCGCCAGCCACGATGGCGGCAAGCAGGCGCTGGAGACGGTGCAGCGGCTGTTGCCGGTGCTGTGCCAGGACCATGGCCTGACCCCGGACCAGGTGGTGGCCATCGCCAGCCATGGCGGCGGCAAGCAGGCGCTGGAGACAGTGCAGCGGCTGTTGCCGGTGCTGTGCCAGGACCATGGCCTGACCCCGGACCAGGTCGTGGCCATCGCCAGCCACGATGGCGGCAAGCAGGCGCTGGAGACGGTGCAGCGGCTGTTGCCGGTGCTGTGCCAGGACCATGGCCTGACCCCGGACCAGGTGGTGGCCATCGCCAGCCACGATGGCGGCAAGCAGGCGCTGGAGACGGTGCAGCGGCTGTTGCCGGTGCTGTGCCAGGACCATGGCCTGACCCTGGACCAGGTGGTGGCCATCGCCAGCAATATTGGCGGCAAGCAGGCGCTGGAGACGGTGCAGCGGCTGTTGCCGGTGCTGTGCCAGGACCATGGCCTGACCCCGGACCAGGTGGTGGCCATCGCCAACAATAACGGCGGCAAGCAGGCGCTGGAGACGGTGCAGCGGCTGTTGCCGGTGCTGTGCCAGGACCATGGCCTGACCCCGGACCAGGTGGTGGCCATCGCCAGCAATGGCGGCGGCAAGCAGGCGCTGGAGAGCATTGTTGCCCAGTTATCTTGCCCTGATCCGGCGTTGGCCGCGTTGACCAACGACCACCTCGTCGCCTTGGCCTGCCTCGGCGGACGTCCTGCCCTGGATGCAGTGAAAAAGGGATTGCCGCACGCGCCGGAATTGATCAGAAGAATCAATCGCCGCATTCCCGAACGCACGTCCCATCGCGTTGCCGACCTCCCCGAACGCACGTCCAATCGCGTTGCCGACCTCGCGCACGTGGTGCGCGTGCTTGGTTTTTTCCAGAGCCACTCCCACCCAGCGCAAGCATTCGATGACGCCATGACGCAGTTCGGGATGAGCAGGCACGGGTTGGTACAGCTCTTTCGCAGAGTGGGCGTCACCGAACTCGAAGCCCGCTGCGGAACGCTCCCCCCAGCCTCGCAGCGTTGGGACCGTATCCTCCAGGCATCAGGGATGAAAAGGGCCAAACCGTCCCCTACTTCAGCTCAAACACCGGATCAGGCGTCTTTGCATGCATTCGCCGATTCGCTGGAGCGTGACCTTGATGCGCCCAGCCCAATGCACGAGGGAGATCAGACGCGGGCAAGCAGCCGTAAACGGTCCCGATCGGATCGTGCTGTCACCGGCCCCTCCGCACAGCAATCTTTCGAGGTACGCGTTCCCGAACAGCGCGATGCGCTGCATTTGCCCCTCAGCTGGAGGGTAAAACGCCCGCGTACCAGGATCGGGGGCGGCCTCCCGGATCC

>***tal*-*Bam*HI-39 (*tal_Xoo_*_K74_-2)**

GGATCCCATTCGTTCGCGCACGCCAAGTCCTGCCCGCGAGCTTCTGCCCGGACCCCAACCGGATAGGGTTCAGCCGACTGCAGATCGGGGGGGGGCTCCGCCTGCTGGCGGCCCCCTGGATGGCTTGCCCGCTCGGCGGACGATGTCCCGGACCCGGCTGCCATCTCCCCCTGCGCCCTCGCCTGCGTTCTCGGCGGGCAGCTTCAGCGATCTGCTCCGTCAGTTCGATCCGTCGCTTCTTGATACATCGCTTCTTGATTCGATGCCTGCCGTCGGCACGCCGCATACAGCGGCTGCCCCAGCAGAGTGGGATGAGGTGCAATCGGGTCTGCGTGCAGCCGATGACCCGCCACCCACCGTGCGTGTCGCTGTCACTGCCGCGCGGCCGCCGCGCGCCAAGCCGGCCCCGCGACGGCGTGCGGCACAACCCTCCGACGCTTCGCCGGCCGCGCAGGTGGATCTACGCACGCTCGGCTACAGTCAGCAGCAGCAAGAGAAGATCAAACCGAAGGTGCGTTCGACAGTGGCGCAGCACCACGAGGCACTGGTGGGCCATGGGTTTACACACGCGCACATCGTTGCGCTCAGCCAACACCCGGCAGCGTTAGGGACCGTCGCTGTCAAGTATCAGCACATAATCACGGCGTTGCCAGAGGCGACACACGAAGACATCGTTGGCGTCGGCAAACAGTGGTCCGGCGCACGCGCCCTGGAGGCCTTGCTCACGAAGGCGGGGGAGTTGAGAGGTCCGCCGTTACAGTTGGACACAGGCCAACTTCTCAAGATTGCAAAACGTGGCGGCTTGACCTCAGTGCAGGCAGTGCATGCATGGCGCAATGCACTGACGGGTGCCCCCCTGAACCTGACCCCGGACCAAGTGGTGGCCATCGCCAGCAATATTGGCGGCAAGCAGGCGCTGGAGACGGTACAGCGGCTGTTGCCGGTGCTGTGCCAGGCCAATGGCCTGACCCCGGACCAGGTCGTGGCCATCGCCAGCCATGGCGGCGGCAAGCAGGCGCTGGAGACGGTGCAGCGCCTGTTGCCGGTGCTGTGCCAGGACCATGGCCTGACCCCGGACCAGGTCGTGGCCATCGCCAGCAATATTGGCGGCAAGCAGGCGCTGGAGACGGTGCAGCGGCTGTTGCCGGTGCTGTGCCAGGACCATGGCCTGACCCCGGACCAGGTCGTGGCCATCGCCAGCCATGGCGGCGGCAAGCAGGCGCTGGAGACGGTGCAGCGGCTGTTGCCGGTGCTGTGCCAGGACCATGGCCTGACCCCGGACCAGGTGGTGGCCATCGCCAGCAATATTGGCGGCAAGCAGGCGCTAGAGACGGTGCAGCGGCTGTTGCCGGTGCTGTGCCAGGCCCATGGCCTGACCCCGGACCAGGTGGTGGCCATCGCCAGCAATATTGGCGGCAAGCAGGCGCTGGAGACGGTGCAGCGGCTGTTGCCGGTGCTGTGCCAGGACCATGGCCTGACCCCGGCCCAGGTGGTGGCCATCGCCAGCAATATTGGCGGCAAGCAGGCGCTGGAGACGGTGCAGCGGCTGTTGCCGGTGCTGTGCCAGGACCATGGCCTGACCCCGGACCAAGTCGTGGCCATCGCCAGCCACGATGGCGGCAAGCAGGCGCTGGAGACGGTGCAGCGGCTGTTGCCGGTGCTGTGCCAGGACCATGGCCTGACCCCGGACCAGGTGGTGGCCATCGCCAACAATAACGGCGGCAAGCAGGCGCTGGAGACGGTGCAGCGGCTGTTGCCGGTGCTGTGCCAGGACCATGGCCTGACCCCGGACCAAGTCGTGGCCATCGCCAGCCACGATGGCGGCAAGCAGGCGCTGGAGACGGTGCAGCGGCTGTTGCCGGTGCTGTGCCAGGACCATGGCCTGACCCCGGACCAGGTCGTGGCCATCGCCAGCCACGATGGCGGCAAGCAGGCGCTGGAGACGGTGCAGCGGCTGTTGCCGGTGCTGTGCCAGGAACATGGCCTGACCCCGGCCCAAGTGGTGGCCATCGCCAGCCACGATGGCGGCAAGCAGGCGCTGGAGACGGTGCAGCGGCTGTTGCCGGTGCTGTGCCAGGACCATGGCCTGACCCCGGACCAGGTGGTGGCCATCGCCAGCAATGGCGGCGGCAAGCAGGCGCTGGCGACGGTGCAGCGGCTGTTGCCGGTGCTGTGCCAGGCCCATGGCCTGACCCCGGACCAGGTCGTGGCCATCGCCAGCCACGATGGCGGCAAGCAGGCGCTGGAGACGGTGCAGCGGCTGTTGCCGGTGCTGTGCCAGGCCAATGGCCTGACCCCGGACCAGGTCGTGGCCATCGCCAGCAATGGCGGCAAGCAGGCGCTGGAGACGGTGCAGCGGCTGTTGCCTGTACAGCGGCTGTTGCCGGTGCTGTGCCAGGACCATGGCCTGACCCAGGACCAGGTGGTGGCCATCGCCAGCAATATTGGCGGCAAGCAGGCGCTGGAGACGGTGCAGCGGCTGTTGCCGGTGCTGTGCCAGGCCAATGGCCTGACCCAGGACCAGGTGGTGGCCATCGCCAGCCACGATGGCGGCAAGCAGGCGCTGGAGACGGTGCAGCGGCTGTTGCCGGTGCTGTGCCAGGACCATGGCCTGACCCCGGACCAGGTCGTGGCCATCGCCAGCCACGATGGCGGCAAACAGGCGCTGGAGACGGTGCAGCGGCTGTTGCCGGTGCTGTGCCAGGACCATGGCCTGACCCCGGCCCAGGTGGTGGCCATCGCCAACAATAACGGCGGCAAGCAGGCGCTGGAGACGGTGCAGCGGCTGTTGCCGGTGCTGTGCCAGGACCATGGCCTGACCCCGGACCAGGTCGTGGCCATCGCCAGCAATATTGGCGGCAAGCAGGCGCTGGCGACGGTGCAGCGGCTGTTGCCGGTGCTGTGCCAGGACCATGGCCTGACCCCGGACCAGGTCGTGGCCATCGCCAACAATAACGGCGGCAAGCAGGCGCTGGAGACGGTGCAGCGGCTGTTGCCGGTGCTGTGCCAGGACCATGGCCTGACCCCGGCGCAGGTGGTGGCCATCGCCAGCAATAACGGCGGCAAGCAGGCGCTGGAGACGGTGCAGCGGCTGTTGCCGGTGCTGTGCCAGGACCATGGCCTGACCCTGGACCAGGTGGTGGCCATTGCCAGCAATGGCGGCAGCAAACAGGCGCTGGAGACGGTGCAGCGGCTGTTGCCGGTGCTGTGCCAGGACCATGGCCTGACCCCGGACCAAGTGGTGGCCATCGCCAACAATAACGGCGGCAAGCAGGCGCTGGAGACGGTGCAGCGGCTGTTGCCGGTGCTGTGCCAGGACCATGGCCTGACCCTGGACCAGGTGGTGGCCATCGCCAGCCACGATGGCGGCAAGCAGGCGCTGGAGACGGTGCAGCGGCTGTTGCCGGTGCTGTGCCAGGACCATGGCCTGACCCTGGACAAGGTGGTGGCCATCGCCAGCAATGGCGGCAAGCAGGCGCTGGAGACGGTGCAGCGGCTGTTGCCGGTGCTGTGCCAGGACCATGGCCTGACCCCGAACCAGGTGGTGGCCATCGCCAGCAATAGTGGCGGCAAGCAGGCGCTGGAGACGGTGCAGCGGCTGTTGCCGGTGCTGTGCCAGGACCATGGCCTGACCCCGAACCAGGTGGTGGCCATCGCCAGCAATGGCGGCAAGCAGGCGCTGGAGAGCATTGTTGCCCAGTTATCTCGCCCTGATCCGGCGTTGGCCGCGTTGACCAACGACCACCTCGTCGCCTTGGCCTGCCTCGGCGGACGTCCTGCCCTGGATGCAGTGAAAAAGGGATTGCCGCACGCGCCGGAATTGATCAGAAGAATCAATCGCCGCATTCCCGAACGCACGTCCCATCGCGTTCCCGACCTCGCGCACGTGGTTCGCGTGCTTGGTTTTTTCCAGAGCCACTCCCACCCAGCGCAAGCATTCGATGACGCCATGACGCAGTTCGAGATGAGCAGGCACGGCTTGGTACAGCTCTTTCGCAGAGTGGGCGTCACCGAATTCGAAGCCCGCTACGGAACGCTCCCCCCAGCCTCGCAGCGTTGGGACCGTATCCTCCAGGCATCAGGGATGAAAAGGGCCAAACCGTCCCCTACTTCAGCTCAAACACCGGATCAGGCGTCTTTGCATGCATTCGCCGATTCGCTGGAGCGTGACCTTGATGCGCCTAGCCCAATGCACGAGGGAGATCAGACAGGGGCAAGCAGCCGTAAACGGTCCCGATCGGATCGTGCTGTCACCGGCCCCTCCGCACAGCAATCTTTCGAGGTGCGCGTTCCCGAACAGCGCGATGCGCTGCATTTGCCCCTCAGCTGGAGGGTAAAACGCCCGCGTACCAGGATCGGGGGCGGCCTCCCGGATCC

>***tal*-*Bam*HI-40 (*tal_Xoo_*_K74_-1)**

GGATCCCATTCGTTCGCGCACGCCAAGTCCTGCCCGCGAGCCTCTGCCCGGACCCCAACCGGATAGGGTTCAGCCGACTGCAGATCGTGGGGTGTCTGCGCCTGCTGGCAGCCCTCTGGATGGCTTGCCCGCTCGGCGGACGGTGTCCCGGACCCGGCTGCCATCTCCCCCTGCCCCCTTGCCTGCGTTCTCGGCGGGCAGCTCCACCGATCGGCTCCGTCCGTTCGATCCGTCGCTTCCTGATACATCGCTTTTTGATTCGATGCCTGCCGTCGGCACGCCTCATACAGAGGCTGCCCCAGCAGACACTTCGCCGGCCGCGCAGGTGGATCTACTCACGCTCGCGACAGTGGCGCAGCACCACGAGGCACTGGTGGGCCATGGGTTTACACACGCGCACATCGTTGCGCTCAGCCAACACCCGGCAGCGTTAGGGACCGTTGCTGTCACGTATCAAGACATAATCACGGCGTTGCCAGAGGCGACACACGAAGACATCGTTGGCGTCGGCAAACAGTTGTCCGGCGCACGCGCCCTGGAGGCCTTGCTCACGAAGGCGGGGGAGTTGAGAGGTCCGCCGTTACAGTTGGACACAGGCCAACTTCTCAAGATTGCAAGACGTGGCGGCGTGACCGCAGTGGAGGCAGTGCATGCATGGCGCAATGCACTGACGGGTGCCCCCCTGAACCTGACCCCGGACCAAGTGGTGGCCATCGCCAGCAATAGTGGCGGCAAGCAGGCGCTGGAGACGGTGCAGCGGCTGTTGCCGGTGCTGTGCCAGGACCATGGCCTGACCCCGGACCAGGTCGTGGCCATCGCCAGCCACGATGGCGGCAAGCAGGCGCTGGAGACGGTGCAGCGGCTGTTGCCGGTGCTGTGCCAGGACCATGGCCTGACCCCGGACCAGGTCGTGGCCATCGCCAGCAATGGCGGCGGCAAGCAGGCGCTGGAGACGGTGCAGCGGCTGTTGCCGGTGCTGTGCCAGGACCATGGCCTGACCCCGGACCAGGTGGTGGCCATCGCCAGCAATGGCGGCGGCAAGCAGGCGCTGGAGACGGTACTGTGCCAGGCCCATGGCCTGACCCCGGCGCAGGTGGTGGCCATCGCCAGCAATGGCGGCGGCAAGCAGGCGCTGGAGACGGTGCAGCGGCTGTTGCCGGTGCTGTGCCAGGCCCATGGCCTGACCCTGGACCAGGTCGTGGCCATTGCCAGCAATGGCGGCGGCAAGCAGGCGCTGGAGACGGTGCAGCGGCTGTTGCCGGTGCTGTGCCAGGCCCATGGCCTGACCCCGGACCAGGTGGTGGCCATCGCCAGCCACGATGGCGGCAAGCAGGCGCTGGAGACGGTGCAGCGGCTGTTGCCGGTGCTGTGCCAGGCCCATGGCCTGACCCCGGCCCAGGTGGTGGCCATCGCCAGCCACGATGGCGGCAAGCAGGCGCTGGAGACGGTGCAGCGGCTGTTGCCGGTGCTGTGCCAGGCCCATGGTCTGACCCTGGACCAGGTAGTGGCCATTGCCAGCCACGATGGCGGCAAGCAGGCGCTGGAGACGGTGCAGCGGCTGTTGCCGGTGCTGTGCCAGGCCCATGGTCTGACCCTGGACCAGGTAGTGGCCATTGCCAGCCACGATGGCGGCAAGCAGGCGCTGGAGACGGTGCAGCGGCTGTTGCCGGTGCTGTGCCAGGACCATGGTCTGACCCCGGCGCAGGTGGTGGCCATCGCCAGCAATAACGGCGGCAAGCAGGCGCTGGAGACGGTGCAGCGGCTGTTGCCGGTGCTGTGCCAGGACCATGGCCTGACCCCGGACCAGGTGGTGGCCATCGCCAGCCACGATGGCGGCAAGCAGGCGCTGGAGACGGTGCAGCGGCTGTTGCCGGTGCTGTGCCAGGCCCATGGCCTGACCCCGGACCAGGTCGTGGCCATCGCCAGCCACGATGGCGGCAAGCAGGCGCTGGAGACGGTGCAGCGGCTGTTGCCGGTGCTGTGCCAGGACCATGGTCTGACCCCGGCGCAGGTGGTGGCCATCGCCAGCCACGATGGCGGCAAGCAGGCGCTGGAGACGGTGCAACGGCTGTTGCAGGTGCTGTGCCAGGACCATGGCCTGACCCCGGACCAGGTCGTGGCCATCGCCAGCCACGATGGCGGCAAGCAGGCGCTGGAGACGGTGCAGCGGCTGTTGCCGGTGCTGTGCCAGGACCATGGCCTGACCCCGGACCAGGTCGTGGCCATCGCCAACAATAACGGCGGCAAGCAGGCGCTGGAGACGGTGCAGCGGCTGTTGCCGGTGCTGTGCCAGGCCCATGGCCTGACCCCGGACCAGGTGGTGGCCATCGCCAGCCATGGCGGCAAGCAGGCGCTGGAGAGCATTGTTGCCCAGTTATCTCGCCGTGATCCGGCGTTGGCCGCGTTGACCAACGACCAACTCGTCGCCTTGGCCTGCCTCGGCGGACGTCCTGCCCCGCATTCAAGGAAGAGGAAATCGCATGATTGATGGAGCTATTGCCTCAGTGAGGCTCAGTCGGGGGACTATCTGAGCGACGTCCCCCCATCCTGAGTAGCAGTCGGGTTTAGAGTCCGGGGTTGATGATATCGCTATTGGCCAACTGCTTGGCATACGTTGCCGGCGTCATTGCGCCGATTGCTTTCTTTGGGCGGTGTTGGTTTTATTCGCGGCGCCAGCGTTTGATACGCCCAGGGTTCCGTAGACACTCAAGACCCTCGTTGCGCGTAGCGCCGTTGAAACTCTACAGGCGATAGGTTGCCGGTTGAACCATGACGGCGTTGTGGGTTGTAGAACATTTCGATGTCGTCGAACACCTCGGCGCGTGCGGCGTCCTTGGTGGGGTAGATCCGCCGCCTGATCCGCTTGCGTTTGAGCAGACCGAAGAAGCTCTCCACCGGGGCATTGTCATGGCAGTTGCCACGTCGACTCATGCTGCACACCAAACCGTGGGACACCAGGAAACTGCGCCAGTCATCGCTGGTGTAGACCGACCCTTGGTCCGAGTGAACCAGGCAACCGGCGCTGGGCTTGCGCCGCCACACCGCCGACAGCAAGGCCTGCACGACCAACTCGGTATCGGCTCGATCGCGCATCGCCCAGCCGACGACCTGCCTGGAAAACAGATCGATCACCACAGCCAGGTACATCCAGCCTTCATGCGTACGGATAAAGGTGAAGTCGCTCGCCCAGGCCGTATCCGGCTCGGTCACGTCGAACTGCCGATCCAGCAGGTTGGCTGCCGCCTTGCACGGCATTCCGCCATGGAAGCGCGGTTTGCGACCATAGCCCACCTGGGCACGCAGCCCATCGGCGCGCATCAGCCGATGCACCCGATGGCGACTGCAACGCTCACCCAGATCGCGCAGATCCTTGGCGATCTTGCGATGCCCATACACACTGCCGCTGGCCAGCCAGTGGTGCTTGATCAGCCCCAGCAGGCGTTCGTCTTCCTTGGCGCGCTCACTGTCGGGCGACTTTAGCCACGCGTCGTATCCAGCCCGGTTCACCCGCAACACCCGGCACATCGCGCACACCCTGAATTCTTCGCGGTGGGCTTGCATGAGGACGTACTTTGCCTTTACCCCTTGGCAAAGTACGCGACGGCCTTTTTTAGGATGTCGCGCTCCTCCGTCACCCGGCGCAACTCGGTCTTCAGCCGCCGAACCTCGGCGCTCTGGTCCACTTCGGCACGCTGCACCACGCCGGGCTTGCCGAACGTACGCAGCCAGGCGTACAGGCTGTGCGTGGTGACACCCAGCCGCTGTGCGACCTCTGCCACCTTGAAGCCACGATCGGTCACTTACCGGACCGCTTCGATCTTGAACTCATCCGTATACCGCTTGCTGCTCATGGACACCTCCGAATCAGTCATTTTCTATGACCTTGAGATGTCTAGGAAACACTGGGCGTATCACTTCTGTCATCCCGCCTAACGGGTTGTCAGAGCTTCCGCTCCTTCCTACCGGCATGGCATGGCGGAATCCGGTGCGGGACTACGACTATGATCGGTCGGAGTGGCAGATCTACATTGTCTTACGACACGCATCGGTAGATCTGGCGCGCGTCAATCAGCTTGCCGAACCATCCGGCACCACGGCGGCAAGCGCAAGGTCAACGGCGTGGTGACCGCCGTGCGGCAGTTGGGCGCCGATGGCGGCTTGTCCAGCTACCGCTTGCGGATACAGCCCGCGCTGGCGCTGTTGGCCTATCGCCGCACCTGCCGCATCTTCCAGGAGGAGAGCGTGCCGGACATCGTCGCGCAGATCGTGCAGGAACATCGCGCCAGCAATCCCCCGATCGCCGCCAGCTTCCGCCTGGACCAGCAGCTGCGCCAGCGACGTCCCCCCGAAGTTGCGTACTGCCATGCGTACTCGGAAGAGCATGTAGGGACCACAGACCGTTAGTGTTGAGGCAACCCAGGTTTCTGCAAAGAGGTATGCCTTATGGATCC

>***tal*-*Bam*HI-41 (*tal_Xoo_*_K74_-10)**

GGATCCCATTCGTTCGCGTACGCCAAGTCCTGCCCGCGAGCTTCTGCCCGGACCCCAACCGGATAGGGTTCAGCCGACTGCAGATCGGGGGGGGGCTCCGCCTGCTGGCGGCCCCCTGGATGGCTTGCCCGCTCGGCGGACGATGTCCCGGACCCGGCTGCCATCTCCCCCTGCGCCCTCGCCTGCGTTCTCGGCGGGCAGCTTCAGCGATCTGCTCCGTCAGTTCGATCCGTCGCTTCTTGATACATCGCTTCTTGATTCGATGCCTGCCGTCGGCACGCCGCATACAGCGGCTGCCCCAGCAGAGTGCGATGAGGTGCAATCGGGTCTGCGTGCAGCCGATGACCCGCCACCCACCGTGCGTGTCGCTGTCACTGCCGCGCGGCCGCCGCGCGCCAAGCCGGCCCCGCGACGGCGTGCGGCGCAACCCTCCGACGCTTCGCCGGCCGCGCAGGTGGATCTACGCACGCTCGGCTACAGTCAGCAGCAGCAAGAGAAGATCAAACCGAAGGTGCGTTCGACAGTGGCGCAGCACCACGAGGCACTGGTGGGCCATGGGTTTACACACGCGCACATCGTTGCGCTCAGCCAACACCCGGCAGCGTTAGGGACCGTTGCTGTCACGTATCAGGACATAATCAGGGCGTTGCCAGAGGCGACACACGAAGACATCGTTGGCGTCGGCAAACAGTGGTCCGGCGCACGCGCCCTGGAGGCCTTGCTCACGGAGGCGGGGGAGTTGAGAGGTCCGCCGTTACAGTTGGACACAGGCCAACTTCTCAAGATTGCAAAACGTGGCGGCGTGACCGCAGTGGAGGCAGTGCATGCATGGCGCAATGCACTGACGGGTGCCCCCCTGAACCTGACCCCGGACCAAGTGGTGGCCATCGCCAGCAATATTGGCGGCAACCAGGCGCTGGAGACGGTGCAGCGGCTGTTGCCGGTGCTGTGCCAGGCCCATGGCCTGACCCCGGACCAGGTCGTGGCCATCGCCAGCCATGGCGGCGGCAAGCAGGCGCTGGAGACGGTGCAGCGGCTGTTGCCGGTGCTGTGCCAGGACCATGGCCTGACCCCGGACCAGGTGGTGGCCATCGCCAGCAATATTGGCGGCAAGCAGGCGCTGGAGACGGTGCAACGGCTGTTGCCGGTGCTGTGCCAGGACCATGGCCTGACCCCGGACCAGGTGGTGGCCATCGCCAGCAATATTGGCGGCAAGCAGGCGCTGGAGACGGTGCAGCGGCTGTTGCCGGTGCTGTGCCAGGACCATGGCCTGACCCCGGACCAGGTGGTGGCCATCGCCAGCAATATTGGCGGCAAGCAGGCGCTGGAGACGGTGCAGCGGCTGTTGCCGGTGCTGTGCCAGGACCATGGCCTGACCCCGGACCAGGTGGTGGCCATCGCCAACAATAACGGCGGCAAGCAGGCGCTGGAGACGGTGCAACGGCTGTTGCCGGTGCTGTGCCAGGACCATGGCCTGACCCCGGACCAGGTGGTGGCCATCGCCAGCCACGATGGCGGCAAGCAGGCGCTGGAGACGGTGCAGCGGCTGTTGCCGGTGCTGTGCCAGGGCCATGGCCTGACCCCGGACAAGGTGGTGGCCATCGCCAGCAATAGTGGCGGCAAGCAGGCGCTGGAGACGGTGCAGCGGCTGTTGCCAGTGCTGTGCCAGGCCCATGGCCTGACCCCGGACAAGGTGGTGGCCATCGCCAACAATAACGGCGGCAAGCAGGCGCTGGAGACGGTGCAGCGGCTGTTGCCGGTGCTGTGCCAGGACCATGGCCTGACCCCGGACCAGGTGGTGGCCATCGCCAGCAATAGTGGCGGCAAGCAGGCGCTGGAGACGGTGCAGCGGCTGTTGCCGGTGCTGTGCCAGGACCATGGTCTGACCCCGGCCCAGGTGGTGGCCATCGCCAACAATAACGGCGGCAAGCAGGCGCTGGAGACGGTGCGGCGGCTGTTGCCGGTGCTGTGCCAGGACCATGGCCTGACCCCGGACCAGGTCGTGGCCATCGCCAGCCACGATGGCGGCAAGCAGGCGCTGGAGACGGTGCAGCGGCTGTTGCCGGTACTGTGCCAGGACCATGGCCTGACCCCGGACCAGGTGGTGGCCATCGCCAACAATAACGGCGGCAAGCAGGCGCTGGAGACGGTGCAGCGGCTGTTGCCGGTGCTGTGCCAGGACCATGGCCTGACCCAGGACCAGGTGGTGGCCATCGCCAGCAATATTGGCGGCAAGCAGGCGCTGGAGACGGTGCAGCGGCTGTTGCCGGTGCTGTGCCAGGACCATGGCCTGACCCTGGACCAGGTGGTGGCCATCGCCAGCCACGATGGCGGCAAACAGGCGCTGGAGACGGTGCAGCGGCTGTTGCCGGTGCTGTGCCAGGACCATGGCCTGACCCCGGCCCAGGTGGTGGCCATCGCCAACAATAACGGCGGCAAGCAGGCGCTGGAGACGGTGCAGCGGCTGTTGCCGGTGCTGTGCCAGGACCATGGCCTGAGCCCGGACCAGGTCGTGGCCATCGCCAGCAATATTGGCGGCAAGCAGGCGCTGGAGACGGTGCAGCGGCTGTTGCCGGTGCTGTGCCAGGACCATGGCCTGACCCCGGACCAGGTGGTGGCCATCGCCAGCAATGGCGGCGGCAAGCAGGCGCTGGAGACGGTGCAGCGGCTGTTGCCGGTGCTGTGCCAGGACCATGGCCTGAGCCCGGACCAGGTCGTGGCCATCGCCAGCCATGATGGCGGCAAGCAGGCGCTGGAGACGGTGCAGCGGCTGTTGCCGGTGCTGTGCCAGGACCATGGCCTGACCCCGGACCAGGTGGTGGCCATCGCCAGCAATGGCGGCGGCAAGCAGGCGCTGGAGAGCATTGTTGCCCAGTTATCTCGCCCTGATCCGGCGTTGGCCGCGTTGACCAACGACCACCTCGTCGCCTTGGCCTGCCTCGGCGGACGTCCTGCCCTGGATGCAGTGAAAAAGGGATTGCCGCACGCGCCGGAATTGATCAGAAGAGTCAATAGCCGTATTGGCGAACGCACGTCCCATCGCGTTGCCGACTACGCGCAAGTGGTTCGCGTGCTGGAGTTTTTCCAGTGCCACTCCCACCCAGCGTACGCATTTGATGAGGCCATGACGCAGTTCGGGATGAGCAGGAACGGGTTGGTACAGCTCTTTCGCAGAGTGGGCGTCACCGAACTCGAAGCCCGCTGCGGAACGCTCCCCCCAGCCTCGCAGCGTTGGGACCGTATCCTCCAGGCATCAGGGATGAAAAGGGCCAAACCGTCCCCTACTTCAGCTCAAACACCGGATCAGGCGTCTTTGCATGCATTCGCCGATTCGCTGGAGCGTGACCTTGATGCGCCCAGCCCAATGCACGAGGGAGATCAGACGCGGGCAAGCAGCCGTAAACGGTCCCGATCGGATCGTGCTGTCACCGGCCCCTCCGCACAGCAATCTTTCGAGGTGCGCGTTCCCGAACAGCGCGATGCGCTGCATTTGCCCCTCAGCTGGAGGGTAAAACGCCCGCGTACCAGGATCGGGGGCGGCCTCCCGGATCC

>***tal*-*Bam*HI-47 (*tal_Xoo_*_K74_-17)**

GGATCCCATTCGTTCGCGTACGCCAAGTCCTGCCCGCGAGCTTCTGCCCGGACCCCAACCGGATAGGGTTCAGCCGACTGCAGATCGGGGGGGGGCTCCGCCTGCTGGCGGCCCCCTGGATGGCTTGCCCGCTCGGCGGACGATGTCCCGGACCCGGCTGCCATCTCCCCCTGCGCCCTCGCCTGCGTTCCCGGCGGGCAGCTTCAGCGATCTGCTCCGTCAAGTCGATACATCGCTTCTTGATTCGATGCCTGCCGTCGGCACGCCGCATACAGCGGCTGCCCCAGCAGAGTGGGATGAGATGCAATCGGGTCTGCGTGCAGCCGATGACCCGCCACCCACCGTGCGTGTCGCTGTCACTGCCGCGCGGCCGCCGCGCGCCAAGCCGGCCCCGCGACGGCGTGCGGCGCAACCCTCCGACGCTTCGCCGGCCGCGCAGGTGGATCTACGCACGCTCGGCTACAGTCAGCAGCAGCAAGAGAAGATCAAACCGAAGGTGCGTTCGACAGTGGCGCAGCACCACGAGGCACTGGTGGGCCATGGGTTTACACACGCGCACATCGTTGCGCTCAGCCAACACCCGGCAGCGTTAGGGACCGTCGCTGTCAAGTATCAGCACATAATCACGGCGTTGCCAGAGGCGACACACGAAGACATCGTTGGCGTCGGCAAACAGTGGTCCGGCGCACGCGCCCTGGAGGCCTTGCTCACGAAGGCGGGGGAGTTGAGAGGTCCGCCGTTACAGTTGGACACAGGCCAACTTCTCAAGATTGCAAAACGTGGCGGCGTGACCGCAGTGGAGGCAGTGCATGCATGGCGCAATGCACTGACGGGTGCCCCCCTGAACCTGACCCCGGACCAAGTGGTGGCCATCGCCAGCAATATTGGCGGCAAGCAGGCGCTGGAGACGGTGCAGCGGCTGTTGCCGGTGCTGTGCCAGGCCCATGGCCTGACCCCGGACCAGGTGGTGGCCATCGCCAACAATAACGGCGGCAAGCAGGCACTGGAGACGGTGCAGCGGCTGTTGCCGGTGCTGTGCCAGGCCCATGGCCTGACCCCGGCGCAGGTGGTGGCCATCGCCAGCAATAACGGCGGCAAGCAGGCGCTGGAGACGGTGCAGCGGCTGTTGCCGGTGCTGTGCCAGGACCATGGCCTGACCCCGGACCAAGTGGTGGCCATCGCCAGCAATATTGGCGGCAAGCAGGCGCTGGAGACGGTGCAACGGCTGTTGCCGGTGCTGTGCCAGGACCATGGCCTGACCCCGGACCAGGTCGTGGCCATCGCCAGCAATATTGGCGGCAAGCAGGCGCTGGAGACGGTGCAGCGGCTGTTGCCGGTGCTGTGCCAGACCCATGCCCTGACCCCGGACCAGGTGGTGGCCATCGCCAGCAATATTGGCGGCAAGCAGGCGCTGGAGACGGTGCAGCGGCTGTTGCCGGTGCTGTGCCAGGACCACGGCCTGACCCCGGCGCAGGTGGTGGCCATCGCCAGCCACGATGGCGGCAAGCAGGCGCTGGAGACGGTGCAGCGGCTGTTGCCGGTGCTGTGCCAGGACCATGGCCTGACCCCGGACCAGGTGGTGGCCATCGCCAGCAATAGTGGCGGCAAGCAGGCGCTGGAGACGGTGCAGCGGCTGTTGCCGGTGCTGTGCCAGGCCCATGGCCTGACCCTGGACCAGGTGGTGGCCATCGCCAGCCATGGCGGCGGCAAGCAGGCGCTGGAGACGGTGCAGCGGCTGTTGCCGGTGCTGTGCCAGGACCATGGCCTGACCCCGGACCAGGTGGTGGCCATCGCCAGCAATAACGGCGGCAAGCAGGCGCTGGAGACGGTGCAGCGGCTGTTGCCGGTGCTGTGCCAGGACCATGGCCTGATCCCGGACCAGGTGGTGGCCATCGCCAACAATAACGGCGGCAAGCAGGCGCTGGAGACGGTGCAGCGGCTGTTGCCGGTGCTGTGCCAGGCCCATGGCCTGACCACGGACCAGGTGGTGACCATCGCCAGCAATAACGGCGGCAAGCAGGCGCTGGAGACGGTGCAACGGCTGTTGCCGGTGCTGTGCCAGGACCATGGCCTGACCCCGGACCAGGTCGTGGCCATCGCCAGCAATATTGGCGGCAAGCAGGCGCTGGAGACGGTGCAGCGGCTGTTGCCGGTGCTGTGCCAGGACCATGGCCTGACCCCGGACCAGGTGGTGGCCATCGCCAGCAATATTGGCGGCAAGCAGGCGCTGGAGACGGTGCAGCGGCTGTTGCCGGTGCTGTGCCAGGACCATGGCCTGACCCCGGACCAGGTCGTGGCCATCGCCAGCAATGGCGGCGGCAAGCAGGCGCTGGAGACGGTGCAGCGGCTGTTGCCGGTGCTGTGCCAGGACCATGGCCTGACCCCGGACCAGGTGGTGGCCATCGCCAGCCACGATGGTGGCAAGCAGGCGCTGGAGAGCATTGTTGCCCAGTTATCTCGCCCTGATCCGGCGTTGGCCGCGTTGACCAACGACCACCTCGTCGCCTTGGCCTGCCTCGGCGGACGTCCTGCCCTGGATGCAGTGAAAAAGGGATTGCCGCACGCGCCGGAATTGATCAGAAGAATCAATCGCCGTATTCCCGAACGCACGTCCCATCGCGTTGCCGACTACGCGCAAGTGGTTCGCGTGCTGGAGTTTTTCCAGTGCCACTCCCACCCAGCGTACGCATTTGATGAGGCCATGACGCAGTTCGGGATGAGCAGGAACGGGTTGGTACAGCTCTTTCGCAGAGTGGGCGTCACCGAACTCGAAGCCCGCGGTGGAACGCTCCCCCCAGCCTCGCAGCGTTGGGACCGTATCCTCCAGGCATCAGGGATGAAAAGGGCCAAACCGTCCCCTACTTCAGCTCAAACACCGGATCAGGCGTCTTTGCATGCATTCGCCGATTCGCTGGAGCGTGACCTTGATGCGCCCAGCCCAATGCACGAGGGAGATCAGACGCGGGCAAGCAGCCGTAAACGGTCCCGATCGGATCGTGCTGTCACCGGCCCCTCCACACAGCAATCTTTCGAGGTGCGCGTTCCCGAACAGCGCGATGCGCTGCATTTGCCCCTCAGCTGGAGGGTAAAACGCCCGCGTACCAGGATCGGGGGCGGCCTCCCGGATCC

>***tal*-*Bam*HI-86(*tal_Xoo_*_K74_-3)**. GGATCCCATTCGTTCGCGCACGCCAAGTCCTGCCCGCGAGCTTCTGCCCGGACCCCAACCGGATAGGGTTCAGCCGACTGCAGATCGGGGGGGGGCTCCGCCTGCTGGCGGCCCCCTGGATGGCTTGCCCGCTCGGCGGACGATGTCCCGGACCCGGCTGCCATCTCCCCCTGCGCCCTCGCCTGCGTTCTCGGCGGGCAGCTTCAGCGATCTGCTCCGTCAGTTCGATCCGTCGCTTCTTGATACATCGCTTCTTGATTCGATGCCTGCCGTCGGCACACCGCATACAGCGGCTGCCCCAGCAGAGTGGGATGAGGTGCAATCGGGTCTGCGTGCAGCCGATGACCCGCCACCCACCGTGCGTGTCGCTGTCACTGCCGCGCGGCCGCCGCGCGCCAAGCCGGCCCCGCGACGGCGTGCGGCGCAACCCTCCGACGCTTCGCCGGCCGCGCAGGTGGATCTACGCACGCTCGGCTACAGTCAGCAGCAGCAAGAGAAGATCAAACCGAAGGTGCGTTCGACAGTGGCGCAGCACCACGAGGCACTGGTGGGCCATGGGTTTACACACGCGCACATCGTTGCGCTCAGCCAACACCCGGCAGCGTTAGGGACCGTCGCTGTCAAGTATCAGCACATAATCACGGCGTTGCCAGAGGCGACACACGAAGACATCGTTGGCGTCGGCAAACAGTGGTCCGGCGCACGCGCCCTGGAGGCCTTGCTCACGGAGGCGGGGGAGTTGAGAGGTCCGCCGTTACAGTTGGACACAGGCCAACTTCTCAAGATTGCAAAACGTGGCGGCGTGACCGCAGTGGAGGCAGTGCATGCATGGCGCAATGCACTGACGGGTGCCCCCCTGAACCTGACCCCGGACCAAGTGGTGGCCATCGCCAGCAATATTGGCGGCAAGCAGGCGCTGGAGACGGTGCAGCGGCTGTTGCCGGTGCTGTGCCAGGACCATGGCCTGACCCCGGACCAGGTCGTGGCCATCGCCAGCCATGGCGGCGGCAAGCAGGCGCTGGAGACGGTGCAGCGGCTGTTGCCGGTGCTGTGCCAGGACCATGGCCTGACCCCGGACCAGGTGGTGGCCATCGCCAGCAATATTGGCGGCAAGCAGGCGCTGGAGACGGTGCAGCGGCTGTTGCCGGTGCTGTGCCAGGACCATGGCCTGACCCCGGACCAGGTGGTGGCCATCGCCAGCAATATTGGCGGCAAGCAGGCGCTGGAGACGGTGCAGCGGCTGTTGCCGGTGCTGTGCCAGGACCATGGCCTGACCCCGGACCAGGTCGTGGCCATCGCCAGCCATGGCGGCGGCAAGCAGGCGCTGGAGACGGTGCAACGGCTGTTGCCGGTGCTGTGCCAGGCCCATGGCCTGACCCCGGACCAAGTCGTGGCCATCGCCAGGCACGATGGCGGCAAGCAGGCGCTGGAGACGGTGCAGCGGCTGTTGCCGGTGCTGTGCCAGGCCCATGGCCTGACCCCAGACCAAGTGGTGGCCATCGCCAACAATAACGGCGGCAAGCAGGCGCTGGAGACGGTGCAGCGGCTGTTGCCGGTGCTGTGCCAGGCCCATGGCCTGACCCCGGACCAAGTGGTGGCCATCGCCAGCCACGATGGCGGCAAGCAGGCGCTGGAGACGGTGCAGCGGCTGTTGCCGGTGCTGTGCCAGGCCCATGGCCTGACCCCGGACCAGGTGGTGGCCATCGCCAGCCACGATGGCGGCAAGCAGGCGCTGGAGACGGTGCAGCGGCTGTTGCCGGTGCTGTGCCAGGACCATGGCCTGACCCCGGACCAGGTGGTGGCCATCGCCAGCCACGATGGCGGCAAGCAGGCGTTGGAGACGGTGCAGCGGCTGTTGCCGGTGCTGTGCCAGGACCATGGCCTGACCCCGGACCAGGTCGTGGCCATCGCCAGCAATATTGGCGGCAAGCAGGCGCTGGAGACGGTGCAGCGGCTGTTGCCGGTGCTGTGCCAGGACCATGGCCTGACCCCGGACCAGGTGGTGGCCATCGCCAGCAATATTGGCGGCAAGCAGGCGCTGGAGACGGTACAGCGGCTGTTGCCGGTGCTGTGCCAGGACCATGGCCTGACCCCGGACCAGGTGGTGGCCATCGCCAACAATAACGGCGGCAAGCAGGCGCTGGAGACGGTGCAGCGGCTGTTGCCGGTACAGCGGCTGGTGCCGGTGCTGTGCCAGGACCATGGCCTGACCCAGGACCAGGTGGTGGCCATCGCCAGCAATATTGGCGGCAAGCAGGCGCTGGAGACGGTGCAGCGGCTGTTGCCGGTGCTGTACCAGGACCATGGCCTGACCCAGGACCAGGTGGTGGCCATCGCCAGCCACGATGGCGGCAAGCAGGCGCTGGAGACGGTGCAGCGGCTGTTGCCGGTGCTGTGCCAGGACCATGGCCTGACCCCGGACCAAGTGGTGGCCATCGCCAGCCACGATGGCGGCAAACAGGCGCTGGAGACGGTGCAGCGGCTGTTGCCGGTGCTGTGCCAGGAACATGGCCTGACCCCGGACCAAGTGGTGGCCATCGCCAGCCACGATGGCGGCAAGCAGGCGCTGGAGACGGTGCAGCGGCTGTTGCCGGTGCTGTGCCAGGACCATGGCCTGACCCCGGACCAGGTGGTGGCCATCGCCAGCCATGGCGGCGGCAAGCAGGCGCTGGGGACGGTGCAACGGCTGTTGCCGGTGCTGTGCCAGGACCATGGCCTGACCCCGGACCAAGTGGTGGCCATCGCCAACAATAACGGCGGCAAGCAGGCGCTGGAGACGGTGCAGCGGCTGTTGCCGGTGCTGTGCCAGGACCATGGCCTGACCCCGGACCAAGTGGTGGCCATCGCCAACAATAACGGCGGCAAGCAGGCGCTGGAGACGGTGCAGCGGCTGTTGCCGGTGCTGTGCCAGGCCCATGGCCTGACCCCGGACCAAGTGGTGGCCATCGCCAGCCACGATGGCGGCAAGCAGGCGCTGGAGACGGTGCAGCGGCTGTTGCCGGTGCTGTGCCAGGACCATGGCCTGACCCCGGCCCAGGTGGTGGCCATCGCCAGCAATAGCGGCGGCAAGCAGGCGCTGGAGACGGTGCAACGGCTGTTGCCGGTGCTGTGCCAGGACCATGGCCTGACCCCGGCCCAGGTCGTGGCCATCGCCAACAATAACGGCGGCAAGCAGGCGCTGGAGACGGTGCAGCGGCTGTTGCCGGTGCTGTGCCAGGACCATGGCCTGACCCCGGACCAGGTCGTGGCCATCGCCAGCCACGATGGCGGCAAGCAGGCGCTGGAGACGGTGCAGCGGCTGTTGCCGGTGCTGTGCCAGGACCATGGCCTGACCCCGGACCAGGTCGTGGCCATCGCCAGCAATGGCGGCGGCAAGCAGGCGCTGGCGACGGTGCAGCGGCTGTTGCCGGTGCTGTGCCAGGCCCATGGCCTGACCCCGGACCAGGTCGTGGCCATCGCCAGCAATAGTGGCGGCAAGCAGGCGCTGGAGACGGTGCAGCGGCTGTTGCCGGTGCTGTGCCAGGACCATGGCCTGACCCCGAACCAGGTGGTGGCCATCGCCAGCAATGGCGGCAAGCAGGCGCTGGAGAGCATTGTTGCCCAGTTATCTCGCCCTGATCCGGCGTTGGCCGCGTTGACCAACGACCACCTCGTCGCCTTGGCCTGCCTCGGCGGACGTCCTGCCCTGGATGCAGTGAAAAAGGGATTGCCGCACGCGCCGGAATTGATCAGAAGAATCAATCGCCGTATTCCCGAACGCACGTCCCATCGCGTTCCCGACCTCGCGCACGTGGTGCGCGTGCTTGGTTTTTTCCAGAGCCACTCCCACCCAGCGCAAGCATTCGATGACGCCATGACGCAGTTCGGGATGAGCAGGCACGGCTTGGTACAGCTCTTTCGCAGAGTGGGCGTCACCGAATTCGAAGCCCGCTACGGAACGCTCCCCCCAGCCTCGCAGCGTTGGGACCGTATCCTCCAGGCATCAGGGATGAAAAGGGCCAAACCGTCCCCTACTTCAGCTCAAACACCGGATCAGGCGTCTTTGCATGCATTCGCCGATTCGCTGGAGCGTGACCTTGATGCGCCCAGCCCAATGCACGAGGGAGATCAGACGCGGGCAAGCAGCCGTAAACGGTCCCGATCGGATCGTGCTGTCACCGACCCCTCCACACAGCAATCTTTCGAGGTGCGCGTTCCCGAACAGCGCGATGCGCTGCATTTGCCCCTCAGCTGGAGGGTAAAACGCCCGCGTACCAGGATCGGGGGCGGCCTCCCGGATCC

>***tal*-*Bam*HI-*talC* (*talC*)**

GGATCCCATTCGTCCGCGCGCGCCAAGTCCTGCCCGCGAGGTTCTGCCCGGCCCCCAACCGGATAGGGTTCAGCCGACTGCAGATCGTGGGGTGTCTGCGCCTGCTGGCAGCCCTCTGGATGGCTTGCCCGCTCGGCGGACGATGTCCCGGACCCGGCTGCCATCTCCCCCTGCCCCCTTGCCTGCGTTCTCGGCGGGCAGCTTCAGCGATCTGCTCCGTCAGTTCGATCCGTCGCTTCTTGATACATCGCTTTTTGATTCGATGCCTGCCGTCGGCACGCCTCATACAGAGGCTGCCCCAGCAGAGGGGGATGAGGTGCAATCGGCTCTGCGTGCAGCCGATGACCCGCCACCCACCGTGCGTGTCGCTGTCACTGCCGCGCAGGTGGATCTACGCACGCTCGGCTACAGTCAGCAGCAAGAGAAGATCAAACCGAATGTTCGTTCGACAGTGGCGCAGCACCACGAGGCACTGGTGGGCCATGGGTTTACACACGCGCACATCGTTGCGCTCAGCCGACACCCGGCAGCGTTAGGGACCGTCGCTGTCAAGTATCAGGACATGATCGCGGCGTTACCAGAGGCGACACACGAAGACATCGTTGGGGTCGGCAAACAGTGTTCCGGCGCACGCGCCCTGGAGGCCTTGCTCACGGTGGCGGGAGAGTTGAGAGGTCCACCGTTACAGTTGGACACAGGCCAACTTGTCAAGATTGCAAAACGTGGCGGCGTGACCGCAGTGGAGGCAGTGCATGCATCGCGCAATGCACTGACGGGTGCCCCCCTGAACCTGACCCCGGCACAGGTGGTGGCCATCGCCAGCAATAGCGGCGGCAAGCAGGCGCTGGAGACGGTGCAGCGGCTGTTGCCGGTGCTGTGCCAGGCCCATGGCCTGACCCCGGAGCAGGTGGTGGCCATCGCCAGCAATGGCGGCGGCAAGCAGGCGCTGGAGACGGTGCAGCGGCTGTTGCCGGTGCTGTGCCAGGCCCATGGCCTGACCCCGGCGCAGGTGGTGGCCATCGCCAGCAATAGCGGCGGCAAGCAGGCGCTGGAGACGGTGCAGCGGCTGTTGCCGGTGCTGTGCCAGGCCCATGGCCTGACCCCGGACCAGGTGGTGGCCATCGCCAGCCACGATGGCGGCAAGCAGGCGCTGGAGACGGTGCAGCGACTGTTGCCGGTGCTGTGCCAGGCCCATGGCCTGACCCCGGAGCAGGTGGTGGCCATCGCCAGCAATATTGGCGGCAAGCAGGCGCTGGAGACGGTGCAGCGGCTGTTGCCGGTGCTGTGCCAGGCCCATGGCCTGACCCCGGAGCAGGTGGTGGCCATCGCCAGCAATGGCGGCGGCAAGCAGGCGCTGGAGACGGTGCAGCGGTTGTTGCCGATGCTGTGCCAGGCCCATGGCCTGACCCCGGAGCAGGTGGTGGCCATCGCCAGCAATAACGGCGGCAAGCAGGCGCTGGAGACGGTGCAGCGGCTGTTGCCGGTGCTGTGCCAGGCCCATGGCCTGACCCCGGAGCAGGTGGTGGCCATCGCCAGCAATGGCGGCGGCAAGCAGGCGCTGGAGACGGTGCAGCGGCTGTTGCCGGTGCTGTGCCAGGCCCATGGCCTGACCCCGGACCAAGTGGTGGCCATCGCCAGCCACGATGGCGGCAAGCAGGCGCTGGAGACGGTGCAGCGGCTGTTGCCGGTGCTGTGCCAGGCCCATGGCCTGACCCCGGCGCAGGTGGTGGCCATCGCCAGCAATATTGGCGGCAAGCAGGCGCTGGAGACGGTGCGGCGGCTGTTGCCGGTGCTGTGCCAGGCCCATGGCCTGACCCCGGCGCAGGTGGTGGCCATCGCCAACAATAACGGCGGCAAGCAGGCGCTGGAGACGGTGCAGCGGCTGTTGCCGGTGCTGTGCCAGGCCCATGGCCTGACCCCGGAGCAGGTGGTGGCCATCGCCAGCAATGGCGGCAAGCAGGCGCTGGAGACGGTGCAGCGGCTGTTGCCGGTGCTGTGCCAGGCCCATGGCCTGACCCCGGAGCAGGTGGTGGCCATCGCCAGCAATATTGGCGGCAAGCAGGCGCTGGAGACGGTGCAGCGGCTGTTGCCGGTGCTGTGCCAGGCCCATGGCCTGACCCCGGAGCAGGTGGTGGCCATCGCCAGCAATAACGGCGGCAAGCAGGCCCTGGAGACGGTGCAGCGGCTGTTGCCGGTGCTGTGCCAGGCCCATGGCCTGACCCCGGACCAGGTGGTGGCCATCGCCAGCCACGATGGCGGCAAGCAGGCGCTGGAGACGGTGCAGCGGCTGTTGCCGGTGCTGTGCCAGGCCCATGGCCTGACCCTGGAGCAGGTGGTGGCCATCGCCAGCAATGGCGGCGGCAAGCAGGCGCTGGAGACGGTGCAGCGGCTGTTGCCGGTGCTGTGCCAGGCCCATGGCCTGACCCCGGCGCAGGTGGTGGCCATCGCCTGCAATATTGGCGGCAAGCAGGCGCTGGAGACGGTGCGGCGGCTGTTGCCGGTGCTGTGCCAGGCCCATGGCCTGACCCCGGCGCAGGTGGTGGCCATCGCCAACAATAACGGCGGCAAGCAGGCGCTGGAGACGGTGCAGCGGCTGTTGCCGGTGCTGTGCCAGGCCCATGGCCTGACCCCGGCGCAGGTGGTGGCCATCGCCAGCAATGGCGGCAAGCAGGCGCTGGAGACGGTGCAGCGGCTGTTGCCGGTGCTGTGCCAGGCCCATGGTCTGACCCCGGCGCAGGTGGTGGCCATCGCCAGCCACGATGGCGGCAAGCAGGCGTTGGAGACGGTGCAGCGGCTGTTGCCGGTGCTGTGCCAGGCCCATGGCCTGACCCCGGACCAGGTGGTGGCCATCGCCAGCAATAACGGCGGCAAGCAGGCGCTGGAGACGGTGCAGCGGCTGTTGCCGGTGCTGTGCCAGGCCCATGGCCTGACCCCGGAGCAGGTGGTGGCCATCGCCAGCAATGGCGGCGGCAAGCAGGCGCTGGAGAGCATTGTTGCCCAGTTATCTCGCCCTGATCCGGCGTTGGCCGCGTTGACCAACGACCACCTCGTCGCCTTGGCCTGCCTCGGCGGACGTCCTGCCCTGGATGCAGTGAAAAAGGGATTGCCGCACGCGCCGGAATTGATCAGAAGAGTCAATAGCCGTATTGCCGAACGCACGTCCGATCGCGTTACCGACTACGCGCAAGTGGTTCGCGTGCTGGAGTTTTTCCAGTGCCACTCCCACCCAGCGCACGCATTTGATGAGGCCATGACGCAGTTCGGTATGAGCAGGAACGGATTGTTACAGCTCTTTCGCAGAGTGGGCGTCACCGAACTCGAAGCCTGCGGTGGAACGCTCCCCCCAGCCTCGCAGCGTTGGCACCGTATCCTCCAAGCATCAGGGATGAAAAGTGCCAAACCGTCCTGTGCTTCGGCTCAAACGCCGGATCAGGCGTCTTTGCATGCATTCGCCGATTCGCCGGAGCGTGACCTTGATGCGCCCAGCCCAATGCACGAGGGAGATCAGACGCGGGCAAGCAGCCGTAAACGGTCCCGATCGGATCGTGCTGTCACCGGCCCCTCCGCACAGCAGGCTGTCGAGGTGCGCGTTCCCGAACAGCGCGATGCGCTGCATTTGCCCCTCAGCTGGAGTGTAAAACGCCCGCGTACCAGGATCGGGGGCGGCCTCCCGGATCC
